# Electrocatalyzed direct arene alkenylations without directing groups for selective late-stage drug diversification

**DOI:** 10.1038/s41467-023-39747-0

**Published:** 2023-07-15

**Authors:** Zhipeng Lin, Uttam Dhawa, Xiaoyan Hou, Max Surke, Binbin Yuan, Shu-Wen Li, Yan-Cheng Liou, Magnus J. Johansson, Li-Cheng Xu, Chen-Hang Chao, Xin Hong, Lutz Ackermann

**Affiliations:** 1 Wöhler Research Institute for Sustainable Chemistry (WISCh), Georg-August-Universität Göttingen, Göttingen, Germany; 2 Center of Chemistry for Frontier Technologies, Department of Chemistry, State Key Laboratory of Clean Energy Utilization, Zhejiang University, Hangzhou, China; 3 Medicinal Chemistry, Research and Early Development, Cardiovascular, Renal and Metabolism (CVRM), BioPharmaceuticals R&D, AstraZeneca, Gothenburg, Sweden; 4 Department of Organic Chemistry, Stockholm University, Stockholm, Sweden; 5 Beijing National Laboratory for Molecular Sciences, Beijing, PR China; 6 Key Laboratory of Precise Synthesis of Functional Molecules of Zhejiang Province, School of Science, Westlake University, Hangzhou, Zhejiang Province China; 7 German Centre for Cardiovascular Research (DZHK), Berlin, Germany

**Keywords:** Electrocatalysis, Synthetic chemistry methodology

## Abstract

Electrooxidation has emerged as an increasingly viable platform in molecular syntheses that can avoid stoichiometric chemical redox agents. Despite major progress in electrochemical C−H activations, these arene functionalizations generally require directing groups to enable the C−H activation. The installation and removal of these directing groups call for additional synthesis steps, which jeopardizes the inherent efficacy of the electrochemical C−H activation approach, leading to undesired waste with reduced step and atom economy. In sharp contrast, herein we present palladium-electrochemical C−H olefinations of simple arenes devoid of exogenous directing groups. The robust electrocatalysis protocol proved amenable to a wide range of both electron-rich and electron-deficient arenes under exceedingly mild reaction conditions, avoiding chemical oxidants. This study points to an interesting approach of two electrochemical transformations for the success of outstanding levels of position-selectivities in direct olefinations of electron-rich anisoles. A physical organic parameter-based machine learning model was developed to predict position-selectivity in electrochemical C−H olefinations. Furthermore, late-stage functionalizations set the stage for the direct C−H olefinations of structurally complex pharmaceutically relevant compounds, thereby avoiding protection and directing group manipulations.

## Abstract (web summary)

Electrochemistry has emerged as an increasingly viable tool in molecular synthesis. Here the authors realize electrocatalyzed C−H activations, with the aid of data science and artificial intelligence, towards selective alkenylations for late-stage drug diversifications.

## Introduction

**Fig. 1:**
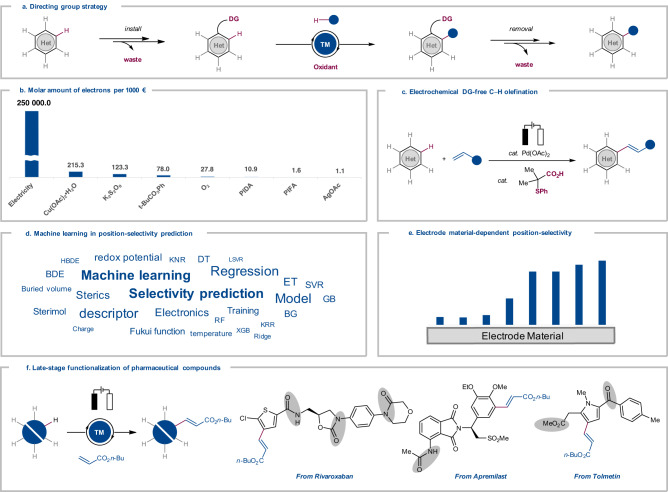
Directing Group (DG)-free Electrochemical C–H Activation. **a** Directing group (DG)-assisted oxidative C–H activation by installation and removal of DG. **b** Molar amount of electrons per 1000 euro from electricity and chemical oxidants. PIFA = (bis(trifluoroacetoxy)iodine)benzene. PIDA = (Diacetoxyiodo)benzene. **c** Electrochemical DG-free C–H olefination. **d** Machine learning in position-selectivity prediction. **e** Effects of the electrode material onto site-selectivity. **f** Late-stage functionalization of pharmaceutical molecules. (Potential DG was highlighted in gray).

In recent years, molecular electro-organic synthesis has surfaced as a uniquely effective toolbars for sustainable organic syntheses^1–4^. Despite indisputable progress^5–11^ by the merger of electrosynthesis and transition metal catalysis, electrochemical C–H activation^12–15^ has been largely restricted to the use of directing groups (DG)^16^ for the C–H functionalization. These DGs require additional steps for their installation and removal, contrasting the inherent efficacy of the C–H activation^17–21^ strategy (Fig. 1a). While the full control of position-selectivity constitutes a major challenge for synthetically useful C–H transformations^22–24^, recent advances^25^ in catalyst-controlled C–H activation^26–47^ have strongly relied on super-stoichiometric amounts of toxic and/or cost-intensive sacrificial chemical oxidants (Fig. 1b).

In this work, we disclose exogenous DG-free palladium-electrochemical C–H olefinations at low temperature without strong stoichiometric oxidants (Fig. 1c)^48^. Additionally, our machine learning (ML) modeling^49–54^ provide accurate and efficient prediction of the position-selectivity (Fig. 1d). Our approach is characterized by outstanding position-selectivity in olefinations of the electron-rich substrates by the judicious choice of electrode material (Fig. 1e). Notably, this strategy exhibits high functional group tolerance and unique position-selectivity undisturbed by potential DG, providing straightforward route for the late-stage C–H functionalization of structurally complex molecules of relevance to drug discovery and chemical biology (Fig. 1f).

## Results

We initiated our studies towards the non-directed electrochemical C–H activation by evaluating several *N*-type ligands^27,28^, (Supplementary Table 5). We were indeed delighted to observe electrocatalytic activity for *o*-xylene (**1h**) with 2-hydroxy-3-(trifluoromethyl)pyridine (**L1**) as the ligand, notably in the absence of chemical oxidants. Variation of the pyridone resulted in an increased efficacy with ligand **L3**. Protected amino acids **L5–L6** and 4,5-diazafluoren-9-one (**L7**) afforded inferior results in the electrocatalysis. Next, a representative set of sulfur-based ligands **L8–L12** was tested^40–42,46^, and we found an almost quantitative conversion to the olefination product **11** with *S,O*-ligand **L12** by the electrocatalysis.

While altering the stoichiometry did not have considerable influence on the efficacy (Supplementary Table 7, entry 2), the product **11** was not observed when *n*-Bu_4_NOAc was used as the supporting electrolyte (entry 3). Likewise, a solvent mixture of TFE and AcOH failed to provide the olefinated product **11** (entry 4). The electrocatalysis occurred efficiently in the absence of 1,4-benzoquinone (BQ), while catalytic amounts of BQ, working as redox mediator in our catalytic system rather than chemical oxidant, is suggested to prevent the aggregation of the palladium catalyst, thereby improving the catalyst’s performance (entry 5). Control experiments revealed the necessity of the palladium catalyst, the ligand and the electricity for the DG-free electrochemical C–H activation (entry 6–8). Importantly, scaling up allows to reduce the arene equivalent without any decrease of its efficacy (Supplementary Table 10, entry 1). Thus, the electrolysis for 48 h with 1.5 equiv. of arene and 1 equiv. of alkene along with catalytic amounts of Pd(OAc)_2_, ligand **L12**, and BQ, followed by addition of sodium acetate, in acetic acid and hexafluoroisopropanol delivers the olefinated product.

**Fig. 2:**
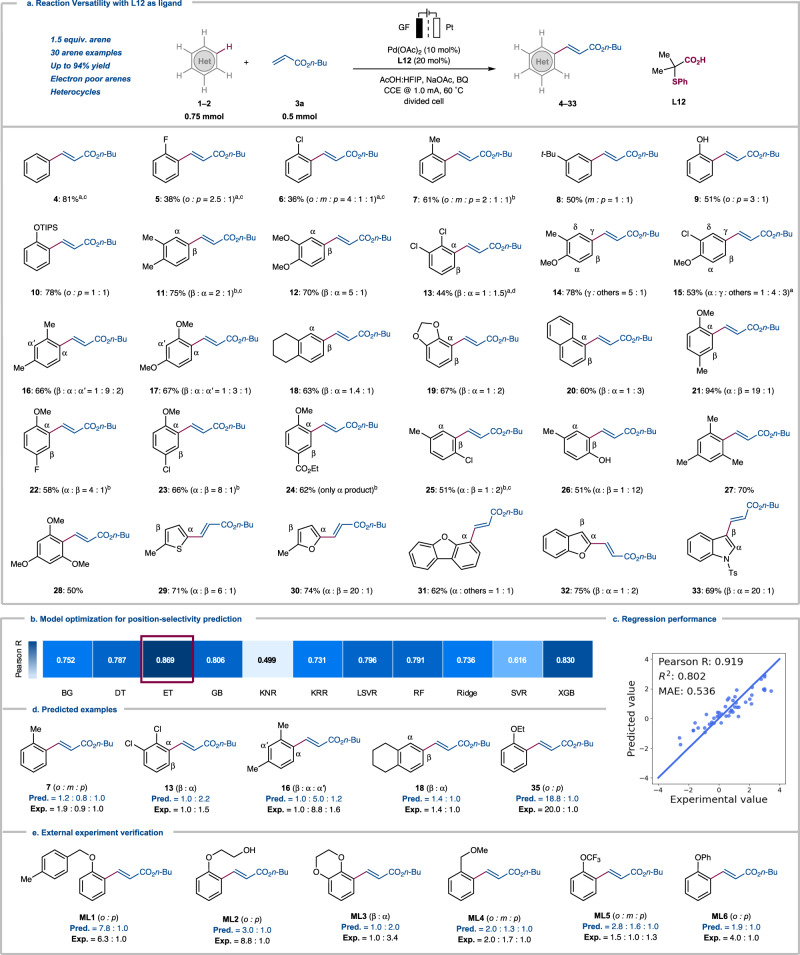
Substrate Scope and Machine Learning. See supplementary information for reaction details. **a** DG-Free Palladium-Electrochemical C–H Activation. General procedure **C**: divided cell, anodic chamber: **1**–**2** (0.75 mmol), **3a** (0.50 mmol), Pd(OAc)_2_ (10 mol %), **L12** (20 mol %), NaOAc (0.20 M), BQ (10 mol %), HFIP:AcOH (1:2); cathodic chamber: NaOAc (0.20 M), BQ (10 mol %), HFIP:AcOH (1:2), constant current at 1.0 mA, 60 °C, 48 h, graphite felt (GF) anode, Pt-plate cathode. ^a^ General procedure **A**, **1**–**2** (2.0 mmol or 4.0 mmol), **3a** (0.20 mmol). ^b^ **1**–**2** (1.0–1.5 mmol). ^c^ 80 °C. ^d^ 100 °C. **b** Machine learning model selection for position-selectivity prediction (Pearson R). The shadings represent the quality of the regression, the abbreviations embody the machine learning models. **c** Regression performances after descriptor selection. **d** Machine learning predictions for out-of-sample (OOS) examples. **e** Machine learning predictions for external experiment examples.

With the optimized electrolysis conditions in hand, we tested the robustness of the DG-free electrochemical olefination (Fig. 2a). We were pleased to find that both electron-rich and more challenging electron-poor arenes **1–2** delivered the mono-olefinated products in good to excellent yields by the palladium-electrocatalysis. Electron-deficient fluoro- and chloro-benzene **1b–1c** provided the mono-olefinated products **5–6** in moderate yields. Subsequently, a wide range of monosubstituted arenes **1d**–**1g** was selectively functionalized, including hydroxyl group OH-free, unprotected phenol **1f** with high yields (**9**). Disubstituted arenes were also examined under the optimized conditions, and for symmetrical disubstituted arenes, the position-selectivity was largely governed by repulsive steric interactions. Dimethoxybenzene (**1i**) yielded mainly the *β*-olefinated product **12**, while dichlorobenzene (**1j**) gave mainly *α*-olefinated product **13** instead. 1,2-Disubstituted arenes **1k–1l** were predominantly olefinated by the electrooxidation at the *γ*-position which is *para* to methoxy (**14–15**). 1,3-Disubstituted arenes **1m** and **1n** were predominantly olefinated at the *α*-position (**16–17**), while benzodioxole (**1p**) and naphthalene (**1q**) afforded predominantly the *α*-isomers (**19**–**20**). When *para*-substituted anisoles **1r–1u** were tested, the *ortho*-substituted anisole olefinated products **21–24** were obtained as the main products. A steric effect was predominant for *para*-chlorotoluene (**1v)** and *p*-cresol (**1w**), since we primarily observed *β*-isomers **25–26**. Furthermore, symmetrical trisubstituted arenes **1x** and **1y** were efficiently converted, delivering the mono-olefinated products **27** and **28**, respectively. It is noteworthy that the robust electrocatalysis was also viable for heteroarenes. Thus, thiophene (**1z**), furan (**2a**), benzofuran (**2b** and **2c**) and indole (**2d**) were efficiently olefinated by the palladium-electrocatalysis to yield the olefinated products **29–33** in the absence of chemical oxidants.

Next, in order to accurately predict the site-selectivity, we developed a ML model based on the collected position-selectivity data of all the arenes. A series of physical organic features including buried volume, Sterimol, Fukui function, charge, bond dissociation energies, etc. were applied to encode the involved molecules and enable the ML modeling (Supplementary Table 13). In addition to these site-specific descriptors, the computed redox potential of arenes were also included, considering the importance of electro-oxidation. These molecular descriptors, together with the reaction temperature, created a 28-dimensional encoding for each pair of regioisomeric competing sites, and an array of ML algorithms were evaluated for the regression performance in leave-one-out data splitting (Fig. 2b). The Extra-Trees (ET) model was found to provide the best performance in the position-selectivity prediction, and subsequent feature selection further improves the model’s prediction ability while decreasing the complexity of the descriptor space. The resulting ML model revealed a high level of accuracy (Pearson R = 0.919 and mean absolute error (MAE) = 0.536) (Fig. 2c). Feature importance elucidated the determining factors responsible for the regioselectivity prediction, in which the Fukui function of the reacting site emerged as the most crucial parameter (Supplementary Fig. 21). To further validate our model, we tested out-of-sample (OOS) predictions by taking selected arenes out of the training set. The model was revalidated without the access to the regioselectivity data of the selected arenes, and Fig. 2d highlighted a few examples of these excellent OOS predictions. Encouraged by the results, we further tested these predictions experimentally with 6 new arenes. Overall, our models align well with the experimental observations (Fig. 2e), which showed the predictive potential of the model in a rational way to reduce the experimental optimization.

**Fig. 3:**
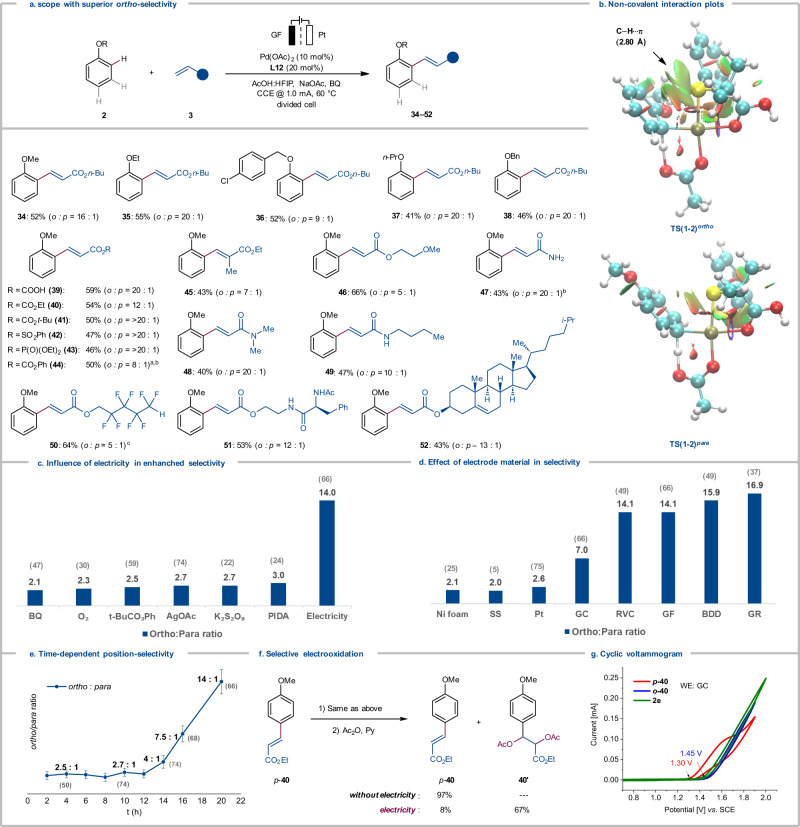
Mechanistic Study for Superior *Ortho*-selectivity. See supplementary information for reaction details. **a** Scope for anisole and olefin. General procedure **A**: divided cell, anodic chamber: **2** (1.0 mmol), **3** (0.20 mmol), Pd(OAc)_2_ (10 mol%), **L12** (20 mol%), NaOAc (0.20 M), BQ (20 mol%), HFIP:AcOH (1:2); cathodic chamber: NaOAc (0.20 M), BQ (20 mol%), HFIP:AcOH (1:2), constant current at 1.0 mA, 60 °C, 20 h, graphite felt (GF) anode, Pt-plate cathode. ^a^ General procedure **C**, **2** (1.0 mmol), **3** (0.5 mmol). ^b^ Pt as anode. ^c^ 80 °C. **b** Non-covalent interaction plots for the TS(1-2)^*ortho*^ and TS(1-2)^*para*^. **c** Chemical oxidants *vs* Electricity. **d** Variation of anode materials. RVC Reticulated vitreous carbon, GF Graphite felt, BDD Boron doped diamond. GR Graphite rod. **e** *Ortho/para*-selectivity profile. Combined yields of *ortho*/*para*-**34** were given in the parenthesis. The error bars indicate the possible selectivity fluctuations generated from crude NMR analysis. **f** Selective electrooxidation of *p*-**40**. **g** Cyclic voltammogram for **40**, glass carbon was used as working electrode.

Thereafter, we examined the versatility of the electrocatalysis with regards to anisole derivatives **2** and alkenes **3** (Fig. 3a). Thus, anisole and ethoxy benzene provided high position-selectivity for the *ortho*-functionalized products **34** and **35**. It is noteworthy that these selectivities are complementary to ones previously observed with pyridine-based ligands, which gave *para*-olefinated products as the major isomer^27,31^. Similarly, (benzyloxy)benzene derivatives and propoxybenzene mirrored the selectivity, delivering mono-alkenylated products **36–38**. Likewise, a range of alkenes **3** was compatible with the versatile electrochemical conditions, providing an array of olefinated products **39–52**. Acrylates **3b–3d** including acrylic acid gave outstanding levels of *ortho*-selectivities to afford the products **39–41**. Similarly, *α*,*β*-unsaturated olefin **3e**–**3g** mirrored this position-selectivity, providing *ortho*-olefinated products **42–44** as the major isomers. *α*-substituted acrylates (**3h**) were also identified as amenable substrates (**45**). Primary, secondary and tertiary acrylamide (**3j–3l**) also well adapted to the superior position-selectivity. Furthermore, the mild nature of the palladium-electrocatalysis manifold allowed for the use of fluorinated alkene **3m,** *NH*-free amino acid derivative **3n** and bio-relevant cholesterol **3o** delivering the predominantly *ortho*-olefinated products **50–52**. Here, it is noteworthy that 5.0 equivalents of arene are needed to provide good *ortho*-selectivity.

To delineate the C–H activation elementary step, the potential energy profile was computed at the PBE0-D4/def2-TZVP + SMD(AcOH)//PBE0-D3BJ/def2-SVP level of theory. The formation of the *ortho*-product was found to be kinetically and thermodynamically preferred, with C–H activation barriers of 9.7 kcal mol^−1^ (TS(1-2)^*ortho*^) and 10.7 kcal mol^−1^ (TS(1-2)^*para*^) respectively (Supplementary Fig. 34a). Non-covalent interactions in the TS(1-2)^*ortho*^ further revealed the presence of a weak stabilization interaction between the anisole’s methoxy group and the *S,O*-ligand phenyl motif, which contributes to the preferential formation of the *ortho*-product (Fig. 3b).

To further understand the origin of the high position-selectivity with anisoles, we conducted detailed studies (Figs. 3c–g). Here, we observed significant improvement in position-selectivities under the electrochemical conditions compared to reactions with commonly employed chemical oxidants (Fig. 3c). Exploring different electrode materials revealed a remarkable dependence of the position-selectivity on the choice of the material, thereby altering the *ortho/para*-selectivity from 2:1 to 17:1 (Fig. 3d). For such sharp change in selectivities, time-resolved analysis revealed critical insights (Fig. 3e). The ratio of the *ortho/para* selectivity remained constant within the first 12 hours, followed by a considerable change thereafter in favor of the *ortho*-functionalized product. This observation was rationalized by a subsequent selective electrochemical oxidation of the alkene only in the *para*-olefinated product. It is noteworthy that the second oxidation occurred selectively after the alkene **3a** was fully consumed (Supplementary Table 21). The independently prepared *para*-olefinated product **40** was subjected to the standard electrochemical conditions, followed by acetoxylation, resulting in the formation of diacetate **40’** (Fig. 3f). Control experiments showed the essential role of the electricity for the two-fold electrooxidation. CV studies confirmed that the *para*-olefinated product is more labile to be oxidized than the *ortho*-olefinated product, and were well aligned with our experimental observation for electrode material dependence on position-selectivity (Fig. 3g & Supplementary Fig. 12). In contrast, a representative set of commonly employed chemical oxidants were tested such as BQ, AgOAc, K_2_S_2_O_8_, PIDA or TBHP for selective oxidation of the products (Supplementary Table 19). The results were unsatisfactory, highlighting crucial and unique role of electricity in the selective oxidation process.

**Fig. 4:**
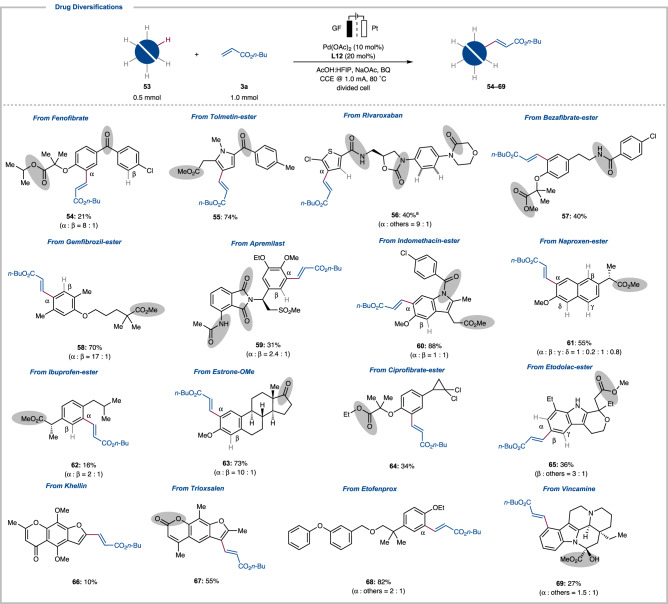
Late-Stage Functionalization of Drug Molecules. See supporting information for details. Brief reaction condition: **53** (0.5 mmol, 1.0 equiv.), **3a** (1.0 mmol, 2.0 equiv.), divided cell, 80 °C, 48 h. ^a^ **53** (0.36 mmol, 1.8 equiv.), **3a** (0.2 mmol, 1.0 equiv.). Potential coordinating directing groups are highlighted in gray.

Finally, the unique power of the DG-free electrocatalysis was exploited for the late-stage functionalization (LSF) of biorelevant drug molecules (Fig. 4)^19–21,47^. C–H olefination of fenofibrate proceeded efficiently to afford the olefinated product **54**. Tolmetin was selectively functionalized at the pyrrole ring to yield the mono-olefinated product **55** in 74% yield. Rivaroxaban was olefinated by the palladium-electrocatalysis to afford **56**. Bezafibrate was selectively converted to product **57**. At the same time, gemfibrozil was effectively converted into the corresponding alkenylated products **58**. Moreover, apremilast was transformed into two separable products **59**. Indomethacin was selectively olefinated to afford **60** in 88% yield at both *ortho*-positions of the anisole. Under the chemical oxidant-free electrocatalysis, naproxen afforded olefins **61**. Ibuprofen was functionalized to provide **62** and ester derivative of estrone were efficiently alkenylated at the *ortho*-position to afford **63**. Likewise, derivatives of ciprofibrate and etodolac provided the olefinated products **64** and **65**. Natural products like khellin and trioxsalen gave single products **66** and **67** under our reaction condition. Etofenprox similarly delivered the olefinated product **68** in 82% yield. Interestingly, vincamine was also tolerated and delivered product **69**. It is noteworthy to mention that our robust electrocatalysis enabled the LSF of complex drug molecules by overruling the presence of myriad of strongly coordinating directing groups ranging from ketone to amide and esters.

We have devised a robust and versatile electrochemical direct alkenylation without chemical oxidants and directing groups. The electrochemical olefination was realized by the synergistic cooperation of electricity, electrode material and a palladium catalyst. A broad variety of alkenes and arenes proved to be compatible with the electrooxidative catalysis. A two-fold electrochemical oxidation was uncovered, leading to outstanding levels of selectivity for anisole functionalization. Detailed studies showed the key effect of electricity and the electrode materials in the selectivity control, and machine learning modeling was developed to enable the data-driven position-selectivity prediction. The transformative nature of our strategy was highlighted by late-stage diversifications of bioactive drug molecules without the installation and removal of any directing groups. Additionally, the electrocatalysis yields molecular hydrogen as the only by product, representing a synthetically useful anodic oxidation to future green hydrogen technology by the hydrogen evaluation reaction (HER).

## Methods

### General Procedure: Non-directed Electrochemical Olefinations

The electrocatalysis was carried out in a divided cell, equipped with a GF anode and a Pt cathode (10 mm × 15 mm × 0.25 mm). Arenes (0.75 mmol, 1.5 equiv.), acrylates (0.50 mmol, 1.0 equiv.), Pd(OAc)_2_ (11.3 mg, 10 mol%), ligand (20 mol%), 1,4-benzoquinone (5.4 mg, 10 mol %) and NaOAc (50 mg, 0.20 M) were placed in the anodic chamber and dissolved in AcOH (2.0 mL) and HFIP (1.0 mL); 1,4-benzoquinone (5.4 mg, 10 mol%) and NaOAc (50 mg, 0.20 M) were placed in the cathodic chamber and dissolved in AcOH (2.0 mL) and HFIP (1.0 mL). Galvanostatic electrocatalysis was performed at 60 °C with a current of 1.0 mA and a stirring rate of 500 rpm maintained for 48 h. At ambient temperature, the resulting mixture was diluted with EtOAc (8.0 mL). The GF anode was washed with EtOAc (3 × 10 mL) in an ultrasonic bath. The combined organic phases were loaded on a column and washed with EtOAc (50 mL). The solvents were removed *in vacuo*. Then, NMR was determined by adding CH_2_Br_2_ (35.0 µL, 0.50 mmol, 1.0 equiv.) as the standard. The crude mixture was purified by flash column chromatography on silica gel to yield the products.

## Supplementary information

Supplementary Information

Peer Review File

Description of Additional Supplementary Files

Supplementary Data 1

## Data Availability

The authors declare that the data supporting the findings of this study are available within the paper and its Supplementary Information files. Cartesian Coordinates used for DFT calculation were included in Supplementary Data 1. All the involved codes and data in this study are freely available at 10.5281/zenodo.8003927. Source data are provided with this paper and deposited in Zenodo under accession code 10.5281/zenodo.8009809. All other requests for materials and information should be addressed to the corresponding authors.

## References

[1] Zhu C, Ang NWJ, Meyer TH, Qiu Y, Ackermann L (2021). Organic electrochemistry: molecular syntheses with potential. ACS Cent. Sci..

[2] Fu N, Sauer GS, Saha A, Loo A, Lin S (2017). Metal-catalyzed electrochemical diazidation of alkenes. Science.

[3] Xiong P, Xu H-C (2019). Chemistry with electrochemically generated N-centered radicals. Acc. Chem. Res..

[4] Yan M, Kawamata Y, Baran PS (2017). Synthetic organic electrochemical methods since 2000: on the verge of a renaissance. Chem. Rev..

[5] Malapit CA (2022). Advances on the merger of electrochemistry and transition metal catalysis for organic synthesis. Chem. Rev..

[6] Ma C (2021). Transition metal-catalyzed organic reactions in undivided electrochemical cells. Chem. Sci..

[7] Novaes LFT (2021). Electrocatalysis as an enabling technology for organic synthesis. Chem. Soc. Rev..

[8] Meyer TH, Choi I, Tian C, Ackermann L (2020). Powering the future: how can electrochemistry make a difference in organic synthesis?. Chem.

[9] Jiao K-J, Xing Y-K, Yang Q-L, Qiu H, Mei T-S (2020). Site-selective C–H functionalization via synergistic use of electrochemistry and transition metal catalysis. Acc. Chem. Res..

[10] Ackermann L (2020). Metalla-electrocatalyzed C–H activation by earth-abundant 3d metals and beyond. Acc. Chem. Res..

[11] Ma C, Fang P, Mei T-S (2018). Recent advances in C–H functionalization using electrochemical transition metal catalysis. ACS Catal..

[12] Dhawa U (2020). Enantioselective pallada-electrocatalyzed C−H activation by transient directing groups: expedient access to helicenes. Angew. Chem. Int. Ed..

[13] Yang Q-L (2017). Palladium-catalyzed C(sp^3^)–H oxygenation *vi*a electrochemical oxidation. J. Am. Chem. Soc..

[14] Kakiuchi F (2009). Palladium-catalyzed aromatic C−H halogenation with hydrogen halides by means of electrochemical oxidation. J. Am. Chem. Soc..

[15] Amatore C, Cammoun C, Jutand A (2007). Electrochemical recycling of benzoquinone in the Pd/Benzoquinone-catalyzed heck-type reactions from arenes. Adv. Synth. Catal..

[16] Sambiagio C (2018). A comprehensive overview of directing groups applied in metal-catalysed C–H functionalisation chemistry. Chem. Soc. Rev..

[17] Rogge T (2021). C–H activation. Nat. Rev. Methods Prim..

[18] He J, Wasa M, Chan KSL, Shao Q, Yu J-Q (2017). Palladium-catalyzed transformations of alkyl C–H bonds. Chem. Rev..

[19] Zhang L, Ritter T (2022). A perspective on late-stage aromatic C–H bond functionalization. J. Am. Chem. Soc..

[20] Guillemard L, Kaplaneris N, Ackermann L, Johansson MJ (2021). Late-stage C–H functionalization offers new opportunities in drug discovery. Nat. Rev. Chem..

[21] Cernak T, Dykstra KD, Tyagarajan S, Vachal P, Krska SW (2016). The medicinal chemist’s toolbox for late stage functionalization of drug-like molecules. Chem. Soc. Rev..

[22] Meng G (2020). Achieving site-selectivity for C–H activation processes based on distance and geometry: a carpenter’s approach. J. Am. Chem. Soc..

[23] Lewis JC, Coelho PS, Arnold FH (2011). Enzymatic functionalization of carbon–hydrogen bonds. Chem. Soc. Rev..

[24] Craven EJ (2021). Programmable late-stage C−H bond functionalization enabled by integration of enzymes with chemocatalysis. Nat. Catal..

[25] Wedi P, van Gemmeren M (2018). Arene-limited nondirected C−H activation of arenes. Angew. Chem. Int. Ed..

[26] Jia C, Kitamura T, Fujiwara Y (2001). Catalytic functionalization of arenes and alkanes *via* C−H bond activation. Acc. Chem. Res..

[27] Wang P (2017). Ligand-accelerated non-directed C–H functionalization of arenes. Nature.

[28] Zhang Y-H, Shi B-F, Yu J-Q (2009). Pd(II)-catalyzed olefination of electron-deficient arenes using 2,6-dialkylpyridine ligands. J. Am. Chem. Soc..

[29] Cook AK, Sanford MS (2015). Mechanism of the palladium-catalyzed arene C–H acetoxylation: a comparison of catalysts and ligand effects. J. Am. Chem. Soc..

[30] Cook AK, Emmert MH, Sanford MS (2013). Steric control of site selectivity in the Pd-catalyzed C–H acetoxylation of simple arenes. Org. Lett..

[31] Kubota A, Emmert MH, Sanford MS (2012). Pyridine ligands as promoters in Pd^II/0^-catalyzed C–H olefination reactions. Org. Lett..

[32] Emmert MH, Cook AK, Xie YJ, Sanford MS (2011). Remarkably high reactivity of Pd(OAc)_2_/pyridine catalysts: nondirected C–H oxygenation of arenes. Angew. Chem. Int. Ed..

[33] Izawa Y, Stahl SS (2010). Aerobic oxidative coupling of *o*-Xylene: discovery of 2-fluoropyridine as a ligand to support selective Pd-catalyzed C–H functionalization. Adv. Synth. Catal..

[34] Wedi P, Farizyan M, Bergander K, Mück-Lichtenfeld C, van Gemmeren M (2021). Mechanism of the arene-limited nondirected C−H activation of arenes with palladium. Angew. Chem. Int. Ed..

[35] Mondal A, van Gemmeren M (2021). Catalyst-controlled regiodivergent C−H alkynylation of thiophenes. Angew. Chem. Int. Ed..

[36] Farizyan M, Mondal A, Mal S, Deufel F, van Gemmeren M (2021). Palladium-catalyzed nondirected late-stage C–H deuteration of arenes. J. Am. Chem. Soc..

[37] Chen H, Farizyan M, Ghiringhelli F, van Gemmeren M (2020). Sterically controlled C−H olefination of heteroarenes. Angew. Chem. Int. Ed..

[38] Mondal A, Chen H, Flämig L, Wedi P, van Gemmeren M (2019). Sterically controlled late-stage C–H alkynylation of arenes. J. Am. Chem. Soc..

[39] Chen H, Wedi P, Meyer T, Tavakoli G, van Gemmeren M (2018). Dual ligand-enabled nondirected C−H olefination of arenes. Angew. Chem. Int. Ed..

[40] Sukowski V, Jia W-L, van Diest R, van Borselen M, Fernández-Ibáñez MÁ (2021). S,O-igand-promoted Pd-catalyzed C−H olefination of anisole derivatives. Eur. J. Org. Chem..

[41] Naksomboon K, Poater J, Bickelhaupt FM, Fernández-Ibáñez MÁ (2019). *para*-selective C–H olefination of aniline derivatives *via* Pd/S,O-ligand catalysis. J. Am. Chem. Soc..

[42] Jia W-L (2019). Selective C–H olefination of indolines (C5) and tetrahydroquinolines (C6) by Pd/S,O-ligand catalysis. Org. Lett..

[43] Naksomboon K, Valderas C, Gómez-Martínez M, Álvarez-Casao Y, Fernández-Ibáñez MÁ (2017). S,O-ligand-promoted palladium-catalyzed C–H functionalization reactions of nondirected arenes. ACS Catal..

[44] Ramadoss B, Jin Y, Asako S, Ilies L (2022). Remote steric control for undirected *meta*-selective C–H activation of arenes. Science.

[45] Kuninobu Y, Ida H, Nishi M, Kanai M (2015). A *meta*-selective C–H borylation directed by a secondary interaction between ligand and substrate. Nat. Chem..

[46] Gorsline BJ, Wang L, Ren P, Carrow BP (2017). C–H alkenylation of heteroarenes: mechanism, rate, and selectivity changes enabled by thioether ligands. J. Am. Chem. Soc..

[47] Zhao D, Xu P, Ritter T (2019). Palladium-catalyzed late-stage direct arene cyanation. Chemistry.

[48] Note: After the publication of this manuscript as a preprint: Ackermann, L. et al. Electrocatalyzed Direct Arene Alkenylations without Directing Groups: Selective Late-Stage Drug Diversification. *Research Square* (2022). [PREPRINT] 10.21203/rs.3.rs-1607467/v1, and during the revision, a study on non-directed palladium-catalysed electrooxidative olefination of arenes was published: Panja, S. et al. Non-directed Pd-catalysed electrooxidative olefination of arenes. *Chem. Sci*. **13**, 9432–9439, (2022).

[49] Ahneman DT, Estrada JG, Lin S, Dreher SD, Doyle AG (2018). Predicting reaction performance in C–N cross-coupling using machine learning. Science.

[50] Beker W, Gajewska EP, Badowski T, Grzybowski BA (2019). Prediction of major regio-, site-, and diastereoisomers in Diels–Alder reactions by using machine-learning: the importance of physically meaningful descriptors. Angew. Chem. Int. Ed..

[51] Reid JP, Proctor RSJ, Sigman MS, Phipps RJ (2019). Predictive multivariate linear regression analysis guides successful catalytic enantioselective minisci reactions of diazines. J. Am. Chem. Soc..

[52] Zahrt AF (2019). Prediction of higher-selectivity catalysts by computer-driven workflow and machine learning. Science.

[53] Li X, Zhang S-Q, Xu L-C, Hong X (2020). Predicting regioselectivity in radical C−H functionalization of heterocycles through machine learning. Angew. Chem. Int. Ed..

[54] Guan Y (2021). Regio-selectivity prediction with a machine-learned reaction representation and on-the-fly quantum mechanical descriptors. Chem. Sci..

