# Supplementary material for: Electrocatalyzed direct arene alkenylations without directing groups for selective late-stage drug diversification
Source: Nat Commun. 2023 Jul 15;14:4224. doi: 10.1038/s41467-023-39747-0 (PMC10349852; doi:10.1038/s41467-023-39747-0)
Supplement: Supplementary file 1 — Supplementary Information [file 41467_2023_39747_MOESM1_ESM.pdf]

Supplementary Information

Electrocatalyzed Direct Arene Alkenylations without Directing Groups for Selective Late-Stage Drug Diversification

Zhipeng Lin,+[1] Uttam Dhawa,+[1] Xiaoyan Hou,+[1] Max Surke,[1] Binbin Yuan,[1] Shu-Wen Li,[2] Yan-Cheng Liou,[1] Magnus J. Johansson,[3,4] Li-Cheng Xu,[2] Chen-Hang Chao,[2] Xin Hong*[2,5,6] Lutz Ackermann*[1,7]

Contents

Supplementary Methods 4

General Remarks 4

General Procedure A: Non-directed Electrochemical C–H Olefinations 5

General Procedure B: Non-directed Electrochemical C–H Olefinations 6

General Procedure C: Non-directed Electrochemical C–H Olefinations 7

General Procedure D: Non-directed Electrochemical C–H Olefinations 8

Syntheses of Ligands 9

Syntheses of Products 12

Heck Reaction 12

Knoevenagel Reaction 12

Product Characterization 13

Syntheses of Acrylate 17

Functional-group Protection for Drug Compounds 17

Carboxylic Acid Protecting Method 1 17

Phenol Protecting 17

Carboxylic Acid Protecting Method 2 17

Reaction Set-up 18

Reactor 18

Potentiostat 18

Supplementary Discussion 19

Optimization Studies 19

Optimization Based on Pyridone Ligand 19

Optimization Based on *S,O*-ligand 24

Role of BQ 26

Reduce the Loading of Arene 27

Miscellaneous Studies 28

Mono- and Di-functionalized Product 28

Other Metal Catalyst 29

Peroxide Test 29

Cyclic Voltammetric Studies 31

CV Studies of Pd and Ligand 31

CV Studies of Product Isomers 33

Machine Learning for Predicting Position-Selectivity 37
Details of the Dataset Used in Machine Learning 37
Structural Optimization 37
Details of Descriptors 38
Details of Machine Learning Algorithms 42
Details of Model Predictions 44
Feature Importance 51
Data and Code Availability 51
External Experimental Verification 52
Mechanistic Investigation for High Site-selectivity 57
Control Experiment 57
Two-fold Electrochemical Oxidation 61
Experiment for Switching Selectivity 64
Selective Oxidation of Other Arene 66
Characterization Data of Products 69
KIE Studies 119
Synthesis of the Palladium Complex 122
Computational Methods 123
NMR Spectrum 132
Supplementary References 254

Supplementary Methods

General Remarks

Catalytic reactions were carried out in a divided electrochemical cell using pre-dried glassware, if not noted otherwise. Arenes **1** or **2**, drug **53**, acrylates **3**, and ligands (**L1-L3**, **L5-L8**, **L13-L35**) were used as obtained by commercial sources. Other chemicals were obtained from commercial sources and were used without further purification. Platinum electrodes (10 mm × 15 mm × 0.25 mm, 99.9%; obtained from ChemPur® Karlsruhe, Germany) and Graphite felt (GF) electrodes (10 mm × 15 mm × 6 mm, SIGRACELL®GFA 6 EA, obtained from SGL Carbon, Wiesbaden, Germany) were connected using stainless steel adapters. Electrocatalysis was conducted using a Metrohm MULTI AUTOLAB M204 potentiostat in two-electrode constant current mode. Yields refer to isolated compounds, estimated to be >95% pure as determined by ^{1}H-NMR. Chromatography: Merck silica gel 60 (40–63 µm). NMR: Spectra were recorded on a Varian Unity 300, Mercury 300, Inova 500 or Bruker Avance III 300, Bruker Avance III HD 400 and Bruker Avance III HD 500 in the solvent indicated; chemical shifts (δ) are given in ppm relative to the residual solvent peak. All IR spectra were recorded on a Bruker FT-IR Alpha device. MS: EI-MS- and ESI-MS-spectra were recorded with Finnigan MAT 95, 70 eV and Finnigan LCQ; High resolution mass spectrometry (HRMS) with APEX IV 7T FTICR. M. p.: Stuart melting point apparatus SMP3, Barloworld Scientific, values are uncorrected.

General Procedure A: Non-directed Electrochemical C–H Olefinations

The electrocatalysis was carried out in a pre-dried divided cell, with a GF anode (10 mm × 15 mm × 6 mm) and a platinum cathode (10 mm × 15 mm × 0.25 mm). Arene **1-2**, or **53** (5.0–20 equiv), acrylates **3** (0.20 mmol, 1.0 equiv), $Pd(OAc)_2$ (4.5 mg, 10 mol %), **L3** (6.5 mg, 20 mol %) or **L12** (7.9 mg, 20 mol %), 1,4-benzoquinone (4.3 mg, 20 mol %) and NaOAc (66.0 mg, 4.0 equiv) were placed in the anodic chamber and dissolved in AcOH (2.6 mL) and HFIP (1.3 mL). 1,4-Benzoquinone (4.3 mg, 20 mol %) and NaOAc (66.0 mg, 4.0 equiv) were placed in the cathodic chamber and dissolved in AcOH (2.6 mL) and HFIP (1.3 mL). Electrocatalysis was performed at 60 °C with a constant current of 1.0 mA and a stirring rate of 500 rpm maintained for 20 h. At ambient temperature, the reaction mixture was diluted with EtOAc (5.0 mL). The GF anode was washed with EtOAc (3 × 10 mL) in an ultrasonic bath and the washings were added to the reaction mixture. The resulting mixture was loaded in a column chromatography with a pad of silica and washed with 50 mL EtOAc. The combined solvents were removed *in vacuo*. The NMR yield was determined by adding CH_2Br_2 (14.0 µL, 0.20 mmol, 1.0 equiv) as internal standard. The crude mixture was purified by flash column chromatography on silica gel to yield the products **4-52, 54-69, MS1-6**. The isomer ratios were measured by ^{1}H-NMR of both crude mixtures and crude mixtures. If the two ratios are same, we used the one from the crude mixture; otherwise, we used the one from the crude mixture.

General Procedure B: Non-directed Electrochemical C–H Olefinations

The electrocatalysis was carried out in a pre-dried divided cell, with a GF anode (10 mm × 15 mm × 6 mm) and a platinum cathode (10 mm × 15 mm × 0.25 mm). Arene **1-2**, or **53** (5.0 – 20 equiv), acrylates **3** (0.20 mmol, 1.0 equiv), $Pd(OAc)_2$ (4.5 mg, 10 mol %), **L3** (6.5 mg, 20 mol %) or **L12** (7.9 mg, 20 mol %), 1,4-benzoquinone (4.3 mg, 20 mol %) and NaOAc (66.0 mg, 4.0 equiv) were placed in the anodic chamber and dissolved in AcOH (2.6 mL) and HFIP (1.3 mL). 1,4-Benzoquinone (4.3 mg, 20 mol %) and NaOAc (66.0 mg, 4.0 equiv) were placed in the cathodic chamber and dissolved in AcOH (2.6 mL) and HFIP (1.3 mL). Electrocatalysis was performed at 60 °C with a constant current of 1.0 mA and a stirring rate of 500 rpm maintained for 20 h. At ambient temperature, the GF anode was washed with EtOAc (3 × 10 mL) in an ultrasonic bath and the washings were added to the reaction mixture. The solvents were removed *in vacuo*. The residues were added sat. $NaHCO_3$ (30 mL), extracted with CH_2Cl_2 (3 x 20 mL). The combined organic layers were dried over Na_2SO_4, filtered and concentrated under reduced pressure. The NMR yield was determined by adding CH_2Br_2 (14.0 µL, 0.20 mmol, 1.0 equiv) as internal standard. The crude mixture was purified by flash column chromatography on silica gel to yield the products **4-52, 54-69, MS1-6**. The isomer ratios were checked by ^{1}H-NMR of both crude mixtures and crude mixtures. If the two ratios are same, we used the one from the crude mixture; otherwise, we used the one from the crude mixture.

General Procedure C: Non-directed Electrochemical C–H Olefinations

The electrocatalysis was carried out in a pre-dried divided cell, with a GF anode (10 mm × 15 mm × 6 mm) and a platinum cathode (10 mm × 15 mm × 0.25 mm). Arene **1-2**, or **53** (1.0–3.0 equiv), acrylates **3** (0.50 mmol, 1.0 equiv), $Pd(OAc)_2$ (11.2 mg, 10 mol %), **L3** (16.3 mg, 20 mol %) or **L12** (19.6 mg, 20 mol %), 1,4-benzoquinone (5.4 mg, 10 mol %) and NaOAc (49.5 mg) were placed in the anodic chamber and dissolved in AcOH (2.0 mL) and HFIP (1.0 mL). 1,4-Benzoquinone (5.4 mg, 10 mol %) and NaOAc (49.5 mg) were placed in the cathodic chamber and dissolved in AcOH (2.0 mL) and HFIP (1.0 mL). Electrocatalysis was performed at 60 °C with a constant current of 1.0 mA and a stirring rate of 500 rpm maintained for 48 h. At ambient temperature, the reaction mixture was diluted with EtOAc (5.0 mL). The GF anode was washed with EtOAc (3 × 10 mL) in an ultrasonic bath and the washings were added to the reaction mixture. The resulting mixture was loaded in a column chromatography with a pad of silica and washed with 50 mL EtOAc. The combined solvents were removed *in vacuo*. The NMR yield was determined by adding CH_2Br_2 (35.0 µL, 0.50 mmol, 1.0 equiv) as internal standard. The crude mixture was purified by flash column chromatography on silica gel to yield the products **4-52, 54-69, MS1-6**. The isomer ratios were measured by ^{1}H-NMR of both crude mixtures and crude mixtures. If the two ratios are same, we used the one from the crude mixture; otherwise, we used the one from the crude mixture.

General Procedure D: Non-directed Electrochemical C–H Olefinations

The electrocatalysis was carried out in a pre-dried divided cell, with a GF anode (10 mm × 15 mm × 6 mm) and a platinum cathode (10 mm × 15 mm × 0.25 mm). Arene **1-2**, or **53** (1.0 equiv), acrylates **3** (1.0 mmol, 2.0 equiv), $Pd(OAc)_2$ (11.2 mg, 10 mol %), **L3** (16.3 mg, 20 mol %) or **L12** (19.6 mg, 20 mol %), 1,4-benzoquinone (5.4 mg, 10 mol %) and NaOAc (49.5 mg) were placed in the anodic chamber and dissolved in AcOH (2.0 mL) and HFIP (1.0 mL). 1,4-Benzoquinone (5.4 mg, 10 mol %) and NaOAc (49.5 mg) were placed in the cathodic chamber and dissolved in AcOH (2.0 mL) and HFIP (1.0 mL). Electrocatalysis was performed at 80 °C with a constant current of 1.0 mA and a stirring rate of 500 rpm maintained for 48 h. At ambient temperature, the reaction mixture was diluted with EtOAc (5.0 mL). The GF anode was washed with EtOAc (3 × 10 mL) in an ultrasonic bath and the washings were added to the reaction mixture. The resulting mixture was loaded in a column chromatography with a pad of silica and washed with 50 mL EtOAc. The combined solvents were removed *in vacuo*. The NMR yield was determined by adding CH_2Br_2 (35.0 µL, 0.50 mmol, 1.0 equiv) as internal standard. The crude mixture was purified by flash column chromatography on silica gel to yield the products **4-52, 54-69, MS1-6**. The isomer ratios were measured by ^{1}H-NMR of both crude mixtures and crude mixtures. If the two ratios are same, we used the one from the crude mixture; otherwise, we used the one from the crude mixture.

Syntheses of Ligands

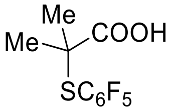

2-Methyl-2-[(perfluorophenyl)thio]propanoic acid (L11)

2-Methyl-2-[(perfluorophenyl)thio]propanoic acid (**L11**) was prepared following the procedure described in the literature.[1] 2,3,4,5,6-Pentafluorothiophenol (1.33 mL, 10 mmol, 1.0 equiv) was added to a mixture of 2-bromo-2-methylpropionic acid (1.68 g, 10 mmol, 1.0 equiv) and NaOH (1.0 g, 20 mmol, 2.0 equiv) in *t*-BuOH (30 mL) and H_2O (5.0 mL) at room temperature. Then the reaction mixture was refluxed overnight. At ambient temperature, the reaction mixture was concentrated in *vacuo*. The resulting pale, yellow crude mixture was acidified with 2M HCl solution. The aqueous layer was extracted with Et_2O (3 x 100 mL) and the combined organic layers were dried over anhydrous Na_2SO_4. Then, the mixture was filtered and concentrated under reduced pressure. Purification by column chromatography on silica gel using *n*-hexane/EtOAc (100:1 to 10:1) as an eluent provided **L11** as white crystals solid. ^{1}H-NMR (400 MHz, $CDCl_3$): δ = 10.76 (brs, 1H), 1.55 (s, 6H). ^{13}C-NMR (100 MHz, $CDCl_3$): δ = 179.3 (C_q), 150.2 (dq, J = 10.7, 3.7 Hz), 147.7 (dq, J = 10.9, 3.8 Hz), 144.2 (tt, J = 13.6, 5.2 Hz, C_q), 141.6 (tt, J = 13.6, 5.2 Hz, C_q), 139.4 – 138.4 (m, C_q), 137.0 – 135.9 (m, C_q), 51.7 (C_q), 24.7 (CH_3). ^{19}F-NMR (377 MHz, $CDCl_3$): δ = -128.9 – -129.4 (m, 2F), -148.6 (tt, J = 20.8, 4.0 Hz, 1F), -160.5 – -161.0 (m, 2F). IR (ATR): 2919, 2633, 2534, 1710, 1447, 1281, 1092, 979, 861, 547 cm^{-1}. MS (ESI) *m/z* (relative intensity): 285 [M - H]$^-$. HR-MS (ESI): *m/z* calcd. for [$C_{13}H_{16}O_2$ - H]$^-$ 285.0014, found 285.0005.

Other *S,O*-ligands (**L9, L10, L12, L36**) were prepared following a modified procedure adapted from description above.[1] 15% water was used as co-solvent along with *t*-BuOH or EtOH.

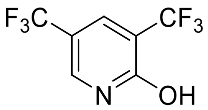

3,5-bis(trifluoromethyl)pyridin-2-ol (L4)

L4 was prepared following the procedure described in the literature.[2] To a suspension of 2-hydroxy-3-trifluoromethylpyridine (2.0 g, 12.25 mmol) in acetonitrile (7.0 mL) was added

glacial acetic acid (0.8 mL, 12.8 mmol) and *N*-Iodosuccinimide (3.3 g, 14.7 mmol). The mixture was heated to 65 °C for 5 h to form a red solution. Then the slurry was cooled, and 10 wt % sodium thiosulfate solution (8.0 mL) and water (11 mL) was added. The yellow solid come out in solution and the mixture was allowed to cool to room temperature, filtered, washed with water and hexane to afford 5-iodo-3-(trifluoromethyl)pyridin-2(*1H*)-one as a light-yellow solid.

To a solution of 5-iodo-3-(trifluoromethyl)pyridin-2(*1H*)-one (2.02 g, 7 mmol) and BnBr (1.37 g, 8 mmol) in DMF (10 mL) was added K_2CO_3 (1.1 g, 8 mmol) at room temperature. The mixture was stirred at rt for overnight. Then the mixture was diluted with EA (50 mL) and washed with water and brine. The organic solution then dried over Mg_2SO_4, concentrated and purified by column to afford 1-benzyl-5-iodo-3-(trifluoromethyl)pyridin-2(*1H*)-one as a yellow solid.

A mixture of 1-benzyl-5-iodo-3-(trifluoromethyl)pyridin-2(*1H*)-one (1.9 g, 5 mmol), CuI (1.90 g, 10 mmol), CF_3-reagent (1.92 g, 10 mmol) in DMF (80 mL) was heated at 100 °C in a sealed tube for 16 hours. After cooled to room temperature, the reaction was quenched with sat. NH_4Cl solution. The water phase was extracted by EA (3 x 50 mL) and the combined organic layers were dried over Mg_2SO_4. The solution was concentrated under vacuum and purified by column to afford 1-benzyl-3,5-bis(trifluoromethyl)pyridin-2(*1H*)-one as white solid.

To a solution 1-benzyl-3,5-bis(trifluoromethyl)pyridin-2(*1H*)-one (2.81g, 8.75 mmol) in AcOH (40 mL) as added 20% $Pd(OH)_2$/C (10 mol%) slowly. Then the air was removed and the flask was back filled with hydrogen three times. A hydrogen balloon was left on the flask and the reaction mixture was stirred for 16 hours at 60 °C. After the 16 hours, the reaction mixture was filtered through a pad of Celite and the solvent was evaporated. The crude product was purified by silica gel chromatography using Hexane/EA (1/2, v/v) as eluent to afford **L4** as white solid.

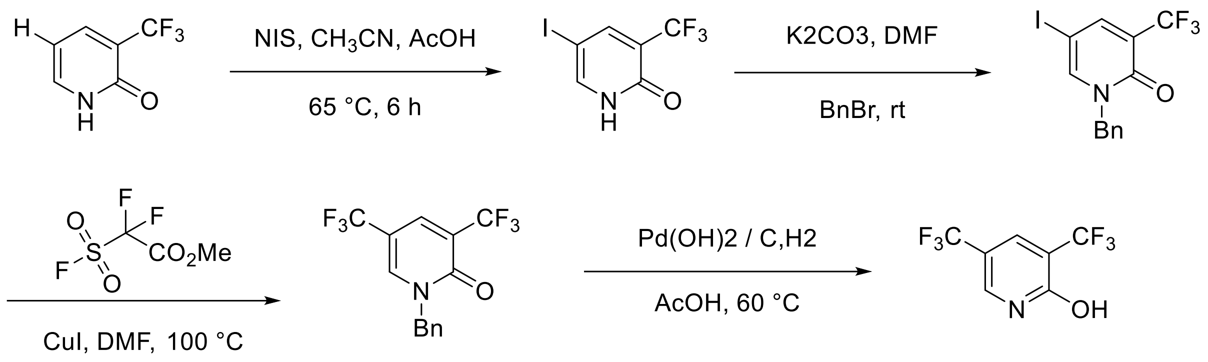

Supplementary Figure 1 Synthesis of L4

Syntheses of Products

To confirmed the position-selectivity, some unknown products (*α*-**14**, *β*-**14**, *δ*-**14**, *α*-**15**, *δ*-**15** *α*-**31**, *β*-**31**, *γ*-**31** and *δ*-**31**) were synthesized separately.

Heck Reaction

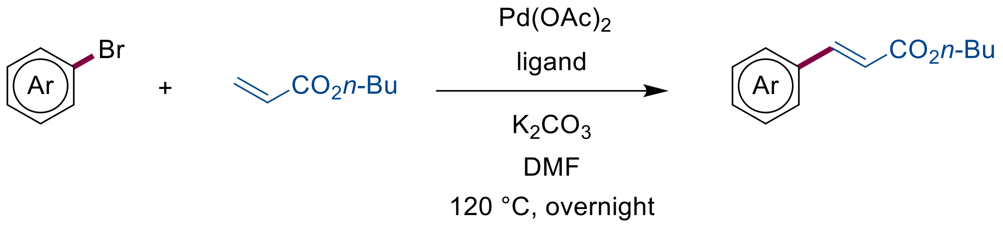

Supplementary Figure 2 Heck reaction

α-**14**, *δ*-**14**, *α*-**31**, *β*-**31**, *γ*-**31** and *δ*-**31** were prepared through Heck reaction followed the procedure described in the literature.[3] Aryl bromine (1.0 mmol), n-butyl arylate (1.5 mmol, 1.5 equiv.), $Pd(OAc)_2$ (10 mol%), K_2CO_3 (2.0 mmol, 2.0 equiv.) and DMF (2 mL) were adding to a flask. For *α*-**14** and *δ*-**14**, tri-*o*-tolylphosphine (15 mol%) was used as the ligand as well. Then the reaction mixture was refluxed overnight. At ambient temperature, the reaction mixture was diluted with 25 mL EtOAc and washed with sat. NH_4Cl solution (3 x 10 mL). The organic layers were dried over anhydrous Na_2SO_4. Then, the mixture was filtered and concentrated under reduced pressure. Purification by column chromatography on silica gel provided the product.

Knoevenagel Reaction

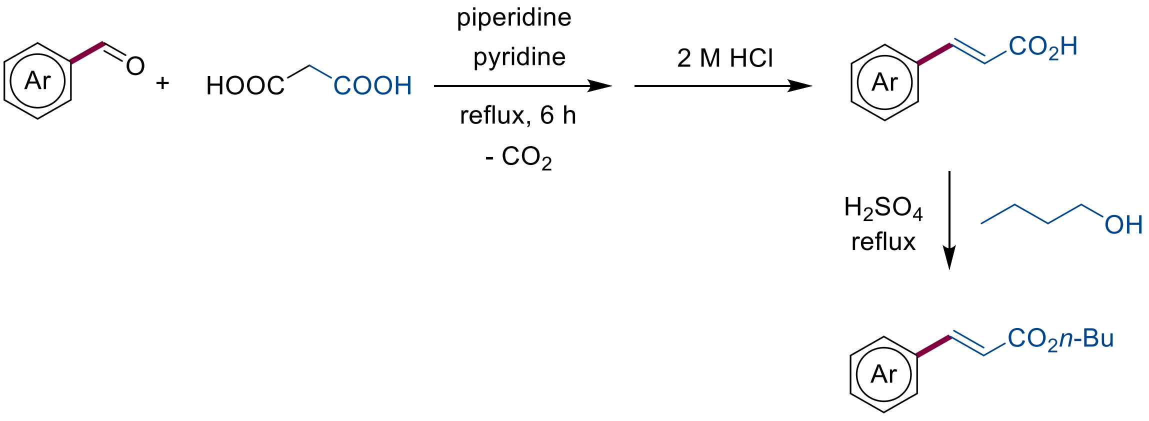

Supplementary Figure 3 Knoevenagel reaction

β-**14**, *α*-**15**, and *δ*-**15** were prepared through Knoevenagel reaction followed the procedure described in the literature.[4] Aryl aldehyde (1.0 mmol), Malonic acid (1.5 mmol, 1.5 equiv.), piperidine (5 mol%), pyridine (2 mL) were adding to a flask. Then the reaction mixture was refluxed for 6 h. At ambient temperature, the reaction mixture was concentrated in *vacuo*. The resulting crude mixture was acidified with 2M HCl solution (10 mL). The aqueous layer was extracted with EtOAc (3 x 10 mL) and the combined organic layers were dried over anhydrous Na_2SO_4. Then, the mixture was added with *n*-butanol (1ml) and 2 drops of H_2SO_4, refluxed for 3 h. At ambient temperature, the reaction mixture was directly purified by column chromatography.

Product Characterization

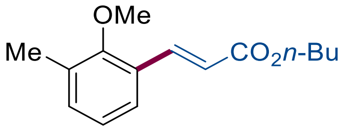

(*E*)-butyl-3-(2-methoxy-3-methylphenyl)acrylate (*α*-14)

^{1}H-NMR (400 MHz, $CDCl_3$): δ = 7.97 (d, *J* = 16.2 Hz, 1H), 7.41 (dd, *J* = 7.8, 1.7 Hz, 1H), 7.21 (ddd, *J* = 7.6, 1.8, 0.9 Hz, 1H), 7.04 (t, *J* = 7.6 Hz, 1H), 6.48 (d, *J* = 16.2 Hz, 1H), 4.21 (t, *J* = 6.7 Hz, 2H), 3.75 (s, 3H), 2.31 (s, 3H), 1.75 – 1.65 (m, 2H), 1.51 – 1.38 (m, 2H), 0.97 (t, *J* = 7.4 Hz, 3H). ^{13}C-NMR (101 MHz, $CDCl_3$): δ = 167.4 (C_q), 157.9 (C_q), 139.7 (CH), 133.2 (CH), 131.9 (C_q), 128.0 (C_q), 125.6 (CH), 124.3 (CH), 119.3 (CH), 64.4 (CH_2), 61.4 (CH_3), 30.8 (CH_2), 19.3 (CH_2), 16.0 (CH_3), 13.8 (CH_3). MS (ESI) *m*/*z* (relative intensity): 271 (100) $[M + Na]^+$, 249 (0) $[M + H]^+$. HR-MS (ESI): *m*/*z* calcd. for $[C_{15}H_{20}O_3 + Na]^+$ 271.1305 found 271.1298.

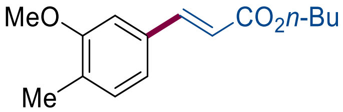

(*E*)-butyl-3-(3-methoxy-4-methylphenyl)acrylate (*β*-14)

^{1}H-NMR (400 MHz, $CDCl_3$): δ = 7.64 (d, *J* = 15.9 Hz, 1H), 7.11 (d, *J* = 7.6 Hz, 1H), 7.02 (dd, *J* = 7.6, 1.6 Hz, 1H), 6.95 (d, *J* = 1.6 Hz, 1H), 6.39 (d, *J* = 15.9 Hz, 1H), 4.20 (t, *J* = 6.7 Hz, 2H), 3.83 (s, 3H), 2.22 (s, 3H), 1.74 – 1.63 (m, 2H), 1.49 – 1.39 (m, 2H), 0.96 (t, *J* = 7.4 Hz, 3H). ^{13}C-NMR (101 MHz, $CDCl_3$): δ = 167.2 (C_q), 158.0 (C_q), 144.9 (CH), 133.4 (C_q), 130.9

(CH), 129.7 (C_q), 120.9 (CH), 117.2 (CH), 108.6 (CH), 64.3 (CH_2), 55.2 (CH_3), 30.9 (CH_2), 19.3 (CH_2), 16.3 (CH_3), 13.8 (CH_3). MS (ESI) *m*/*z* (relative intensity): 271 (100) $[M + Na]^+$, 249 (40) $[M + H]^+$. HR-MS (ESI): *m*/*z* calcd. for $[C_{15}H_{20}O_3 + Na]^+$ 271.1305 found 271.1307.

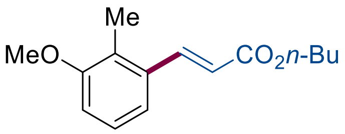

(*E*)-butyl-3-(3-methoxy-2-methylphenyl)acrylate (*δ*-14)

^{1}H-NMR (400 MHz, $CDCl_3$): δ = 8.02 (d, *J* = 15.8 Hz, 1H), 7.19 – 7.14 (m, 2H), 6.90 – 6.83 (m, 1H), 6.34 (d, *J* = 15.8 Hz, 1H), 4.20 (d, *J* = 6.7 Hz, 2H), 3.84 (s, 3H), 2.30 (s, 3H), 1.76 – 1.66 (m, 2H), 1.50 – 1.38 (m, 2H), 0.97 (t, *J* = 7.4 Hz, 3H). ^{13}C NMR (101 MHz, CDC13) δ 167.2 (C_q), 157.9 (C_q), 142.6 (CH), 134.8 (C_q), 126.6 (CH), 126.5 (CH), 120.0 (CH), 118.7 (CH), 111.4 (CH), 64.4 (CH_2), 55.7 (CH_3), 30.8 (CH_2), 19.3 (CH), 13.8 (CH_3), 11.5 (CH_3). MS (ESI) *m*/*z* (relative intensity): 271 (100) $[M + Na]^+$, 249 (0) $[M + H]^+$. HR-MS (ESI): *m*/*z* calcd. for $[C_{15}H_{20}O_3 + Na]^+$ 271.1305 found 271.1298.

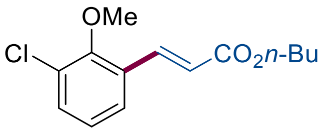

(*E*)-butyl-3-(3-chloro-2-methoxyphenyl)acrylate (*α*-15)

^{1}H-NMR (400 MHz, $CDCl_3$): δ = 7.89 (d, *J* = 16.2 Hz, 1H), 7.42 (dd, *J* = 7.8, 1.6 Hz, 1H), 7.36 (dd, *J* = 8.0, 1.5 Hz, 1H), 7.03 (t, *J* = 7.9 Hz, 1H), 6.47 (d, *J* = 16.2 Hz, 1H), 4.19 (t, *J* = 6.7 Hz, 2H), 3.83 (s, 3H), 1.72 – 1.61 (m, 2H), 1.48 – 1.35 (m, 2H), 0.94 (t, *J* = 7.4 Hz, 3H). ^{13}C-NMR (101 MHz, $CDCl_3$): δ = 166.8 (C_q), 155.1 (C_q), 138.4 (CH), 132.0 (CH), 130.1 (C_q), 128.8 (C_q), 126.3 (CH), 125.0 (CH), 120.7 (CH), 64.5 (CH_2), 61.6 (CH_3), 30.8 (CH_2), 19.2 (CH_2), 13.8 (CH_3). MS (ESI) *m*/*z* (relative intensity): 291 (100) $[M + Na]^+$, 269 (10) $[M + H]^+$. HR-MS (ESI): *m*/*z* calcd. for $[C_{14}H_{17}ClO_3 + Na]^+$ 291.0758 found 291.0758.

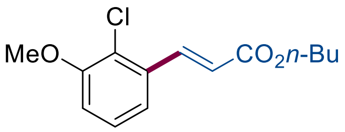

(*E*)-butyl-3-(2-chloro-3-methoxyphenyl)acrylate (*δ*-15)

^{1}H-NMR (400 MHz, $CDCl_3$): δ = 8.11 (d, J = 16.0 Hz, 1H), 7.20 (dd, J = 4.8, 0.8 Hz, 2H), 6.95 – 6.88 (m, 1H), 6.40 (d, J = 16.0 Hz, 1H), 4.20 (t, J = 6.7 Hz, 2H), 3.88 (s, 3H), 1.72 – 1.62 (m, 2H), 1.49 – 1.35 (m, 2H), 0.95 (t, J = 7.4 Hz, 3H). ^{13}C-NMR (101 MHz, $CDCl_3$): δ = 166.6 (C_q), 155.5 (C_q), 140.6 (CH), 134.2 (C_q), 127.3 (CH), 123.4 (C_q), 121.3 (CH), 119.3 (CH), 112.9 (CH), 64.6 (CH_2), 56.3 (CH_3), 30.8 (CH_2), 19.2 (CH_2), 13.8 (CH_3). MS (ESI) *m*/*z* (relative intensity): 291 (100) $[M + Na]^+$, 269 (10) $[M + H]^+$. HR-MS (ESI): *m*/*z* calcd. for $[C_{14}H_{17}ClO_3 + Na]^+$ 291.0758 found 291.0763.

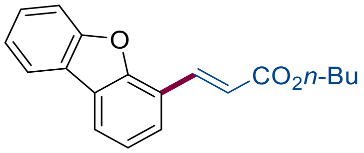

(*E*)-butyl-3-(dibenzo[*b*,*d*]furan-4-yl)acrylate (*α*-31)

^{1}H-NMR (600 MHz, $CDCl_3$): δ = 7.98 – 7.93 (m, 3H), 7.65 (dt, J = 8.3, 0.8 Hz, 1H), 7.55 (ddd, J = 7.5, 1.3, 0.6 Hz, 1H), 7.49 (ddd, J = 8.4, 7.3, 1.3 Hz, 1H), 7.39 – 7.33 (m, 2H), 7.08 (d, J = 16.1 Hz, 1H), 4.28 (t, J = 6.7 Hz, 2H), 1.78 – 1.73 (m, 2H), 1.53 – 1.46 (m, 2H), 1.00 (t, J = 7.4 Hz, 3H). ^{13}C-NMR (126 MHz, $CDCl_3$): δ = 167.4 (C_q), 156.1 (C_q), 154.4 (C_q), 139.3 (CH), 128.3 (CH), 127.6 (CH), 125.0 (C_q), 123.5 (C_q), 123.1 (CH), 123.0 (CH), 122.2 (CH), 121.6 (CH), 120.7 (CH), 119.7 (C_q), 111.9 (CH), 64.5 (CH_2), 30.8 (CH_2), 19.2 (CH_2), 13.8 (CH_3). MS (ESI) *m*/*z* (relative intensity): 317 (100) $[M + Na]^+$, 249 (0) $[M + H]^+$. HR-MS (ESI): *m*/*z* calcd. for $[C_{19}H_{18}O_3 + Na]^+$ 317.1148 found 317.1148.

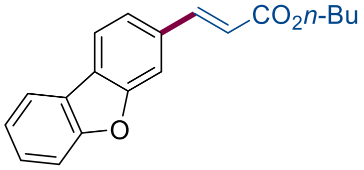

(*E*)-butyl-3-(dibenzo[*b*,*d*]furan-3-yl)acrylate (*β*-31)

^{1}H-NMR (600 MHz, $CDCl_3$): δ = 7.95 (ddd, J = 8.4, 1.7, 1.0 Hz, 1H), 7.94 (d, J = 8.3 Hz, 1H), 7.81 (d, J = 15.9 Hz, 1H), 7.72 (dt, J = 1.3, 0.6 Hz, 1H), 7.58 (dt, J = 8.3, 0.8 Hz, 1H), 7.53 (ddd, J = 8.0, 1.5, 0.5 Hz, 1H), 7.49 (ddd, J = 8.4, 7.3, 1.3 Hz, 1H), 7.38 – 7.34 (m, 1H), 6.54 (d, J = 15.9 Hz, 1H), 4.24 (t, J = 6.7 Hz, 2H), 1.72 (ddt, J = 9.0, 7.7, 6.7 Hz, 2H), 1.50 – 1.43 (m, 2H), 0.98 (t, J = 7.4 Hz, 3H). ^{13}C-NMR (126 MHz, $CDCl_3$): δ = 167.0 (C_q), 157.0 (C_q), 156.4 (C_q), 144.5 (CH), 133.7 (C_q), 127.9 (CH), 126.1 (C_q), 123.7 (C_q), 123.1 (CH), 123.0 (CH),

120.9 (CH), 120.8 (CH), 118.5 (CH), 111.8 (CH), 110.9 (CH), 64.5 (CH_2), 30.8 (CH_2), 19.2 (CH_2), 13.7 (CH_3). MS (ESI) *m*/*z* (relative intensity): 317 (100) [M + Na]$^+$, 249 (0) [M + H]$^+$. HR-MS (ESI): *m*/*z* calcd. for [$C_{19}H_{18}O_3$ + Na]$^+$ 317.1148 found 317.1140.

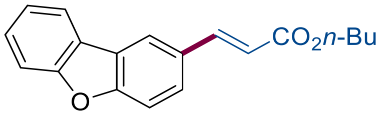

(*E*)-butyl-3-(dibenzo[*b*,*d*]furan-2-yl)acrylate (*γ*-31)

^{1}H-NMR (400 MHz, $CDCl_3$): δ = 8.12 (d, *J* = 1.8 Hz, 1H), 7.96 (ddd, *J* = 7.7, 1.4, 0.6 Hz, 1H), 7.85 (d, *J* = 15.9 Hz, 1H), 7.65 (dd, *J* = 8.7, 1.8 Hz, 1H), 7.60 – 7.54 (m, 2H), 7.49 (ddd, *J* = 8.3, 7.2, 1.4 Hz, 1H), 7.38 (td, *J* = 7.5, 1.0 Hz, 1H), 6.51 (d, *J* = 16.0 Hz, 1H), 4.24 (t, *J* = 6.7 Hz, 2H), 1.77 – 1.67 (m, 2H), 1.52 – 1.41 (m, 2H), 0.99 (t, *J* = 7.4 Hz, 3H). ^{13}C-NMR (101 MHz, $CDCl_3$): δ = 167.2 (C_q), 157.3 (C_q), 156.7 (C_q), 144.6 (CH), 129.5 (C_q), 127.7 (CH), 127.3 (CH), 124.9 (C_q), 123.7 (C_q), 123.1 (CH), 120.8 (CH), 120.6 (CH), 117.4 (CH), 112.1 (CH), 111.9 (CH), 64.4 (CH_2), 30.8 (CH_2), 19.2 (CH_2), 13.8 (CH_3). MS (ESI) *m*/*z* (relative intensity): 317 (100) [M + Na]$^+$, 249 (0) [M + H]$^+$. HR-MS (ESI): *m*/*z* calcd. for [$C_{19}H_{18}O_3$ + Na]$^+$ 317.1148 found 317.1144.

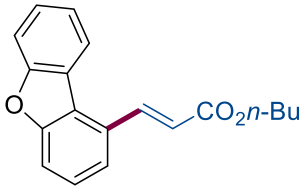

(*E*)-butyl-3-(dibenzo[*b*,*d*]furan-1-yl)acrylate (*δ*-31)

^{1}H-NMR (600 MHz, $CDCl_3$): δ = 8.54 (d, *J* = 15.9 Hz, 1H), 8.15 (ddd, *J* = 8.0, 1.2, 0.5 Hz, 1H), 7.60 (dt, *J* = 8.2, 0.9 Hz, 1H), 7.58 (dd, *J* = 8.1, 0.9 Hz, 1H), 7.57 (dt, *J* = 7.8, 0.8 Hz, 1H), 7.50 (ddd, *J* = 8.4, 7.3, 1.3 Hz, 1H), 7.45 (td, *J* = 7.9, 0.6 Hz, 1H), 7.39 (ddd, *J* = 7.8, 7.3, 1.0 Hz, 1H), 6.61 (d, *J* = 15.8 Hz, 1H), 4.29 (t, *J* = 6.7 Hz, 2H), 1.75 (ddt, *J* = 9.0, 7.8, 6.6 Hz, 2H), 1.54 – 1.46 (m, 2H), 1.01 (t, *J* = 7.4 Hz, 3H). ^{13}C-NMR (126 MHz, $CDCl_3$): δ = 166.9 (C_q), 156.4 (C_q), 156.3 (C_q), 140.8 (CH), 130.5 (C_q), 127.6 (CH), 127.1 (CH), 123.6 (C_q), 123.2 (C_q), 123.2 (C_q), 123.0 (CH), 120.8 (CH), 120.5 (CH), 112.8 (CH), 111.8 (CH), 64.6 (CH_2), 30.8 (CH_2), 19.3 (CH_2), 13.8 (CH_3). MS (ESI) *m*/*z* (relative intensity): 317 (100) [M + Na]$^+$, 249 (0) [M + H]$^+$. HR-MS (ESI): *m*/*z* calcd. for [$C_{19}H_{18}O_3$ + Na]$^+$ 317.1148 found 317.1136.

Syntheses of Acrylate

Acrylates **3n** and **3o** were synthesized according to known methods.[5,6] The mixture of alcohols or phenols and Et_3N (1.5 eq.) in dry CH_2Cl_2 was cooled to 0 °C in an ice-water bath and acryloyl chloride (1.2 eq.) was added dropwise. The mixture was warmed to room temperature and stirred for overnight. The solvent was removed under reduced pressure and the residue was chromatographed on silica gel to get the desired product.

Functional-group Protection for Drug Compounds

Drug derivatives **53b**, **53d**, **53e**, **53g**, **53h**, **53j-53l** were synthesized according to previous literature.[7-10]

Carboxylic Acid Protecting Method 1

For **53b**, **53d**, **53e**, **53g**, **53h**, to a solution of carboxylic acid deriverities in dimethylformamide at 0 °C was added potassium carbonate (5.0 eq.). Iodomethane (3.0 eq.) was then added slowly and the reaction mixture allowed to warm to room temperature. After 16 hours, the reaction mixture was diluted with ethyl acetate (10 mL), washed with sodium hydrogen carbonate (1 x 15 mL of a saturated aqueous solution), water (2 x 15 mL) and brine (2 x 15 mL) and the organic layer dried ($MgSO_4$) and concentrated in vacuo. The residue was chromatographed on silica gel to get the desired product.

Phenol Protecting

For **53j**, A solution of phenol deriverties in dimethylformamide was treated with ground potassium hydroxide (1.2 eq.) and iodomethane (1.2 eq.) in 20 mL DMF (dropwise over 5 min at 0 °C). the reaction was stirred at room temperature for 30 min and, after this time period, additional ground potassium hydroxide (1.2 eq.) and iodomethane (1.2 eq.) in DMF were added at 0 °C. The reaction was then left to stir for 3 h. The solution was poured onto ice and extracted with ethyl acetate (3 x 50 mL). The organic layers were washed with water, brine and dried by $MgSO_4$. The solvent was removed under reduced pressure to yield the desired product.

Carboxylic Acid Protecting Method 2

For **53k-l**, to a solution of carboxylic acid deriverities in corresponding alcohol solution, a drop of concentrated H_2SO_4 was added and the reaction mixture was refluxed overnight. Then the reaction mixture was concentrated in vacuo. The residue was chromatographed on silica gel to get the desired product.

Reaction Set-up

Reactor

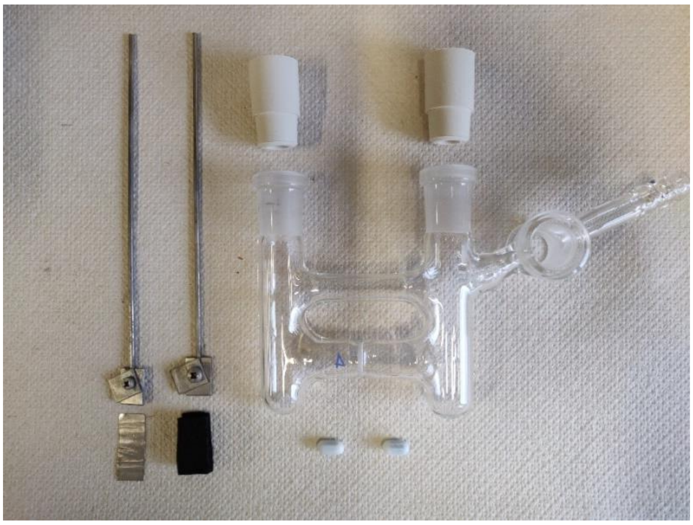

Supplementary Figure 4 Divided cell

Potentiostat

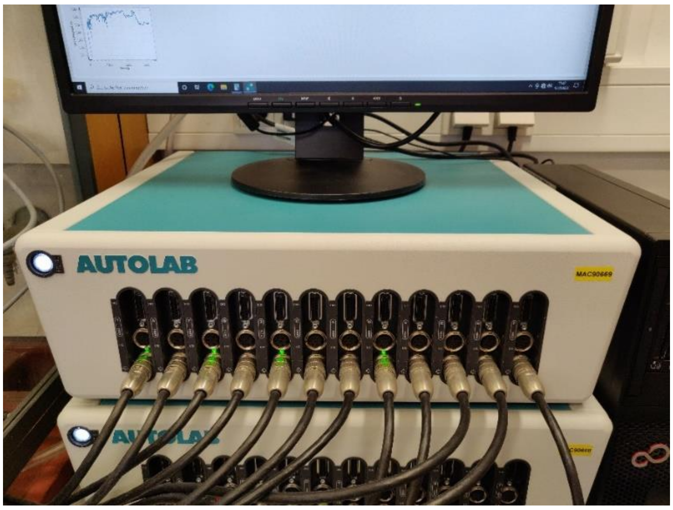

Supplementary Figure 5 Metrohm MULTI AUTOLAB M204 potentiostat S1

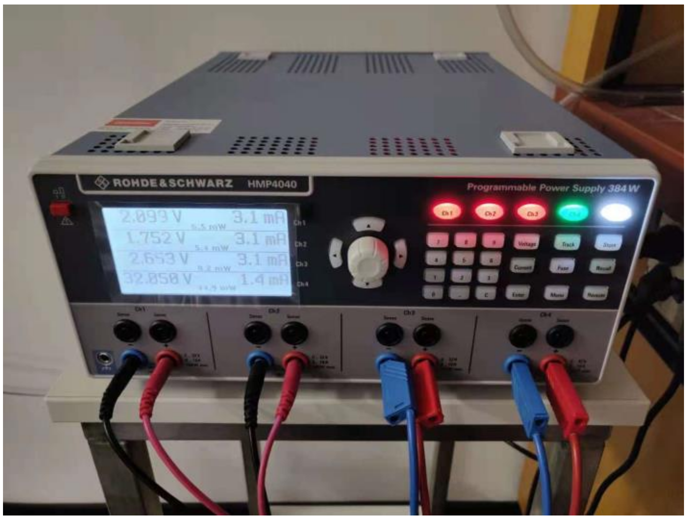

Supplementary Figure 6 **ROHDE & SCHWARZ HMP4040 Potentiostat S2**

Supplementary Discussion

Optimization Studies

Optimization Based on Pyridone Ligand

Supplementary Table 1 **Optimization of the non-directed electrochemical C–H olefination**

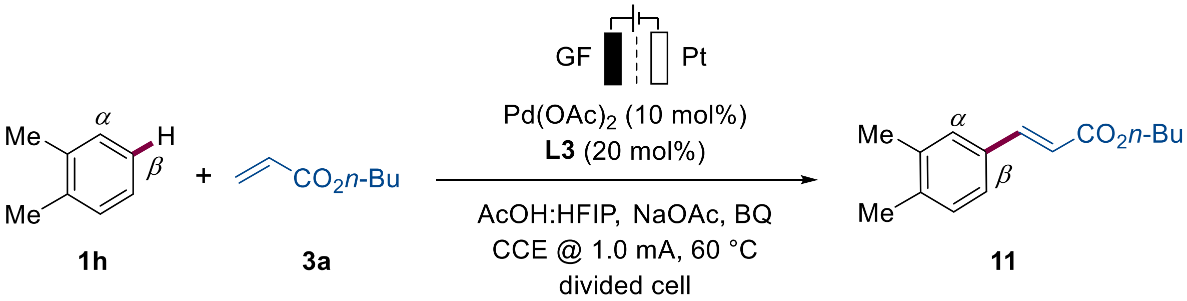

Entry	Deviation from standard condition	Yield [%]	*α:β*
1	None	69[a]	1:6
2	No electricity	27	1:7
3	No BQ	37[b]	1:8
4	No BQ in cathodic cell	45	1:6
5	No $Pd(OAc)_2$	---[b,d]	---
6	No NaOPiv	11[b,d]	1:6

7	Undivided cell	<5[b,c,d]	---
8	No ligand	36[c]	1:4
9	Under N_2	38[c]	1:12
10	BQ (1.0 equiv) without electricity, under N_2	8[c]	---
11	10 mol % BQ instead of 20 mol % BQ	59[c]	1:5

Reaction conditions: divided cell, anodic chamber: **1h** (4.0 mmol), **3a** (0.20 mmol), [Pd] (10 mol %), **L3** (20 mol %), NaOPiv (4.0 equiv), BQ (20 mol %), HFIP:AcOH (1.3 mL:2.6 mL), cathodic chamber: NaOPiv (4.0 equiv), BQ (20 mol %), HFIP:AcOH (1.3 mL:2.6 mL), 60 °C, constant current at 1.0 mA, 20 h, graphite felt (GF) anode, Pt-plate cathode. Without special note, yields and isomer ratios were determined by crude ^{1}H-NMR by using CH_2Br_2 as internal standard. [a] Isolated yield. [b] No BQ. [c] NaOAc (4.0 equiv). [d] HFIP:AcOH (2.0 mL:2.0 mL).

Supplementary Table 2 Additive screening

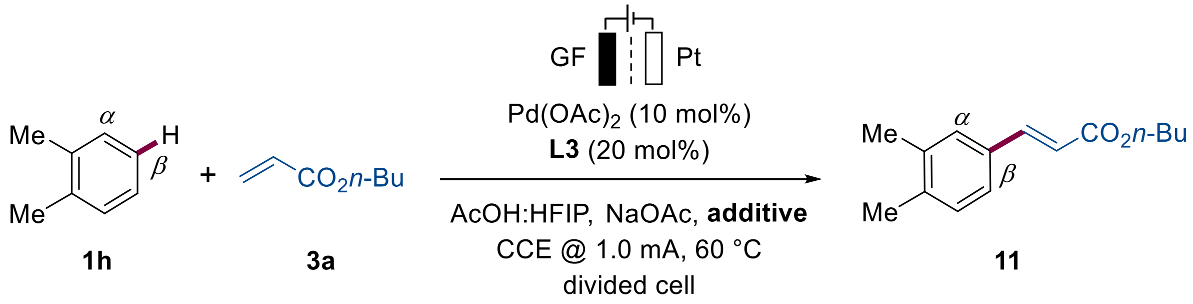

1h **3a** **11**

Entry	Additive	Yield	*α:β*
1	None	37%	1:8
**2**	**1,4-benzoquinone**	**45%**	**1:6**
3	TBAI	<5%	---
4	Ferrocene	15%	1:8
5	2,5-di-*tert*-butylcyclohexa-2,5-diene-1,4-dione	33%	1:6

Reaction conditions: divided cell, anodic chamber: **1h** (4.0 mmol), **3a** (0.20 mmol), [Pd] (10 mol %), **L3** (20 mol %), NaOPiv (4.0 equiv), additive (20 mol %), HFIP:AcOH (1.3 mL:2.6 mL), cathodic chamber: NaOPiv (4.0 equiv), HFIP:AcOH (1.3 mL:2.6 mL), 60 °C, constant

current at 1.0 mA, 20 h, graphite felt (GF) anode, Pt-plate cathode. Without special note, yields and isomer ratios were determined by crude ^{1}H-NMR by using CH_2Br_2 as internal standard.

Supplementary Table 3 Base screening

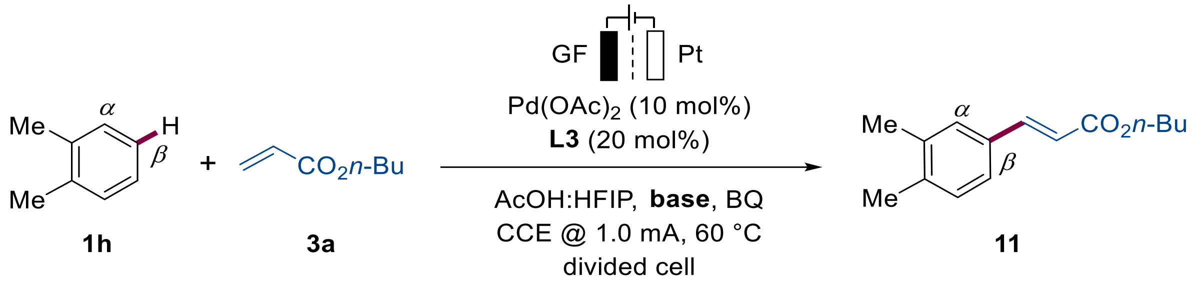

Entry	Base	Yield [%]	*α*:*β*
1	NaOPiv	76	1:6
**2**	**NaOAc**	**73**	**1:7**
3	KOAc	51	1:7
4	LiOAc	40	1:7
5	*n*-Bu_4NOAc	25	1:5

Reaction conditions: divided cell, anodic chamber: **1h** (4.0 mmol), **3a** (0.20 mmol), [Pd] (10 mol %), **L3** (20 mol %), BQ (20 mol %), base (4.0 equiv), HFIP:AcOH (1.3 mL:2.6 mL); cathodic chamber: BQ (20 mol %), base (4.0 equiv), HFIP:AcOH (1.3 mL:2.6 mL), 60 °C, constant current at 1.0 mA, 20 h, graphite felt (GF) anode, Pt-plate cathode. Without special note, yields and isomer ratios were determined by crude ^{1}H-NMR by using CH_2Br_2 as internal standard.

Supplementary Table 4 Solvent screening

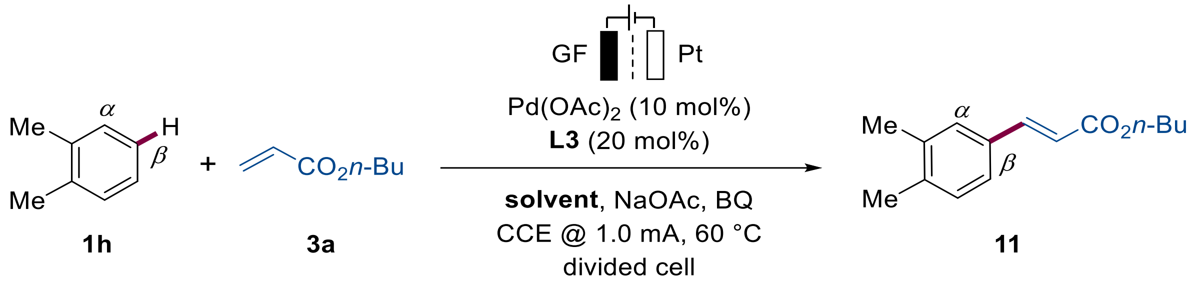

Entry	Solvent	Yield [%]	*α*:*β*
1	HFIP:AcOH (1:1)	63	1:8
**2**	**HFIP:AcOH (1:2)**	**76**	**1:6**
3	HFIP:AcOH (1:3)	60	1:6
4	HFIP:AcOH (1:4)	60	1:6
5	NMP:AcOH (1:2)	<5	---

Reaction conditions: divided cell, anodic chamber: **1h** (4.0 mmol), **3a** (0.20 mmol), [Pd] (10 mol %), **L3** (20 mol %), NaOPiv (4.0 equiv), BQ (20 mol %), solvent (4 mL); cathodic chamber: NaOPiv (4.0 equiv), BQ (20 mol %), solvent (4 mL), 60 °C, constant current at 1.0 mA, 20 h, graphite felt (GF) anode, Pt-plate cathode. Without special note, yields and isomer ratios were determined by crude ^{1}H-NMR by using CH_2Br_2 as internal standard.

Supplementary Table 5 Ligand screening

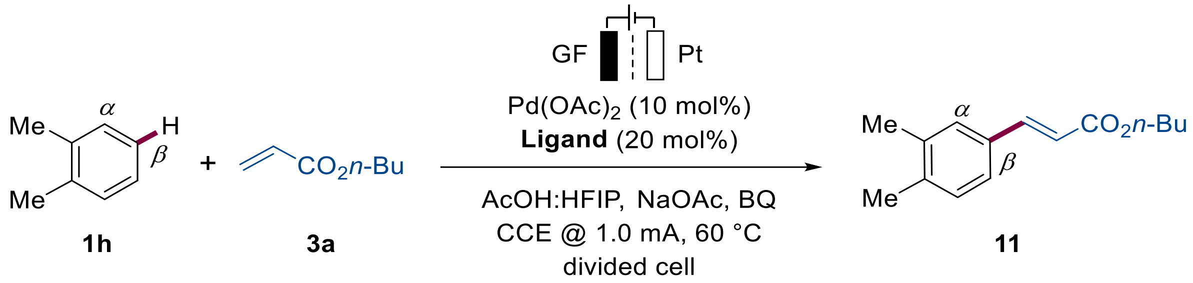

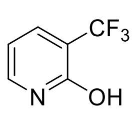
L1
28% (1:8)

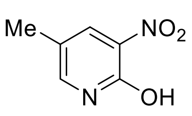
L2
69% (1:13)

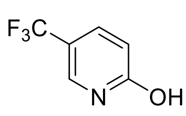
L3
73% (1:7)

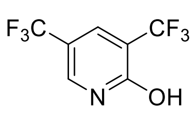
L4
40% (1:7)

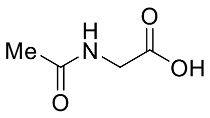
L5
37% (1:5)

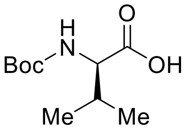
L6
36% (1:5)

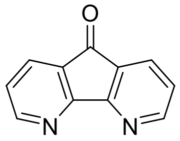
L7
15% (1:1.5)

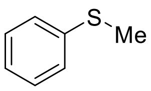
L8
25% (1:4)

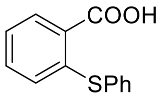
L9
63% (1:2)

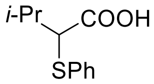
L10
30% (1:2.3)

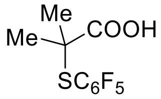
L11
72% (1:2.2)

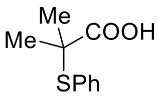
L12
99% (1:1.6)

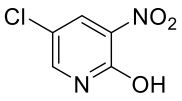
L13
47% (1:5)

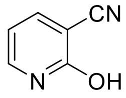
L14
32% (1:6)

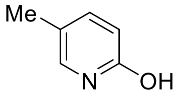
L15
32% (1:5)

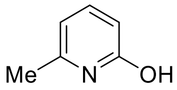
L16
36% (1:5)

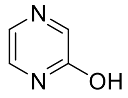
L17
trace

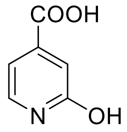
L18
32% (1:10)

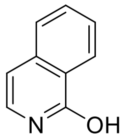
L19
26% (1:8)

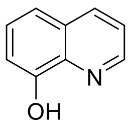
L20
trace

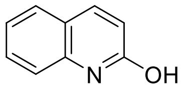
L21
51% (1:7)

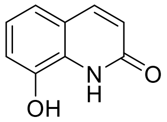
L22
25% (1:8)

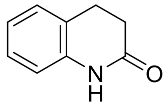
L23
52% (1:5)

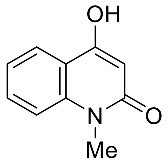
L24
55% (1:6)

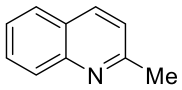
L25
44% (1:7)

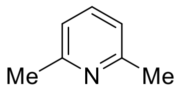
L26
40% (1:9)

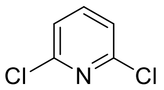
L27
44% (1:7)

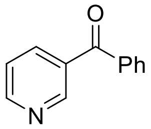
L28
7% (1:6)

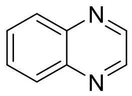
L29
trace

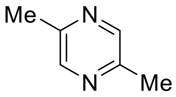
L30
4% (1:10)

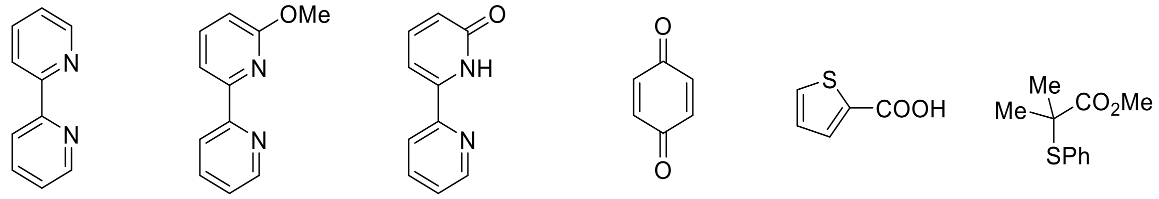

L31	L32	L33	L34	L35	L36
n.r.	n.r.	n.r.	36% (1:4)[a]	47% (1:5)	25% (1:1.8)

Reaction conditions: divided cell, anodic chamber: **1h** (4.0 mmol), **3a** (0.20 mmol), [Pd] (10 mol %), **Ligand** (20 mol %), NaOAc (4.0 equiv), BQ (20 mol %), HFIP:AcOH (1.3 mL:2.6 mL), cathodic chamber: NaOAc (4.0 equiv), BQ (20 mol %), HFIP:AcOH (1.3 mL:2.6 mL), 60 °C, constant current at 1.0 mA, 20 h, graphite felt (GF) anode, Pt-plate cathode. Without special note, yields and isomer ratios were determined by crude ^{1}H-NMR by using CH_2Br_2 as internal standard. α/β selectivities are given in the parentheses. [a] Without additional ligand.

Optimization Based on *S*,*O*-ligand

Supplementary Table 6 Different potentiostat

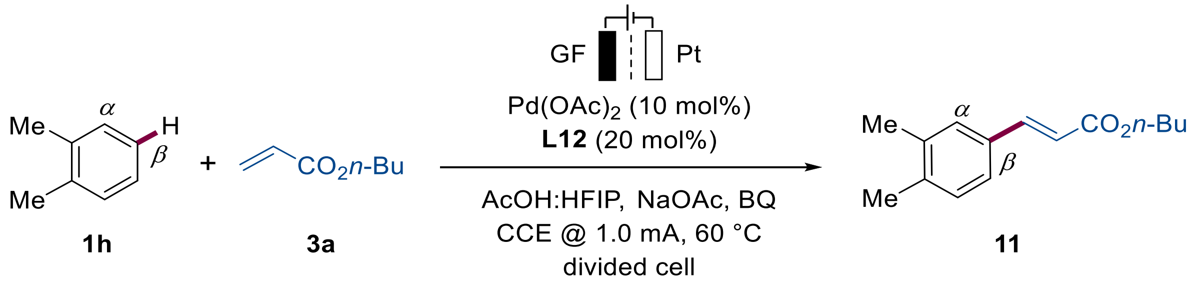

Entry	Potentiostat	Yield [%]	*α:β*
1	**S1**	**89**	**1:2**
2	**S2**	82	1:2

Reaction conditions: divided cell, anodic chamber: **1h** (1.0 mmol), **3a** (0.20 mmol), [Pd] (10 mol %), **L12** (20 mol %), BQ (20 mol %), NaOAc (4.0 equiv), HFIP:AcOH (1.3 mL:2.6 mL); cathodic chamber: BQ (20 mol %), NaOAc (4.0 equiv), HFIP:AcOH (1.3 mL:2.6 mL), 60 °C, constant current at 1.0 mA, 20 h, graphite felt (GF) anode, Pt-plate cathode. Without special note, yields and isomer ratios were determined by crude ^{1}H-NMR by using CH_2Br_2 as internal standard.

Supplementary Table 7 Supplemental control experiments

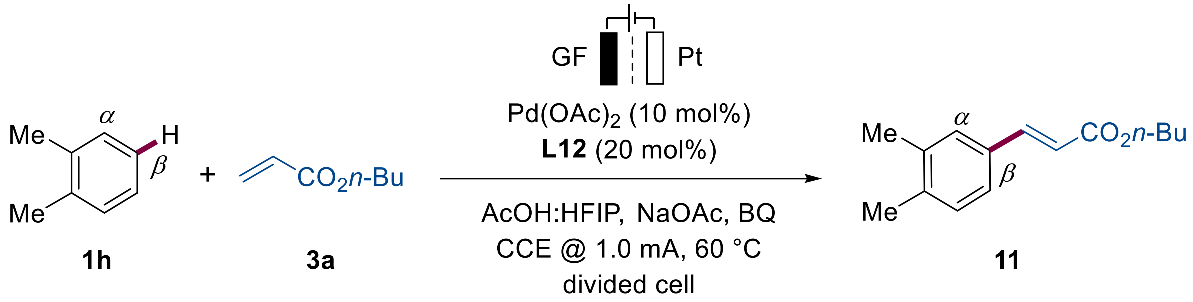

Entry	Deviations from standard condition	Yield [%]	*α:β*
1	none	95[a]	1:1.6
2	**1h** (5.0 equiv)	88[a]	1:1.6
3	*n*-Bu_4NOAc instead of NaOAc	--	--
4	TFE:AcOH as solvent	<5	1:1.3
5	No BQ	80	1:1.7
6	No $Pd(OAc)_2$	--	--
7	No **L12**	31	1:4
8	No electricity	30	1:1.6
9	40 °C	5	1:2

Reaction conditions: divided cell, anodic chamber: **1h** (2.0 mmol), **3a** (0.20 mmol), [Pd] (10 mol %), **L12** (20 mol %), BQ (20 mol %), NaOAc (4.0 equiv), HFIP:AcOH (1.3 mL:2.6 mL); cathodic chamber: BQ (20 mol %), NaOAc (4.0 equiv), HFIP:AcOH (1.3 mL:2.6 mL), 60 °C, constant current at 1.0 mA, 20 h, graphite felt (GF) anode, Pt-plate cathode. Without special note, yields and isomer ratios were determined by crude ^{1}H-NMR by using CH_2Br_2 as internal standard. [a] isolated yield; [b] anodic chamber: **1h** (1.0 mmol), **3a** (0.50 mmol), [Pd] (10 mol %), **L12** (20 mol %), BQ (5 mol %), NaOAc (50 mg), HFIP:AcOH (1.0 mL:2.0 mL); cathodic chamber: BQ (10 mol %), NaOAc (50 mg), HFIP:AcOH (1.0 mL:2.0 mL), 60 °C, constant current at 1.0 mA, 48 h.

Role of BQ

Supplementary Table 8 Control experiments for the role of air and BQ with L3

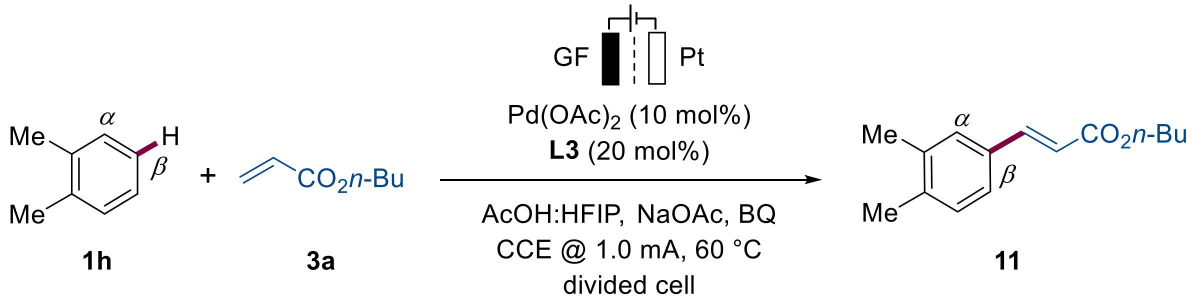

Entry	Deviation from standard conditions	Yield ($\beta : \alpha$)
1	none	69% (6 : 1)
2	No electricity	27% (7 : 1)
3	No BQ	37% (8 : 1)
4	Under N_2	38% (12 : 1)
5	1 eq. BQ instead of electricity, N_2	8% (---)

Reaction conditions: divided cell, anodic chamber: **1h** (4.0 mmol), **3a** (0.20 mmol), [Pd] (10 mol %), **L12** (20 mol %), BQ (20 mol %), NaOAc (4.0 equiv), HFIP:AcOH (1.3 mL:2.6 mL); cathodic chamber: BQ (20 mol %), NaOAc (4.0 equiv), HFIP:AcOH (1.3 mL:2.6 mL), 60 °C, constant current at 1.0 mA, 20 h, graphite felt (GF) anode, Pt-plate cathode. Without special note, yields and isomer ratios were determined by crude ^{1}H-NMR by using CH_2Br_2 as internal standard.

Supplementary Table 9 Control experiments for the role of air and BQ with L12

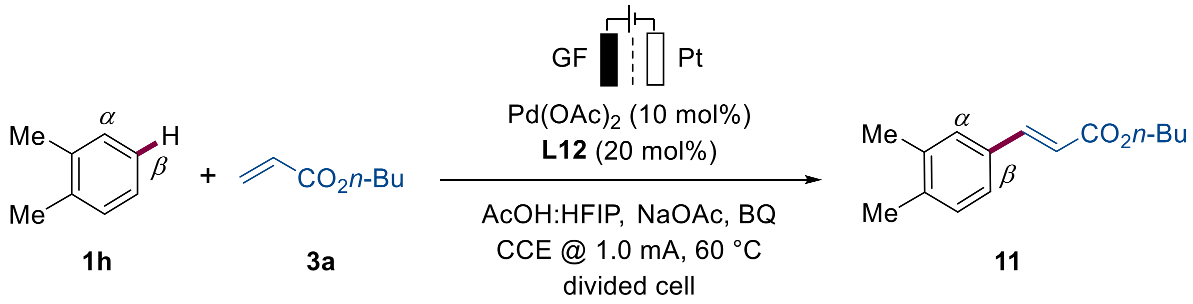

Entry	Deviation from standard conditions	Yield ($\beta : \alpha$)
1	none	88% (1.6:1)

2	No electricity	30% (1.6:1)
3	No electricity, no BQ	6% (1.6:1)
4	No BQ	80% (1.7:1)
5	Under N_2, No BQ	trace
6	1 eq. BQ instead of electricity	41% (1.5:1)
7	1 eq. BQ instead of electricity, N_2	27% (1.5:1)
8	Under O_2, no electricity, no BQ	14% (1.5:1)

Reaction conditions: divided cell, anodic chamber: **1h** (1.0 mmol), **3a** (0.20 mmol), [Pd] (10 mol %), **L12** (20 mol %), BQ (20 mol %), NaOAc (4.0 equiv), HFIP:AcOH (1.3 mL:2.6 mL); cathodic chamber: BQ (20 mol %), NaOAc (4.0 equiv), HFIP:AcOH (1.3 mL:2.6 mL), 60 °C, constant current at 1.0 mA, 20 h, graphite felt (GF) anode, Pt-plate cathode. Without special note, yields and isomer ratios were determined by crude ^{1}H-NMR by using CH_2Br_2 as internal standard.

Reduce the Loading of Arene

Supplementary Table 10 Optimization for lower equivalent of arene

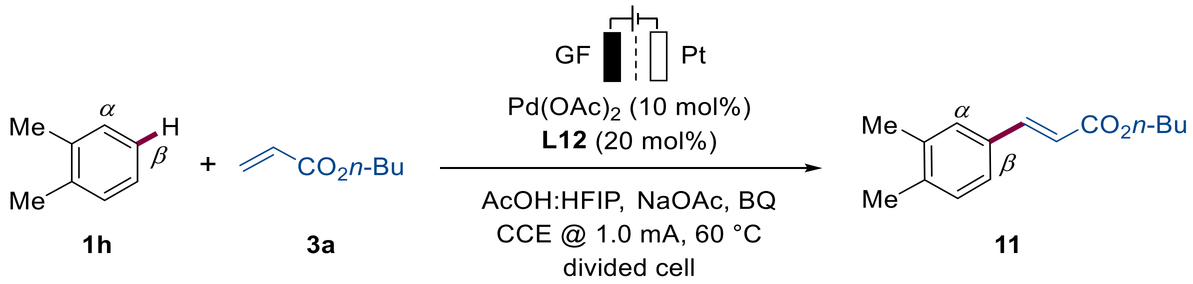

Entry	Deviations from standard condition	Yield [%]	*α*:*β*
1	none	75[a]	1:1.6
2	**1h** (0.5 mmol), **3a** (1.0 mmol)	34	1:1.2
3	**1h** (0.75 mmol), **3a** (0.5 mmol)	59[a]	1:1.5

Reaction conditions: divided cell, anodic chamber: **1h** (1.0 mmol), **3a** (0.50 mmol), [Pd] (10 mol %), **L12** (20 mol %), BQ (5 mol %), NaOAc (50 mg), HFIP:AcOH (1.0 mL:2.0 mL); cathodic chamber: BQ (5 mol %), NaOAc (50 mg), HFIP:AcOH (1.0 mL:2.0 mL), 60 °C, constant current at 1.0 mA, 48 h. graphite felt (GF) anode, Pt-plate cathode. Without special note, yields and isomer ratios were determined by crude ^{1}H-NMR by using CH_2Br_2 as internal standard. [a] isolated yield. The key factor for the reaction is the concentration of the arene

substrate, thus, our strategy is to increase the reaction scale (from 0.2 mmol to 0.5 mmol) and decrease the solvent amount. We did try other strategies like designing new reaction cells or employing fillings, but the selected method gave us optimized results.

Miscellaneous Studies

Mono- and Di-functionalized Product

Supplementary Table 11 Lower amount of arene cause C-H di-functionlization

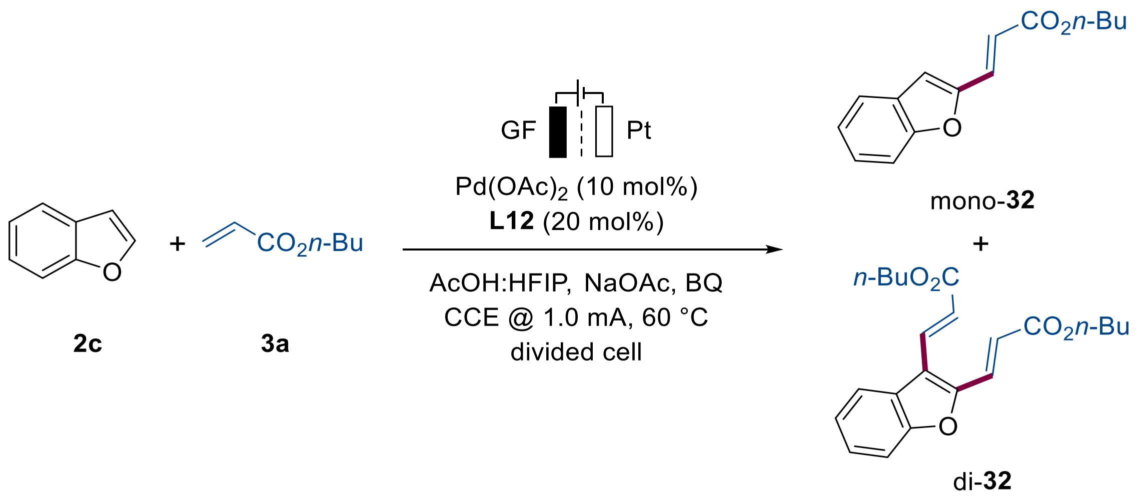

Entry	Amount of **2c:3a**	Yield (mono : di)
1	0.50 mmol :1.00 mmol	60% (1: 1)
2	0.75 mmol :0.50 mmol	75% (1.5: 1)[a]
3	1.50 mmol :0.50 mmol	76% (2.8: 1)

Reaction conditions: divided cell, anodic chamber: **2c** (x mmol), **3a** (x mmol), [Pd] (10 mol %), **L12** (20 mol %), BQ (10 mol %), NaOAc (0.2 M), HFIP:AcOH (1.0 mL:2.0 mL); cathodic chamber: BQ (10 mol %), NaOAc (0.2 M), HFIP:AcOH (1.0 mL:2.0 mL), 60 °C, constant current at 1.0 mA, 48 h, graphite felt (GF) anode, Pt-plate cathode. Without special note, yields and isomer ratios were determined by crude ^{1}H-NMR by using CH_2Br_2 as internal standard. [a] isolated yield. Lower equivalent of arene will lead to more di-functionalization product.

Other Metal Catalyst

Supplementary Table 12   Other metal catalyst

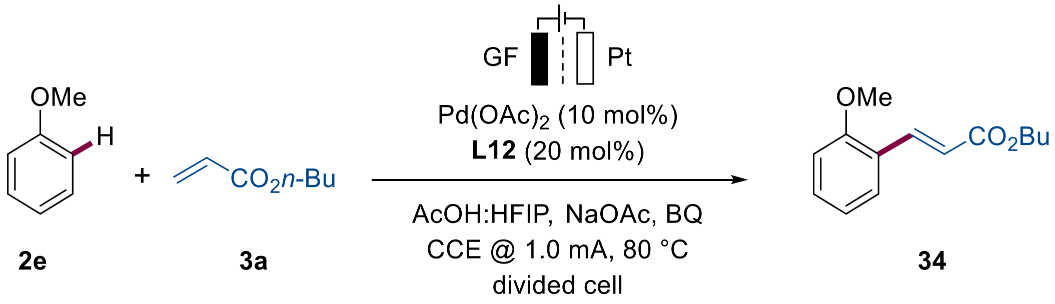

Entry	Metal catalyst	Yield (*o* : *m* : *p*)
1	Co(OAc)·4H_2O	n.d.
2	Ni(OAc)·4H_2O	n.d.

Reaction conditions: divided cell, anodic chamber: **2e** (0.25 mmol), **3a** (0.50 mmol), [Pd] (10 mol %), **L12** (20 mol %), BQ (20 mol %), NaOAc (4.0 equiv), HFIP:AcOH (1.3 mL:2.6 mL); cathodic chamber: BQ (20 mol %), NaOAc (4.0 equiv), HFIP:AcOH (1.3 mL:2.6 mL), 80 °C, constant current at 1.0 mA, 20 h, graphite felt (GF) anode, Pt-plate cathode. Without special note, yields and isomer ratios were determined by crude ^{1}H-NMR by using CH_2Br_2 as internal standard. n.d. refers to not detected.

Peroxide Test

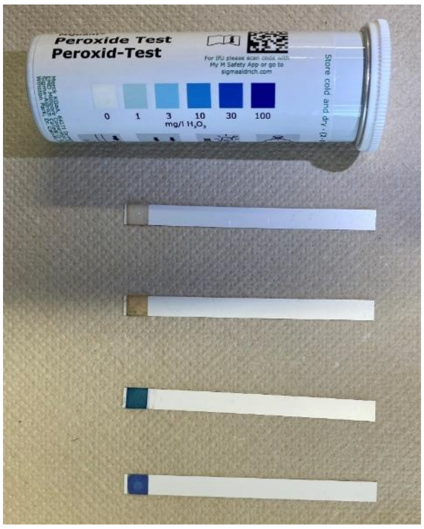

Supplementary Figure 7   Peroxide test

Peroxide tests were conducted with a semi-quantitative hydrogen peroxide test paper from Sigma-Aldrich (Method: Colorimetric, with test strips, 1 - 3 - 10 - 30 - 100 mg/L (H_2O_2), MQuant®). The detection method is on the package. A set of solutions were prepared for the peroxide test (from top to bottom in Supplementary Figure 7): a) reaction solution in cathodic cell after standard condition; b) reaction solution in anodic cell after standard condition; c) 5 μL 35% H_2O_2 in 2 mL HFIP:AcOH (1:2) with NaOAc; d) 35% H_2O_2 aqueous solution.

Cyclic Voltammetric Studies

CV measurements were conducted with a Metrohm Autolab PGSTAT204 potentiostat and Nova 2.1 software. A glassy carbon or platinum working electrode (disk, diameter: 3 mm), a coiled platinum wire counter electrode and a saturated calomel (SCE) reference electrode were employed. The voltammograms were recorded at room temperature in HFIP:AcOH (1.3 mL:2.6 mL) with 0.1 M $n\mathrm{Bu_4NPF_6}$ as supporting electrolyte. The scan rate is 100 mV/s. Deviations from the general experimental conditions are indicated in the respective figures and descriptions.

CV Studies of Pd and Ligand

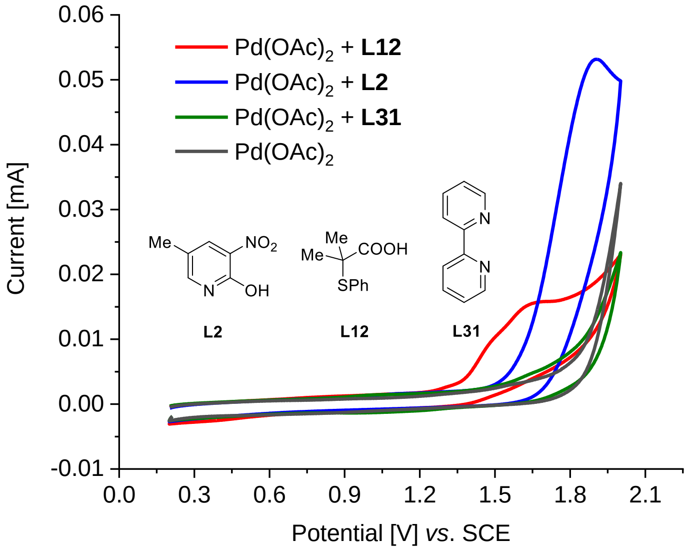

Supplementary Figure 8 CV studies of Pd and ligand. Cyclic voltammograms in HFIP:AcOH (1.3 mL:2.6 mL) with $n\mathrm{Bu_4NPF_6}$ (0.1 M) at 100 mV/s, $\mathrm{Pd(OAc)_2}$ (5.0 mM), ligand (10 mM); the catalyst and ligand mixtures were pre-stirred overnight before CV measurement.

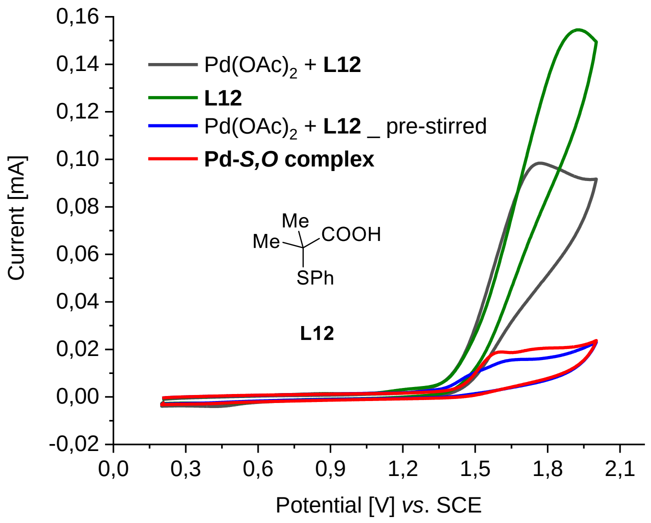

Supplementary Figure 9 CV studies of Pd, *S,O*-ligand and complex. Cyclic voltammograms in HFIP:AcOH (1.3 mL:2.6 mL) with nBu$_4$NPF$_6$ (0.1 M) at 100 mV/s, Pd(OAc)$_2$ (5.0 mM), **L12** (10 mM), **Pd-*S,O* complex** (1.5 mM); the catalyst and ligand mixtures were pre-stirred overnight before CV measurement.

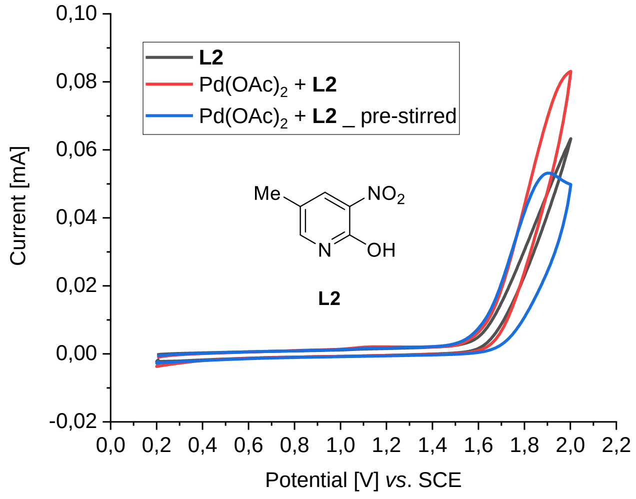

Supplementary Figure 10 CV studies of Pd and pyridone-ligand. Cyclic voltammograms in HFIP:AcOH (1.3 mL:2.6 mL) with nBu$_4$NPF$_6$ (0.1 M) at 100 mV/s, Pd(OAc)$_2$ (5.0 mM), **L22** (10 mM), the catalyst and ligand mixtures were pre-stirred overnight before CV measurement.

CV Studies of Product Isomers

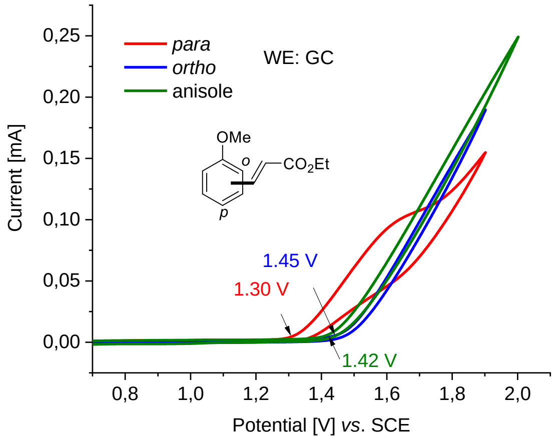

Supplementary Figure 11 CV studies of product isomers using glassy-carbon working electrode. Cyclic voltammograms in HFIP:AcOH (1.3 mL:2.6 mL) with *n*Bu_4NPF_6 (0.1 M) at 100 mV/s, the concentration of all the substrates is 10 mM.

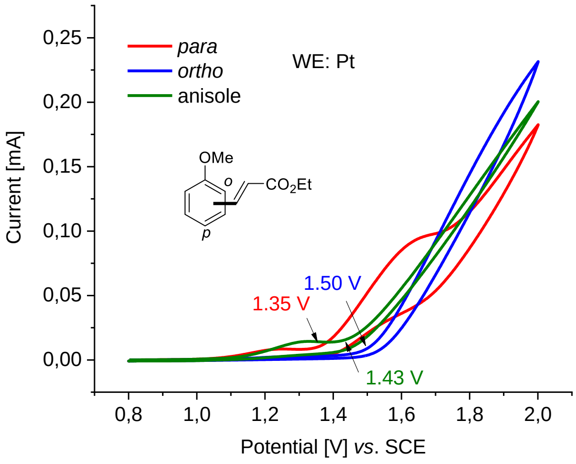

Supplementary Figure 12 CV studies of product isomers using Pt working electrode. Cyclic voltammograms in HFIP:AcOH (1.3 mL:2.6 mL) with *n*Bu_4NPF_6 (0.1 M) at 100 mV/s, the concentration of all the substrates is 10 mM. Pt was used as working electrode.

Despite the strong effect of electrode material in the outcome of the reaction, we couldn't reverse the selectivity at this point. To delineate its mode of impact, we conducted CV studies of *ortho* and *para* products with different electrode materials (Supplementary Figure 11 and Supplementary Figure 12). First of all, the results were intriguing which showed that the onset

potential difference of *ortho* and *para* products are similar. However, the onset potential was found to be lower on glass carbon electrode than that on Pt electrode, indicating *para* product can be more efficiently oxidized on carbon base electrode. Secondly, the new electrooxidation events (between 1.1 V and 1.4 V) due to the using of Pt electrode could obstruct the selective oxidation. Thirdly, we thought anisole play an important role in highly efficient selective oxidation as well, because the oxidation potential of anisole is higher than the one of *p*-product but lower than the one of *o*-product in the case of using GC, thus, the higher-concentration anisole could prevent *o*-product from oxidation by electricity and provide good selectivity. However, changing the electrode material to Pt could cause the onset potential of anisole shifts closer to the one of *p*-product, which could lead to competing oxidation between anisole and *p*-product, and thus inefficient oxidation of *p*-product. These three factors could explain how electrode material change the position-selectivity.

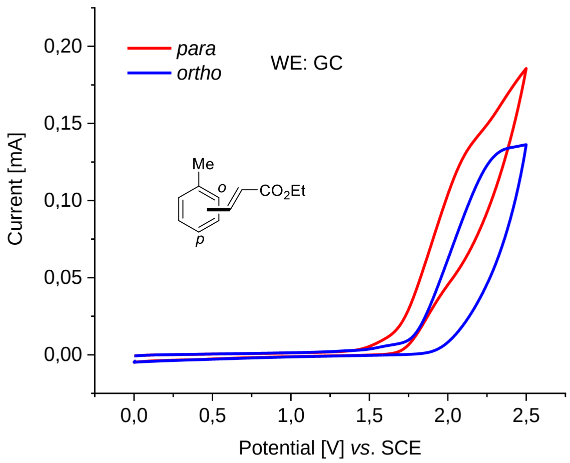

Supplementary Figure 13 CV studies of product isomers_toluene. Cyclic voltammograms in HFIP:AcOH (1.3 mL:2.6 mL) with *n*Bu_4NPF_6 (0.1 M) at 100 mV/s, the concentration of all the substrates is 10 mM.

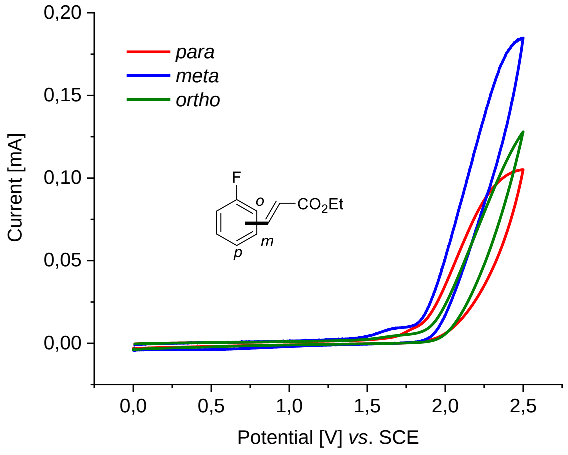

Supplementary Figure 14 CV studies of product isomers_fluorobenzene. Cyclic voltammograms in HFIP:AcOH (1.3 mL:2.6 mL) with *n*Bu_4NPF_6 (0.1 M) at 100 mV/s, the concentration of all the substrates is 10 mM.

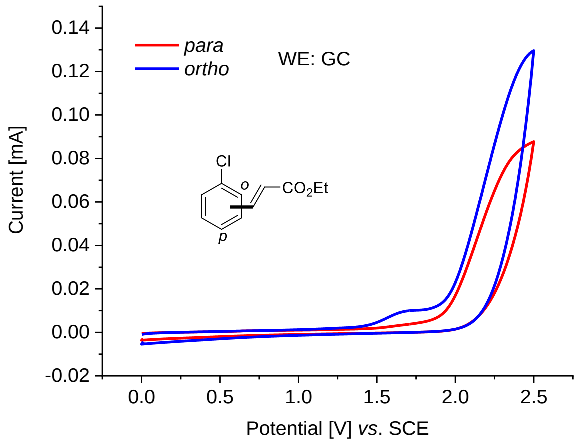

Supplementary Figure 15 CV studies of product isomers_chlorobenzene. Cyclic voltammograms in HFIP:AcOH (1.3 mL:2.6 mL) with *n*Bu_4NPF_6 (0.1 M) at 100 mV/s, the concentration of all the substrates is 10 mM.

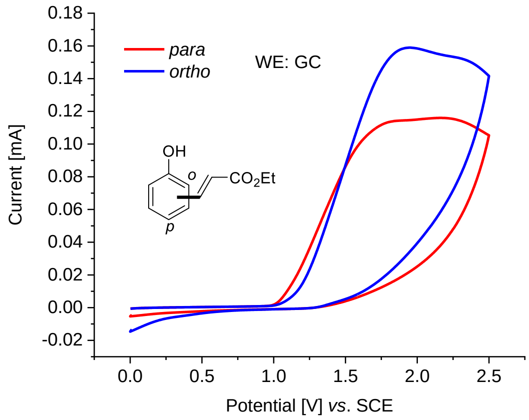

Supplementary Figure 16 CV studies of product isomers_phenol. Cyclic voltammograms in HFIP:AcOH (1.3 mL:2.6 mL) with *n*Bu_4NPF_6 (0.1 M) at 100 mV/s, the concentration of all the substrates is 10 mM.

Machine Learning for Predicting Position-Selectivity

Details of the Dataset Used in Machine Learning

49 data of the optimized palladium-electrochemical C−H olefinations of simple arenes devoid of exogenous directing groups were accumulated during the arenes screening. The details of the 49 data were elaborated in https://github.com/Shuwen-Li/Regioselectivity-Prediction.

Structural Optimization

To generate the 3D geometry for the recorded compounds, we used RDKit's[11] built-in ETKDG[12] method to generate the initial 3D structure. Subsequent geometric optimization was performed using the semiempirical extended tight-binding program package xTB[13], at the GFN2-xTB[14] level of theory. To obtain the Fukui functions and redox potential of arenes, the arenes were also optimized at the B3LYP/def2SVP level of theory. All the optimized geometries are available in our GitHub project (https://github.com/Shuwen-Li/Regioselectivity-Prediction).

Details of Descriptors

A series of steric and electronic features were applied to describe the arenes in the palladium-electrocatalyzed C−H olefinations, including buried volume, Sterimol, Fukui functions, atomic charge, homolytic bond dissociation energy, heterolytic bond dissociation energy, frontier molecular orbital, Wiberg Bond Order, bond length and redox potential.

Sterimol and buried volume were generated by moRFeus[15] based on GFN2-xTB-optimized geometry. Wiberg Bond Order and frontier molecular orbital were obtained based on GFN2-xTB-optimized geometry. Bond length were calculated based on GFN2-xTB-optimized geometry.

The bond dissociation energy of C–H bond in aldehyde were calculated as follows (Supplementary Figure 17).

$$\mathrm{R{-}H} \longrightarrow \mathrm{R}^{\bullet} + \mathrm{H}^{\bullet} \qquad \Delta H = \mathrm{BDE}$$

$$\mathrm{R{-}H} \longrightarrow \mathrm{R}^{+} + \mathrm{H}^{-} \qquad \Delta H = \mathrm{HBDE}$$

Supplementary Figure 17 The bond dissociation energy of C–H bond in aldehyde

Fukui functions of reacting carbon atom (f_c^{+}, f_c^{0} and f_c^{-}) were calculated as follows. q_C is the charge of reacting carbon atom and N is the number of electrons. Atomic charge in the neutral state, anionic state and cationic state generated at the B3LYP/def2SVP level of theory, using optimized geometry at GFN2-xTB level.

$$f_c^{+} = q_{\mathrm{C}}^{N+1} - q_{\mathrm{C}}^{N} \qquad (1)$$

$$f_c^{0} = (q_{\mathrm{C}}^{N+1} - q_{\mathrm{C}}^{N-1})/2 \qquad (2)$$

$$f_c^{-} = q_{\mathrm{C}}^{N} - q_{\mathrm{C}}^{N-1} \qquad (3)$$

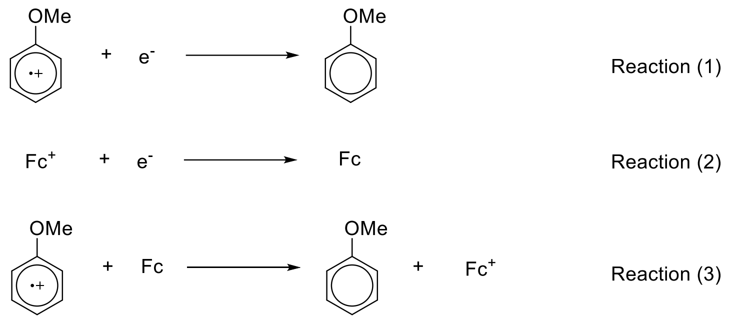

Supplementary Figure 18 Redox reactions of arenes and ferrocene

Redox potential of arenes were calculated as follows. Redox reactions of arenes and ferrocene were shown in Supplementary Figure 18, the redox potentials are $E_{\mathrm{Ar}^{+}/\mathrm{Ar}}$ and $E_{\mathrm{Fc}^{+}/\mathrm{Fc}}$ respectively, ΔG_{Ar} and ΔG_{Fc} represent the correspongding free energy. Reaction (1) minus reaction (2), reaction (3) was obtained. In reaction (3), the reduction of free energy $\Delta G_{\mathrm{Ar}} - \Delta G_{\mathrm{Fc}})$ is equal to the maximum of electrical work $n\mathrm{F}(E_{\mathrm{Ar}^{+}/\mathrm{Ar}} - E_{\mathrm{Fc}^{+}/\mathrm{Fc}})$ at constant temperature and pressure, so the equation (4) was obtained. Where n is the number of electron transfers in the redox equation, and F is Faraday constant. ΔG_{Ar} and ΔG_{Fe} were calculated based on B3LYP/def2TZVPP level of theory, using optimized geometry at B3LYP/def2SVP level. Using the experimental of ferrocene[16], the redox potential of arenes were obtained by equation (4).

$$-(\Delta G_{\mathrm{Ar}} - \Delta G_{\mathrm{Fe}}) = n\mathrm{F}(E_{\mathrm{Ar}^{+}/\mathrm{Ar}} - E_{\mathrm{Fe}^{+}/\mathrm{Fe}}) \quad (4)$$

The entire reaction encoding is a 28-dimensional physical organic space containing 8-dimensional steric information, 18-dimensional electronic information, 1-dimensional redox potential and 1-dimensional temperature. Full details of the used descriptors are elaborated in Supplementary Table 13.

Supplementary Table 13 Symbol, definition and access of descriptors

Entry	Molecul e	Symbol	Definition	Access
1	Arene	$\%V_{Bur}^{3.5Å}$@A	The buried volume of the reacting carbon atom at arenes, sphere radius = 3.5Å (position A)	Calculation based on xTB optimized geometries
2	Arene	B_1@A	Sterimol parameter B_1 of the hydrogen atom near reacting carbon atom at arenes, dummy index: hydrogen-atom, attached index: atom in substituent group attach to hydrogen-atom (position A)	Calculation based on xTB optimized geometries

3	Arene	B_5@A	Sterimol parameter B_5 of the hydrogen atom near reacting carbon atom at arenes, dummy index: hydrogen-atom, attached index: atom in substituent group attach to hydrogen-atom (position A)	Calculation based on xTB optimized geometries
4	Arene	L@A	Sterimol parameter L of the hydrogen atom near reacting carbon atom at arenes, dummy index: hydrogen-atom, attached index: atom in substituent group attach to hydrogen-atom (position A)	Calculation based on xTB optimized geometries
5	Arene	$\%V_{Bur}^{3.5Å}$@B	The buried volume of the reacting carbon atom at arenes, sphere radius = 3.5Å (position B)	Calculation based on xTB optimized geometries
6	Arene	B_1@ B	Sterimol parameter B_1 of the hydrogen atom near reacting carbon atom at arenes, dummy index: hydrogen-atom, attached index: atom in substituent group attach to hydrogen-atom (position B)	Calculation based on xTB optimized geometries
7	Arene	B_5@ B	Sterimol parameter B_5 of the hydrogen atom near reacting carbon atom at arenes, dummy index: hydrogen-atom, attached index: atom in substituent group attach to hydrogen-atom (position B)	Calculation based on xTB optimized geometries
8	Arene	L@ B	Sterimol parameter L of the hydrogen atom near reacting carbon atom at arenes, dummy index: hydrogen-atom, attached index: atom in substituent group attach to hydrogen-atom (position B)	Calculation based on xTB optimized geometries
9	Arene	q@ A	Charge of reacting carbon atom at arenes (position A)	Based on xTB optimization
10	Arene	q@ B	Charge of reacting carbon atom at arenes (position B)	Based on xTB optimization
11	Arene	f_c^+@ A	Fukui function of the reacting carbon atom of arenes (position A)	$f_c^+(Ar)_A = q_C^{N+1} - q_C^N$
12	Arene	f_c^+@ B	Fukui function of the reacting carbon atom of arenes (position B)	$f_c^+(Ar)_B = q_C^{N+1} - q_C^N$
13	Arene	f_c^0@ A	Fukui function of the reacting carbon atom of arenes (position A)	$f_c^0(Ar)_A = (q_C^{N+1} - q_C^{N-1})/2$
14	Arene	f_c^0@ B	Fukui function of the reacting carbon atom of arenes (position B)	$f_c^0(Ar)_B = (q_C^{N+1} - q_C^{N-1})/2$
15	Arene	f_c^-@ A	Fukui function of the reacting carbon atom of arenes (position A)	$f_c^-(Ar)_A = q_C^N - q_C^{N-1}$
16	Arene	f_c^-@ B	Fukui function of the reacting carbon atom of arenes (position B)	$f_c^0(Ar)_B = q_C^N - q_C^{N-1}$
17	Arene	D_{C-H}@ A	The homolytic bond dissociation energy of the reacting C–H bond of arenes (position A)	DFT calculation based on xTB optimized geometries
18	Arene	D_{C-H}@ B	The homolytic bond dissociation energy of the reacting C–H bond of arenes (position B)	DFT calculation based on xTB optimized geometries
19	Arene	$D_{C^-H^+}$@ A	The heterolytic bond dissociation energy of the reacting C–H bond of arenes (position A)	DFT calculation based on xTB optimized geometries

20	Arene	D_{C-H^+}@ B	The heterolytic bond dissociation energy of the reacting C–H bond of arenes (position B)	DFT calculation based on xTB optimized geometries
21	Arene	B_{C-H}@ A	Wiberg Bond Order of C-H bond involving the reacting carbon atom of arenes (position A)	Based on xTB optimization
22	Arene	B_{C-H}@ B	Wiberg Bond Order of C-H bond involving the reacting carbon atom of arenes (position B)	Based on xTB optimization
23	Arene	d_{C-H}@ A	Bond length of C-H bond involving the reacting carbon atom of arenes (position A)	Based on xTB optimized geometries
24	Arene	d_{C-H}@ B	Bond length of C-H bond involving the reacting carbon atom of arenes (position B)	Based on xTB optimized geometries
25	Arene	π^{HOMO}@ A	The energy of π occupied molecular orbital of arenes	Based on xTB optimization
26	Arene	π^{HOMO}@ B	The energy of π unoccupied molecular orbital of arenes	Based on xTB optimization
27	Arene	Redox potential	Redox potential value	Calculation based on DFT optimization
28		Temperature	Reaction temperature	Temperature value

Details of Machine Learning Algorithms

A series of widely used machine learning algorithms were tested for model training, including Bagging regression[17], Decision Trees[18], Extra-Trees[19], Gradient Boosting[20], k-Nearest Neighbors regression[21], Kernel Ridge regression[22], Linear Support Vector Regression[23], Random Forest Regression[24], Ridge[25], Support Vector Regression[23] and XGBoost[26]. The model training was performed using scikit-learn[27] and xgboost python packages[28]. The parameters of each tested algorithm were included in Supplementary Table 14. All the related scripts for model training were available in our GitHub project (https://github.com/Shuwen-Li/Regioselectivity-Prediction).

Supplementary Table 14 The hyper-parameters of the tested machine learning algorithms for model training.

Model	Modules and parameters
Bagging (BG)	sklearn.ensemble.BaggingRegressor(base_estimator=None, n_estimators=10, max_samples=1.0, max_features=1.0, bootstrap=True, bootstrap_features=False, oob_score=False, warm_start=False, n_jobs=60, random_state=None, verbose=0)
Decision Tree (DT)	sklearn.tree.DecisionTreeRegressor(criterion='mse', splitter='best', max_depth=None, min_samples_split=2, min_samples_leaf=1, min_weight_fraction_leaf=0.0, max_features=None, random_state=None, max_leaf_nodes=None, min_impurity_decrease=0.0, min_impurity_split=None, presort='deprecated', ccp_alpha=0.0)
Extra-Trees (ET)	sklearn.ensemble.ExtraTreesRegressor(n_estimators=100, criterion='mse', max_depth=None, min_samples_split=2, min_samples_leaf=1, min_weight_fraction_leaf=0.0, max_features='auto', max_leaf_nodes=None, min_impurity_decrease=0.0, bootstrap=False, oob_score=False, n_jobs=60, random_state=None, verbose=0, warm_start=False, ccp_alpha=0.0, max_samples=None)
Gradient Boosting (GB)	sklearn.ensemble.GradientBoostingRegressor(loss='ls', learning_rate=0.1, n_estimators=100, subsample=1.0, criterion='friedman_mse', min_samples_split=2, min_samples_leaf=1, min_weight_fraction_leaf=0.0, max_depth=3, min_impurity_decrease=0.0, init=None, random_state=None, max_features=None, alpha=0.9, verbose=0, max_leaf_nodes=None, warm_start=False, validation_fraction=0.1, n_iter_no_change=None, tol=0.0001, ccp_alpha=0.0)

k-Nearest Neighbors Regression (KNR)	sklearn.neighbors.NearestNeighbors(n_neighbors=5,weights='uniform',algorithm='auto',leaf_size=30,p=2,metric='minkowski',metric_params=None,n_jobs=None)
KernelRidge (KRR)	sklearn.kernel_ridge.KernelRidge(alpha=1, kernel='linear', gamma= None, degree=3, coef0=1, kernel_params=None)
Linear Support Vector Regression (LSVR)	sklearn.svm.LinearSVR(epsilon=0.0,tol=0.0001,C=1.0,loss='epsilon_insensitive',fit_intercept=True,intercept_scaling=1.0,dual=True,verbose=0,random_state=None,max_iter=1000)
RandomForest (RF)	sklearn.ensemble.RandomForestRegressor(n_estimators=100,criterion='mse',max_depth=None,min_samples_split=2,min_samples_leaf=1,min_weight_fraction_leaf=0.0,max_features='auto',max_leaf_nodes=None,min_impurity_decrease=0.0,bootstrap=True,oob_score=False,n_jobs=60,random_state=None,verbose=0,warm_start=False, ccp_alpha=0.0,max_samples=None)
Ridge	sklearn.linear_model.Ridge(alpha=1.0,fit_intercept=True,copy_X=True,max_iter=None,tol=0.001)
Support Vector Regression (SVR)	sklearn.svm.SVR(kernel='rbf', degree=3, gamma='scale', coef0=0.0, tol=0.001, C=1.0, epsilon=0.1, shrinking=True, cache_size=200, verbose=False, max_iter=- 1)
XGBoost (XGB)	xgboost.XGBRegressor(base_score=0.5, oster='gbtree', olsample_bylevel=1, colsample_bynode=1, colsample_bytree=1, gamma=0, gpu_id=-1, importance_type='gain', interaction_constraints='', learning_rate=0.3, max_delta_step=0, max_depth=10, min_child_weight=1, missing=np.nan, monotone_constraints='()', n_estimators=60, num_parallel_tree=1, random_state=0, reg_alpha=0, reg_lambda=1, scale_pos_weight=1, subsample=1, tree_method='exact', validate_parameters=1, verbosity=None)

Details of Model Predictions

Model Selection

Eleven regression algorithms (Bagging Regression, Decision Trees, Extra-Trees, Gradient Boosting, k-Nearest Neighbors Regression, Kernel Ridge Regression, Linear Support Vector Regression, Random Forest Regression, Ridge, Support Vector Regression, and XGBoost) were tested for the regioselectivity prediction, using the full 28-dimensional encodings. The Pearson Rs and MAEs in leave-one-out are shown in Supplementary Table 15.

Supplementary Table 15 Pearson R and MAE of the regioselectivity predictions in leave-one-out using various algorithms.

Model	MAE (kcal/mol)	Pearson R
Bagging	0.7874	0.7523
Decision Tree	0.7480	0.7865
Extra-Trees	0.6614	0.8690
Gradient Boosting	0.6505	0.8060
k-Nearest Neighbors Regression	1.0390	0.4994
Kernel Ridge Regression	0.7981	0.7314
Linear Support Vector Regression	0.7255	0.7960
Random Forest	0.7775	0.7906
Ridge	0.7693	0.7357
Support Vector Regression	0.9779	0.6157
XGBoost	0.6330	0.8303

Descriptor Selection

Using Extra-Trees which is the optimal algorithm for leave-one-out prediction, descriptor selection was performed. For each round of descriptor addition, the highest performance-improving descriptor was selected and added until no further improvement can be achieved. The regression performances prior to and after the descriptor selection are shown in Supplementary Figure 19. Python script is available at GitHub: https://github.com/Shuwen-Li/Regioselectivity-Prediction.

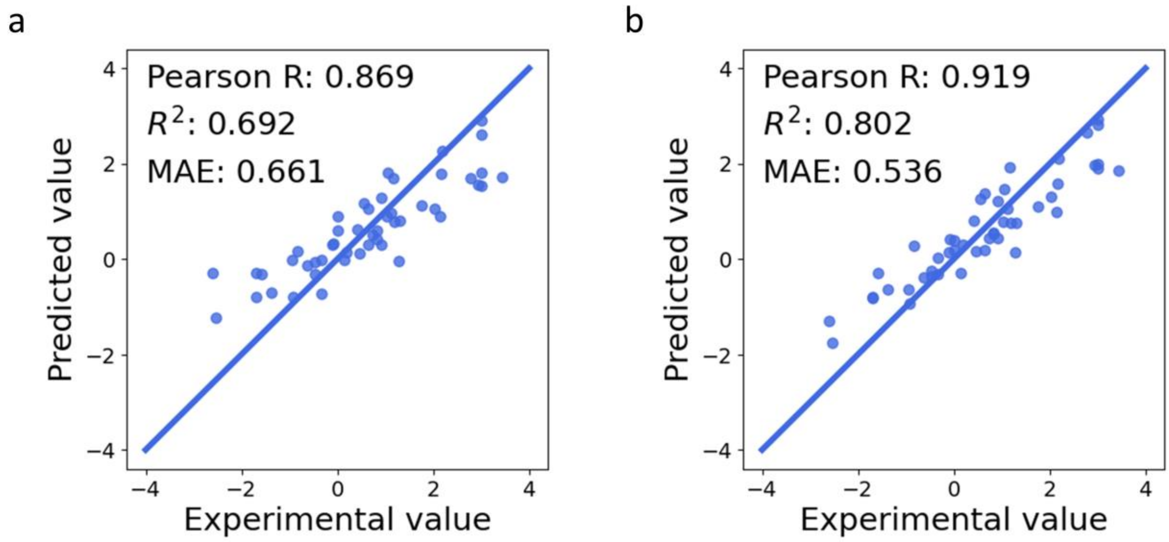

Supplementary Figure 19 Regression performances. Regression performances prior to the descriptor selection (a) and after descriptor selection (b).

Details of the Out-of-sample Prediction for Arenes

The trained model was used to predict the regioselectivity in a number of out-of-sample tests. By selecting these compounds out, the model did not access to the encodings of these C-H sites during the model training. Results of the out-of-sample regioselectivity prediction were shown as follows.

Supplementary Table 16 Experimental and predicted regioselectivities in the out-of-sample prediction task

Entry	Arenes	Sites	Experimental ratio	Predicted ratio
1	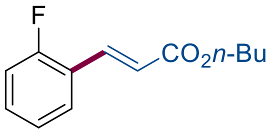	*o*:*p*	2.5	3.4
2	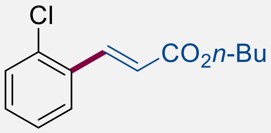	*o*:*m*	3.1	2.1
3	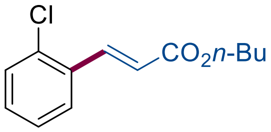	*o*:*p*	3.7	2.0
4	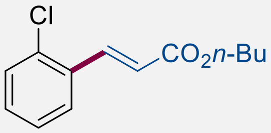	*m*:*p*	1.2	0.9
5	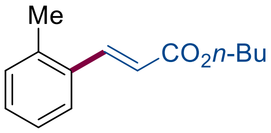	*o*:*m*	2.1	1.7
6	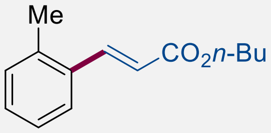	*o*:*p*	1.9	1.2
7	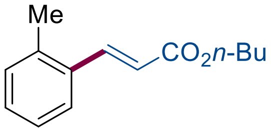	*m*:*p*	0.9	0.8

8	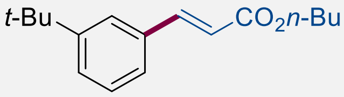	*m*:*p*	1.0	1.2
9	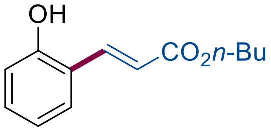	*o*:*p*	3.2	6.9
10	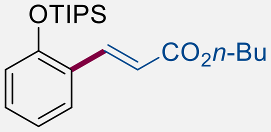	*o*:*p*	1.0	1.5
11	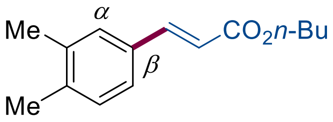	$\beta:\alpha$	1.6	1.4
12	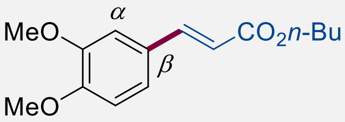	$\beta:\alpha$	5.4	2.3
13	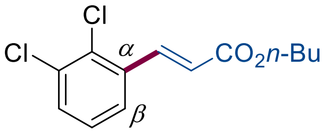	$\beta:\alpha$	0.7	0.5
14	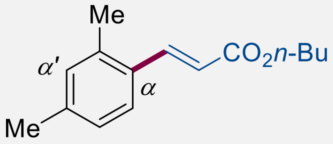	$\alpha':\alpha$	0.2	0.4
15	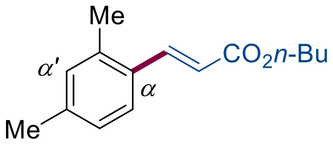	$\alpha':\beta$	1.6	1.2
16	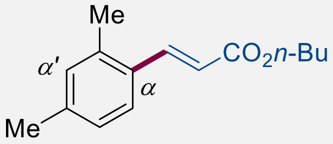	$\alpha:\beta$	8.8	5.0
17	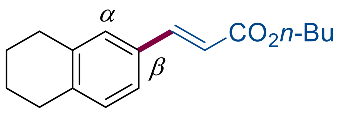	$\beta:\alpha:\alpha'$	1.4	1.4
18	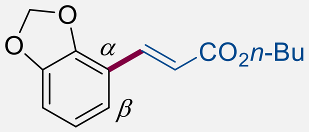	$\beta:\alpha$	0.5	0.3

19	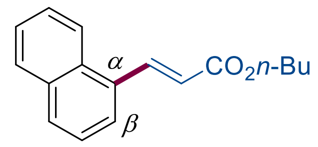	$\beta:\alpha$	0.4	0.5
20	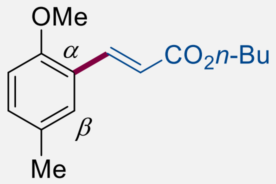	$\beta:\alpha$	19.0	7.2
21	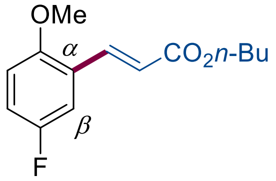	$\beta:\alpha$	3.6	1.2
22	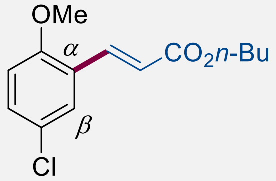	$\beta:\alpha$	7.6	3.7
23	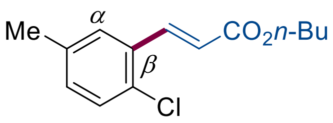	$\beta:\alpha$	0.4	1.3
24	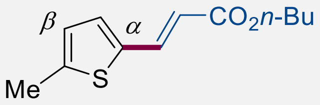	$\alpha:\beta$	5.8	3.0
25	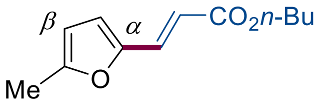	$\alpha:\beta$	20.0	7.4
26	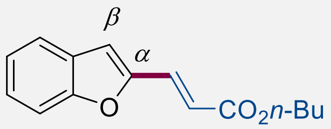	$\beta:\alpha$	0.4	0.6
27	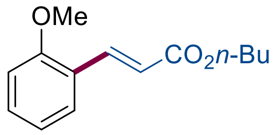	*o*:*p*	16.0	14.2
28	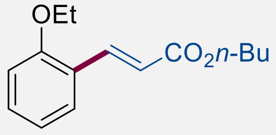	*o*:*p*	20.0	18.8

29	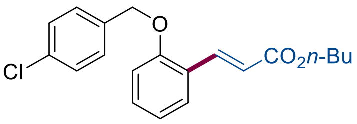	*o*:*p*	9.0	8.3
30	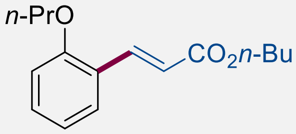	*o*:*p*	20.0	16.6
31	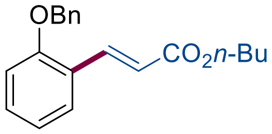	*o*:*p*	20.0	6.7
32	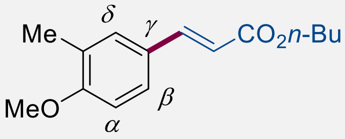	$\alpha:\beta$	0.9	1.2
33	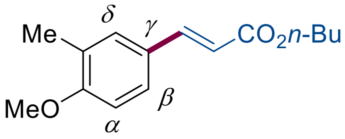	$\alpha:\gamma$	0.1	1.0
34	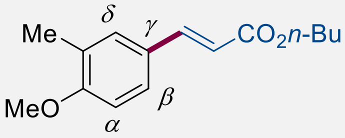	$\alpha:\delta$	2.3	1.6
35	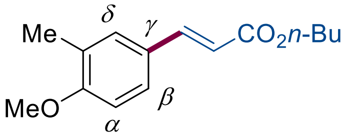	$\beta:\gamma$	0.1	0.6
36	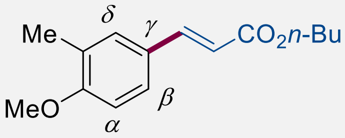	$\beta:\delta$	2.5	1.0
37	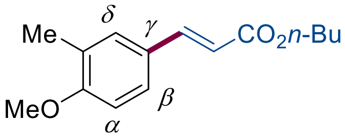	$\gamma:\delta$	31.4	5.0
38	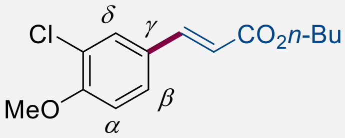	$\alpha:\beta$	0.6	1.4

39	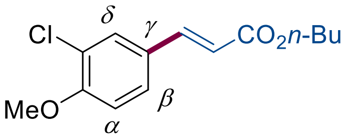	$\alpha:\gamma$	0.3	1.2
40	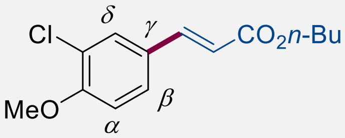	$\alpha:\delta$	0.7	1.6
41	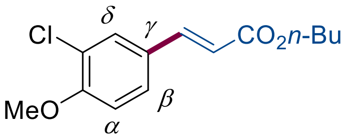	$\beta:\gamma$	0.4	0.9
42	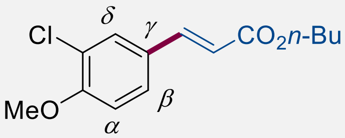	$\beta:\delta$	1.1	1.2
43	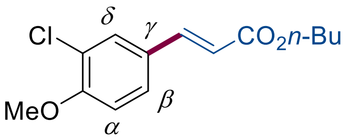	$\gamma:\delta$	2.9	5.5
44	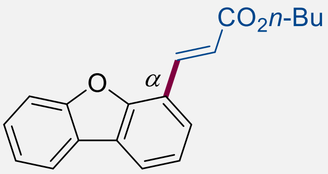	$\alpha:\beta$	8.5	2.5
45	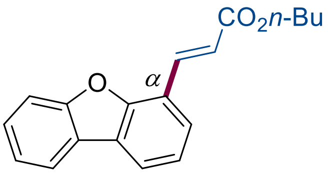	$\alpha:\gamma$	3.3	1.5
46	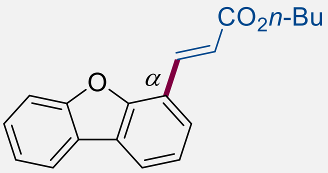	$\alpha:\delta$	1.7	2.3
47	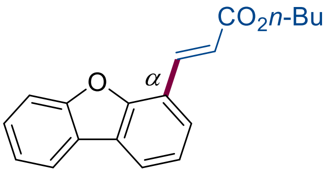	$\beta:\gamma$	0.4	2.0

48	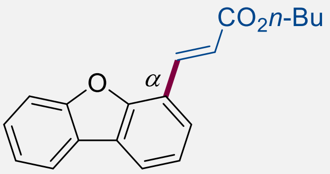	$\beta:\delta$	0.2	3.1
49	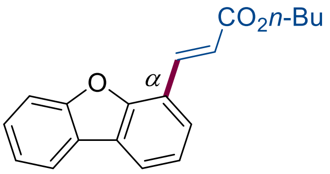	$\gamma:\delta$	0.5	1.2

Feature Importance

To determine the critical paramters that are responsible for the regioselectivity prediction, we evaluated the feature importance scores of the trained model. Supplementary Figure 20 elaborates the feature importance scores of the top-ranking features, in which the Fukui function of the reacting site is the most influential property for the model output.

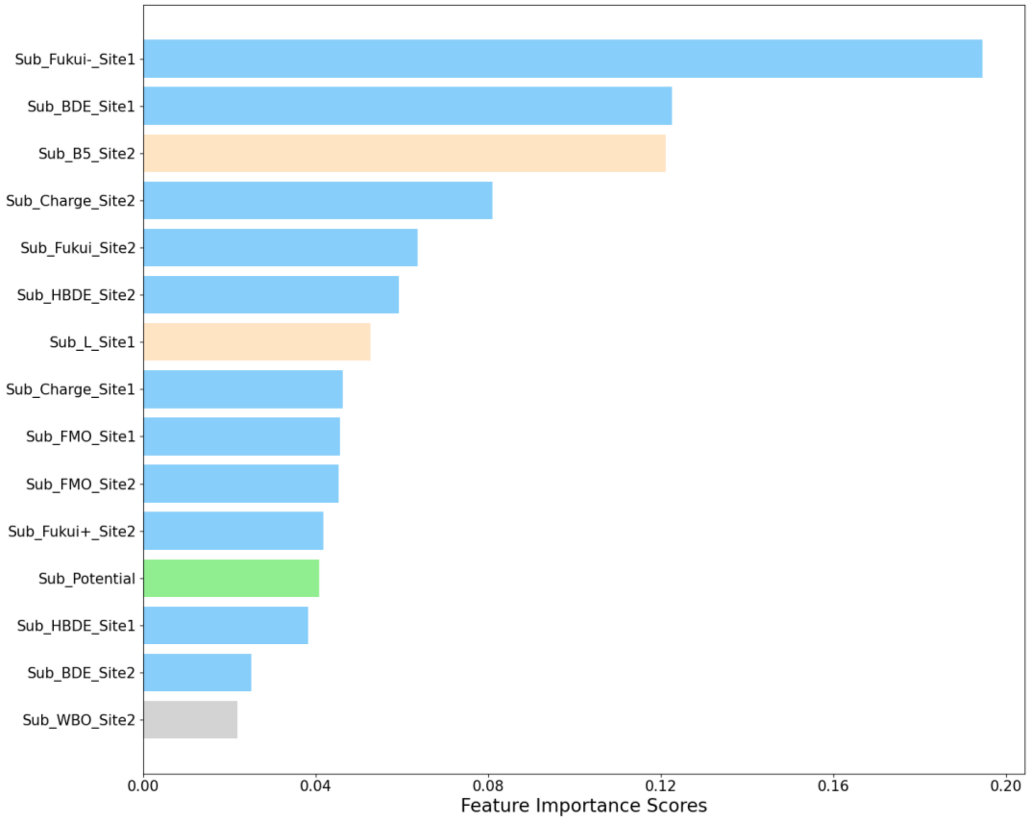

Supplementary Figure 21 Feature importance scores of top-ranking features.

Data and Code Availability

All the involved codes and data in this study were freely available at https://github.com/Shuwen-Li/Regioselectivity-Prediction.

External Experimental Verification

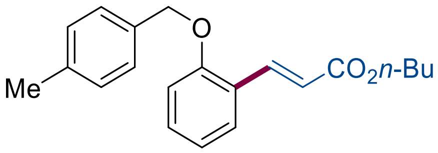

(*E*)-*n*-butyl-3-(2-((4-methylbenzyl)oxy)phenyl)acrylate (*o*-ML1)

The general procedure **A** was followed using 1-methyl-4-(phenoxymethyl)benzene (**2j**) (198.3 mg, 1.0 mmol, 5.0 equiv) and *n*-butyl acrylate (**3a**) (28.8 µL, 0.20 mmol, 1.0 equiv) at 60 °C for 24 h. **L12** was used as ligand. Isolation by column chromatography (*n*-hexane/EtOAc = 30:1 to 15:1) yielded **ML1** (28.2 mg, 43%) as a colorless oil. The ratio of the isomers was determined by the ^{1}H-NMR of the crude mixture, *o* : *p* = 6.3 : 1. Functionalization on the benzyl group was also observed. For *o*- **ML1**, ^{1}H-NMR (400 MHz, $CDCl_3$): δ = 8.08 (d, J = 16.1 Hz, 1H), 7.56 – 7.52 (m, 1H), 7.35 – 7.30 (m, 3H), 7.22 – 7.18 (m, 2H), 6.98 – 6.93 (m, 2H), 6.54 (d, J = 16.2 Hz, 1H), 5.13 (s, 2H), 4.20 (t, J = 6.6 Hz, 2H), 2.37 (s, 4H), 1.76 – 1.64 (m, 3H), 1.51 – 1.38 (m, 3H), 0.97 (t, J = 7.4 Hz, 3H). ^{13}C-NMR (100 MHz, $CDCl_3$): δ = 167.5 (C_q), 157.3 (C_q), 139.8 (CH), 137.7 (C_q), 133.5 (C_q), 131.3 (CH), 129.2 (CH), 128.7 (CH), 127.2 (CH), 123.9 (C_q), 120.9 (CH), 118.8 (CH), 112.7 (CH), 70.3 (CH_2), 64.2 (CH_2), 30.8 (CH_2), 21.2 (CH_3), 19.2 (CH_2), 13.7 (CH_3). IR (ATR): 2958, 2931, 2872, 1708, 1631, 1599, 1455, 1315, 1241, 1168 cm^{-1}. MS (ESI) *m*/*z* (relative intensity): 325 (30) $[M + H]^+$, 347 (100) $[M + Na]^+$. HR-MS (ESI): *m*/*z* calcd. for $[C_{21}H_{24}O_3 + Na]^+$ 347.1618 found 347.1617.

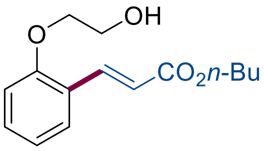

(*E*)-*n*-butyl-3-(2-(2-hydroxyethoxy)phenyl)acrylate (*o*-ML2) & (*E*)-*n*-butyl-3-(4-(2-hydroxyethoxy)phenyl)acrylate (*p*- ML2)

The general procedure **A** was followed using 2-phenoxyethan-1-ol (**2k**) (124.5 µL, 1.0 mmol, 5.0 equiv) and *n*-butyl acrylate (**3a**) (28.8 µL, 0.20 mmol, 1.0 equiv) at 60 °C for 20 h. **L12**

was used as ligand. Isolation by column chromatography (*n*-hexane/EtOAc = 3:1) yielded **ML2** (30.1 mg, 51%) as a mixture. Esterification of the products was also observed. The site selectivity was determined by ^{1}H-NMR and COSY NMR, *o* : *m* : *p* = 8.8 : 0.2 : 1. ^{1}H-NMR (400 MHz, $CDCl_3$): δ = 8.03 (d, *J* = 16.2 Hz, 1H^o), 7.66 – 7.60 (m, 1H^{m+p}), 7.53 (dd, *J* = 7.7, 1.7 Hz, 1H^o), 7.47 (d, *J* = 8.8 Hz, 2H^p), 7.33 (ddd, *J* = 8.7, 7.4, 1.8 Hz, 1H^o), 6.98 (td, *J* = 7.5, 1.1 Hz, 1H^o), 6.95 – 6.89 (m, 1H^o+2H^p), 6.50 (d, J = 16.2 Hz, 1H^o), 6.42 (d, J = 16.0 Hz, 1H^m), 6.31 (d, *J* = 16.0 Hz, 1H^p), 4.23 – 4.17 (m, 2H^{o+m+p}), 4.17 – 4.13 (m, 2H^o), 4.13 – 4.10 (m, 2H^p), 4.04 – 4.01 (m, 2H^o), 4.00 – 3.96 (m, 2H^p), 2.42 (s, 1H^{o+m+p}), 1.81 (s, 1H^{o+m+p}), 1.74 – 1.63 (m, 2H^{o+m+p}), 1.51 – 1.37 (m, 2H^{o+m+p}), 1.01 – 0.90 (m, 3H^{o+m+p}). For *o*- **ML2**, ^{13}C-NMR (101 MHz, $CDCl_3$): δ =167.7 (C_q), 157.3 (C_q), 139.7 (CH), 131.5 (CH), 128.6 (CH), 123.7 (C_q), 121.2 (CH), 118.7 (CH), 112.4 (CH), 70.0 (CH_2), 64.4 (CH_2), 61.4 (CH_2), 30.8 (CH_2), 19.2 (CH_2), 13.8 (CH_3). IR (ATR): 3428, 2959, 2934, 2874, 1707, 16307, 1452, 1319, 1246, 1171 cm^{-1}. MS (ESI) *m*/*z* (relative intensity): 287 (100) [M + Na]$^+$. HR-MS (ESI): *m*/*z* calcd. for $[C_{15}H_{20}O_4 + Na]^+$ 287.1254 found 287.1263.

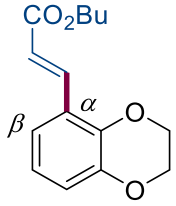

(*E*)-*n*-butyl-3-(2,3-dihydrobenzo[*b*][1,4]dioxin-5-yl)acrylate (α-ML3) &(*E*)-*n*-butyl-3-(2,3-dihydrobenzo[*b*][1,4]dioxin-6-yl)acrylate (β-ML3)

The general procedure **A** was followed using benzo-1,4-dioxane (**2l**) (119.4 µL, 1.0 mmol, 5 equiv.) and *n*-butyl acrylate (**3a**) (28.8 µL, 1.0 mmol, 1.0 equiv.) at 60 °C for 20 h. **L12** was used as ligand. Isolation by column chromatography (*n*-hexane/EtOAc = 5:1) yielded **ML3** (51.4 mg, 98%) as a mixture. The site selectivity was determined by ^{1}H-NMR and HMBC NMR, α:β= 3.4:1. ^{1}H-NMR (600 MHz, $CDCl_3$): δ = 7.89 (d, *J* = 16.2 Hz, 1H$^\alpha$), 7.56 (d, *J* = 15.9 Hz, 1H$^\beta$), 7.07 (ddd, *J* = 7.7, 1.6, 0.6 Hz, 1H$^\alpha$), 7.05 (d, *J* = 2.1 Hz, 1H$^\beta$), 7.02 (dd, *J* = 8.5, 2.0 Hz, 1H$^\beta$), 6.88 (dd, *J* = 8.1, 1.6 Hz, 1H$^\alpha$), 6.85 (d, *J* = 8.4 Hz, 1H$^\beta$), 6.82 (t, *J* = 7.9 Hz, 1H$^\alpha$), 6.52 (d, *J* = 16.2 Hz, 1H$^\alpha$), 6.28 (d, *J* = 15.9 Hz, 1H$^\beta$), 4.36 – 4.31 (m, 2H$^\alpha$), 4.28 – 4.24 (m, 2H$^\alpha$+4H$^\beta$), 4.22 – 4.18 (m, 2H$^{\alpha+\beta}$), 1.74 – 1.63 (m, 2H$^{\alpha+\beta}$), 1.48 – 1.39 (m, 2H$^{\alpha+\beta}$), 1.02 – 0.91 (m, 3H$^{\alpha+\beta}$) ppm. ^{13}C-NMR (126 MHz, $CDCl_3$): δ = 167.6 (C_q), 144.0 (C_q), 143.0 (C_q), 139.2 (CH), 123.8 (C_q), 121.2 (CH), 121.0 (CH), 119.5 (CH), 119.1 (CH), 64.6 (CH_2), 64.4 (CH_2), 64.1 (CH_2), 30.9 (CH_2), 19.3 (CH_2), 13.9 (CH_3) ppm. IR (ATR): 2958, 2929, 2874,

1706, 1632, 1454, 1310, 1275, 1199, 731 cm^{-1}. HRMS (ESI): *m/z* calced for [$C_{15}H_{18}O_4$+H^+], 263.1278 found 263.1276.

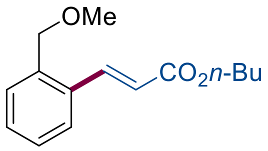

(*E*)-*n*-butyl-3-(2-(methoxymethyl)phenyl)acrylate (*o*-ML4) & (*E*)-*n*-butyl-3-(3-(methoxymethyl)phenyl)acrylate (*m*- ML4) & (*E*)-*n*-butyl-3-(4-(methoxymethyl)phenyl)acrylate (*p*- ML4)

The general procedure **A** was followed using (methoxymethyl)benzene (**2m**) (126.8 µL, 1.0 mmol, 5.0 equiv) and *n*-butyl acrylate (**3a**) (28.8 µL, 0.20 mmol, 1.0 equiv) at 80 °C for 20 h. **L12** was used as ligand. Isolation by column chromatography (*n*-hexane/EtOAc = 20:1) yielded **ML4** (8.5 mg, 17%) as a mixture. The site selectivity was determined by ^{1}H-NMR and NOESY NMR, *o : m : p* = 2 : 1.7 : 1. ^{1}H-NMR (600 MHz, $CDCl_3$): δ = 7.99 (d, *J* = 15.9 Hz, $1H^o$), 7.68 (d, *J* = 16.0 Hz, $1H^m$), 7.67 (d, *J* = 16.0 Hz, $1H^p$), 7.61 (dd, *J* = 7.6, 1.5 Hz, $1H^o$), 7.53 – 7.50 (m, $1H^m$+$2H^p$), 7.49 – 7.30 (m, $3H^o$+$3H^m$+$2H^p$), 6.46 (d, *J* = 15.9 Hz, $1H^m$), 6.44 (d, *J* = 16.1 Hz, $1H^p$), 6.39 (d, *J* = 15.9 Hz, $1H^o$), 4.57 (s, $2H^o$), 4.47 (s, $2H^{m+p}$), 4.26 – 4.17 (m, $2H^{o+m+p}$), 3.44 (s, $3H^o$), 3.41 (s, $3H^m$), 3.40 (s, $3H^p$), 1.74 – 1.65 (m, $2H^{o+m+p}$), 1.48 – 1.39 (m, $2H^{o+m+p}$), 1.01 – 0.93 (m, $3H^{o+m+p}$). ^{13}C-NMR (126 MHz, $CDCl_3$): δ = 167.1 (C_q), 167.1 (C_q), 167.0 (C_q), 144.4 (CH), 144.2 (CH), 141.5 (CH), 140.6 (C_q), 139.0 (C_q), 137.0 (C_q), 134.7 (C_q), 133.8 (C_q), 133.7 (C_q), 129.9 (CH), 129.5 (CH), 129.4 (CH), 128.9 (CH), 128.3 (CH), 128.1 (CH), 128.0 (CH), 127.4 (CH), 127.1 (CH), 126.7 (CH), 120.1 (CH), 118.5 (CH), 118.2 (CH), 74.3 (CH_2), 74.52 (CH_2), 72.5 (CH_2), 64.4 (CH_2), 58.4 (CH_3), 58.3 (CH_3), 30.8 (CH_2), 19.2 (CH_2), 13.7 (CH_3). IR (ATR): 2960, 2934, 2873, 1712, 1637, 1312, 1275, 1172, 1105, 982 cm^{-1}. MS (ESI) *m*/*z* (relative intensity): 271 (100) [M + Na]$^+$. HR-MS (ESI): *m*/*z* calcd. for [$C_{15}H_{20}O_3$ + Na]$^+$ 271.1305 found 271.1305.

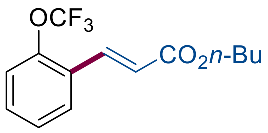

(*E*)-*n*-butyl-3-(2-(trifluoromethoxy)phenyl)acrylate (*o*-ML5) & (*E*)-*n*-butyl-3-(3-(trifluoromethoxy)phenyl)acrylate (*m*- ML5) & (*E*)-*n*-butyl-3-(4-(trifluoromethoxy)phenyl)acrylate (*p*- ML5)

The general procedure **A** was followed using (trifluoromethoxy)benzene (**2n**) (264.4 µL, 2.0 mmol, 10 equiv) and *n*-butyl acrylate (**3a**) (28.8 µL, 0.20 mmol, 1.0 equiv) at 80 °C for 20 h. **L12** was used as ligand. Isolation by column chromatography (*n*-hexane/EtOAc = 20:1) yielded **ML5** (6.0 mg, 10%) as a mixture, including an unidentified impurity. The site selectivity was determined by ^{1}H-NMR and NOESY NMR, *o* : *m* : *p* = 1.5 : 1.0 : 1.3. ^{1}H-NMR (600 MHz, $CDCl_3$): δ = 7.92 (d, *J* = 16.1 Hz, $1H^{o}$), 7.69 – 7.18 (m, $4H^{o+m+p}$), 6.53 – 6.37 (m, $1H^{o+m+p}$), 4.29 – 4.12 (m, $2H^{o+m+p}$), 1.77 – 1.65 (m, $2H^{o+m+p}$), 1.49 – 1.42 (m, $2H^{o+m+p}$), 0.97 (td, *J* = 7.4, 1.3 Hz, $3H^{o+m+p}$). ^{13}C-NMR (126 MHz, CDCl3): δ = 166.8 (C_q), 166.6 (C_q), 166.5 (C_q), 150.3 (C_q, -OCF_3), 149.7 (C_q, -OCF_3), 147.5 (C_q, -OCF_3), 144.5 (CH), 142.8 (CH), 142.7 (CH), 137.2 (CH), 136.6 (C_q), 134.5 (C_q), 133.1 (C_q), 131.2 (CH), 130.3 (CH), 130.2 (CH), 129.4 (CH), 128.9 (CH), 128.0 (CH), 128.0 (CH), 127.1 (CH), 126.4 (CH), 122.4 (CH), 121.5 (C_q, CF_3), 121.4 (CH), 121.3 (CH), 121.1 (CH), 120.1 (CH), 120.1 (CH), 119.5 (C_q, CF_3), 119.3 (C_q, CF_3), 118.3 (CH), 64.7 (CH_2), 64.6 (CH_2), 64.6 (CH_2), 30.7 (CH_2), 30.7 (CH_2), 30.7 (CH_2), 19.2 (CH_2), 19.2 (CH_2), 13.7 (CH_3), 13.7 (CH_3). The *J* coupling values couldn't be identified due to the poor yield, and the number of CH is not accurate because of the impurity. ^{19}F-NMR (471 MHz, $CDCl_3$): δ = -57.33 (d, *J* = 1.6 Hz), -57.75, -57.81. IR (ATR): 2962, 2934, 2875, 1715, 1640, 1456, 1313, 1254, 1212, 982 cm^{-1}. MS (ESI) *m*/*z* (relative intensity): 311 (100) $[M + Na]^{+}$. HR-MS (ESI): *m*/*z* calcd. for $[C_{14}H_{15}F_3O_3 + Na]^{+}$ 311.0866 found 311.0872.

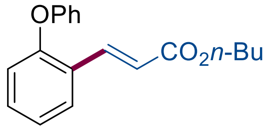

(*E*)-*n*-Butyl-3-(2-phenoxyphenyl)acrylate (*o*-ML6) & (*E*)-*n*-butyl-3-(4-phenoxyphenyl) acrylate (*p*-ML6)

The general procedure **A** was followed using ethoxybenzene (**2o**) (157.6 µL, 1.0 mmol, 5.0 equiv) and *n*-butyl acrylate (**2a**) (28.8 µL, 0.20 mmol, 1.0 equiv) at 60 °C for 24 h. **L12** was used as ligand. Isolation by column chromatography (*n*-hexane/EtOAc = 30:1 to 15:1) yielded

ML6 (38.6 mg, 65%) as a colorless oil. The ratio of the isomers was determined by the ^{1}H-NMR of the crude mixture, *o* : *p* = 4.0 : 1. ^{1}H-NMR (400 MHz, $CDCl_3$): δ = 8.02 (d, *J* = 16.2 Hz, $1H^o$), 7.70 – 7.61 (m, $1H^{o+p}$), 7.52 – 7.48 (m, $1H^p$), 7.40 – 7.27 (m, $3H^{o+p}$), 7.17 – 7.09 (m, $2H^{o+p}$), 7.08 – 6.96 (m, $2H^o$+$3H^p$), 6.88 (dd, *J* = 8.3, 1.1 Hz, $1H^o$), 6.56 (d, *J* = 16.2 Hz, $1H^o$), 6.36 (d, *J* = 16.0 Hz, $1H^p$), 4.30 – 4.11 (m, $2H^{o+p}$), 1.73 – 1.57 (m, $2H^{o+p}$), 1.48 – 1.31 (m, $2H^{o+p}$), 1.02 – 0.88 (m, $3H^{o+p}$). ^{13}C-NMR (100 MHz, $CDCl_3$): δ^o = 167.2 (C_q), 156.9 (C_q), 156.0 (C_q), 139.1 (CH), 131.3 (CH), 129.9 (CH), 128.6 (CH), 126.0 (C_q), 124.1 (CH), 123.6 (CH), 123.6 (CH), 119.7 (CH), 119.0 (CH), 64.4 (CH_2), 30.8 (CH_2), 19.2 (CH_2), 13.8 (CH_3). IR (ATR): 2960, 2873, 2255, 1706, 1634, 1589, 1486, 1234, 1173, 908 cm^{-1}. MS (ESI) *m*/*z* (relative intensity): 297 (40) $[M + H]^+$, 319 (100) $[M + Na]^+$. HR-MS (ESI): *m*/*z* calcd. for $[C_{19}H_{20}O_3 + Na]^+$ 319.1305 found 319.1305.

Mechanistic Investigation for High Site-selectivity

Control Experiment

Supplementary Table 17 Control experiment

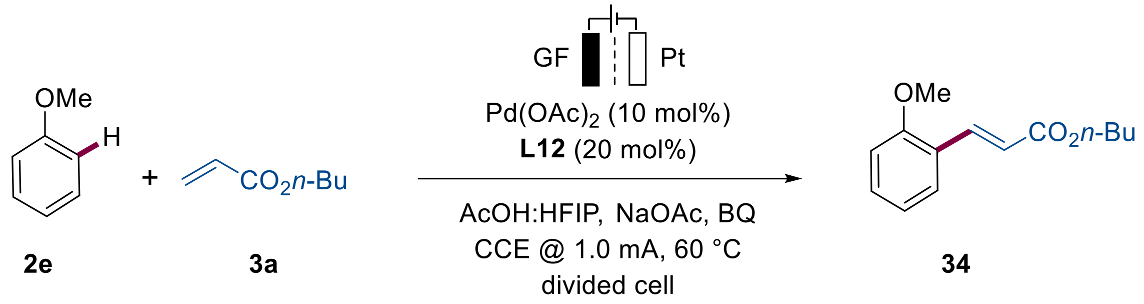

2e **3a** **34**

Entry	Deviations from standard condition	Yield [%]	*ortho*:*para*
1	None	52[a]	14:1
2	No electricity	37	2.6:1
3	Under N_2	54	2.6:1
4	No ligand	8	1:1
5	No BQ	56	11:1

Reaction conditions: divided cell, anodic chamber: **2e** (1.0 mmol), **3a** (0.20 mmol), [Pd] (10 mol %), **L12** (20 mol %), NaOAc (4.0 equiv), BQ (20 mol %), HFIP:AcOH (1.3 mL:2.6 mL); cathodic chamber: NaOAc (4.0 equiv), BQ (20 mol %), HFIP:AcOH (1.3 mL:2.6 mL), 60 °C, constant current at 1.0 mA, 20 h, graphite felt (GF) anode, Pt-plate cathode. Without special note, yields and isomer ratios were determined by crude ^{1}H-NMR by using CH_2Br_2 as internal standard. [a] Isolated yield.

Supplementary Table 18 Chemical oxidant

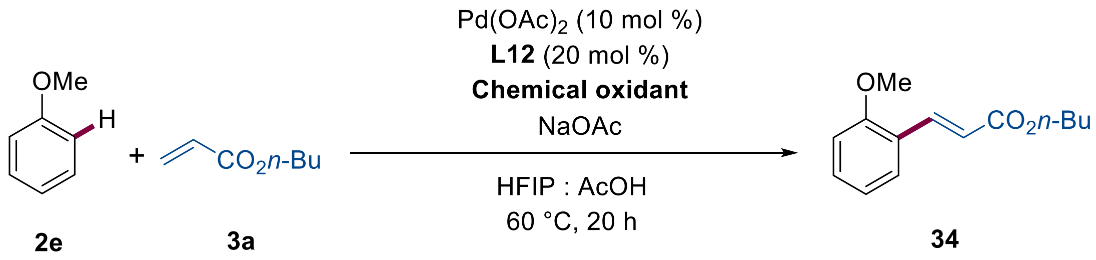

Entry	Oxidant	Amount	Yield [%]	*ortho*:*para*
1	*t*-$BuCO_3Ph$	1.0 equiv	59	2.5:1
2	BQ	1.0 equiv	47	2.1:1
3	AgOAc	2.0 equiv	74	2.7:1
4	O_2 (1 atm.)	---	30	2.3:1
5	$K_2S_2O_8$	1.0 equiv	22	2.7:1
6	PIDA	1.0 equiv	24	3:1

An oven-dried 10 mL Schlenk tube was charged with $Pd(OAc)_2$ (4.5 mg, 0.02 mmol, 10 mol %), **L12** (7.9 mg, 0.04 mmol, 20 mol %), NaOAc (66.0 mg, 0.8 mmol, 4.0 equiv), anisole **2e** (109.0 μL, 1 mmol, 5.0 equiv), *n*-butyl acrylate **3a** (28.8 μL, 0.2 mmol, 1.0 equiv), the corresponding oxidant, HFIP:AcOH (1.3 mL:2.6 mL). The tube was sealed with a septum and the reaction was placed in a 60 °C oil bath for 20 h. The mixture was filtered through a short pad of silica column and eluent with EtOAc (50 mL), the solvent was removed under reduced pressure. Yields and isomer ratios were determined by crude ^{1}H-NMR by using CH_2Br_2 as internal standard. PIDA = (Diacetoxyiodo)benzene.

Supplementary Table 19 ***Ortho* and *para* product with chemical oxidant**

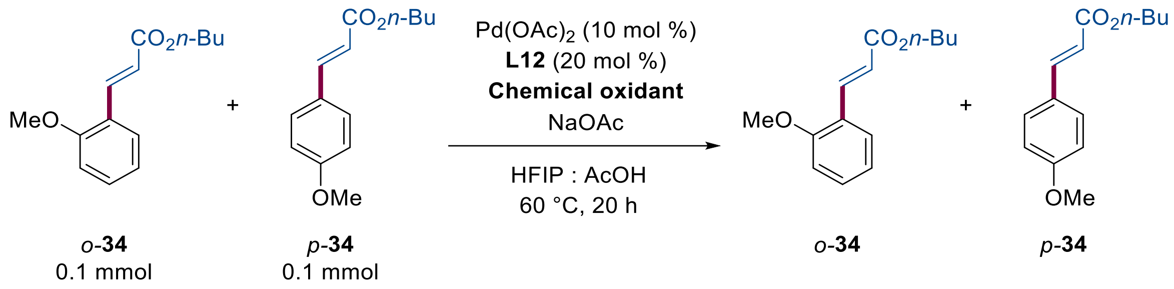

Entry	Oxidant	Amount	*o*-**34**	*p*-**34**
1	air	1 atm.	80%	84%
2	BQ	0.4 mmol	96%	96%
3	AgOAc	0.4 mmol	92%	96%
4	H_2O_2	0.4 mmol	12%	4%
5	$K_2S_2O_8$	0.4 mmol	99%	99%
6	PIDA	0.4 mmol	90%	62%
7	oxone	0.4 mmol	0%	0%
8	TBHP	0.4 mmol	66%	42%
9	PIFA	0.4 mmol	0%	0%
10	PIFA	0.2 mmol	77%	51%

An oven-dried 10 mL Schlenk tube was charged with $Pd(OAc)_2$ (4.5 mg, 0.02 mmol, 10 mol %), **L12** (7.9 mg, 0.04 mmol, 20 mol %), NaOAc (66.0 mg, 0.8 mmol, 4.0 equiv), *o*-**34** (20.6 mg, 0.1 mmol), *p*-**34** (20.6 mg, 0.1 mmol), the corresponding oxidant, HFIP:AcOH (1.3 mL:2.6 mL). The tube was sealed with a septum and the reaction was placed in a 60 °C oil bath for 20 h. The mixture was filtered through a short pad of silica column and eluent with EtOAc (50 mL), the solvent was removed under reduced pressure. Yields and isomer ratios were determined by crude ^{1}H-NMR by using CH_2Br_2 as internal standard. PIDA = Diacetoxyiodo)benzene. TBHP = *tert*-Butylhydroperoxid. PIFA = (Bis(trifluoroacetoxy)iodine)benzene.

Supplementary Table 20 Effects of anode material

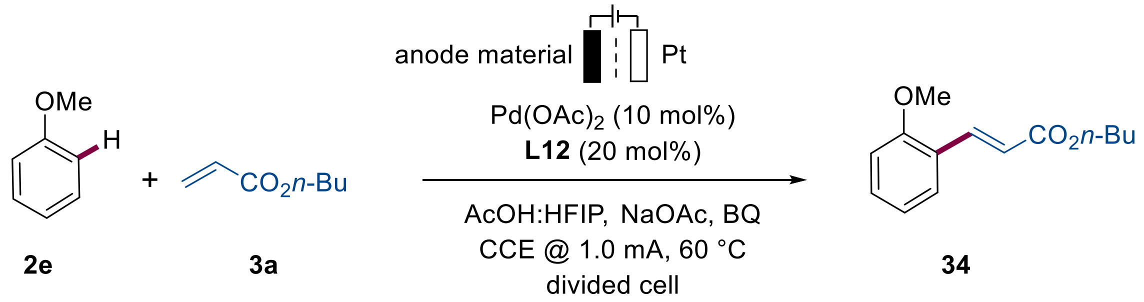

Entry	Anode material	Yield [%]	*ortho*:*para*
1	Carbon felt	52[a]	14:1
2	Glass carbon	66	7:1
3	BDD	49	16:1
4	RVC	49	14:1
5	Graphite rod	37	17:1
6	Pt	75	2.6:1
7	Ni foam	25	2.1:1
8	Stainless steel	5	2.0:1
9	Al	29	2.3:1
10	Cu	7	2.8:1
11	Nb	53	3.1:1
12	Ag	86	2.0:1

Reaction conditions: divided cell, anodic chamber: **2e** (1.0 mmol), **3a** (0.20 mmol), [Pd] (10 mol %), **L12** (20 mol %), NaOAc (4.0 equiv), BQ (20 mol %), HFIP:AcOH (1.3 mL:2.6 mL); cathodic chamber: NaOAc (4.0 equiv), BQ (20 mol %), HFIP:AcOH (1.3 mL:2.6 mL), 60 °C, constant current at 1.0 mA, 20 h, Pt-plate cathode. Without special note, yields and isomer ratios were determined by crude ^{1}H-NMR by using CH_2Br_2 as internal standard. [a] Isolated yield. SS = Stainless steel. GC = Glass carbon. RVC = Reticulated vitreous carbon. GF = Graphite felt. BDD = Boron doped diamond. GR = Graphite rod.

Supplementary Table 21 Variation of Selectivity and yield over time

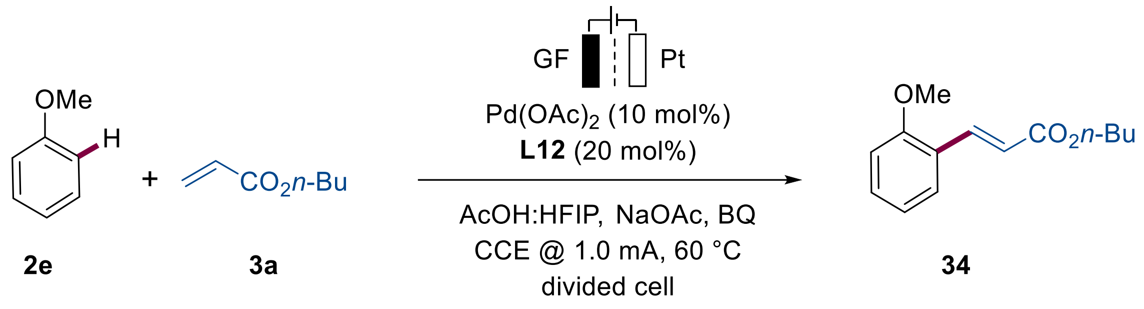

Entry	Time (h)	Yield [%]	*ortho*-Isomer [%]	*para*-Isomer [%]	*ortho*:*para*
1	2	33	23	10	2.3:1
2	4	50	36	14	2.5:1
3	6	59	42	17	2.4:1
4	8	66	46	20	2.1:1
5	10	74	54	20	2.7:1
6	12	87	62	25	2.5:1
7	14	74	59	15	4:1
8	16	68	68	8	7.5:1
9	20	66	62	4	14:1

Reaction conditions: divided cell, anodic chamber: **2e** (1.0 mmol), **3a** (0.20 mmol), [Pd] (10 mol %), **L12** (20 mol %), NaOAc (4.0 equiv), BQ (20 mol %), HFIP:AcOH (1.3 mL:2.6 mL); cathodic chamber: NaOAc (4.0 equiv), BQ (20 mol %), HFIP:AcOH (1.3 mL:2.6 mL), 60 °C, constant current at 1.0 mA, graphite felt (GF) anode, Pt-plate cathode. Yields and isomer ratios were determined by crude ^{1}H-NMR by using CH_2Br_2 as internal standard.

Two-fold Electrochemical Oxidation

The experiment was carried out in a pre-dried divided cell, with a GF anode (10 mm × 15 mm × 6 mm) and a platinum cathode (10 mm × 15 mm × 0.25 mm). (*E*)-Ethyl 3-(4-methoxyphenyl) acrylate **34** or **40** (0.20 mmol, 1.0 equiv), $Pd(OAc)_2$ (4.5 mg, 10 mol %), **L12** (20 mol %), 1,4-benzoquinone (4.3 mg, 20 mol %) and NaOAc (66.0 mg, 4.0 equiv) were

placed in the anodic chamber and dissolved in AcOH (2.6 mL) and HFIP (1.3 mL); 1,4-benzoquinone (4.3 mg, 20 mol %) and NaOAc (66.0 mg, 4.0 equiv) were placed in the cathodic chamber and dissolved in AcOH (2.6 mL) and HFIP (1.3 mL). Electrocatalysis was performed at 60 °C at a constant current of 1.0 mA and a stirring rate of 500 rpm was maintained for 20 h. At ambient temperature, the GF anode was washed with EtOAc (3 × 10 mL) in an ultrasonic bath and the washings were added to the reaction mixture. The solvents were removed *in vacuo*. The residue was added in the sat. $NaHCO_3$ (30 mL), extracted with CH_2Cl_2 (3 x 20 mL). The combined organic layers were dried over Na_2SO_4, filtered and concentrated under reduced pressure. Then the crude mixture was dissolved with 4 mL DCM, then added with Et_3N (70 µL, 0.5 mmol, 2.5 equiv), acetyl chloride (18 µL, 0.25 mmol, 1.25 equiv) and stirred at RT for 1 hour. The solvents were removed *in vacuo*. The crude mixture was purified by flash column chromatography (*n*-hexane/EtOAc = 4:1) on silica gel to yield the products.

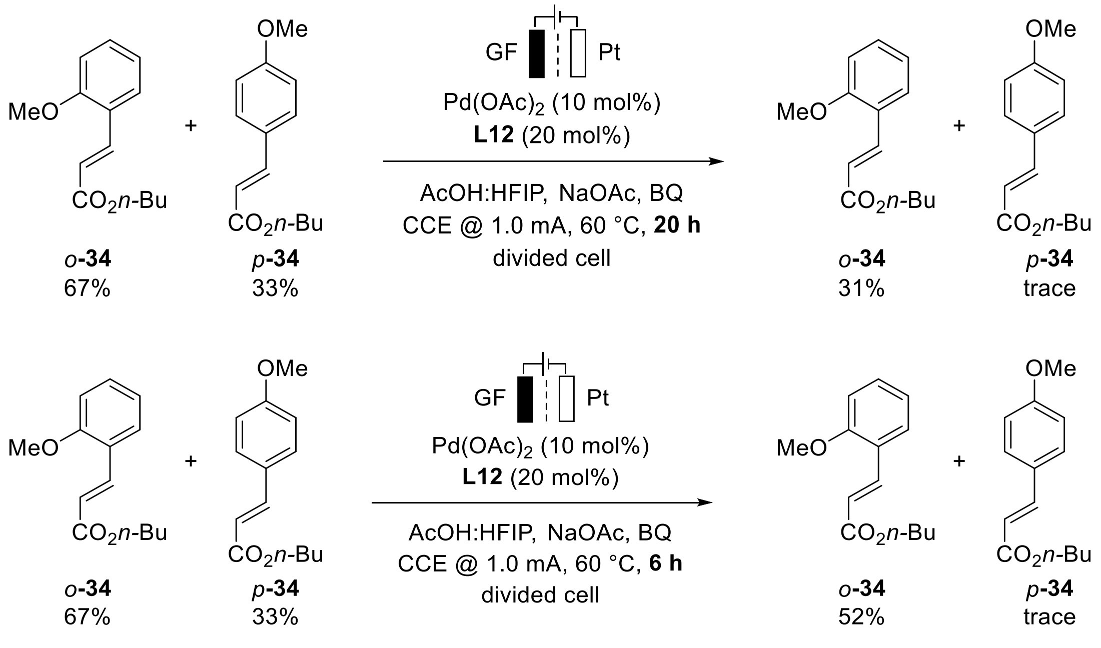

Supplementary Figure 22 Selective oxidation of *p*-isomer

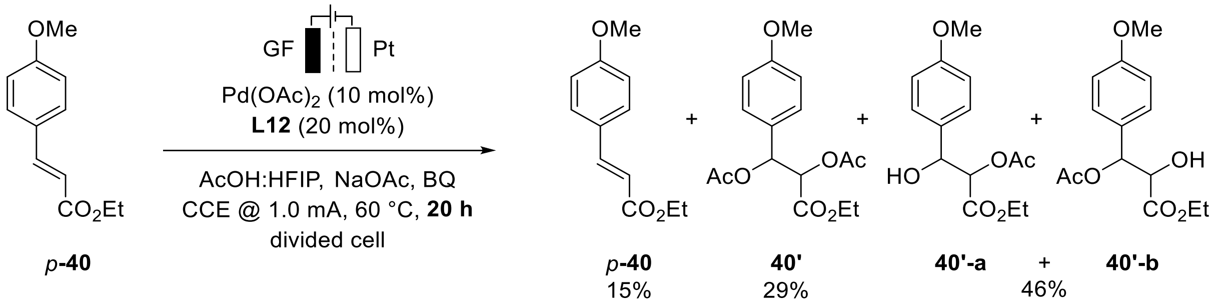

Supplementary Figure 23 Second fold oxidation

Supplementary Table 22 Controlled experiment for second oxidation

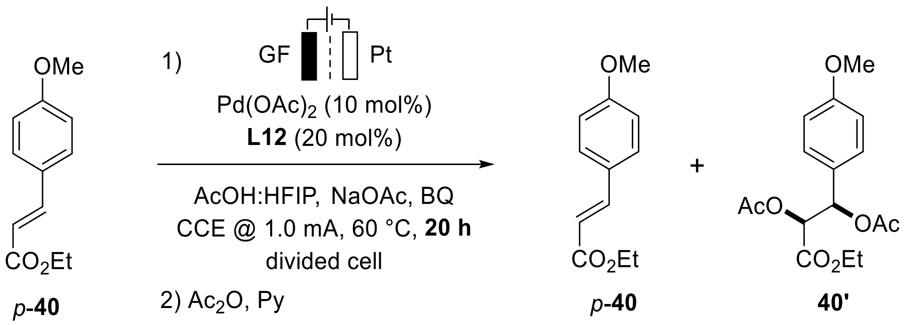

Entry	Deviation from the standard conditions	*p*-**34**	**40’**
1	none	8%	67%
2	Pt as anode	24%	65%
3	No electricity	97%	---
4	No $Pd(OAc)_2$	16%	57%

Experiment for Switching Selectivity

Supplementary Table 23 Effects of Lewis acid

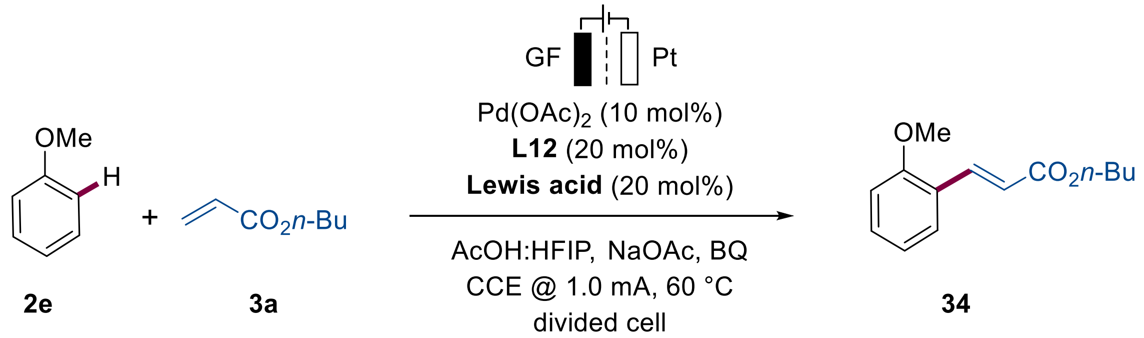

Entry	Lewis acid	Yield [%]	*ortho*:*para*
1	$Cu(OTf)_2$	56	7:1
2	$AlCl_3$	10	10:1
3	$ZnCl_2$	7	16:1
4	$FeCl_3$	10	20:1
5	$TiCl_4$	4	3:1
6	$ZrCl_4$	5	20:1

Reaction conditions: divided cell, anodic chamber: **2e** (1.0 mmol), **3a** (0.20 mmol), [Pd] (10 mol %), **L12** (20 mol %), NaOAc (4.0 equiv.), BQ (20 mol %), HFIP:AcOH (1.3 mL:2.6 mL); cathodic chamber: NaOAc (4.0 equiv.), BQ (20 mol %), Lewis acid (20 mol%), HFIP:AcOH (1.3 mL:2.6 mL), 60 °C, constant current at 1.0 mA, 20 h, graphite felt (GF) anode, Pt-plate cathode. Without special note, yields and isomer ratios were determined by crude ^{1}H-NMR by using CH_2Br_2 as internal standard.

Supplementary Table 24 Effects of dual catalyst

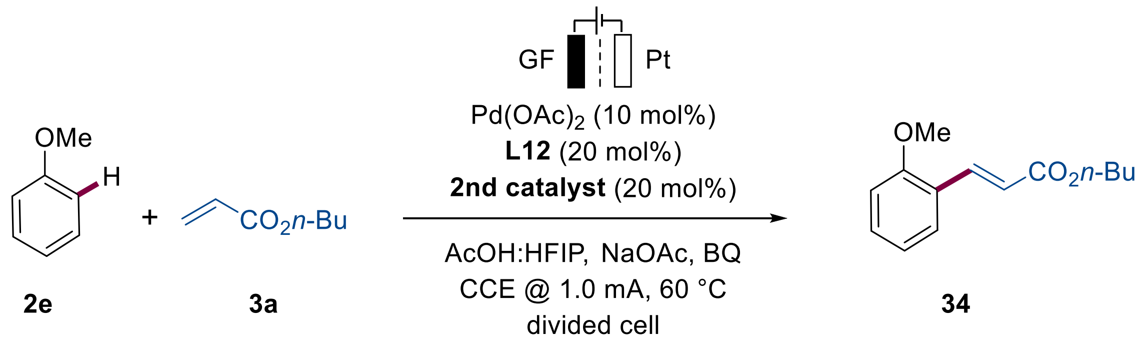

Entry	2nd catalyst (5 mol%)	Yield [%]	*ortho*:*para*
1	[Ru(*p*-cymene)Cl_2]$_2$	23%	2.4 : 1
2	[Cp*RhCl_2]$_2$	44%	14 : 1
3	[Os(*p*-cymene)Cl_2]$_2$	n.r.	--
4	Cp*Co(CO)I_2	6%	3.4 : 1

Reaction conditions: divided cell, anodic chamber: **2e** (1.0 mmol), **3a** (0.20 mmol), [Pd] (10 mol %), **L12** (20 mol %), NaOAc (4.0 equiv.), BQ (20 mol %), HFIP:AcOH (1.3 mL:2.6 mL); cathodic chamber: NaOAc (4.0 equiv.), BQ (20 mol %), catalyst (5 mol%), HFIP:AcOH (1.3 mL:2.6 mL), 60 °C, constant current at 1.0 mA, 20 h, graphite felt (GF) anode, Pt-plate cathode. Without special note, yields and isomer ratios were determined by crude ^{1}H-NMR by using CH_2Br_2 as internal standard.

Selective Oxidation of Other Arene

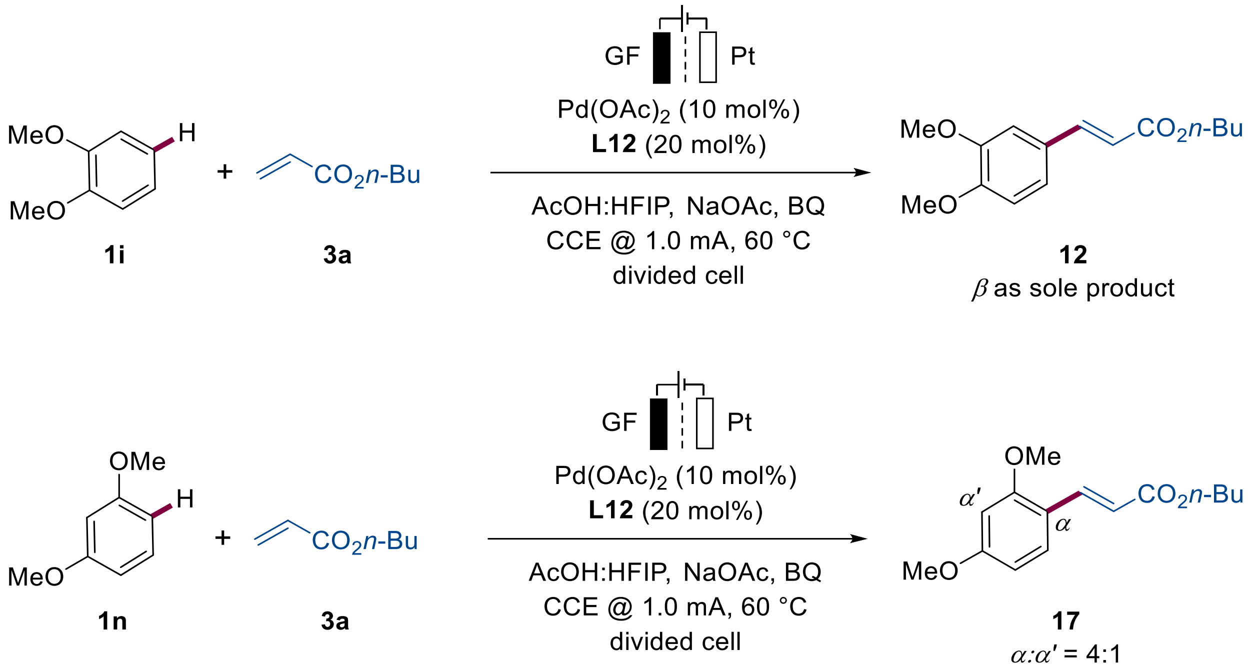

Supplementary Figure 24 Selective oxidation of dimethoxybenzene.

Reaction conditions: divided cell, anodic chamber: **1i** or **1n** (1.0 mmol), **3a** (0.20 mmol), [Pd] (10 mol %), **L12** (20 mol %), NaOAc (4.0 equiv), BQ (20 mol %), HFIP:AcOH (1.3 mL:2.6 mL); cathodic chamber: NaOAc (4.0 equiv), BQ (20 mol %), HFIP:AcOH (1.3 mL:2.6 mL), 60 °C, constant current at 1.0 mA, graphite felt (GF) anode, Pt-plate cathode. Yields and isomer ratios were determined by crude ^{1}H-NMR by using CH_2Br_2 as internal standard. When compared these results with the scale-up results, some isomers are missing, indicating that selective oxidation happened to **12** and **17** as well

Supplementary Table 25 Overoxidation for phenylpiperidine

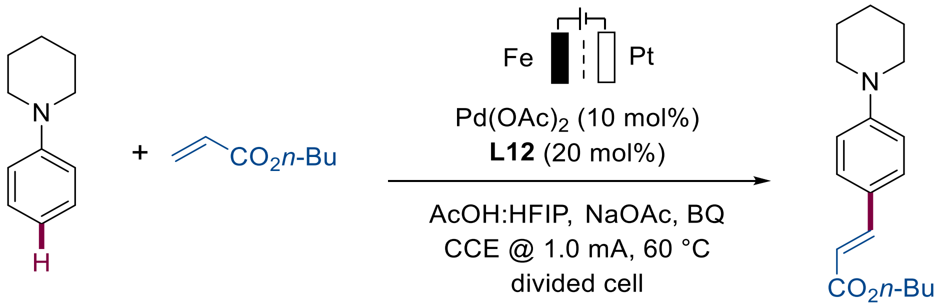

2p **3a** **S1**

Entry	Time (h)	Yield [%]	*o* : *p*
1	6	12%	1 : 6.3
2	12	12%	1 : 6.2
3	16	19%	1 : 6.1
4	20	18%	1 : 5.0
5	24	26%	1 : 4.2
6	6 (Pt as anode)	13%	1 : 5.5
7	20 (Pt as anode)	21%	1 : 2

Reaction conditions: divided cell, anodic chamber: phenylpiperidine (**2p**) (1.0 mmol), **3a** (0.20 mmol), [Pd] (10 mol %), **L12** (20 mol %), NaOAc (4.0 equiv), BQ (20 mol %), HFIP:AcOH (1.3 mL:2.6 mL); cathodic chamber: NaOAc (4.0 equiv), BQ (20 mol %), HFIP:AcOH (1.3 mL:2.6 mL), 60 °C, constant current at 1.0 mA, stainless steel (Fe) anode, Pt-plate cathode. Yields and isomer ratios were determined by crude ^{1}H-NMR by using CH_2Br_2 as internal standard.

Supplementary Table 26 Overoxidation for naphthalene

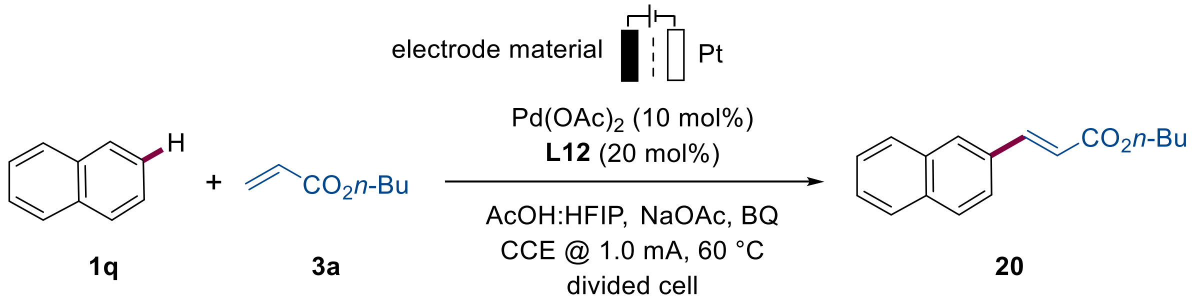

Entry	Anode material	Yield [%]	*α:β*
1	Carbon felt	73	2.8: 1
3	Nb	74	2.1: 1
4	Stainless steel	78	2.8: 1
5	Pt	70	1.9: 1

Reaction conditions: divided cell, anodic chamber: **1q** (1.0 mmol), **3a** (0.20 mmol), [Pd] (10 mol %), **L12** (20 mol %), NaOAc (4.0 equiv), BQ (20 mol %), HFIP:AcOH (1.3 mL:2.6 mL); cathodic chamber: NaOAc (4.0 equiv), BQ (20 mol %), HFIP:AcOH (1.3 mL:2.6 mL), 60 °C, constant current at 1.0 mA for 20 h, graphite felt (GF) anode, Pt-plate cathode. Yields and isomer ratios were determined by crude ^{1}H-NMR by using CH_2Br_2 as internal standard.

Characterization Data of Products

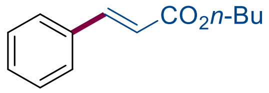

***n*-Butyl cinnamate (4)**

The general procedure **A** was followed using benzene (**1a**) (356.4 µL, 4.0 mmol, 20 equiv) and *n*-butyl acrylate (**3a**) (28.8 µL, 0.20 mmol, 1.0 equiv) at 80 °C for 20 h. **L12** was used as ligand. Isolation by column chromatography (*n*-hexane/EtOAc = 20:1) yielded **4** (33.1 mg, 81%) as a colorless oil. The product is known compound.[29] ^{1}H-NMR (600 MHz, $CDCl_3$): δ = 7.68 (d, J = 16.0 Hz, 1H), 7.55 – 7.51 (m, 2H), 7.40 – 7.37 (m, 3H), 6.44 (d, J = 16.0 Hz, 1H), 4.21 (t, J = 6.7 Hz, 2H), 1.73 – 1.67 (m, 2H), 1.49 – 1.40 (m, 2H), 0.97 (t, J = 7.4 Hz, 3H). ^{13}C-NMR (150 MHz, $CDCl_3$): δ = 167.1 (C_q), 144.5 (CH), 134.5 (C_q), 130.2 (CH), 128.8 (CH), 128.0 (CH), 118.3 (CH), 64.4 (CH_2), 30.8 (CH_2), 19.2 (CH_2), 13.7 (CH_3). IR (ATR): 3059, 2960, 2931, 2873, 2251, 1708, 1638, 1265, 909, 737 cm^{-1}. MS (ESI) m/z (relative intensity): 227 (100) $[M + Na]^+$, 205 (10) $[M + H]^+$. HR-MS (ESI): m/z calcd. for $[C_{13}H_{16}O_2 + Na]^+$ 227.1043 found 227.1043.

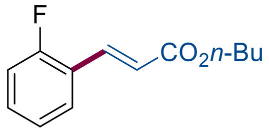

(*E*)-*n*-butyl-3-(2-fluorophenyl)acrylate (*o*-5) & (*E*)-*n*-butyl-3-(3-fluorophenyl)acrylate (*m*-5) & (*E*)-*n*-butyl-3-(4-fluorophenyl)acrylate (*p*-5)

The general procedure **A** was followed using fluorobenzene (**1b**) (187.5 µL, 2.0 mmol, 10 equiv) and *n*-butyl acrylate (**3a**) (28.8 µL, 0.20 mmol, 1.0 equiv) at 80 °C for 20 h. **L12** was used as ligand. Isolation by column chromatography (*n*-hexane/EtOAc = 50:1 to 20:1) yielded **5** (16.8 mg, 38%) as a colorless oil. The *o*, *m* and *p*-olefinated products are known compounds[30] and the ratio of the isomers was determined by the ^{1}H-NMR of the crude mixture, $o : m : p$ = 15 : 1 : 6. ^{1}H-NMR (400 MHz, $CDCl_3$): δ = 7.81 (d, J = 16.2 Hz, 1H^o), 7.64 (d, J = 16.0 Hz, 1H^p), 7.63 (d, J = 16.0 Hz, 1H^m), 7.57 – 7.49 (m, 1H^o+2H^p), 7.39 – 7.31 (m, 1H^o), 7.16 (td, J = 7.6, 1.2 Hz, 1H^o), 7.13 – 7.05 (m, 1H^o+2H^p), 6.54 (d, J = 16.2 Hz, 1H^o), 6.43 (d, J = 16.0 Hz, 1H^m), 6.36 (d, J = 16.0 Hz, 1H^p), 4.27 – 4.17 (m, 2H^{o+m+p}), 1.74 – 1.64 (m, 2H^{o+m+p}), 1.50 – 1.38 (m, 2H^{o+m+p}), 1.02 – 0.92 (m, 3H^{o+m+p}). ^{13}C-NMR (100 MHz, $CDCl_3$): δ = 167.0 (C_q),

166.9 (C_q), 163.8 (d, *J* = 252.3 Hz, C_q), 161.3 (d, *J* = 253.9 Hz, C_q), 143.2 (CH), 137.2 (d, *J* = 2.9 Hz, CH), 131.6 (d, *J* = 8.8 Hz, CH), 129.9 (d, *J* = 8.6 Hz, CH), 129.1 (d, *J* = 3.1 Hz, CH), 124.4 (d, *J* = 3.6 Hz, CH), 122.6 (d, *J* = 11.6 Hz, C_q), 120.9 (d, *J* = 6.5 Hz, CH), 118.1 (d, *J* = 2.4 Hz, CH), 116.2 (d, *J* = 22.0 Hz, CH), 116.0 (d, *J* = 22.0 Hz, CH), 64.6 (CH_2), 64.5 (CH_2), 30.8 (CH_2), 30.8 (CH_2), 19.2 (CH_2), 13.8 (CH_3). ^{19}F-NMR (377 MHz, $CDCl_3$): δ = -109.77 (tt, *J* = 8.6, 5.4 Hz), -112.57 (td, *J* = 9.1, 5.7 Hz), -114.37 (ddd, *J* = 10.6, 7.4, 5.2 Hz). IR (ATR): 2960, 2935, 2874, 1710, 1638, 1487, 1316, 1276, 1168, 981 cm^{-1}. MS (ESI) *m*/*z* (relative intensity): 223 (100) $[M + Na]^+$, 245 (60) $[M + H]^+$. HR-MS (ESI): *m*/*z* calcd. for $[C_{13}H_{15}O_2F + Na]^+$ 245.0948 found 245.0942.

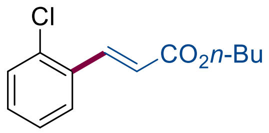

(*E*)-*n*-Butyl-3-(2-chlorophenyl)acrylate (*o*-6) & (*E*)-*n*-butyl-3-(3-chlorophenyl)acrylate (*m*-6) & (*E*)-*n*-butyl-3-(4-chlorophenyl)acrylate (*p*-6)

The general procedure **A** was followed using chlorobenzene (**1c**) (202.8 µL, 2.0 mmol, 10 equiv) and *n*-butyl acrylate (**3a**) (28.8 µL, 0.20 mmol, 1.0 equiv) at 80 °C for 20 h. **L12** was used as ligand. Isolation by column chromatography (*n*-hexane/EtOAc = 50:1 to 20:1) yielded **6** (17.2 mg, 36%) as a colorless oil. The *o*, *m* and *p*-olefinated products are known compounds[31] and the ratio of the isomers was determined by the ^{1}H-NMR of the crude mixture, *o* : *m* : *p* = 4 : 1 : 1. ^{1}H-NMR (600 MHz, $CDCl_3$): δ = 8.09 (d, *J* = 16.0 Hz, $1H^{o}$), 7.64 – 7.26 (m, $4H^{o+m+p}$), 6.46 – 6.39 (m, $1H^{o+m+p}$), 4.32 – 4.14 (m, $2H^{o+m+p}$), 1.74 – 1.65 (m, $2H^{o+m+p}$), 1.48 – 1.39 (m, $2H^{o+m+p}$), 1.01 – 0.93 (m, $3H^{o+m+p}$). ^{13}C-NMR (150 MHz, $CDCl_3$): δ = 166.8 (C_q), 166.6 (C_q), 166.5 (C_q), 143.0 (CH), 142.9 (CH), 140.3 (CH), 136.3 (C_q), 136.1 (C_q), 134.9 (C_q), 134.9 (C_q), 132.9 (C_q), 132.7 (C_q), 130.9 (CH), 130.1 (CH), 130.1 (CH), 130.0 (CH), 129.2 (CH), 129.1 (CH), 128.8 (CH), 128.0 (CH), 127.7 (CH), 127.6 (CH), 127.0 (CH), 126.2 (CH), 120.9 (CH), 119.8 (CH), 118.9 (CH), 64.6 (CH_2), 64.5 (CH_2), 30.7 (CH_2), 30.7 (CH_2), 19.2 (CH_2), 19.2 (CH_2), 13.7 (CH_3), 13.7 (CH_3). IR (ATR): 3053, 2962, 2874, 1708, 1636, 1313, 1265, 1173, 907, 731 cm^{-1}. MS (ESI) *m*/*z* (relative intensity): 261 (100) $[M + Na]^+$, 239 (20) $[M + H]^+$. HR-MS (ESI): *m*/*z* calcd. for $[C_{13}H_{15}O_2Cl + Na]^+$ 261.0653 found 261.0650.

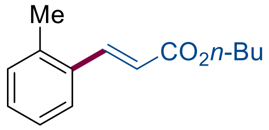

(*E*)-*n*-Butyl-3-(*o*-tolyl)acrylate (*o*-7) & (*E*)-*n*-butyl-3-(*m*-tolyl)acrylate (*m*-7) & (*E*)-*n*-butyl-3-(*p*-tolyl)acrylate (*p*-7)

The general procedure **C** was followed using toluene (**1d**) (158.8 µL, 1.5 mmol, 3.0 equiv) and *n*-butyl acrylate (**3a**) (72.0 µL, 0.50 mmol, 1.0 equiv) at 60 °C for 48 h. **L12** was used as ligand. Isolation by column chromatography (*n*-hexane/EtOAc = 50:1 to 20:1) yielded **7** (66.4 mg, 61%) as a colorless oil. The *o, m,* and *p*-olefinated products are known compounds[31] and the ratio of the isomers was determined by the ^{1}H-NMR of the crude mixture, *o* : *m* : *p* = 2 : 1 : 1. ^{1}H-NMR (400 MHz, $CDCl_3$): δ = 7.98 (d, J = 15.9 Hz, 1H^{o}), 7.66 (d, J = 16.0 Hz, 1H^{m+p}), 7.59 – 7.15 (m, 4H^{o+m+p}), 6.51 – 6.28 (m, 1H^{o+m+p}), 4.31 – 4.17 (m, 2H^{o+m+p}), 2.44 (s, 3H^{o}), 2.37 (s, 3H^{m+p}), 1.79 – 1.61 (m, 2H^{o+m+p}), 1.52 – 1.37 (m, 2H^{o+m+p}), 1.04 – 0.92 (m, 3H^{o+m+p}). ^{13}C-NMR (100 MHz, $CDCl_3$): δ = 167.3 (C_q), 167.1 (C_q), 144.7 (CH), 144.5 (CH), 142.2 (CH), 140.6 (C_q), 138.5 (C_q), 137.6 (C_q), 134.4 (C_q), 133.4 (C_q), 131.7 (C_q), 131.0 (CH), 130.7 (CH), 129.9 (CH), 129.6 (CH), 128.7 (CH), 128.7 (CH), 128.0 (CH), 126.4 (CH), 126.3 (CH), 125.2 (CH), 119.3 (CH), 118.0 (CH), 117.2 (CH), 64.4 (CH_2), 64.4 (CH_2), 64.3 (CH_2), 30.8 (CH_2), 30.8 (CH_2), 21.4 (CH_3), 21.3 (CH_3), 19.8 (CH_3), 19.2 (CH_2), 13.7 (CH_3). IR (ATR): 3055, 2958, 2931, 2872, 1711, 1636, 1311, 1273, 1170, 982 cm^{-1}. MS (ESI) *m*/*z* (relative intensity): 241 (100) $[M + Na]^+$, 219 (10) $[M + H]^+$. HR-MS (ESI): *m*/*z* calcd. for $[C_{14}H_{18}O_2 + Na]^+$ 241.1199 found 241.1201.

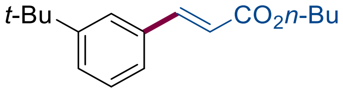

(*E*)-*n*-Butyl-3-(3-(*tert*-butyl)phenyl)acrylate (*m*-8) & (*E*)-*n*-butyl-3-(4-(*tert*-butyl)phenyl)acrylate (*p*-8)

The general procedure **C** was followed using *tert*-butylbenzene (**1e**) (116.1 µL, 0.75 mmol, 1.5 equiv) and *n*-butyl acrylate (**3a**) (72.0 µL, 0.50 mmol, 1.0 equiv) at 60 °C for 48 h. **L12** was used as ligand. Isolation by column chromatography (*n*-hexane/EtOAc = 50:1 to 20:1) yielded **8** (48.2 mg, 37%) as a colorless oil and 13% difunctionalized product. The *p*-olefinated product is known compound[32] and the ratio of the isomers was determined by the ^{1}H-NMR of the crude

mixture, $o : m : p$ = 0 : 1 : 1. ^{1}H-NMR (600 MHz, $CDCl_3$): δ = 7.70 (d, J = 16.0 Hz, 1H^m), 7.67 (d, J = 16.0 Hz, 1H^p), 7.55 – 7.30 (m, 4H^{m+p}), 6.45 (d, J = 16.0 Hz, 1H^m), 6.41 (d, J = 16.0 Hz, 1H^p), 4.25 – 4.17 (m, 2H^{m+p}), 1.73 – 1.66 (m, 2H^{m+p}), 1.50 – 1.40 (m, 2H^{m+p}), 1.37 – 1.29 (m, 9H^{m+p}), 1.00 – 0.94 (m, 3H^{m+p}). ^{13}C-NMR (150 MHz, $CDCl_3$): δ = 167.3 (C_q), 167.2 (C_q), 153.7 (C_q), 151.8 (C_q), 145.1 (CH), 144.4 (CH), 134.1 (C_q), 131.7 (C_q), 128.6 (CH), 127.9 (CH), 127.4 (CH), 125.8 (CH), 125.2 (CH), 125.1 (CH), 117.8 (CH), 117.3 (CH), 64.4 (CH_2), 64.3 (CH_2), 34.8 (C_q), 34.7 (C_q), 31.2 (CH_3), 31.1 (CH_3), 30.8 (CH_2), 19.2 (CH_2), 13.7 (CH_3). IR (ATR): 3061, 2959, 2871, 1711, 1636, 1308, 1166, 982, 828, 694 cm^{-1}. MS (ESI) m/z (relative intensity): 283 (30) $[M + Na]^+$, 261 (100) $[M + H]^+$. HR-MS (ESI): m/z calcd. for $[C_{17}H_{24}O_2 + H]^+$ 261.1849 found 261.1849.

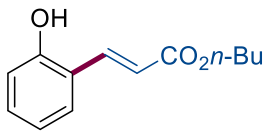

(*E*)-*n*-Butyl-3-(2-hydroxyphenyl)acrylate (*o*-9) & (*E*)-*n*-butyl-3-(4-hydroxyphenyl) acrylate (*p*-9) & 2*H*-chromen-2-one (*o*'-9)

The general procedure **C** was followed using phenol (**1f**) (70.6 mg, 0.75 mmol, 1.5 equiv) and *n*-butyl acrylate (**3a**) (72.0 µL, 0.50 mmol, 1.0 equiv) at 60 °C for 48 h. **L12** was used as ligand. Isolation by column chromatography (*n*-hexane/EtOAc = 5:1) yielded **9** (38.6 mg, 35%) as a mixture and 2*H*-chromen-2-one (11.7 mg, 16%). The *o* and *p*-olefinated products are known compounds.[33] 2*H*-chromen-2-one was known as well.[34] The ratio of the isomers was determined by the ^{1}H-NMR of crude mixture, $o : p : o'$ = 2 : 1 : 1. ^{1}H-NMR (600 MHz, $CDCl_3$): δ = 8.03 (d, J = 16.1 Hz, 1H^o), 7.63 (d, J = 16.0 Hz, 1H^p), 7.47 (dd, J = 7.8, 1.7 Hz, 1H^o), 7.43 – 7.39 (m, 2H^p), 7.27 – 7.20 (m, 1H^o), 6.93 – 6.89 (m, 1H^o), 6.88 – 6.84 (m, 1H^o+2H^p), 6.63 (d, J = 16.1 Hz, 1H^o), 6.30 (d, J = 15.9 Hz, 1H^p), 4.22 (m, 2H^{o+p}), 1.74 – 1.64 (m, 2H^{o+p}), 1.50 – 1.39 (m, 2H^{o+p}), 1.00 – 0.92 (m, 3H^{o+p}). ^{13}C-NMR (150 MHz, $CDCl_3$): δ = 168.6 (C_q), 167.9 (C_q), 158.0 (C_q), 155.4 (C_q), 144.6 (CH), 140.6 (CH), 131.4 (CH), 129.9 (CH), 129.2 (CH), 127.0 (C_q), 121.7 (C_q), 120.6 (CH), 118.4 (CH), 116.4 (CH), 115.9 (CH), 115.4 (CH), 64.6 (CH_2), 64.5 (CH_2), 30.8 (CH_2), 30.7 (CH_2), 19.2 (CH_2), 13.7 (CH_3). IR (ATR): 3335, 2959, 2933, 2873, 1705, 1586, 1453, 1167, 828, 751 cm^{-1}. MS (ESI) m/z (relative intensity): 243 (100) $[M + Na]^+$, 221 (50) $[M + H]^+$. HR-MS (ESI): m/z calcd. for $[C_{13}H_{16}O_3 + Na]^+$ 243.0992 found 243.0985. For *o*'-**9**, ^{1}H-NMR (600 MHz, $CDCl_3$): δ = 7.71 (dd, J = 9.5, 0.6 Hz, 1H), 7.54 (ddd,

J = 8.6, 7.3, 1.6 Hz, 1H), 7.49 (dd, J = 7.7, 1.6 Hz, 1H), 7.34 (ddt, J = 8.3, 1.1, 0.5 Hz, 1H), 7.28 (td, J = 7.5, 1.1 Hz, 1H), 6.43 (d, J = 9.5 Hz, 1H). ^{13}C-NMR (150 MHz, $CDCl_3$): δ = 160.8 (C_q), 154.1 (C_q), 143.4 (CH), 131.8 (CH), 127.8 (CH), 124.4 (CH), 118.8 (C_q), 116.9 (CH), 116.7 (CH). IR (ATR): 3069, 2933, 1627, 1320, 1256, 1227, 1063, 984, 599, 524 cm^{-1}. MS (ESI) m/z (relative intensity): 147 (100) $[M + Na]^+$, 169 (10) $[M + H]^+$. HR-MS (ESI): m/z calcd. for $[C_9H_6O_2 + H]^+$ 147.0441 found 147.0434.

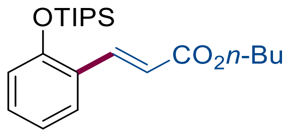

(*E*)-butyl-3-(2-((triisopropylsilyl)oxy)phenyl)acrylate (*o*-10) & (*E*)-butyl-3-(4-((triisopropylsilyl)oxy)phenyl)acrylate (*p*-10)

The general procedure **C** was followed using triisopropyl(phenoxy)silane (**1g**) (212.0 µL, 0.75 mmol, 1.5 equiv) and *n*-butyl acrylate (**3a**) (72.0 µL, 0.50 mmol, 1.0 equiv) at 60 °C for 48 h. **L12** was used as ligand. Isolation by column chromatography (*n*-hexane/EtOAc = 10:1) yielded **10** (146.9 mg, 78%). The site selectivity was determined by COSY NMR and the ratio of the isomers was determined by the ^{1}H-NMR of the crude mixture, $o : p$ = 1 : 1. ^{1}H-NMR (600 MHz, $CDCl_3$): δ = 8.15 (d, J = 16.2 Hz, $1H^o$), 7.62 (d, J = 15.9 Hz, $1H^p$), 7.54 (dd, J = 7.8, 1.7 Hz, $1H^o$), 7.41 (d, J = 8.6 Hz, $2H^p$), 7.22 (ddd, J = 8.2, 7.3, 1.7 Hz, $1H^o$), 6.93 (dddd, J = 7.8, 7.2, 1.1, 0.6 Hz, $1H^o$), 6.88 – 6.86 (m, $2H^p$), 6.85 (dd, J = 8.2, 1.2 Hz, $1H^o$), 6.39 (d, J = 16.2 Hz, $1H^o$), 6.30 (d, J = 15.9 Hz, $1H^p$), 4.23 – 4.17 (m, $2H^{o+p}$), 1.71 – 1.65 (m, $2H^{o+p}$), 1.48 – 1.40 (m, $2H^{o+p}$), 1.36 – 1.24 (m, $3H^{o+p}$), 1.15 – 1.08 (m, $18H^{o+p}$), 0.99 – 0.94 (m, $3H^{o+p}$). ^{13}C-NMR (125 MHz, $CDCl_3$): δ = 167.4 (C_q), 167.3 (C_q), 158.2 (C_q), 155.0 (C_q), 144.3 (CH), 139.8 (CH), 131.2 (CH), 129.6 (CH), 127.5 (C_q), 127.3 (CH), 125.5 (C_q), 121.1 (CH), 120.3 (CH), 119.3 (CH), 117.8 (CH), 115.8 (CH), 64.2 (CH_2), 64.2 (CH_2), 30.8 (CH_2), 30.8 (CH_2), 19.2 (CH_2), 19.2 (CH_2), 18.0 (CH_3), 17.9 (CH_3), 13.7 (CH_3), 13.0 (CH), 12.6 (CH). IR (ATR): 2945, 1712, 1633, 1600, 1509, 1455, 1267, 1165, 910, 882, 832 cm^{-1}. MS (ESI) m/z (relative intensity): 399 (100) $[M + Na]^+$, 377 (15) $[M + H]^+$. HR-MS (ESI): m/z calcd. for $[C_{22}H_{36}O_3Si + H]^+$ 377.2506 found 377.2501.

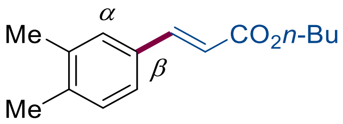

(*E*)-*n*-Butyl-3-(2,3-dimethylphenyl)acrylate (*α*-11) & (*E*)-*n*-butyl-3-(3,4-dimethylphenyl)acrylate (*β*-11)

The general procedure **C** was followed using *o*-xylene (**1h**) (120.6 µL, 1.0 mmol, 2.0 equiv) and *n*-butyl acrylate (**3a**) (72.0 µL, 0.50 mmol, 1.0 equiv) at 60 °C for 48 h. **L12** was used as ligand. Isolation by column chromatography (*n*-hexane/EtOAc = 50:1 to 20:1) yielded **11** (87.3 mg, 75%) as a colorless oil. The *α* and *β*-olefinated products are known compounds[31] and the ratio of the isomers was determined by the ^{1}H-NMR of the crude mixture, $\alpha : \beta = 1 : 1.6$. ^{1}H-NMR (600 MHz, $CDCl_3$): δ = 8.03 (d, J = 15.8 Hz, 1H^{α}), 7.60 (d, J = 16.0 Hz, 1H^{β}), 7.39 – 6.99 (m, 3$H^{\alpha+\beta}$), 6.35 (d, J = 16.0 Hz, 1H^{β}), 6.27 (d, J = 15.8 Hz, 1H^{α}), 4.25 – 4.10 (m, 2$H^{\alpha+\beta}$), 2.33 – 2.18 (m, 6$H^{\alpha+\beta}$), 1.74 – 1.59 (m, 2$H^{\alpha+\beta}$), 1.48 – 1.35 (m, 2$H^{\alpha+\beta}$), 0.98 – 0.89 (m, 3$H^{\alpha+\beta}$). ^{13}C-NMR (150 MHz, $CDCl_3$): δ = 167.3 (C_q), 167.2 (C_q), 144.7 (CH), 143.3 (CH), 139.3 (C_q), 137.3 (C_q), 137.0 (C_q), 136.0 (C_q), 133.8 (C_q), 132.1 (C_q), 131.4 (CH), 130.1 (CH), 129.2 (CH), 125.7 (CH), 125.6 (CH), 124.4 (CH), 119.7 (CH), 117.0 (CH), 64.3 (CH_2), 64.2 (CH_2), 30.8 (CH_2), 30.8 (CH_2), 20.5 (CH_3), 19.7 (CH_3), 19.7 (CH_3), 19.2 (CH_2), 15.4 (CH_3), 13.7 (CH_3). IR (ATR): 3060, 2957, 2933, 2872, 1711, 1455, 1312, 1236, 982, 817 cm^{-1}. MS (ESI) *m*/*z* (relative intensity): 255 (100) $[M + Na]^+$, 233 (100) $[M + H]^+$. HR-MS (ESI): *m*/*z* calcd. for $[C_{15}H_{20}O_2 + H]^+$ 233.1536 found 233.1534.

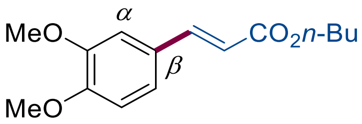

(*E*)-*n*-Butyl-3-(2,3-dimethoxyphenyl)acrylate (*o*-12) & (*E*)-*n*-Butyl-3-(3,4-dimethoxyphenyl)acrylate (*p*-12)

The general procedure **C** was followed using 1,2-dimethoxybenzene (**1i**) (97.0 µL, 0.75 mmol, 1.5 equiv) and *n*-butyl acrylate (**3a**) (72.0 µL, 0.50 mmol, 1.0 equiv) at 60 °C for 48 h. **L12** was used as ligand. Isolation by column chromatography (*n*-hexane/EtOAc = 10:1 to 5:1) yielded **12** (71.4 mg, 54%) as a colorless oil and 16% difunctionalized product. The *α* and *β*-

olefinated products are known compounds[31] and the ratio of the isomers was determined by the ^{1}H-NMR of the crude mixture, $\alpha : \beta = 1 : 5$. For ***p*-12**, ^{1}H-NMR (600 MHz, $CDCl_3$): δ = 7.61 (d, J = 15.9 Hz, 1H), 7.09 (dd, J = 8.6, 2.0 Hz, 1H), 7.04 (d, J = 2.0 Hz, 1H), 6.85 (d, J = 8.2 Hz, 1H), 6.30 (d, J = 15.9 Hz, 1H), 4.19 (t, J = 6.7 Hz, 2H), 3.90 (s, 3H), 3.89 (s, 3H), 1.71 – 1.63 (m, 2H), 1.47 – 1.38 (m, 2H), 0.95 (t, J = 7.4 Hz, 3H). ^{13}C-NMR (150 MHz, $CDCl_3$): δ = 167.3 (C_q), 151.0 (C_q), 149.1 (C_q), 144.4 (CH), 127.4 (C_q), 122.5 (CH), 115.9 (CH), 111.0 (CH), 109.5 (CH), 64.2 (CH_2), 55.9 (CH_3), 55.8 (CH_3), 30.8 (CH_2), 19.2 (CH_2), 13.7 (CH_3). IR (ATR): 3000, 2957, 2932, 2871, 2834, 1704, 1633, 1512, 1257, 1025 cm^{-1}. MS (ESI) *m*/*z* (relative intensity): 287 (100) $[M + Na]^+$, 265 (50) $[M + H]^+$. HR-MS (ESI): *m*/*z* calcd. for $[C_{15}H_{20}O_4 + Na]^+$ 287.1254 found 287.1252.

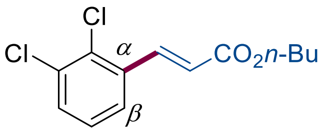

(*E*)-*n*-Butyl-3-(2,3-dichlorophenyl)acrylate (*α*-13) & (*E*)-*n*-butyl-3-(3,4-dichlorophenyl) acrylate (*β*-13)

The general procedure **A** was followed using 1,2-dichlorobenzene (**1j**) (225.1 µL, 2.0 mmol, 10 equiv) and *n*-butyl acrylate (**3a**) (28.8 µL, 0.20 mmol, 1.0 equiv) at 100 °C for 20 h. **L12** was used as ligand. Isolation by column chromatography (*n*-hexane/EtOAc = 50:1 to 20:1) yielded **13** (24.0 mg, 44%) as a colorless oil. The *α* and *β*-olefinated products are known compound[30] and the ratio of the isomers was determined by the ^{1}H-NMR of the crude mixture, $\alpha : \beta = 1.5 : 1$. ^{1}H-NMR (600 MHz, $CDCl_3$): δ = 8.07 (d, J = 15.9 Hz, $1H^{\alpha}$), 7.59 (d, J = 2.1 Hz, $1H^{\beta}$), 7.57 – 7.53 (d, J = 15.9 Hz, $1H^{\beta}$), 7.51 (ddd, J = 7.9, 1.5, 0.5 Hz, $1H^{\alpha}$), 7.47 (dd, J = 8.0, 1.5 Hz, $1H^{\alpha}$), 7.45 (d, J = 8.3 Hz, $1H^{\beta}$), 7.34 (ddd, J = 8.3, 2.1, 0.5 Hz, $1H^{\beta}$), 7.21 (td, J = 7.9, 0.6 Hz, $1H^{\alpha}$), 6.44 – 6.38 (m, $1H^{\alpha+\beta}$), 4.26 – 4.17 (m, $2H^{\alpha+\beta}$), 1.73 – 1.65 (m, $2H^{\alpha+\beta}$), 1.48 – 1.39 (m, $2H^{\alpha+\beta}$), 0.99 – 0.94 (m, $3H^{\alpha+\beta}$). ^{13}C-NMR (150 MHz, $CDCl_3$): δ = 166.4 (C_q), 166.2 (C_q), 141.7 (CH), 140.2 (CH), 135.1 (C_q), 134.5 (C_q), 134.0 (C_q), 133.9 (C_q), 133.2 (C_q), 132.9 (C_q), 131.4 (CH), 130.8 (CH), 129.5 (CH), 127.3 (CH), 126.9 (CH), 125.7 (CH), 122.2 (CH), 120.1 (CH), 64.7 (CH_2), 64.6 (CH_2), 30.7 (CH_2), 19.1 (CH_2), 13.7 (CH_3), 13.7 (CH_3). IR (ATR): 3064, 2932, 2872, 1713, 1638, 1309, 1274, 1176, 979, 785 cm^{-1}. MS (ESI) *m*/*z* (relative intensity): 295 (100) $[M + Na]^+$, 273 (20) $[M + H]^+$. HR-MS (ESI): *m*/*z* calcd. for $[C_{13}H_{14}O_2Cl_2 + Na]^+$ 295.0263 found 295.0255.

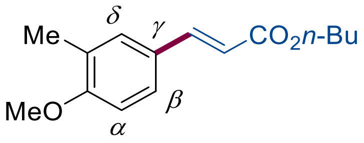

(*E*)-butyl-3-(3-methyl-2-methoxyphenyl)acrylate (*α*-14) & (*E*)-butyl-3-(4-methyl-3-methoxyphenyl)acrylate (*β*-14) & (*E*)-butyl-3-(3-methyl-4-methoxyphenyl)acrylate (*γ*-14) & (*E*)-butyl-3-(2-methyl-3-methoxyphenyl)acrylate (*δ*-14)

The general procedure **C** was followed using 1-methoxy-2-methylbenzene (**1k**) (93.5 µL, 0.75 mmol, 1.5 equiv) and *n*-butyl acrylate (**3a**) (72.0 µL, 0.50 mmol, 1.0 equiv) at 60 °C for 48 h. **L12** was used as ligand. Isolation by column chromatography (*n*-hexane/EtOAc = 30:1 to 15:1) yielded **14** (77.0 mg, 62%) and 16% difunctionalized product. The site selectivity was determined by ^{1}H-NMR and NOESY NMR, *γ : others* = 5 : 1. For *γ*-**15**, ^{1}H-NMR (600 MHz, $CDCl_3$): δ = 7.61 (d, J = 15.9 Hz, 1H), 7.35 – 7.32 (m, 2H), 6.81 (d, J = 9.0 Hz, 1H), 6.30 (d, J = 15.9 Hz, 1H), 4.19 (t, J = 6.7 Hz, 2H), 3.86 (s, 3H), 2.22 (s, 3H), 1.72 – 1.65 (m, 2H), 1.48 – 1.39 (m, 2H), 0.96 (t, J = 7.4 Hz, 3H). ^{13}C-NMR (125 MHz, $CDCl_3$): δ = 167.5 (C_q), 159.6 (C_q), 144.5 (CH), 130.0 (CH), 127.8 (CH), 127.2 (C_q), 126.7 (C_q), 115.4 (CH), 109.9 (CH), 64.2 (CH_2), 55.4 (CH_3), 30.8 (CH_2), 19.2 (CH_2), 16.2 (CH_3), 13.8 (CH_3). IR (ATR): 2959, 2933, 2252, 1702, 1604, 1502, 1254, 1175, 1132, 909 cm^{-1}. MS (ESI) *m*/*z* (relative intensity): 271 (100) [M + Na]$^+$, 249 (0) [M + H]$^+$. HR-MS (ESI): *m*/*z* calcd. for [$C_{15}H_{20}O_3$ + Na]$^+$ 271.1305 found 271.1304.

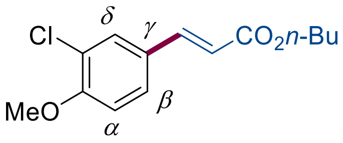

(*E*)-butyl-3-(3-chloro-2-methoxyphenyl)acrylate (*α*-15) & (*E*)-butyl-3-(4-chloro-3-methoxyphenyl)acrylate (*β*-15) & (*E*)-butyl-3-(3-chloro-4-methoxyphenyl)acrylate (*γ*-15) & (*E*)-butyl-3-(2-chloro-3-methoxyphenyl)acrylate (*δ*-15)

The general procedure **A** was followed using 1-chloro-2-methoxybenzene (**1l**) (228.5 µL, 2.0 mmol, 10 equiv) and *n*-butyl acrylate (**3a**) (28.8 µL, 0.20 mmol, 1.0 equiv) at 80 °C for 20 h. **L12** was used as ligand. Isolation by column chromatography (*n*-hexane/EtOAc = 30:1 to 15:1) yielded **15** (28.5 mg, 53%). The site selectivity was determined by ^{1}H-NMR and NOESY NMR,

$\alpha : \gamma :$ *others* = 1 : 4 : 3. For α-**15**, ^{1}H-NMR (400 MHz, $CDCl_3$): δ = 7.92 (d, J = 16.2 Hz, 1H), 7.46 (dd, J = 7.9, 1.6 Hz, 1H), 7.40 (dd, J = 8.0, 1.6 Hz, 1H), 7.08 (t, J = 7.9 Hz, 1H), 6.51 (d, J = 16.2 Hz, 1H), 4.22 (t, J = 6.7 Hz, 2H), 3.87 (s, 3H), 1.74 – 1.65 (m, 2H), 1.50 – 1.38 (m, 2H), 0.97 (t, J = 7.4 Hz, 3H). ^{13}C-NMR (100 MHz, $CDCl_3$): δ = 166.9 (C_q), 155.1 (C_q), 138.5 (CH), 132.0 (CH), 130.1 (C_q), 128.9 (C_q), 126.4 (CH), 125.0 (CH), 120.8 (CH), 64.6 (CH_2), 61.6 (CH_3), 30.8 (CH_2), 19.2 (CH_2), 13.8 (CH_3). For γ-**15**, ^{1}H-NMR (400 MHz, $CDCl_3$): δ = 7.57 (d, J = 2.2 Hz, 1H), 7.56 (d, J = 15.9 Hz, 1H), 7.39 (dd, J = 8.6, 2.2 Hz, 1H), 6.93 (d, J = 8.5 Hz, 1H), 6.32 (d, J = 16.0 Hz, 1H), 4.20 (t, J = 6.7 Hz, 2H), 3.93 (s, 3H), 1.75 – 1.62 (m, 2H), 1.50 – 1.37 (m, 2H), 0.96 (t, J = 7.4 Hz, 3H). ^{13}C-NMR (100 MHz, $CDCl_3$): δ = 167.1 (C_q), 156.5 (C_q), 142.8 (CH), 129.5 (CH), 128.2 (CH), 128.1 (C_q), 123.2 (C_q), 117.3 (CH), 112.0 (CH), 64.5 (CH_2), 56.3 (CH_3), 30.80 (CH_2), 19.2 (CH_2), 13.8 (CH_3). IR (ATR): 2959, 2934, 2873, 1710, 1637, 1572, 1471, 1268, 1172, 1064 cm^{-1}. MS (ESI) *m*/*z* (relative intensity): 291 (100) $[M + Na]^+$, 269 (0) $[M + H]^+$. HR-MS (ESI): *m*/*z* calcd. for $[C_{14}H_{17}ClO_3 + Na]^+$ 291.0758 found 291.0751.

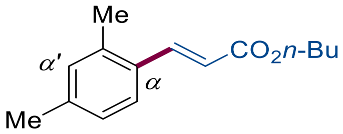

(*E*)-*n*-Butyl-3-(1,6-dimethylphenyl)acrylate (α'-16) & (*E*)-*n*-butyl-3-(2,4-dimethylphenyl)acrylate (α-16) & (*E*)-*n*-butyl-3-(3,5-dimethylphenyl)acrylate (β-16)

The general procedure **C** was followed using *m*-xylene (**1m**) (91.7 µL, 0.75 mmol, 1.5 equiv) and *n*-butyl acrylate (**3a**) (72.0 µL, 0.50 mmol, 1.0 equiv) at 60 °C for 48 h. **L12** was used as ligand. Isolation by column chromatography (*n*-hexane/EtOAc = 50:1 to 20:1) yielded **16** (58.1 mg, 50%) as a colorless oil and 16% difunctionalized product. The α', α and β-olefinated products are known compounds[33] and the ratio of the isomers was determined by the ^{1}H-NMR of the crude mixture, $\beta : \alpha : \alpha'$ = 1 : 9 : 2. ^{1}H-NMR (600 MHz, $CDCl_3$): δ = 7.96 (d, J = 15.9 Hz, $1H^{\alpha}$), 7.85 (d, J = 16.4 Hz, $1H^{\alpha'}$), 7.63 (d, J = 16.0 Hz, $1H^{\beta}$), 7.47 (d, J = 8.4 Hz, $1H^{\alpha}$), 7.19 – 6.98 (m, $3H^{\alpha'+\beta}$ + $2H^{\alpha}$), 6.42 (d, J = 16.0 Hz, $1H^{\beta}$), 6.34 (d, J = 15.8 Hz, $1H^{\alpha}$), 6.07 (d, J = 16.4 Hz, $1H^{\alpha'}$), 4.22 (m, $2H^{\alpha'+\alpha+\beta}$), 2.43 – 2.32 (m, $6H^{\alpha'+\alpha+\beta}$), 1.86 – 1.61 (m, $2H^{\alpha'+\alpha+\beta}$), 1.54 – 1.41 (m, $2H^{\alpha'+\alpha+\beta}$), 1.08 – 0.89 (m, $3H^{\alpha'+\alpha+\beta}$). ^{13}C-NMR (150 MHz, $CDCl_3$): δ = 167.3 (C_q), 167.2 (C_q), 166.9 (C_q), 144.8 (CH), 143.2 (CH), 142.1 (CH), 140.2 (C_q), 138.3 (C_q), 137.6

(C_q), 136.6 (C_q), 134.4 (C_q), 134.0 (C_q), 132.0 (CH), 131.5 (CH), 130.5 (C_q), 128.2 (CH), 128.2 (CH), 127.1 (CH), 126.3 (CH), 125.9 (CH), 123.9 (CH), 118.1 (CH), 117.8 (CH), 64.5 (CH_2), 64.3 (CH_2), 30.8 (CH_2), 30.8 (CH_2), 30.7 (CH_2), 21.3 (CH_3), 21.1 (CH_3), 21.0 (CH_3), 19.7 (CH_3), 19.2 (CH_2), 13.7 (CH_3). IR (ATR): 2958, 2930, 2872, 1711, 1633, 1610, 1310, 1274, 1159, 981 cm^{-1}. MS (ESI) *m*/*z* (relative intensity): 255 (95) $[M + Na]^+$, 233 (100) $[M + H]^+$. HR-MS (ESI): *m*/*z* calcd. for $[C_{15}H_{20}O_2 + H]^+$ 233.1536 found 233.1532.

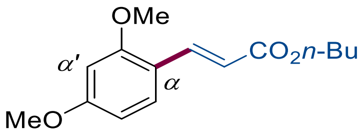

(*E*)-*n*-Butyl-3-(2,6-dimethoxyphenyl)acrylate (α'-17) & (*E*)-*n*-butyl-3-(2,4-dimethoxyphenyl)acrylate (α-17)

The general procedure **C** was followed using 1,3-dimethoxybenzene (**1n**) (97.0 µL, 0.75 mmol, 1.5 equiv) and *n*-butyl acrylate (**3a**) (72.0 µL, 0.50 mmol, 1.0 equiv) at 60 °C for 48 h. **L12** was used as ligand. Isolation by column chromatography (*n*-hexane/EtOAc = 10:1 to 5:1) yielded **17** (88.6 mg, 67%) as a colorless oil. The α', α and β-olefinated products are known compounds[31] and the ratio of the isomers was determined by the ^{1}H-NMR of the crude mixture, $\beta : \alpha : \alpha'$ = 1 : 3 : 1. ^{1}H-NMR (400 MHz, $CDCl_3$): δ = 8.13 (d, J = 16.3 Hz, $1H^{\alpha'}$), 7.90 (d, J = 16.1 Hz, $1H^{\alpha}$), 7.43 (d, J = 8.5 Hz, $1H^{\alpha}$), 7.25 (t, J = 8.4 Hz, $1H^{\alpha'}$), 6.87 (d, J = 16.3 Hz, $1H^{\alpha'}$), 6.55 (d, J = 8.4 Hz, $2H^{\alpha'}$), 6.49 (dd, J = 8.5, 2.4 Hz, $1H^{\alpha}$), 6.46 – 6.40 (m, $2H^{\alpha}$), 4.22 – 4.16 (m, $2H^{\alpha'+\alpha}$), 3.90 – 3.81 (m, $6H^{\alpha'+\alpha}$), 1.72 – 1.63 (m, $2H^{\alpha'+\alpha}$), 1.49 – 1.37 (m, $2H^{\alpha'+\alpha}$), 0.99 – 0.93 (m, $3H^{\alpha'+\alpha}$). ^{13}C-NMR (100 MHz, $CDCl_3$): δ = 168.7 (C_q), 168.0 (C_q), 162.6 (C_q), 160.0 (C_q), 159.8 (C_q), 139.9 (CH), 135.3 (CH), 131.1 (CH), 130.4 (CH), 120.7 (CH), 116.6 (C_q), 116.1 (CH), 112.3 (C_q), 105.1 (CH), 103.6 (CH), 98.4 (CH), 64.1 (CH_2), 64.0 (CH_2), 55.7 (CH_3), 55.4 (CH_3), 30.9 (CH_2), 30.8 (CH_2), 19.2 (CH_2), 13.7 (CH_3). IR (ATR): 3003, 2958, 2934, 2872, 2838, 1703, 1604, 1300, 1255, 1210 cm^{-1}. MS (ESI) *m*/*z* (relative intensity): 287 (50) $[M + Na]^+$, 265 (100) $[M + H]^+$. HR-MS (ESI): *m*/*z* calcd. for $[C_{15}H_{20}O_4 + H]^+$ 265.1434 found 265.1430.

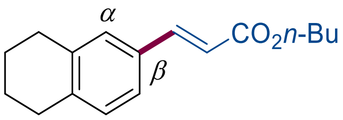

(*E*)-*n*-Butyl-3-(5,6,7,8-tetrahydronaphthalen-1-yl)acrylate (*α*-18) & (*E*)-*n*-butyl-3-(5,6,7,8-tetrahydronaphthalen-2-yl)acrylate (*β*-18)

The general procedure **C** was followed using 1,2,3,4-tetrahydronaphthalene (**1o**) (102.2 µL, 0.75 mmol, 1.5 equiv) and *n*-butyl acrylate (**3a**) (72.0 µL, 0.50 mmol, 1.0 equiv) at 60 °C for 48 h. **L12** was used as ligand. Isolation by column chromatography (*n*-hexane/EtOAc = 50:1 to 20:1) yielded **18** (81.4 mg, 63%) as a colorless oil. The *α*-olefinated product is known compound[30] and the ratio of the isomers was determined by the ^{1}H-NMR of the crude mixture, *α* : *β* = 1 : 1 (1 : 1.4). ^{1}H-NMR (600 MHz, $CDCl_3$): δ = 7.99 (d, *J* = 15.8 Hz, $1H^{\alpha}$), 7.63 (d, *J* = 16.0 Hz, $1H^{\beta}$), 7.40 – 7.03 (m, $3H^{\alpha+\beta}$), 6.39 (d, *J* = 16.0 Hz, $1H^{\beta}$), 6.32 (d, *J* = 15.8 Hz, $1H^{\alpha}$), 4.25 – 4.16 (m, $2H^{\alpha+\beta}$), 2.92 – 2.73 (m, $4H^{\alpha+\beta}$), 1.92 – 1.76 (m, $4H^{\alpha+\beta}$), 1.75 – 1.64 (m, $2H^{\alpha+\beta}$), 1.49 – 1.40 (m, $2H^{\alpha+\beta}$), 1.09 – 0.92 (m, $3H^{\alpha+\beta}$). ^{13}C-NMR (150 MHz, $CDCl_3$): δ = 167.3 (C_q), 167.2 (C_q), 144.8 (CH), 142.4 (CH), 140.0 (C_q), 137.9 (C_q), 137.6 (C_q), 136.3 (C_q), 133.6 (C_q), 131.7 (C_q), 131.1 (CH), 129.6 (CH), 129.0 (CH), 125.5 (CH), 125.0 (CH), 124.0 (CH), 119.5 (CH), 116.9 (CH), 64.3 (CH_2), 64.3 (CH_2), 30.8 (CH_2), 30.8 (CH_2), 30.0 (CH_2), 29.4 (CH_2), 29.3 (CH_2), 26.5 (CH_2), 23.1 (CH_2), 23.0 (CH_2), 23.0 (CH_2), 22.5 (CH_2), 19.2 (CH_2), 13.7 (CH_3). IR (ATR): 3379, 2955, 2931, 2864, 1711, 1634, 1309, 1222, 1171, 983 cm^{-1}. MS (ESI) *m*/*z* (relative intensity): 281 (100) $[M + Na]^+$, 259 (100) $[M + H]^+$. HR-MS (ESI): *m*/*z* calcd. for $[C_{17}H_{22}O_2 + H]^+$ 259.1693 found 259.1688.

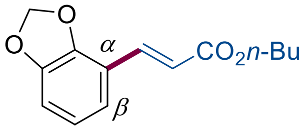

(*E*)-butyl-3-(benzo[*d*][1,3]dioxol-4-yl)acrylate (*α*-19) & (*E*)-Butyl 3-(benzo[*d*][1,3]dioxol-5-yl)acrylate (*β*-19)

The general procedure **C** was followed using 1,3-Benzodioxole (**1p**) (86.1 µL, 0.75 mmol, 1.5 equiv) and *n*-butyl acrylate (**3a**) (72.0 µL, 0.50 mmol, 1.0 equiv) at 60 °C for 48 h. **L12** was used as ligand. Isolation by column chromatography (*n*-hexane/EtOAc = 10:1) yielded **19** (83.2 mg, 67%). The *β*-olefinated products are known compounds[35] and the ratio of the isomers was determined by the ^{1}H-NMR of the crude mixture, *α* : *β* = 2 : 1. For *α*-**19**, ^{1}H-NMR (300 MHz,

$CDCl_3$): δ = 7.60 (d, J = 16.1 Hz, 1H), 6.94 – 6.88 (m, 1H), 6.84 – 6.80 (m, 2H), 6.63 (d, J = 16.0 Hz, 1H), 6.05 (s, 2H), 4.20 (t, J = 6.7 Hz, 2H), 1.75 – 1.62 (m, 2H), 1.44 (ddt, J = 14.4, 9.6, 7.3 Hz, 2H), 0.96 (t, J = 7.3 Hz, 3H). ^{13}C-NMR (75 MHz, $CDCl_3$): δ = 167.5 (C_q), 148.1 (C_q), 146.6 (C_q), 139.1 (CH), 122.7 (CH), 121.9 (CH), 121.1 (CH), 117.6 (C_q), 109.9 (CH), 101.6 (CH_2), 64.6 (CH_2), 30.9 (CH_2), 19.3 (CH_2), 13.9 (CH_3). For *β*-**19**, ^{1}H-NMR (300 MHz, $CDCl_3$): δ = 7.58 (d, J = 15.9 Hz, 1H), 7.06 – 6.96 (m, 2H), 6.81 (d, J = 8.0 Hz, 1H), 6.26 (d, J = 15.9 Hz, 1H), 6.00 (s, 2H), 4.19 (t, J = 6.7 Hz, 2H), 1.73 – 1.62 (m, 2H), 1.42 (dt, J = 14.5, 7.4 Hz, 2H), 0.96 (t, J = 7.4 Hz, 3H).^{13}C-NMR (75 MHz, $CDCl_3$): δ = 167.4 (C_q), 149.7 (C_q), 148.5 (C_q), 144.4 (CH), 129.1 (C_q), 124.5 (CH), 116.4 (CH), 108.7 (CH), 106.6 (CH), 101.7 (CH_2), 64.5 (CH_2), 30.9 (CH_2), 19.4 (CH_2), 13.9 (CH_3). IR (ATR): 2959, 2874, 1707, 1632, 1450, 1315, 1270, 1149, 1072, 1056 cm^{-1}. MS (ESI) *m/z* (relative intensity): 271 (100) $[M + Na]^+$. HR-MS (ESI): *m/z* calcd. for $[C_{14}H_{16}O_4 + Na]^+$ 271.0941 found 271.0949.

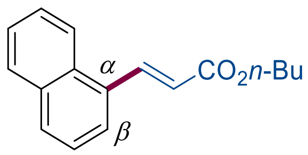

(*E*)-*n*-Butyl-3-(naphthalen-1-yl)acrylate (*α*-20) & (*E*)-*n*-butyl-3-(naphthalen-2-yl)acrylate (*β*-20)

The general procedure **C** was followed using naphthalene (**1q**) (96.2 mg, 0.75 mmol, 1.5 equiv) and *n*-butyl acrylate (**3a**) (72.0 µL, 0.50 mmol, 1.0 equiv) at 60 °C for 48 h. **L12** was used as ligand. Isolation by column chromatography (*n*-hexane/EtOAc = 50:1 to 20:1) yielded **20** (76.3 mg, 60%) as a mixture. The *α* and *β*-olefinated products are known compounds[31] and the ratio of the isomers was determined by the ^{1}H-NMR of the crude mixture, $\alpha : \beta$ = 3 : 1. ^{1}H-NMR (600 MHz, $CDCl_3$): δ = 8.54 (d, J = 15.7 Hz, $1H^{\beta}$), 8.20 (d, J = 8.5 Hz, $1H^{\beta}$), 8.20 (s, $1H^{\alpha}$), 7.91 – 7.81 (m, $4H^{\alpha}$+$2H^{\beta}$), 7.76 (d, J = 7.2 Hz, $1H^{\beta}$), 7.67 (dd, J = 8.6, 1.8 Hz, $1H^{\alpha}$), 7.61 – 7.46 (m, $2H^{\alpha}$+$3H^{\beta}$), 6.58 – 6.52 (m, $1H^{\alpha+\beta}$), 4.39 – 4.19 (m, $2H^{\alpha+\beta}$), 1.78 – 1.69 (m, $2H^{\alpha+\beta}$), 1.53 – 1.43 (m, $2H^{\alpha+\beta}$), 1.04 – 0.97 (m, $3H^{\alpha+\beta}$). ^{13}C-NMR (150 MHz, $CDCl_3$): δ = 167.1 (C_q), 166.9 (C_q), 144.5 (CH), 141.5 (CH), 134.1 (C_q), 133.6 (C_q), 133.2 (C_q), 131.9 (C_q), 131.8 (C_q), 131.4 (C_q), 130.4 (CH), 129.8 (CH), 128.7 (CH), 128.6 (CH), 128.5 (CH), 127.7 (CH), 127.1 (CH), 126.8 (CH), 126.6 (CH), 126.2 (CH), 125.4 (CH), 124.9 (CH), 123.5 (CH), 123.3 (CH), 120.9 (CH), 118.4 (CH), 64.5 (CH_2), 64.4 (CH_2), 30.8 (CH_2), 19.2 (CH_2), 19.2 (CH_2), 13.7

(CH_3), 13.7 (CH_3). IR (ATR): 3058, 2958, 2932, 2872, 1708, 1633, 1303, 1166, 977, 776 cm^{-1}. MS (ESI) *m*/*z* (relative intensity): 277 (100) $[M + Na]^+$, 255 (50) $[M + H]^+$. HR-MS (ESI): *m*/*z* calcd. for $[C_{17}H_{18}O_2 + Na]^+$ 277.1199 found 277.1191.

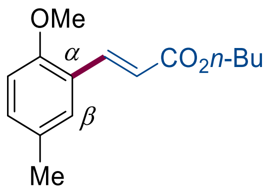

(*E*)-*n*-Butyl-3-(2-methoxy-5-methylphenyl)acrylate (*α*-21) & (*E*)-*n*-butyl-3-(5-methoxy-2-methylphenyl)acrylate (*β*-21)

The general procedure **C** was followed using 1-methoxy-4-methylbenzene (**1r**) (94.6 μL, 0.75 mmol, 1.5 equiv) and *n*-butyl acrylate (**3a**) (72.0 μL, 0.50 mmol, 1.0 equiv) at 60 °C for 48 h. **L12** was used as ligand. Isolation by column chromatography (*n*-hexane/EtOAc = 30:1 to 15:1) yielded **21** (74.5 mg, 60%) as a colorless oil and 34% difunctionalized product. The site selectivity was determined by NOESY NMR and the ratio of the isomers was determined by the ^{1}H-NMR of the crude mixture, $\alpha : \beta$ = 19 : 1. ^{1}H-NMR (600 MHz, $CDCl_3$): δ^α = 7.96 (d, *J* = 16.2 Hz, 1H), 7.31 (d, *J* = 2.2 Hz, 1H), 7.13 (ddd, *J* = 8.4, 2.3, 0.8 Hz, 1H), 6.80 (d, *J* = 8.4 Hz, 1H), 6.52 (d, *J* = 16.1 Hz, 1H), 4.20 (t, *J* = 6.7 Hz, 2H), 3.85 (s, 3H), 2.29 (s, 3H), 1.76 – 1.63 (m, 2H), 1.49 – 1.39 (m, 2H), 0.96 (t, *J* = 7.4 Hz, 3H). ^{13}C-NMR (150 MHz, $CDCl_3$): δ^α = 167.6 (C_q), 156.3 (C_q), 140.0 (CH), 131.8 (CH), 129.7 (C_q), 129.3 (CH), 123.1 (C_q), 118.5 (CH), 111.1 (CH), 64.2 (CH_2), 55.5 (CH_3), 30.8 (CH_2), 20.3 (CH_3), 19.2 (CH_2), 13.7 (CH_3). IR (ATR): 2959, 2933, 2872, 2252, 1702, 1631, 1250, 1031, 908, 731 cm^{-1}. MS (ESI) *m*/*z* (relative intensity): 271 (100) $[M + Na]^+$, 249 (95) $[M + H]^+$. HR-MS (ESI): *m*/*z* calcd. for $[C_{15}H_{20}O_3 + H]^+$ 249.1485 found 249.1483.

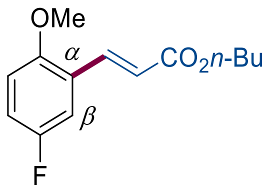

(*E*)-*n*-Butyl-3-(5-fluoro-2-methoxyphenyl)acrylate (*α*-22) & (*E*)-*n*-butyl-3-(2-fluoro-5-methoxyphenyl)acrylate (*β*-22)

The general procedure **C** was followed using 1-fluoro-4-methoxybenzene (**1s**) (141.5 µL, 1.25 mmol, 2.5 equiv) and *n*-butyl acrylate (**3a**) (72.0 µL, 0.50 mmol, 1.0 equiv) at 60 °C for 48 h. **L12** was used as ligand. Isolation by column chromatography (*n*-hexane/EtOAc = 30:1 to 15:1) yielded **22** (73.2 mg, 58%) as mixture. The site-selectivity was determined by ^{13}C NMR and the ratio of the isomers was determined by the ^{1}H-NMR of crude mixture, $\alpha : \beta = 4 : 1$. For α-**22**, ^{1}H-NMR (600 MHz, $CDCl_3$): δ = 7.93 (dd, J = 16.4, 1.1 Hz, 1H), 7.20 (dd, J = 9.1, 3.1 Hz, 1H), 7.03 (ddd, J = 9.1, 7.7, 3.1 Hz, 1H), 6.84 (dd, J = 9.1, 4.4 Hz, 1H), 6.47 (d, J = 16.2 Hz, 1H), 4.20 (t, J = 6.7 Hz, 2H), 3.86 (s, 3H), 1.73 – 1.65 (m, 2H), 1.47 – 1.40 (m, 2H), 0.96 (t, J = 7.4 Hz, 3H). ^{13}C-NMR (150 MHz, $CDCl_3$): δ = 167.1 (C_q), 156.8 (d, J = 239.0 Hz, C_q), 154.4 (d, J = 1.9 Hz, C_q), 138.6 (d, J = 2.3 Hz, CH), 124.6 (d, J = 7.4 Hz, C_q), 119.9 (CH), 117.4 (d, J = 23.1 Hz, CH), 114.5 (d, J = 23.4 Hz, CH), 112.2 (d, J = 8.1 Hz, CH), 64.4 (CH_2), 56.0 (CH_3), 30.8 (CH_2), 19.2 (CH_2), 13.7 (CH_3). ^{19}F-NMR (565 MHz, $CDCl_3$): δ = -123.67 (td, J = 8.4, 4.4 Hz). IR (ATR): 2959, 2931, 2873, 1709, 1633, 1492, 1251, 1170, 1029, 861 cm^{-1}. MS (ESI) *m*/*z* (relative intensity): 275 (100) $[M + Na]^+$, 253 (50) $[M + H]^+$. HR-MS (ESI): *m*/*z* calcd. for $[C_{14}H_{17}O_3F + Na]^+$ 275.1054 found 275.1051. For β-**22**, ^{1}H-NMR (600 MHz, $CDCl_3$): δ = 7.77 (d, J = 16.2 Hz, 1H), 7.06 – 6.98 (m, 2H), 6.88 (m, 1H), 6.51 (d, J = 16.2 Hz, 1H), 4.22 (t, J = 6.7 Hz, 2H), 3.80 (s, 3H), 1.74 – 1.64 (m, 2H), 1.48 – 1.40 (m, 2H), 0.97 (t, J = 7.4 Hz, 3H). ^{13}C-NMR (150 MHz, $CDCl_3$): δ = 166.8 (C_q), 155.9 (d, J = 246.8 Hz, C_q), 155.7 (d, J = 2.1 Hz, C_q), 137.2 (d, J = 2.7 Hz, CH), 122.8 (d, J = 13.3 Hz, C_q), 120.9 (d, J = 6.4 Hz, CH), 117.3 (d, J = 8.3 Hz, CH), 116.8 (d, J = 24.1 Hz, CH), 112.6 (d, J = 3.0 Hz, CH), 64.6 (CH_2), 55.8 (CH_3), 30.7 (CH_2), 19.2 (CH_2), 13.7 (CH_3). ^{19}F-NMR (565 MHz, $CDCl_3$): δ = -125.08 (ddd, J = 9.9, 5.8, 4.1 Hz). IR (ATR): 2959, 2931, 2873, 1709, 1633, 1492, 1251, 1170, 981, 807 cm^{-1}. MS (ESI) *m*/*z* (relative intensity): 275 (100) $[M + Na]^+$, 253 (50) $[M + H]^+$. HR-MS (ESI): *m*/*z* calcd. for $[C_{14}H_{17}O_3F + Na]^+$ 275.1054 found 275.1051.

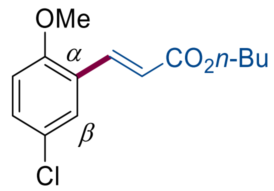

(*E*)-*n*-Butyl-3-(5-chloro-2-methoxyphenyl)acrylate (α-23) & (*E*)-*n*-butyl-3-(2-chloro-5-methoxyphenyl)acrylate (β-23)

The general procedure **C** was followed using 1-chloro-4-methoxybenzene (**1t**) (153.1 µL, 1.25 mmol, 2.5 equiv) and *n*-butyl acrylate (**3a**) (72.0 µL, 0.50 mmol, 1.0 equiv) at 60 °C for 48 h. **L12** was used as ligand. Isolation by column chromatography (*n*-hexane/EtOAc = 30:1 to 15:1) yielded **23** (88.7 mg, 66%) as a colorless oil. The site selectivity was determined by NOESY NMR and the ratio of the isomers was determined by the ^{1}H-NMR of the crude mixture, $\alpha : \beta$ = 8 : 1. ^{1}H-NMR (400 MHz, $CDCl_3$): δ = 7.99 (d, *J* = 16.0 Hz, 1H$^{\beta}$), 7.84 (d, *J* = 16.2 Hz, 1H$^{\alpha}$), 7.41 (d, *J* = 2.6 Hz, 1H$^{\alpha}$), 7.25 – 7.20 (m, 1H$^{\alpha+\beta}$), 7.06 (d, *J* = 2.9 Hz, 1H$^{\beta}$), 6.84 – 6.75 (m, 1H$^{\alpha+\beta}$), 6.44 (d, *J* = 16.1 Hz, 1H$^{\alpha}$), 6.36 (d, *J* = 15.9 Hz, 1H$^{\beta}$), 4.20 – 4.13 (m, 2H$^{\alpha+\beta}$), 3.82 (s, 3H$^{\alpha}$), 3.76 (s, 3H$^{\beta}$), 1.69 – 1.58 (m, 2H$^{\alpha+\beta}$), 1.46 – 1.31 (m, 2H$^{\alpha+\beta}$), 0.96 – 0.88 (m, 3H$^{\alpha+\beta}$). ^{13}C-NMR (100 MHz, $CDCl_3$): δ = 167.1 (C_q), 166.5 (C_q), 158.3 (C_q), 156.7 (C_q), 140.4 (CH), 138.4 (CH), 133.3 (C_q), 130.7 (CH), 130.7 (CH), 128.2 (CH), 126.5 (C_q), 125.7 (C_q), 124.9 (C_q), 120.9 (CH), 120.0 (CH), 117.3 (CH), 112.4 (CH), 112.1 (CH), 64.6 (CH_2), 64.4 (CH_2), 55.8 (CH_3), 55.5 (CH_3), 30.7 (CH_2), 30.7 (CH_2), 19.2 (CH_2), 13.7 (CH_3). IR (ATR): 2958, 2934, 2872, 2840, 1709, 1633, 1484, 1252, 1171, 1026 cm^{-1}. MS (ESI) *m*/*z* (relative intensity): 291 (100) [M + Na]$^+$, 269 (70) [M + H]$^+$. HR-MS (ESI): *m*/*z* calcd. for [$C_{14}H_{17}O_3Cl$ + Na]$^+$ 291.0758 found 291.0753.

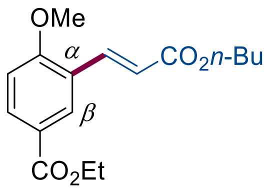

(*E*)-Ethyl-3-(3-butoxy-3-oxoprop-1-en-1-yl)-4-methoxybenzoate (24)

The general procedure **C** was followed using ethyl 4-methoxybenzoate (**1u**) (204.2 µL, 1.25 mmol, 2.5 equiv) and *n*-butyl acrylate (**3a**) (72.0 µL, 0.50 mmol, 1.0 equiv) at 80 °C for 48 h. **L12** was used as ligand. Isolation by column chromatography (*n*-hexane/EtOAc = 10:1 to 5:1) yielded **24** (95.0 mg, 62%) as a colorless oil. ^{1}H-NMR (600 MHz, $CDCl_3$): δ = 8.18 (d, *J* = 2.2 Hz, 1H), 8.01 (dd, *J* = 8.7, 2.2 Hz, 1H), 7.93 (dd, *J* = 16.2, 0.5 Hz, 1H), 6.91 (d, *J* = 8.7 Hz, 1H), 6.56 (d, *J* = 16.2 Hz, 1H), 4.34 (q, *J* = 7.1 Hz, 2H), 4.19 (t, *J* = 6.7 Hz, 2H), 3.92 (s, 3H), 1.70 – 1.62 (m, 2H), 1.45 – 1.39 (m, 2H), 1.37 (t, *J* = 7.1 Hz, 3H), 0.94 (t, *J* = 7.4 Hz, 3H). ^{13}C-NMR (150 MHz, $CDCl_3$): δ = 167.2 (C_q), 165.9 (C_q), 161.4 (C_q), 138.9 (CH), 132.9 (CH), 130.3 (CH), 123.3 (C_q), 123.0 (C_q), 119.9 (CH), 110.6 (CH), 64.36 (CH_2), 60.9 (CH_2), 55.8 (CH_3), 30.8 (CH_2), 19.2 (CH_2), 14.3 (CH_3), 13.7 (CH_3). IR (ATR): 2958, 2933, 2872, 1710,

1632, 1605, 1459, 1306, 1122, 1025 cm^{-1}. MS (ESI) *m*/*z* (relative intensity): 329 (100) $[M + Na]^+$, 307 (50) $[M + H]^+$. HR-MS (ESI): *m*/*z* calcd. for $[C_{17}H_{22}O_5 + Na]^+$ 329.1359 found 329.1355.

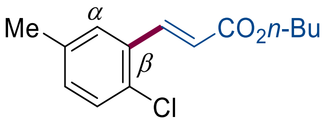

(*E*)-*n*-Butyl-3-(5-chloro-2-methylphenyl)acrylate (*α*-25) & (*E*)-*n*-butyl-3-(2-chloro-5-methylphenyl)acrylate (*β*-25)

The general procedure **C** was followed using 1-chloro-4-methylbenzene (**1v**) (177.5 µL, 1.5 mmol, 3.0 equiv) and *n*-butyl acrylate (**3a**) (72.0 µL, 0.50 mmol, 1.0 equiv) at 80 °C for 48 h. **L12** was used as ligand. Isolation by column chromatography (*n*-hexane/EtOAc = 50:1 to 30:1) yielded **25** (64.2 mg, 51%) as a colorless oil. The site selectivity was determined by NOESY NMR and the ratio of the isomers was determined by the ^{1}H-NMR of the crude mixture, $\alpha : \beta$ = 1 : 2. ^{1}H-NMR (400 MHz, $CDCl_3$): δ = 8.05 (d, *J* = 16.2 Hz, $1H^{\beta}$), 7.86 (d, *J* = 15.9 Hz, $1H^{\alpha}$), 7.50 (d, *J* = 2.3 Hz, $1H^{\alpha}$), 7.41 (d, *J* = 1.7 Hz, $1H^{\beta}$), 7.27 (d, *J* = 8.1 Hz, $1H^{\beta}$), 7.21 (dd, *J* = 8.2, 2.3 Hz, $1H^{\alpha}$), 7.14 – 7.07 (m, $1H^{\alpha+\beta}$), 6.41 (d, *J* = 16.0 Hz, $1H^{\beta}$), 6.34 (d, *J* = 15.9 Hz, $1H^{\alpha}$), 4.26 – 4.16 (m, $2H^{\alpha+\beta}$), 2.38 (s, $3H^{\alpha}$), 2.33 (s, $3H^{\beta}$), 1.75 – 1.64 (m, $2H^{\alpha+\beta}$), 1.52 – 1.37 (m, $2H^{\alpha+\beta}$), 1.01 – 0.91 (m, $3H^{\alpha+\beta}$). ^{13}C-NMR (100 MHz, $CDCl_3$): δ = 166.7 (C_q), 166.6 (C_q), 140.8 (CH), 140.4 (CH), 136.8 (C_q), 135.8 (C_q), 135.0 (C_q), 132.2 (C_q), 132.0 (C_q), 132.0 (CH), 131.9 (C_q), 131.8 (CH), 129.8 (CH), 129.6 (CH), 128.0 (CH), 126.1 (CH), 120.5 (CH), 64.5 (CH_2), 64.5 (CH_2), 30.7 (CH_2), 20.8 (CH_3), 19.2 (CH_2), 13.7 (CH_3). IR (ATR): 2959, 2932, 2874, 1714, 1637, 1472, 1314, 1173, 979, 811 cm^{-1}. MS (ESI) *m*/*z* (relative intensity): 275 (100) $[M + Na]^+$, 253 (40) $[M + H]^+$. HR-MS (ESI): *m*/*z* calcd. for $[C_{14}H_{17}O_2Cl + Na]^+$ 275.0809 found 275.0804.

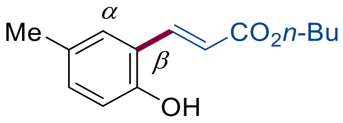

(*E*)-butyl-3-(5-hydroxy-2-methylphenyl)acrylate (*α*-26) & (*E*)-butyl-3-(2-hydroxy-5-methylphenyl)acrylate (*β*-26) & 6-methyl-*2H*-chromen-2-one (*β*'-26)

The general procedure **C** was followed using *p*-cresol (**1w**) (81.1 mg, 0.75 mmol, 1.5 equiv) and *n*-butyl acrylate (**3a**) (72.0 µL, 0.50 mmol, 1.0 equiv) at 60 °C for 48 h. **L12** was used as ligand. Isolation by column chromatography (*n*-hexane/EtOAc = 10:1) yielded **26** (40.8 mg, 51%). The α,β-olefinated products are known compounds[36,37] and the ratio of the isomers was determined by the ^{1}H-NMR of the crude mixture, $\alpha : \beta = 1 : 12$. Product β-**26** and β'-**26** were seperated and characterized. For β-**26**, ^{1}H-NMR (400 MHz, $CDCl_3$): δ = 7.99 (d, J = 16.1 Hz, 1H), 7.26 (d, J = 1.9 Hz, 1H), 7.03 (ddd, J = 8.2, 2.3, 0.8 Hz, 1H), 6.75 (d, J = 8.2 Hz, 1H), 6.61 (d, J = 16.1 Hz, 1H), 6.47 (s, 1H), 4.22 (t, J = 6.7 Hz, 2H), 2.27 (s, 3H), 1.74 – 1.64 (m, 2H), 1.50 – 1.38 (m, 2H), 0.96 (t, J = 7.4 Hz, 3H). ^{13}C-NMR (100 MHz, $CDCl_3$): δ = 168.5 (C_q), 153.2 (C_q), 140.6 (CH), 132.1 (CH), 129.8 (C_q), 129.4 (CH), 121.4 (C_q), 118.2 (CH), 116.3 (CH), 64.5 (CH_2), 30.8 (CH_2), 20.4 (CH_3), 19.2 (CH_2), 13.7 (CH_3). IR (ATR): 2960, 2874, 1681, 1630, 1509, 1313, 1257, 1186, 989, 816 cm^{-1}. MS (ESI) *m*/*z* (relative intensity): 257 (100) $[M + Na]^+$, 235 (10) $[M + H]^+$. HR-MS (ESI): *m*/*z* calcd. for $[C_{14}H_{18}O_3 + Na]^+$ 257.1148 found 257.1151. For β'-**26**, ^{1}H-NMR (600 MHz, $CDCl_3$): δ = 7.65 (d, J = 9.5 Hz, 1H), 7.33 (dd, J = 8.4, 2.2 Hz, 1H), 7.27 (d, J = 1.2 Hz, 1H), 7.23 (d, J = 8.5 Hz, 1H), 6.40 (d, J = 9.5 Hz, 1H), 2.40 (s, 3H). ^{13}C-NMR (150 MHz, $CDCl_3$): δ = 161.1 (C_q), 152.1 (C_q), 143.4 (CH), 134.1 (C_q), 132.8 (CH), 127.6 (CH), 118.5 (C_q), 116.6 (CH), 116.5 (CH), 20.7 (CH_3). IR (ATR): 3079, 2926, 1716, 1573, 1261, 1168, 1100, 906, 811, 759 cm^{-1}. MS (ESI) *m*/*z* (relative intensity): 183 (100) $[M + Na]^+$, 161 (10) $[M + H]^+$. HR-MS (ESI): *m*/*z* calcd. for $[C_{10}H_8O_2 + Na]^+$ 183.0417 found 183.0427.

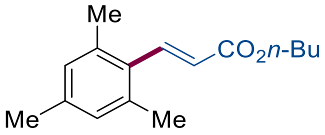

(*E*)-*n*-Butyl-3-mesitylacrylate (27)

The general procedure **C** was followed using mesitylene (**1x**) (104.4 µL, 0.75 mmol, 1.5 equiv) and *n*-butyl acrylate (**3a**) (72.0 µL, 0.50 mmol, 1.0 equiv) at 60 °C for 48 h. **L12** was used as ligand. Isolation by column chromatography (*n*-hexane/EtOAc = 20:1) yielded **27** (86.2 mg, 70%) as a white solid. The product is known compound.[38] ^{1}H-NMR (400 MHz, $CDCl_3$): δ = 7.84 (d, J = 16.3 Hz, 1H), 6.90 (s, 2H), 6.06 (d, J = 16.3 Hz, 1H), 4.22 (t, J = 6.7 Hz, 2H), 2.34 (s, 6H), 2.29 (s, 3H), 1.71 (p, J = 7.0 Hz, 2H), 1.51 – 1.39 (m, 2H), 0.98 (t, J = 7.4 Hz, 3H). ^{13}C-NMR (100 MHz, $CDCl_3$): δ = 167.1 (C_q), 143.1 (CH), 138.3 (C_q), 136.8 (C_q), 131.0 (C_q),

129.1 (CH), 123.2 (CH), 64.4 (CH_2), 30.8 (CH_2), 21.1 (CH_3), 21.1 (CH_3), 19.2 (CH_2), 13.8 (CH_3). IR (ATR): 2961, 2931, 2872, 1708, 1636, 1459, 1305, 1265, 1173, 728 cm^{-1}. MS (ESI) *m*/*z* (relative intensity): 269 (100) $[M + Na]^+$, 247 (20) $[M + H]^+$. HR-MS (ESI): *m*/*z* calcd. for $[C_{16}H_{22}O_2 + Na]^+$ 269.1512 found 269.1508.

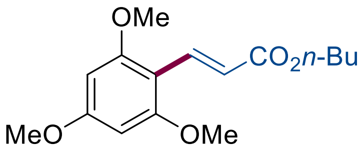

(*E*)-*n*-Butyl-3-(2,4,6-trimethoxyphenyl)acrylate (28)

The general procedure **C** was followed using 1,3,5-trimethoxybenzene (**1y**) (126.2 mg, 0.75 mmol, 1.5 equiv) and *n*-butyl acrylate (**3a**) (72.0 µL, 0.50 mmol, 1.0 equiv) at 60 °C for 40 h. **L12** was used as ligand. Isolation by column chromatography (*n*-hexane/EtOAc = 15:1) yielded **28** (73.6 mg, 50%) as a white solid. The product is known compound.[39] ^{1}H-NMR (600 MHz, $CDCl_3$): δ = 8.08 (d, *J* = 16.2 Hz, 1H), 6.74 (d, *J* = 16.2 Hz, 1H), 6.10 (s, 2H), 4.18 (t, *J* = 6.8 Hz, 2H), 3.86 (s, 6H), 3.83 (s, 3H), 1.73 – 1.61 (m, 2H), 1.47 – 1.34 (m, 2H), 0.95 (t, *J* = 7.4 Hz, 3H). ^{13}C-NMR (150 MHz, $CDCl_3$): δ = 169.1 (C_q), 162.7 (C_q), 161.2 (C_q), 135.4 (CH), 117.6 (CH), 105.9 (C_q), 90.4 (CH), 63.9 (CH_2), 55.7 (CH_3), 55.3 (CH_3), 30.9 (CH_2), 19.2 (CH_2), 13.8 (CH_3). IR (ATR): 2957, 2893, 2841, 1697 ,1599, 1454, 1415, 1293, 1148, 1119 cm^{-1}. MS (ESI) *m*/*z* (relative intensity): 295 (100) $[M + H]^+$, 317 (51) $[M + Na]^+$. HR-MS (ESI): *m*/*z* calcd. for $[C_{16}H_{22}O_5 + H]^+$ 295.1540 found 295.1534.

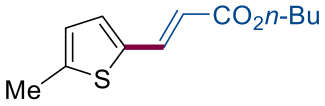

(*E*)-butyl-3-(5-methylthiophen-2-yl)acrylate (α-29) & (*E*)-butyl-3-(2-methylthiophen-3-yl)acrylate (α-29)

The general procedure **C** was followed using 2-methylfuran (**1z**) (72.2 µL, 0.75 mmol, 1.5 equiv) and *n*-butyl acrylate (**3a**) (72.0 µL, 0.50 mmol, 1.0 equiv) at 60 °C for 48 h. **L12** was used as ligand. Isolation by column chromatography (*n*-hexane/EtOAc = 20:1) yielded **29** (58.3 mg, 52%) and 19% difunctionalized product. The site selectivity was determined by HMBC NMR and the ratio of the isomers was determined by the ^{1}H-NMR of the crude mixture, $\alpha : \beta = 6 : 1$. ^{1}H-NMR (600 MHz, $CDCl_3$): δ = 7.68 (dt, *J* = 15.9, 0.7 Hz, $1H^{\beta}$), 7.68 (dd, *J* =

15.6, 0.6 Hz, 1H$^{\alpha}$), 7.16 (d, J = 5.4 Hz, 1H$^{\beta}$), 7.05 (dd, J = 5.4, 0.7 Hz, 1H$^{\beta}$), 7.03 (d, J = 3.6 Hz, 1H$^{\alpha}$), 6.69 (dq, J = 3.4, 1.1 Hz, 1H$^{\alpha}$), 6.19 (d, J = 15.8 Hz, 1H$^{\beta}$), 6.09 (d, J = 15.6 Hz, 1H$^{\alpha}$), 4.21 – 4.14 (m, 2H$^{\alpha+\beta}$), 2.53 (s, 3H$^{\alpha}$), 2.48 (d, J = 1.2 Hz, 3H$^{\beta}$), 1.71 – 1.61 (m, 2H$^{\alpha+\beta}$), 1.48 – 1.37 (m, 2H$^{\alpha+\beta}$), 1.02 – 0.86 (m, 3H$^{\alpha+\beta}$). ^{13}C-NMR (125 MHz, CDCl$_3$): δ = 167.7 (C$_q$), 167.1 (C$_q$), 143.8 (C$_q$), 142.2 (C$_q$), 137.6 (C$_q$), 137.4 (CH), 136.2 (CH), 133.0 (C$_q$), 131.5 (CH), 126.4 (CH), 125.3 (CH), 122.7 (CH), 117.0 (CH), 115.5 (CH), 64.3 (CH$_2$), 64.3 (CH$_2$), 30.8 (CH$_2$), 19.2 (CH$_2$), 15.8 (CH$_3$), 15.8 (CH$_3$), 13.7 (CH$_3$). IR (ATR): 2958, 1700, 1620, 1469, 1275, 1202, 1153, 1047, 967, 796 cm^{-1}. MS (ESI) m/z (relative intensity): 247 (100) [M + Na]$^+$, 225 (5) [M + H]$^+$. HR-MS (ESI): m/z calcd. for [$C_{12}H_{16}O_2S$ + Na]$^+$ 247.0763 found 247.0771.

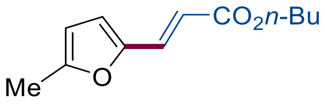

(*E*)-butyl-3-(5-methylfuran-2-yl)acrylate (*α*-30)

The general procedure **C** was followed using 2-methylfuran (**2a**) (67.0 µL, 0.75 mmol, 1.5 equiv) and *n*-butyl acrylate (**3a**) (72.0 µL, 0.50 mmol, 1.0 equiv) at 60 °C for 48 h. **L12** was used as ligand. Isolation by column chromatography (*n*-hexane/EtOAc = 20:1) yielded **30** (58.4 mg, 56%) and 18% difunctionalized product. The site selectivity was determined by HMBC NMR and the ratio of the isomers was determined by the ^{1}H-NMR of the crude mixture, *β*-olefinated product is sole product. ^{1}H-NMR (600 MHz, CDCl$_3$): δ = δ 7.34 (d, J = 15.7 Hz, 1H), 6.49 (dq, J = 3.3, 0.6 Hz, 1H), 6.22 (d, J = 15.7 Hz, 1H), 6.06 (dd, J = 3.3, 1.0 Hz, 1H), 4.17 (t, J = 6.7 Hz, 2H), 2.37 – 2.32 (m, 3H), 1.69 – 1.62 (m, 2H), 1.45 – 1.38 (m, 2H), 0.94 (t, J = 7.4 Hz, 3H). ^{13}C-NMR (125 MHz, CDCl$_3$): δ = 167.4 (C$_q$), 155.3 (C$_q$), 149.5 (C$_q$), 131.0 (CH), 116.3 (CH), 116.3 (CH), 114.0 (CH), 108.7 (CH), 64.2 (CH$_2$), 30.8 (CH$_2$), 19.2 (CH$_2$), 13.8 (CH$_3$), 13.7 (CH$_3$). IR (ATR): 2960, 2875, 1702, 1635, 1581, 1526, 1468, 1366, 1256, 1156 cm^{-1}. MS (ESI) m/z (relative intensity): 231 (100) [M + Na]$^+$, 209 (2) [M + H]$^+$. HR-MS (ESI): m/z calcd. for [$C_{12}H_{16}O_3$ + H]$^+$ 209.1172 found 209.1178.

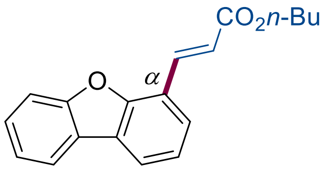

(*E*)-*n*-butyl-4-(dibenzo[*b,d*]furan-4-yl)acrylate (α-31) & (*E*)-*n*-butyl-3-(dibenzo[*b,d*]furan-3-yl)acrylate (β-31) & (*E*)-*n*-butyl-3-(dibenzo[*b,d*]furan-2-yl)acrylate (γ-31) & (*E*)-*n*-Butyl-3-(dibenzo[*b,d*]furan-1-yl)acrylate (δ-31)

The general procedure **C** was followed using dibenzo[*b*,*d*]furan (**2b**) (126.2 mg, 0.75 mmol, 1.5 equiv) and *n*-butyl acrylate (**3a**) (72.0 µL, 0.50 mmol, 1.0 equiv) at 60 °C for 48 h. **L12** was used as ligand. Isolation by column chromatography (*n*-hexane/EtOAc = 20:1 to 10:1) yielded **31** (91.3 mg, 62%) as a colorless oil. The site selectivity was determined by HMBC and the ratio of the isomers was determined by the ^{1}H-NMR of the crude mixture, α : others = 1 : 1. For α-**31**, ^{1}H-NMR (400 MHz, $CDCl_3$): δ = 8.01 – 7.94 (m, 3H), 7.67 (d, J = 8.3 Hz, 1H), 7.57 (dd, J = 7.6, 1.3 Hz, 1H), 7.51 (ddd, J = 8.4, 7.3, 1.4 Hz, 1H), 7.43 – 7.32 (m, 2H), 7.09 (d, J = 16.1 Hz, 1H), 4.28 (t, J = 6.7 Hz, 2H), 1.79 – 1.71 (m, 2H), 1.54 – 1.44 (m, 2H), 1.00 (t, J = 7.4 Hz, 3H). ^{13}C-NMR (100 MHz, $CDCl_3$): δ = 167.5 (C_q), 156.2 (C_q), 154.4 (C_q), 139.3 (CH), 128.3 (CH), 127.6 (CH), 125.1 (C_q), 123.6 (C_q), 123.2 (CH), 123.1 (CH), 122.3 (CH), 121.6 (CH), 120.8 (CH), 119.80 (C_q), 112.00 (CH), 64.58 (CH_2), 30.88 (CH_2), 19.28 (CH_2), 13.83 (CH_3). IR (ATR): 2933, 2871, 1711, 1600, 1451, 1190, 1172, 1022, 959, 749 cm^{-1}. MS (ESI) *m*/*z* (relative intensity): 317 (40) [M + Na]$^+$, 295 (100) [M + H]$^+$. HR-MS (ESI): *m*/*z* calcd. for [$C_{19}H_{18}O_3$ + H]$^+$ 295.1329 found 295.1320.

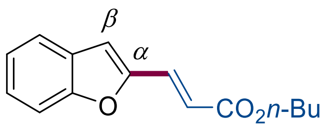

(*E*)-*n*-butyl-3-(benzofuran-2-yl)acrylate (α-32) & (*E*)-*n*-Butyl-3-(benzofuran-3-yl)acrylate (β-32)

The general procedure **C** was followed using benzofuran (**2c**) (81.2 µL, 0.75 mmol, 1.5 equiv) and *n*-butyl acrylate (**3a**) (72.0 µL, 0.50 mmol, 1.0 equiv) at 60 °C for 48 h. **L12** was used as ligand. Isolation by column chromatography (*n*-hexane/EtOAc = 30:1 to 20:1) yielded **32** (54.9 mg, 45%) as a colorless oil and 30% difunctionalized product. The α and β-olefinated products are known compounds[31] and the ratio of the isomers was determined by the ^{1}H-NMR of the crude mixture, $\beta : \alpha$ = 1 : 2. ^{1}H-NMR (400 MHz, $CDCl_3$): δ = 7.88 (s, 1H$^\beta$), 7.87 – 7.84 (m, 1H$^\beta$), 7.79 (d, J = 16.1 Hz, 1H$^\beta$), 7.58 (d, J = 7.6 Hz, 1H$^\alpha$), 7.56 – 7.52 (m, 1H$^{\alpha+\beta}$), 7.48 (dd, J = 8.3, 0.9 Hz, 1H^2), 7.39 – 7.32 (m, 1H$^\alpha$+2H$^\beta$), 7.28 – 7.19 (m, 1H$^\alpha$), 6.93 (s, 1H$^\alpha$), 6.62 – 6.53

(m, 1H$^{\alpha+\beta}$), 4.26 – 4.20 (m, 2H$^{\alpha+\beta}$), 1.74 – 1.66 (m, 2H$^{\alpha+\beta}$), 1.50 – 1.40 (m, 2H$^{\alpha+\beta}$), 1.01 – 0.94 (m, 3H$^{\alpha+\beta}$). ^{13}C-NMR (150 MHz, $CDCl_3$): δ = 167.2 (C_q), 166.7 (C_q), 156.1 (C_q), 155.5 (C_q), 152.4 (C_q), 147.7 (CH), 134.3 (CH), 131.1 (CH), 128.3 (C_q), 126.4 (CH), 125.3 (CH), 124.8 (C_q), 123.7 (CH), 123.3 (CH), 121.7 (CH), 121.0 (CH), 119.0 (CH), 118.4 (CH), 117.9 (C_q), 112.0 (CH), 111.4 (CH), 110.9 (CH), 64.6 (CH_2), 64.4 (CH_2), 30.8 (CH_2), 30.7 (CH_2), 19.2 (CH_2), 19.2 (CH_2), 13.7 (CH_3), 13.7 (CH_3). IR (ATR): 2960, 2934, 2874, 1711, 1638, 1451, 1300, 1263, 1169, 750 cm^{-1}. MS (ESI) *m*/*z* (relative intensity): 267 (100) [M + Na]$^+$, 245 (30) [M + H]$^+$. HR-MS (ESI): *m*/*z* calcd. for [$C_{15}H_{16}O_3$ + Na]$^+$ 267.0992 found 267.0995.

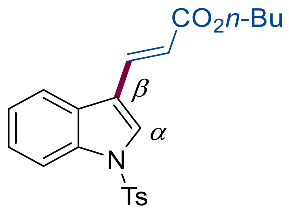

(*E*)-butyl-3-(1-tosyl-*1H*-indol-3-yl)acrylate (*α*-33)

The general procedure **C** was followed using 1-tosyl-*1H*-indole (**2d**) (203.5 mg, 0.75 mmol, 1.5 equiv) and *n*-butyl acrylate (**3a**) (72.0 µL, 0.50 mmol, 1.0 equiv) at 60 °C for 48 h. **L12** was used as ligand. Isolation by column chromatography (*n*-hexane/EtOAc = 5:1) yielded **33** (137.1 mg, 69%). The site selectivity was determined by HMBC NMR and the ratio of the isomers was determined by the ^{1}H-NMR of the reaction mixture, *β*-olefinated product is sole product. ^{1}H-NMR (300 MHz, $CDCl_3$): δ = 8.00 (dt, *J* = 8.4, 0.9 Hz, 1H), 7.84 (s, 1H), 7.82 – 7.74 (m, 4H), 7.38 (ddd, *J* = 8.4, 7.3, 1.5 Hz, 1H), 7.31 (td, *J* = 7.5, 1.3 Hz, 1H), 7.24 (d, *J* = 8.2 Hz, 2H), 6.52 (d, *J* = 16.1 Hz, 1H), 4.22 (t, *J* = 6.6 Hz, 2H), 2.34 (s, 3H), 1.76 – 1.62 (m, 2H), 1.52 – 1.36 (m, 2H), 0.97 (t, *J* = 7.3 Hz, 3H). ^{13}C-NMR (150 MHz, $CDCl_3$): δ = 167.2 (C_q), 145.5 (C_q), 135.6 (C_q), 135.5 (CH), 134.7 (C_q), 130.1 (CH), 128.3 (CH), 128.1 (C_q), 126.9 (CH), 125.4 (CH), 124.0 (CH), 120.7 (CH), 118.4 (CH), 118.2 (C_q), 113.8 (CH), 64.4 (CH_2), 30.8 (CH_2), 21.6 (CH_3), 19.2 (CH_2), 13.7 (CH_3). IR (ATR): 3126, 2963, 1711, 1635, 1445, 1364, 1162, 1124, 977, 826 cm^{-1}. MS (ESI) *m*/*z* (relative intensity): 420 (100) [M + Na]$^+$, 398 (5) [M + H]$^+$. HR-MS (ESI): *m*/*z* calcd. for [$C_{22}H_{23}NO_4S$ + Na]$^+$ 420.1240 found 420.1234.

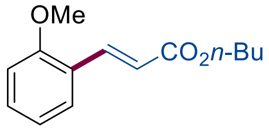

(*E*)-*n*-Butyl-3-(2-methoxyphenyl)acrylate (*o*-34) & (*E*)-*n*-butyl-3-(4-methoxyphenyl)acrylate (*p*-34)

The general procedure **A** was followed using anisole (**2e**) (108.7 µL, 1.0 mmol, 5.0 equiv) and *n*-butyl acrylate (**3a**) (28.8 µL, 0.20 mmol, 1.0 equiv) at 60 °C for 20 h. **L12** was used as ligand. Isolation by column chromatography (*n*-hexane/EtOAc = 30:1 to 15:1) yielded **34** (24.4 mg, 52%) as a colorless oil. The *o* and *p*-olefinated products are known compounds[31] and the ratio of the isomers was determined by the ^{1}H-NMR of the crude mixture, *o* : *p* = 16 : 1. ^{1}H-NMR (600 MHz, $CDCl_3$): δ^o = 7.99 (d, *J* = 16.2 Hz, 1H), 7.50 (dd, *J* = 7.7, 1.7 Hz, 1H), 7.34 (ddd, *J* = 8.3, 7.4, 1.7 Hz, 1H), 6.97 – 6.93 (m, 1H), 6.91 (dd, *J* = 8.3, 1.0 Hz, 1H), 6.53 (d, J = 16.1 Hz, 1H), 4.20 (t, *J* = 6.7 Hz, 2H), 3.88 (s, 3H), 1.74 – 1.64 (m, 2H), 1.50 – 1.37 (m, 2H), 0.96 (t, *J* = 7.4 Hz, 3H). ^{13}C-NMR (150 MHz, $CDCl_3$): δ^o = 167.6 (C_q), 158.3 (C_q), 139.9 (CH), 131.3 (CH), 128.9 (CH), 123.4 (C_q), 120.6 (CH), 118.8 (CH), 111.1 (CH), 64.2 (CH_2), 55.4 (CH_3), 30.8 (CH_2), 19.2 (CH_2), 13.7 (CH_3). IR (ATR): 3060, 2959, 2934, 2872, 1708, 1634, 1304, 1165, 978, 775 cm^{-1}. MS (ESI) *m*/*z* (relative intensity): 257 (100) $[M + Na]^+$, 235 (20) $[M + H]^+$. HR-MS (ESI): *m*/*z* calcd. for $[C_{14}H_{18}O_3 + Na]^+$ 257.1148 found 257.1152.

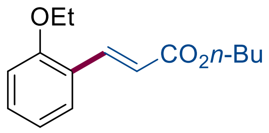

(*E*)-*n*-Butyl-3-(2-ethoxyphenyl)acrylate (35)

The general procedure **A** was followed using ethoxybenzene (**2f**) (126.3 µL, 1.0 mmol, 5.0 equiv) and *n*-butyl acrylate (**3a**) (28.8 µL, 0.20 mmol, 1.0 equiv) at 60 °C for 20 h. **L12** was used as ligand. Isolation by column chromatography (*n*-hexane/EtOAc = 30:1 to 15:1) yielded **35** (27.3 mg, 55%) as a colorless oil. The ratio of the isomers was determined by the ^{1}H-NMR of the crude mixture, *o* : *p* = 20 : 1. ^{1}H-NMR (400 MHz, $CDCl_3$): δ^o = 8.02 (d, *J* = 16.2 Hz, 1H), 7.51 (dd, *J* = 7.7, 1.8 Hz, 1H), 7.31 (ddd, *J* = 8.3, 7.4, 1.7 Hz, 1H), 6.94 (td, *J* = 7.4, 0.6 Hz, 1H), 6.89 (dd, *J* = 8.3, 1.0 Hz, 1H), 6.53 (d, *J* = 16.2 Hz, 1H), 4.21 (t, *J* = 6.7 Hz, 2H), 4.10 (q, *J* = 7.0 Hz, 2H), 1.76 – 1.62 (m, 2H), 1.51 – 1.40 (m, 5H), 0.97 (t, *J* = 7.4 Hz, 3H). ^{13}C-NMR (100 MHz, $CDCl_3$): δ = 167.7 (C_q), 157.7 (C_q), 140.1 (CH), 131.3 (CH), 128.8 (CH), 123.5 (C_q), 120.5 (CH), 118.6 (CH), 112.1 (CH), 64.2 (CH_2), 64.0 (CH_2), 30.8 (CH_2), 19.2 (CH_2), 14.8 (CH_3), 13.8 (CH_3). IR (ATR): 2960, 2933, 2252, 1702, 1631, 1317, 1173, 908, 733,

650 cm^{-1}. MS (ESI) *m*/*z* (relative intensity): 249 (40) $[M + H]^+$, 271 (100) $[M + Na]^+$. HR-MS (ESI): *m*/*z* calcd. for $[C_{15}H_{20}O_3 + Na]^+$ 271.1305 found 271.1299.

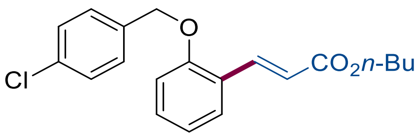

(*E*)-*n*-Butyl-3-(2-((4-chlorobenzyl)oxy)phenyl)acrylate (36)

The general procedure **A** was followed using 1-chloro-4-(phenoxymethyl)benzene (**2g**) (218.7 mg, 1.0 mmol, 5.0 equiv) and *n*-butyl acrylate (**3a**) (28.8 µL, 0.20 mmol, 1.0 equiv) at 60 °C for 24 h. **L12** was used as ligand. Isolation by column chromatography (*n*-hexane/EtOAc = 30:1 to 15:1) yielded **36** (36.2 mg, 52%) as a colorless oil. The ratio of the isomers was determined by the ^{1}H-NMR of the crude mixture, *o* : *p* = 9 : 1. ^{1}H-NMR (400 MHz, $CDCl_3$): δ^o = 8.06 (d, *J* = 16.2 Hz, 1H), 7.55 (dd, *J* = 7.7, 1.7 Hz, 1H), 7.38 – 7.35 (m, 4H), 7.31 (ddd, *J* = 8.3, 7.4, 1.7 Hz, 1H), 6.98 (td, *J* = 7.7, 1.1 Hz, 1H), 6.92 (dd, *J* = 8.4, 1.0 Hz, 1H), 6.52 (d, *J* = 16.1 Hz, 1H), 5.12 (s, 2H), 4.20 (t, *J* = 6.6 Hz, 2H), 1.74 – 1.64 (m, 2H), 1.50 – 1.38 (m, 2H), 0.97 (t, *J* = 7.4 Hz, 3H). ^{13}C-NMR (100 MHz, $CDCl_3$): δ = 167.4 (C_q), 157.0 (C_q), 139.5 (CH), 135.1 (C_q), 133.8 (C_q), 131.3 (CH), 128.8 (CH), 128.7 (CH), 128.5 (CH), 123.9 (C_q), 121.2 (CH), 119.0 (CH), 112.6 (CH), 69.6 (CH_2), 64.2 (CH_2), 30.8 (CH_2), 19.2 (CH_2), 13.7 (CH_3). IR (ATR): 2960, 2933, 2252, 1703, 1632, 1599, 1271, 1172, 909, 736 cm^{-1}. MS (ESI) *m*/*z* (relative intensity): 345 (40) $[M + H]^+$, 367 (100) $[M + Na]^+$. HR-MS (ESI): *m*/*z* calcd. for $[C_{20}H_{21}O_3Cl + Na]^+$ 367.1071 found 367.1073.

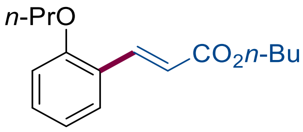

(*E*)-*n*-Butyl-3-(2-propoxyphenyl)acrylate (37)

The general procedure **A** was followed using propoxybenzene (**2h**) (140.5 µL, 1.0 mmol, 5.0 equiv) and *n*-butyl acrylate (**3a**) (28.8 µL, 0.20 mmol, 1.0 equiv) at 60 °C for 20 h. **L12** was used as ligand. Isolation by column chromatography (*n*-hexane/EtOAc = 30:1 to 15:1) yielded **37** (21.6 mg, 41%) as a colorless oil. The ratio of the isomers was determined by the ^{1}H-NMR of the crude mixture, *o* : *p* = 20 : 1. ^{1}H-NMR (600 MHz, $CDCl_3$): δ^o = 8.02 (d, *J* = 16.2 Hz, 1H), 7.51 (dd, *J* = 7.7, 1.7 Hz, 1H), 7.31 (ddd, *J* = 8.3, 7.3, 1.7 Hz, 1H), 6.94 (td, *J* = 7.5, 1.0

Hz, 1H), 6.90 (dd, J = 8.3, 1.0 Hz, 1H), 6.53 (d, J = 16.1 Hz, 1H), 4.20 (t, J = 6.7 Hz, 2H), 3.99 (t, J = 6.5 Hz, 2H), 1.93 – 1.83 (m, 2H), 1.73 – 1.65 (m, 2H), 1.50 – 1.39 (m, 2H), 1.08 (t, J = 7.5 Hz, 3H), 0.97 (t, J = 7.4 Hz, 3H). ^{13}C-NMR (150 MHz, $CDCl_3$): δ = 167.6 (C_q), 157.8 (C_q), 140.0 (CH), 131.3 (CH), 128.7 (CH), 123.5 (C_q), 120.4 (CH), 118.5 (CH), 112.0 (CH), 69.9 (CH_2), 64.1 (CH_2), 30.8 (CH_2), 22.5 (CH_2), 19.2 (CH_2), 13.7 (CH_3), 10.6 (CH_3). IR (ATR): 2963, 2876, 2252, 1702, 1631, 1270, 1173, 906, 729, 649 cm^{-1}. MS (ESI) m/z (relative intensity): 263 (10) $[M + H]^+$, 285 (100) $[M + Na]^+$. HR-MS (ESI): m/z calcd. for $[C_{16}H_{22}O_3 + Na]^+$ 285.1461 found 285.1460.

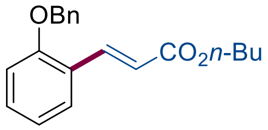

(*E*)-*n*-Butyl-3-(2-(benzyloxy)phenyl)acrylate (38)

The general procedure **A** was followed using (benzyloxy)benzene (**2i**) (184.2 µL, 1.0 mmol, 5.0 equiv) and *n*-butyl acrylate (**3a**) (28.8 µL, 0.20 mmol, 1.0 equiv) at 60 °C for 20 h. **L12** was used as ligand. Isolation by column chromatography (*n*-hexane/EtOAc = 30:1 to 15:1) yielded **38** (28.5 mg, 46%) as a colorless oil. **38** is a known compound.[40] The ratio of the isomers was determined by the ^{1}H-NMR of the crude mixture, o : p = 20 : 1. ^{1}H-NMR (400 MHz, $CDCl_3$): δ^o = 8.09 (d, J = 16.2 Hz, 1H), 7.55 (dd, J = 7.7, 1.7 Hz, 1H), 7.47 – 7.27 (m, 6H), 7.01 – 6.93 (m, 2H), 6.54 (d, J = 16.1 Hz, 1H), 5.17 (s, 2H), 4.20 (t, J = 6.6 Hz, 2H), 1.76 – 1.62 (m, 2H), 1.51 – 1.38 (m, 2H), 0.97 (t, J = 7.4 Hz, 3H). ^{13}C-NMR (100 MHz, $CDCl_3$): δ = 167.6 (C_q), 157.4 (C_q), 139.8 (CH), 136.7 (C_q), 131.4 (CH), 128.7 (CH), 128.7 (CH), 128.0 (CH), 127.2 (CH), 124.0 (C_q), 121.1 (CH), 119.0 (CH), 112.8 (CH), 70.4 (CH_2), 64.3 (CH_2), 30.8 (CH_2), 19.3 (CH_2), 13.8 (CH_3). IR (ATR): 2960, 2873, 2253, 1702, 1632, 1272, 1173, 905, 729, 649 cm^{-1}. MS (ESI) m/z (relative intensity): 311 (10) $[M + H]^+$, 333 (100) $[M + Na]^+$. HR-MS (ESI): m/z calcd. for $[C_{20}H_{22}O_3 + Na]^+$ 333.1461 found 333.1467.

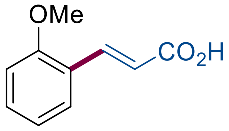

(*E*)-3-(2-Methoxyphenyl)acrylic acid (39)

The general procedure **A** was followed using anisole (**2e**) (108.6 µL, 1.0 mmol, 5equiv) and acrylic acid (**3b**) (13.7 µL, 0.20 mmol, 1.0 equiv) at 60 °C for 20 h. **L12** was used as ligand. Isolation by column chromatography (*n*-hexane/EtOAc = 30:1 to 5:1) yielded **39** (21.0 mg, 59%) as a white solid. The product is known compound.[41] The ratio of the isomers was determined by the ^{1}H-NMR of the crude mixture, *o* : *p* > 20 : 1. ^{1}H-NMR (400 MHz, $CDCl_3$): δ = 8.10 (d, *J* = 16.1 Hz, 1H), 7.58 – 7.50 (m, 1H), 7.42 – 7.34 (m, 1H), 6.98 (td, *J* = 7.6, 1.1 Hz, 1H), 6.93 (dd, *J* = 8.3, 1.0 Hz, 1H), 6.55 (d, *J* = 16.1 Hz, 1H), 3.90 (s, 3H). ^{13}C-NMR (100 MHz, $CDCl_3$): δ = 172.6 (C_q), 158.6 (C_q), 142.5 (CH), 132.0 (CH), 129.3 (CH), 123.0 (C_q), 120.7 (CH), 117.6 (CH), 111.2 (CH), 55.5 (CH_3). IR (ATR): 2970, 2936, 2839, 1686, 1599, 1488, 1464, 1248, 1163, 1026 cm^{-1}. MS (ESI) *m*/*z* (relative intensity): 201 (30) $[M + Na]^+$. HR-MS (ESI): *m*/*z* calcd. for $[C_{10}H_{10}O_3 + Na]^+$ 201.0522 found 201.0519.

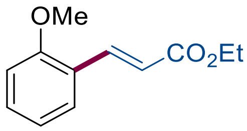

(*E*)-Ethyl-3-(2-methoxyphenyl)acrylate (*o*-40) & (*E*)-ethyl-3-(4-methoxyphenyl)acrylate (*p*-40)

The general procedure **A** was followed using anisole (**2e**) (108.6 µL, 1.0 mmol, 5.0 equiv) and ethyl acrylate (**3c**) (21.7 µL, 0.20 mmol, 1.0 equiv). **L12** was used as ligand. Isolation by column chromatography (*n*-hexane/EtOAc = 30:1 to 15:1) yielded **40** (22.2 mg, 54%) as a colorless oil. The *o* and *p*-olefinated products are known compounds[42] and the ratio of the isomers was determined by the ^{1}H-NMR of the crude mixture, *o* : *p* = 12 : 1. ^{1}H-NMR (400 MHz, $CDCl_3$): δ^{o} = 7.99 (d, *J* = 16.2 Hz, 1H), 7.50 (dd, *J* = 7.6, 1.8 Hz, 1H), 7.34 (ddd, *J* = 8.2, 7.4, 1.7 Hz, 1H), 7.00 – 6.91 (m, 1H), 6.91 (dd, *J* = 8.4, 1.0 Hz, 1H), 6.53 (d, *J* = 16.1 Hz, 1H), 4.26 (q, *J* = 7.1 Hz, 2H), 3.89 (s, 3H), 1.34 (t, *J* = 7.1 Hz, 3H). ^{13}C-NMR (100 MHz, $CDCl_3$): δ = 167.5 (C_q), 158.3 (C_q), 140.0 (CH), 131.3 (CH), 129.6 (CH), 128.9 (CH), 123.4 (C_q), 120.6 (CH), 118.8 (CH), 114.3 (CH), 111.1 (CH), 60.3 (CH_2), 55.4 (CH_3), 14.3 (CH_3). IR (ATR): 2976, 2938, 2839, 1709, 1632, 1465, 1249, 1172, 1029, 753 cm^{-1}. MS (ESI) *m*/*z* (relative intensity): 229 (100) $[M + Na]^+$, 207 (10) $[M + H]^+$. HR-MS (ESI): *m*/*z* calcd. for $[C_{12}H_{14}O_3 + Na]^+$ 229.0835 found 229.0831.

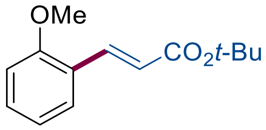

(*E*)-*t*-Butyl-3-(2-methoxyphenyl)acrylate (*o*-41) & (*E*)-*t*-butyl-3-(4-methoxyphenyl)acrylate (*p*-41)

The general procedure **A** was followed using anisole (**2e**) (108.6 µL, 1.0 mmol, 5.0 equiv) and *tert*-butyl acrylate (**3d**) (29.1 µL, 0.20 mmol, 1.0 equiv) at 60 °C for 20 h. **L12** was used as ligand. Isolation by column chromatography (*n*-hexane/EtOAc = 30:1 to 15:1) yielded **41** (23.4 mg, 50%) as a colorless oil. The *o* and *p*-olefinated products are known compounds[43] and the ratio of the isomers was determined by the ^{1}H-NMR of the crude mixture, *o* : *p* = >20 : 1. ^{1}H-NMR (600 MHz, $CDCl_3$): δ^{o} = 7.91 (d, *J* = 16.1 Hz, 1H), 7.49 (dd, *J* = 7.7, 1.7 Hz, 1H), 7.32 (ddd, *J* = 8.3, 7.4, 1.7 Hz, 1H), 6.97 – 6.92 (m, 1H), 6.90 (dd, *J* = 8.4, 1.0 Hz, 1H), 6.44 (d, *J* = 16.1 Hz, 1H), 3.87 (s, 3H), 1.53 (s, 9H). ^{13}C-NMR (150 MHz, $CDCl_3$): δ^{o} = 166.8 (C_q), 158.2 (C_q), 138.9 (CH), 131.1 (CH), 128.7 (CH), 123.6 (C_q), 120.6 (CH), 120.6 (CH), 111.0 (CH), 80.2 (C_q), 55.4 (CH_3), 28.2 (CH_3). IR (ATR): 3003, 2977, 2930, 2839, 1704, 1598, 1489, 1322, 1148, 987 cm^{-1}. MS (ESI) *m*/*z* (relative intensity): 257 (100) $[M + Na]^+$. HR-MS (ESI): *m*/*z* calcd. for $[C_{14}H_{18}O_3 + Na]^+$ 257.1148 found 257.1141.

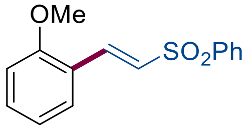

(*E*)-1-Methoxy-2-(2-(phenylsulfonyl)vinyl)benzene (42)

The general procedure **A** was followed using anisole (**2e**) (108.6 µL, 1.0 mmol, 5.0 equiv) and (vinylsulfonyl)benzene (**3e**) (33.6 mg, 0.20 mmol, 1.0 equiv) at 60 °C for 20 h. **L12** was used as ligand. Isolation by column chromatography (*n*-hexane/EtOAc = 5:1) yielded **42** (25.8 mg, 47%) as a colorless oil. The product is known compound.[44] The ratio of the isomers was determined by the ^{1}H-NMR of the crude mixture, *o* : *p* > 20 : 1. ^{1}H-NMR (400 MHz, $CDCl_3$): δ = 7.95 (d, *J* = 6.9 Hz, 2H), 7.89 (d, *J* = 15.4 Hz, 1H), 7.63 – 7.57 (m, 1H), 7.56 – 7.49 (m, 2H), 7.44 – 7.34 (m, 2H), 7.08 (d, *J* = 15.5 Hz, 1H), 6.96 (td, *J* = 7.5, 1.1 Hz, 1H), 6.92 (dd, *J* = 8.4, 1.0 Hz, 1H), 3.88 (s, 3H). ^{13}C-NMR (100 MHz, $CDCl_3$): δ = 158.8 (C_q), 141.2 (C_q), 138.5 (CH), 133.1 (CH), 132.5 (CH), 130.8 (CH), 129.2 (CH), 127.8 (CH), 127.6 (CH), 121.2

(C_q), 120.8 (CH), 111.2 (CH), 55.5 (CH_3). IR (ATR): 3062, 2973, 2944, 2843, 1597, 1483, 1306, 1249, 1141, 1081 cm^{-1}. MS (ESI) *m*/*z* (relative intensity): 297 (100) $[M + Na]^+$, 275 (60) $[M + H]^+$. HR-MS (ESI): *m*/*z* calcd. for $[C_{15}H_{14}O_3S + Na]^+$ 297.0556 found 297.0553.

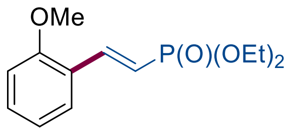

(*E*)-Diethyl-(2-methoxystyryl)phosphonate (43)

The general procedure **A** was followed using anisole (**2e**) (108.6 µL, 1.0 mmol, 5.0 equiv) and diethyl vinylphosphonate (**3f**) (30.7 µL, 0.20 mmol, 1.0 equiv) at 60 °C for 20 h. **L12** was used as ligand. Isolation by column chromatography (*n*-hexane/EtOAc = 1:1) yielded **43** (24.9 mg, 46%) as a colorless oil. The product is known compound.[45] The ratio of the isomers was determined by the ^{1}H-NMR of the crude mixture, *o* : *p* > 20 : 1. ^{1}H-NMR (400 MHz, $CDCl_3$): δ = 7.80 (dd, *J* = 23.6, 17.7 Hz, 1H), 7.47 (dd, *J* = 7.7, 1.7 Hz, 1H), 7.32 (ddd, *J* = 8.8, 7.3, 1.7 Hz, 1H), 6.94 (td, *J* = 7.5, 1.1 Hz, 1H), 6.89 (dd, *J* = 8.3, 1.0 Hz, 1H), 6.31 (dd, *J* = 19.6, 17.7 Hz, 1H), 4.17 – 4.06 (m, 4H), 3.85 (s, 3H), 1.34 (t, *J* = 7.1 Hz, 6H). ^{13}C-NMR (100 MHz, $CDCl_3$): δ = 158.0 (C_q), 144.0 (d, *J* = 7.8 Hz, CH), 131.3 (CH), 128.4 (CH), 123.7 (d, *J* = 23.3 Hz, C_q), 120.5 (CH), 114.2 (d, *J* = 190 Hz, CH), 111.1 (CH), 61.7 (s, CH_2), 61.6 (s, CH_2), 55.4 (CH_3), 16.4 (s, CH_3), 16.3 (s, CH_3). ^{31}P-NMR (162 MHz, $CDCl_3$): δ = 20.5. IR (ATR): 2981, 2938, 2904, 1598, 1487, 1464, 1292, 1243, 1163, 1018 cm^{-1}. MS (ESI) *m*/*z* (relative intensity): 271 (100) $[M + H]^+$, 293 (30) $[M + Na]^+$. HR-MS (ESI): *m*/*z* calcd. for $[C_{13}H_{19}O_4P + H]^+$ 271.1094 found 271.1096.

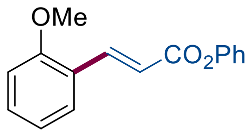

(*E*)-Phenyl-3-(2-methoxyphenyl)acrylate (*o*-44) & (*E*)-phenyl-3-(4-methoxyphenyl)acrylate (*p*-44)

The general procedure **C** was followed using anisole (**2e**) (108.6 µL, 1.0 mmol, 2.0 equiv) and phenyl acrylate (**3g**) (68.8 µL, 0.50 mmol, 1.0 equiv) at 60 °C for 48 h. **L12** was used as ligand and Pt was used as anode. Isolation by column chromatography (*n*-hexane/EtOAc = 30:1) yielded **44** (63.4 mg, 50%) as a mixture. The *p*-olefinated product is known compound[46] and

the ratio of the isomers was determined by the ^{1}H-NMR of the crude mixture, *o* : *p* = 8 : 1. ^{1}H-NMR (600 MHz, $CDCl_3$): δ = 8.18 (d, *J* = 16.1 Hz, 1H), 7.57 (dd, *J* = 7.7, 1.7 Hz, 1H), 7.42 – 7.38 (m, 3H), 7.26 – 7.23 (m, 1H), 7.19 – 7.17 (m, 2H), 7.00 (td, *J* = 7.5, 1.1 Hz, 1H), 6.95 (dd, *J* = 8.4, 1.0 Hz, 1H), 6.74 (d, *J* = 16.1 Hz, 1H), 3.92 (s, 3H). ^{13}C-NMR (126 MHz, $CDCl_3$): δ = 165.9 (C_q), 158.6 (C_q), 150.9 (C_q), 142.0 (CH), 131.9 (CH), 129.4 (CH), 129.3 (CH), 125.6 (C_q), 123.2 (CH), 121.7 (CH), 120.8 (CH), 117.8 (CH), 111.2 (CH), 55.5 (CH_3). IR (ATR): 3072, 2937, 2838, 1720, 1630, 1598, 1488, 1309, 1194, 1134 cm^{-1}. MS (ESI) *m*/*z* (relative intensity): 277 (100) [M + Na]$^+$, 255 (64) [M + H]$^+$. HR-MS (ESI): *m*/*z* calcd. for [$C_{16}H_{14}O_3$ + Na]$^+$ 277.0835 found 277.0833.

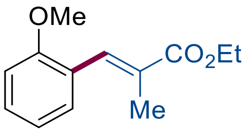

(*E*)-Ethyl-3-(2-methoxyphenyl)-2-methylacrylate (*o*-45) & (*E*)-ethyl-3-(4-methoxyphenyl)-2-methylacrylate (*p*-45)

The general procedure **A** was followed using anisole (**2e**) (108.6 µL, 1.0 mmol, 5.0 equiv) and ethyl methacrylate (**3h**) (24.8 µL, 0.20 mmol, 1.0 equiv) at 60 °C for 20 h. **L12** was used as ligand. Isolation by column chromatography (*n*-hexane/EtOAc = 15:1 to 10:1) yielded **45** (18.9 mg, 43%) as a yellow oil. The ratio of the isomers was determined by the ^{1}H-NMR of the crude mixture, *o* : *p* = 7 : 1. ^{1}H-NMR (400 MHz, $CDCl_3$): δ = 7.83 (s, $1H^{o}$), 7.64 (d, *J* = 1.6 Hz, $1H^{p}$), 7.42 – 7.27 (m, $2H^{o+p}$), 7.01 – 6.87 (m, $2H^{o+p}$), 4.31 – 4.23 (m, $2H^{o+p}$), 3.86 (s, $3H^{o}$), 3.84 (s, $3H^{p}$), 2.13 (d, *J* = 1.5 Hz, $3H^{p}$), 2.05 (d, *J* = 1.5 Hz, $3H^{o}$), 1.40 – 1.31 (m, $3H^{o+p}$). ^{13}C-NMR (100 MHz, $CDCl_3$): δ = 168.6 (C_q), 157.5 (C_q), 134.6 (CH), 130.2 (CH), 129.7 (CH), 128.7 (C_q), 124.9 (C_q), 120.0 (CH), 110.4 (CH), 60.7 (CH_2), 55.4 (CH_3), 14.3 (CH_3), 14.2 (CH_3). IR (ATR): 2980, 2935, 2837, 1703, 1597, 1487, 1366, 1241, 1104, 1027 cm^{-1}. MS (ESI) *m*/*z* (relative intensity): 243 (100) [M + Na]$^+$, 221 (30) [M + H]$^+$. HR-MS (ESI): *m*/*z* calcd. for [$C_{13}H_{16}O_3$ + Na]$^+$ 243.0992 found 243.0989.

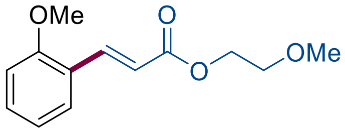

(*E*)-2-Methoxyethyl-3-(2-methoxyphenyl)acrylate (46)

The general procedure **A** was followed using anisole (**2e**) (108.6 µL, 1.0 mmol, 5.0 equiv) and 2-methoxyethyl acrylate (**3i**) (25.7 µL, 0.20 mmol, 1.0 equiv) at 60 °C for 20 h. **L12** was used as ligand. Isolation by column chromatography (*n*-hexane/EtOAc = 50:1 to 10:1) yielded **46** (31.2 mg, 66%) as a colorless oil. The ratio of the isomers was determined by the crude ^{1}H-NMR, *o* : *p* = 5 : 1. The *o*-product was isolated. ^{1}H-NMR (600 MHz, $CDCl_3$): δ^o = 8.01 (d, *J* = 16.1 Hz, 1H), 7.49 (dd, *J* = 7.7, 1.8 Hz, 1H), 7.34 (ddd, *J* = 8.3, 7.4, 1.7 Hz, 1H), 6.96 – 6.93 (m, 1H), 6.90 (dd, *J* = 8.4, 1.0 Hz, 1H), 6.59 (d, *J* = 16.1 Hz, 1H), 4.41 – 4.31 (m, 2H), 3.87 (s, 3H), 3.69 – 3.63 (m, 2H), 3.42 (s, 3H). ^{13}C-NMR (150 MHz, $CDCl_3$): δ^o = 167.5 (C_q), 158.4 (C_q), 140.6 (CH), 131.5 (CH), 129.1 (CH), 123.3 (C_q), 120.7 (CH), 118.3 (CH), 111.1 (CH), 70.6 (CH_2), 63.4 (CH_2), 59.0 (CH_3), 55.4 (CH_3). IR (ATR): 2939, 2887, 2838, 1708, 1630, 1598, 1438, 1267, 1161, 1124 cm^{-1}. MS (ESI) *m*/*z* (relative intensity): 259 (100) $[M + Na]^+$. HR-MS (ESI): *m*/*z* calcd. for $[C_{13}H_{16}O_4 + Na]^+$ 259.0941 found 259.0938.

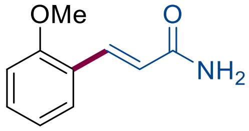

(*E*)-3-(2-methoxyphenyl)acrylamide (47)

The general procedure **A** was followed using anisole (**2e**) (108.6 µL, 1.0 mmol, 5.0 equiv) and acrylamide (**3j**) (14.2 mg, 0.20 mmol, 1.0 equiv) at 60 °C for 20 h. **L12** was used as ligand and Pt was used as anode. Isolation by column chromatography (DCM/MeOH = 10:1) yielded **47** (15.1 mg, 43%) as a white solid. The *o* products are known compounds[47] and the ratio of the isomers was determined by the ^{1}H-NMR of the crude mixture, *o* : *p* = 20 : 1. ^{1}H-NMR (600 MHz, $(CD_3)_2CO$): δ = 7.86 (d, *J* = 15.9 Hz, 1H), 7.55 (ddt, *J* = 7.7, 1.9, 0.5 Hz, 1H), 7.34 (ddd, *J* = 8.3, 7.3, 1.7 Hz, 1H), 7.05 (dd, *J* = 8.3, 1.1 Hz, 1H), 6.99 – 6.92 (m, 2H), 6.74 (d, *J* = 15.9 Hz, 1H), 6.36 (brs, 1H), 3.89 (s, 3H). ^{13}C-NMR (125 MHz, $(CD_3)_2CO$): δ = 167.1 (C_q), 158.1 (C_q), 135.2 (CH), 130.7 (CH), 127.9 (CH), 123.9 (C_q), 121.9 (CH), 120.5 (CH), 111.3 (CH), 55.0 (CH_3). IR (ATR): 3372, 3178, 1656, 1602, 1489, 1465, 1401, 1248, 1109, 977 cm^{-1}. MS

(ESI) *m/z* (relative intensity): 200 (100) [M + Na]$^+$, 178 (50) [M + H]$^+$. HR-MS (ESI): *m/z* calcd. for [$C_{10}H_{11}NO_2$ + Na]$^+$ 200.0682 found 200.0680.

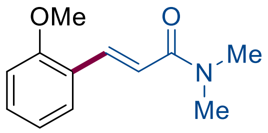

(*E*)-3-(2-methoxyphenyl)-*N*,*N*-dimethylacrylamide (48)

The general procedure **A** was followed using anisole (**2e**) (108.6 µL, 1.0 mmol, 5.0 equiv) and *N*,*N*-dimethylacrylamide (**3k**) (19.8 mg, 0.20 mmol, 1.0 equiv) at 60 °C for 20 h. **L12** was used as ligand. Isolation by column chromatography (*n*-hexane/EtOAc = 1:1) yielded **48** (16.5 mg, 40%) as a colorless oil. The *o* products are known compounds and the ratio of the isomers was determined by the ^{1}H-NMR of the crude mixture, *o* : *p* = 20 : 1. ^{1}H-NMR (600 MHz, $CDCl_3$): δ = 7.91 (d, *J* = 15.6 Hz, 1H), 7.49 (dd, *J* = 7.6, 1.8 Hz, 1H), 7.31 (ddd, *J* = 8.3, 7.4, 1.8 Hz, 1H), 7.00 (d, *J* = 15.6 Hz, 1H), 6.95 (td, *J* = 7.4, 1.1 Hz, 1H), 6.91 (dd, *J* = 8.3, 1.1 Hz, 1H), 3.87 (s, 3H), 3.16 (s, 3H), 3.06 (s, 3H). ^{13}C-NMR (125 MHz, $CDCl_3$): δ = 167.3 (C_q), 158.2 (C_q), 137.8 (CH), 130.5 (CH), 129.0 (CH), 124.5 (C_q), 120.6 (CH), 118.4 (CH), 111.1 (CH), 55.4 (CH_3), 37.4 (CH_3), 35.9 (CH_3). IR (ATR): 2936, 2838, 1740, 1646, 1489, 1462, 1391, 1244, 1137, 1024 cm^{-1}. MS (ESI) *m/z* (relative intensity): 228 (100) [M + Na]$^+$, 205 (80) [M + H]$^+$. HR-MS (ESI): *m/z* calcd. for [$C_{12}H_{15}NO_2$ + Na]$^+$ 228.0995 found 228.0996.

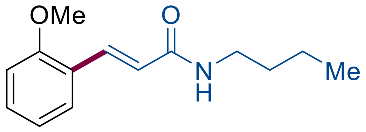

(*E*)-*n*-butyl-3-(2-methoxyphenyl)acrylamide (49)

The general procedure **A** was followed using anisole (**2e**) (108.6 µL, 1.0 mmol, 5.0 equiv) and *N*-butylacrylamide (**3l**) (29.0 µL, 0.20 mmol, 1.0 equiv) at 60 °C for 20 h. **L12** was used as ligand. Isolation by column chromatography (*n*-hexane/EtOAc = 5:1) yielded **49** (22.0 mg, 47%) as a colorless oil. The *o* and *p*-olefinated products are known compounds[48,49] and the ratio of the isomers was determined by the ^{1}H-NMR of the crude mixture, *o* : *p* = 10 : 1. ^{1}H-NMR (600 MHz, $CDCl_3$): δ = 7.85 (d, *J* = 15.8 Hz, 1H), 7.46 (dd, *J* = 7.6, 1.7 Hz, 1H), 7.31 (ddd, *J* = 8.3, 7.4, 1.7 Hz, 1H), 6.94 (td, *J* = 7.5, 1.1 Hz, 1H), 6.91 (dd, *J* = 8.3, 1.1 Hz, 1H), 6.50 (d, *J* = 15.8 Hz, 1H), 3.88 (s, 3H), 3.39 (td, *J* = 7.2, 5.8 Hz, 2H), 1.58 – 1.51 (m, 2H), 1.45

– 1.36 (m, 2H), 0.95 (t, J = 7.3 Hz, 3H). ^{13}C-NMR (125 MHz, $CDCl_3$): δ = 166.5 (C_q), 158.2 (C_q), 136.3 (CH), 130.6 (CH), 129.1 (CH), 123.9 (C_q), 121.8 (CH), 120.6 (CH), 111.1 (CH), 55.4 (CH_2), 39.4 (CH_2), 31.8 (CH_2), 20.1 (CH_2), 13.8 (CH_3). IR (ATR): 3280, 3074, 2958, 2932, 2872, 1652, 1614, 1550, 1488, 1247 cm^{-1}. MS (ESI) m/z (relative intensity): 256 (100) $[M + Na]^+$, 234 (60) $[M + H]^+$. HR-MS (ESI): m/z calcd. for $[C_{14}H_{18}NO_2 + Na]^+$ 256.1308 found 256.1310.

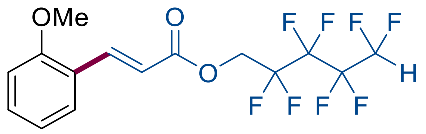

(*E*)-2,2,3,3,4,4,5,5-Octafluoropentyl-3-(2-methoxyphenyl)acrylate (*o*-50) & (*E*)-2,2,3,3,4,4,5,5-octafluoropentyl-3-(4-methoxyphenyl)acrylate (*p*-50)

The general procedure **A** was followed using anisole (**2e**) (108.6 µL, 1.0 mmol 5.0 equiv) and 2,2,3,3,4,4,5,5-octafluoropentyl acrylate (**3m**) (40.7 µL, 0.20 mmol, 1.0 equiv) at 80 °C for 24 h. **L12** was used as ligand. Isolation by column chromatography (*n*-hexane/EtOAc = 30:1 to 15:1) yielded **50** (50.2 mg, 64%) as a white solid. The site selectivity was determined by NOESY NMR and the ratio of the isomers was determined by the ^{1}H-NMR of the reaction mixture, $o : p$ = 5 : 1. ^{1}H-NMR (400 MHz, $CDCl_3$): δ = 8.09 (d, J = 16.2 Hz, $1H^o$), 7.74 (d, J = 16.0 Hz, $1H^p$), 7.56 – 7.47 (m, $1H^o$+$2H^p$), 7.38 (ddd, J = 8.3, 7.4, 1.7 Hz, $1H^o$), 7.03 – 6.88 (m, $2H^{o+p}$), 6.58 (d, J = 16.1 Hz, $1H^o$), 6.34 (d, J = 15.9 Hz, $1H^p$), 6.07 (tt, J = 51.9, 5.4 Hz, $1H^{o+p}$), 4.74 – 4.64 (m, $2H^{o+p}$), 3.90 (s, $3H^o$), 3.85 (s, $3H^p$). ^{13}C-NMR (100 MHz, $CDCl_3$): δ = 165.8 (C_q), 165.5 (C_q), 161.9 (C_q), 158.6 (C_q), 146.9 (CH), 142.8 (CH), 132.1 (CH), 130.1 (CH), 129.4 (CH), 126.6 (C_q), 122.8 (C_q), 120.7 (CH), 116.2 (CH), 114.7 (t, J = 31.0 Hz, C_q), 114.4 (CH), 113.2 (CH), 111.2 (CH), 110.29 – 109.63 (m, C_q), 107.58 (t, J = 30.8 Hz, C_q), 105.06 (t, J = 31.2 Hz, C_q), 59.34 (t, J = 26.6 Hz, CH_2), 55.5 (CH_3), 55.4 (CH_3). ^{19}F-NMR (282 MHz, $CDCl_3$): δ = -119.35 – -120.70 (m), -125.22 – -126.43 (m), -130.06 – -130.94 (m), -137.52 – -138.99 (m). IR (ATR): 3016, 2968, 2843, 1731, 1630, 1601, 1490, 1251, 1169, 1028 cm^{-1}. MS (ESI) m/z (relative intensity): 415 (100) $[M + Na]^+$, 393 (30) $[M + H]^+$. HR-MS (ESI): m/z calcd. for $[C_{15}H_{12}O_3F_8 + Na]^+$ 415.0551 found 415.0556.

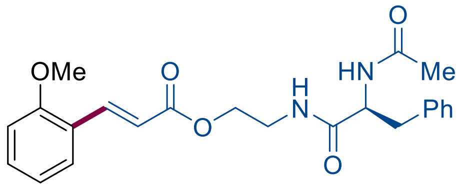

(*S*,*E*)-2-(2-Acetamido-3-phenylpropanamido)ethyl-3-(2-methoxyphenyl)acrylate (*o*-51) & (*S*,*E*)-2-(2-acetamido-3-phenylpropanamido)ethyl-3-(4-methoxyphenyl)acrylate (*p*-51)

The general procedure **B** was followed using anisole (**2e**) (108.6 µL, 1.0 mmol, 5.0 equiv) and (*S*)-2-(2-acetamido-3-phenylpropanamido)ethyl acrylate (**3n**) (60.9 mg, 0.20 mmol, 1.0 equiv) at 60 °C for 24 h. **L12** was used as ligand. Isolation by column chromatography (DCM/MeOH = 20:1) yielded **51** (43.5 mg, 53%) as a white solid. The site selectivity was determined by ^{1}H-NMR and the ratio of the isomers was determined by the ^{1}H-NMR of the crude mixture, *o* : *p* = 12 : 1. ^{1}H-NMR (400 MHz, $CDCl_3$): δ^{o} = 7.96 (d, *J* = 16.1 Hz, 1H), 7.49 (dd, *J* = 7.7, 1.8 Hz, 1H), 7.35 (ddd, *J* = 8.4, 7.4, 1.7 Hz, 1H), 7.29 – 7.22 (m, 2H), 7.22 – 7.16 (m, 3H), 6.96 (td, *J* = 7.5, 1.0 Hz, 1H), 6.91 (dd, *J* = 8.5, 1.0 Hz, 1H), 6.55 (d, *J* = 8.0 Hz, 1H), 6.50 – 6.39 (m, 1H), 4.68 (td, *J* = 8.0, 6.3 Hz, 1H), 4.21 – 4.01 (m, 2H), 3.87 (s, 3H), 3.59 – 3.35 (m, 2H), 3.13 – 2.97 (m, 2H), 1.96 (s, 3H). ^{13}C-NMR (100 MHz, $CDCl_3$): δ^{o} = 171.1 (C_q), 170.0 (C_q), 167.3 (C_q), 158.3 (C_q), 140.9 (CH), 136.6 (C_q), 131.7 (CH), 129.1 (CH), 129.0 (CH), 128.6 (CH), 127.0 (CH), 123.1 (C_q), 120.7 (CH), 117.8 (CH), 111.1 (CH), 62.7 (CH_2), 55.4 (CH_3), 54.6 (CH), 38.7 (CH_2), 38.6 (CH_2), 23.1 (CH_3). IR (ATR): 3280, 3070, 3030, 2925, 2837, 1711, 1320, 1247, 1159, 698 cm^{-1}. MS (ESI) *m*/*z* (relative intensity): 433 (100) $[M + Na]^+$, 411 (40) $[M + H]^+$. HR-MS (ESI): *m*/*z* calcd. for $[C_{23}H_{26}N_2O_5 + Na]^+$ 433.1734 found 433.1715.

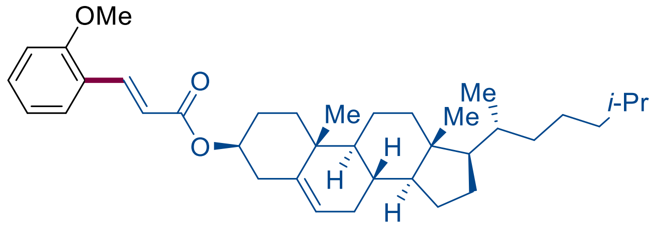

(3*S*,8*S*,9*S*,10*R*,13*R*,14*S*,17*R*)-10,13-Dimethyl-17-[(*R*)-6-methylheptan-2-yl]-2,3,4,7,8,9,10,11,12,13,14,15,16,17-tetradecahydro-1H-cyclopenta[*a*]phenanthren-3-yl (*E*)-3-(2-methoxyphenyl)acrylate (*o*-52) & (3*S*,8*S*,9*S*,10*R*,13*R*,14*S*,17*R*)-10,13-dimethyl-

17-[(*R*)-6-methylheptan-2-yl]-2,3,4,7,8,9,10,11,12,13,14,15,16,17-tetradecahydro-1H-cyclopenta[*a*]phenanthren-3-yl (*E*)-3-(4-methoxyphenyl)acrylate (*p*-52)

The general procedure **A** was followed using anisole (**2e**) (108.6 µL, 1.0 mmol, 5.0 equiv) and (3*S*,8*S*,9*S*,10*R*,13*R*,14*S*,17*R*)-10,13-dimethyl-17-[(*R*)-6-methylheptan-2-yl]-2,3,4,7,8,9,10,11,12,13,14,15,16,17-tetradecahydro-1H-cyclopenta[*a*]phenanthren-3-yl acrylate (**3o**) (88.1 mg, 0.20 mmol, 1.0 equiv) at 60 °C for 30 h. **L12** was used as ligand. Isolation by column chromatography (*n*-hexane/EtOAc = 30:1 to 15:1) yielded **52** (47.0 mg, 43%) as a white solid. The *o* and *p*-olefinated products are known compounds[50] and the ratio of the isomers was determined by the ^{1}H-NMR of the crude mixture, *o* : *p* = 13 : 1. ^{1}H-NMR (600 MHz, $CDCl_3$): δ^o = 7.97 (d, *J* = 16.1 Hz, 1H), 7.50 (dd, *J* = 7.8, 1.7 Hz, 1H), 7.34 (ddd, *J* = 8.3, 7.4, 1.7 Hz, 1H), 6.95 (td, *J* = 7.5, 1.0 Hz, 1H), 6.91 (dd, *J* = 8.4, 1.0 Hz, 1H), 6.51 (d, *J* = 16.1 Hz, 1H), 5.40 (d, *J* = 5.6 Hz, 1H), 4.79 – 4.72 (m, 1H), 3.89 (s, 3H), 2.44 – 2.39 (m, 2H), 2.04 – 1.79 (m, 5H), 1.72 – 0.97 (m, 24H), 0.92 (d, *J* = 6.6 Hz, 3H), 0.87 (dd, *J* = 6.6, 2.7 Hz, 6H), 0.69 (s, 3H). ^{13}C-NMR (126 MHz, $CDCl_3$): δ^o = 166.9 (C_q), 158.3 (C_q), 139.8 (CH), 139.8 (C_q), 131.3 (CH), 128.9 (CH), 123.5 (C_q), 122.6 (CH), 120.6 (CH), 119.2 (CH), 111.1 (CH), 73.9 (CH), 56.7 (CH), 56.1 (CH), 55.4 (CH_3), 50.0 (CH), 42.3 (C_q), 39.7 (CH_2), 39.5 (CH_2), 38.2 (CH_2), 37.0 (CH_2), 36.6 (C_q), 36.2 (CH_2), 35.8 (CH), 31.9 (CH_2), 31.9 (CH), 28.2 (CH_2), 28.0 (CH), 27.9 (CH_2), 24.3 (CH_2), 23.8 (CH_2), 22.8 (CH_3), 22.6 (CH_3), 21.0 (CH_2), 19.4 (CH_3), 18.7 (CH_3), 11.9 (CH_3). IR (ATR): 2946, 2868, 2152, 1710, 1631, 1438, 1321, 1248, 1167, 1014 cm^{-1}. MS (ESI) *m*/*z* (relative intensity): 569 (100) $[M + Na]^+$, 547 (30) $[M + H]^+$. HR-MS (ESI): *m*/*z* calcd. for $[C_{37}H_{54}O_3 + H]^+$ 569.3965 found 569.3955.

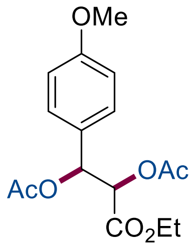

3-Ethoxy-1-(4-methoxyphenyl)-3-oxopropane-1,2-diyl diacetate (40')

The product is known compound.[51] ^{1}H-NMR (400 MHz, $CDCl_3$): δ = 7.34 – 7.27 (m, 2H), 6.89 – 6.84 (m, 2H), 6.18 (dd, *J* = 24.8, 4.9 Hz, 1H), 5.46 – 5.25 (m, 1H), 4.21 – 4.07 (m, 2H), 3.79 (d, *J* = 1.4 Hz, 3H), 2.13 – 2.05 (m, 6H), 1.27 – 1.10 (m, 3H). ^{13}C-NMR (100 MHz, $CDCl_3$): δ = 169.9 (C_q), 169.8 (C_q), 169.4 (C_q), 169.4 (C_q), 167.0 (C_q), 166.9 (C_q), 159.8 (C_q), 159.8 (C_q),

128.9 (CH), 128.3 (CH), 127.5 (C_q), 127.3 (C_q), 113.8 (CH), 113.7 (CH), 74.3 (CH), 73.5 (CH), 73.5 (CH), 73.1 (CH), 61.7 (CH_2), 61.7 (CH_2), 55.2 (CH_3), 55.2 (CH_3), 20.9 (CH_3), 20.8 (CH_3), 20.5 (CH_3), 20.4 (CH_3), 14.0 (CH_3), 13.9 (CH_3). IR (ATR): 3012, 2977, 2200, 2041, 1710, 1614, 1516, 1372, 1219, 1033 cm^{-1}. MS (ESI) *m*/*z* (relative intensity): 347 (100) $[M + Na]^+$, 363 (20) $[M + K]^+$. HR-MS (ESI): *m*/*z* calcd. for $[C_{16}H_{20}O_7 + Na]^+$ 347.1101 found 347.1097.

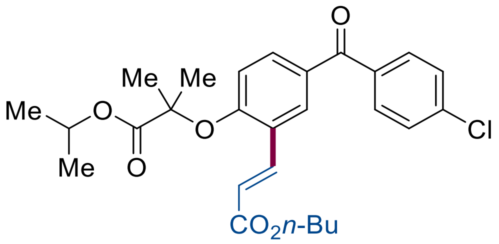

(*E*)-butyl-3-(5-(4-chlorobenzoyl)-2-((1-isopropoxy-2-methyl-1-oxopropan-2-yl)oxy)phenyl)acrylate (54a)

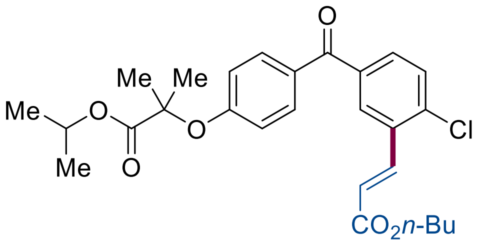

(*E*)-butyl-3-(2-chloro-5-(4-((1-isopropoxy-2-methyl-1-oxopropan-2-yl)oxy)benzoyl)phenyl)acrylate (54b)

The general procedure **D** was followed using fenofibrate (**53a**) (180.4 mg, 0.50 mmol, 1.0 equiv) and *n*-butyl acrylate (**3a**) (144.0 µL, 1.0 mmol, 2.0 equiv) at 80 °C for 48 h. **L12** was used as ligand. Isolation by column chromatography (*n*-hexane/EtOAc = 20:1) yielded **54** (51.1 mg, 21%) as a mixture. The site selectivity was determined by ^{1}H-NMR and NOESY NMR, the ratio of the isomers was determined by the ^{1}H-NMR of the crude mixture, **54a** : **54b** = 8 : 1. For **54a**, ^{1}H-NMR (600 MHz, $CDCl_3$): δ = 8.02 (d, J = 16.0 Hz, 1H), 8.00 (d, J = 2.3 Hz, 1H), 7.71 – 7.69 (m, 3H), 7.49 – 7.45 (m, 2H), 6.75 (d, J = 8.7 Hz, 1H), 6.49 (d, J = 16.2 Hz, 1H), 5.08 (p, J = 6.3 Hz, 1H), 4.21 (t, J = 6.7 Hz, 2H), 1.71 (s, 6H), 1.71 – 1.63 (m, 2H), 1.48 – 1.40 (m, 2H), 1.20 (s, 3H), 1.19 (s, 3H), 0.96 (t, J = 7.4 Hz, 3H). ^{13}C-NMR (125 MHz, $CDCl_3$): δ = 193.8 (C_q), 172.7 (C_q), 167.1 (C_q), 157.9 (C_q), 138.9 (CH), 138.7 (C_q), 136.0 (C_q), 132.5 (CH), 131.1 (CH), 130.8 (CH), 130.2 (C_q), 128.7 (CH), 125.2 (C_q), 120.1 (CH), 115.3 (CH), 80.4

(C_q), 69.5 (CH), 64.5 (CH_2), 30.7 (CH_2), 25.4 (CH_3), 21.5 (CH_3), 19.2 (CH_2), 13.7 (CH_3). For **54b**, ^{1}H-NMR (600 MHz, $CDCl_3$): δ = 8.09 (d, J = 16.0 Hz, 1H), 7.99 (d, J = 2.1 Hz, 1H), 7.75 – 7.72 (m, 2H), 7.67 (dd, J = 8.2, 2.0 Hz, 1H), 7.52 (d, J = 8.2 Hz, 1H), 6.90 – 6.86 (m, 2H), 6.45 (d, J = 16.0 Hz, 1H), 5.13 – 5.04 (m, 1H), 4.21 (t, J = 6.7 Hz, 2H), 1.71 – 1.63 (m, 2H), 1.67 (s, 6H), 1.48 – 1.40 (m, 2H), 1.21 (s, 3H), 1.20 (s, 3H), 0.96 (t, J = 7.4 Hz, 3H). IR (ATR): 2960, 2935, 2254, 1710, 1655, 1488, 1386, 1266, 1177, 909 cm^{-1}. MS (ESI) *m*/*z* (relative intensity): 509 (100) $[M + Na]^+$, 487 (20) $[M + H]^+$. HR-MS (ESI): *m*/*z* calcd. for $[C_{27}H_{31}ClO_6 + Na]^+$ 509.1701 found 509.1691.

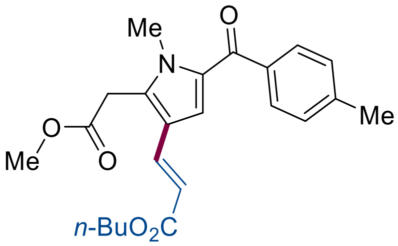

(*E*)-butyl-3-(5-(2-methoxy-2-oxoethyl)-1-methyl-2-(4-methylbenzoyl)-1*H*-pyrrol-3-yl)acrylate (55)

The general procedure **D** was followed using protected tolmetin (**53b**) (135.5 mg, 0.50 mmol, 1.0 equiv) and *n*-butyl acrylate (**3a**) (144.0 µL, 1.0 mmol, 2.0 equiv) at 80 °C for 48 h. **L12** was used as ligand. Isolation by column chromatography (*n*-hexane/EtOAc = 5:1) yielded **55** (147.2 mg, 74%). The site selectivity was determined by 1H-NMR and NOESY NMR. ^{1}H-NMR (400 MHz, $CDCl_3$): δ = 7.72 (d, J = 8.2 Hz, 2H), 7.59 (d, J = 15.6 Hz, 1H), 7.27 (d, J = 7.8 Hz, 2H), 6.90 (s, 1H), 6.12 (d, J = 15.6 Hz, 1H), 4.16 (t, J = 6.7 Hz, 2H), 3.95 (s, 3H), 3.85 (s, 2H), 3.75 (s, 3H), 2.44 (s, 3H), 1.69 – 1.59 (m, 2H), 1.44 – 1.32 (m, 2H), 0.94 (t, J = 7.4 Hz, 3H). ^{13}C-NMR (100 MHz, $CDCl_3$): δ = 186.3 (C_q), 168.9 (C_q), 167.7 (C_q), 142.7 (C_q), 136.7 (C_q), 135.7 (CH), 135.2 (C_q), 132.0 (C_q), 129.5 (CH), 129.0 (CH), 118.9 (CH), 118.7 (C_q), 115.4 (CH), 64.2 (CH_2), 52.7 (CH_3), 33.5 (CH_3), 30.8 (CH_2), 30.3 (CH_2), 21.6 (CH_3), 19.2 (CH_2), 13.7 (CH_3). IR (ATR): 2355, 2134, 2037, 1734, 1670, 1623, 1465, 1249, 1163, 906 cm^{-1}. MS (ESI) *m*/*z* (relative intensity): 420 (100) $[M + Na]^+$, 398 (30) $[M + H]^+$. HR-MS (ESI): *m*/*z* calcd. for $[C_{23}H_{27}NO_5 + Na]^+$ 420.1781 found 420.1776.

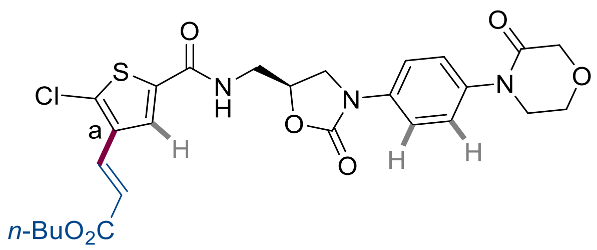

(*S*,*E*)-butyl-3-(2-chloro-5-(((2-oxo-3-(4-(3-oxomorpholino)phenyl)oxazolidin-5-yl)methyl)carbamoyl)thiophen-3-yl)acrylate (56a)

The general procedure **B** was followed using rivaroxaban (**53c**) (180.0 mg, 0.41 mmol, 2.1 equiv) and *n*-butyl acrylate (**3a**) (28.8 µL, 0.20 mmol, 1.0 equiv) at 80 °C for 20 h. **L12** was used as ligand. Isolation by two column chromatographies (*n*-hexane/Acetone = 1:1 & EtOAc) yielded **56a** (41.4 mg, 37%). Other isomers were also observed, but we failed to isolated and characterized. The site selectivity was determined by NOESY, the ratio of the isomers was determined by the ^{1}H-NMR of the crude mixture, **56a** : others = 9 : 1. For **56a**, ^{1}H-NMR (600 MHz, $CDCl_3$): δ = 7.75 (s, 1H), 7.60 (d, J = 16.0 Hz, 1H), 7.54 – 7.49 (m, 3H), 7.32 – 7.28 (m, 2H), 6.27 (d, J = 16.0 Hz, 1H), 4.81 – 4.76 (m, 1H), 4.33 (d, J = 1.1 Hz, 2H), 4.20 (t, J = 6.7 Hz, 2H), 4.05 – 4.00 (m, 3H), 3.81 (dd, J = 9.1, 6.7 Hz, 1H), 3.74 – 3.69 (m, 3H), 3.68 – 3.63 (m, 1H), 1.71 – 1.65 (m, 2H), 1.46 – 1.38 (m, 2H), 0.95 (t, J = 7.4 Hz, 3H). ^{13}C-NMR (125 MHz, $CDCl_3$): δ = 167.1 (C_q), 166.7 (C_q), 161.5 (C_q), 154.5 (C_q), 137.3 (C_q), 136.9 (C_q), 136.6 (C_q), 136.2 (C_q), 134.2 (C_q), 133.9 (CH), 126.3 (CH), 125.6 (CH), 120.3 (CH), 119.0 (CH), 71.6 (CH), 68.5 (CH_2), 64.8 (CH_2), 64.0 (CH_2), 49.7 (CH_2), 47.6 (CH_2), 42.3 (CH_2), 30.7 (CH_2), 19.2 (CH_2), 13.7 (CH_3). IR (ATR): 3323, 3285, 2961, 2928, 2007, 1754, 1713, 1644, 1517, 1278 cm^{-1}. MS (ESI) *m*/*z* (relative intensity): 584 (100) $[M + Na]^+$, 562 (10) $[M + H]^+$. HR-MS (ESI): *m*/*z* calcd. for $[C_{26}H_{28}ClN_3O_7S + Na]^+$ 584.1229 found 584.1229.

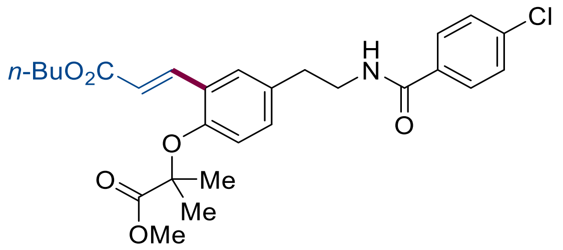

(*E*)-butyl-3-(5-(2-(4-chlorobenzamido)ethyl)-2-((1-methoxy-2-methyl-1-oxopropan-2-yl)oxy)phenyl)acrylate (57)

The general procedure **D** was followed using protected gemfibrozil (**53d**) (188.0 mg, 0.50 mmol, 1.0 equiv) and *n*-butyl acrylate (**3a**) (144.0 µL, 1.0 mmol, 2.0 equiv) at 80 °C for 48 h. **L12** was used as ligand. Isolation by column chromatography (*n*-hexane/EtOAc = 2:1) yielded **57** (100.4 mg, 40%). The site selectivity was determined by ^{1}H-NMR and NOESY NMR. ^{1}H-NMR (600 MHz, $CDCl_3$): δ = 8.01 (d, J = 16.5 Hz, 1H), 7.65 – 7.61 (m, 2H), 7.40 (d, J = 2.3 Hz, 1H), 7.39 – 7.37 (m, 2H), 7.10 (dd, J = 8.4, 2.3 Hz, 1H), 6.67 (d, J = 8.4 Hz, 1H), 6.43 (d, J = 16.2 Hz, 1H), 6.09 (brs, 1H), 4.20 (t, J = 6.7 Hz, 2H), 3.77 (s, 3H), 3.69 – 3.65 (m, 2H), 2.87 (t, J = 7.0 Hz, 2H), 1.72 – 1.66 (m, 2H), 1.63 (s, 6H), 1.48 – 1.41 (m, 2H), 0.97 (t, J = 7.4 Hz, 3H). ^{13}C-NMR (125 MHz, $CDCl_3$): δ = 174.5 (C_q), 167.3 (C_q), 166.4 (C_q), 153.0 (C_q), 139.6 (CH), 137.7 (C_q), 132.9 (C_q), 132.3 (C_q), 131.0 (CH), 128.9 (CH), 128.5 (CH), 128.2 (CH), 126.5 (C_q), 119.0 (CH), 117.6 (CH), 79.9 (C_q), 64.3 (CH_2), 52.6 (CH_3), 41.1 (CH_2), 34.8 (CH_2), 30.8 (CH_2), 25.3 (CH_3), 19.2 (CH_2), 13.7 (CH_3). IR (ATR): 2959, 2866, 2253, 1731, 1706, 1485, 1248, 1173, 905, 729 cm^{-1}. MS (ESI) *m*/*z* (relative intensity): 524 (100) $[M + Na]^+$, 502 (40) $[M + H]^+$. HR-MS (ESI): *m*/*z* calcd. for $[C_{27}H_{32}ClNO_6 + Na]^+$ 524.1810 found 524.1807.

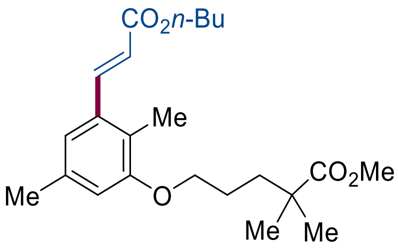

(*E*)-methyl-5-(3-(3-butoxy-3-oxoprop-1-en-1-yl)-2,5-dimethylphenoxy)-2,2-dimethylpentanoate (58a)

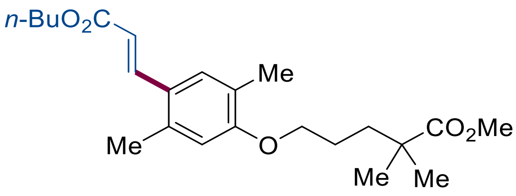

(*E*)-methyl-5-(4-(3-butoxy-3-oxoprop-1-en-1-yl)-2,5-dimethylphenoxy)-2,2-dimethylpentanoate (58b)

The general procedure **D** was followed using protected gemfibrozil (**53e**) (132.6 µL, 0.50 mmol, 1.0 equiv) and *n*-butyl acrylate (**3a**) (144.0 µL, 1.0 mmol, 2.0 equiv) at 80 °C for 48 h. **L12**

was used as ligand. Isolation by column chromatography (*n*-hexane/EtOAc = 20:1) yielded **58a** (7.8 mg, 4%) and **58b** (128.9 mg, 66%). The site selectivity was determined by ^{1}H-NMR and HMBC. For **58a**, ^{1}H-NMR (600 MHz, $CDCl_3$): δ = 7.99 (d, J = 15.8 Hz, 1H), 6.98 (s, 1H), 6.65 (s, 1H), 6.33 (d, J = 15.8 Hz, 1H), 4.21 (t, J = 6.7 Hz, 2H), 3.91 (t, J = 5.6 Hz, 2H), 3.67 (s, 3H), 2.31 (s, 3H), 2.26 (s, 3H), 1.76 – 1.66 (m, 6H), 1.48 – 1.40 (m, 2H), 1.22 (s, 6H), 0.97 (t, J = 7.4 Hz, 3H). ^{13}C-NMR (125 MHz, $CDCl_3$): δ = 178.2 (C_q), 167.2 (C_q), 157.1 (C_q), 142.8 (CH), 136.0 (C_q), 134.3 (C_q), 123.8 (C_q), 119.5 (CH), 119.0 (CH), 113.5 (CH), 68.4 (CH_2), 64.3 (CH_2), 51.7 (CH_3), 42.1 (C_q), 37.1 (CH_2), 30.8 (CH_2), 25.2 (CH_3), 25.1 (CH_2), 21.4 (CH_3), 19.2 (CH_2), 13.7 (CH_3), 11.2 (CH_3). For **58b**, ^{1}H-NMR (400 MHz, $CDCl_3$): δ = 7.90 (d, J = 15.8 Hz, 1H), 7.37 (s, 1H), 6.58 (s, 1H), 6.26 (d, J = 15.8 Hz, 1H), 4.19 (t, J = 6.7 Hz, 2H), 3.94 (t, J = 5.6 Hz, 2H), 3.66 (s, 3H), 2.40 (s, 3H), 2.18 (s, 3H), 1.77 – 1.64 (m, 6H), 1.49 – 1.39 (m, 2H), 1.22 (s, 6H), 0.96 (t, J = 7.4 Hz, 3H). ^{13}C-NMR (100 MHz, $CDCl_3$): δ = 178.3 (C_q), 167.7 (C_q), 158.7 (C_q), 141.9 (CH), 137.2 (C_q), 128.7 (CH), 125.1 (C_q), 124.8 (C_q), 116.0 (CH), 112.8 (CH), 68.0 (CH_2), 64.2 (CH_2), 51.8 (CH_3), 42.1 (C_q), 37.0 (CH_2), 30.9 (CH_2), 25.2 (CH_3), 25.1 (CH_2), 19.8 (CH_3), 19.3 (CH_2), 15.8 (CH_3), 13.8 (CH_3). IR (ATR): 2983, 2934, 1738, 1606, 1505, 1373, 1240, 1164, 1094, 1046 cm^{-1}. MS (ESI) *m*/*z* (relative intensity): 413 (100) $[M + Na]^{+}$, 391 (10) $[M + H]^{+}$. HR-MS (ESI): *m*/*z* calcd. for $[C_{23}H_{34}O_5 + Na]^{+}$ 413.2298 found 413.2296.

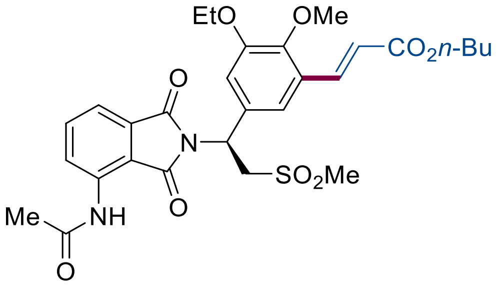

(*S*,*E*)-butyl-3-(5-(1-(4-acetamido-1,3-dioxoisoindolin-2-yl)-2-(methylsulfonyl)ethyl)-3-ethoxy-2-methoxyphenyl)acrylate (59a)

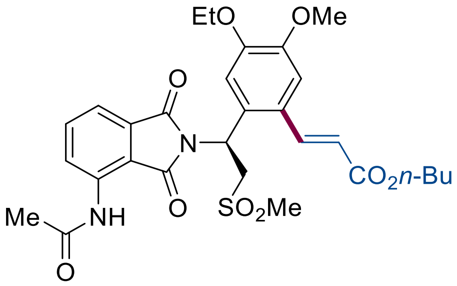

(*S*,*E*)-butyl-3-(2-(1-(4-acetamido-1,3-dioxoisoindolin-2-yl)-2-(methylsulfonyl)ethyl)-4-ethoxy-5-methoxyphenyl)acrylate (59b)

The general procedure **D** was followed using protected gemfibrozil (**53f**) (230.3 mg, 0.50 mmol, 1.0 equiv) and *n*-butyl acrylate (**3a**) (144.0 µL, 1.0 mmol, 2.0 equiv) at 80 °C for 48 h. **L12** was used as ligand. Isolation by column chromatography (*n*-hexane/EtOAc = 1:1) yielded **59a** (64.5 mg, 22%) and **59b** (26.4 mg, 9%). The site selectivity was determined by ^{1}H-NMR and NOESY NMR. For **59a**, ^{1}H-NMR (600 MHz, $CDCl_3$): δ = 9.44 (s, 1H), 8.76 (dd, *J* = 8.6, 0.8 Hz, 1H), 7.92 (d, *J* = 16.6 Hz, 1H), 7.65 (ddd, *J* = 8.5, 7.3, 0.4 Hz, 1H), 7.49 (dd, *J* = 7.3, 0.8 Hz, 1H), 7.29 (d, *J* = 2.1 Hz, 1H), 7.12 (d, *J* = 2.1 Hz, 1H), 6.51 (d, *J* = 16.2 Hz, 1H), 5.88 (dd, *J* = 10.8, 3.9 Hz, 1H), 4.62 (ddd, *J* = 14.4, 10.9, 0.7 Hz, 1H), 4.21 (t, *J* = 6.7 Hz, 2H), 4.10 (q, *J* = 7.0 Hz, 2H), 3.85 (s, 3H), 3.66 (dd, *J* = 14.3, 4.0 Hz, 1H), 2.92 (s, 3H), 2.28 (s, 3H), 1.74 – 1.66 (m, 2H), 1.52 – 1.40 (m, 5H), 0.97 (t, *J* = 7.4 Hz, 3H). ^{13}C-NMR (125 MHz, $CDCl_3$): δ = 169.4 (C_q), 169.2 (C_q), 167.5 (C_q), 167.0 (C_q), 152.8 (C_q), 148.9 (C_q), 138.6 (CH), 137.7 (C_q), 136.2 (CH), 132.7 (C_q), 131.0 (C_q), 129.0 (C_q), 125.1 (CH), 120.5 (CH), 118.6 (CH), 118.3 (CH), 115.0 (C_q), 114.4 (CH), 64.7 (CH_2), 64.5 (CH_2), 61.1 (CH_3), 54.0 (CH_2), 48.4 (CH), 41.8 (CH_3), 30.8 (CH_2), 25.0 (CH_3), 19.2 (CH_2), 14.6 (CH_3), 13.7 (CH_3). For **59b**, ^{1}H-NMR (600 MHz, $CDCl_3$): δ = 9.45 (s, 1H), 8.77 (dd, *J* = 8.6, 0.8 Hz, 1H), 8.32 (d, *J* = 15.5 Hz, 1H), 7.66 (dd, *J* = 8.5, 7.3 Hz, 1H), 7.50 (dd, *J* = 7.3, 0.8 Hz, 1H), 7.34 (s, 1H), 7.03 (s, 1H), 6.30 (d, *J* = 15.5 Hz, 1H), 6.28 (dd, *J* = 11.0, 3.7 Hz, 1H), 4.56 (ddd, *J* = 14.7, 10.9, 0.9 Hz, 1H), 4.25 (td, *J* = 6.7, 2.0 Hz, 2H), 4.20 – 4.13 (m, 2H), 3.89 (s, 3H), 3.54 (dd, *J* = 14.6, 3.7 Hz, 1H), 2.95 (s, 3H), 2.27 (s, 3H), 1.77 – 1.71 (m, 2H), 1.50 (t, *J* = 7.0 Hz, 3H), 1.52 – 1.45 (m, 2H), 0.99 (t, *J* = 7.4 Hz, 3H). ^{13}C-NMR (125 MHz, $CDCl_3$): δ = 169.6 (C_q), 169.2 (C_q), 167.8 (C_q), 166.6 (C_q), 150.4 (C_q), 149.6 (C_q), 139.9 (CH), 137.7 (C_q), 136.2 (CH), 131.1 (C_q), 128.8 (C_q), 125.7 (C_q), 125.1 (CH), 120.4 (CH), 118.4 (CH), 115.0 (C_q), 112.2 (CH), 109.4 (CH), 64.7 (CH_2), 64.6 (CH_2), 56.0 (CH_3), 54.5 (CH_2), 44.6 (CH), 41.2 (CH_3), 30.8 (CH_2), 25.0 (CH_3), 19.2 (CH_2), 14.6 (CH_3), 13.8 (CH_3). IR (ATR): 2983, 2925, 2357, 2186, 1699, 1169, 990, 968, 951, 756 cm^{-1}. MS (ESI) *m*/*z* (relative intensity): 609 (100) $[M + Na]^+$, 587 (10) $[M + H]^+$. HR-MS (ESI): *m*/*z* calcd. for $[C_{29}H_{34}N_2O_9S + Na]^+$ 609.1877 found 609.1879.

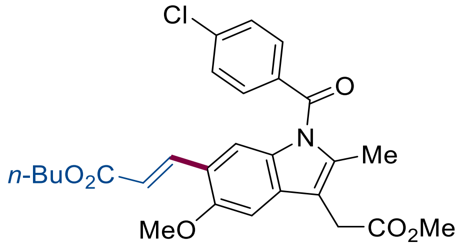

(*E*)-butyl-3-(1-(4-chlorobenzoyl)-5-methoxy-3-(2-methoxy-2-oxoethyl)-2-methyl-1H-indol-6-yl)acrylate (60a)

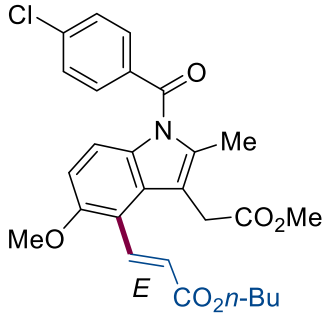

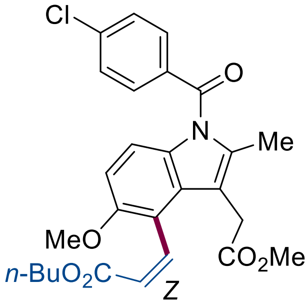

(*E*)-butyl-3-(1-(4-chlorobenzoyl)-5-methoxy-3-(2-methoxy-2-oxoethyl)-2-methyl-1H-indol-4-yl)acrylate (*trans*-60b) & (*Z*)-butyl-3-(1-(4-chlorobenzoyl)-5-methoxy-3-(2-methoxy-2-oxoethyl)-2-methyl-1H-indol-4-yl)acrylate (*cis*-60b)

The general procedure **D** was followed using protected gemfibrozil (**53g**) (185.6 mg, 0.50 mmol, 1.0 equiv) and *n*-butyl acrylate (**3a**) (144.0 µL, 1.0 mmol, 2.0 equiv) at 80 °C for 48 h. **L12** was used as ligand. Isolation by column chromatography (*n*-hexane/EtOAc = 5:1) yielded **60a** (109.4 mg, 44%) and **60b** (110.3 mg, 44%). The site selectivity was determined by ^{1}H-NMR and NOESY NMR. For **60a**, ^{1}H-NMR (400 MHz, $CDCl_3$): δ = 7.91 (d, *J* = 16.0 Hz, 1H), 7.69 – 7.64 (m, 2H), 7.52 – 7.48 (m, 2H), 7.23 (s, 1H), 6.93 (s, 1H), 6.23 (d, *J* = 16.1 Hz, 1H), 4.17 (t, *J* = 6.7 Hz, 2H), 3.93 (s, 3H), 3.71 (s, 3H), 3.67 (s, 2H), 2.35 (s, 3H), 1.73 – 1.61 (m, 2H), 1.49 – 1.36 (m, 2H), 0.95 (t, *J* = 7.4 Hz, 3H). ^{13}C-NMR (100 MHz, $CDCl_3$): δ = 171.1 (C_q), 168.2 (C_q), 167.7 (C_q), 155.2 (C_q), 140.7 (CH), 139.7 (C_q), 137.6 (C_q), 133.6 (C_q), 132.2 (C_q), 131.2 (CH), 130.7 (C_q), 129.3 (CH), 120.1 (C_q), 117.7 (CH), 114.4 (CH), 112.7 (C_q), 99.6 (CH), 64.2 (CH_2), 55.9 (CH_3), 52.7 (CH_3), 30.8 (CH_2), 30.2 (CH_2), 19.2 (CH_2), 13.8 (CH_3), 13.7 (CH_3). For **60b,** both *trans*- and *cis*-form were observed as a mixture, *E*:*Z*=1:0.8. For *trans*-form, ^{1}H-NMR (400 MHz, $CDCl_3$): δ = 8.13 (d, *J* = 15.9 Hz, 1H), 7.67 (dd, *J* = 8.6, 1.3 Hz, 2H), 7.47 (dd, *J* = 8.6, 2.6 Hz, 2H), 7.06 (d, *J* = 9.1 Hz, 1H), 6.72 (d, *J* = 9.2 Hz, 1H), 6.57

(d, J = 16.0 Hz, 1H), 4.22 (t, J = 6.7 Hz, 2H), 3.85 (s, 3H), 3.84 (s, 2H), 3.73 (s, 3H), 2.31 (s, 3H), 1.75 – 1.66 (m, 2H), 1.51 – 1.42 (m, 2H), 0.97 (t, J = 7.4 Hz, 3H). For *cis*-form, ^{1}H-NMR (400 MHz, $CDCl_3$): δ = 7.69 – 7.64 (m, 2H), 7.49 – 7.45 (m, 2H), 7.26 (d, J = 11.9 Hz, 1H), 6.96 (dd, J = 9.1, 0.8 Hz, 1H), 6.68 (d, J = 9.1 Hz, 1H), 6.22 (d, J = 11.9 Hz, 1H), 3.95 (t, J = 6.6 Hz, 2H), 3.75 (s, 3H), 3.72 (s, 2H), 3.68 (s, 3H), 2.31 (s, 3H), 1.41 – 1.33 (m, 2H), 1.17 – 1.06 (m, 2H), 0.80 (t, J = 7.3 Hz, 3H). Mixed ^{13}C-NMR (100 MHz, $CDCl_3$): δ = 171.7 (C_q), 171.5 (C_q), 168.2 (C_q), 168.2 (C_q), 167.5 (C_q), 165.9 (C_q), 154.8 (C_q), 152.1 (C_q), 139.6 (C_q), 139.3 (C_q), 138.1 (CH), 137.8 (CH), 137.7 (C_q), 136.9 (C_q), 133.9 (C_q), 133.7 (C_q), 131.6 (C_q), 131.3 (CH), 131.3 (C_q), 131.2 (CH), 129.2 (CH), 129.1 (CH), 129.0 (C_q), 127.7 (C_q), 124.6 (CH), 123.7 (CH), 116.5 (C_q), 115.3 (C_q), 115.3 (CH), 113.9 (CH), 112.6 (C_q), 112.4 (C_q), 107.6 (CH), 107.5 (CH), 64.2 (CH_2), 63.9 (CH_2), 56.5 (CH_3), 56.3 (CH_3), 52.2 (CH_3), 52.1 (CH_3), 31.9 (CH_2), 31.1 (CH_2), 30.8 (CH_2), 30.4 (CH_2), 19.2 (CH_2), 18.9 (CH_2), 13.8 (CH_3), 13.6 (CH_3), 13.3 (CH_3). IR (ATR): 2955, 2933, 2872, 1737, 1693, 1593, 1430, 1329, 1224, 1167 cm^{-1}. MS (ESI) *m*/*z* (relative intensity): 520 (100) $[M + Na]^+$, 498 (50) $[M + H]^+$. HR-MS (ESI): *m*/*z* calcd. for $[C_{27}H_{28}ClNO_6 + Na]^+$ 520.1497 found 520.1491.

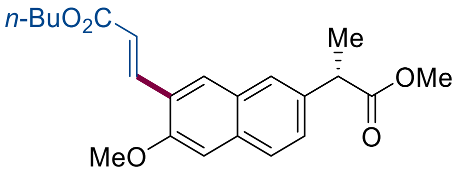

(*S*,*E*)-butyl-3-(3-methoxy-7-(1-methoxy-1-oxopropan-2-yl)naphthalen-2-yl)acrylate (61a)

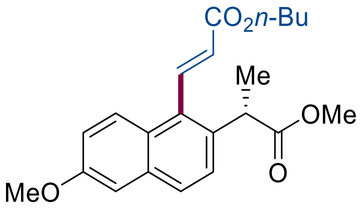

(*S*,*E*)-butyl-3-(3-methoxy-7-(1-methoxy-1-oxopropan-2-yl)naphthalen-1-yl)acrylate (61b)

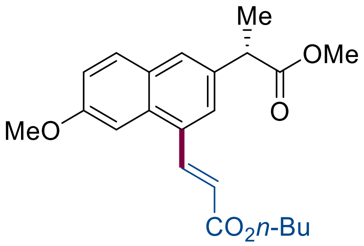

(*S*,*E*)-butyl-3-(7-methoxy-3-(1-methoxy-1-oxopropan-2-yl)naphthalen-1-yl)acrylate (61c)

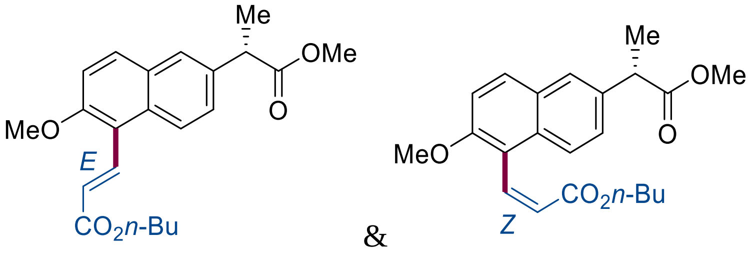

(*S*,*E*)-butyl-3-(2-methoxy-6-(1-methoxy-1-oxopropan-2-yl)naphthalen-1-yl)acrylate & (*S*,*Z*)-butyl-3-(2-methoxy-6-(1-methoxy-1-oxopropan-2-yl)naphthalen-1-yl)acrylate (61d)

The general procedure **D** was followed using protected Naproxen (**53h**) (122.1 mg, 0.50 mmol, 1.0 equiv) and *n*-butyl acrylate (**3a**) (144.0 µL, 1.0 mmol, 2.0 equiv) at 80 °C for 48 h. **L12** was used as ligand. Isolation by column chromatography (*n*-hexane/EtOAc = 5:1) yielded **61** (101.7 mg, 55%) as a mixture. The site selectivity was determined by ^{1}H-NMR and NOESY NMR, $\alpha : \beta : \gamma : \delta$ = 1 : 0.2 : 1 : 0.8. For **61a**, ^{1}H-NMR (600 MHz, $CDCl_3$): δ = 8.06 (dd, *J* = 16.1, 0.6 Hz, 1H), 7.94 (t, *J* = 0.6 Hz, 1H), 7.68 (d, *J* = 8.5 Hz, 1H), 7.66 (dd, *J* = 1.3, 0.7 Hz, 1H), 7.42 (dd, *J* = 8.5, 1.9 Hz, 1H), 7.11 (s, 1H), 6.69 (d, *J* = 16.1 Hz, 1H), 4.23 (t, *J* = 6.7 Hz, 2H), 3.98 (s, 3H), 3.86 (q, *J* = 7.2 Hz, 1H), 3.67 (s, 3H), 1.74 – 1.68 (m, 2H), 1.58 (d, *J* = 7.2 Hz, 3H), 1.50 – 1.42 (m, 2H), 0.98 (t, *J* = 7.4 Hz, 3H). ^{13}C-NMR (125 MHz, $CDCl_3$): δ = 175.0 (C_q), 167.5 (C_q), 156.1 (C_q), 140.3 (CH), 136.3 (C_q), 134.3 (C_q), 129.4 (CH), 128.3 (C_q), 127.3 (CH), 126.9 (CH), 126.4 (CH), 125.5 (C_q), 120.2 (CH), 105.5 (CH), 64.4 (CH_2), 55.5 (CH_3), 52.1 (CH_3), 45.3 (CH), 30.8 (CH_2), 19.2 (CH_2), 18.5 (CH_3), 13.8 (CH_3). For **61b**, ^{1}H-NMR (600 MHz, $CDCl_3$): δ = 8.19 (d, *J* = 16.3 Hz, 1H), 7.87 (d, *J* = 9.1 Hz, 1H), 7.71 (d, *J* = 8.5 Hz, 1H), 7.42 (d, *J* = 8.6 Hz, 1H), 7.16 (dd, *J* = 9.2, 2.7 Hz, 1H), 7.12 (d, *J* = 2.6 Hz, 1H), 6.24 (d, *J* = 16.3 Hz, 1H), 4.29 – 4.27 (m, 2H), 4.17 (q, *J* = 7.1 Hz, 1H), 3.92 (s, 3H), 3.65 (s, 3H), 1.77 – 1.69 (m, 2H), 1.51 – 1.44 (m, 5H), 0.99 (t, *J* = 7.4, 3H). For **61c**, ^{1}H-NMR (600 MHz, $CDCl_3$): δ = 8.42 (d, *J* = 15.7 Hz, 1H), 7.74 (d, *J* = 9.0 Hz, 1H), 7.72 (d, *J* = 1.8 Hz, 1H), 7.68 (d, *J* = 1.8 Hz, 1H), 7.36 (d, *J* = 2.4 Hz, 1H), 7.19 (dd, *J* = 8.9, 2.4 Hz, 1H), 6.55 (d, *J* = 15.7 Hz, 1H), 4.27 (t, *J* = 6.7 Hz, 2H), 3.95 (s, 3H), 3.86 (q, *J* = 7.2 Hz, 1H), 3.68 (s, 3H), 1.76 – 1.71 (m, 2H), 1.59 (d, *J* = 7.2 Hz, 3H), 1.52 – 1.44 (m, 2H), 0.99 (t, *J* = 7.4 Hz, 3H). ^{13}C-NMR (125 MHz, $CDCl_3$): δ = 174.9 (C_q), 167.9 (C_q), 156.6 (C_q), 137.5 (CH), 135.9 (C_q), 131.9 (C_q), 131.3 (CH), 129.0 (C_q), 127.3 (CH), 126.7 (CH), 123.8 (CH), 123.4 (CH), 116.7 (C_q), 113.0 (CH), 64.4 (CH_2), 56.2 (CH_3), 52.1 (CH_3), 45.1 (CH), 30.8 (CH_2), 19.2 (CH_2), 18.4 (CH_3), 13.8 (CH_3).

For **61d**, both *trans*- and *cis*-form were observed as a mixture, *E*:*Z*=5:1. For *trans*-form, ^{1}H-NMR (600 MHz, $CDCl_3$): δ = 8.32 (d, *J* = 16.2 Hz, 1H), 8.15 (d, *J* = 8.8 Hz, 1H), 7.82 (d, *J* = 9.0 Hz, 1H), 7.69 (d, *J* = 2.0 Hz, 1H), 7.48 (dd, *J* = 8.9, 2.0 Hz, 1H), 7.29 (d, *J* = 9.1 Hz, 1H), 6.75 (d, *J* = 16.2 Hz, 1H), 4.26 (t, *J* = 6.8 Hz, 2H), 4.00 (s, 3H), 3.87 (q, *J* = 7.1 Hz, 1H), 3.67 (s, 3H), 1.76 – 1.70 (m, 2H), 1.59 (d, *J* = 7.2 Hz, 3H), 1.51 – 1.43 (m, 2H), 0.98 (t, *J* = 7.4 Hz, 3H). ^{13}C-NMR (125 MHz, $CDCl_3$): δ = 174.9 (C_q), 167.9 (C_q), 156.6 (C_q), 137.5 (CH), 135.9 (C_q), 131.9 (C_q), 131.3 (CH), 129.0 (C_q), 127.3 (CH), 126.7 (CH), 123.8 (CH), 123.4 (CH), 116.7 (C_q), 113.0 (CH), 64.4 (CH_2), 56.2 (CH_3), 52.1 (CH_3), 45.1 (CH), 30.8 (CH_2), 19.2 (CH_2), 18.4 (CH_3), 13.8 (CH_3). For *cis*-form, ^{1}H-NMR (600 MHz, $CDCl_3$): δ = 7.78 (d, *J* = 9.1 Hz, 1H), 7.73 (dt, *J* = 8.8, 0.8 Hz, 1H), 7.67 (d, *J* = 2.0 Hz, 1H), 7.38 (dd, *J* = 8.8, 1.9 Hz, 1H), 7.26 (d, *J* = 9.1 Hz, 1H), 7.24 (d, *J* = 11.9 Hz, 1H), 6.32 (d, *J* = 12.0 Hz, 1H), 3.91 (s, 3H), 3.85 (t, *J* = 6.6 Hz, 2H), 3.85 (q, *J* = 7.2 Hz, 1H), 3.66 (s, 3H), 1.57 (d, *J* = 7.1 Hz, 3H), 1.20 – 1.14 (m, 2H), 0.95 – 0.90 (m, 2H), 0.69 (t, *J* = 7.4 Hz, 3H). ^{13}C-NMR (125 MHz, $CDCl_3$): δ = 175.0 (C_q), 166.1 (C_q), 153.5 (C_q), 137.4 (CH), 135.6 (C_q), 131.0 (C_q), 129.5 (CH), 128.7 (C_q), 126.5 (CH), 126.3 (CH), 124.5 (CH), 124.5 (CH), 119.2 (C_q), 113.1 (CH), 63.9 (CH_2), 56.4 (CH_3), 52.0 (CH_3), 45.2 (CH), 30.2 (CH_2), 18.8 (CH_2), 18.5 (CH_3), 13.5 (CH_3). IR (ATR): 2961, 2936, 2254, 1731, 1706, 1627, 1462, 1260, 1172, 905 cm^{-1}. MS (ESI) *m*/*z* (relative intensity): 393 (100) $[M + Na]^+$, 371 (0) $[M + H]^+$. HR-MS (ESI): *m*/*z* calcd. for $[C_{22}H_{26}O_5 + Na]^+$ 393.1672 found 393.1673.

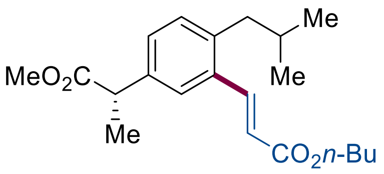

(*S*,*E*)-butyl-3-(2-isobutyl-5-(1-methoxy-1-oxopropan-2-yl)phenyl)acrylate (62a)

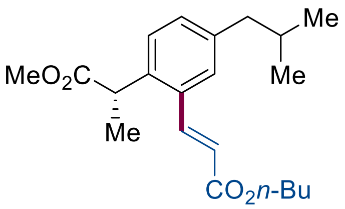

(*S*,*E*)-butyl-3-(5-isobutyl-2-(1-methoxy-1-oxopropan-2-yl)phenyl)acrylate (62b)

The general procedure **D** was followed using protected *S*-form ibuprofen (**53i**) (112.9 µL, 0.50 mmol, 1.0 equiv) and *n*-butyl acrylate (**3a**) (144.0 µL, 1.00 mmol, 2.0 equiv) at 80 °C for 48 h. **L12** was used as ligand. Isolation by column chromatography (*n*-hexane/EtOAc = 30:1) yielded **62a** (17.3 mg, 10%) and **62b** (10.4 mg, 6%). **62b** was known compound.[52] The site selectivity was determined by ^{1}H-NMR and NOESY NMR. For **62a**, ^{1}H-NMR (600 MHz, $CDCl_3$): δ = 7.97 (d, J = 15.8 Hz, 1H), 7.49 (d, J = 2.0 Hz, 1H), 7.23 (dd, J = 7.9, 2.0 Hz, 1H), 7.11 (d, J = 7.9 Hz, 1H), 6.37 (d, J = 15.8 Hz, 1H), 4.21 (t, J = 6.7 Hz, 2H), 3.71 (q, J = 7.2 Hz, 1H), 3.67 (s, 3H), 2.59 (d, J = 7.2 Hz, 2H), 1.80 (hept, J = 7.0 Hz, 1H), 1.73 – 1.67 (m, 2H), 1.50 (d, J = 7.2 Hz, 3H), 1.48 – 1.42 (m, 2H), 0.97 (t, J = 7.4 Hz, 3H), 0.91 (s, 3H), 0.90 (s, 3H). ^{13}C-NMR (125 MHz, $CDCl_3$): δ = 174.8 (C_q), 167.1 (C_q), 142.2 (CH), 140.3 (C_q), 138.51 (C_q), 133.4 (C_q), 131.3 (CH), 128.7 (CH), 125.5 (CH), 119.3 (CH), 64.4 (CH_2), 52.1 (CH_3), 45.0 (CH), 42.1 (CH_2), 30.8 (CH_2), 30.4 (CH), 22.4 (CH_3), 19.2 (CH_2), 18.5 (CH_3), 13.7 (CH_3). For **62b**, ^{1}H-NMR (600 MHz, $CDCl_3$): δ = 8.05 (d, J = 15.7 Hz, 1H), 7.32 (d, J = 1.9 Hz, 1H), 7.22 (d, J = 8.0 Hz, 1H), 7.14 (dd, J = 8.0, 1.9 Hz, 1H), 6.35 (d, J = 15.7 Hz, 1H), 4.22 (t, J = 6.5 Hz, 2H), 4.09 (q, J = 7.1 Hz, 1H), 3.66 (s, 3H), 2.45 (d, J = 7.2 Hz, 2H), 1.86 (hept, J = 6.7 Hz, 1H), 1.73 – 1.67 (m, 2H), 1.47 (d, J = 7.2 Hz, 3H), 1.46 – 1.43 (m, 2H), 0.97 (t, J = 7.4 Hz, 3H), 0.91 (s, 3H), 0.90 (s, 3H). ^{13}C-NMR (125 MHz, $CDCl_3$): δ = 174.8 (C_q), 166.9 (C_q), 142.0 (CH), 140.8 (C_q), 137.3 (C_q), 132.8 (C_q), 131.2 (CH), 127.7 (CH), 127.0 (CH), 120.7 (CH), 64.5 (CH_2), 52.1 (CH_3), 44.9 (CH_2), 40.7 (CH), 30.8 (CH_2), 30.1 (CH), 22.4 (CH_3), 22.4 (CH_3), 19.2 (CH_2), 18.6 (CH_3), 13.7 (CH_3). IR (ATR): 2960, 2936, 2169, 1738, 1717, 1449, 1372, 1234, 1169, 1045 cm^{-1}. MS (ESI) m/z (relative intensity): 369 (100) $[M + Na]^+$, 347 (0) $[M + H]^+$. HR-MS (ESI): m/z calcd. for $[C_{21}H_{30}O_4 + Na]^+$ 369.2036 found 369.2040.

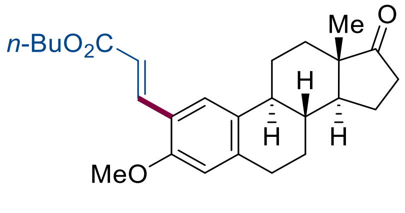

(*E*)-butyl-3-((*8R,9S,13S,14S*)-3-methoxy-13-methyl-17-oxo-7,8,9,11,12,13,14,15,16,17-decahydro-6*H*-cyclopenta[*a*]phenanthren-2-yl)acrylate (63a)

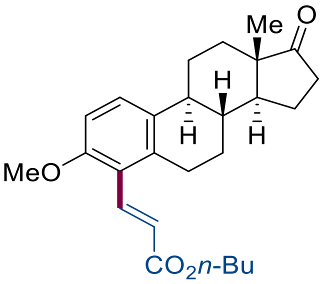

(*E*)-butyl-3-((*8R,9S,13S,14S*)-3-methoxy-13-methyl-17-oxo-7,8,9,11,12,13,14,15,16,17-decahydro-6*H*-cyclopenta[*a*]phenanthren-4-yl)acrylate (63b)

The general procedure **D** was followed using Estrone-OMe (**53j**) (142.2 mg, 0.5 mmol, 1.0 equiv) and *n*-butyl acrylate (**3a**) (144.0 µL, 1.0 mmol, 2.0 equiv) at 80 °C for 48 h. **L12** was used as ligand. Isolation by column chromatography (*n*-hexane/EtOAc = 5:1) yielded **63** (150.0 mg, 73%) as a mixture. The site selectivity was determined by ^{1}H-NMR and HMBC, $\alpha : \beta$ = 10 : 1. We failed to obtain pure NMR spectra of **63b**. For **63a**, ^{1}H-NMR (600 MHz, $CDCl_3$): δ = 7.93 (d, J = 16.1 Hz, 1H), 7.42 (d, J = 1.1 Hz, 1H), 6.63 (d, J = 1.3 Hz, 1H), 6.50 (d, J = 16.1 Hz, 1H), 4.19 (t, J = 6.7 Hz, 2H), 3.85 (s, 3H), 2.92 (dd, J = 9.0, 4.2 Hz, 2H), 2.55 – 2.47 (m, 1H), 2.46 – 2.40 (m, 1H), 2.28 – 2.22 (m, 1H), 2.18 – 2.11 (m, 1H), 2.08 – 2.00 (m, 2H), 1.99 – 1.94 (m, 1H), 1.71 – 1.65 (m, 2H), 1.65 – 1.60 (m, 1H), 1.60 – 1.48 (m, 4H), 1.47 – 1.41 (m, 3H), 0.96 (t, J = 7.4 Hz, 3H), 0.91 (s, 3H). ^{13}C-NMR (125 MHz, $CDCl_3$): δ = 220.7 (C_q), 167.8 (C_q), 156.4 (C_q), 140.5 (C_q), 140.3 (CH), 132.0 (C_q), 126.2 (CH), 121.0 (C_q), 117.8 (CH), 111.4 (CH), 64.2 (CH_2), 55.5 (CH_3), 50.3 (CH), 47.9 (C_q), 43.7 (CH), 38.2 (CH), 35.8 (CH_2), 31.5 (CH_2), 30.8 (CH_2), 29.9 (CH_2), 26.4 (CH_2), 25.9 (CH_2), 21.6 (CH_2), 19.2 (CH_2), 13.8 (CH_3), 13.8 (CH_3). IR (ATR): 2933, 2867, 1710, 1610, 1500, 1408, 1377, 1285, 1256, 1179 cm^{-1}. MS (ESI) *m*/*z* (relative intensity): 433 (100) $[M + Na]^+$, 411 (50) $[M + H]^+$. HR-MS (ESI): *m*/*z* calcd. for $[C_{26}H_{34}O_4 + Na]^+$ 433.2349 found 433.2342.

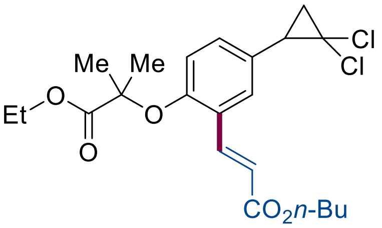

(*E*)-butyl-3-(5-(2,2-dichlorocyclopropyl)-2-((1-ethoxy-2-methyl-1-oxopropan-2-yl)oxy)phenyl)acrylate (64)

The general procedure **D** was followed using protected ciprofibrate (**53k**) (158.6 µL, 0.50 mmol, 1.0 equiv) and *n*-butyl acrylate (**3a**) (144.0 µL, 1.00 mmol, 2.0 equiv) at 80 °C for 48 h. **L12** was used as ligand. Isolation by column chromatography (*n*-hexane/EtOAc = 15:1) yielded **64** (75.4 mg, 34%) as sole product. The site selectivity was determined by ^{1}H-NMR and NOESY NMR. ^{1}H-NMR (300 MHz, $CDCl_3$): δ = 8.02 (d, *J* = 16.2 Hz, 1H), 7.40 (d, *J* = 2.3 Hz, 1H), 7.11 (dd, *J* = 8.5, 2.3 Hz, 1H), 6.71 (d, *J* = 8.6 Hz, 1H), 6.48 (d, *J* = 16.2 Hz, 1H), 4.27 – 4.17 (m, 4H), 2.83 (dd, *J* = 10.6, 8.3 Hz, 1H), 1.96 (dd, *J* = 10.6, 7.5 Hz, 1H), 1.79 (t, *J* = 7.9 Hz, 1H), 1.74 – 1.60 (m, 8H), 1.52 – 1.38 (m, 2H), 1.21 (t, *J* = 7.1 Hz, 3H), 0.97 (t, *J* = 7.3 Hz, 3H). ^{13}C-NMR (75 MHz, $CDCl_3$): δ = 173.9 (C_q), 167.3 (C_q), 153.8 (C_q), 139.7 (CH), 131.0 (CH), 128.7 (CH), 128.1 (C_q), 125.9 (C_q), 119.1 (CH), 116.83 (CH), 80.0 (C_q), 64.3 (CH_2), 61.6 (CH_2), 60.6 (C_q), 34.7 (CH), 30.8 (CH_2), 25.9 (CH_2), 25.4 (CH_3), 25.4 (CH_3), 19.2 (CH_2), 14.0 (CH_3), 13.8 (CH_3). IR (ATR): 2960, 2934, 1733, 1711, 1633, 1494, 1466, 1384, 1274, 1172 cm^{-1}. MS (ESI) *m*/*z* (relative intensity): 465 (100) $[M + Na]^+$, 443 (10) $[M + H]^+$. HR-MS (ESI): *m*/*z* calcd. for $[C_{22}H_{28}O_5Cl_2 + Na]^+$ 465.1206 found 465.1200.

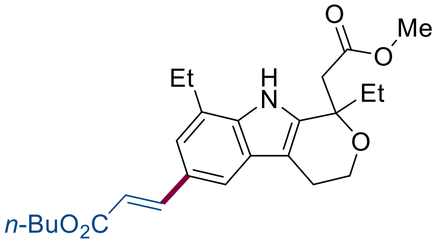

(*E*)−butyl-3-(1,8-diethyl-1-(2-methoxy-2-oxoethyl)-1,3,4,9-tetrahydropyrano[3,4-b]indol-6-yl)acrylate (65b)

The general procedure **D** was followed using protected etodolac (**53l**) (150.7 mg, 1.0 mmol, 5.0 equiv) and *n*-butyl acrylate (**3a**) (144.0 µL, 1.00 mmol, 2.0 equiv) at 80 °C for 48 h. **L12** was used as ligand. Isolation by column chromatography (*n*-hexane/EtOAc = 10:1) yielded **65** (77.0 mg, 36%). **65b** was separated while others couldn't be separated. The site selectivity was determined by ^{1}H-NMR. **65b** : others = 3:1. For **65b**, ^{1}H-NMR (400 MHz, $CDCl_3$): δ = 9.31 (s, 1H), 7.82 (d, *J* = 15.9 Hz, 1H), 7.54 (d, *J* = 1.6 Hz, 1H), 7.25 (d, *J* = 1.5 Hz, 1H), 6.42 (d, *J* = 15.9 Hz, 1H), 4.21 (t, *J* = 6.7 Hz, 2H), 4.09 – 4.02 (m, 1H), 3.97 – 3.90 (m, 1H), 3.73 (s, 3H), 3.07 – 2.71 (m, 6H), 2.15 (dq, *J* = 14.7, 7.4 Hz, 1H), 1.99 (dq, *J* = 14.6, 7.2 Hz, 1H), 1.75 – 1.65 (m, 2H), 1.52 – 1.42 (m, 2H), 1.38 (t, *J* = 7.6 Hz, 3H), 0.98 (t, *J* = 7.4 Hz, 3H), 0.84 (t, *J* = 7.4 Hz, 3H). ^{13}C-NMR (100 MHz, $CDCl_3$): δ = 173.4 (C_q), 167.9 (C_q), 146.8 (CH), 137.2

(C_q), 135.9 (C_q), 127.1 (C_q), 126.5 (C_q), 126.5 (C_q), 120.0 (CH), 117.8 (CH), 114.6 (CH), 109.3 (C_q), 74.5 (C_q), 64.1 (CH_2), 60.5 (CH_2), 52.1 (CH_3), 42.7 (CH_2), 30.9 (CH_2), 30.5 (CH_2), 24.1 (CH_2), 22.3 (CH_2), 19.3 (CH_2), 13.8 (CH_3), 13.6 (CH_3), 7.5 (CH_3). IR (ATR): 3366, 2981, 2875, 1737, 1630, 1465, 1445, 1372, 1163, 1046 cm^{-1}. MS (ESI) *m/z* (relative intensity): 450 (100) $[M + Na]^+$, 428 (10) $[M + H]^+$. HR-MS (ESI): *m/z* calcd. for $[C_{25}H_{33}NO_5 + Na]^+$ 450.2251 found 450.2253.

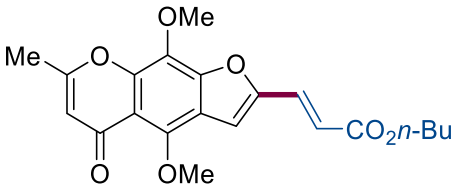

(*E*)-butyl-3-(4,9-dimethoxy-7-methyl-5-oxo-*5H*-furo[3,2-*g*]chromen-2-yl)acrylate (66)

The general procedure **D** was followed using khellin (**53m**) (136.9 mg, 0.5 mmol, 95%, 1 equiv.) and *n*-butyl acrylate (**3a**) (144.0 µL, 1.0 mmol, 2.0 equiv) at 80 °C for 48 h. **L12** was used as ligand. Isolation by column chromatography (*n*-hexane/EtOAc = 1:1) yielded product and starting material as an inseparable mixture. The product was purified by GPC to yield **66** (18.5 mg, 10%). The site selectivity was determined by ^{1}H-NMR and NOESY NMR. ^{1}H NMR (400 MHz, $CDCl_3$) δ 7.50 (d, *J* = 15.7 Hz, 1H), 7.14 (s, 1H), 6.60 (dd, *J* = 15.7, 0.6 Hz, 1H), 6.16 - 5.99 (m, 1H), 4.23 (t, *J* = 6.6 Hz, 2H), 4.20 (s, 3H), 4.05 (s, 3H), 2.39 (d, *J* = 0.7 Hz, 3H), 1.78 – 1.62 (m, 2H), 1.53 – 1.37 (m, 2H), 0.96 (t, *J* = 7.4 Hz, 3H) ppm. ^{13}C NMR (126 MHz, $CDCl_3$) δ 178.0 (C_q), 166.5 (C_q), 164.2 (C_q), 153.3 (C_q), 149.1 (C_q), 148.7 (C_q), 148.2 (C_q), 130.1 (CH), 129.7 (C_q), 120.5 (CH), 120.5 (C_q), 114.1 (C_q), 111.0 (CH), 109.2 (CH), 64.9 (CH_2), 62.7 (CH_3), 61.7 (CH_3), 30.8 (CH_2), 20.2 (CH_3), 19.3 (CH_2), 13.9 (CH_3) ppm. IR (ATR): 2959, 1715, 1663, 1637, 1619, 1483, 1365, 1297, 1162, 1064 cm^{-1}. HRMS (ESI): *m/z* calced for $[C_{21}H_{22}O_7+H^+]$ 387.1444, found 387.1438.

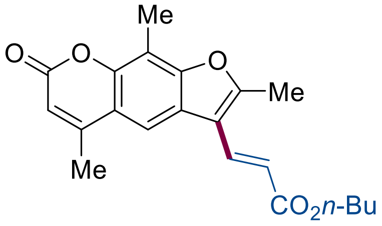

(*E*)-butyl-3-(2,5,9-trimethyl-7-oxo-*7H*-furo[3,2-*g*]chromen-3-yl)acrylate (67)

The general procedure **D** was followed using trioxsalen (**53n**) (115.3 mg, 0.5 mmol, 99%, 1 equiv.) and *n*-butyl acrylate (**3a**) (144.0 µL, 1.0 mmol, 2.0 equiv) at 80 °C for 48 h. **L12** was used as ligand. Isolation by column chromatography (*n*-hexane/EtOAc = 2:1) yielded **67** (97.4 mg, 55%). The site selectivity was determined by ^{1}H-NMR and NOESY NMR. ^{1}H-NMR (600 MHz, $CDCl_3$): δ = 7.78 (d, *J* = 16.0 Hz, 1H), 7.74 (s, 1H), 6.47 (d, *J* = 16.0 Hz, 1H), 6.29 (q, *J* = 1.3 Hz, 1H), 4.26 (t, *J* = 6.8 Hz, 2H), 2.63 (s, 3H), 2.59 (s, 3H), 2.55 (d, *J* = 1.3 Hz, 3H), 1.78 – 1.69 (m, 2H), 1.52 – 1.43 (m, 2H), 0.99 (t, *J* = 7.4 Hz, 3H). ^{13}C-NMR (126 MHz, $CDCl_3$): δ = 167.4 (C_q), 161.3 (C_q) 160.3 (C_q), 155.0 (C_q), 153.1 (C_q), 149.7 (C_q), 134.6 (CH), 122.6 (C_q), 117.6 (CH), 116.9 (C_q), 113.6 (CH), 112.8 (C_q), 112.6 (CH), 110.0 (C_q), 64.8 (CH_2), 31.0 (CH_2), 19.6 (CH_3), 19.4 (CH_2), 13.9 (CH_3), 13.2 (CH_3), 8.7 (CH_3). IR (ATR): 2960, 2931, 2875, 1731, 1714, 1637, 1599, 1293, 1166, 1102 cm^{-1}. HR-MS (ESI): *m/z* calced for [$C_{21}H_{22}O_5$+H] 355.1540, found 355.1535.

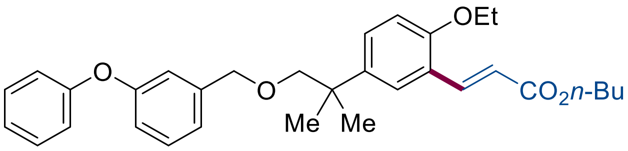

(*E*)-butyl-3-(2-ethoxy-5-(2-methyl-1-((3-phenoxybenzyl)oxy)propan-2-yl)phenyl)acrylate (68)

The general procedure **D** was followed using etofenprox (**53o**) (198.2 mg, 0.5 mmol, 95%, 1 equiv.) and *n*-butyl acrylate (**3a**) (144.0 µL, 1.0 mmol, 2.0 equiv) at 80 °C for 48 h. **L12** was used as ligand. Isolation by column chromatography (*n*-hexane/EtOAc = 15:1) yielded **68** (142.5 mg, 57%) and 25% other isomers. The site selectivity was determined by ^{1}H-NMR and NOESY NMR. ^{1}H-NMR (600 MHz, $CDCl_3$): *δ* = 8.00 (d, *J* = 16.1 Hz, 1H), 7.49 (d, *J* = 2.5 Hz, 1H), 7.37 – 7.30 (m, 3H), 7.30 – 7.25 (m, 1H), 7.15 – 7.08 (m, 1H), 7.02 – 6.99 (m, 2H), 6.98 (d, *J* = 7.7 Hz, 1H), 6.93 – 6.88 (m, 2H), 6.80 (d, *J* = 8.7 Hz, 1H), 6.53 (d, *J* = 16.1 Hz, 1H), 4.45 (s, 2H), 4.21 (t, *J* = 6.7 Hz, 2H), 4.07 (q, *J* = 7.0 Hz, 2H), 3.41 (s, 2H), 1.82 – 1.61 (m, 2H), 1.46 (t, *J* = 7.1 Hz, 5H), 1.32 (s, 6H), 0.97 (t, *J* = 7.4 Hz, 3H). ^{13}C-NMR (126 MHz, $CDCl_3$) δ = 167.9 (C_q), 157.5 (C_q), 157.3 (C_q), 156.1 (C_q), 141.0 (C_q), 140.9 (CH), 139.6 (C_q), 129.9 (CH), 129.7 (CH), 129.4 (CH), 126.9 (CH), 123.4 (CH), 122.9 (C_q), 122.1 (CH), 119.1 (CH), 118.4 (CH), 117.8 (CH), 117.7 (CH), 111.8 (CH), 80.2 (CH_2), 72.9 (CH_2), 64.4 (CH_2), 64.2 (CH_2), 38.7 (C_q), 31.0 (CH_2), 26.2 (CH_3), 19.4 (CH_2), 15.0 (CH_3), 13.9 (CH_3). IR (ATR):

2960, 2932, 2872, 1709, 1631, 1584, 1448, 1250, 1167, 1102 cm^{-1}. HR-MS (ESI): *m/z* calced for [$C_{32}H_{38}O_5$+Na] 525.2611, found 525.2605

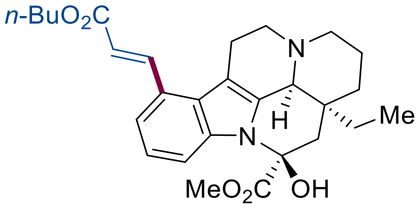

Methyl (4^1*S*,12*S*,13a*S*)-8-((*E*)-3-butoxy-3-oxoprop-1-en-1-yl)-13a-ethyl-12-hydroxy-2,3,4^1,5,6,12,13,13a-octahydro-*1H*-indolo[3,2,1-*de*]pyrido[3,2,1-*ij*][1,5]naphthyridine-12-carboxylate (69)

The general procedure **D** was followed using vincamine (**53p**) (180.8 mg, 0.5 mmol, 98%, 1.0 equiv.) and *n*-butyl acrylate (**3a**) (144.0 µL, 1.0 mmol, 2.0 equiv) at 80 °C for 48 h. **L12** was used as ligand. Isolation by column chromatography (*n*-hexane/EtOAc/NEt_3 = 1:1:0.1) yielded **69** (38.4 mg, 16%) and 11% other isomers. The site selectivity was determined by ^{1}H-NMR and NOESY NMR. ^{1}H-NMR (600 MHz, $CDCl_3$): δ = 8.39 (d, *J* = 15.8 Hz, 1H), 7.45 (dd, *J* = 6.1, 1.9 Hz, 1H), 7.13 – 7.09 (m, 2H), 6.46 (d, *J* = 15.8 Hz, 1H), 4.68 (s, 1H), 4.22 (, *J* = 6.7, 2H), 3.94 (s, 1H), 3.82 (s, 3H), 3.39 – 3.21 (m, 3H), 2.97 – 2.77 (m, 1H), 2.64 (dt, *J* = 11.5, 3.1 Hz, 1H), 2.58 – 2.46 (m, 1H), 2.31 – 2.22 (m, 1H), 2.21 (d, *J* = 14.2 Hz, 1H), 2.14 (d, *J* = 14.3 Hz, 1H), 1.83 – 1.57 (m, 4H), 1.55 – 1.35 (m, 5H), 0.97 (t, *J* = 7.4 Hz, 3H), 0.91 (t, *J* = 7.6 Hz, 3H). ^{13}C-NMR (126 MHz, $CDCl_3$): δ = 174.4 (C_q), 167.6 (C_q), 142.7 (CH), 134.9 (C_q), 132.9 (C_q), 128.2 (C_q), 127.7 (C_q), 121.9 (CH), 118.4 (CH), 118.1 (CH), 112.2 (CH), 106.0 (C_q), 82.0 (C_q), 64.4 (CH_2), 59.4 (CH), 54.5 (CH_3), 51.0 (CH_2), 44.6 (CH_2), 44.5 (CH_2), 35.2 (C_q), 30.9 (CH_2), 28.9 (CH_2), 25.1 (CH_2), 20.7 (CH_2), 20.0 (CH_2), 19.4 (CH_2), 13.9 (CH_3), 7.6 (CH_3). IR (ATR): 2956, 2872, 1738, 1705, 1628, 1436, 1290, 1255, 1165, 732 cm^{-1}. HR-MS (ESI): *m/z* calced for [$C_{28}H_{36}N_2O_5$+H] 481.2702, found 481.2697

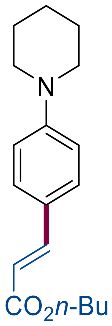

(*E*)-*n*-butyl-3-(4-(piperidin-1-yl)phenyl)acrylate (*p*-S1) & (*E*)-*n*-butyl-3-(2-(piperidin-1-yl)phenyl)acrylate (*o*-S1)

The general procedure **A** was followed using 1-phenylpiperidine (**2p**) (161.7 µL, 1.0 mmol, 5.0 equiv) and *n*-butyl acrylate (**2a**) (28.8 µL, 0.20 mmol, 1.0 equiv) at 60 °C for 20 h. **L12** was used as ligand and Fe was used as anode. Isolation by column chromatography (*n*-hexane/EtOAc = 15:1) yielded **S1** (12.5 mg, 22%). The *o* and *p*-olefinated products are unknown compounds and the ratio of the isomers was determined by the ^{1}H-NMR of the product mixture, *o* : *p* = 4.2 : 1. For *p*-**S1**, ^{1}H-NMR (600 MHz, $CDCl_3$): δ = 7.60 (d, *J* = 15.9 Hz, 1H), 7.44 – 7.38 (m, 2H), 6.88 – 6.85 (m, 2H), 6.25 (d, *J* = 15.9 Hz, 1H), 4.18 (t, *J* = 6.7 Hz, 2H), 3.29 – 3.26 (m, 4H), 1.72 – 1.64 (m, 6H), 1.64 – 1.60 (m, 2H), 1.48 – 1.40 (m, 2H), 0.96 (t, *J* = 7.4 Hz, 3H). ^{13}C-NMR (125 MHz, $CDCl_3$): δ = 167.9 (C_q), 153.0 (C_q), 144.7 (CH), 129.6 (CH), 124.1 (C_q), 114.9 (CH), 113.7 (CH), 64.1 (CH_2), 49.2 (CH_2), 30.9 (CH_2), 25.5 (CH_2), 24.3 (CH_2), 19.2 (CH_2), 13.8 (CH_3). For *o*-**S1**, ^{1}H-NMR (600 MHz, $CDCl_3$): δ = 8.07 (d, *J* = 16.2 Hz, 1H), 7.54 – 7.51 (m, 1H), 7.32 (ddd, *J* = 8.1, 7.3, 1.6 Hz, 1H), 7.05 – 6.99 (m, 2H), 6.39 (d, *J* = 16.2 Hz, 1H), 4.21 (t, *J* = 6.6 Hz, 2H), 2.90 (t, *J* = 5.3 Hz, 4H), 1.79 – 1.73 (m, 4H), 1.73 – 1.66 (m, 2H), 1.61 – 1.56 (m, 2H), 1.50 – 1.43 (m, 2H), 0.97 (t, *J* = 7.4 Hz, 3H). ^{13}C-NMR (125 MHz, $CDCl_3$): δ = 167.6 (C_q), 153.9 (C_q), 142.4 (CH), 130.7 (CH), 128.7 (C_q), 127.7 (CH), 122.3 (CH), 119.0 (CH), 117.3 (CH), 64.2 (CH_2), 54.3 (CH_2), 30.8 (CH_2), 26.3 (CH_2), 24.2 (CH_2), 19.3 (CH_2), 13.8 (CH_3). MS (ESI) *m*/*z* (relative intensity): 310 (10) $[M + Na]^+$, 288 (100) $[M + H]^+$. HR-MS (ESI): *m*/*z* calcd. for $[C_{18}H_{25}O_2N + Na]^+$ 288.1958 found 288.1960.

KIE Studies

1) Procedure for **L12** KIE study (Intermolecular competition reaction)

General procedure was followed using **1d** (5.0 equiv), [D]$_8$-**1d** (5.0 equiv), *n*-butyl acrylates **3a** (0.20 mmol, 1.0 equiv), Pd(OAc)$_2$ (4.5 mg, 10 mol %), **L12** (7.9 mg, 20 mol %), 1,4-benzoquinone (4.3 mg, 20 mol %), NaOAc (66.0 mg, 4.0 equiv), AcOH (2.6 mL) and HFIP (1.3 mL) in the anodic chamber, 1,4-Benzoquinone (4.3 mg, 20 mol %) and NaOAc (66.0 mg, 4.0 equiv), AcOH (2.6 mL) and HFIP (1.3 mL) in the cathodic chamber. Electrocatalysis was performed at 60 °C with a constant current of 1.0 mA and a stirring rate of 500 rpm maintained for 20 h.

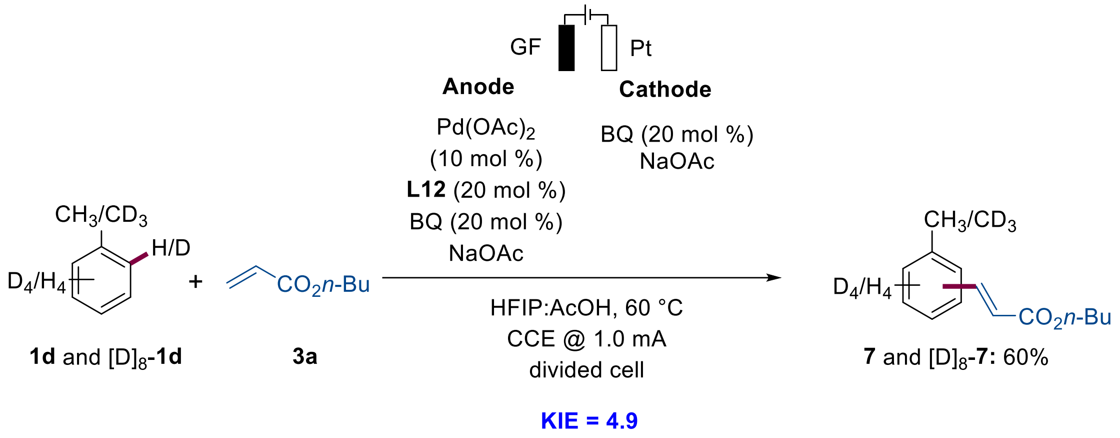

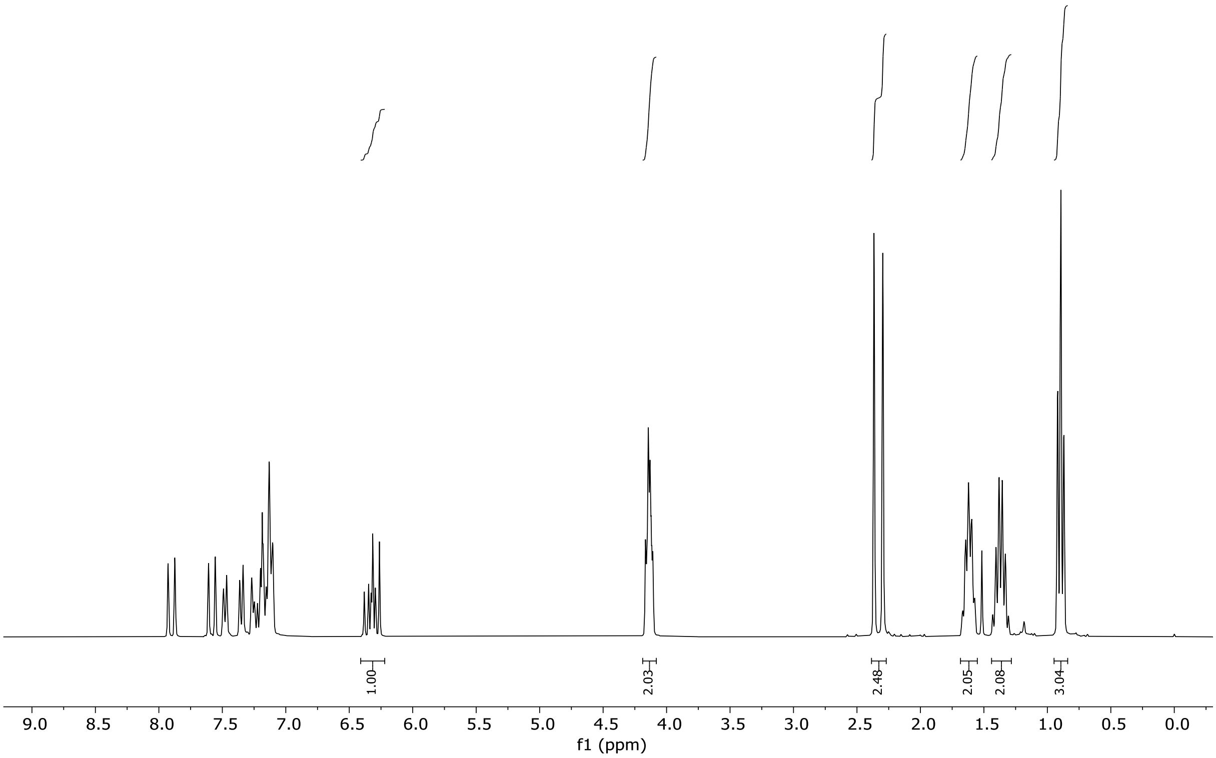

Supplementary Figure 25 KIE study of L12 by intermolecular competition reaction.

Note: CH_3 of aromatic and CH_3 of butyl group should be compared.

2) Procedure for **L3** KIE study (Intermolecular competition reaction)

General procedure was followed using **1h** (10 equiv), $[D]_{10}$-**1h** (10 equiv), *n*-butyl acrylates **3a** (0.20 mmol, 1.0 equiv), $Pd(OAc)_2$ (4.5 mg, 10 mol %), **L3** (6.5 mg, 20 mol %), 1,4-benzoquinone (4.3 mg, 20 mol %), NaOAc (66.0 mg, 4.0 equiv), AcOH (2.6 mL) and HFIP (1.3 mL) in the anodic chamber, 1,4-Benzoquinone (4.3 mg, 20 mol %) and NaOAc (66.0 mg, 4.0 equiv), AcOH (2.6 mL) and HFIP (1.3 mL) in the cathodic chamber. Electrocatalysis was performed at 60 °C with a constant current of 1.0 mA and a stirring rate of 500 rpm maintained for 20 h.

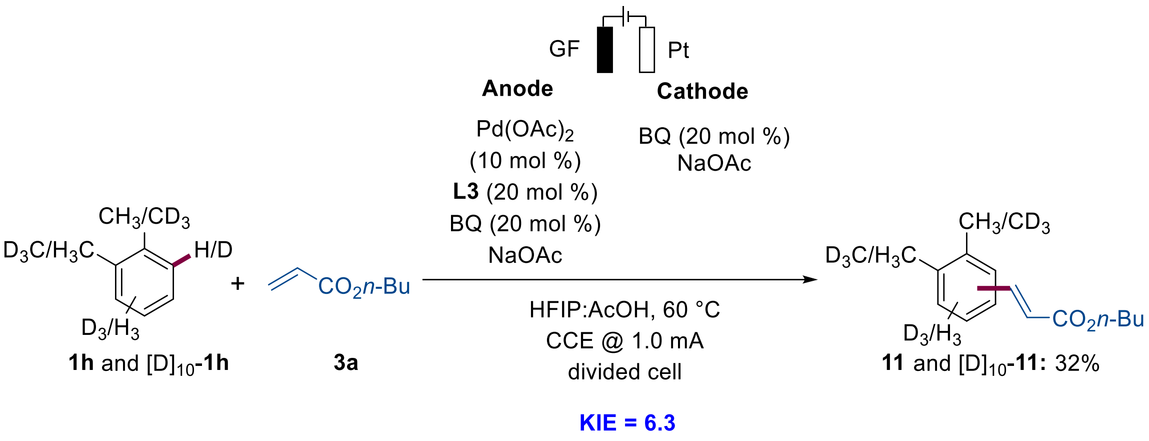

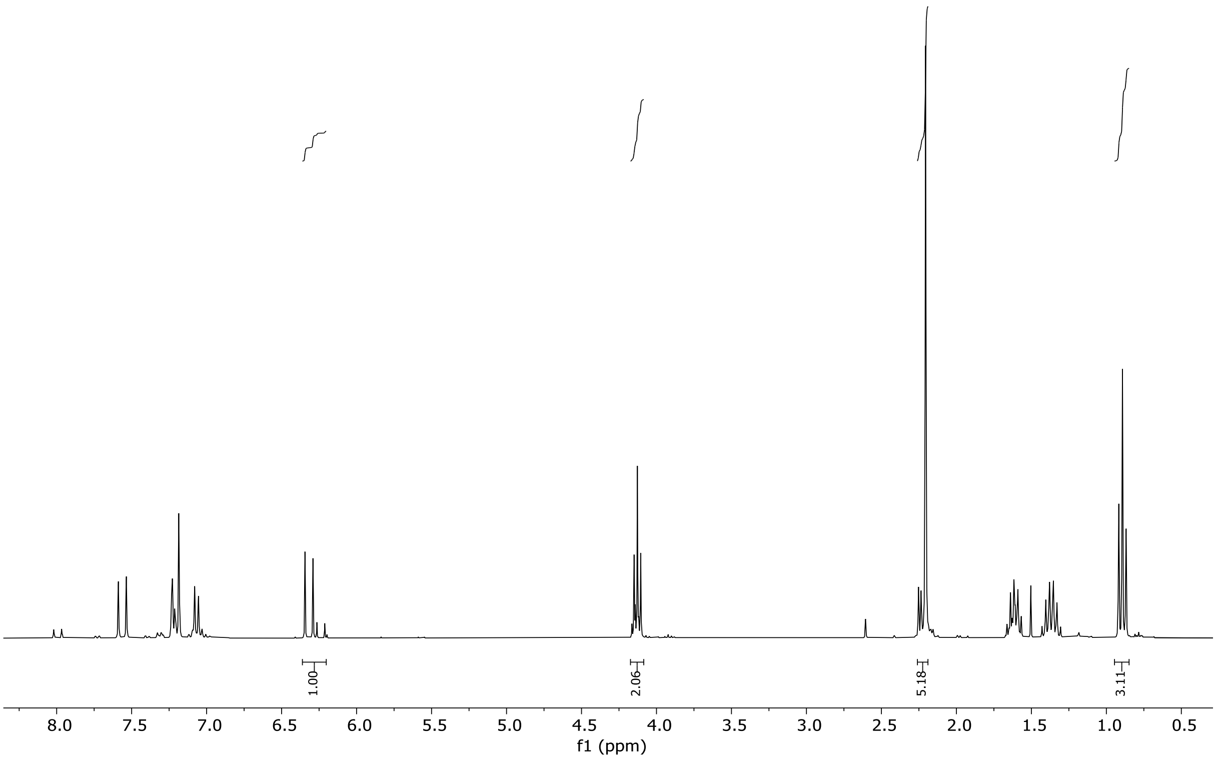

Supplementary Figure 26 KIE study of L3 by intermolecular competition reaction.

Synthesis of the Palladium Complex

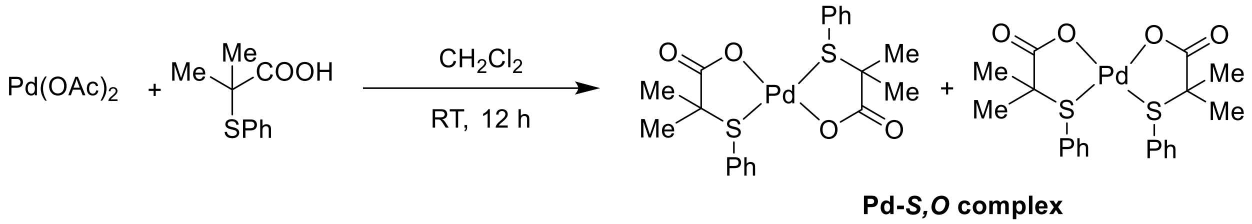

Supplementary Figure 27 Synthesis of palladium complex

A solution of 2-methyl-2-(phenylthio)propanoic acid (**L12**) (49.0 mg, 0.25 mmol, 2.0 equiv) and $Pd(OAc)_2$ (28.0 mg, 0.125 mmol, 1.0 equiv) in CH_2Cl_2 (10 mL) was stirred at room temperature overnight. The reaction mixture was filtrated through a pad of Celite and concentrated. Excess amount of Et_2O was added in the reaction mixture and filtrated. The filtrate was evaporated under air to crystalize and afford **Pd-*S,O* complex**. ^{1}H-NMR (400 MHz, $CDCl_3$): δ = 8.04 – 7.13 (m, 10H), 1.83 (m, 6H), 1.22 (t, J = 8.8 Hz, 6H). MS (ESI) m/z (relative intensity): 518 (100) $[M + Na]^+$, 497 (50) $[M + 2H]^+$. HR-MS (ESI): m/z calcd. for $[C_{20}H_{22}O_4PdS_2 + Na]^+$ 518.9893 found 518.9890. The analytical data correspond with those reported in the literature.[4]

Computational Methods

All DFT calculations were performed with Gaussian 16, Revision A.03 package.[53] Geometry optimizations were performed at the PBE0[54,55] level of theory including D3 dispersion corrections with a Becke-Johnson damping scheme (D3BJ)[56,57] in the gas phase. Analytical frequency calculations were carried out at the same level of theory to identify all stationary points as either minima (zero imaginary frequencies) or as transition states (one imaginary frequency). All atoms were described with a def2-SVP basis set,[57-60] while palladium was also described with a SSD pseudopotential.[61,62] The electronic energy was then further refined through single point calculations at the PBE0, PW6B95[63] or ωB97XD[64] level of theory including Grimme's D4 dispersion corrections[65,66] for PBE0 and PW6B95 functionals with a def2-TZVP basis set combined with SSD pseudopotential for palladium.[61,62] Solvent effects were taken into account through the use of steered molecular dynamics (SMD) solvent model[67] with a dielectric constant of $\varepsilon = 6.2528$, which corresponds to acetic acid as implemented in Gaussian 16. All reported energies are based on single electronic energies with addition of the gas-phase thermal and non-thermal corrections at 333.15 K and 1 atm.

Wiberg bond order analysis was performed using natural population analysis which is native to Gaussian 16 at the PBE0-D3(BJ)/def2-TZVP level of theory.

To better understand the *o*-xylene C–H activation elementary step, three possible pathways were probed by means of DFT calculations at the PBE0-D4/def2-TZVP+SMD(AcOH)//PBE0-D3BJ/def2-SVP level of theory (Supplementary Figure 28). The difference between each path rely on the nature of the *S*,*O*-ligand coordination to the metal center. In the neutral pathway, the *S*,*O*-ligand underwent deprotonation prior to the coordination to the palladium (Supplementary Figure 28a). C–H activation was proven to be facile with a barrier of 11.7 kcal mol^{-1}. Alternatively the C–H cleavage in the absence of acetate was also considered (Supplementary Figure 28b). Such featured a considerably prohibitive barrier of 50.7 kcal mol^{-1}, providing support for an acetate assisted C–H cleavage mechanism. Additionally, the direct ligation of the *S*,*O*-ligand to the palladium center was also taken into consideration (Supplementary Figure 28c). Here both C_α–H and C_β–H activation paths were probed, which presented associated barriers of 10.7 (TS(1-2)'$^{\alpha}$) and 10.3 kcal mol^{-1} (TS(1-2)'$^{\beta}$) respectively. Therefore, the pathway leading to the formation of the β product was shown to be slightly kinetically preferred. This catalyst mode of action was proven to be the most energetically favored pathway. Other DFT functionals (PW6B95, Supplementary Figure 29 and wB97XD,

Supplementary Figure 30) were also accounted for, which further confirmed that the non-directed pallada-electrocatalyzed C–H activations follow a cationic pathway.

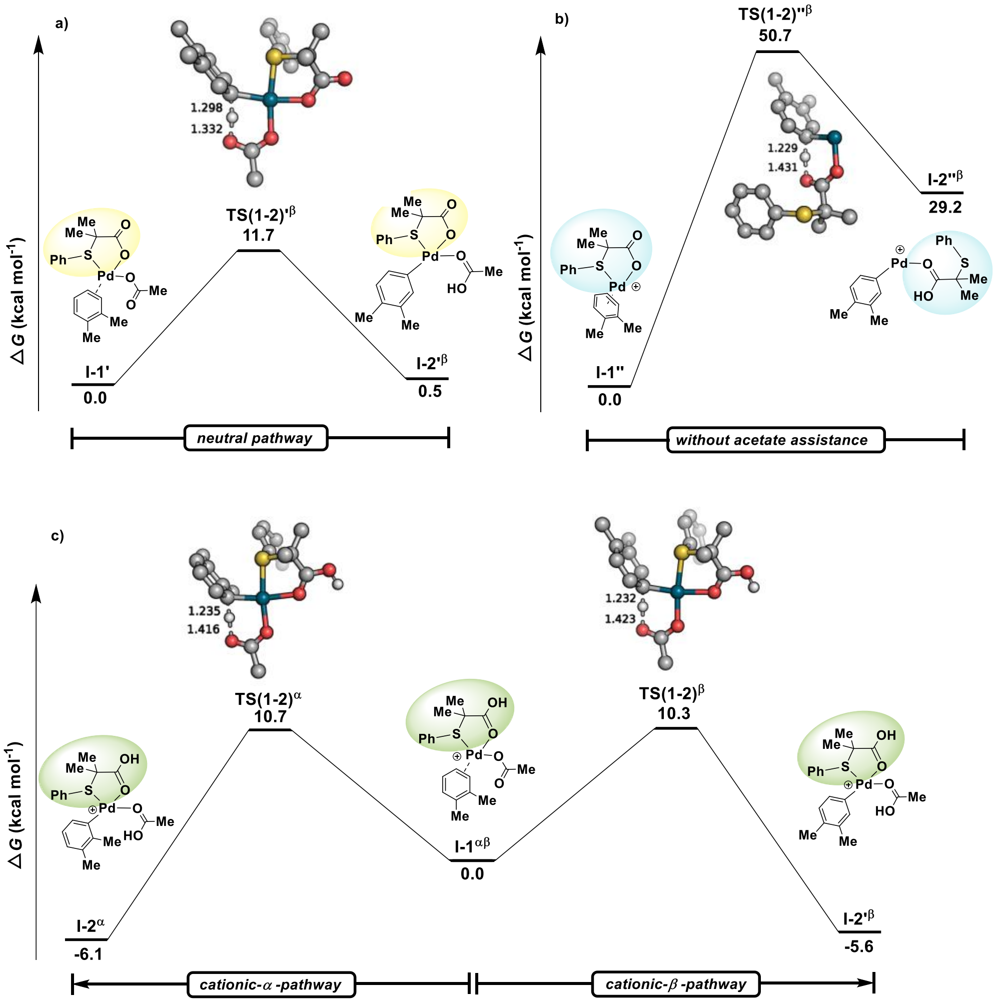

Supplementary Figure 28 Computed relative Gibbs free energy profile ($\Delta G_{333.15}$) in kcal mol^{-1} for *o*-xylene with three possible C–H activation pathways. a) neutral pathway; b) without the assistance of acetate; c) cationic α and β pathways at the PBE0-D4/def2-TZVP+SMD(AcOH)//PBE0-D3BJ/def2-SVP level of theory. Non-participating hydrogen atoms in the transition state structures were omitted for clarity, with bond lengths given in Å.

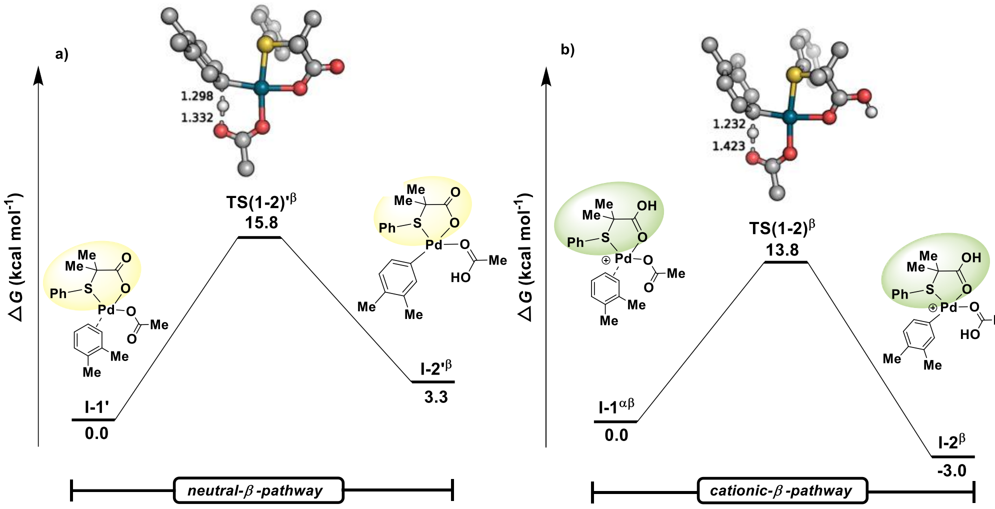

Supplementary Figure 29 Computed relative Gibbs free energy profile ($\Delta G_{333.15}$) in kcal mol^{-1} for *o*-xylene. a) neutral-α-pathway and b) cationic-β-pathways at the PW6B95-D4/def2-TZVP+SMD(AcOH)//PBE0-D3BJ/def2-SVP level of theory. Non-participating hydrogen atoms in the transition state structures were omitted for clarity, with bond lengths given in Å.

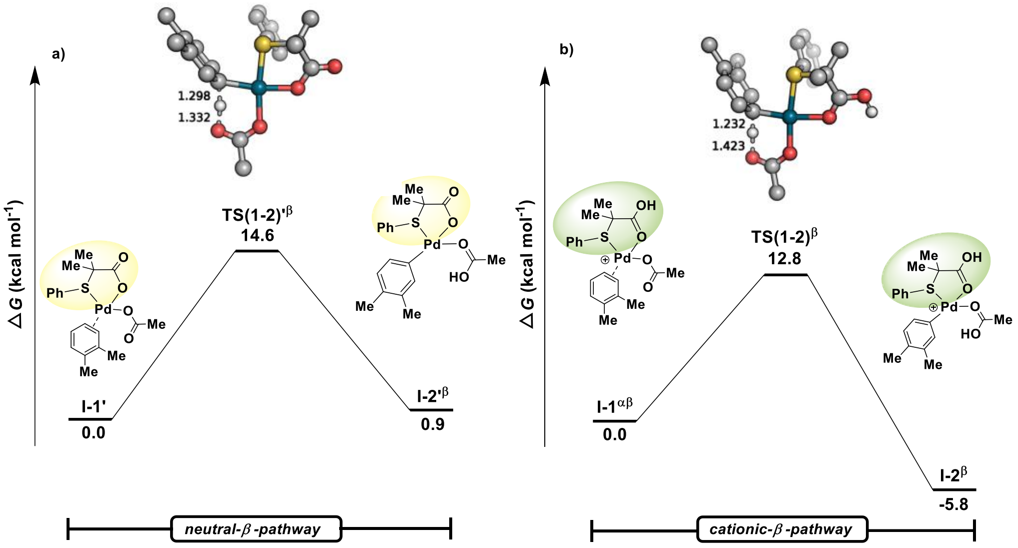

Supplementary Figure 30 Computed relative Gibbs free energy profile ($\Delta G_{333.15}$) in kcal mol^{-1} for *o*-xylene. a) neutral-α-pathway and b) cationic-β-pathways at the ωB97XD/def2-TZVP+SMD(AcOH)//PBE0-D3BJ/def2-SVP level of theory.

Non-participating hydrogen atoms in the transition state structures were omitted for clarity, with bond lengths given in Å.

A More O'Ferrall-Jencks analysis on the nature of the C–H cleavage was further carried out for the *o*-xylene Cα–H and Cβ–H pathways. Such provided strong support for C–H activation to occur through a based-assisted internal electrophilic substitution (BIES) mechanism.

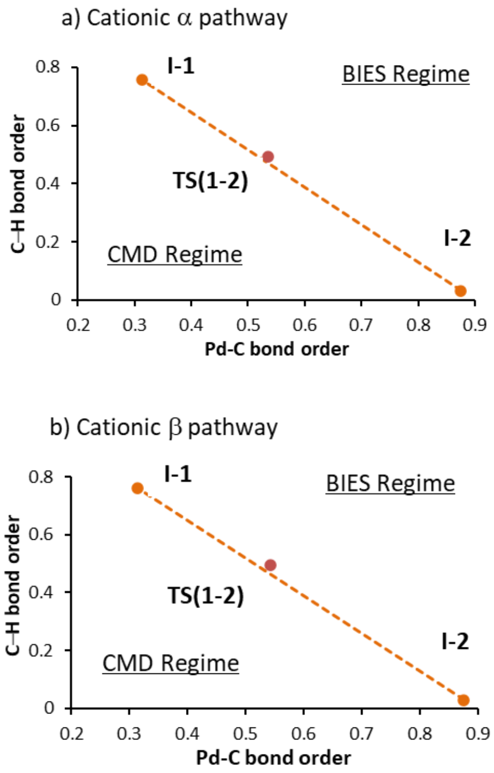

Supplementary Figure 31 Wiberg bond order analysis. Wiberg bond order analysis of the C–H activation step for α (a) and β (b) pathways.

In order to gain better insights into the regioselectivity of anisole, density functional theory (DFT) calculations were carried out for the C–H activation, insertion and *β*-H elimination elementary steps for *ortho*- and *para*-functionalized products. Both profiles show that the C–H activation was the rate-determining step, with energy barrier of 9.7 kcal mol^{-1} for *ortho*-product and 10.7 kcal mol^{-1} for *para*-product, which is consistent with the KIE results (Supplementary Figure 25). Later NCIPLOT analysis revealed the presence of attraction interaction between the anisole methoxy group and *S*,*O*-ligand phenyl group contributes to the preference for *ortho* product.

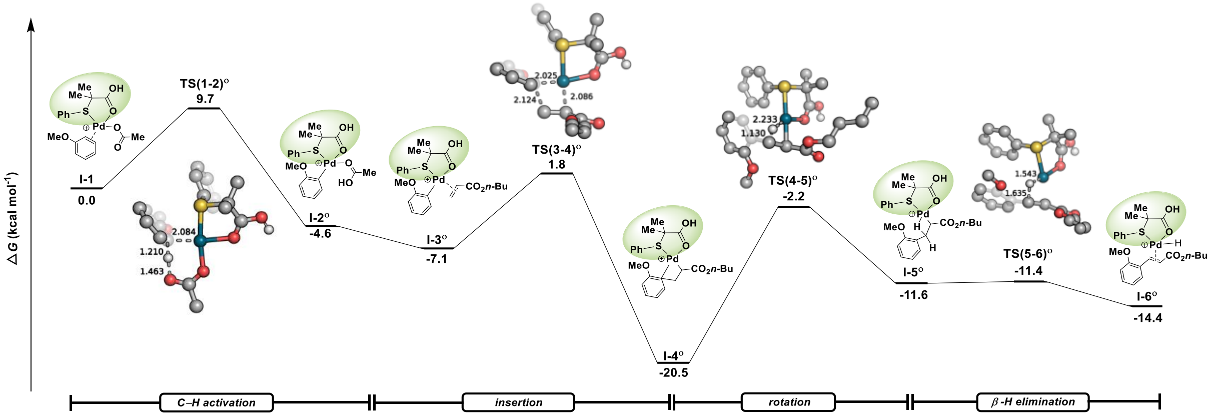

Supplementary Figure 32 Computed relative Gibbs free energy profile ($\Delta G_{333.15}$) in kcal mol^{-1} for anisole cationic pathways to get ortho product at the PBE0-D4/def2-TZVP+SMD(AcOH)//PBE0-D3BJ/def2-SVP level of theory. Non-participating hydrogen atoms in the transition state structures were omitted for clarity, with bond lengths given in Å.

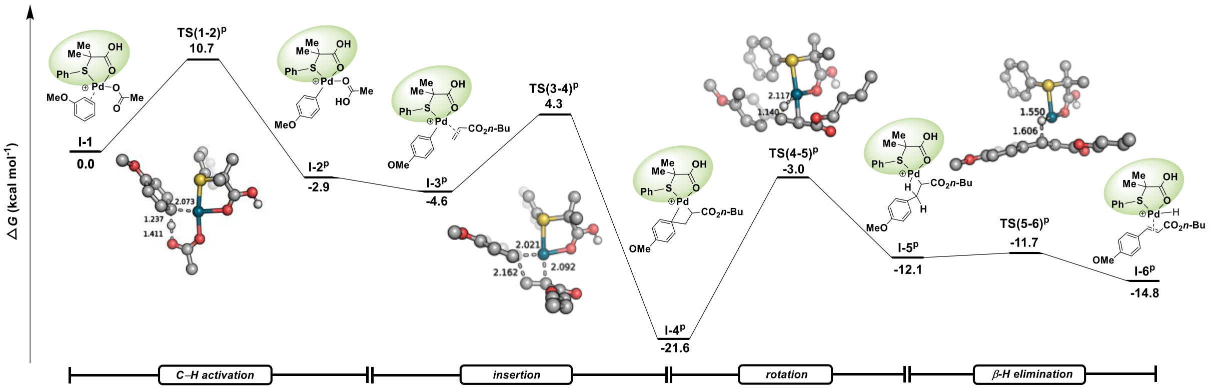

Supplementary Figure 33 Computed relative Gibbs free energy profile ($\Delta G_{333.15}$) in kcal mol^{-1} for anisole cationic pathways to get para product at the PBE0-D4/def2-TZVP+SMD(AcOH)//PBE0-D3BJ/def2-SVP level of theory. Non-participating hydrogen atoms in the transition state structures were omitted for clarity, with bond lengths given in Å.

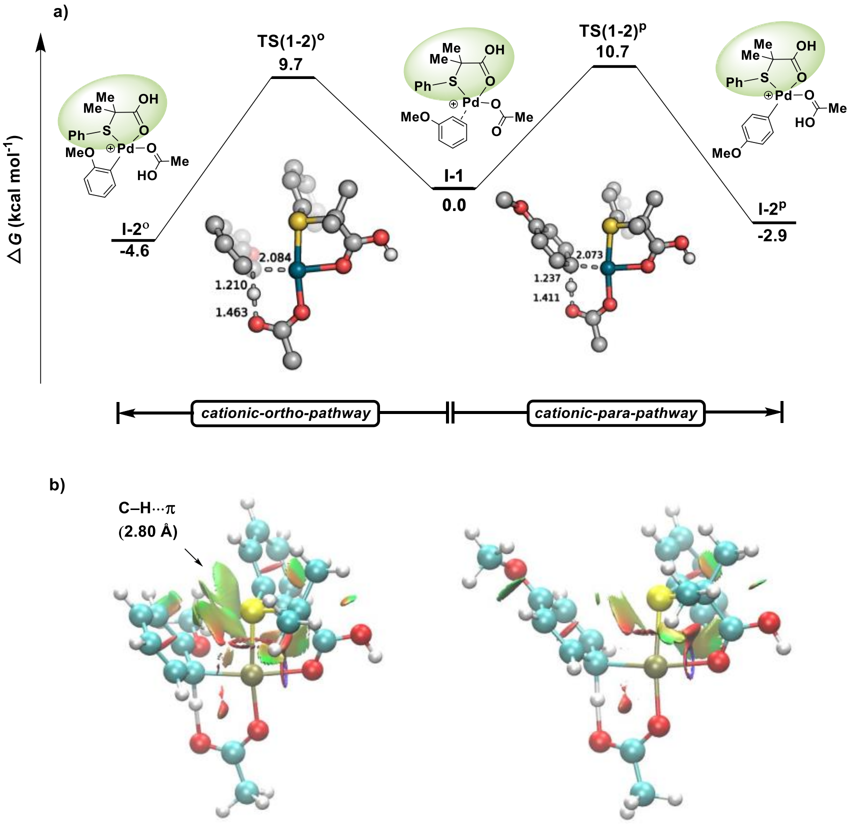

Supplementary Figure 34 Computed relative Gibbs free energy profile and Non-covalent interaction plots. a) Computed relative Gibbs free energy profile ($\Delta G_{333.15}$) in kcal mol^{-1} for cationic C–H activation pathways at the PBE0-D4/def2-TZVP+SMD(AcOH)//PBE0-D3BJ/def2-SVP level of theory. Non-participating hydrogen atoms in the transition state structures were omitted, with bond lengths given in Å. b) Non-covalent interaction plots for the TS(1-2)^{ortho} and TS(1-2)^{para}.

Supplementary Table 27 Calculated electronic energies for anisole cationic pathways at the PBE0 D4/def2 TZVP+SMD(AcOH) level of theory and Gibbs free energies with dispersion corrections for all structures (all in Hartree).

Structure	Electronic Energy	Total Gibbs Free Energy	Imaginary Frequency
I-1	-1639.093971	-1638.772166	
TS(1-2)^{o}	-1639.075726	-1638.756720	-106.72i
I-2°	-1639.099272	-1638.779469	
I-3°	-1834.263116	-1833.833091	
TS(3-4)^{o}	-1834.247767	-1833.818921	-239.23i
I-4°	-1834.287186	-1833.854442	
TS(4-5)^{o}	-1834.256167	-1833.825201	-118.02i
I-5°	-1834.269301	-1833.840217	
TS(5-6)^{o}	-1834.266473	-1833.839987	-348.79i
I-6°	-1834.272705	-1833.844763	
TS(1-2)^{p}	-1639.071488	-1638.755138	-205.33i
I-2^{p}	-1639.094692	-1638.776853	
I-3^{p}	-1834.259236	-1833.829151	
TS(3-4)^{p}	-1834.242206	-1833.814927	-236.48i
I-4^{p}	-1834.286959	-1833.856271	
TS(4-5)^{p}	-1834.256632	-1833.826536	-234.60i
I-5^{p}	-1834.266798	-1833.841058	
TS(5-6)^{p}	-1834.264526	-1833.840489	-384.31i
I-6^{p}	-1834.273677	-1833.845311	
Acetic Acid	-228.937746	-228.906343	
Anisole	-424.094301	-423.955947	

Supplementary Table 28 Calculated electronic energies for o-xylene at the PBE0 D4/def2 TZVP+SMD(AcOH) level of theory and Gibbs free energies with dispersion corrections for all structures (all in Hartree).

Structure	Electronic Energy	Total Gibbs Free Energy	Imaginary Frequency
I-1’	-1602.780710	-1602.450299	
TS(1-2)’$^{\beta}$	-1602.755194	-1602.431606	-744.29i
I-2’$^{\beta}$	-1602.776783	-1602.449541	
I-1$^{\alpha\beta}$	-1603.213915	-1602.870474	
TS(1-2)$^{\alpha}$	-1603.193363	-1602.853494	-209.15i
TS(1-2)$^{\beta}$	-1603.192459	-1602.854105	-196.66i
I-2$^{\alpha}$	-1603.219708	-1602.880148	

I-2$^{\beta}$	-1603.219399	-1602.879477	
I-1''	-1374.252790	-1373.968194	
TS(1-2)''$^{\beta}$	-1374.165923	-1373.887452	-320.20i
I-2''$^{\beta}$	-1374.203983	-1373.921725	

Supplementary Table 29 Calculated electronic energies for o-xylene at the PW6B95 D4/def2 TZVP+SMD(AcOH) level of theory and Gibbs free energies with dispersion corrections for all structures (all in Hartree).

Structure	Electronic Energy	Total Gibbs Free Energy
I-1'	-1605.913081	-1605.581456
TS(1-2)'$^{\beta}$	-1605.879848	-1605.556260
I-2'$^{\beta}$	-1605.903407	-1605.576165
I-1$^{\alpha\beta}$	-1606.344439	-1606.000998
TS(1-2)$^{\beta}$	-1606.317404	-1605.979050
I-2$^{\beta}$	-1606.345668	-1606.005746

Supplementary Table 30 Calculated electronic energies for o-xylene at the □B97XD D4/def2 TZVP+SMD(AcOH) level of theory and Gibbs free energies with dispersion corrections for all structures (all in Hartree).

Structure	Electronic Energy	Total Gibbs Free Energy
I-1’	-1603.801981	-1603.471570
TS(1-2)’$^{\beta}$	-1603.771547	-1603.447959
I-2’$^{\beta}$	-1603.797176	-1603.469934
I-1$^{\alpha\beta}$	-1604.236726	-1603.893285
TS(1-2)$^{\beta}$	-1604.211188	-1603.872834
I-2$^{\beta}$	-1604.242379	-1603.902457

Supplementary Table 31 Bond orders for the *o*-xylene cationic I-1, TS(1-2) and I-2.

Structure	Pd-C bond order	C–H bond order
I-1$^{\alpha\beta}$	0.3131	0.8755
TS(1-2)$^{\alpha}$	0.4915	0.5354
I-2$^{\alpha}$	0.7569	0.0316
TS(1-2)$^{\beta}$	0.4956	0.5435
I-2$^{\beta}$	0.7631	0.0284

NMR Spectrum

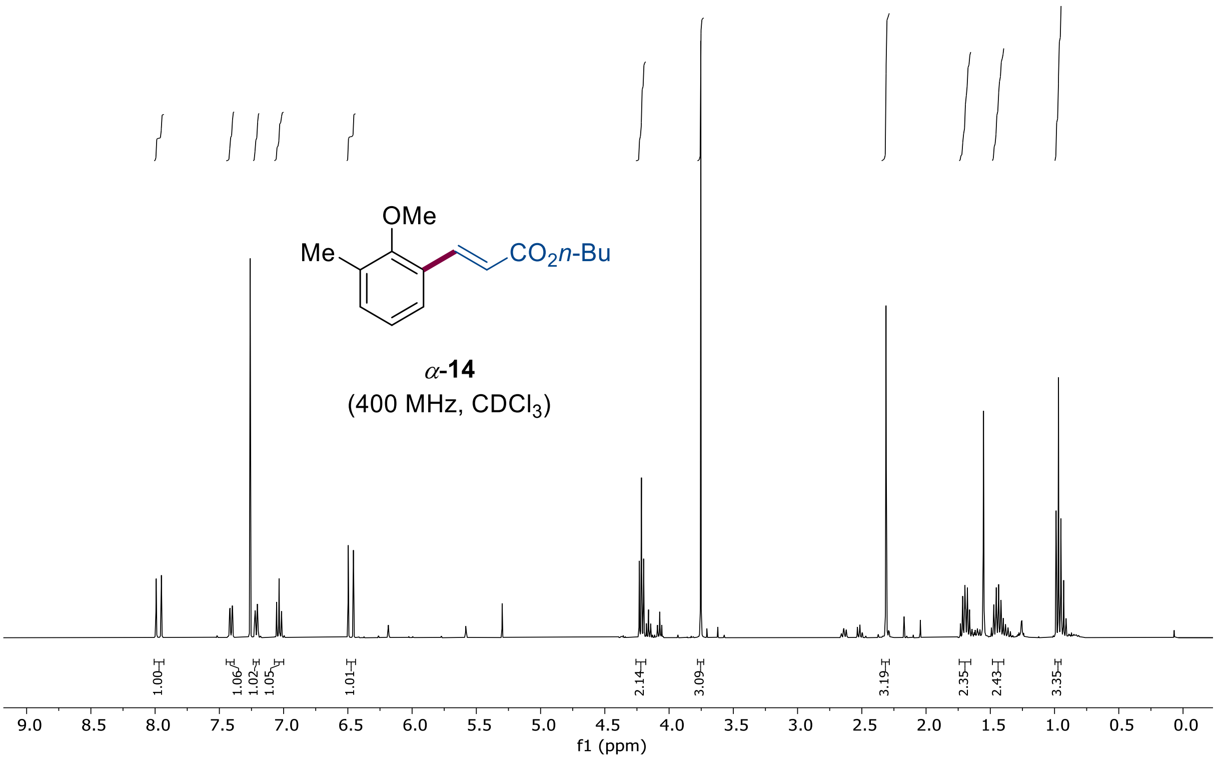

Supplementary Figure 35 H-NMR of compound *α*-14. 400 MHz, $CDCl_3$, RT

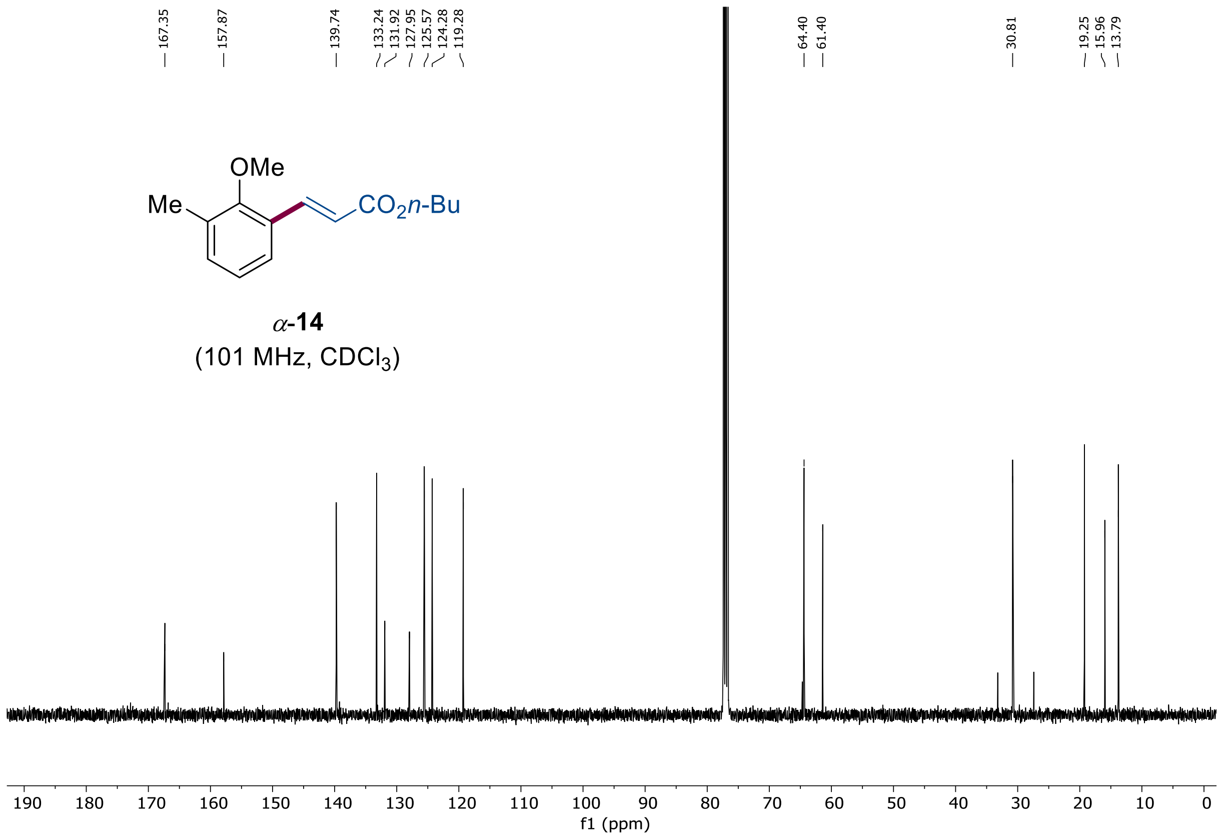

Supplementary Figure 36 C-NMR of compound *α*-14, 101 MHz, $CDCl_3$, RT

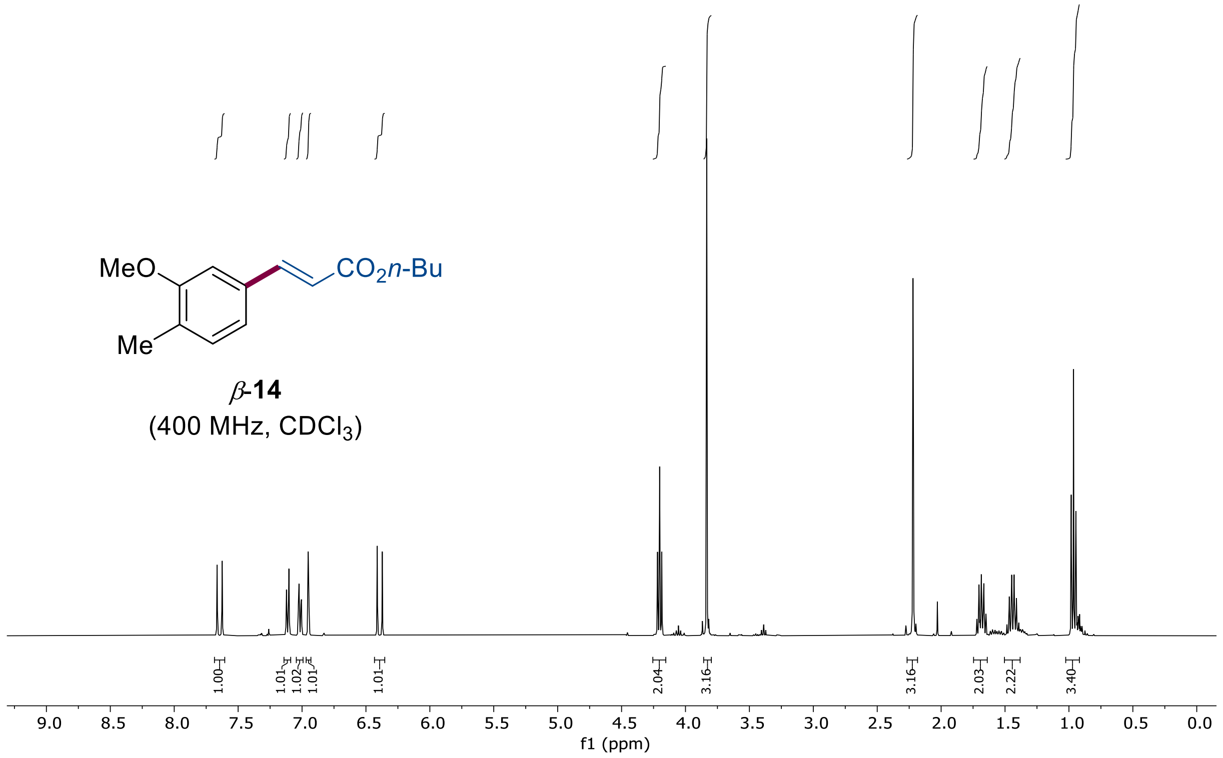

Supplementary Figure 37 H-NMR of compound *β*-14. 400 MHz, $CDCl_3$, RT

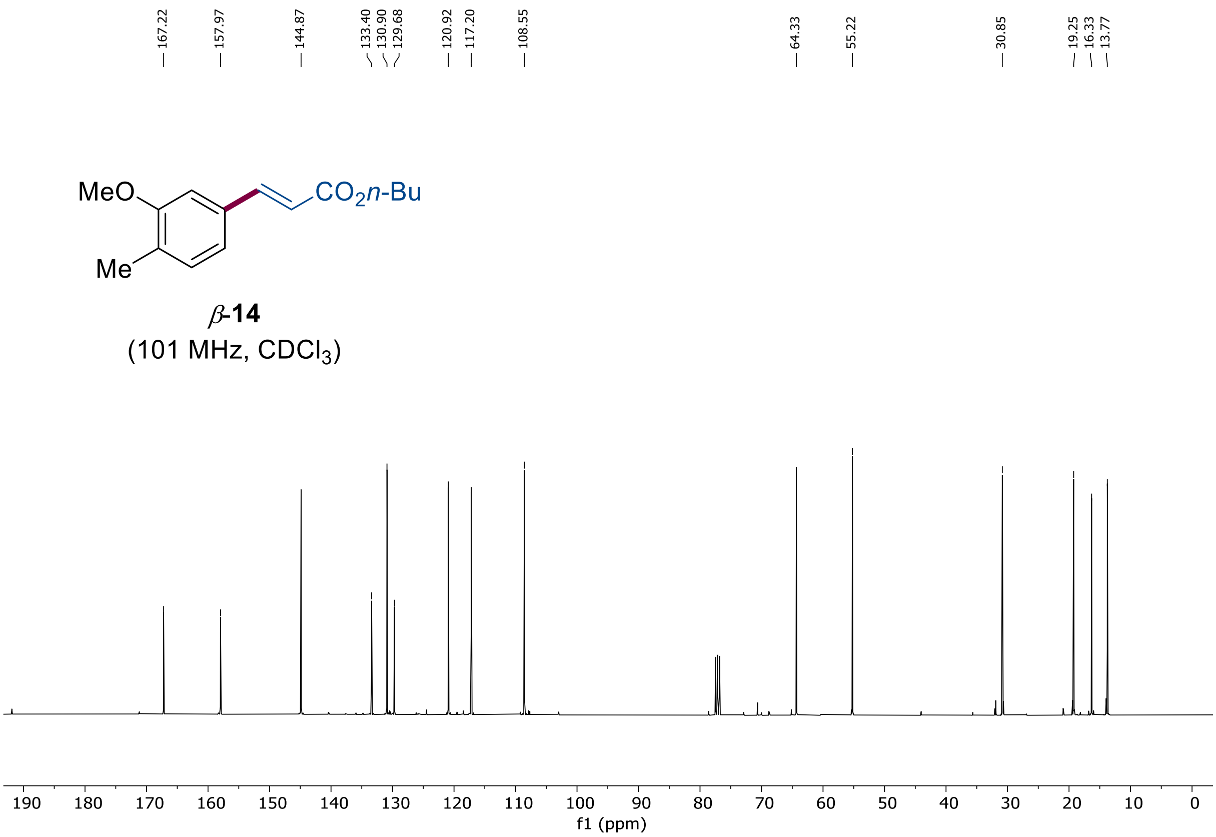

Supplementary Figure 38 C-NMR of compound β-14. 101 MHz, $CDCl_3$, RT

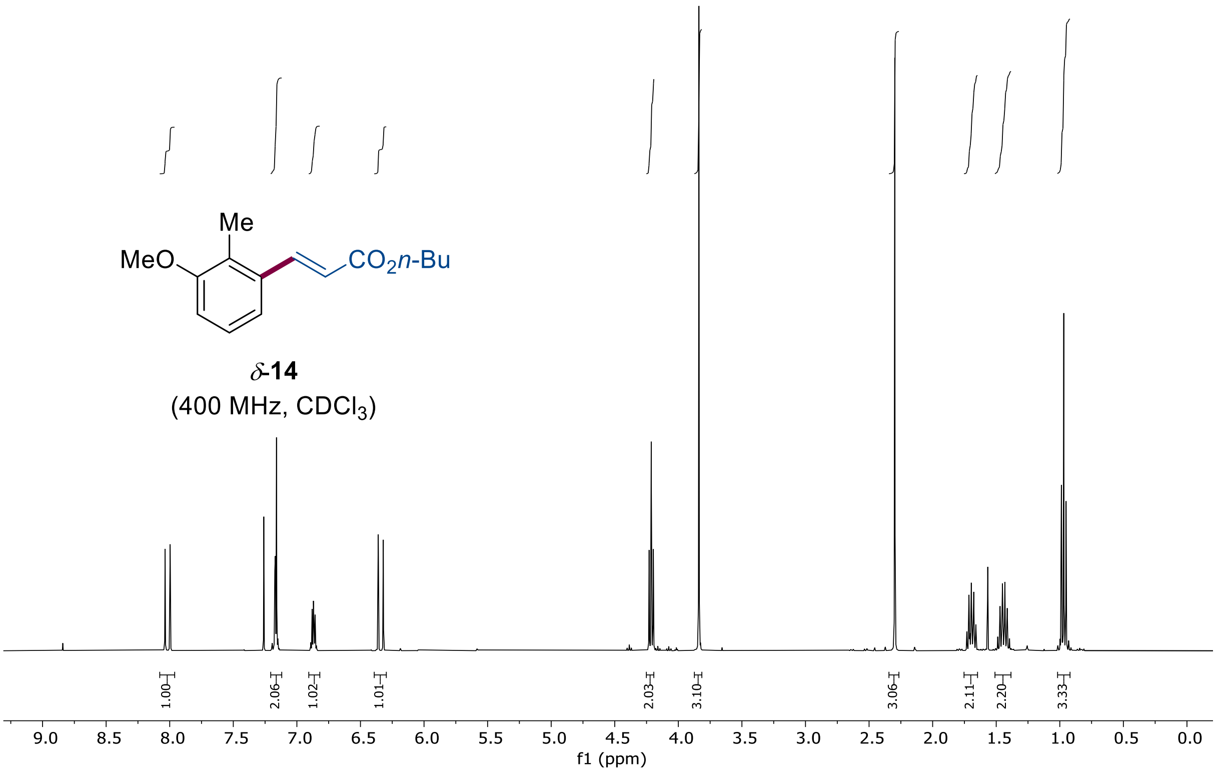

Supplementary Figure 39 H-NMR of compound δ-14. 400 MHz, $CDCl_3$, RT

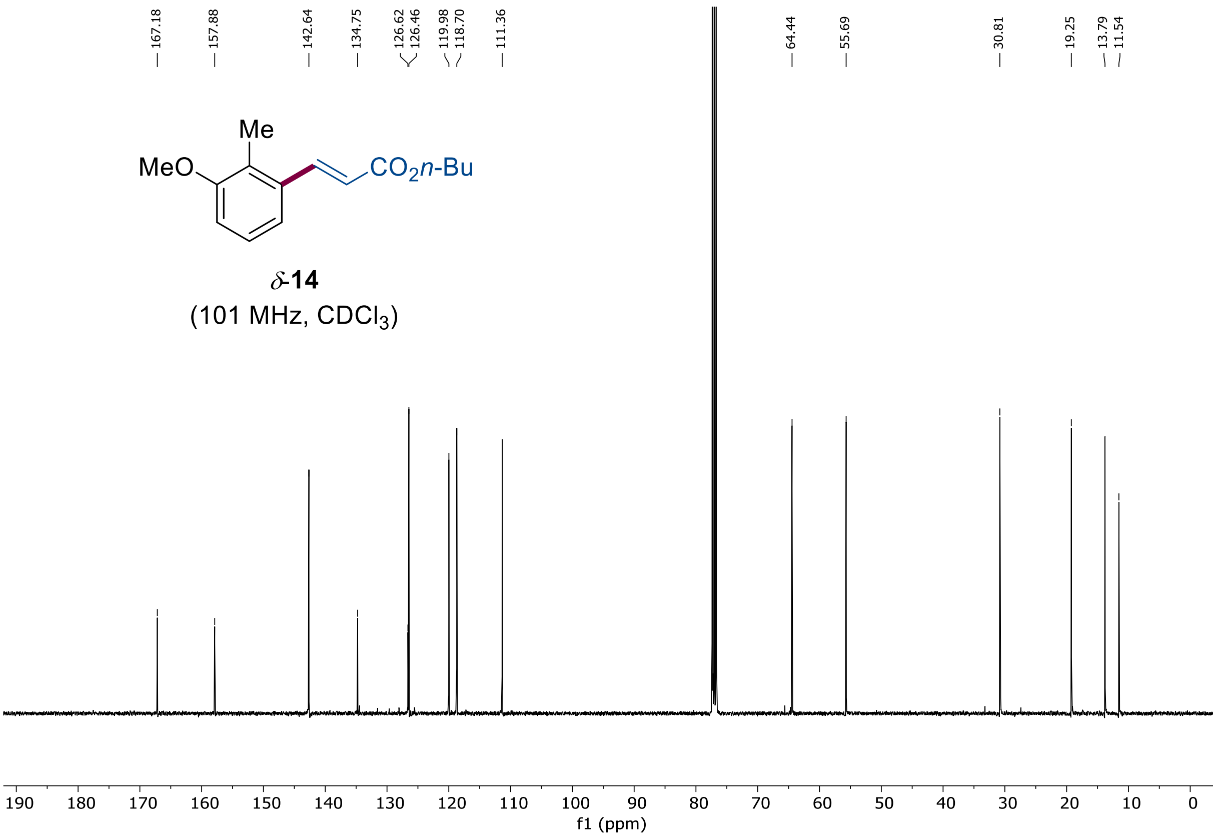

Supplementary Figure 40 **C-NMR of compound δ-14. 101 MHz, $CDCl_3$, RT**

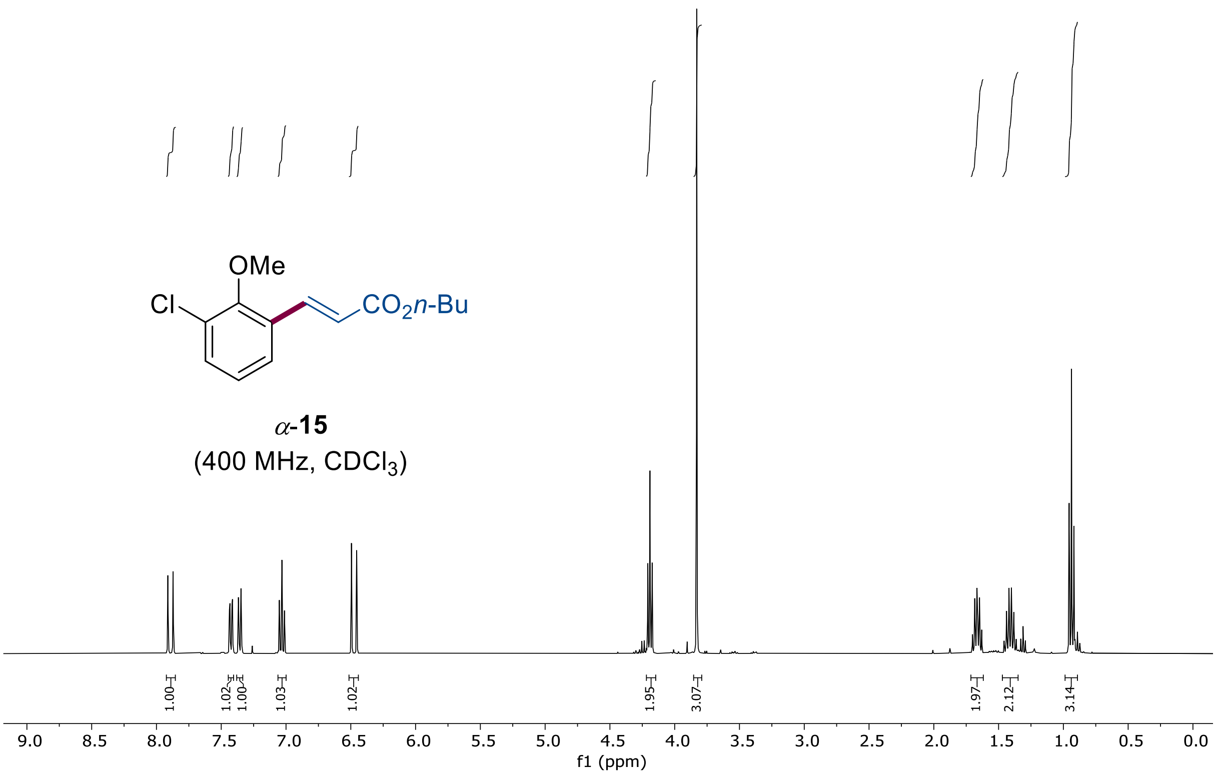

Supplementary Figure 41 **H-NMR of compound α-15. 400 MHz, $CDCl_3$, RT**

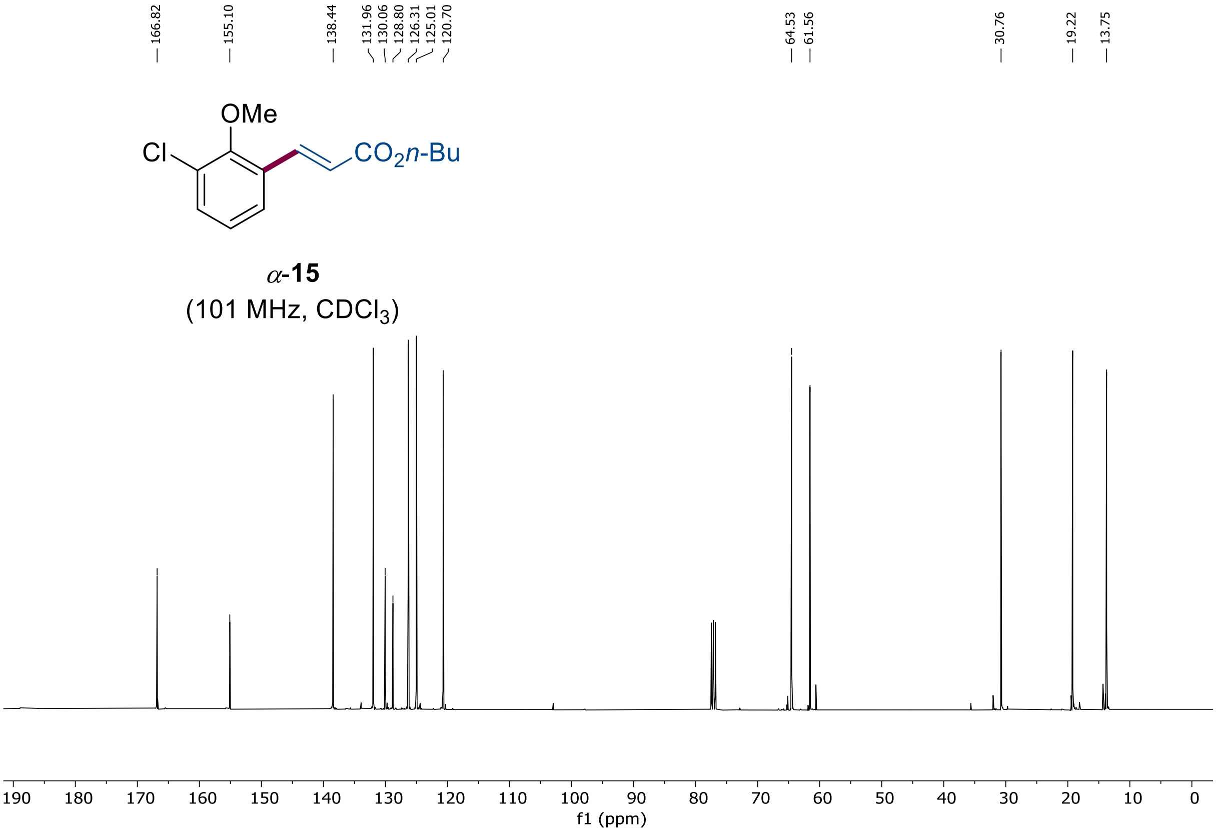

Supplementary Figure 42 C-NMR of compound α-15. 101 MHz, $CDCl_3$, RT

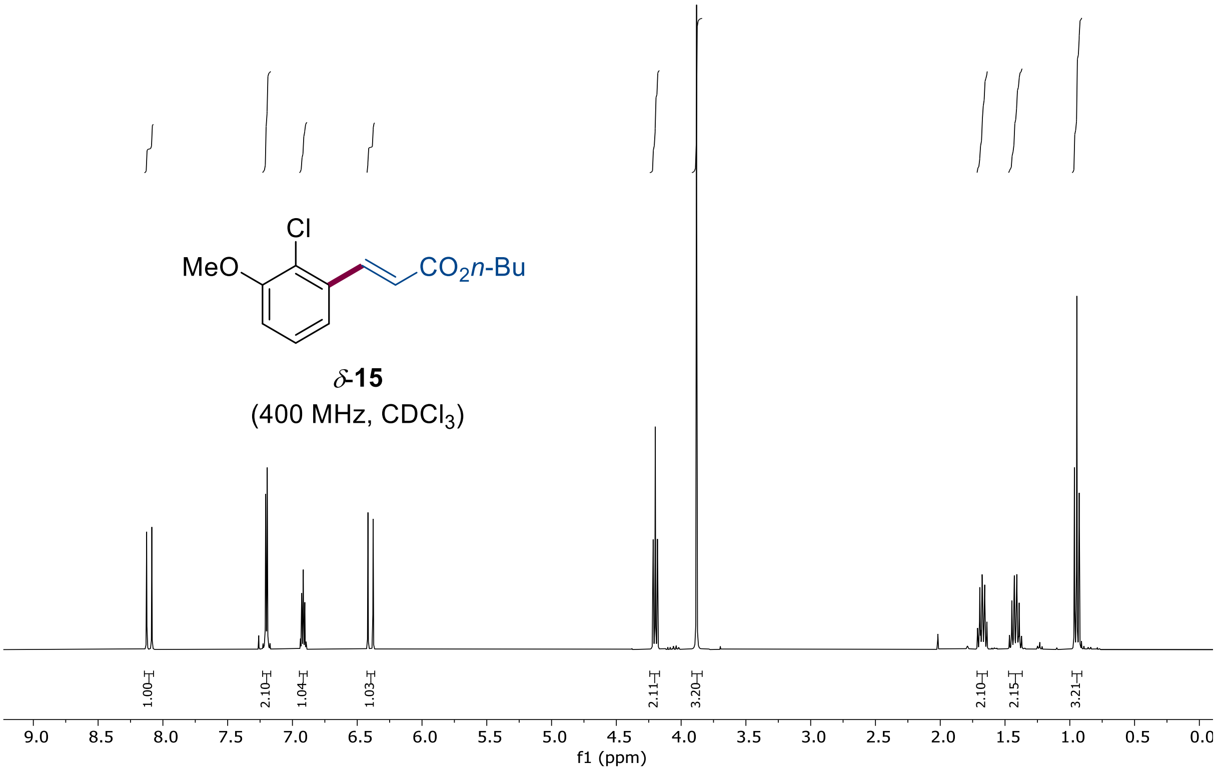

Supplementary Figure 43 H-NMR of compound δ-15. 400 MHz, $CDCl_3$, RT

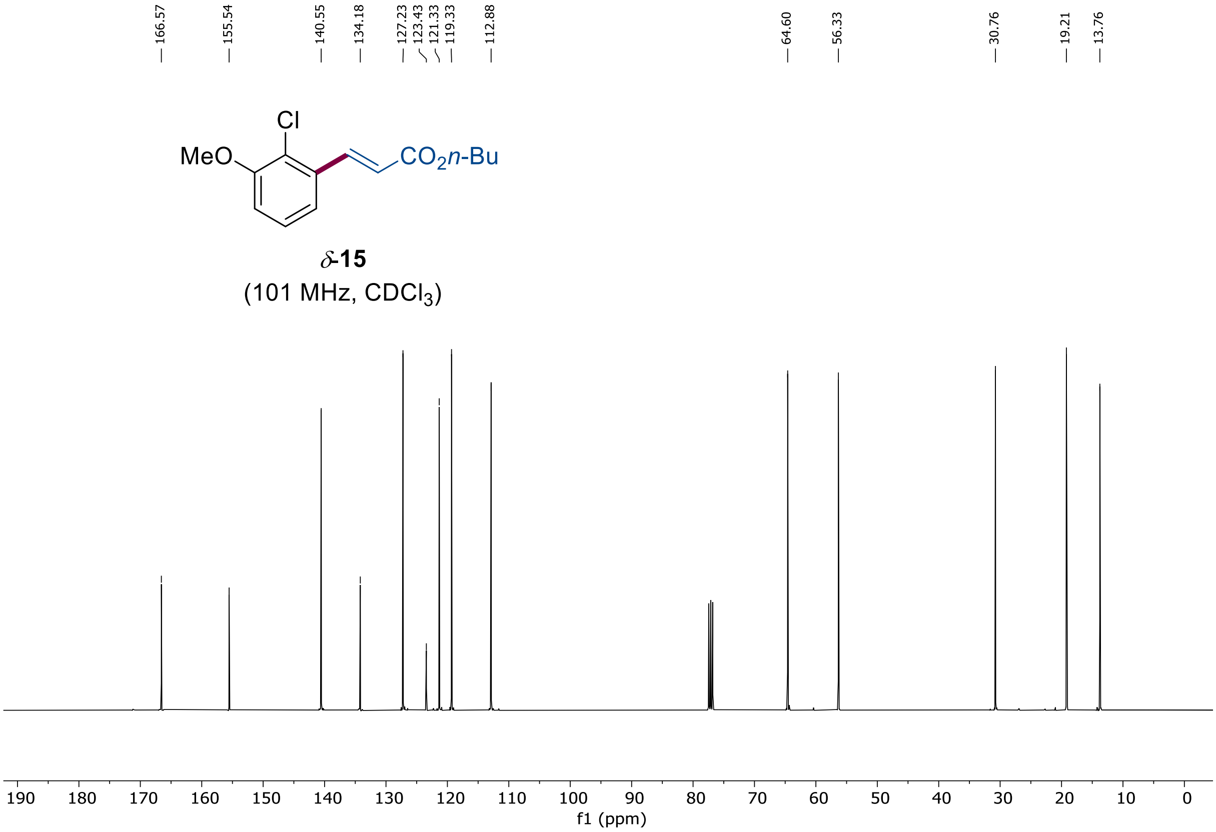

Supplementary Figure 44 C-NMR of compound δ-15. 101 MHz, $CDCl_3$, RT

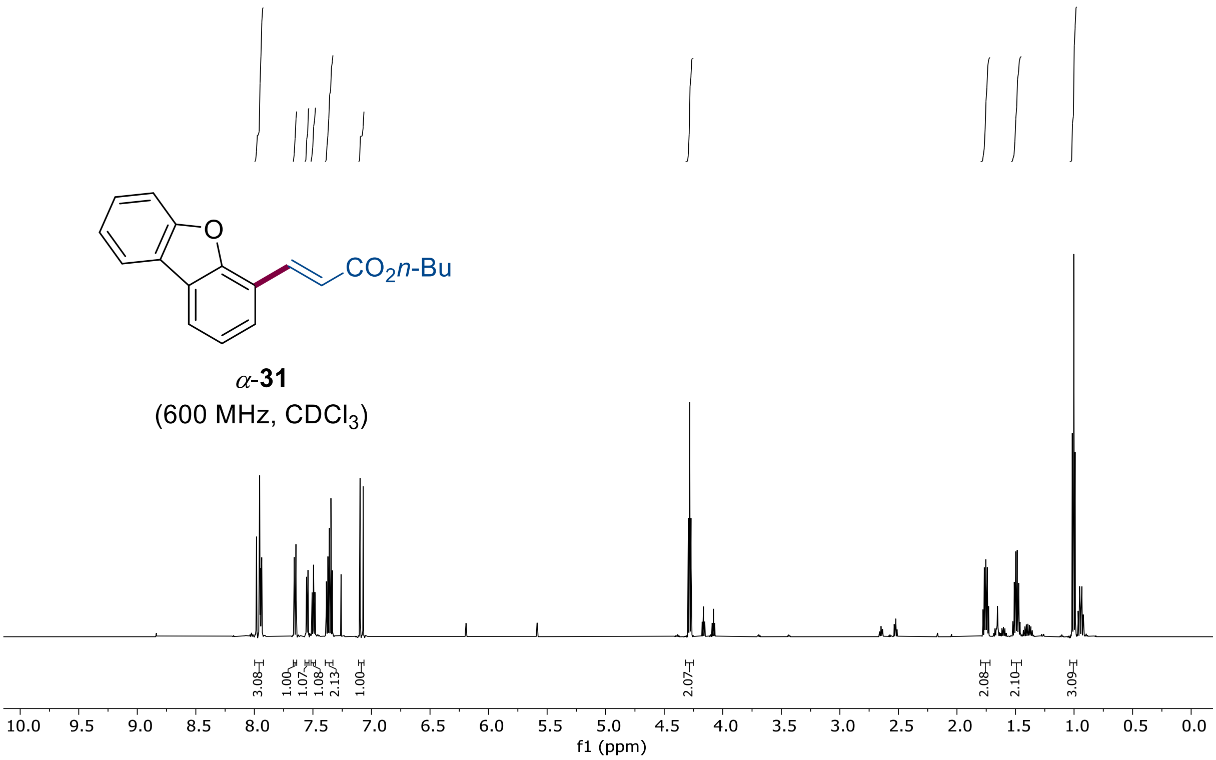

Supplementary Figure 45 H-NMR of compound α-31. 600 MHz, $CDCl_3$, RT

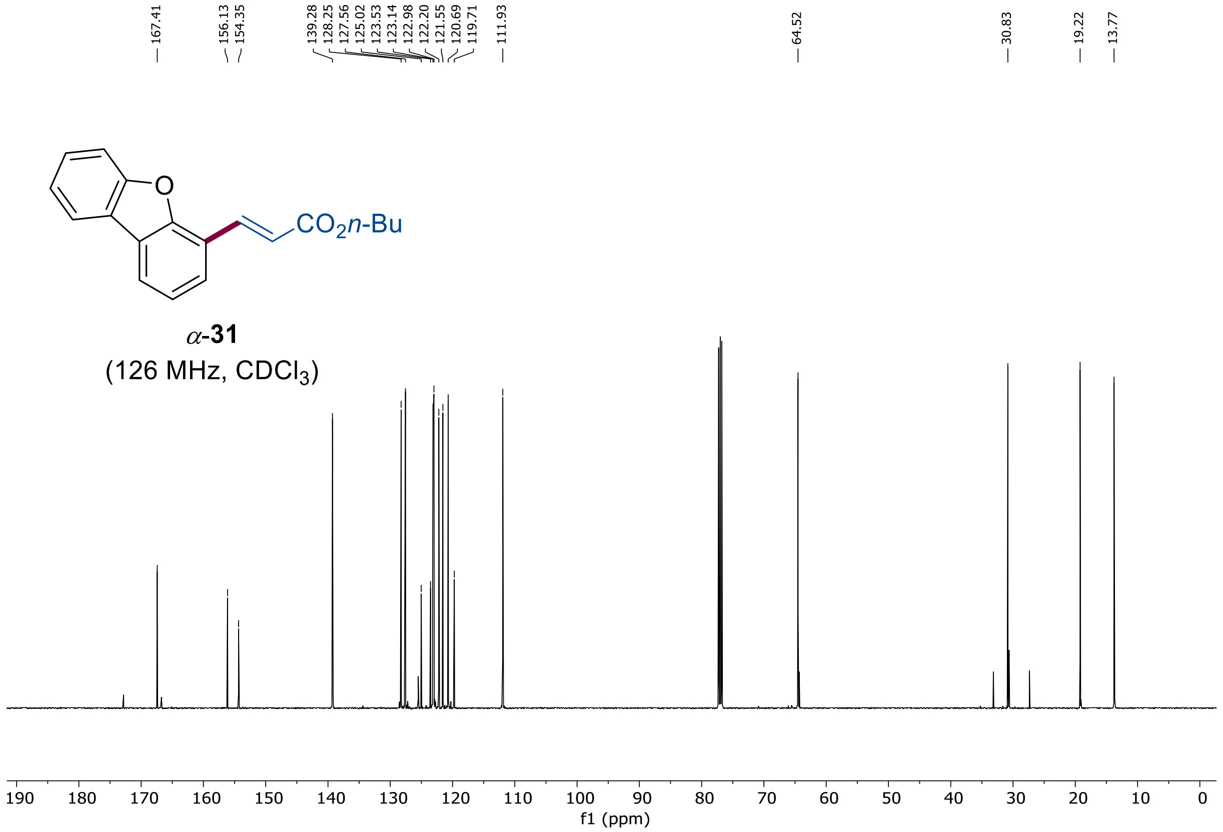

Supplementary Figure 46 C-NMR of compound α-31. 126 MHz, $CDCl_3$, RT

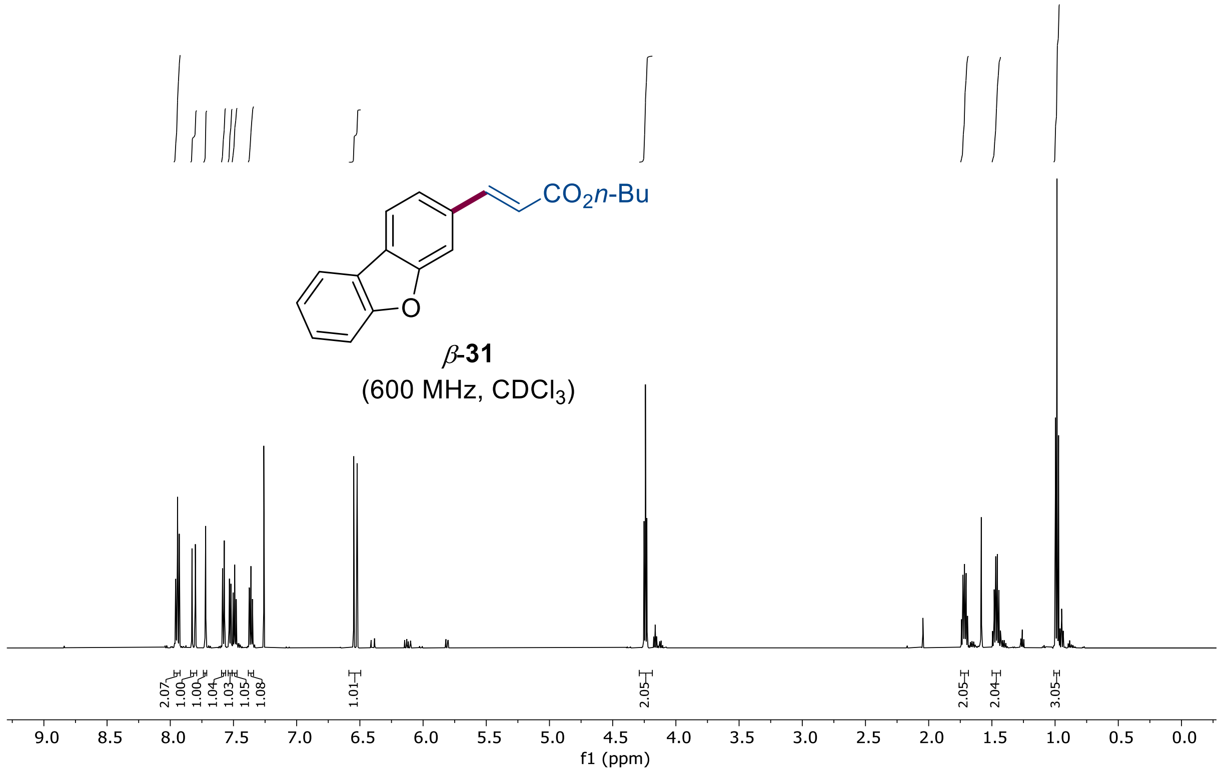

Supplementary Figure 47 H-NMR of compound β-31. 600 MHz, $CDCl_3$, RT

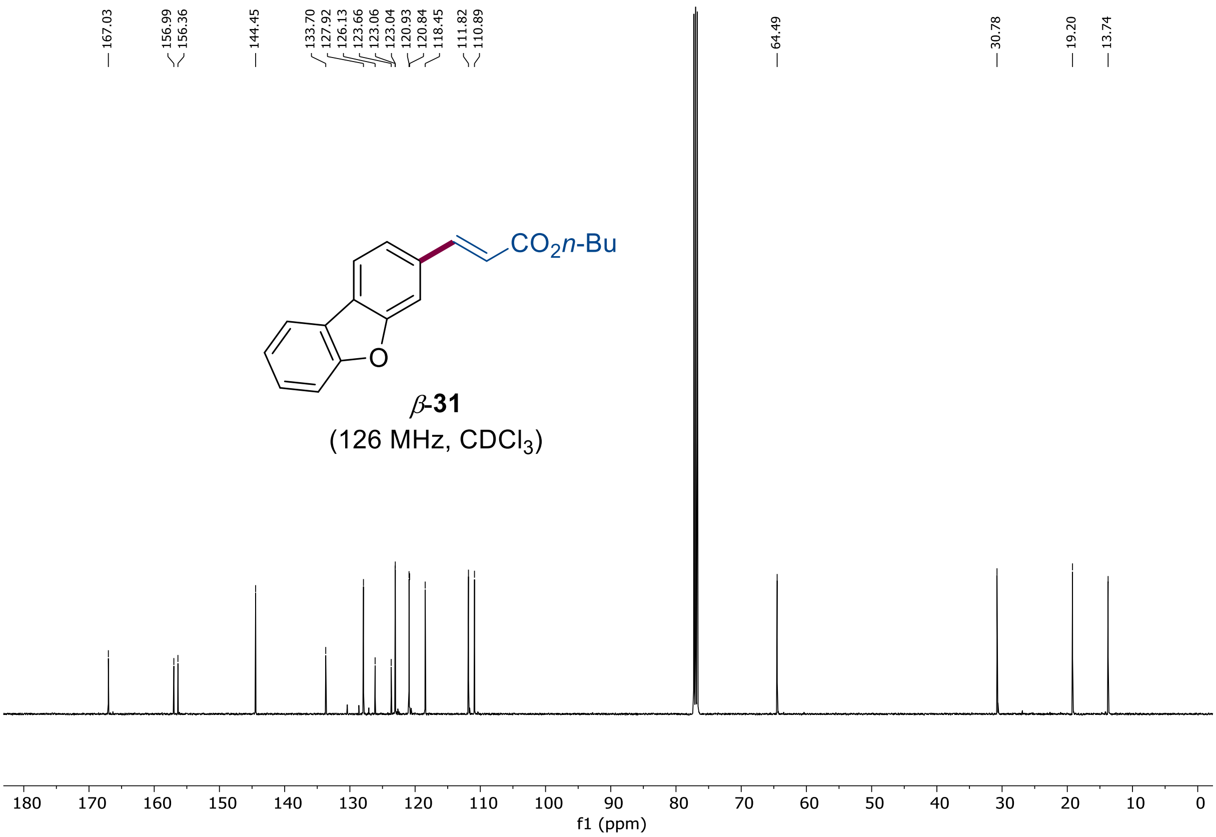

Supplementary Figure 48 **C-NMR of compound *β*-31. 126 MHz, $CDCl_3$, RT**

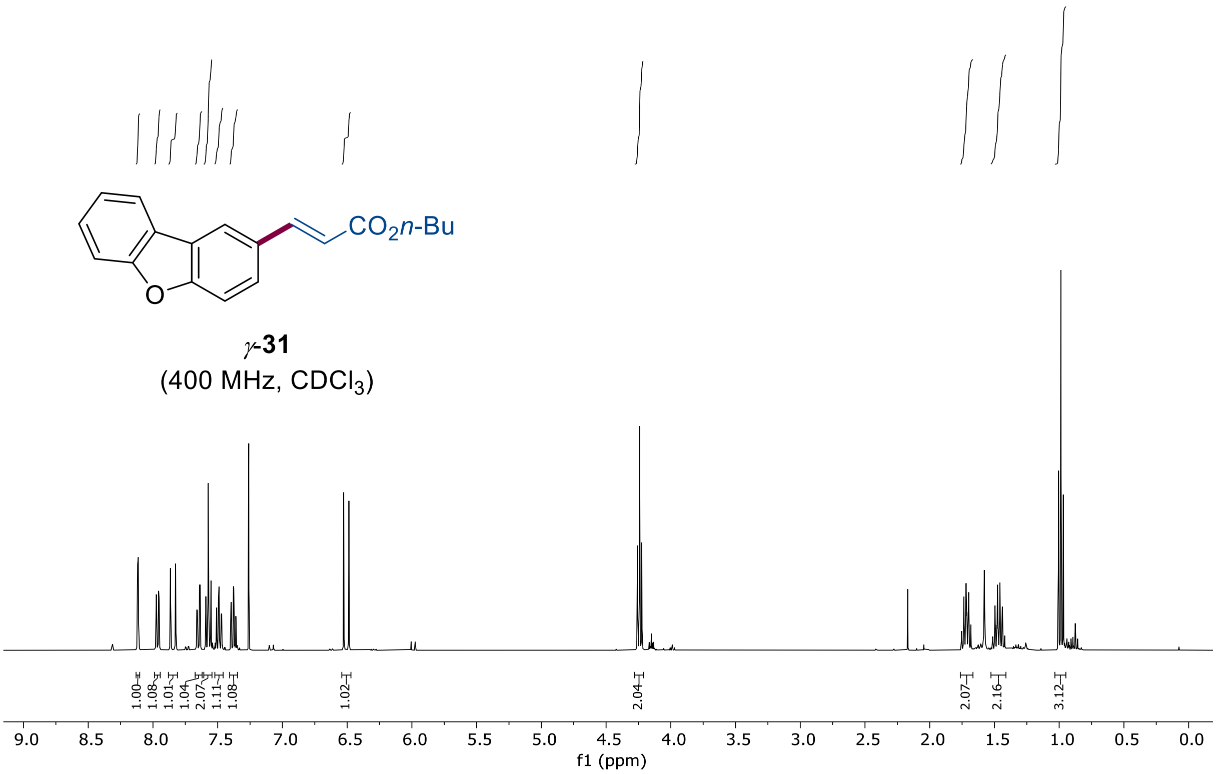

Supplementary Figure 49 **H-NMR of compound *γ*-31. 400 MHz, $CDCl_3$, RT**

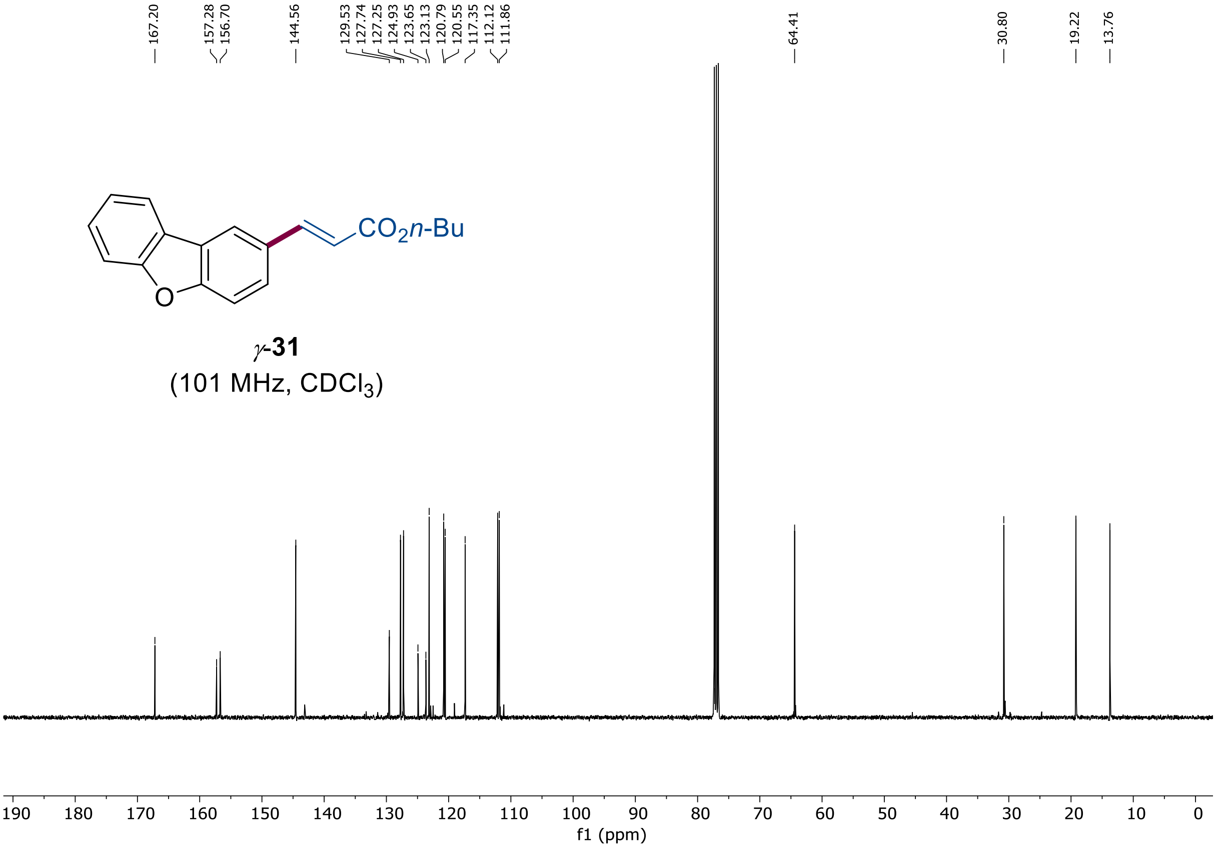

Supplementary Figure 50 **C-NMR of compound *γ*-31. 101 MHz, CDCl₃, RT**

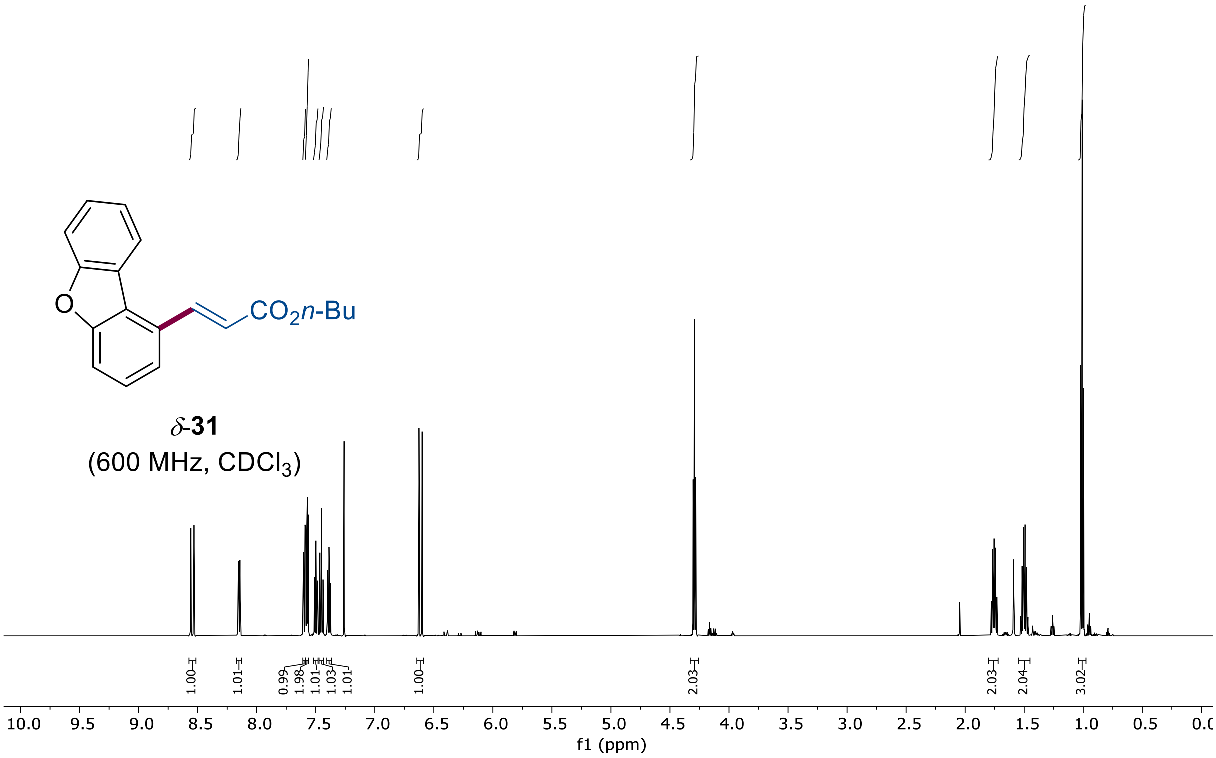

Supplementary Figure 51 **H-NMR of compound *δ*-31. 600 MHz, CDCl₃, RT**

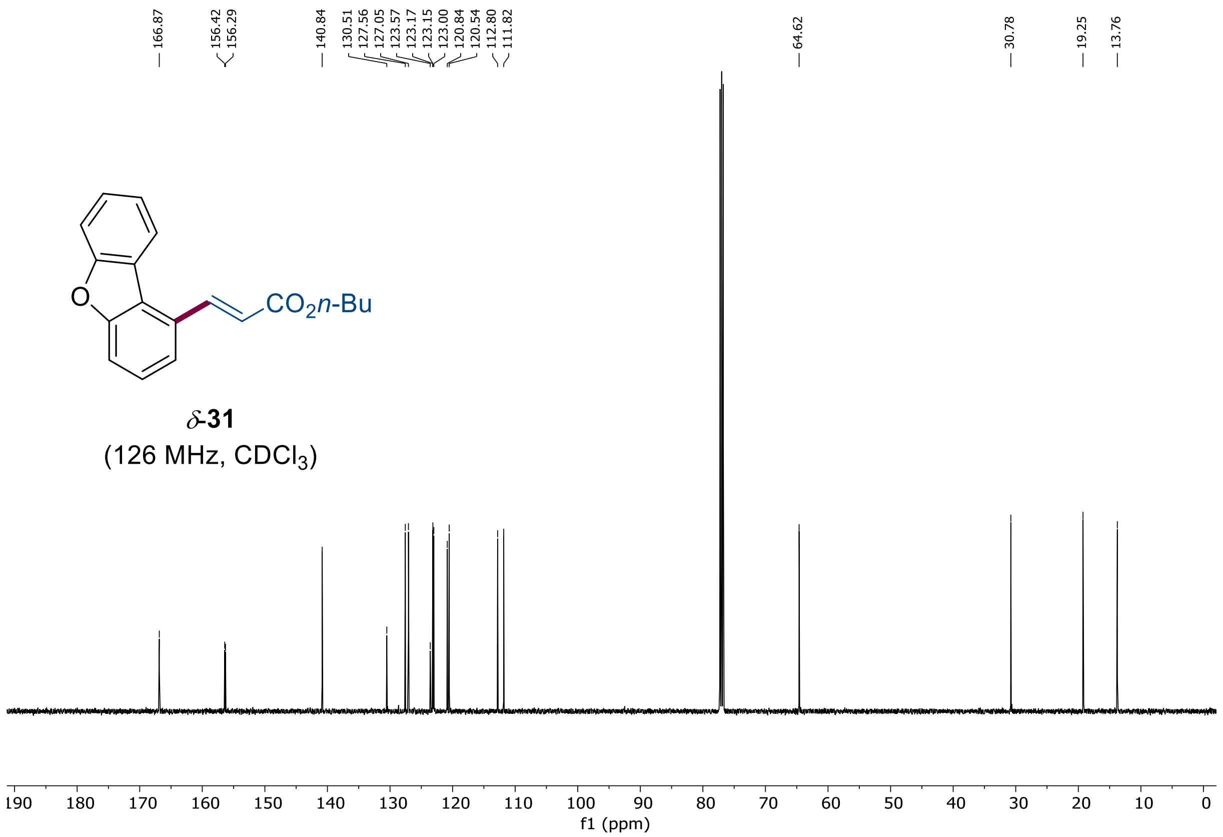

Supplementary Figure 52 C-NMR of compound δ-31. 126 MHz, $CDCl_3$, RT

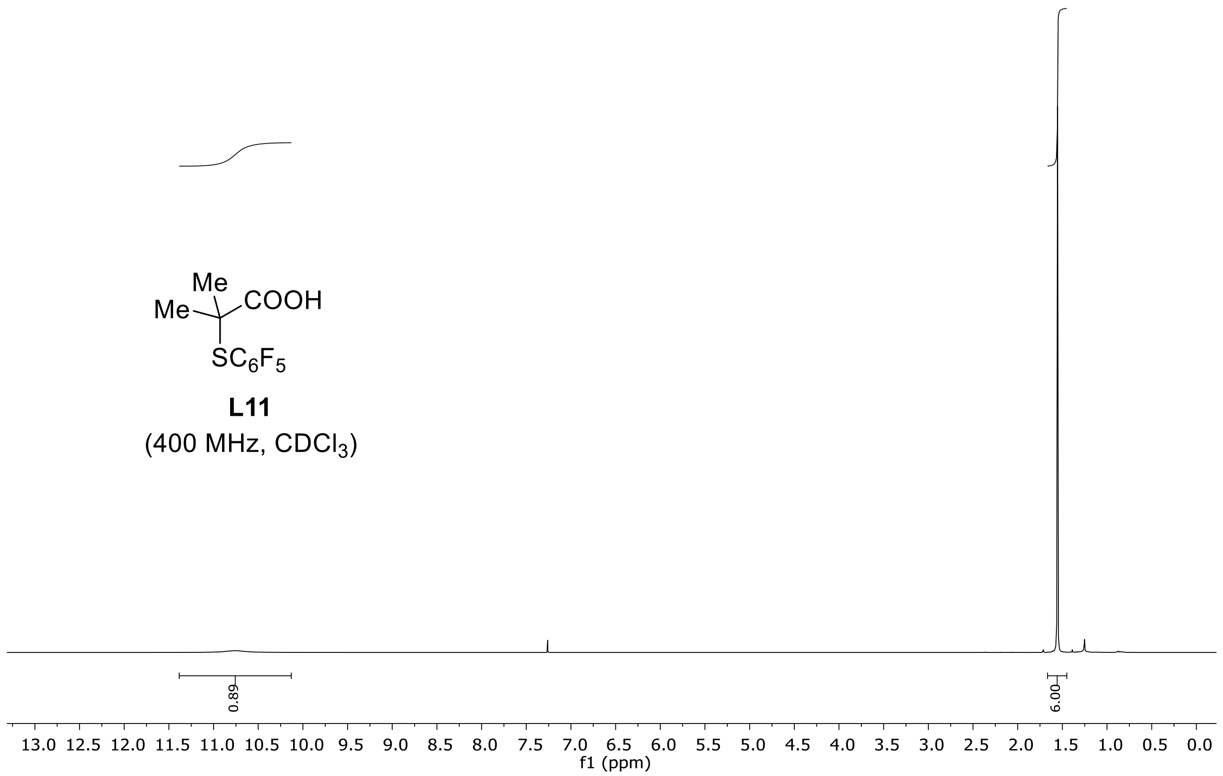

Supplementary Figure 53 H-NMR of compound L11. 400 MHz, $CDCl_3$, RT

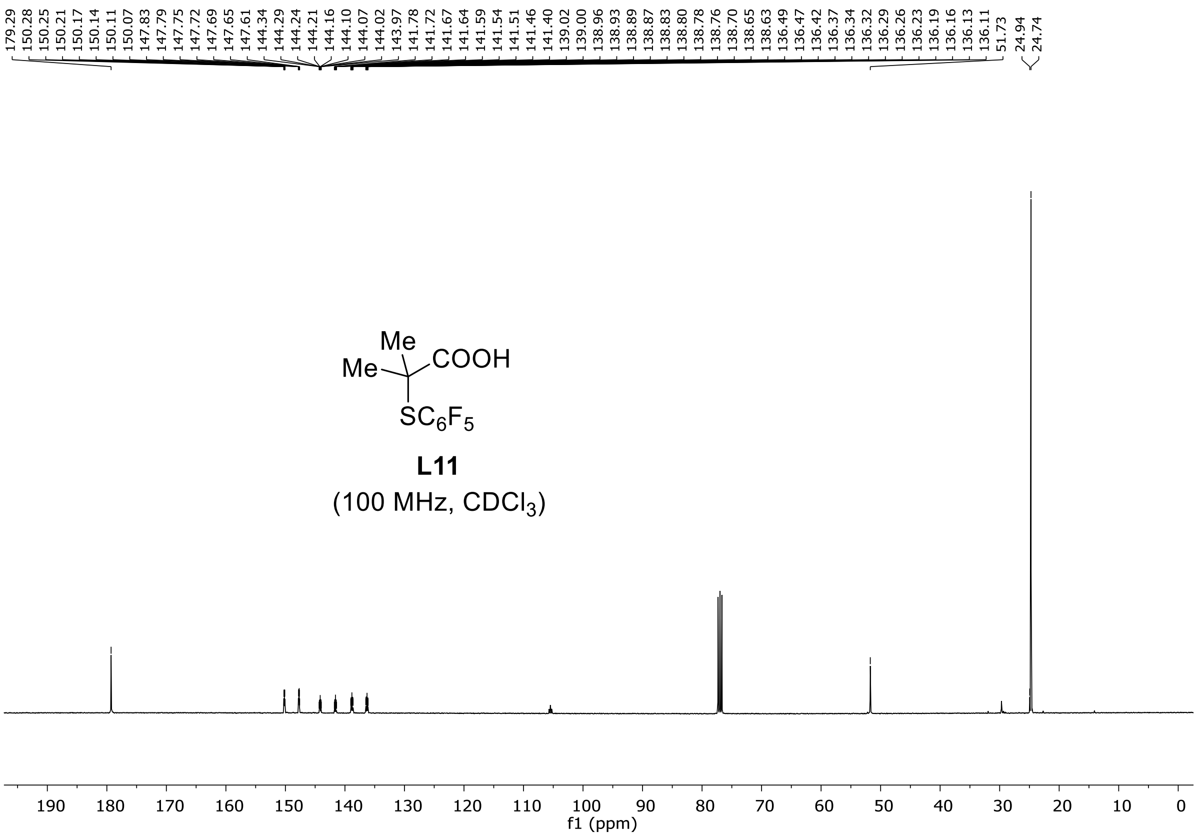

Supplementary Figure 54 **C-NMR of compound L11. 101 MHz, $CDCl_3$, RT**

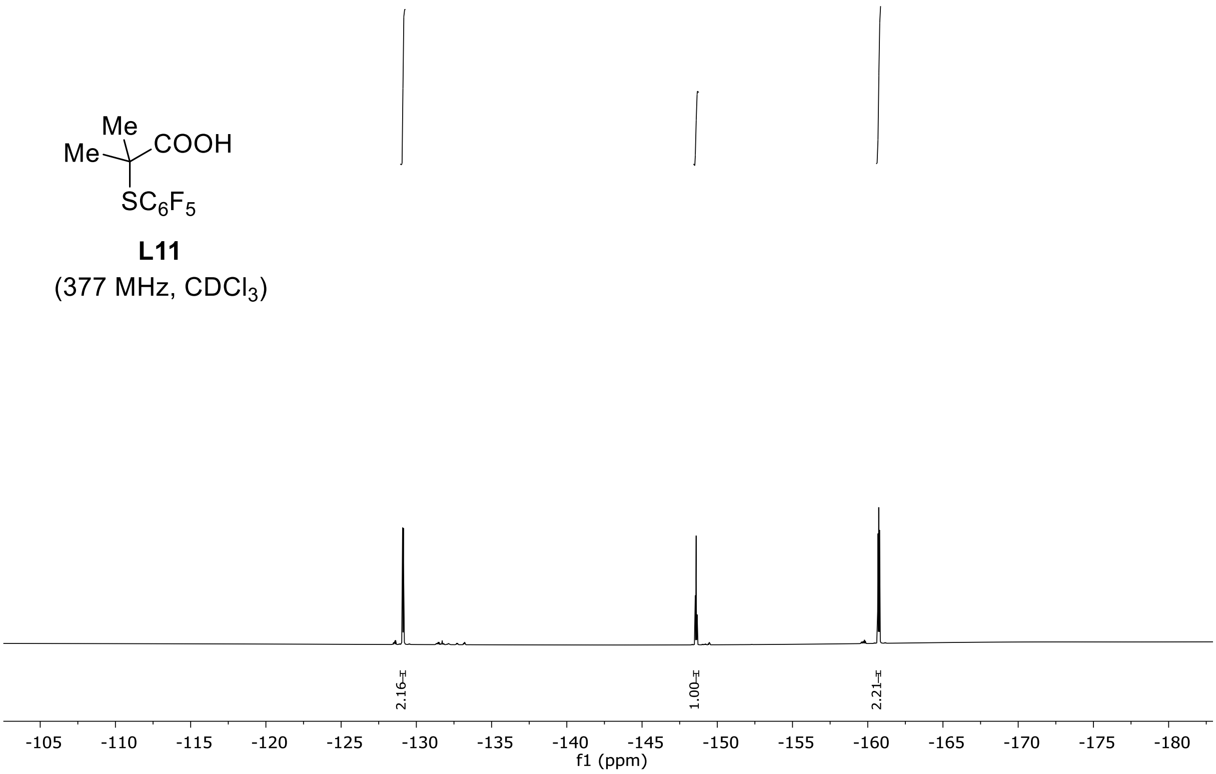

Supplementary Figure 55 **F-NMR of compound L11. 377 MHz, $CDCl_3$, RT**

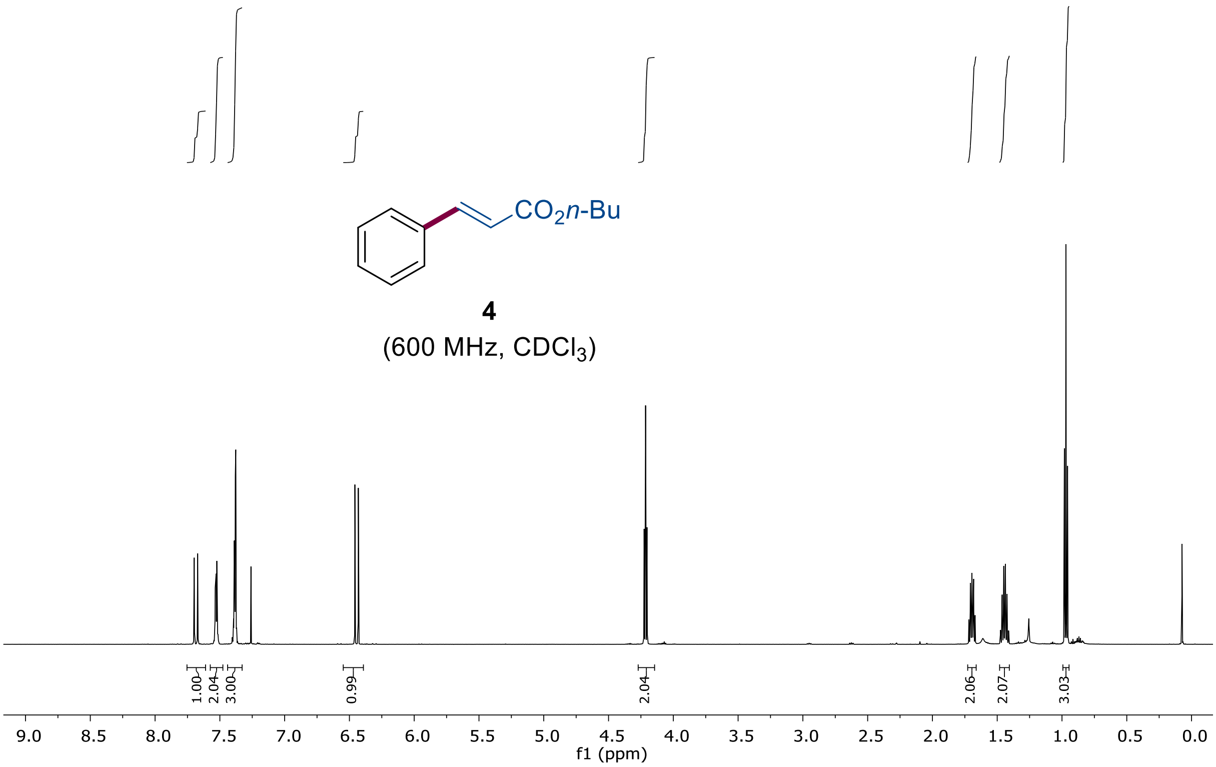

Supplementary Figure 56 H-NMR of compound 4. 600 MHz, $CDCl_3$, RT

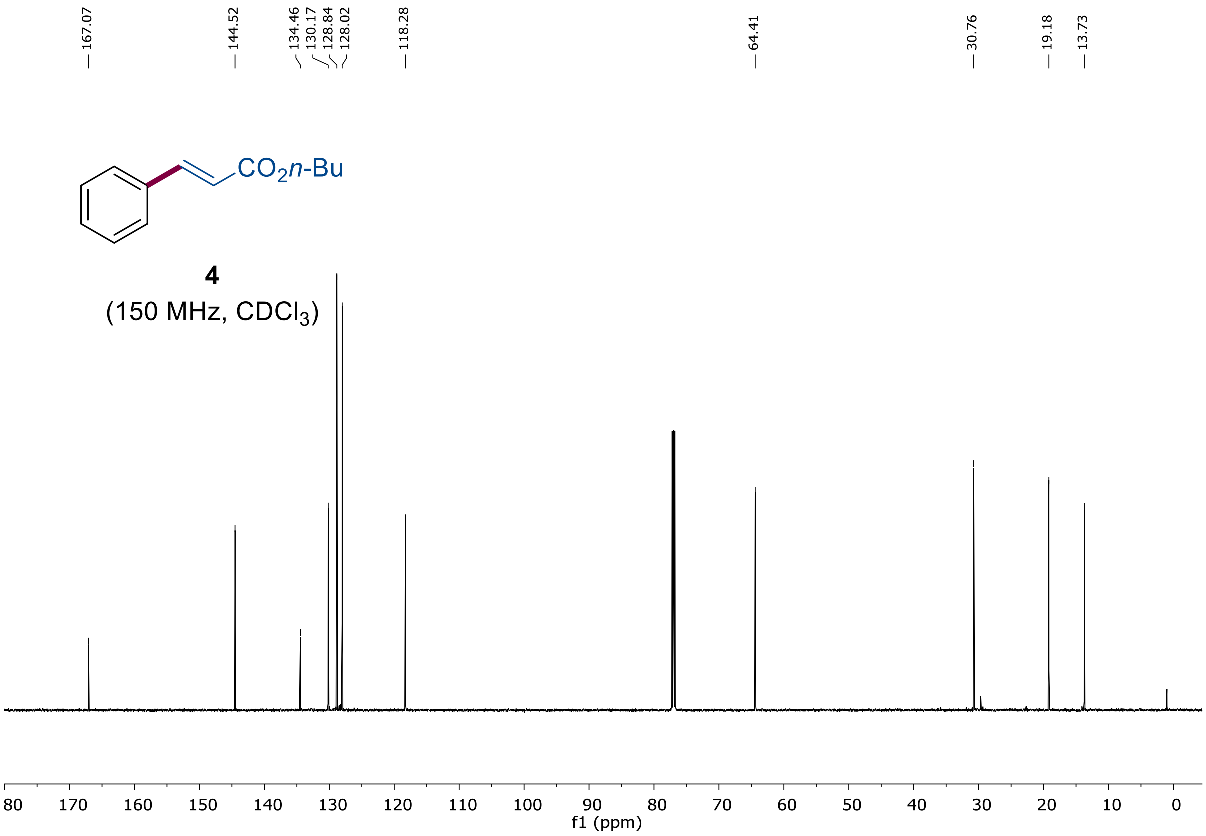

Supplementary Figure 57 C-NMR of compound 4. 150 MHz, $CDCl_3$, RT

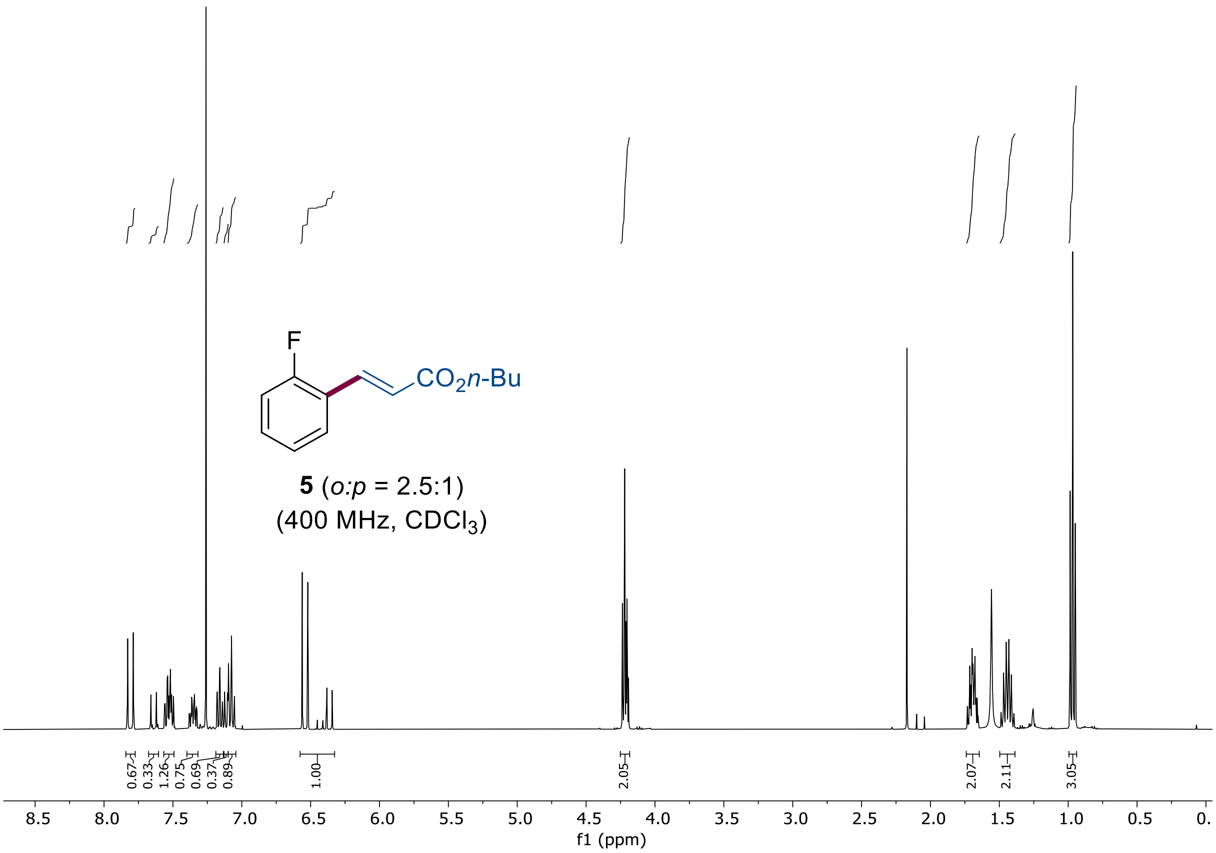

Supplementary Figure 58 H-NMR of compound 5. 400 MHz, $CDCl_3$, RT

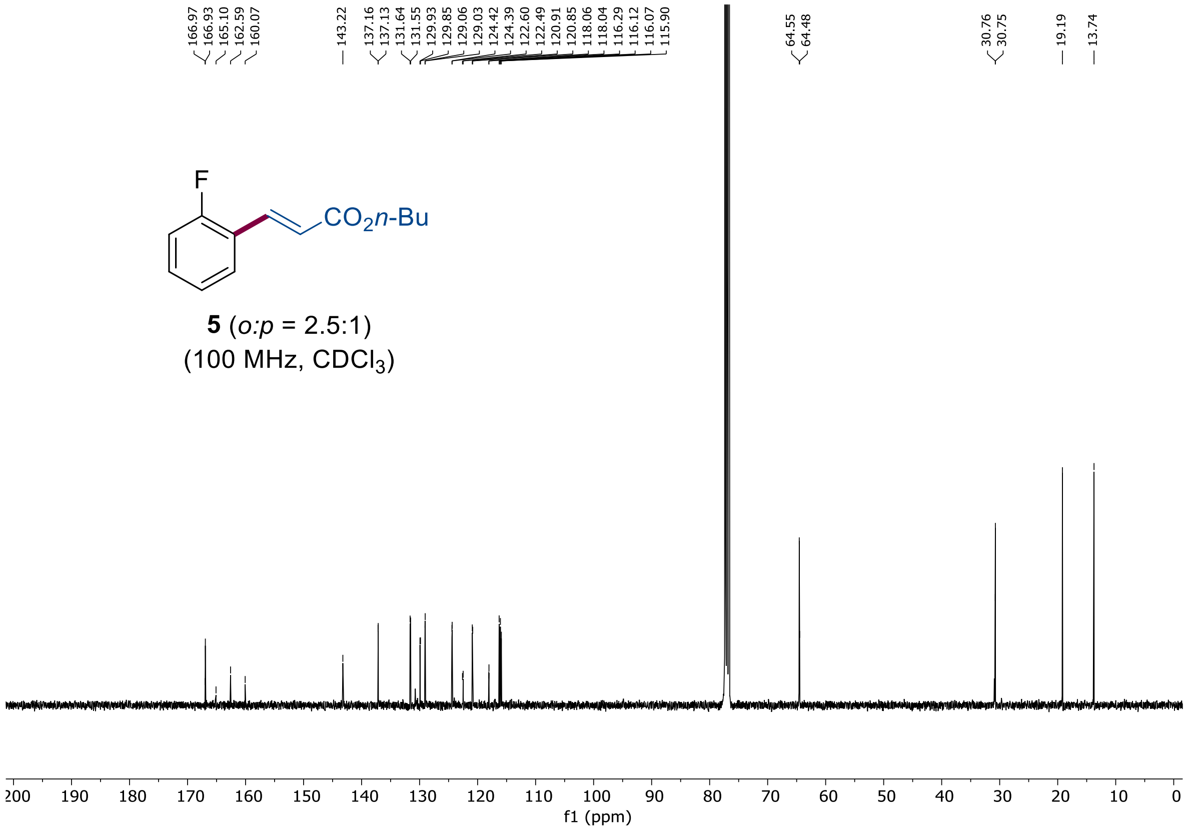

Supplementary Figure 59 C-NMR of compound 5. 100 MHz, $CDCl_3$, RT

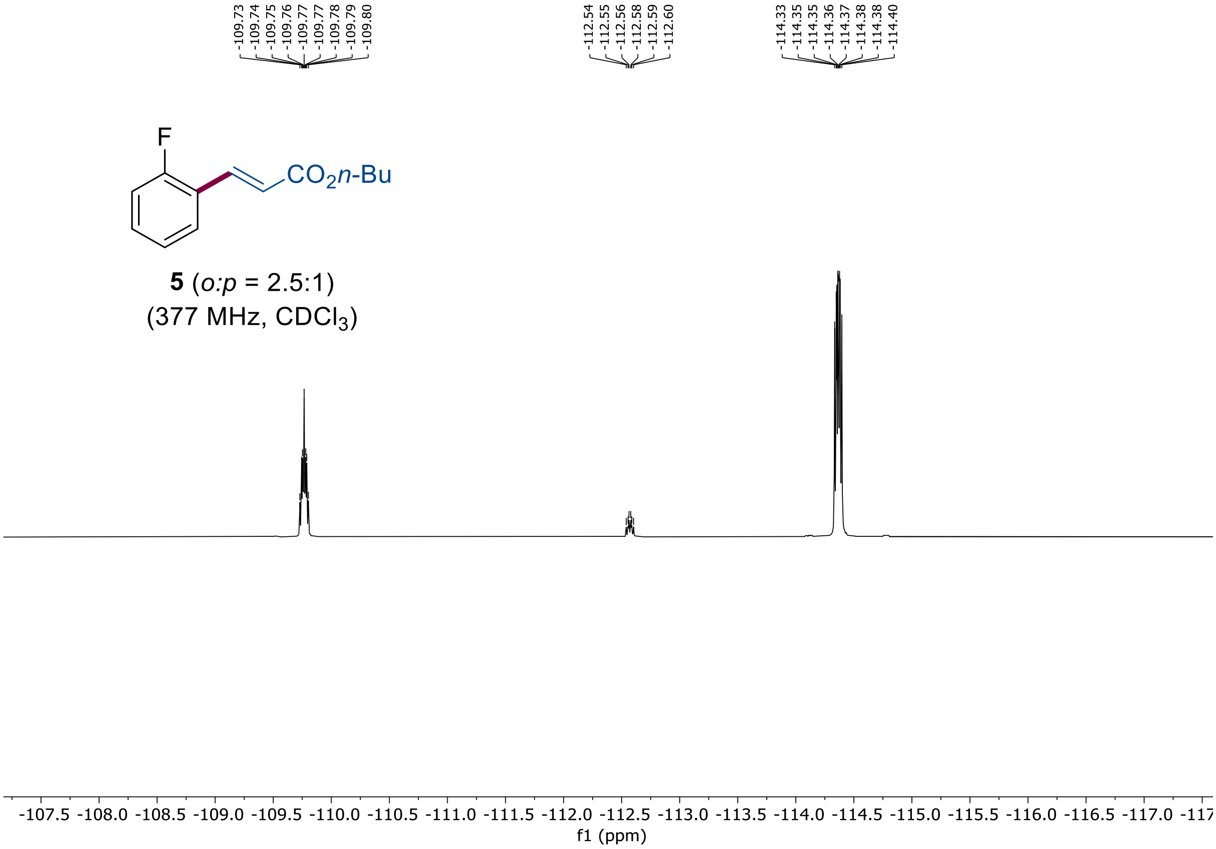

Supplementary Figure 60 F-NMR of compound 5. 377 MHz, $CDCl_3$, RT

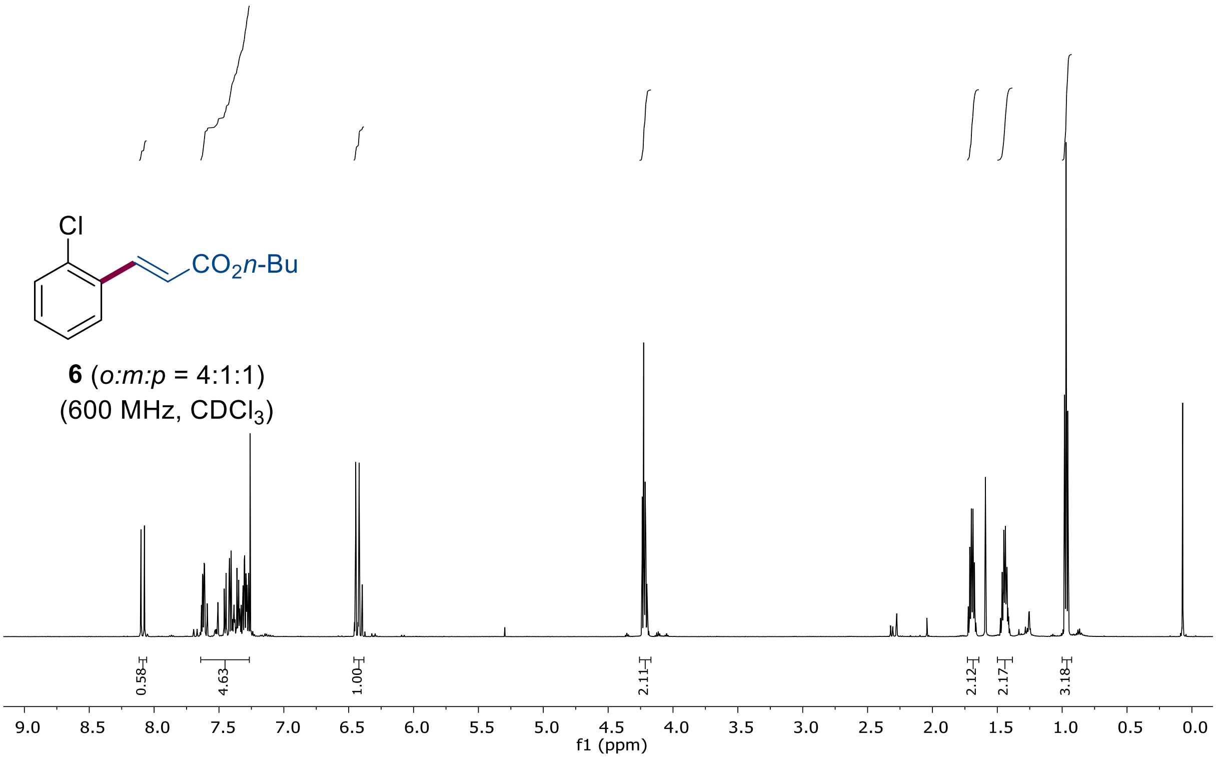

Supplementary Figure 61 H-NMR of compound 6. 600 MHz, $CDCl_3$, RT

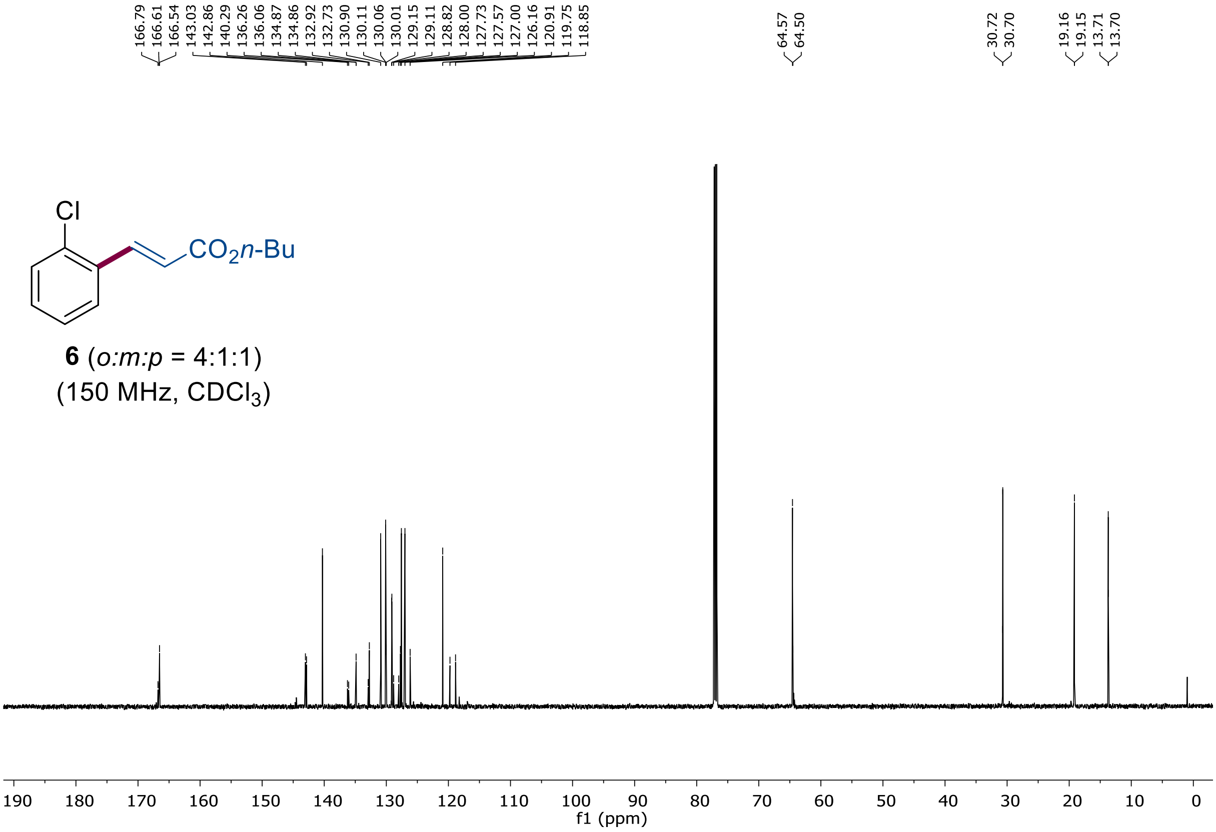

Supplementary Figure 62 C-NMR of compound 6. 150 MHz, CDCl₃, RT

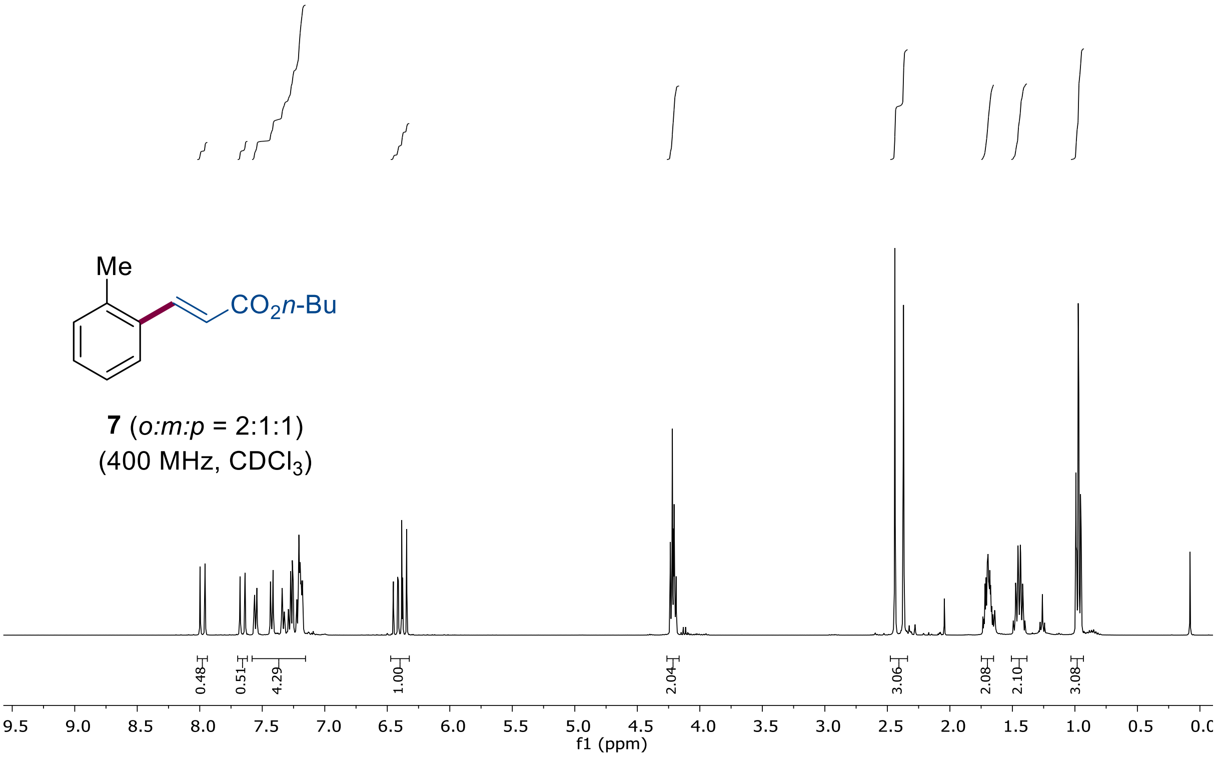

Supplementary Figure 63 H-NMR of compound 7. 400 MHz, CDCl₃, RT

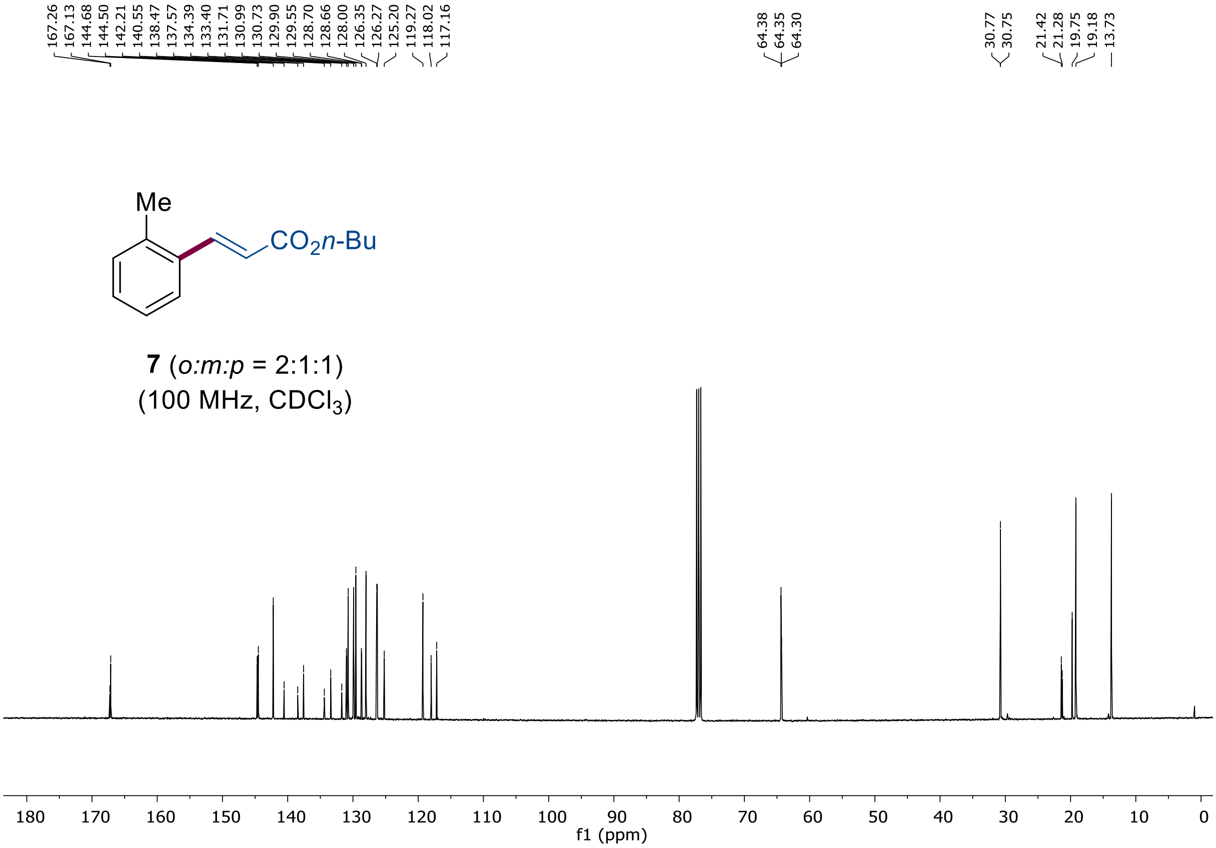

Supplementary Figure 64 C-NMR of compound 7. 100 MHz, CDCl₃, RT

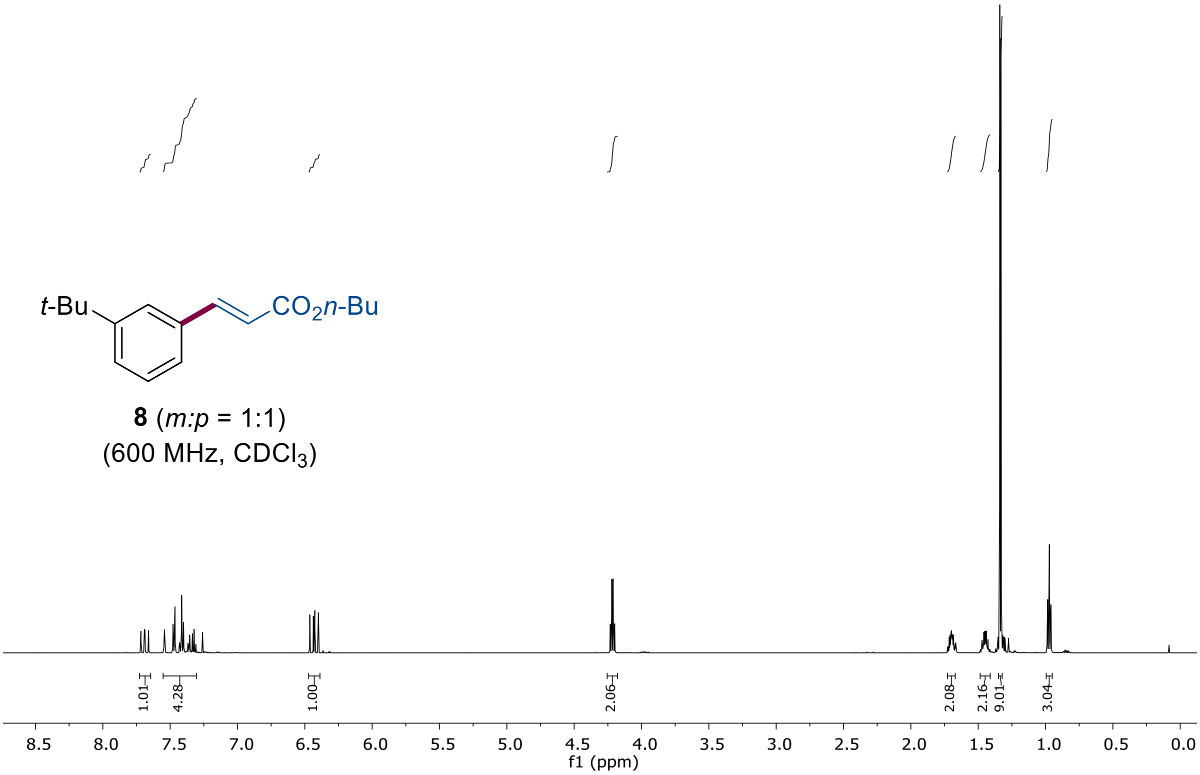

Supplementary Figure 65 H-NMR of compound 8. 600 MHz, CDCl₃, RT

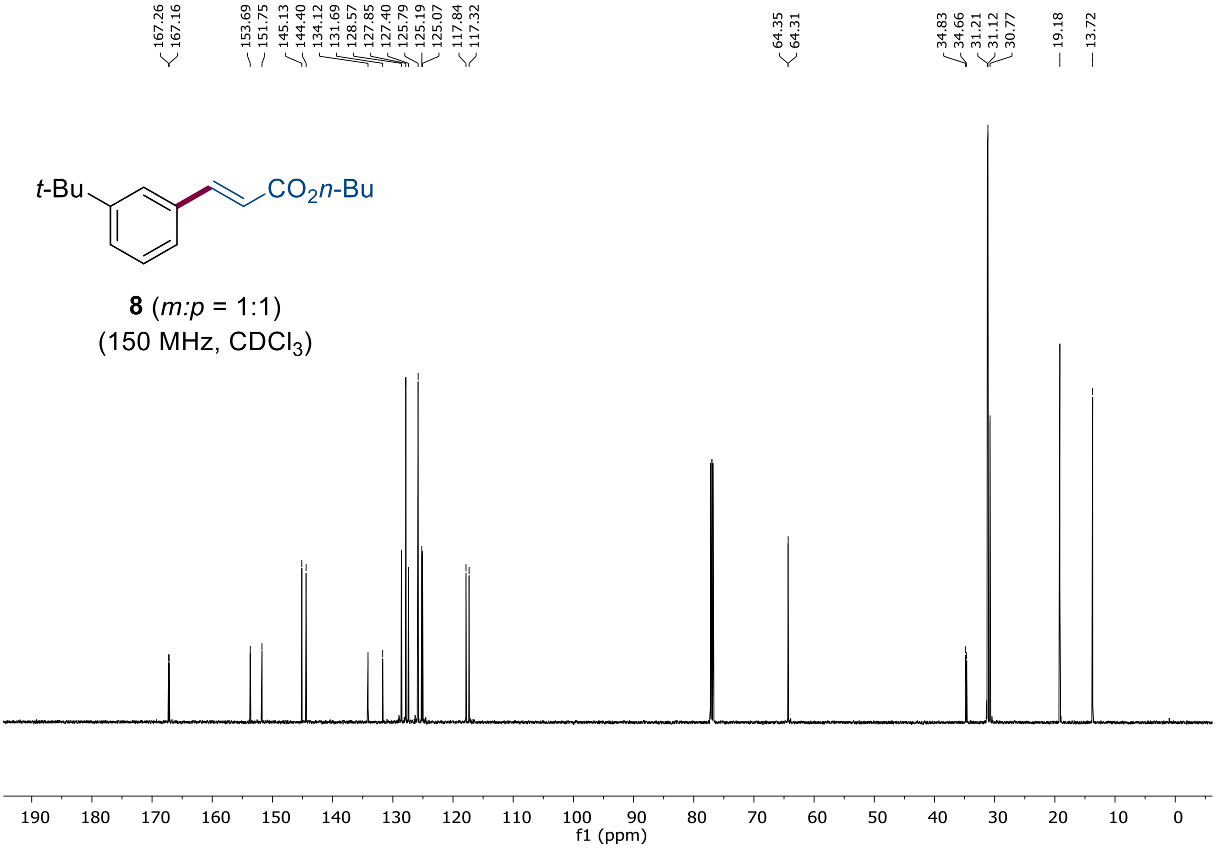

Supplementary Figure 66 **C-NMR of compound 8. 150 MHz, $CDCl_3$, RT**

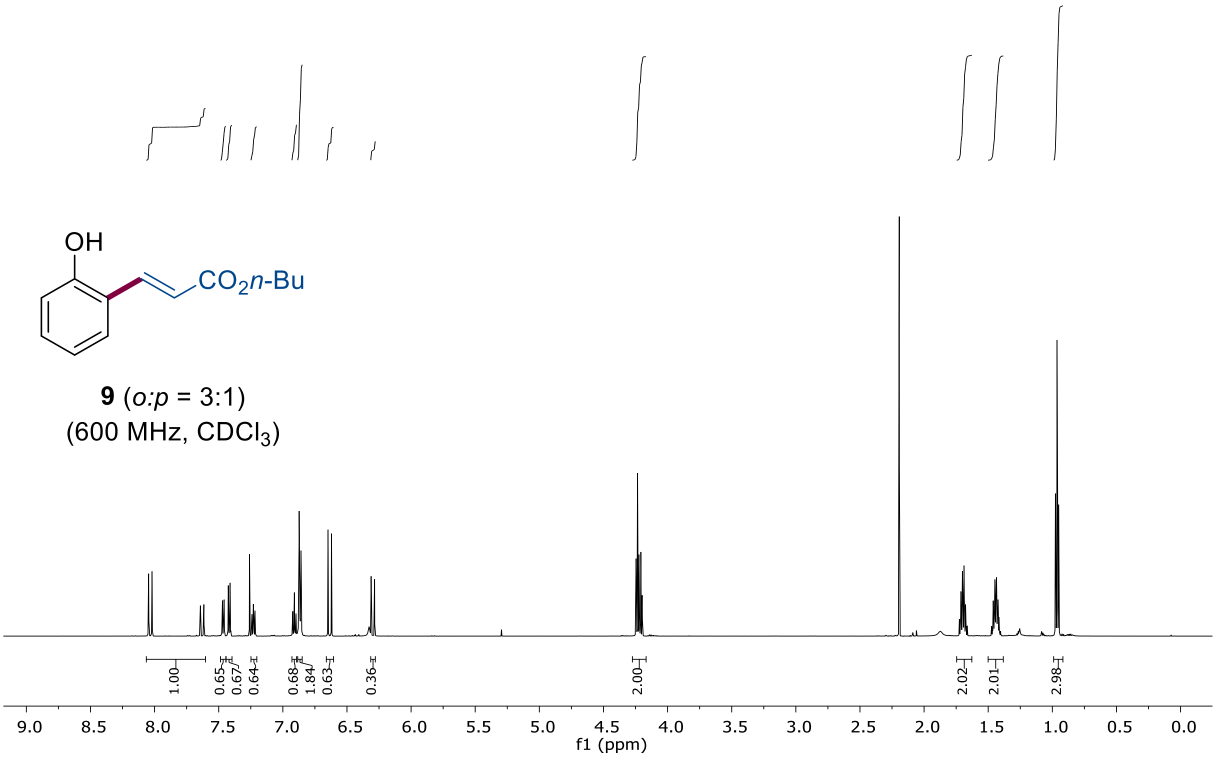

Supplementary Figure 67 **H-NMR of compound 9. 600 MHz, $CDCl_3$, RT**

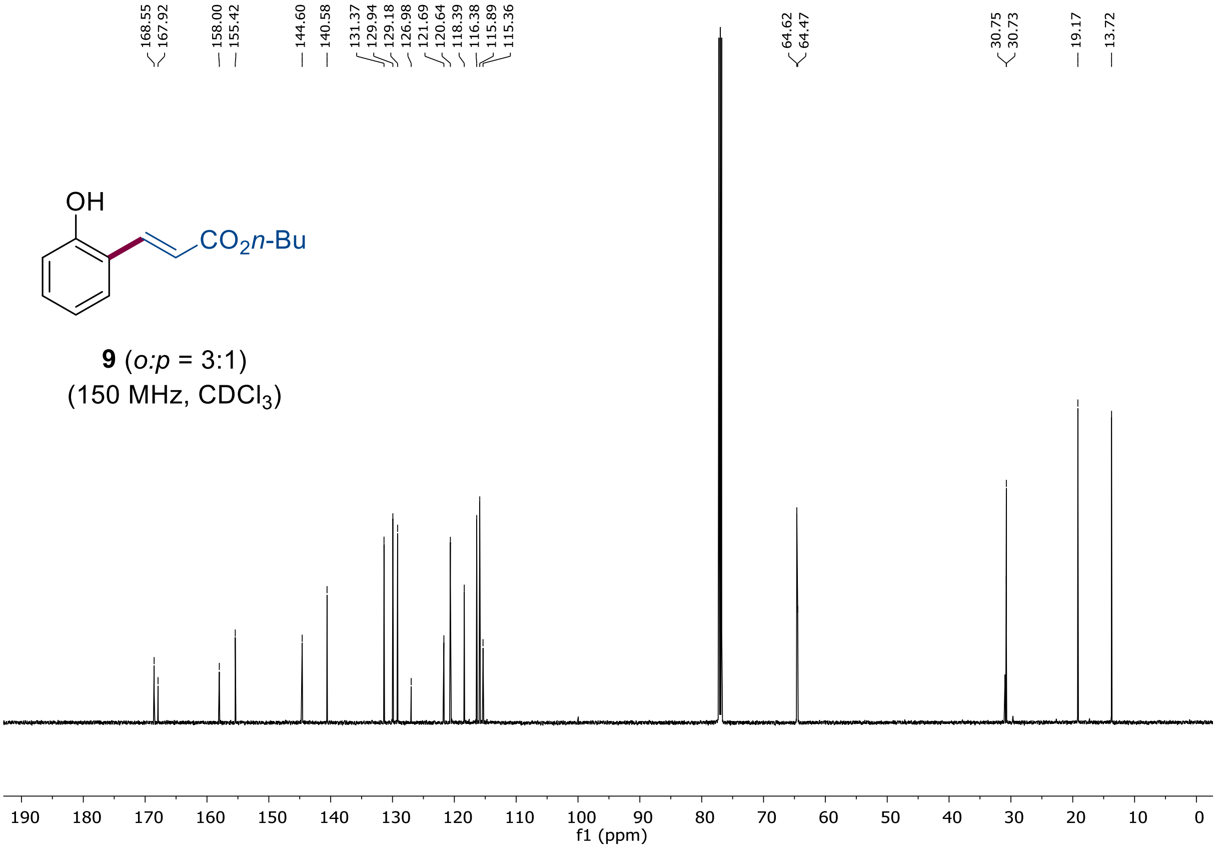

Supplementary Figure 68 **C-NMR of compound 9. 150 MHz, $CDCl_3$, RT**

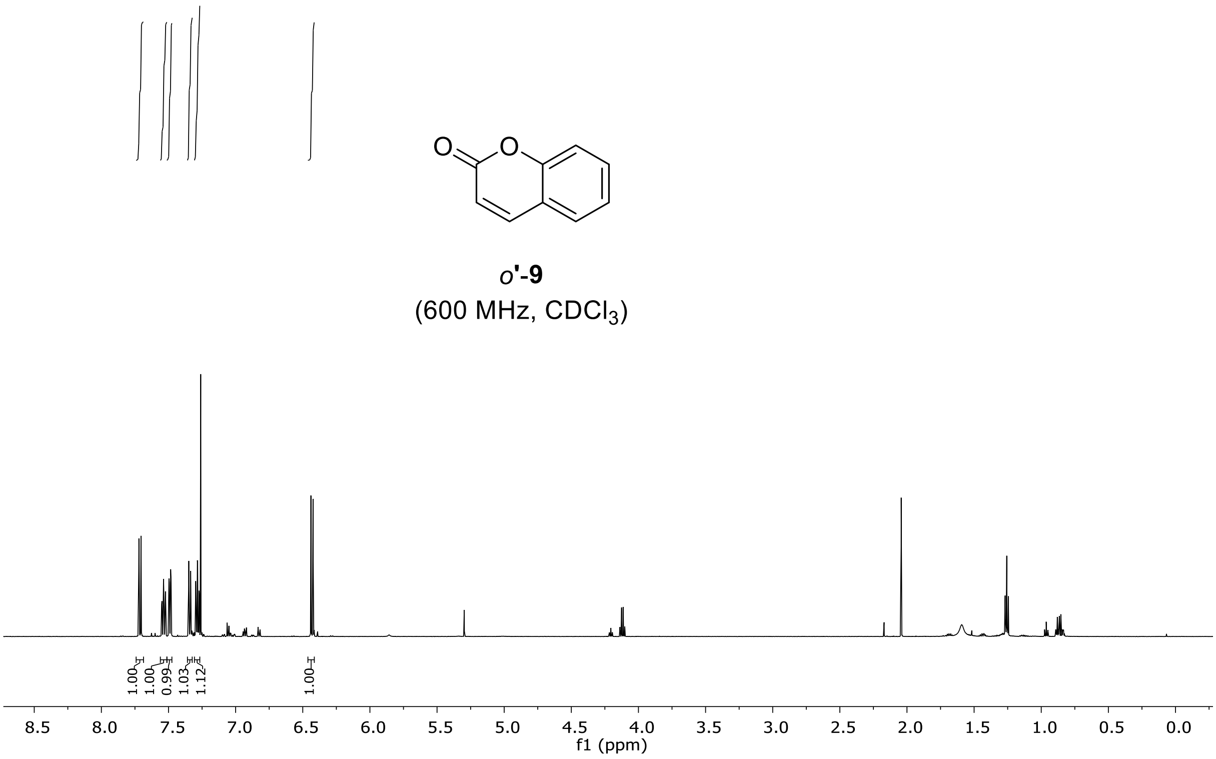

Supplementary Figure 69 **H-NMR of compound *o'*-9. 600 MHz, $CDCl_3$, RT**

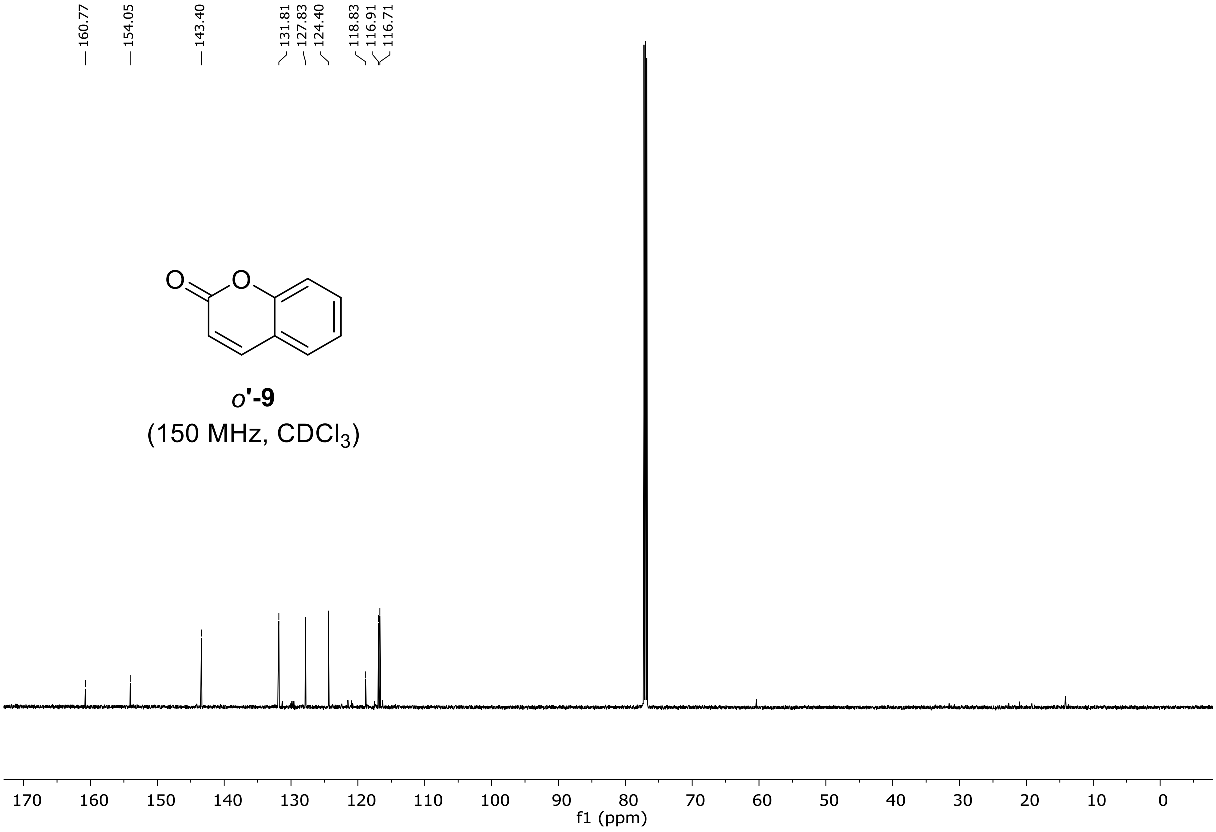

Supplementary Figure 70 H-NMR of compound *o'*-9. 150 MHz, $CDCl_3$, RT

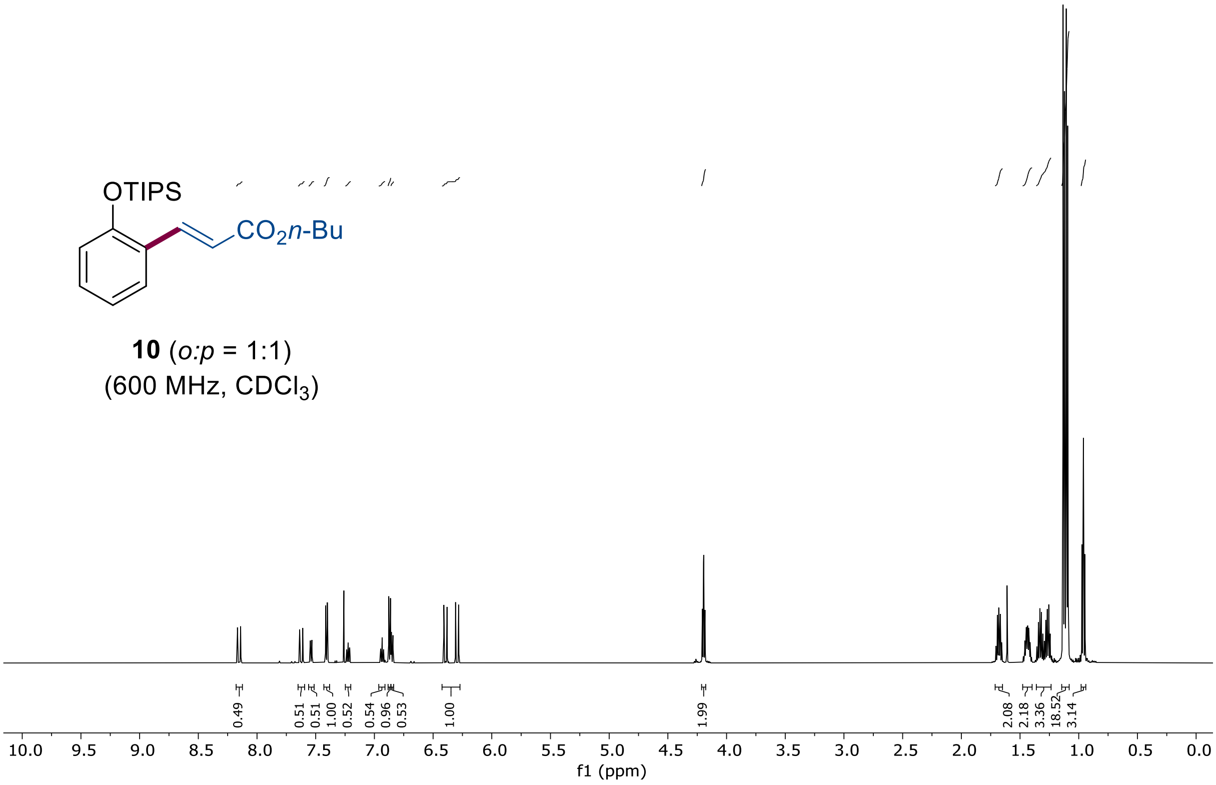

Supplementary Figure 71 H-NMR of compound 10. 600 MHz, $CDCl_3$, RT

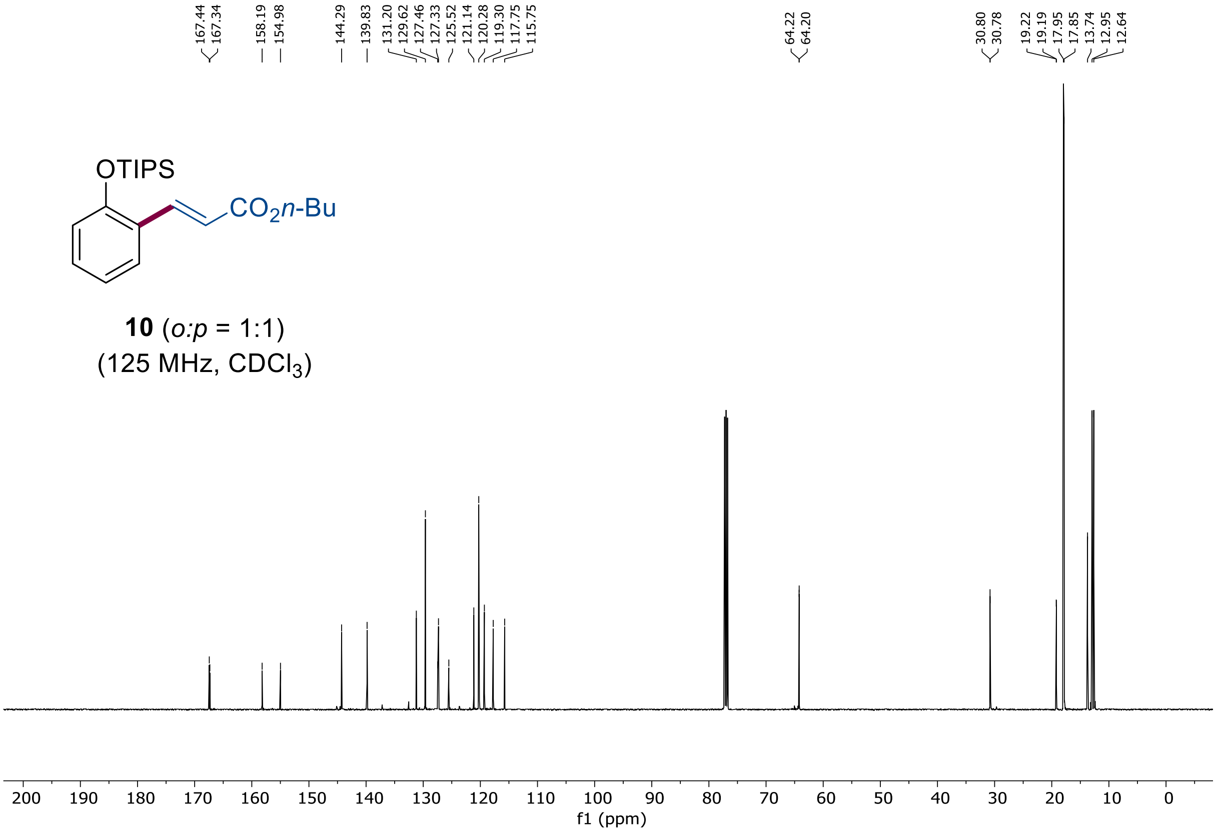

Supplementary Figure 72 C-NMR of compound 10. 125 MHz, $CDCl_3$, RT

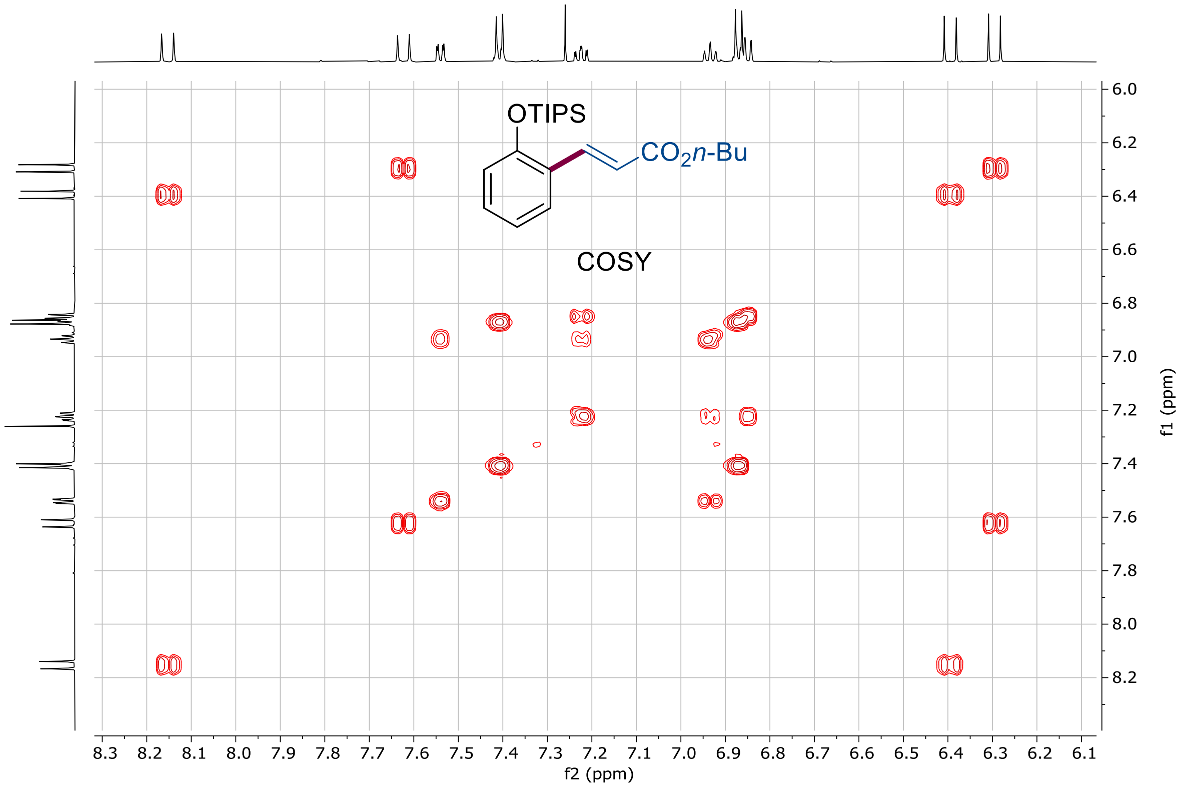

Supplementary Figure 73 COSY-NMR of compound 10. $CDCl_3$, RT

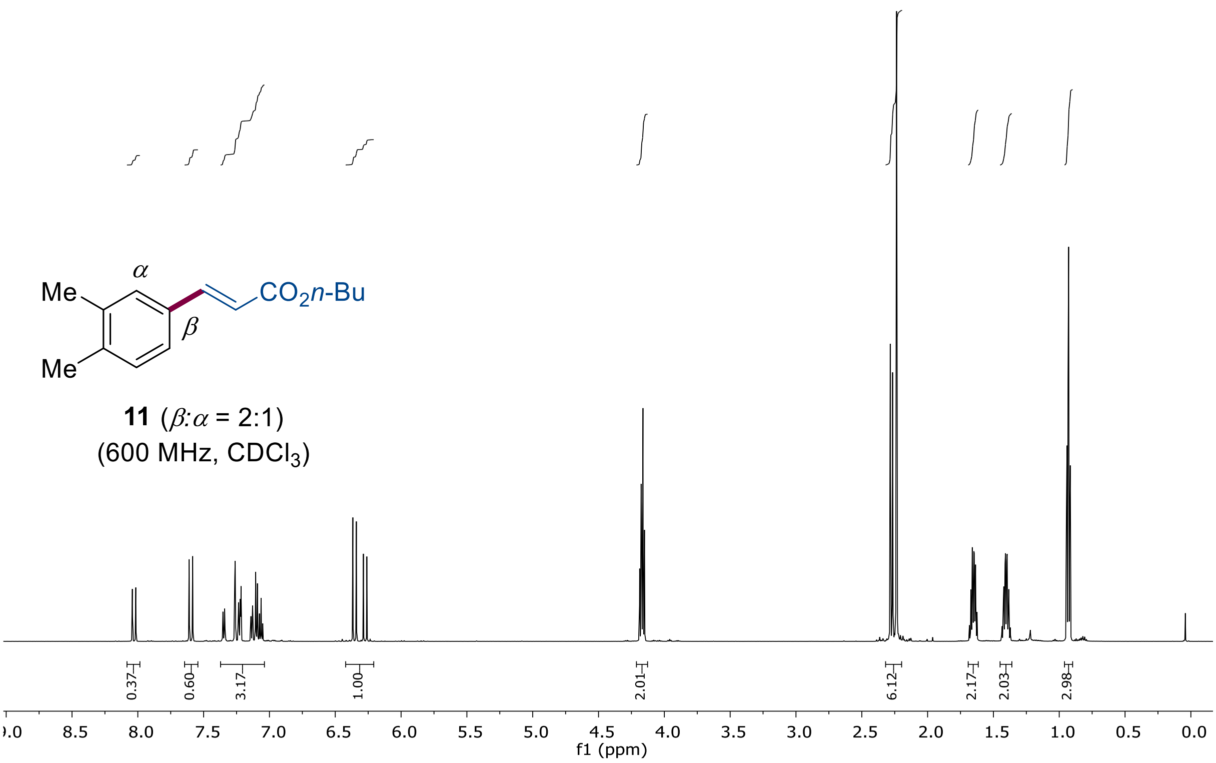

Supplementary Figure 74 H-NMR of compound 11. 600 MHz, $CDCl_3$, RT

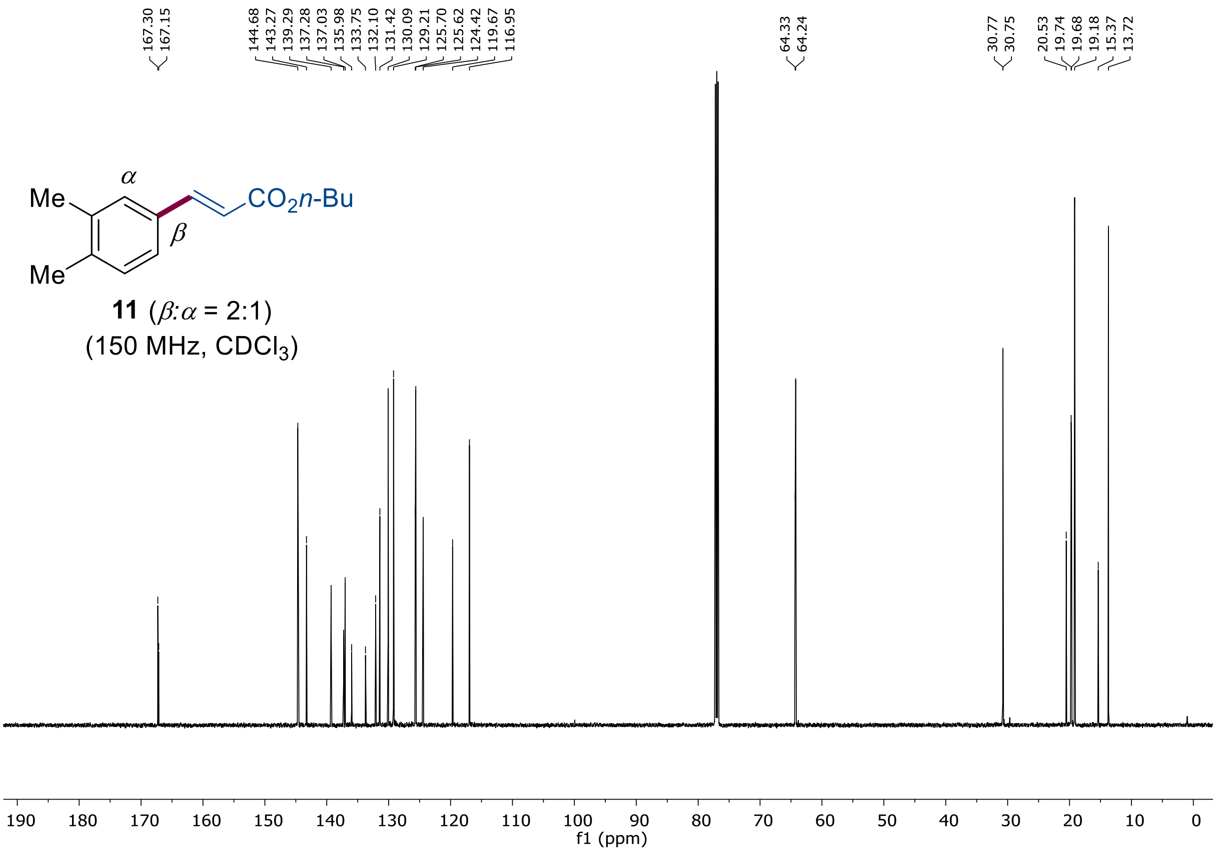

Supplementary Figure 75 C-NMR of compound 11. 150 MHz, $CDCl_3$, RT

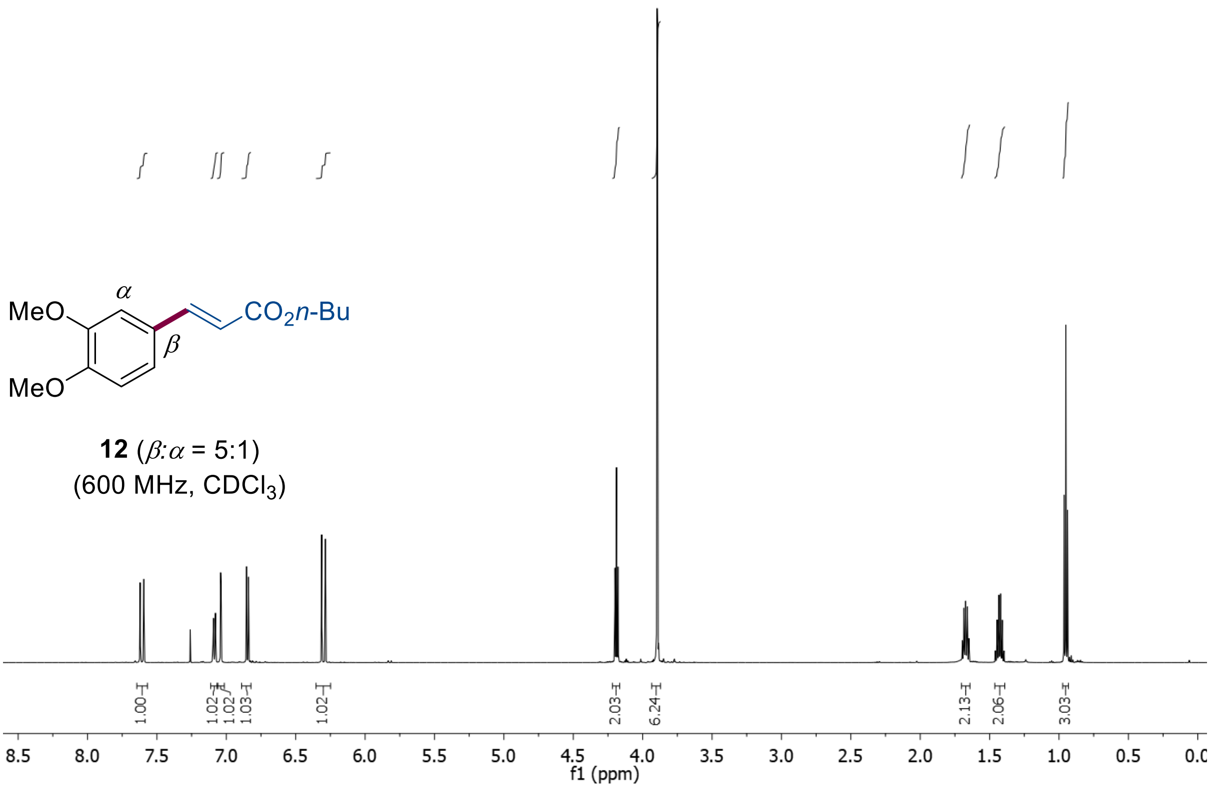

Supplementary Figure 76 H-NMR of compound 12. 600 MHz, $CDCl_3$, RT

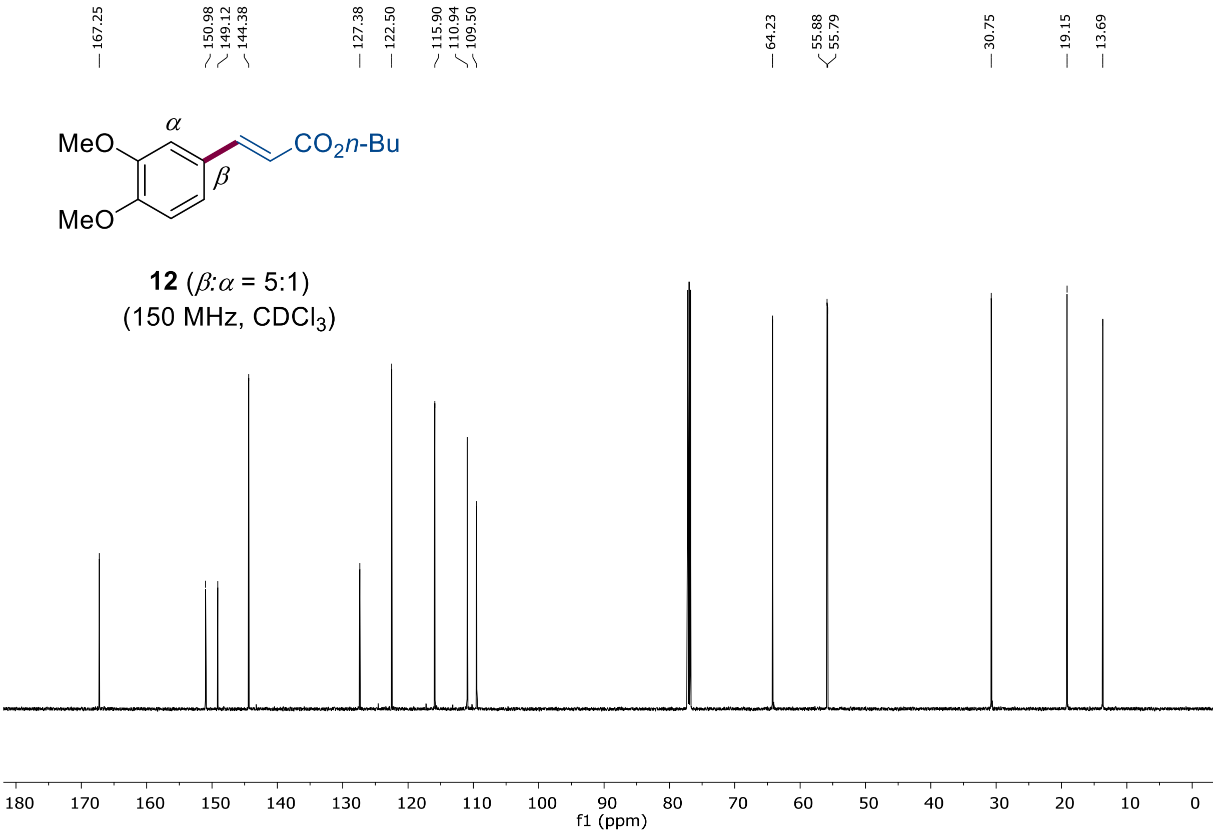

Supplementary Figure 77 C-NMR of compound 12. 150 MHz, $CDCl_3$, RT

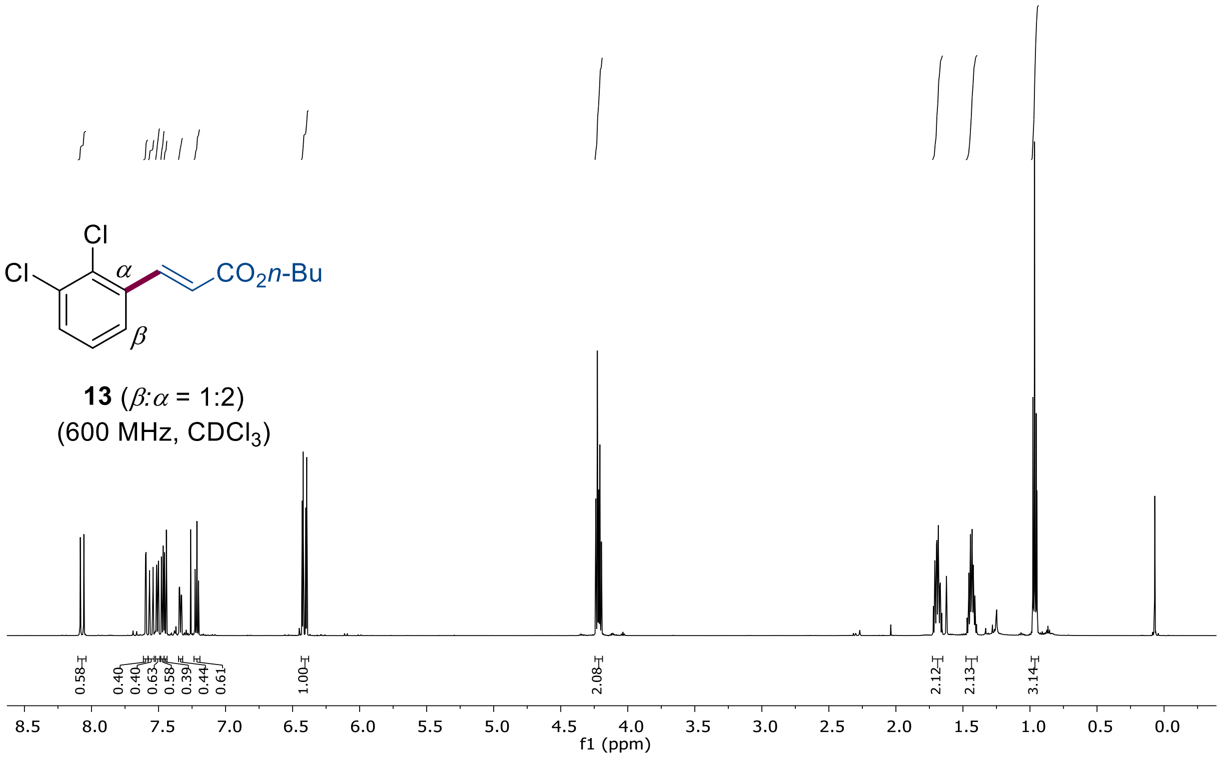

Supplementary Figure 78 H-NMR of compound 13. 600 MHz, $CDCl_3$, RT

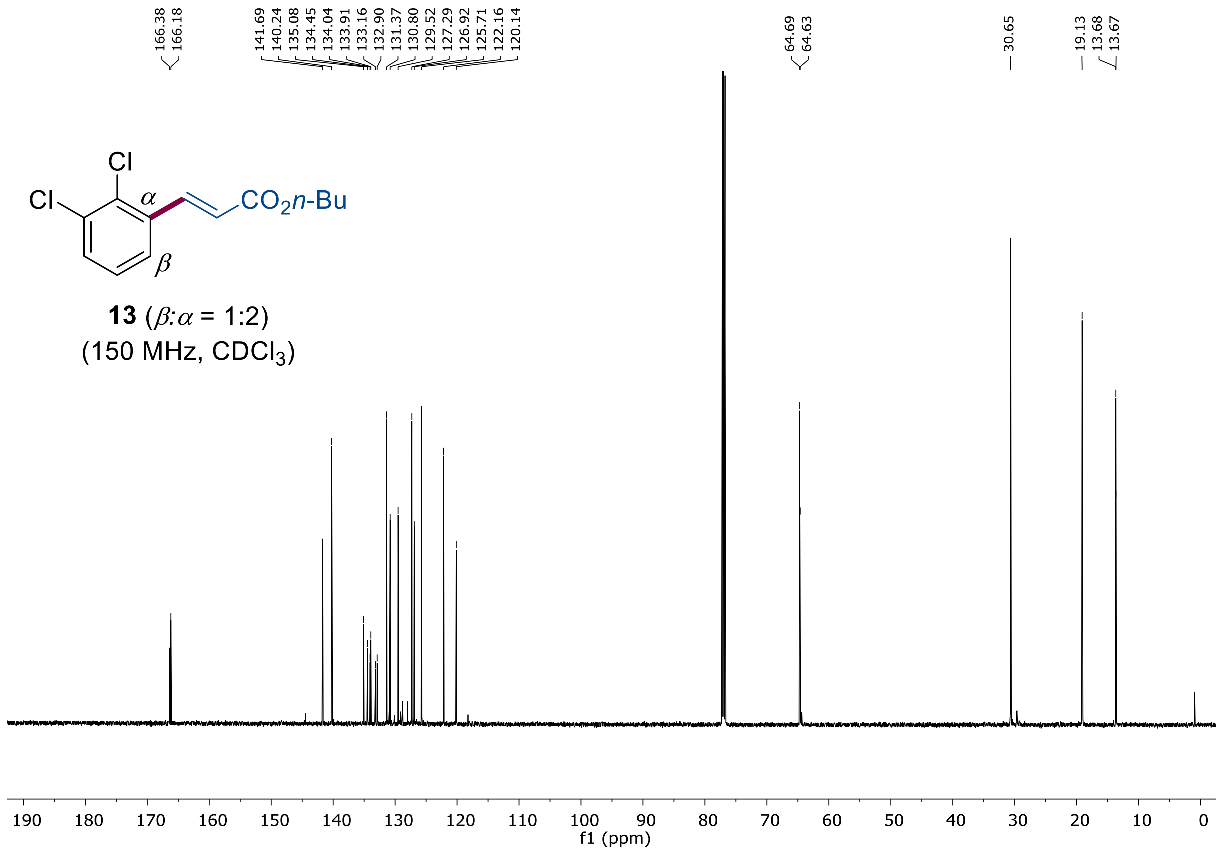

Supplementary Figure 79 C-NMR of compound 13. 150 MHz, $CDCl_3$, RT

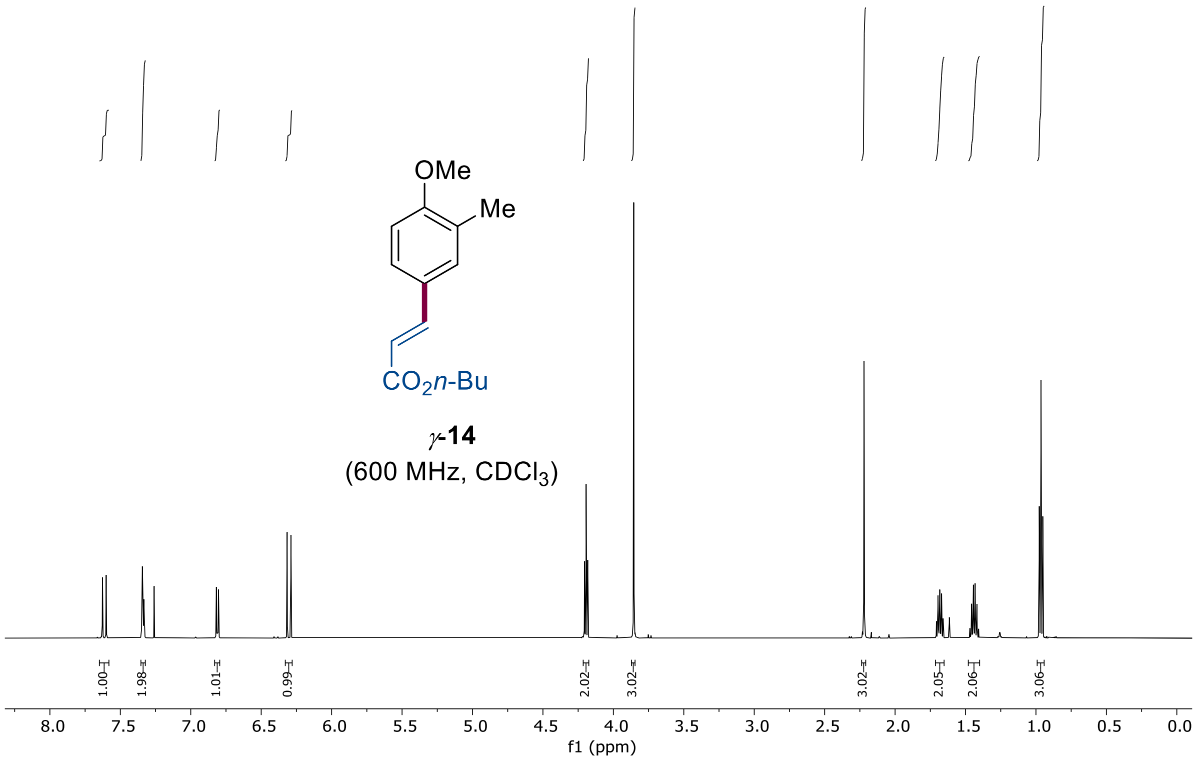

Supplementary Figure 80 H-NMR of compound *γ*-14. 600 MHz, $CDCl_3$, RT

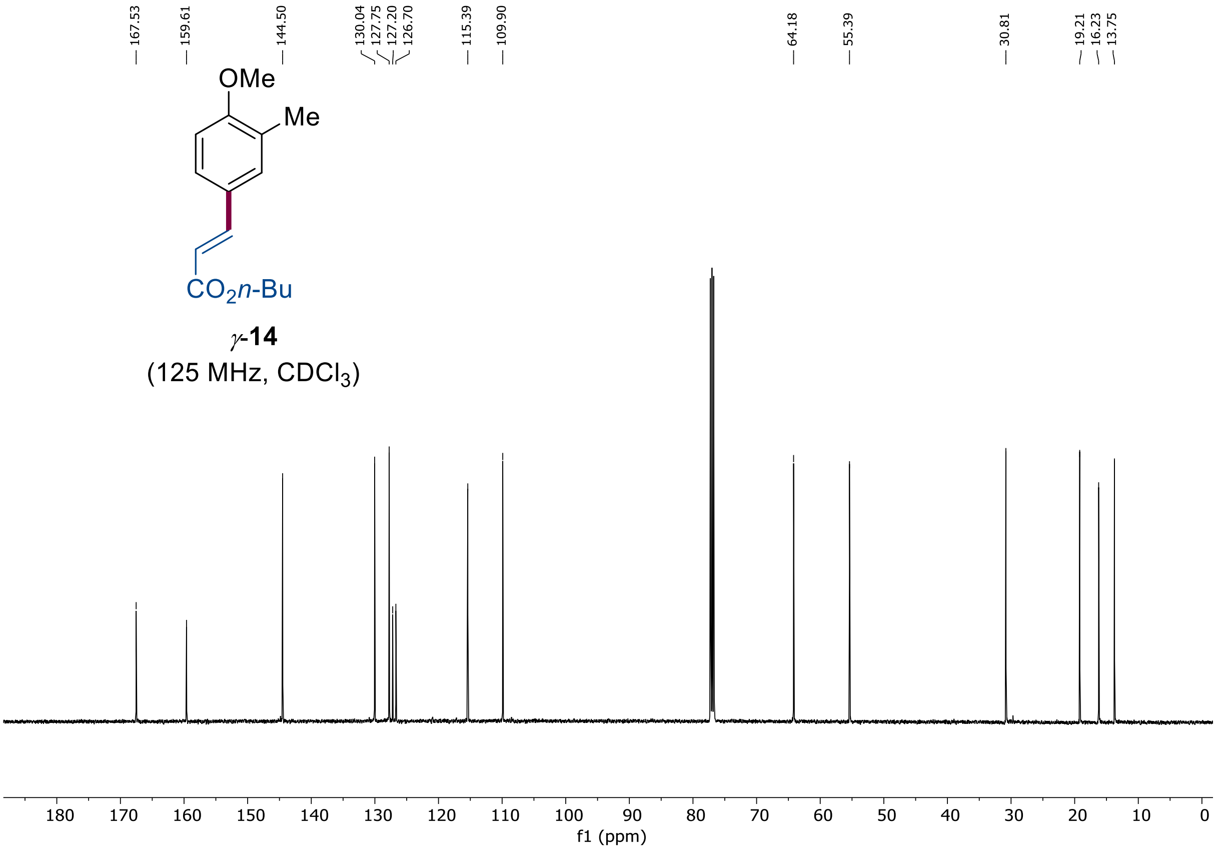

Supplementary Figure 81 C-NMR of compound *γ*-14. 125 MHz, $CDCl_3$, RT

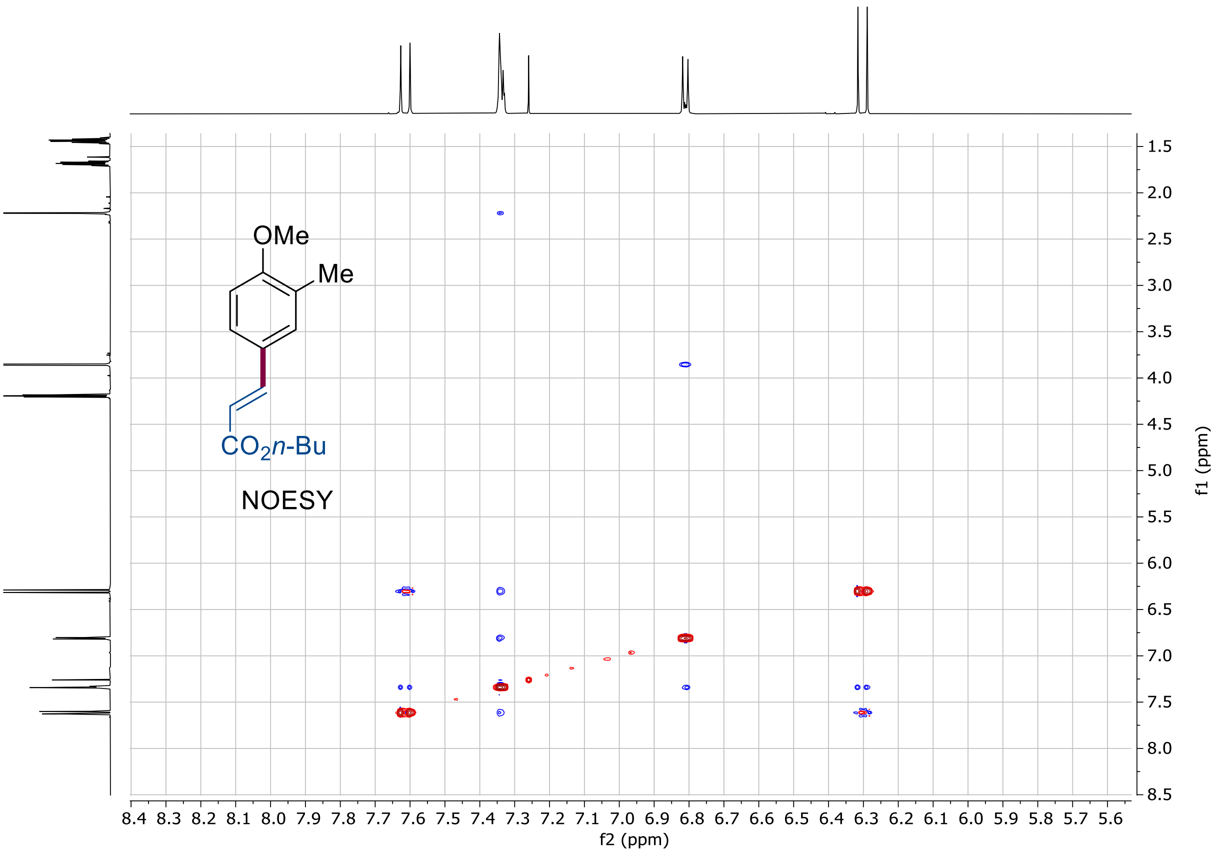

Supplementary Figure 82 NOESY-NMR of compound *γ*-14. $CDCl_3$, RT

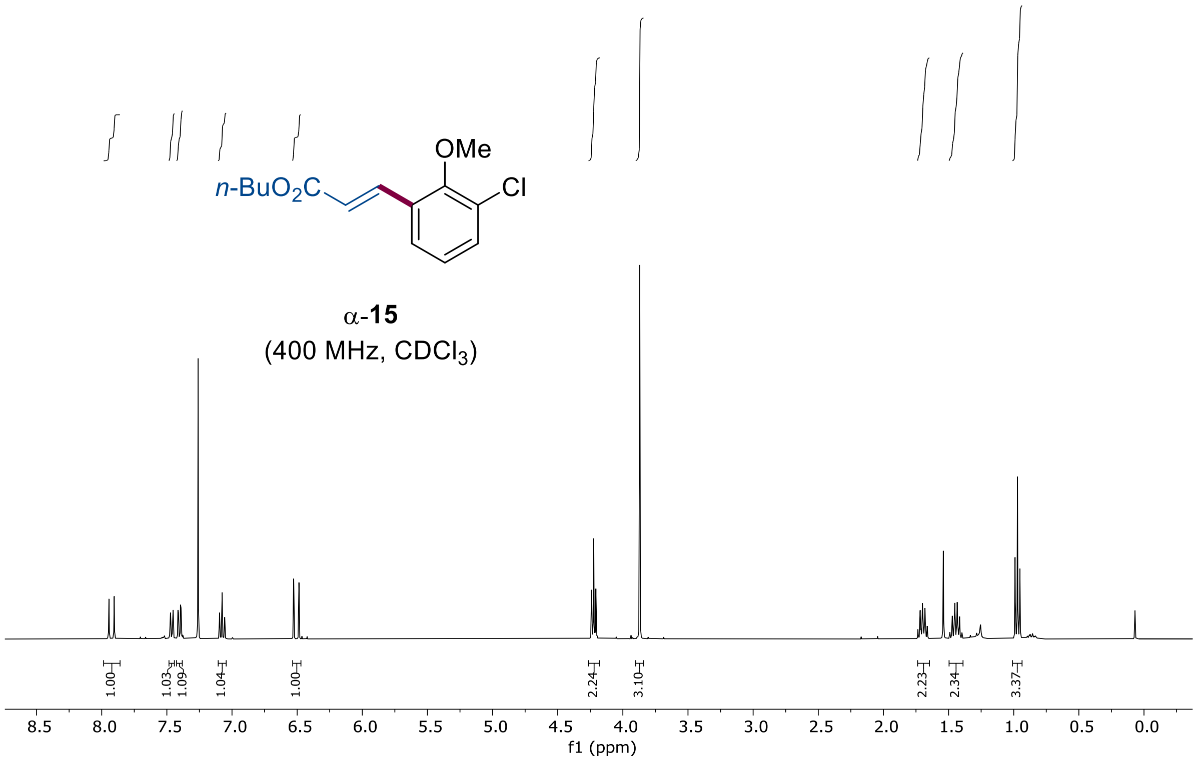

Supplementary Figure 83 H-NMR of compound *α*-15. 400 MHz, $CDCl_3$, RT

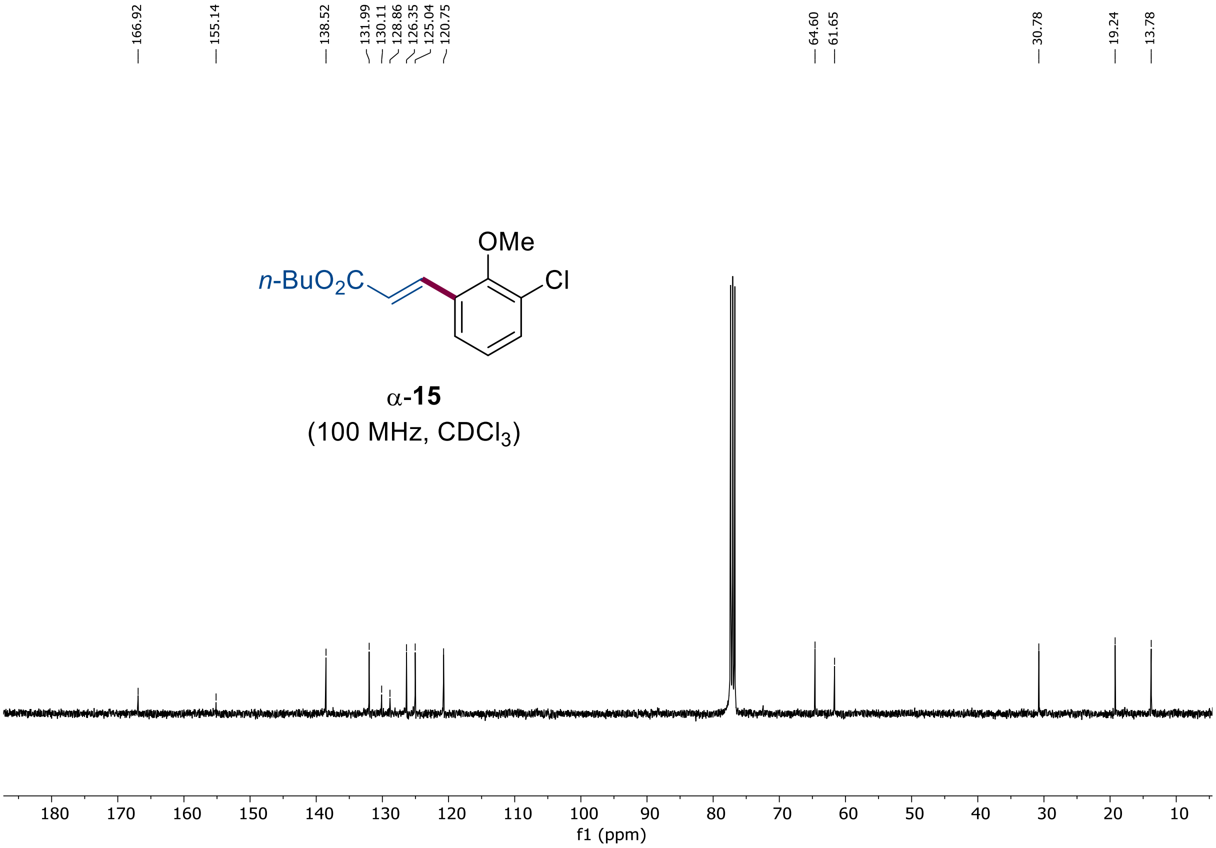

Supplementary Figure 84 C-NMR of compound *α*-15. 100 MHz, $CDCl_3$, RT

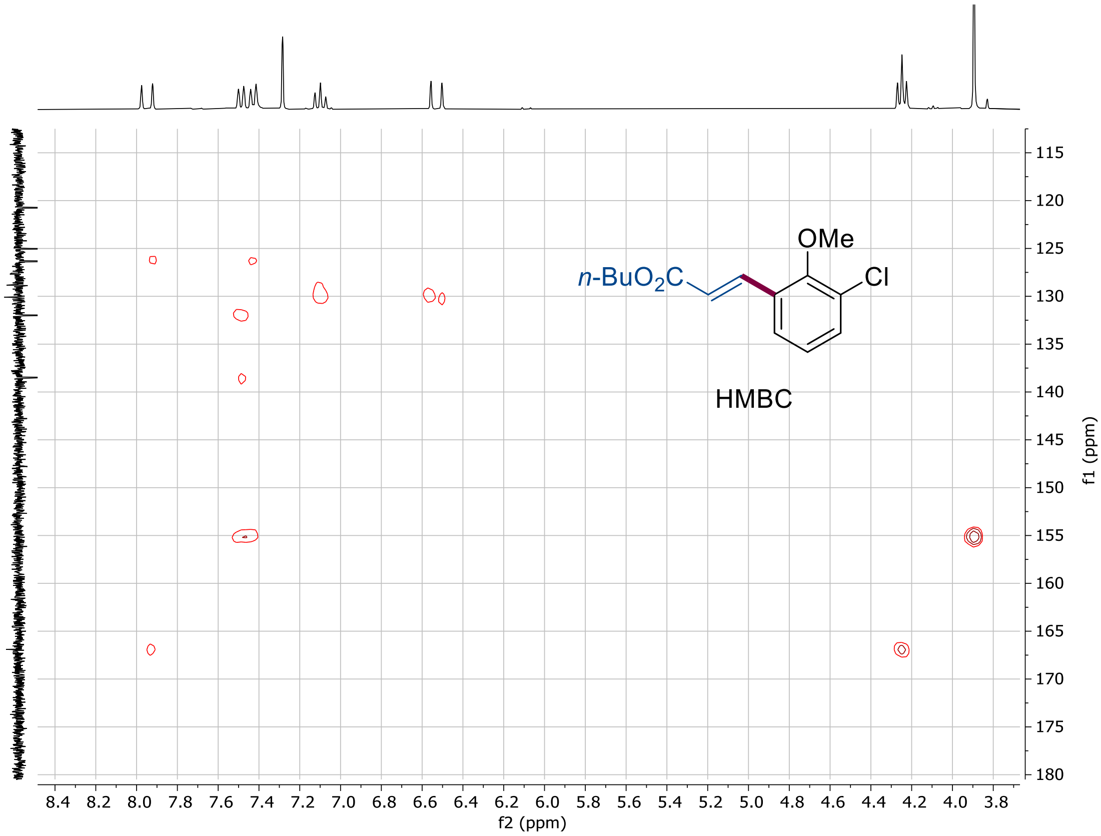

Supplementary Figure 85 HMBC-NMR of compound *α*-15. $CDCl_3$, RT

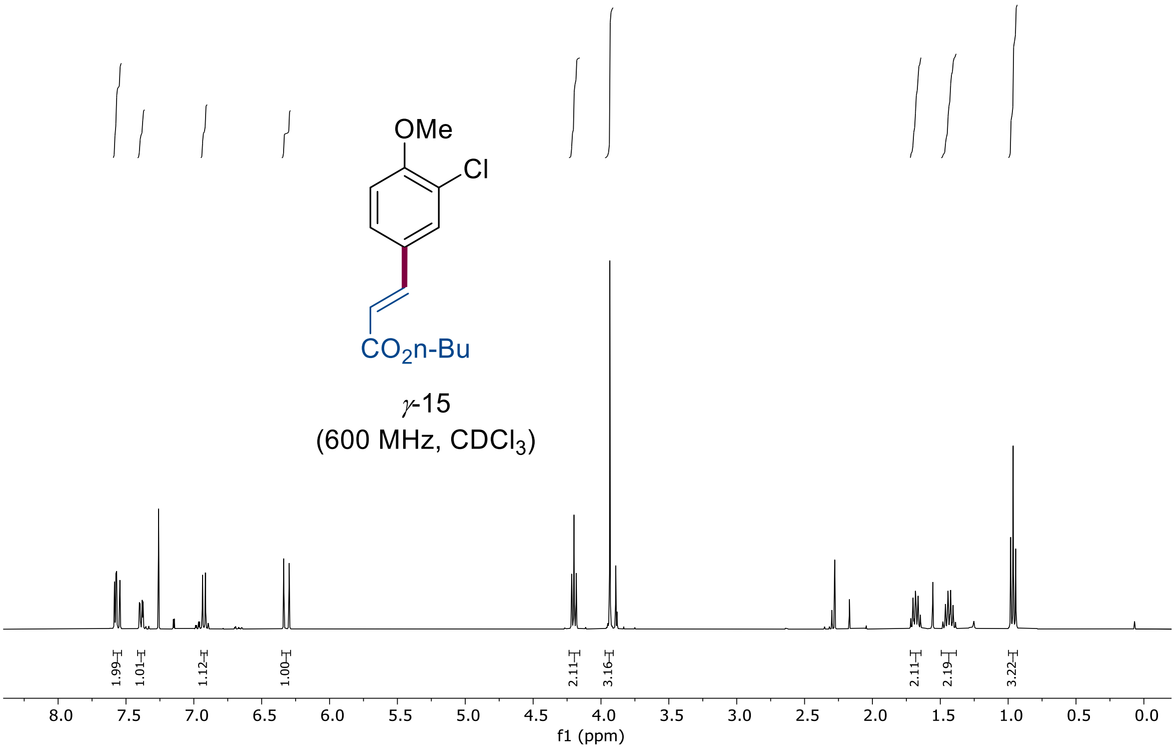

Supplementary Figure 86 H-NMR of compound *γ*-15. 600 MHz, $CDCl_3$, RT

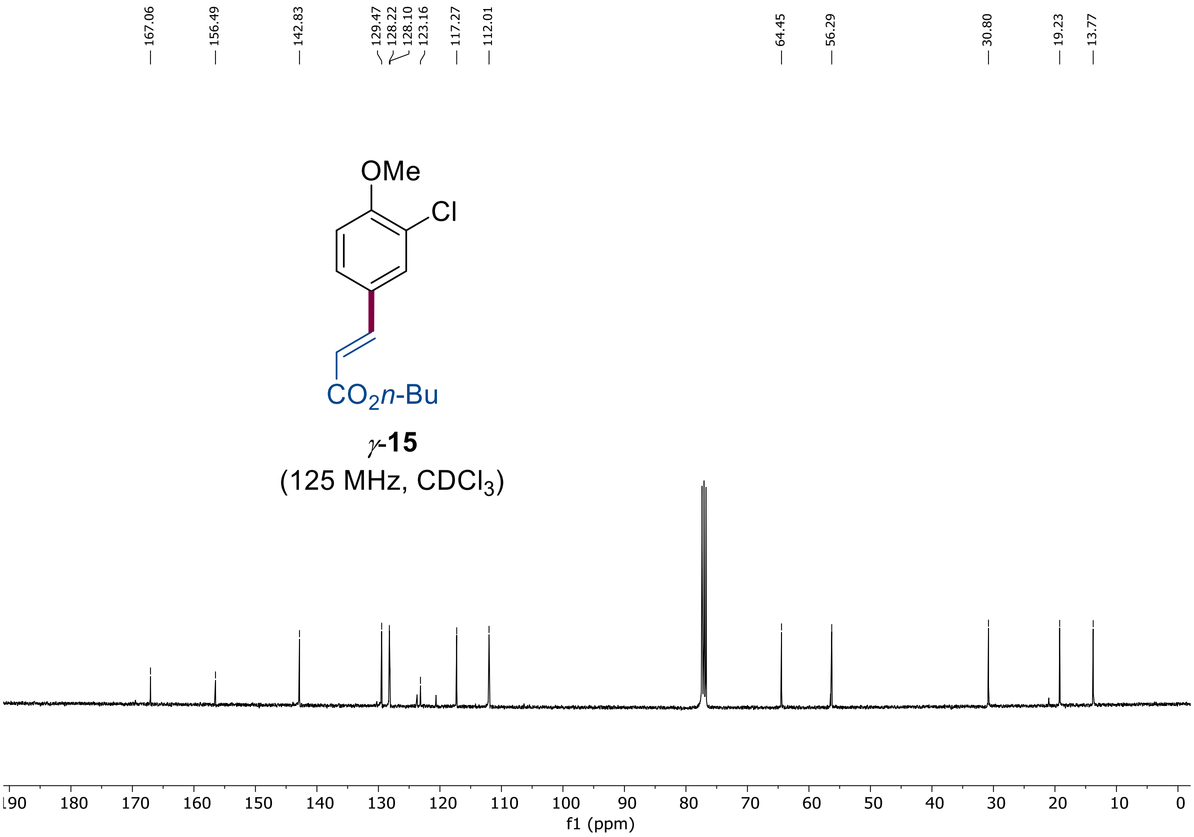

Supplementary Figure 87 C-NMR of compound *γ*-15. 125 MHz, $CDCl_3$, RT

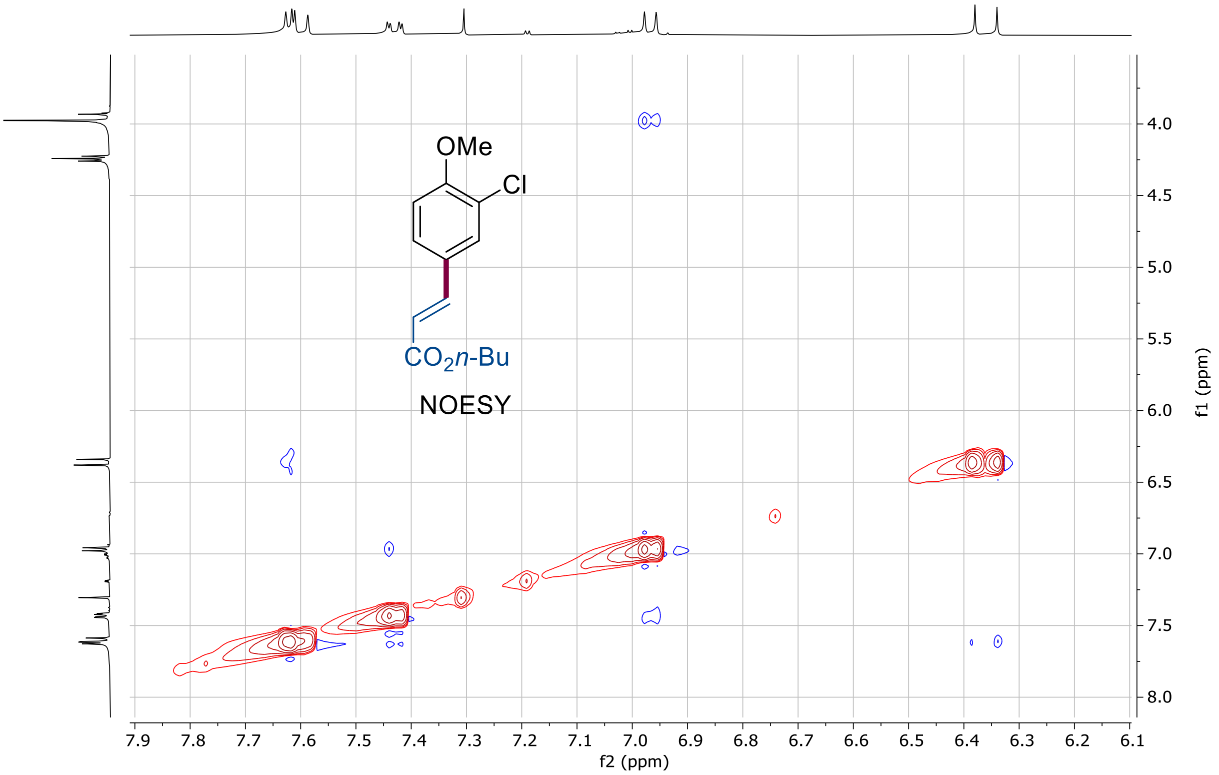

Supplementary Figure 88 NOESY-NMR of compound γ-15. $CDCl_3$, RT

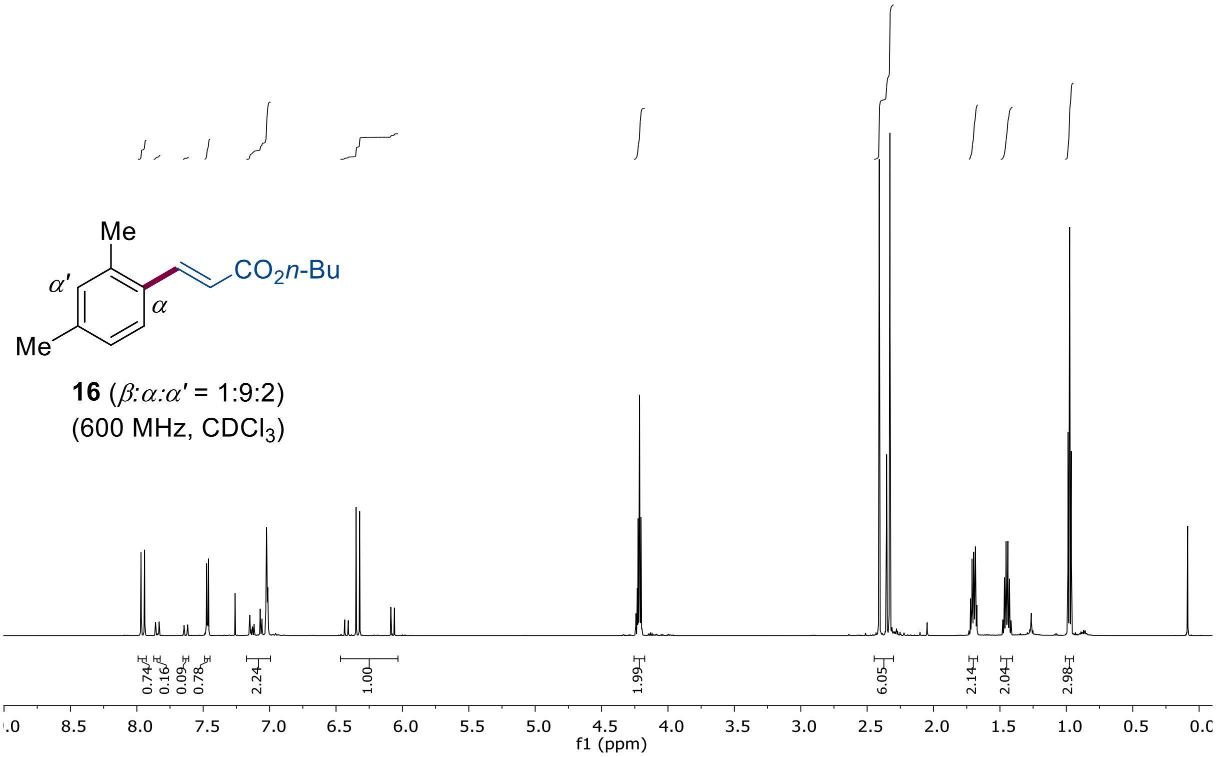

Supplementary Figure 89 H-NMR of compound 16. 600 MHz, $CDCl_3$, RT

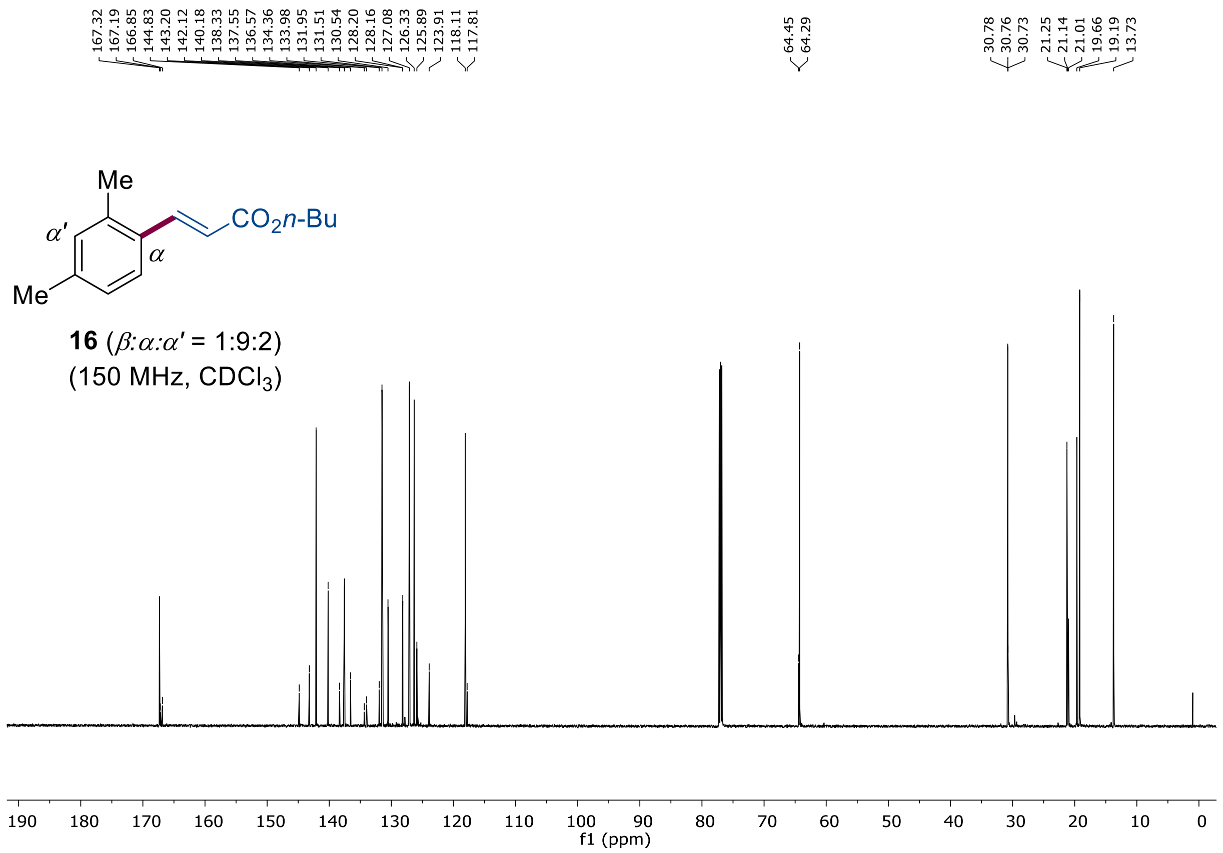

Supplementary Figure 90 C-NMR of compound 16. 150 MHz, $CDCl_3$, RT

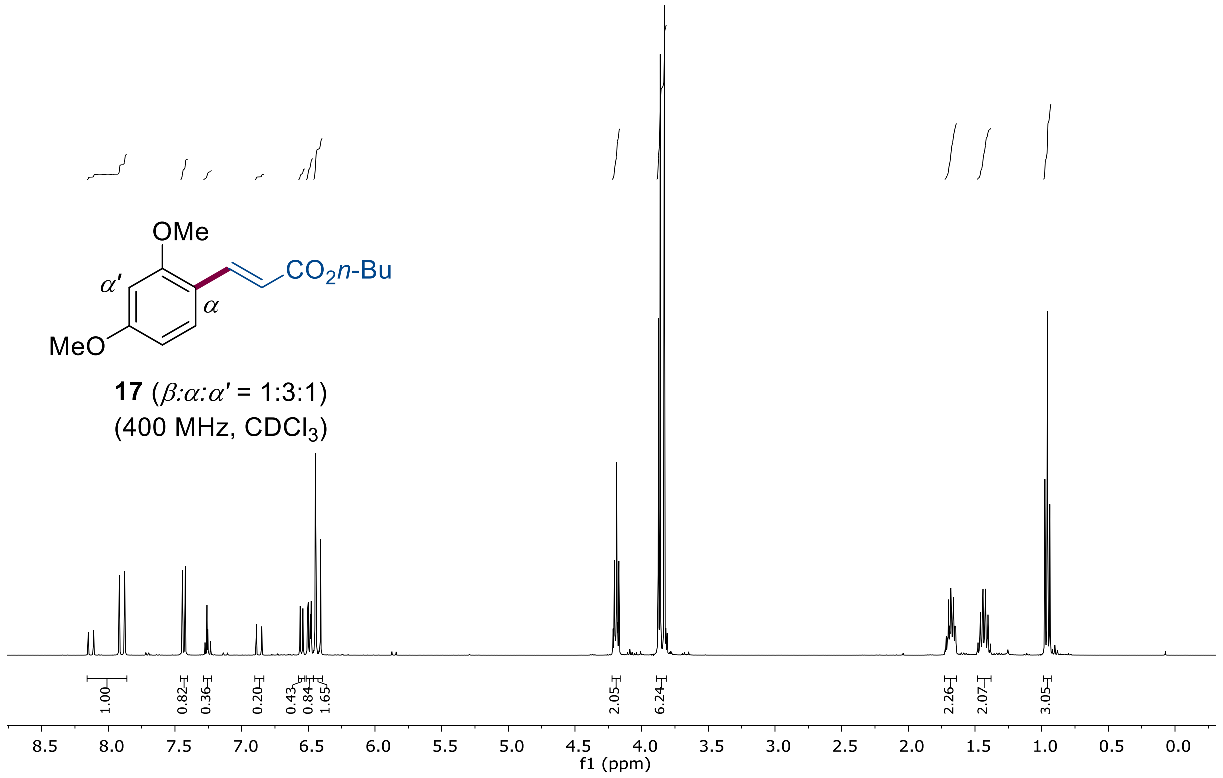

Supplementary Figure 91 H-NMR of compound 17. 400 MHz, $CDCl_3$, RT

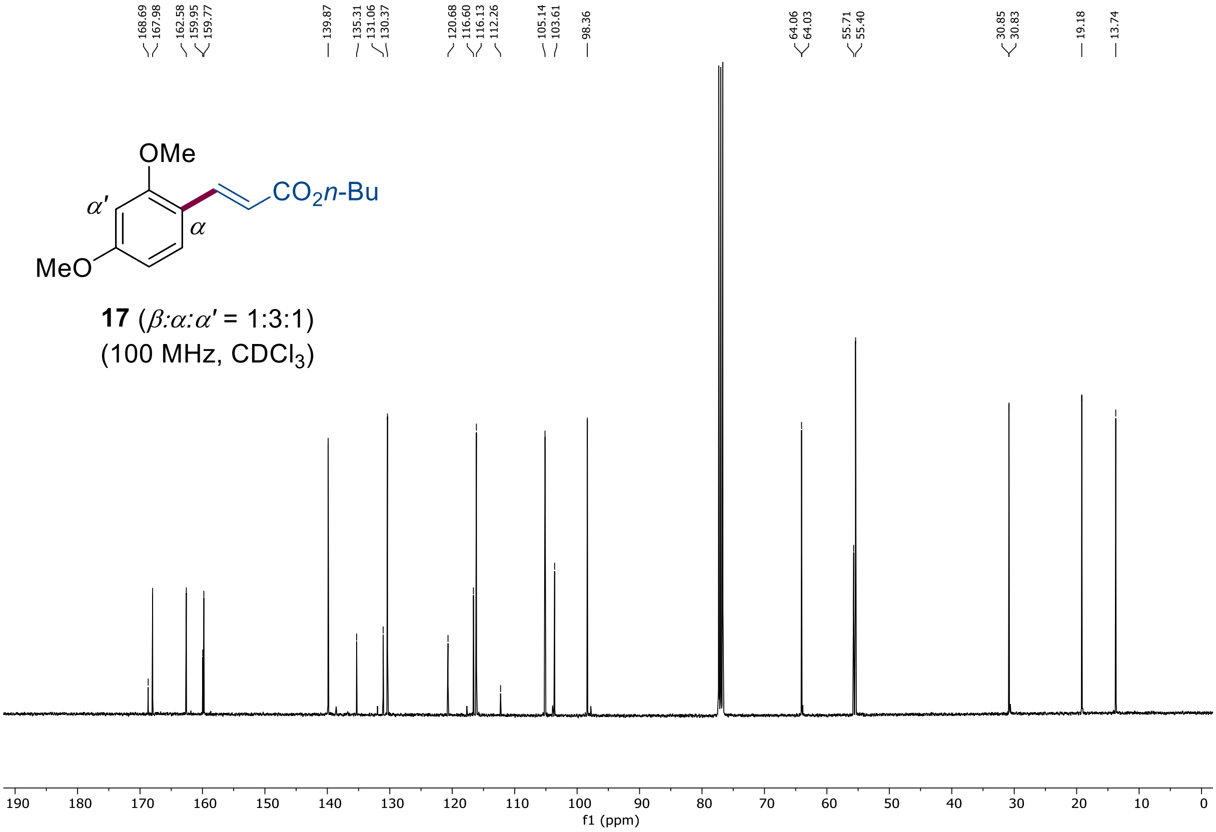

Supplementary Figure 92 **C-NMR of compound 17. 100 MHz, CDCl$_3$, RT**

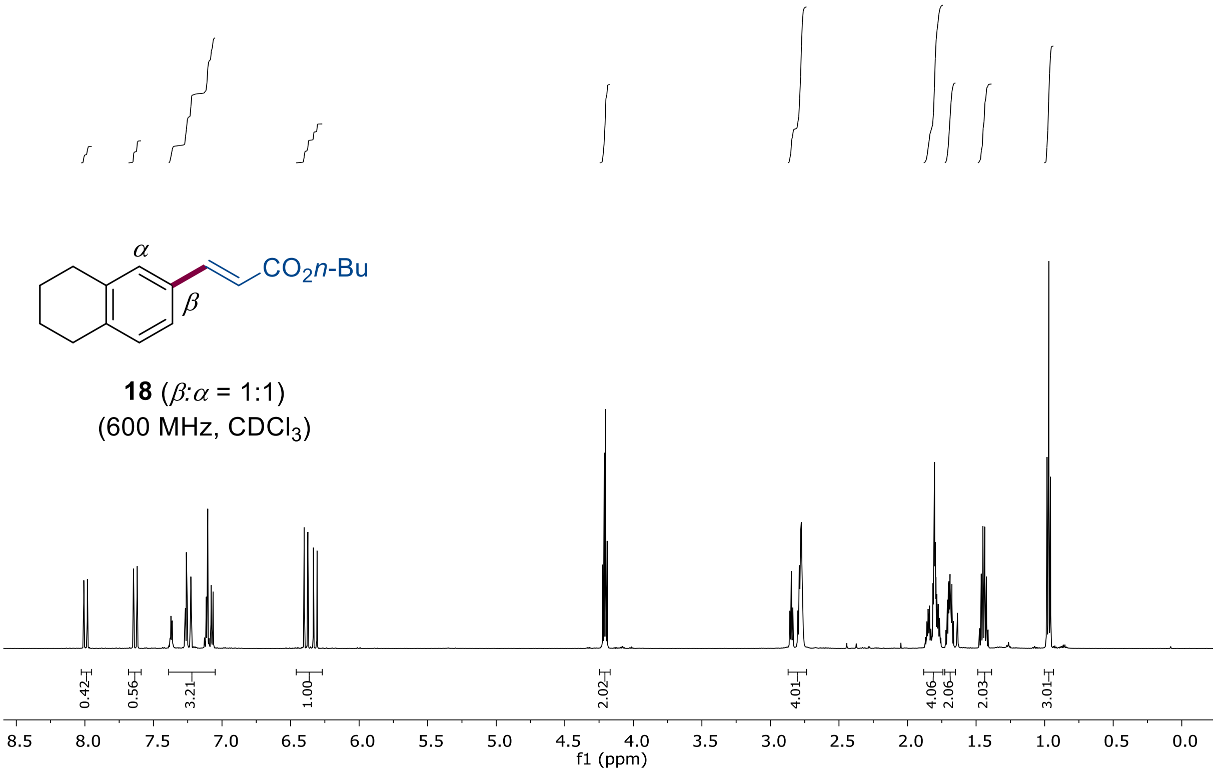

Supplementary Figure 93 **H-NMR of compound 18. 600 MHz, CDCl$_3$, RT**

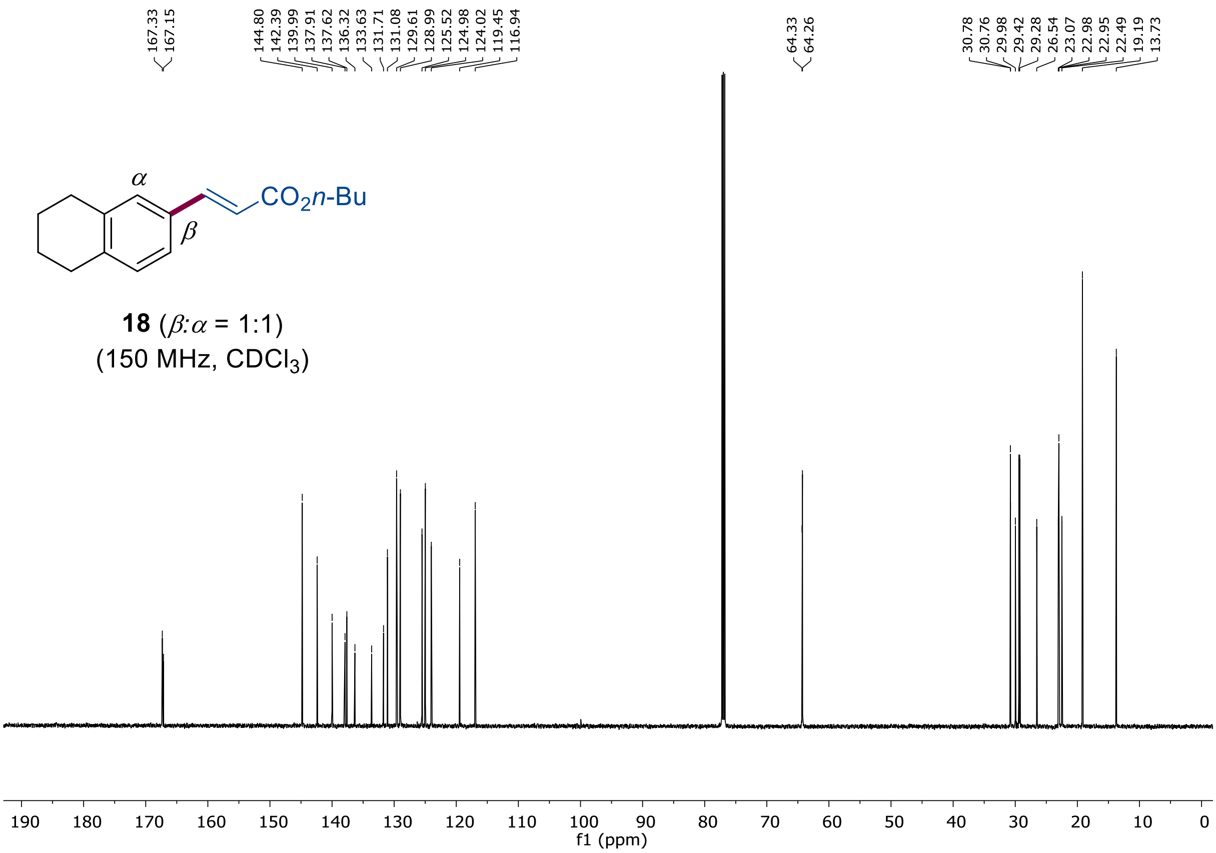

Supplementary Figure 94 C-NMR of compound 18. 150 MHz, $CDCl_3$, RT

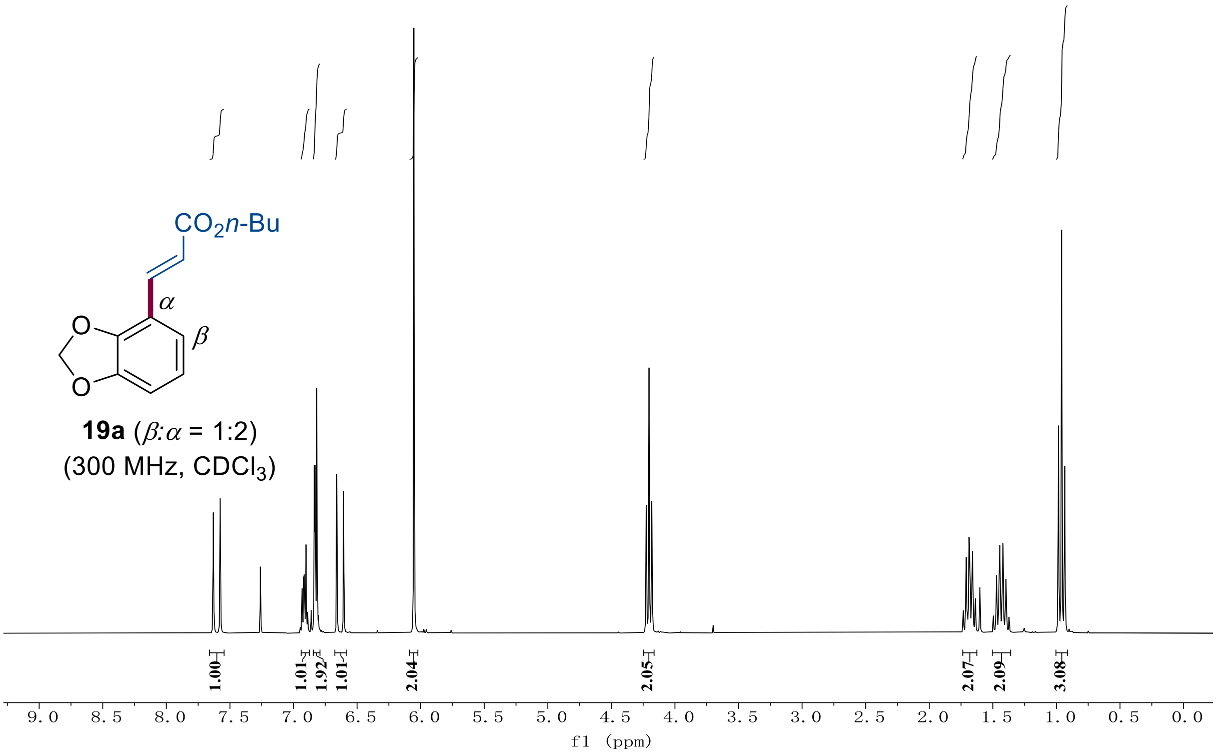

Supplementary Figure 95 H-NMR of compound 19a. 300 MHz, $CDCl_3$, RT

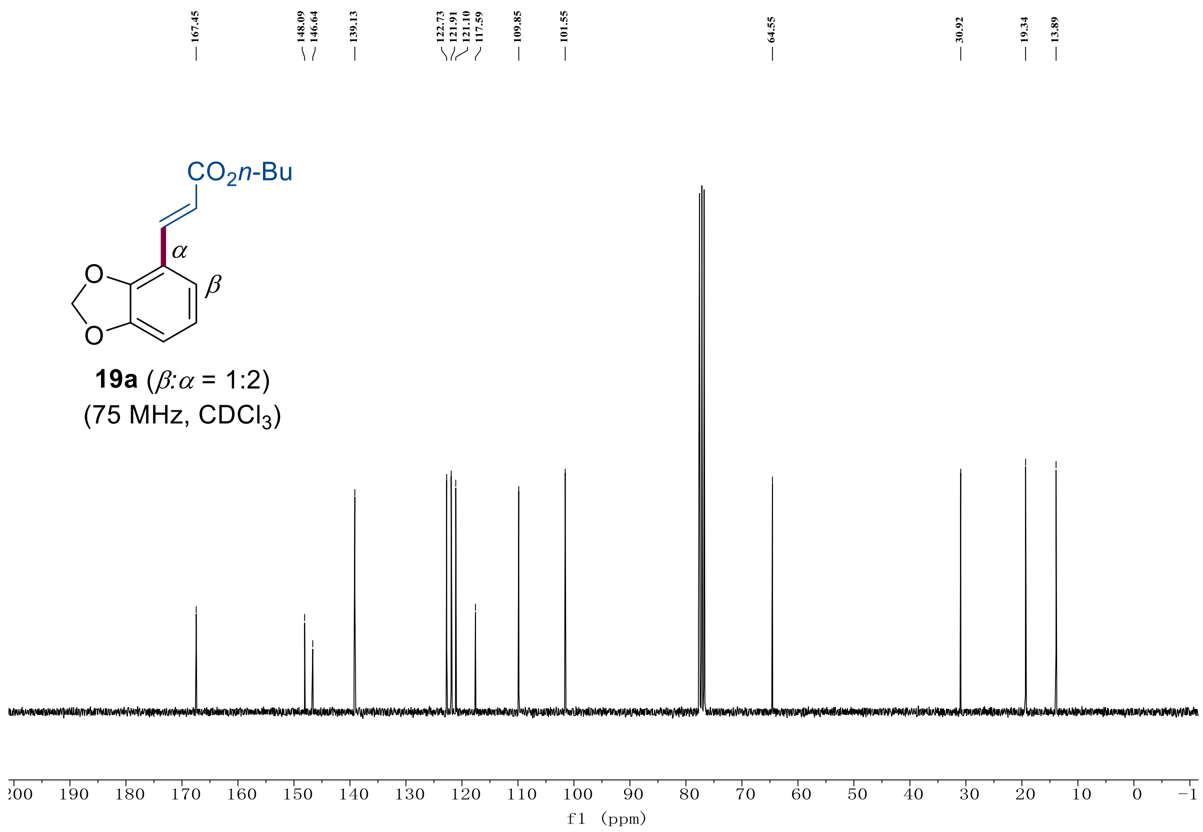

Supplementary Figure 96 C-NMR of compound 19a. 75 MHz, $CDCl_3$, RT

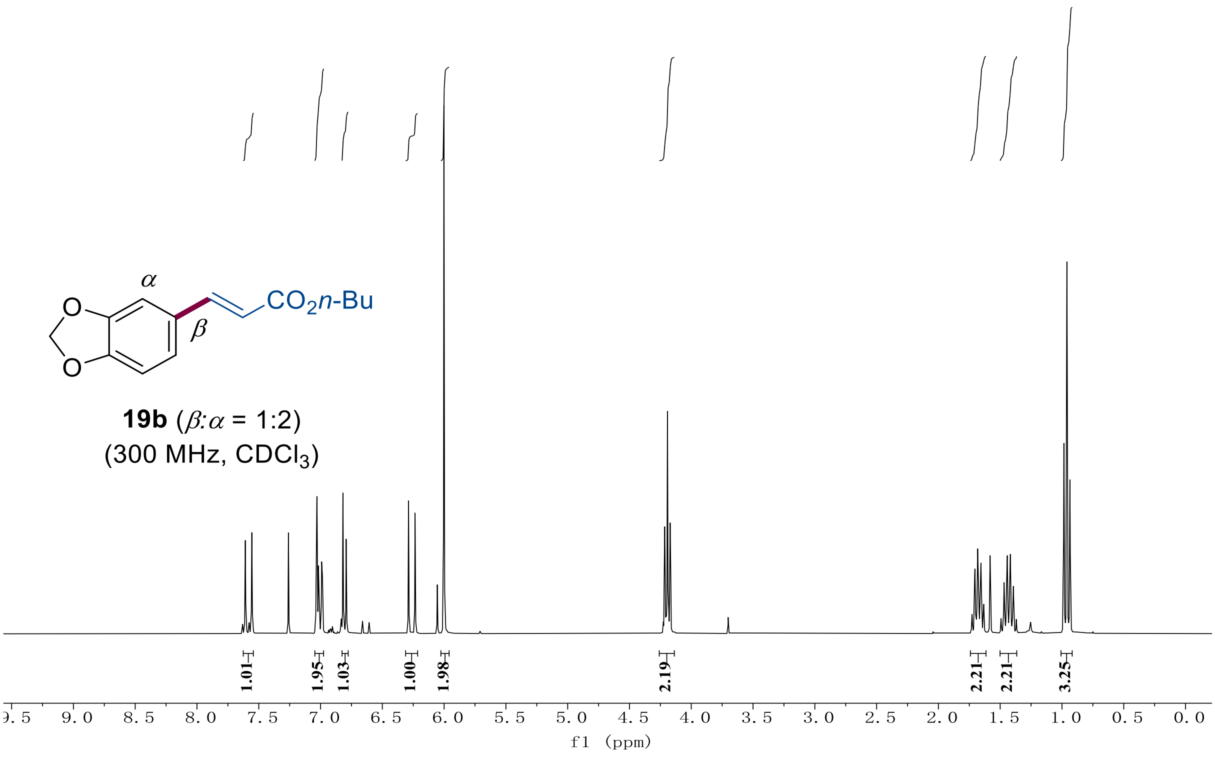

Supplementary Figure 97 H-NMR of compound 19b. 300 MHz, $CDCl_3$, RT

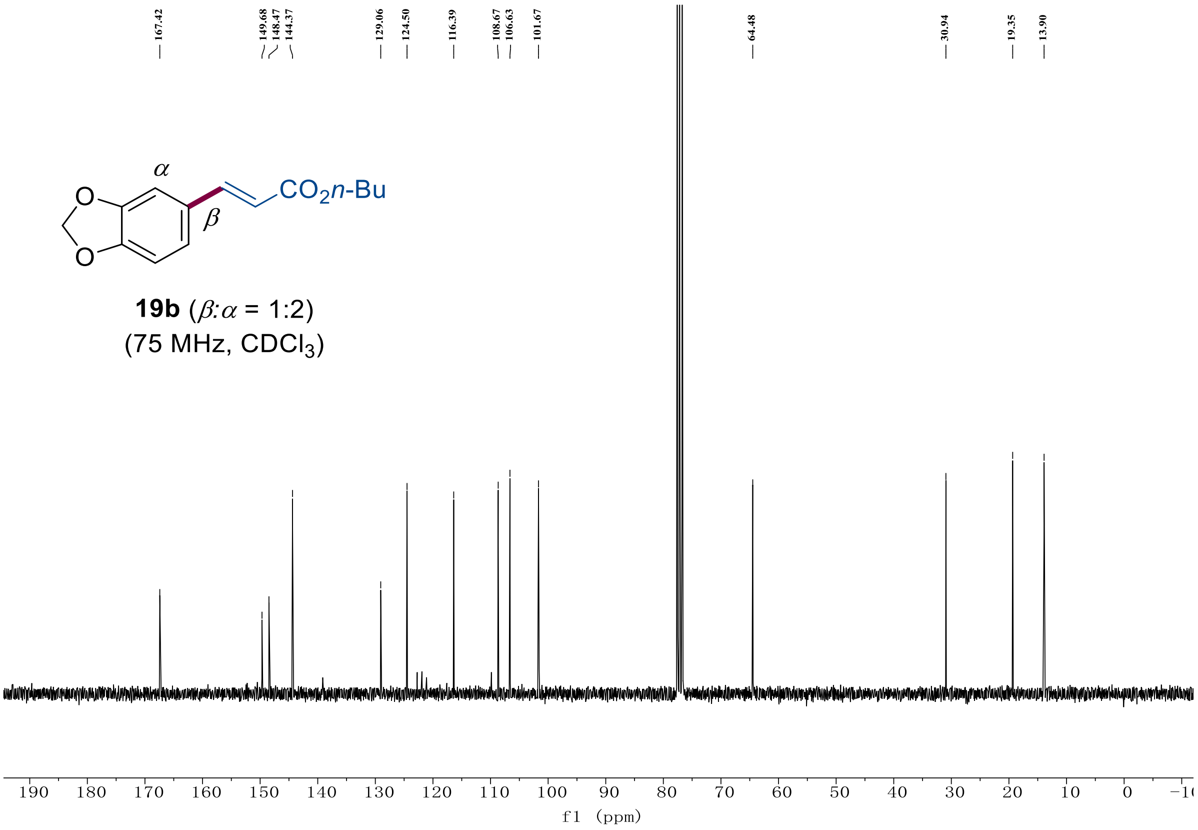

Supplementary Figure 98 C-NMR of compound 19b. 75 MHz, $CDCl_3$, RT

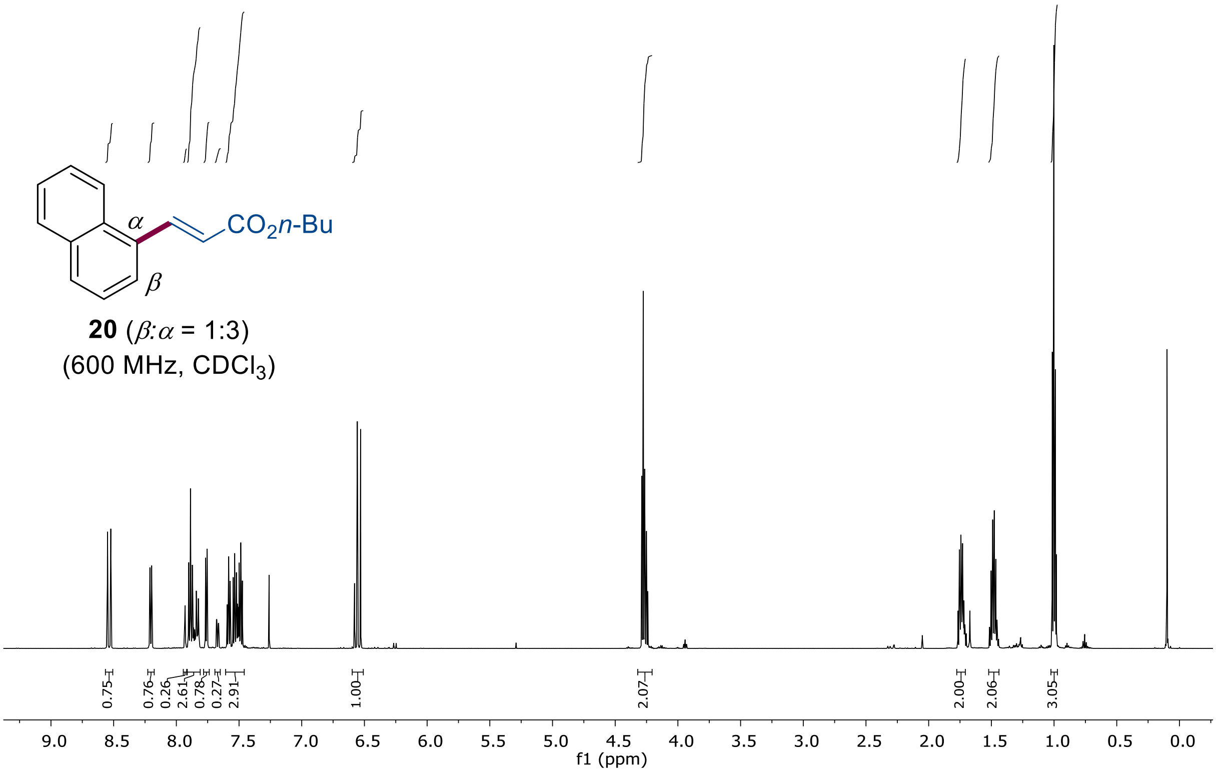

Supplementary Figure 99 H-NMR of compound 20. 600 MHz, $CDCl_3$, RT

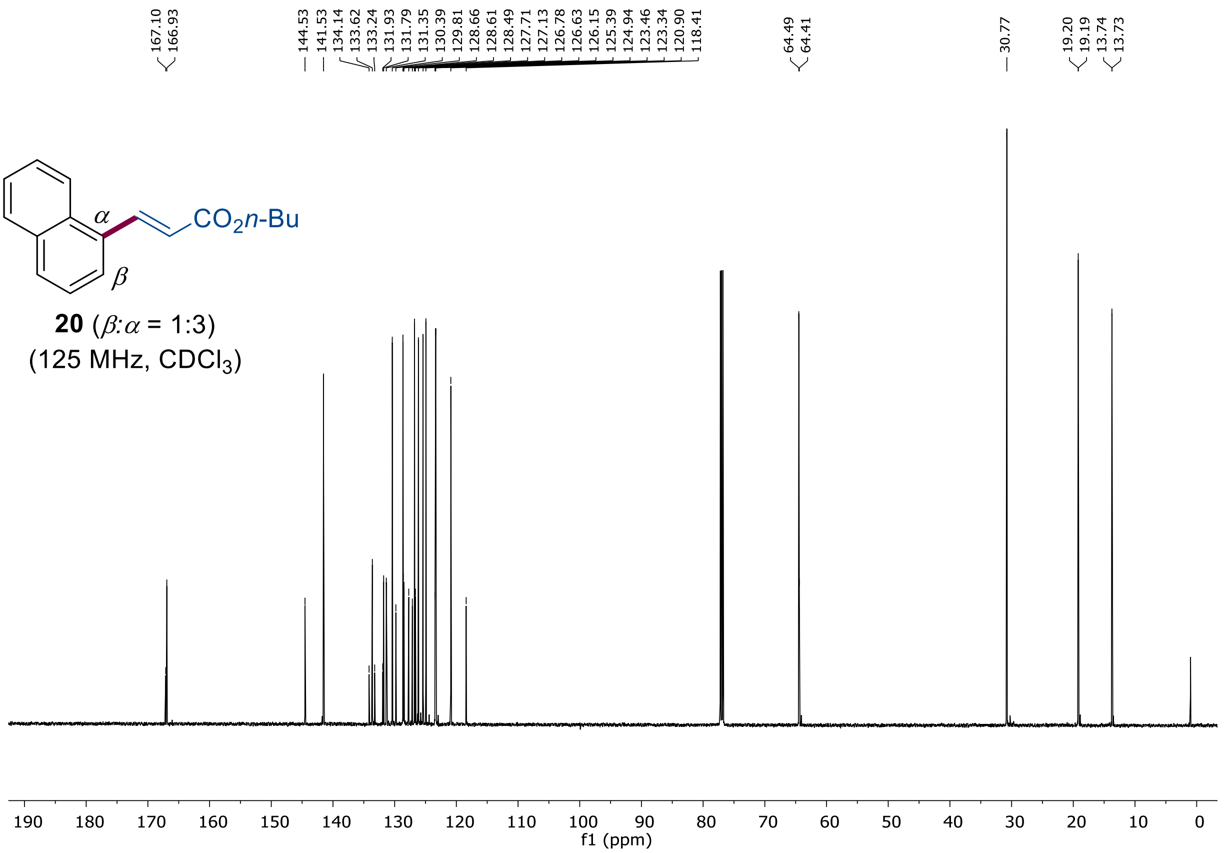

Supplementary Figure 100 C-NMR of compound 20. 125 MHz, $CDCl_3$, RT

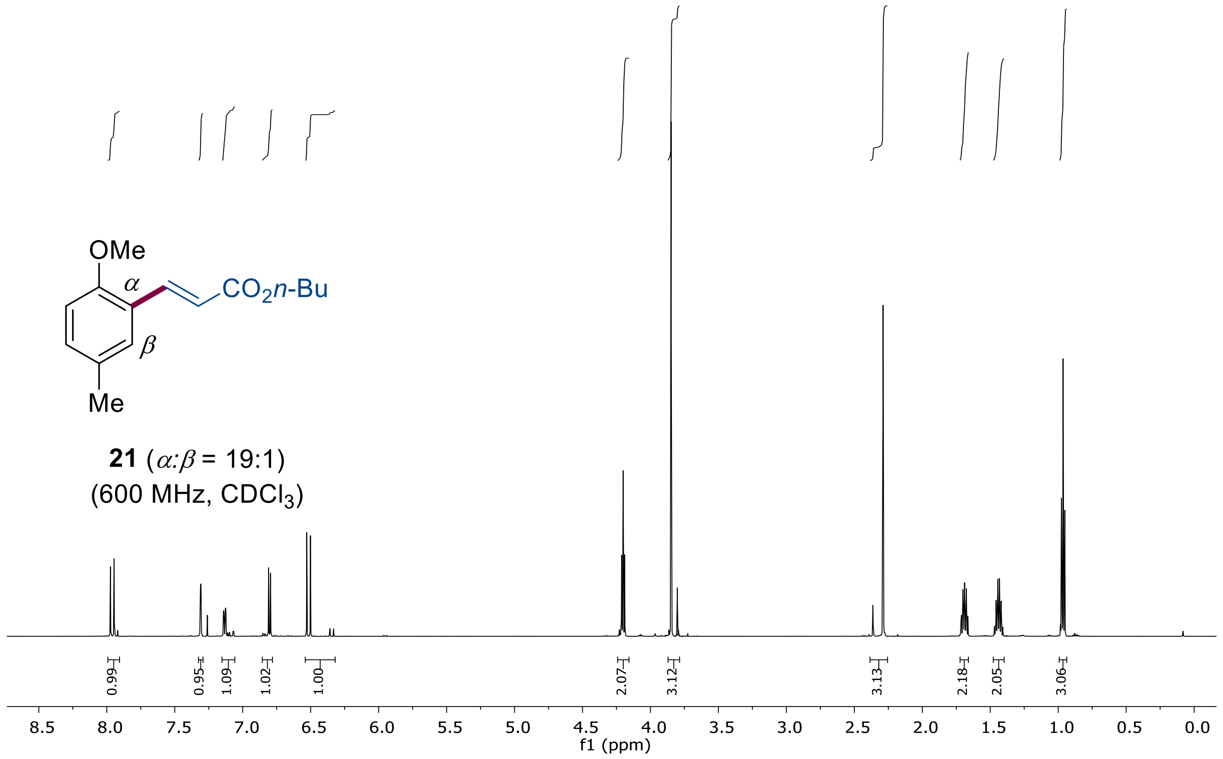

Supplementary Figure 101 H-NMR of compound 21. 600 MHz, $CDCl_3$, RT

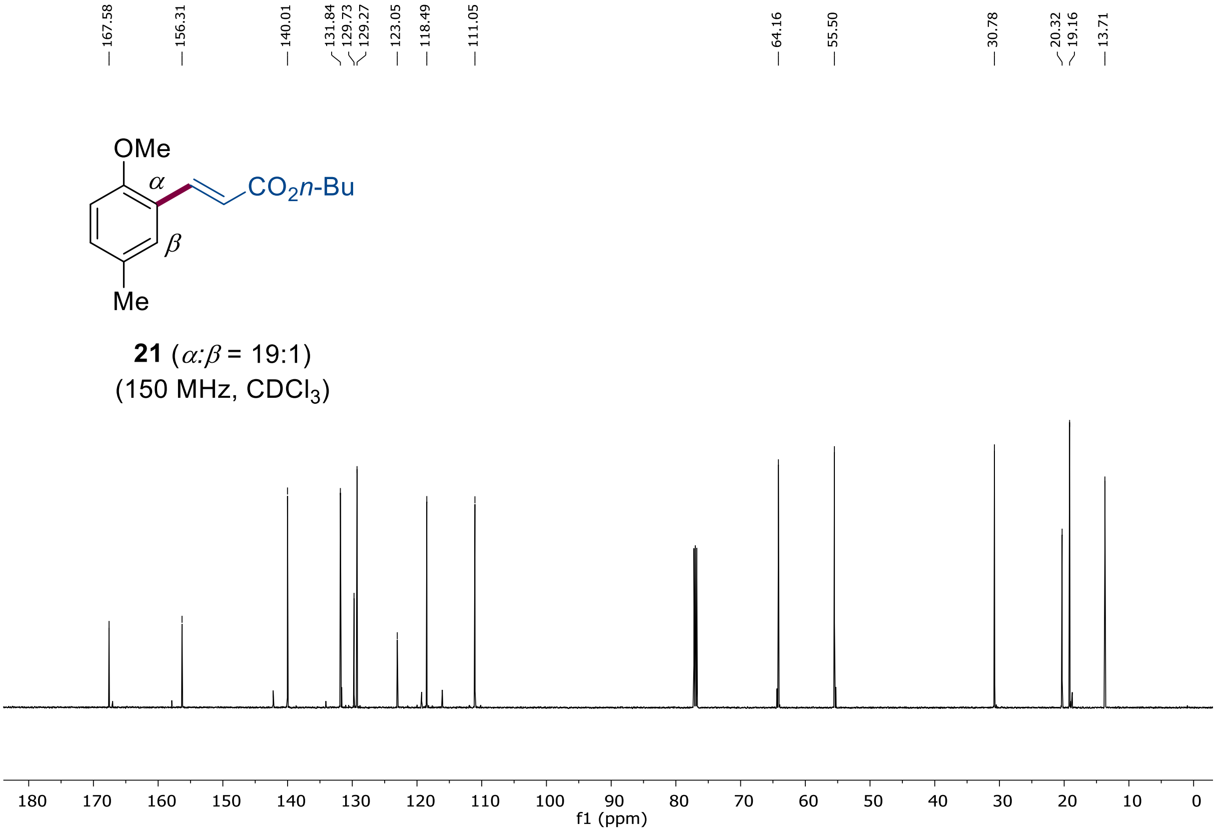

Supplementary Figure 102 C-NMR of compound 21. 150 MHz, $CDCl_3$, RT

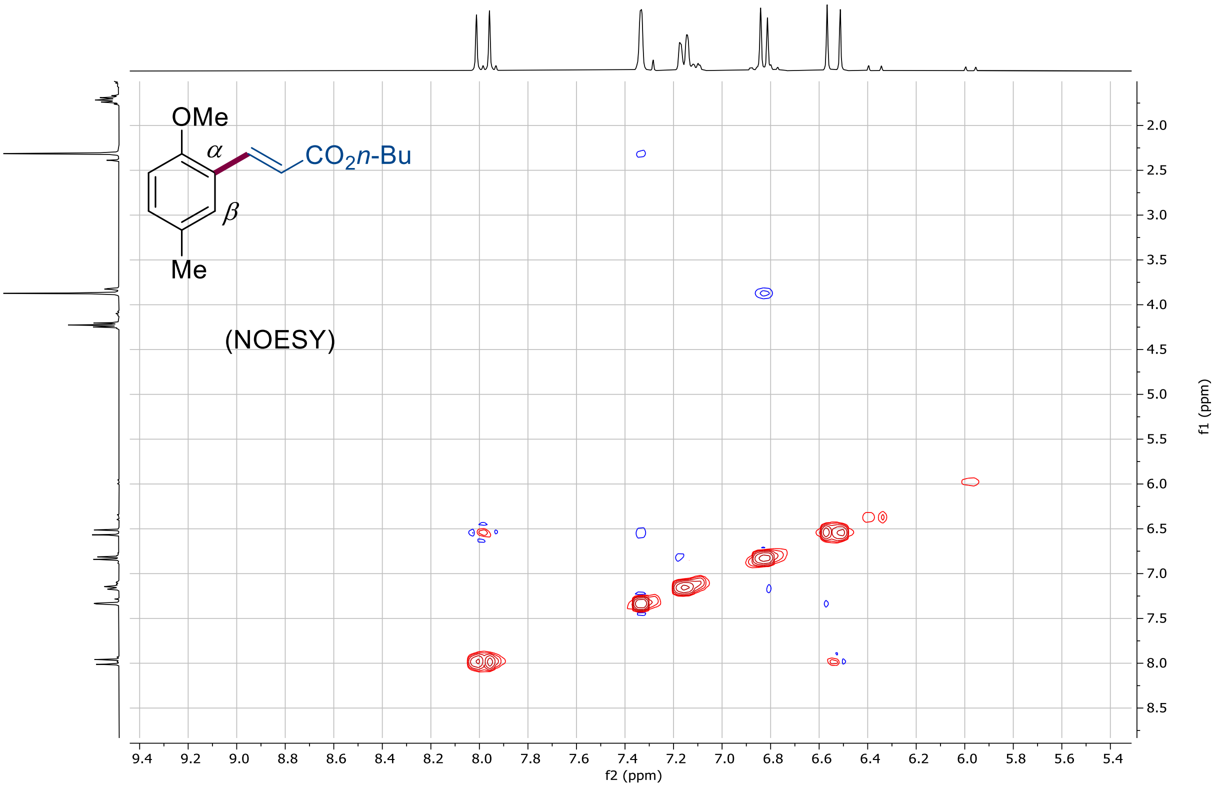

Supplementary Figure 103 NOESY-NMR of compound 21. $CDCl_3$, RT

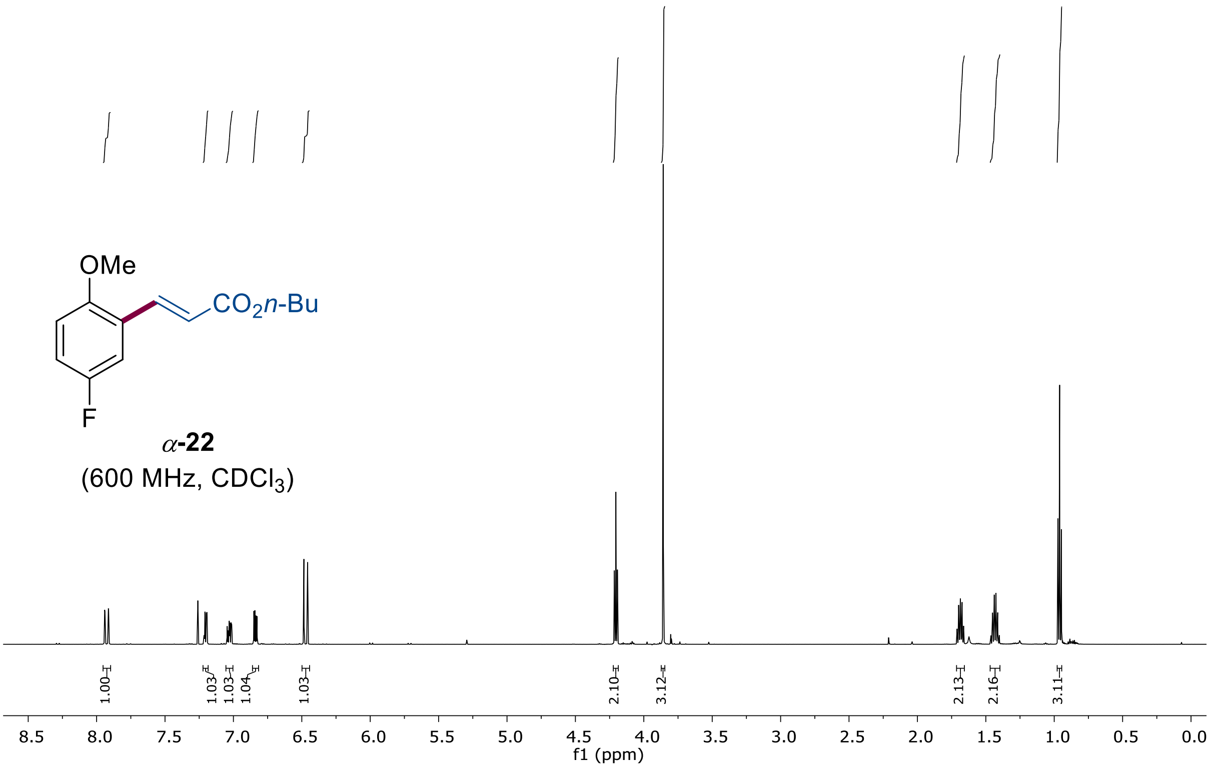

Supplementary Figure 104 H-NMR of compound α-22. 600 MHz, $CDCl_3$, RT

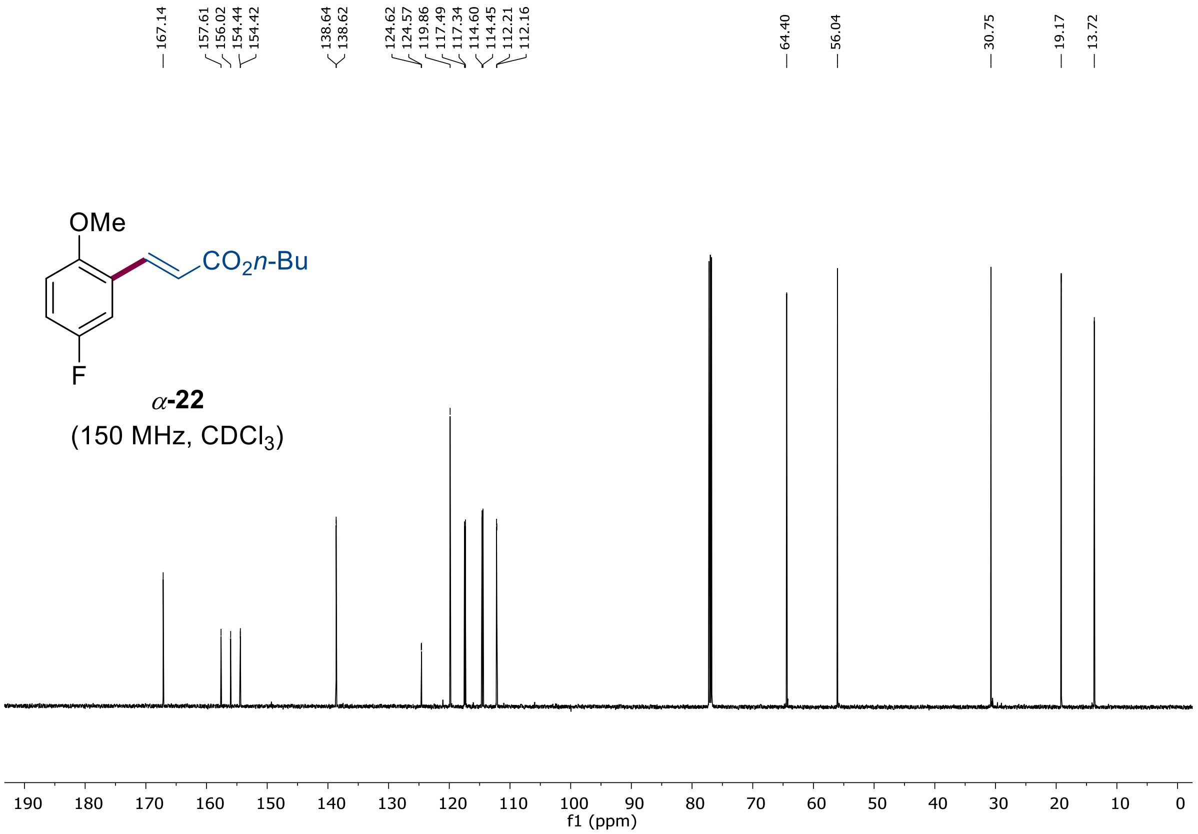

Supplementary Figure 105 C-NMR of compound α-22. 150 MHz, $CDCl_3$, RT

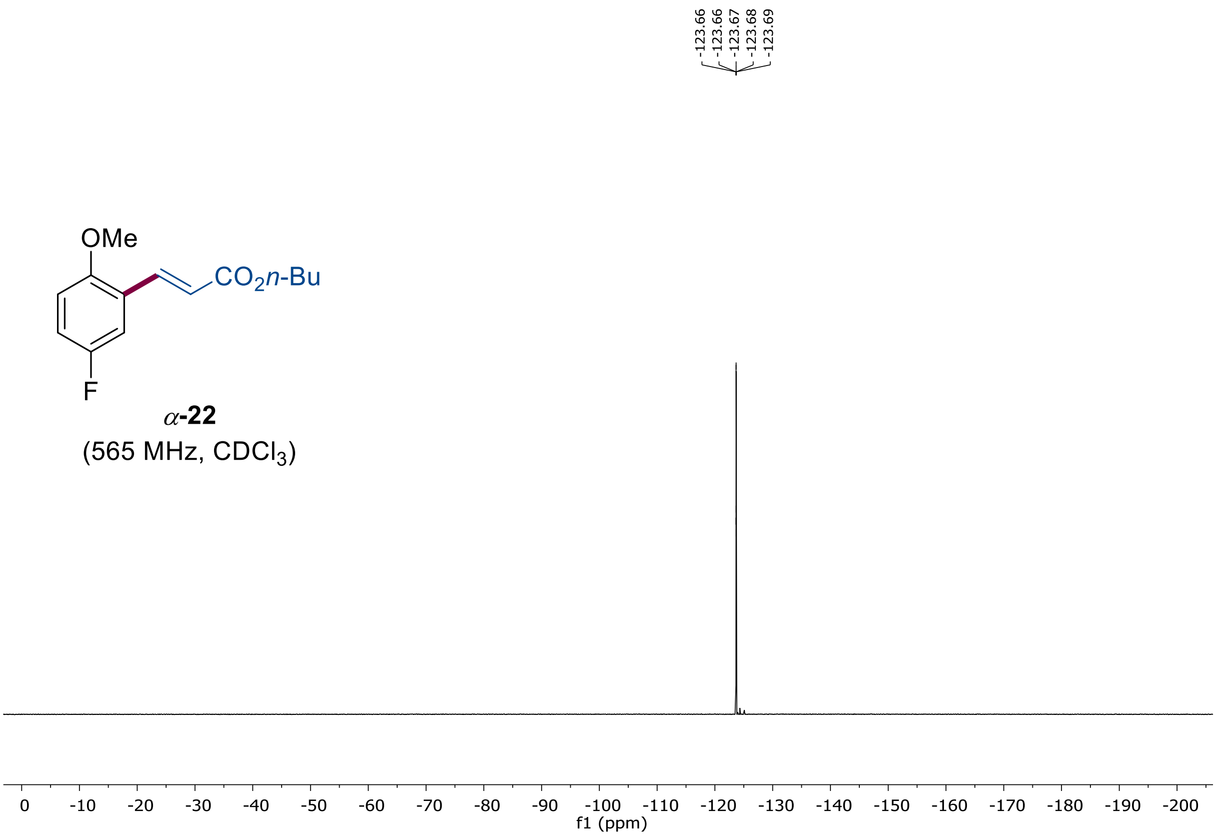

Supplementary Figure 106 F-NMR of compound *α*-22. 565 MHz, CDCl₃, RT

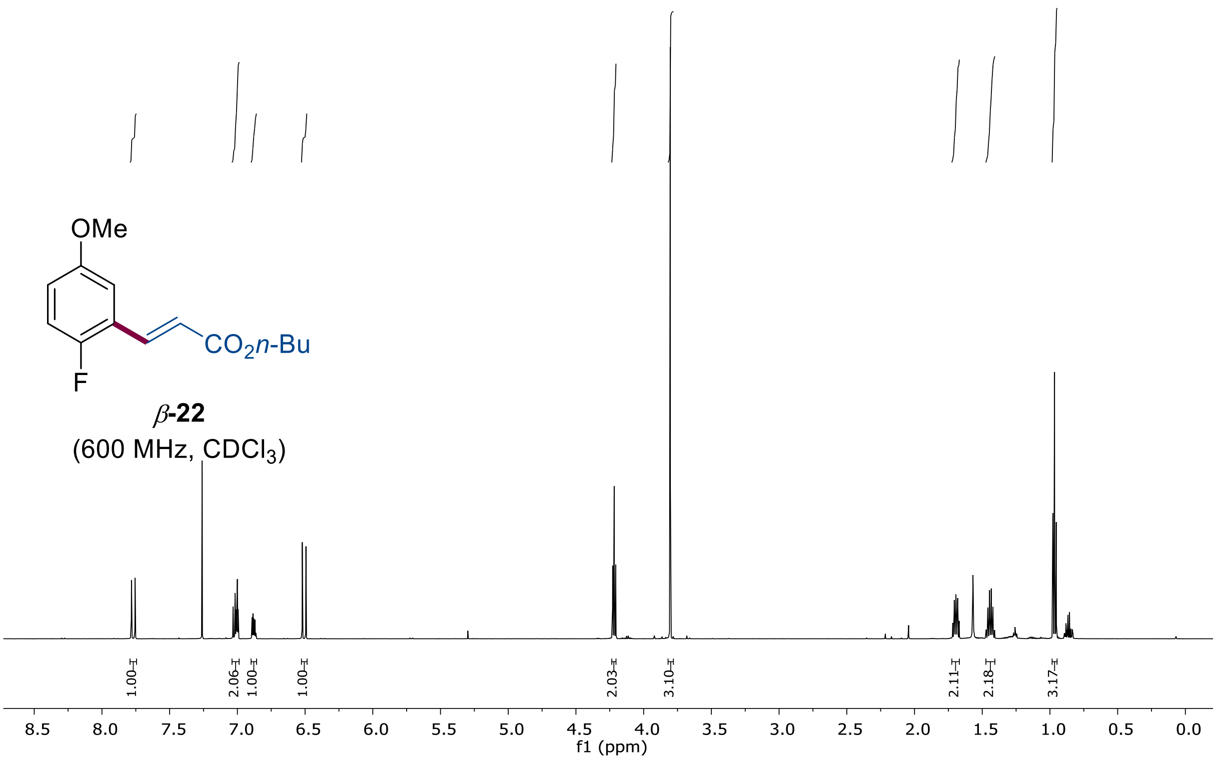

Supplementary Figure 107 H-NMR of compound *β*-22. 600 MHz, $CDCl_3$, RT

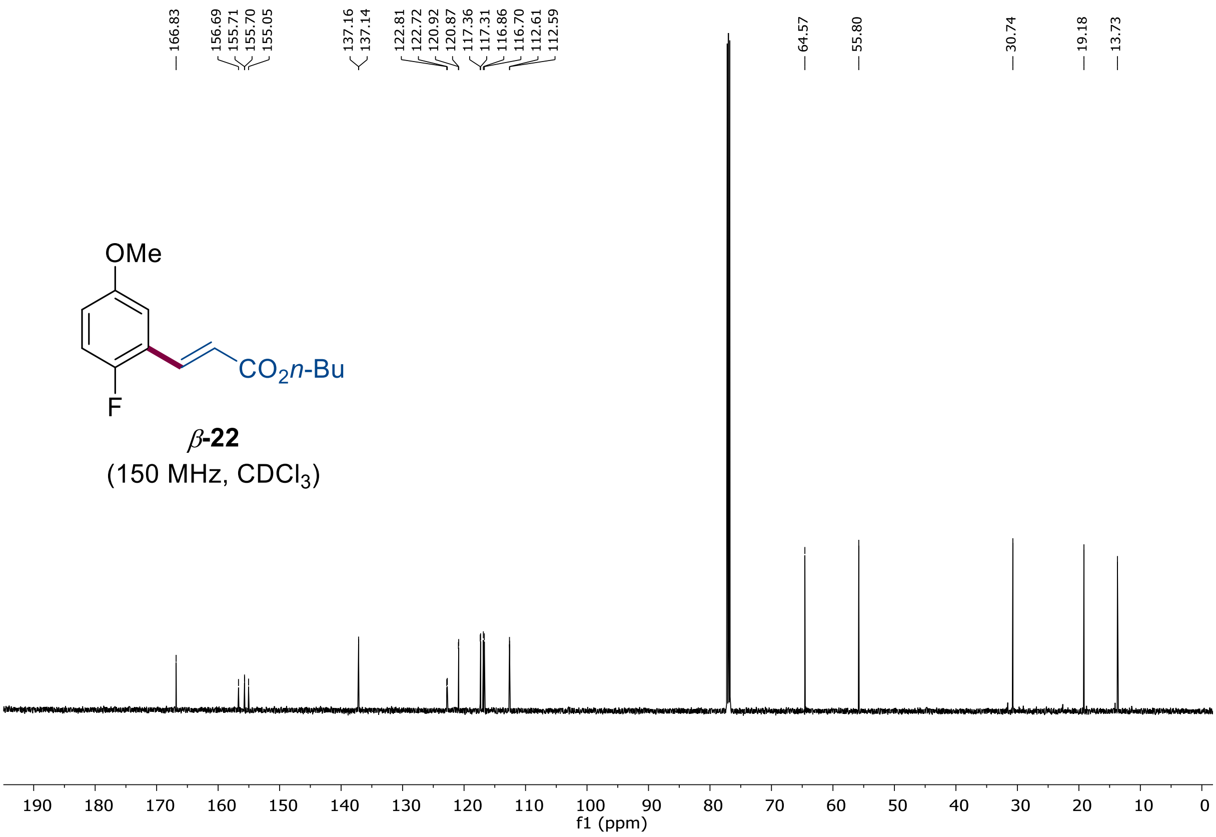

Supplementary Figure 108 C-NMR of compound β-22. 150 MHz, $CDCl_3$, RT

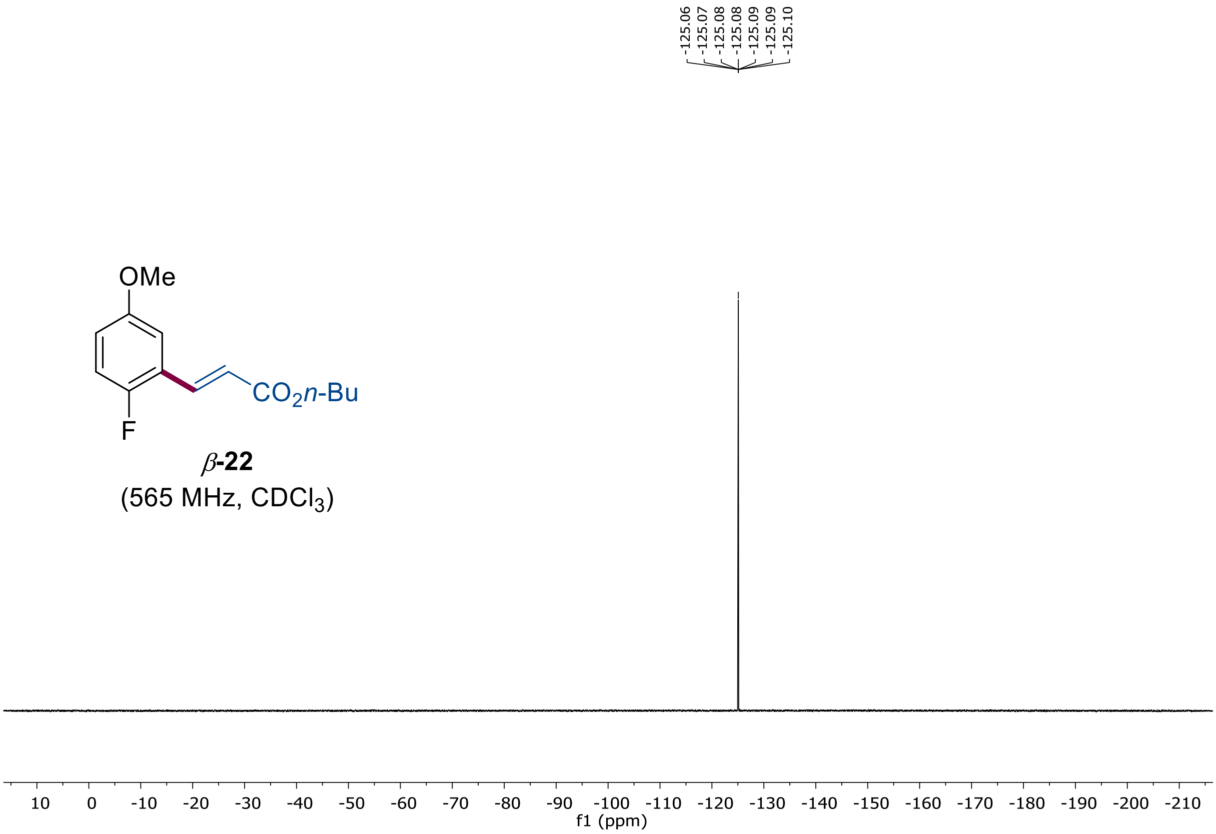

Supplementary Figure 109 F-NMR of compound β-22. 565 MHz, $CDCl_3$, RT

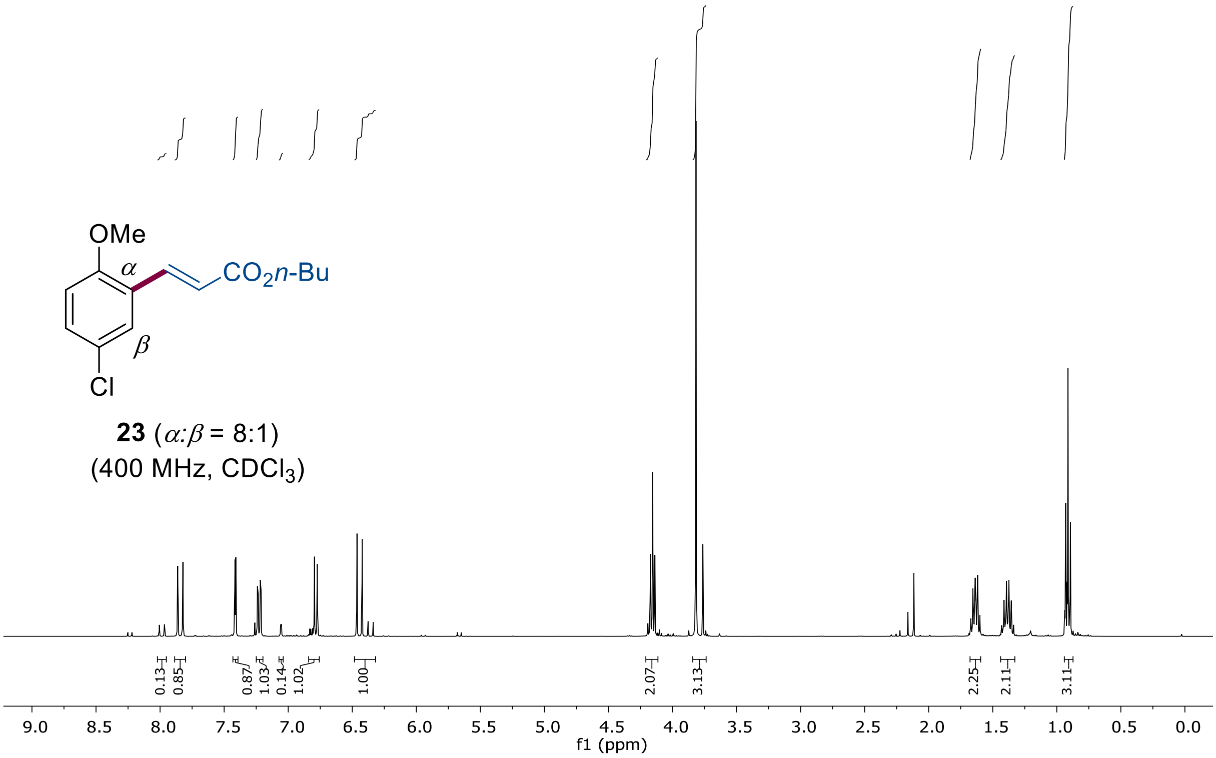

Supplementary Figure 110 H-NMR of compound 23. 400 MHz, CDCl₃, RT

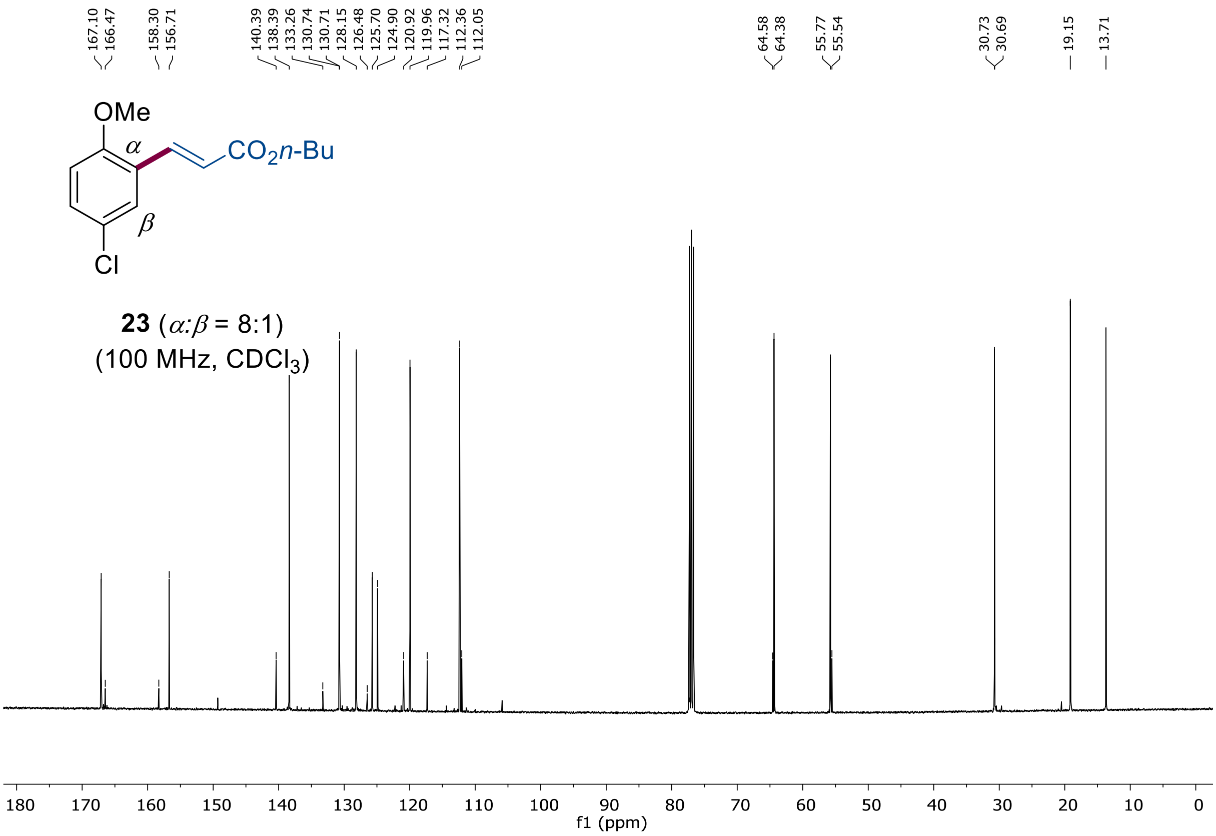

Supplementary Figure 111 C-NMR of compound 23. 100 MHz, CDCl₃, RT

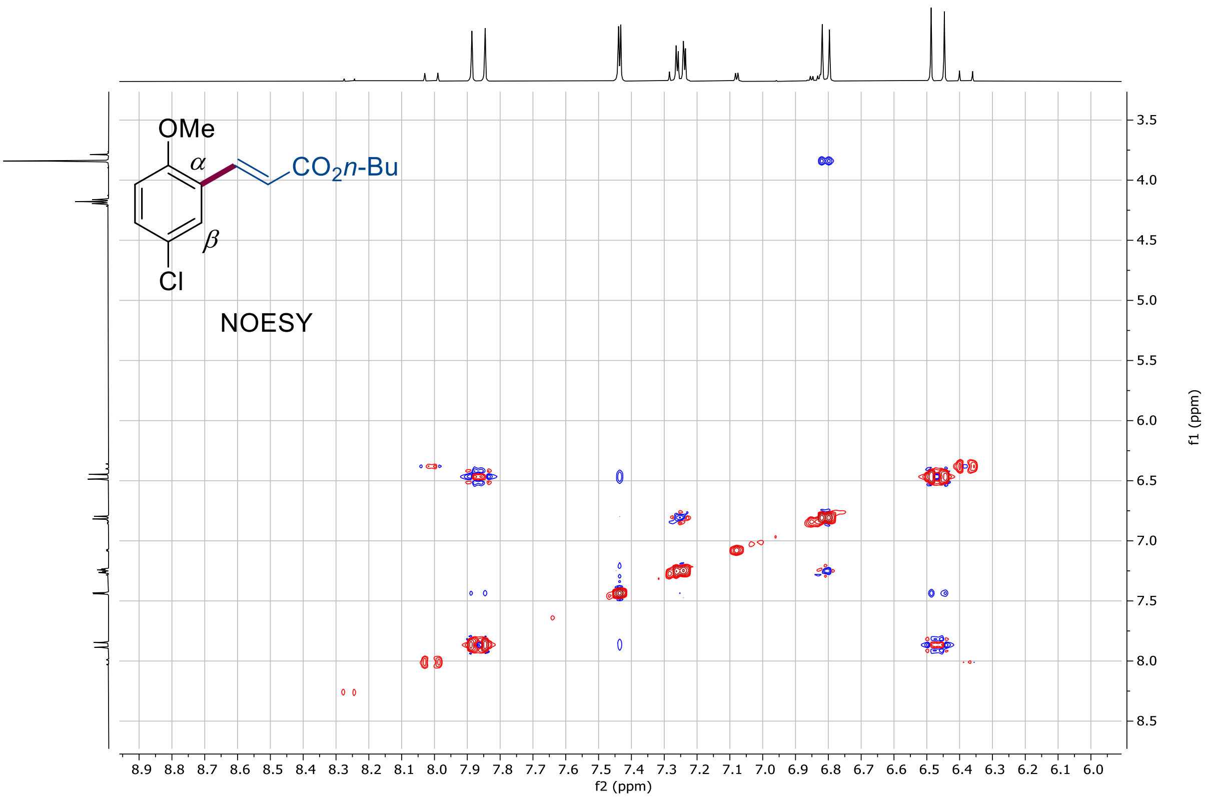

Supplementary Figure 112 NOESY-NMR of compound 23. CDCl3, RT

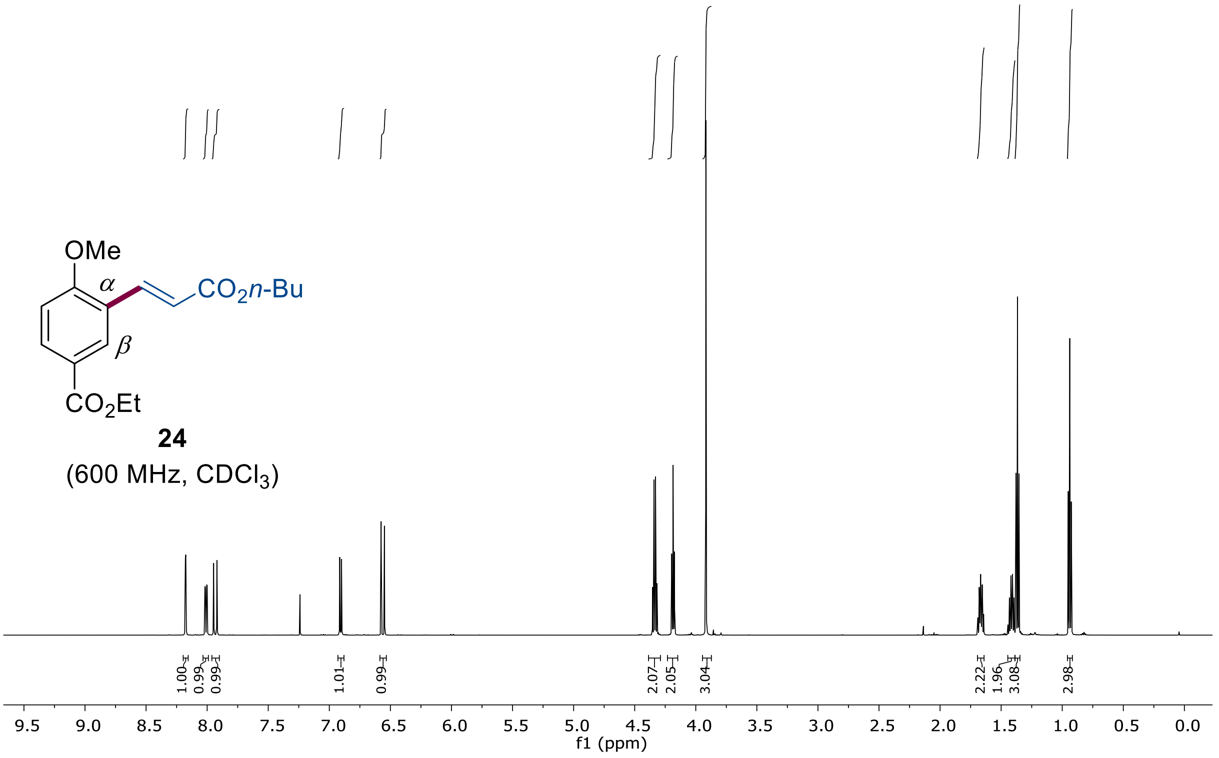

Supplementary Figure 113 H-NMR of compound 24. 600 MHz, CDCl3, RT

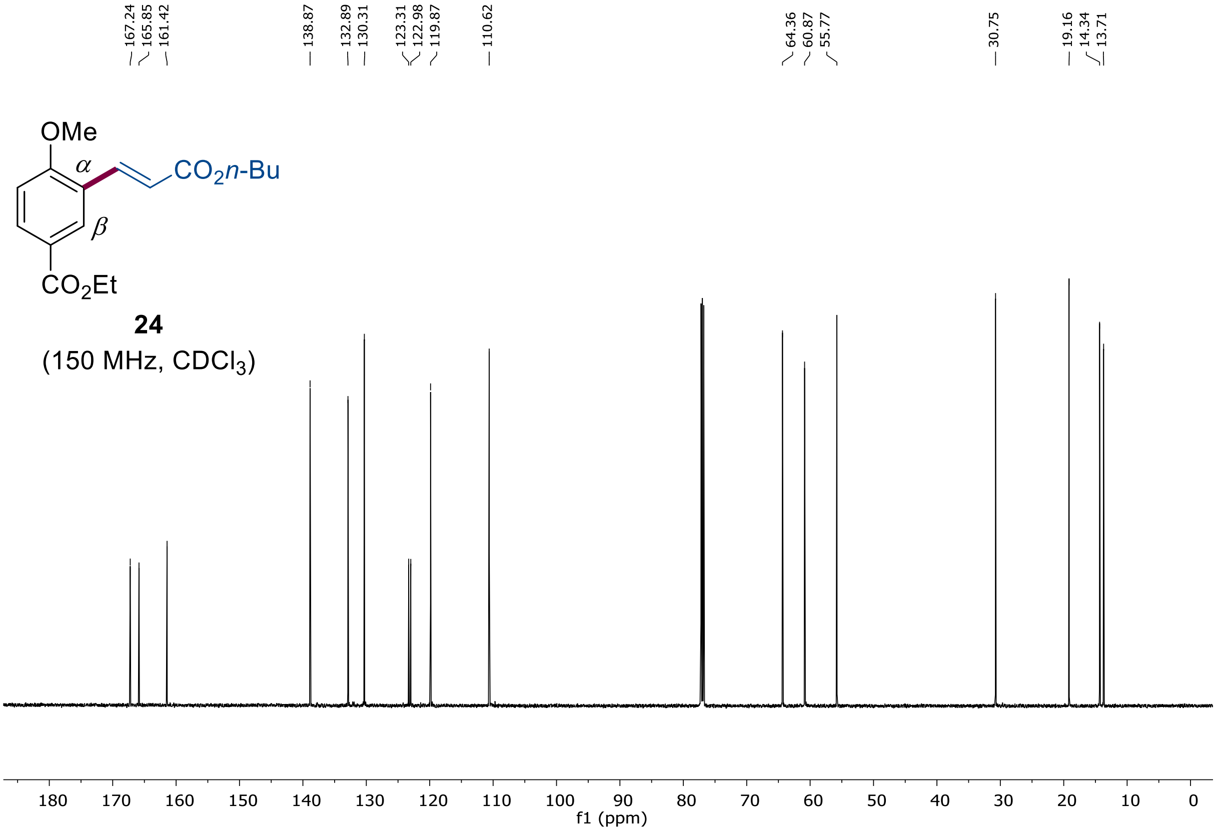

Supplementary Figure 114 H-NMR of compound 24. 150 MHz, $CDCl_3$, RT

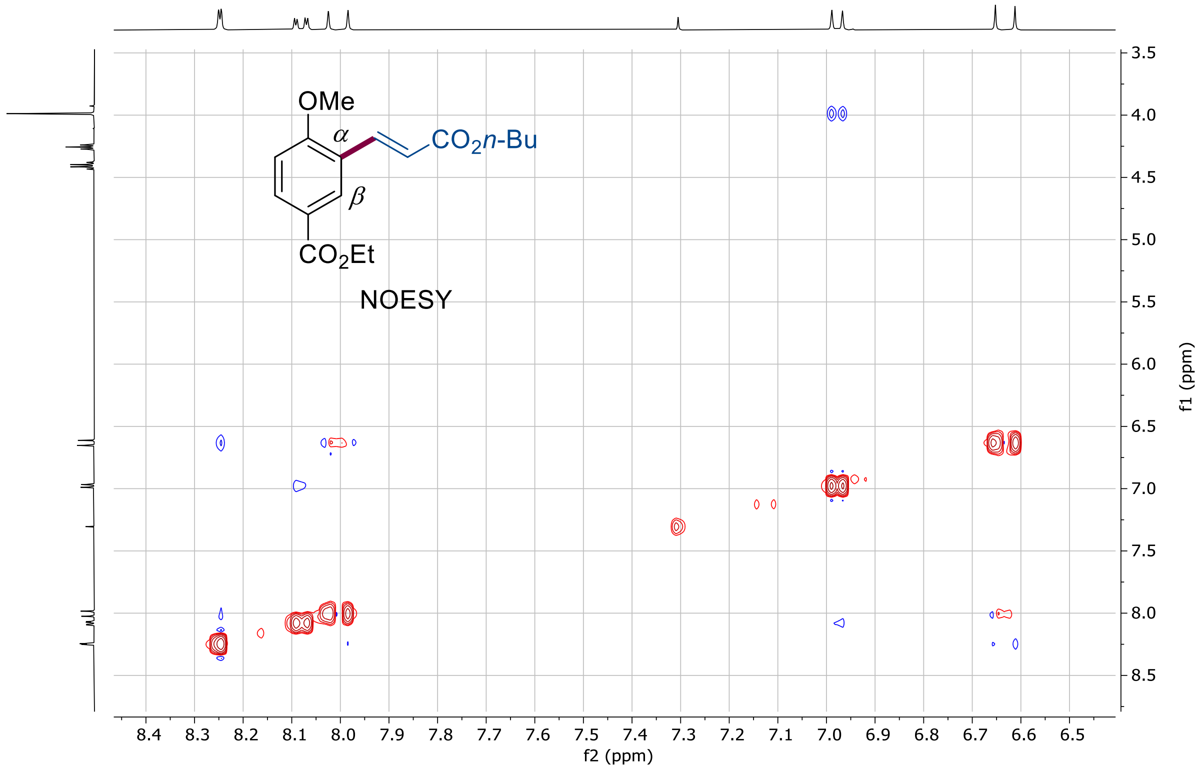

Supplementary Figure 115 NOESY-NMR of compound 24. $CDCl_3$, RT

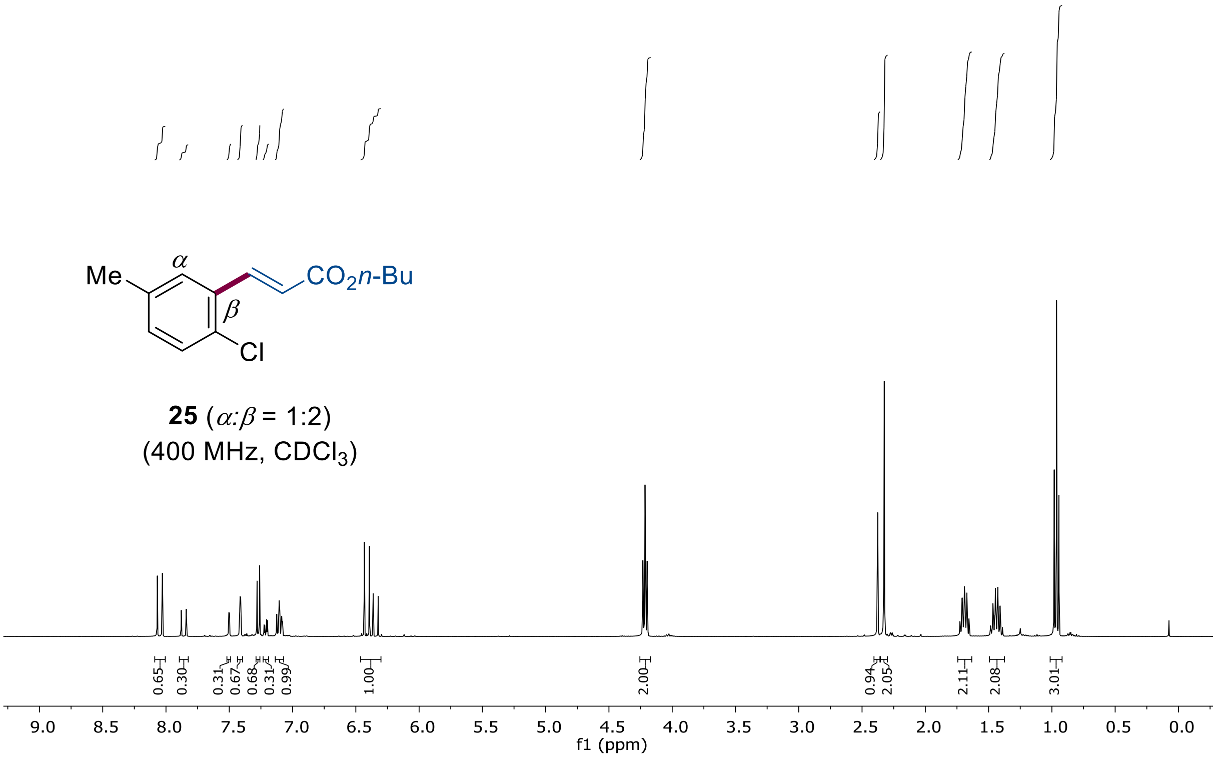

Supplementary Figure 116 H-NMR of compound 25. 400 MHz, $CDCl_3$, RT

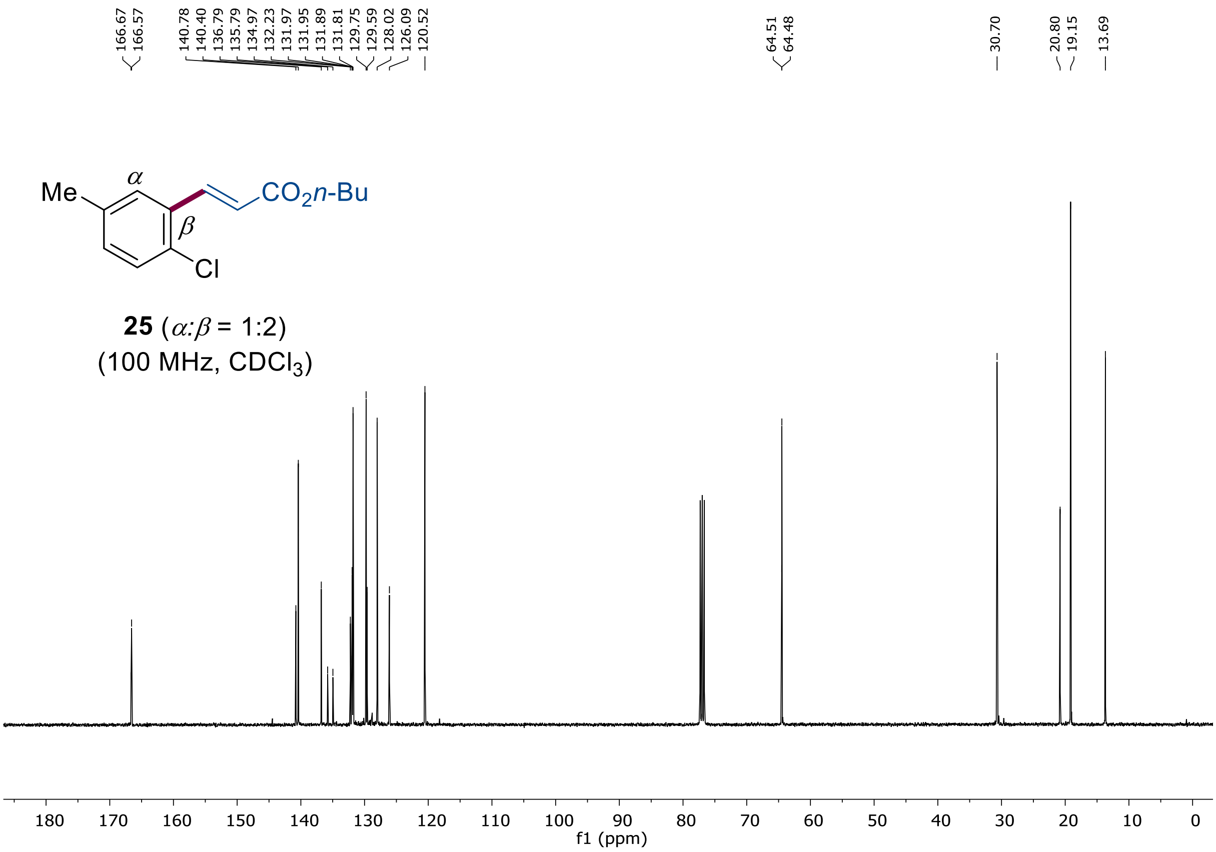

Supplementary Figure 117 C-NMR of compound 25. 100 MHz, $CDCl_3$, RT

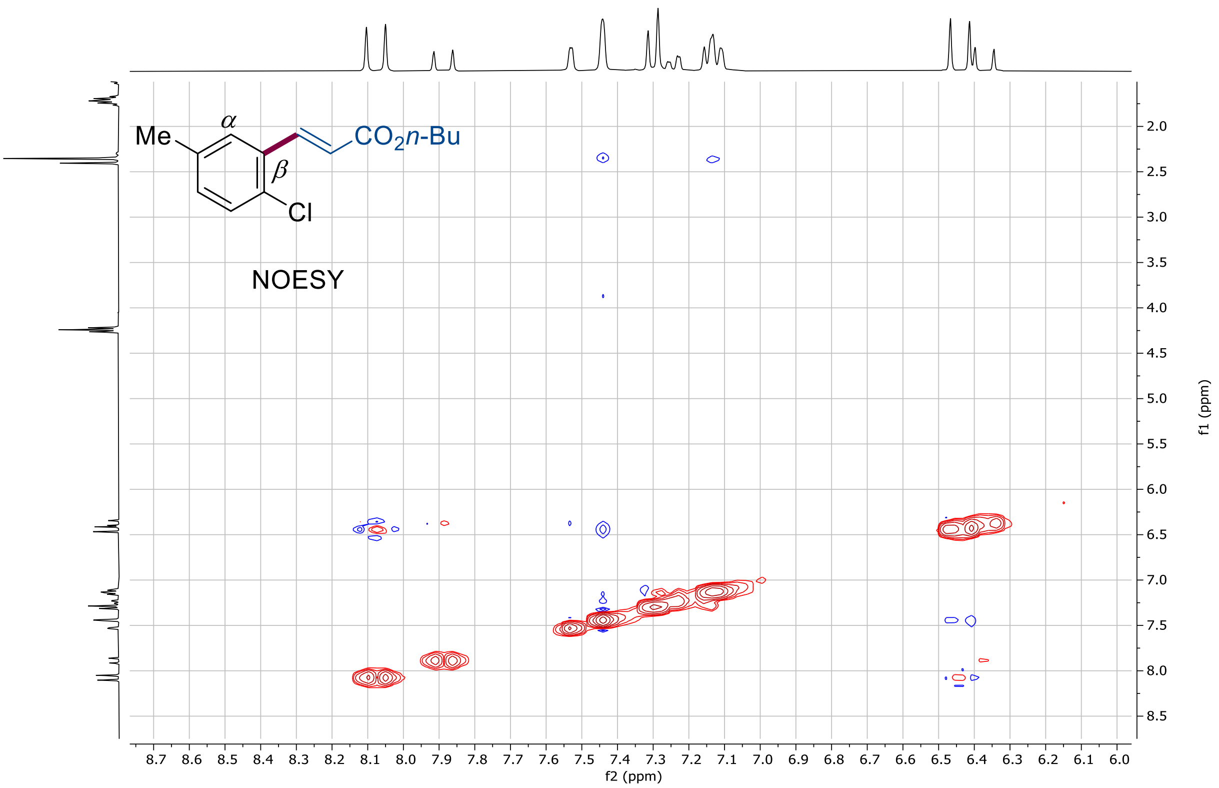

Supplementary Figure 118 NOESY-NMR of compound 25. 100 MHz, CDCl₃, RT

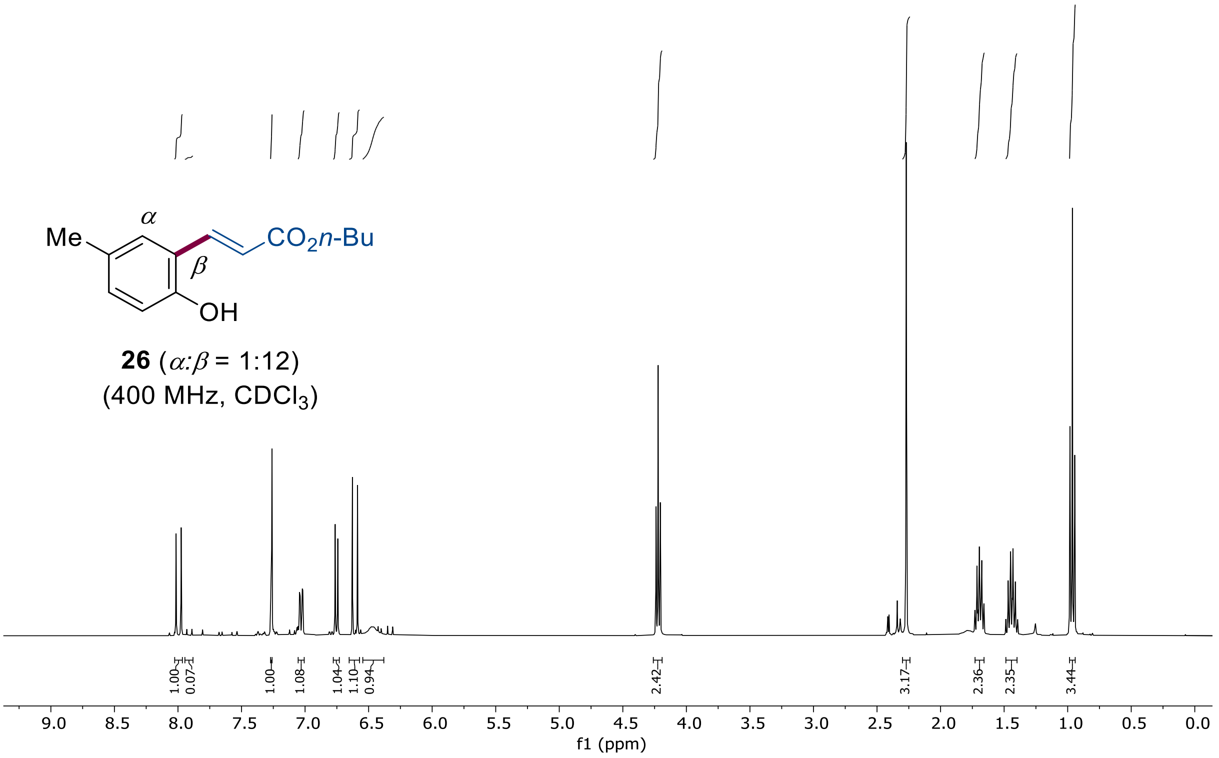

Supplementary Figure 119 H-NMR of compound 26. 400 MHz, CDCl₃, RT

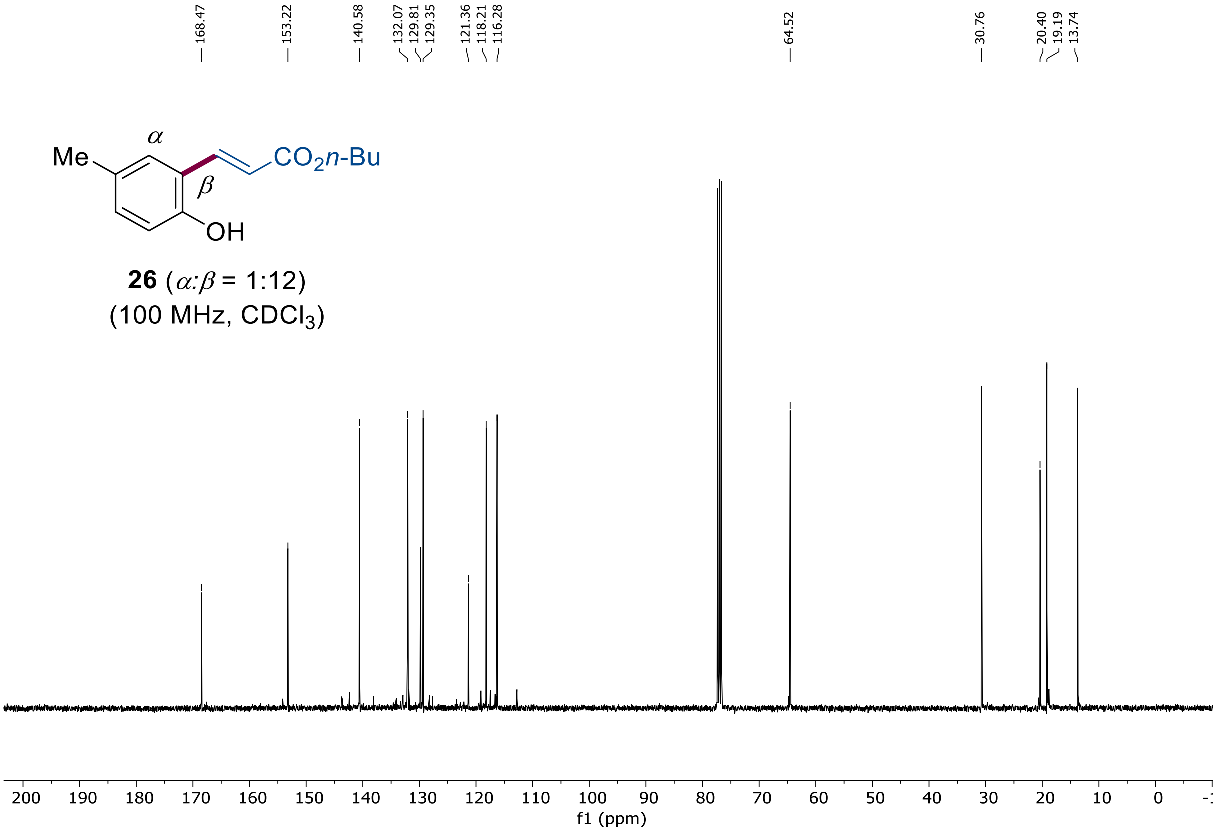

Supplementary Figure 120 C-NMR of compound 26. 100 MHz, $CDCl_3$, RT

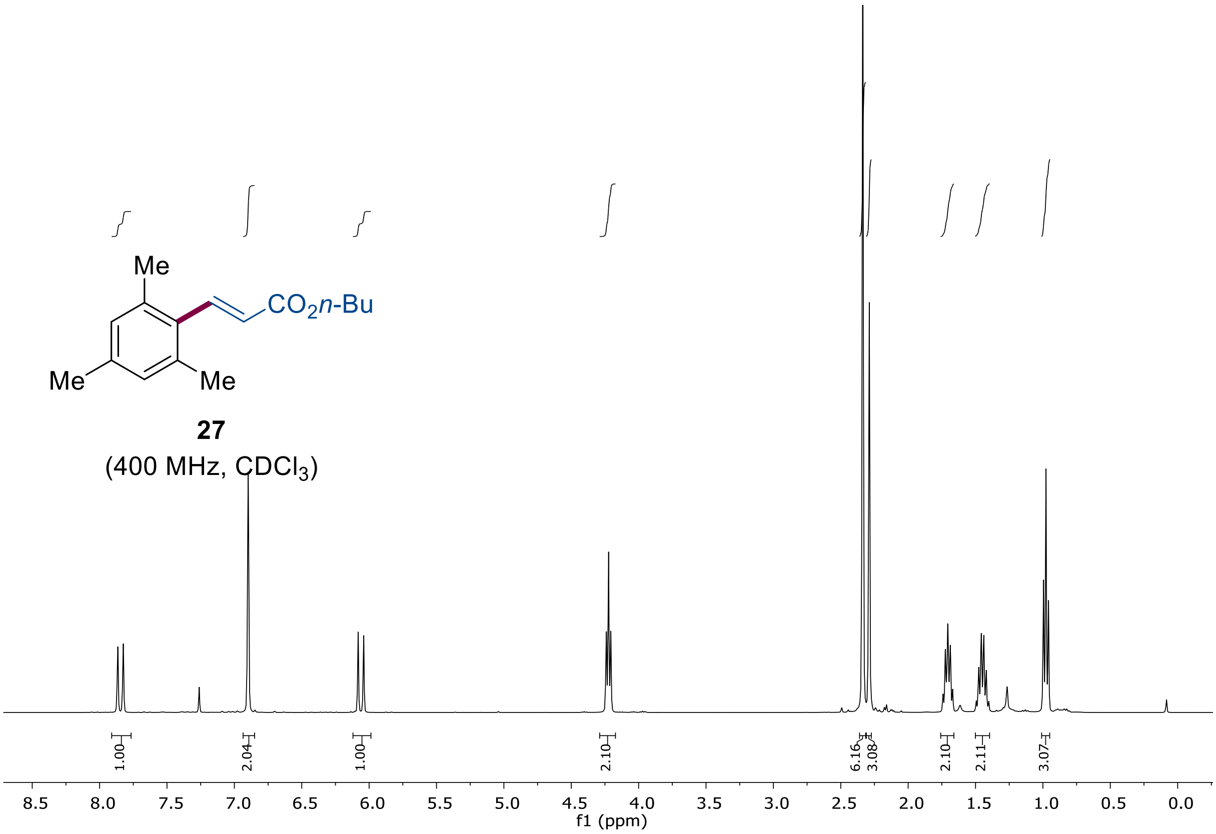

Supplementary Figure 121 H-NMR of compound 27. 400 MHz, $CDCl_3$, RT

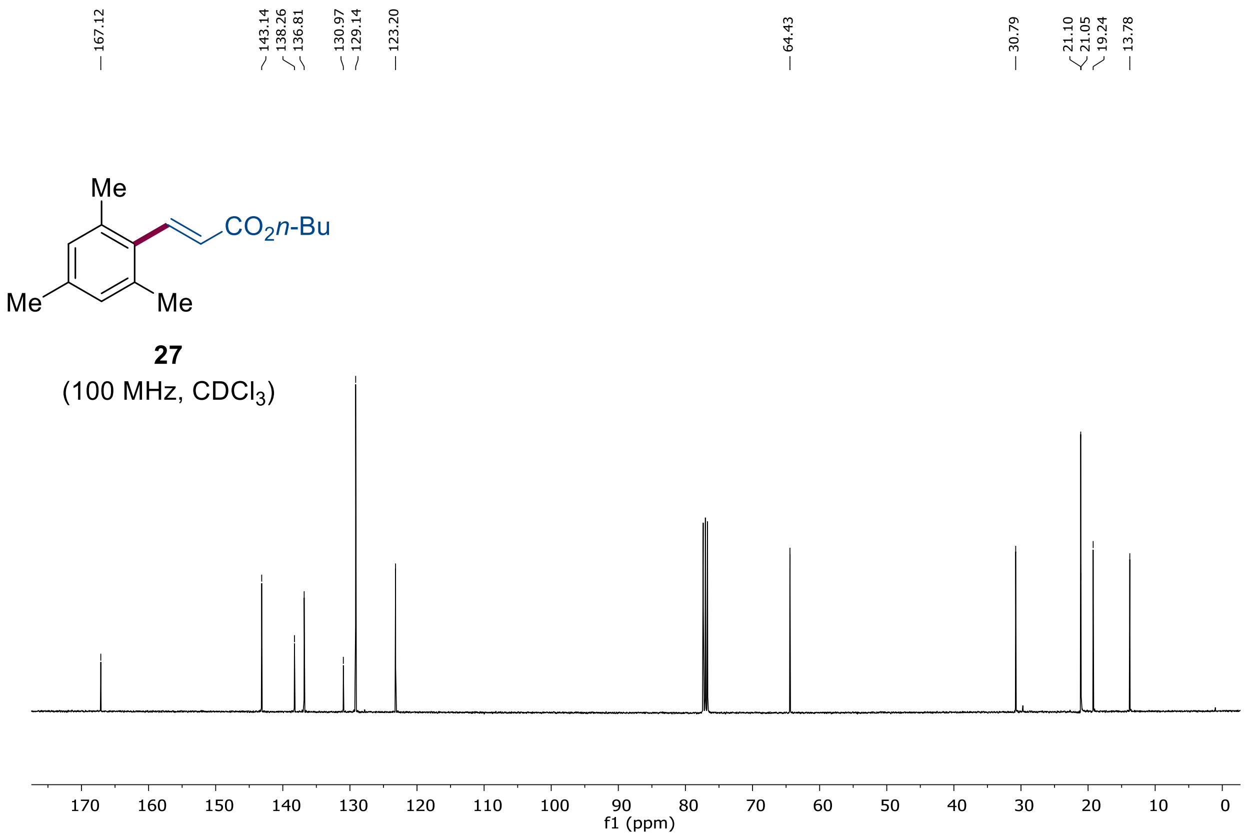

Supplementary Figure 122 C-NMR of compound 27. 100 MHz, $CDCl_3$, RT

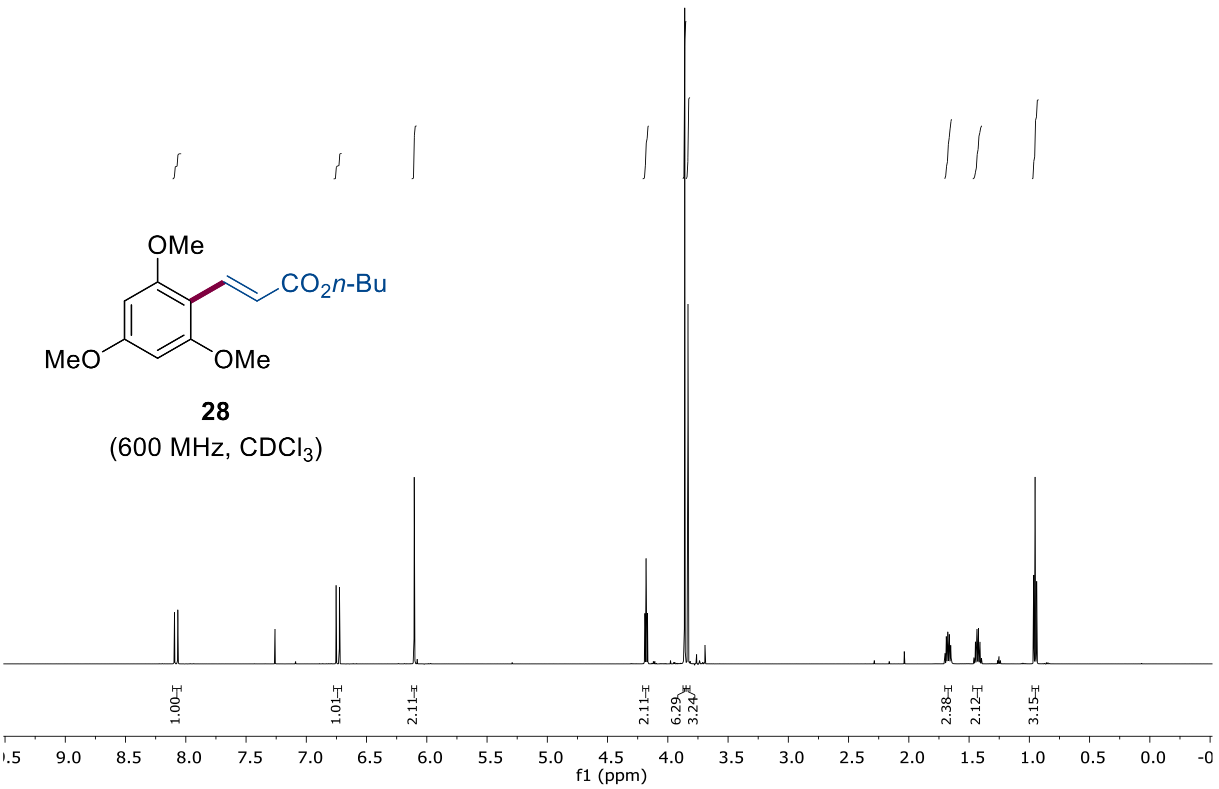

Supplementary Figure 123 H-NMR of compound 28. 600 MHz, $CDCl_3$, RT

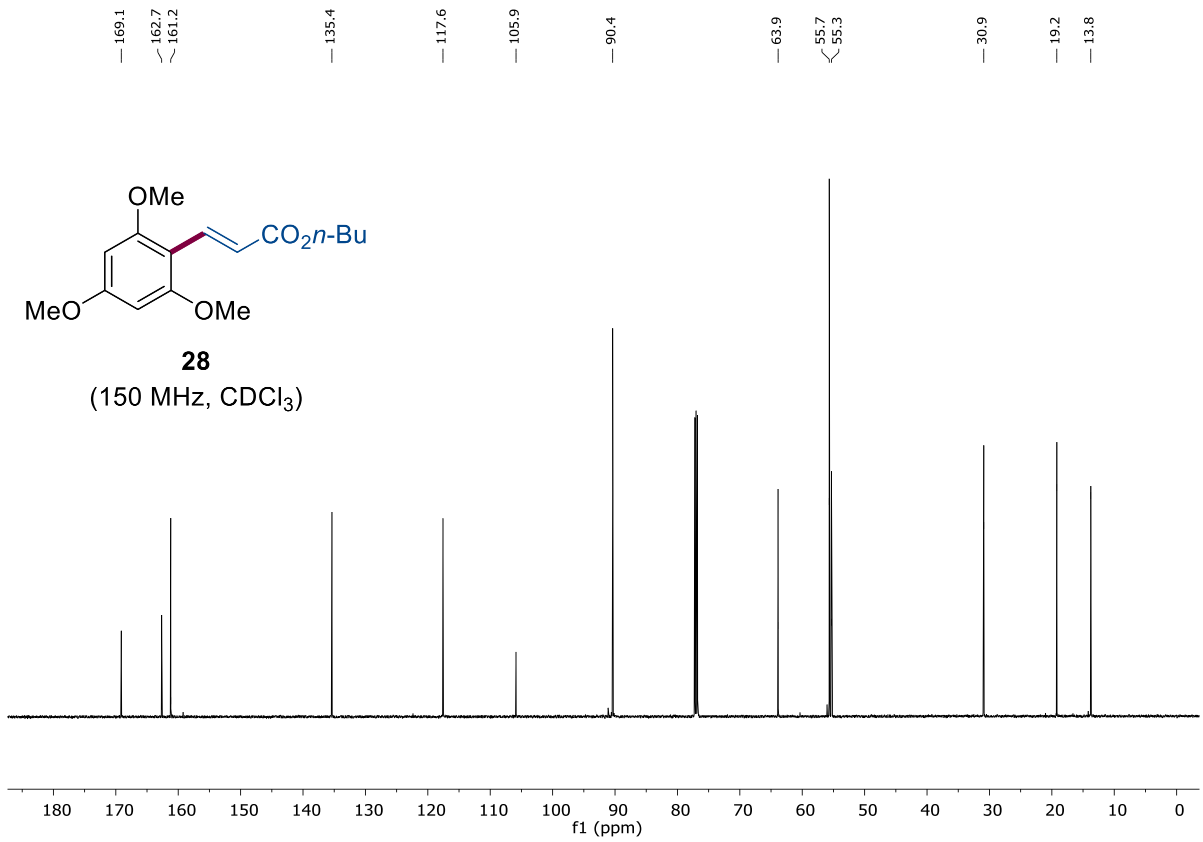

Supplementary Figure 124 C-NMR of compound 28. 150 MHz, $CDCl_3$, RT

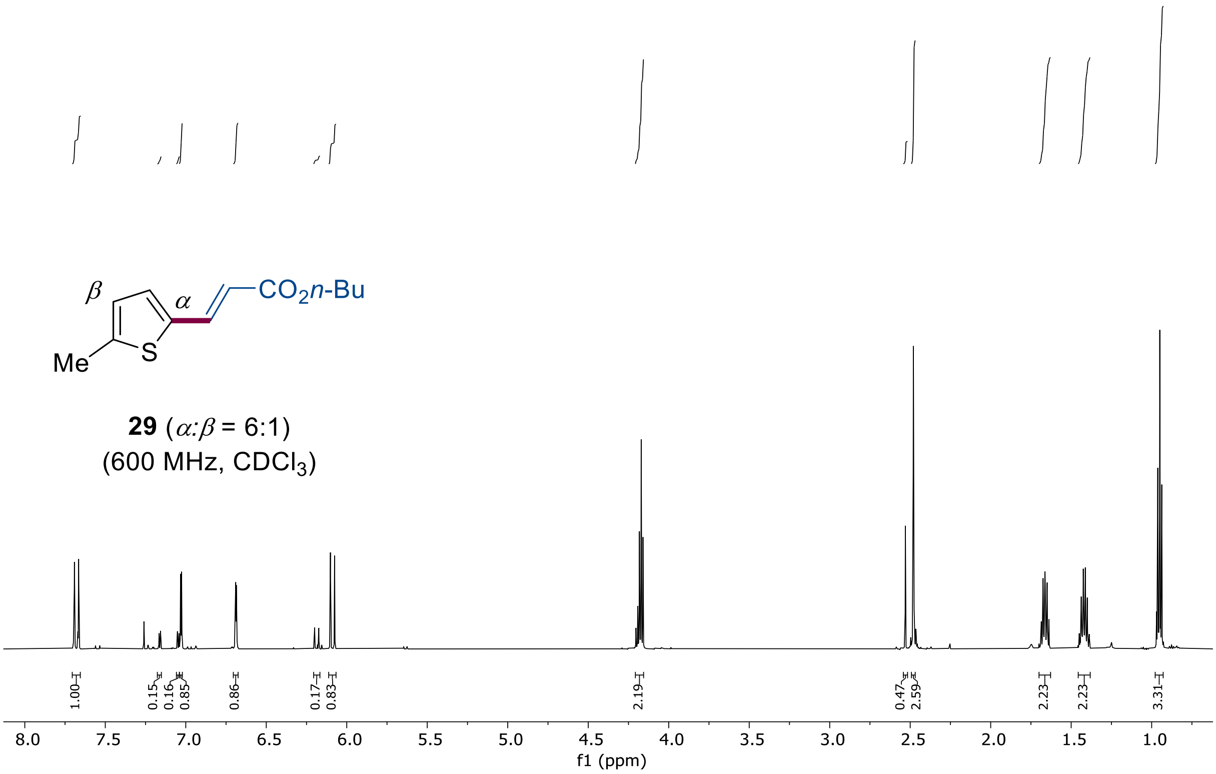

Supplementary Figure 125 H-NMR of compound 29. 600 MHz, $CDCl_3$, RT

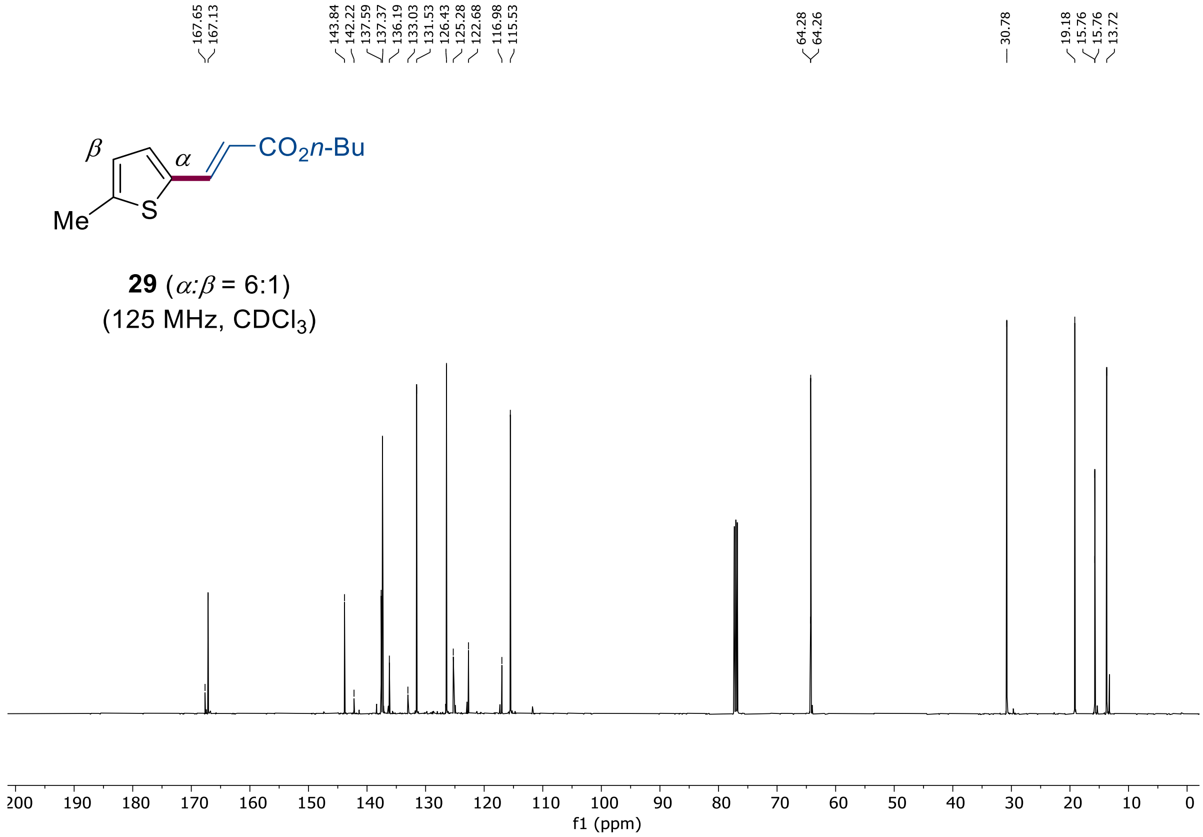

Supplementary Figure 126 C-NMR of compound 29. 125 MHz, CDCl$_3$, RT

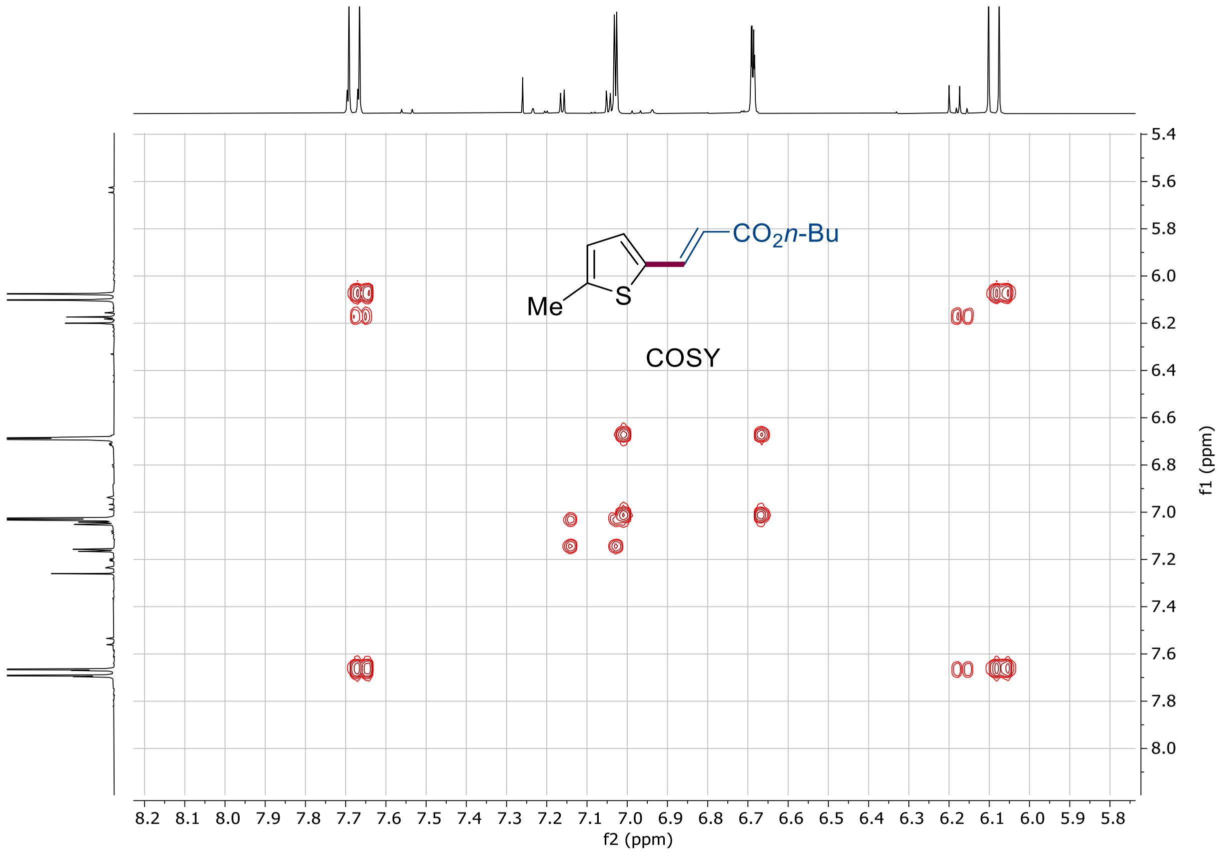

Supplementary Figure 127 COSY-NMR of compound 29. CDCl$_3$, RT

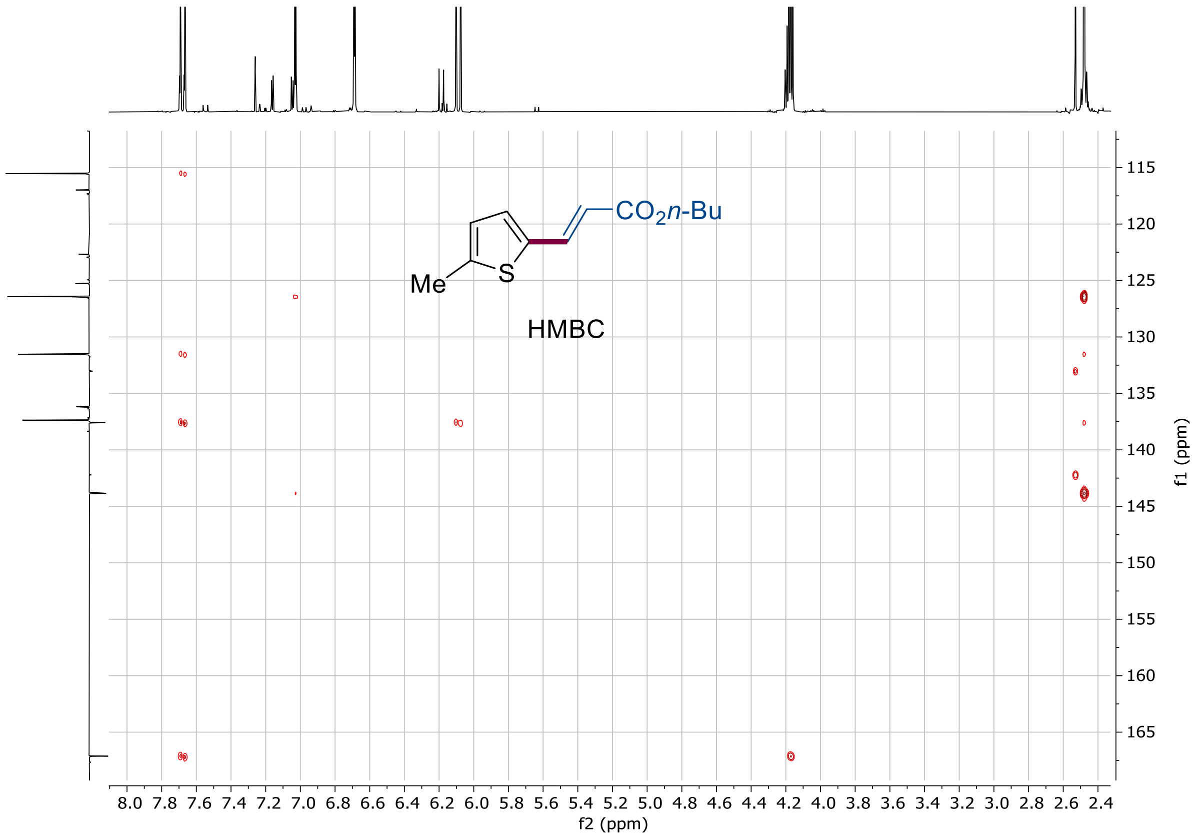

Supplementary Figure 128 **HMBC-NMR of compound 29. $CDCl_3$, RT**

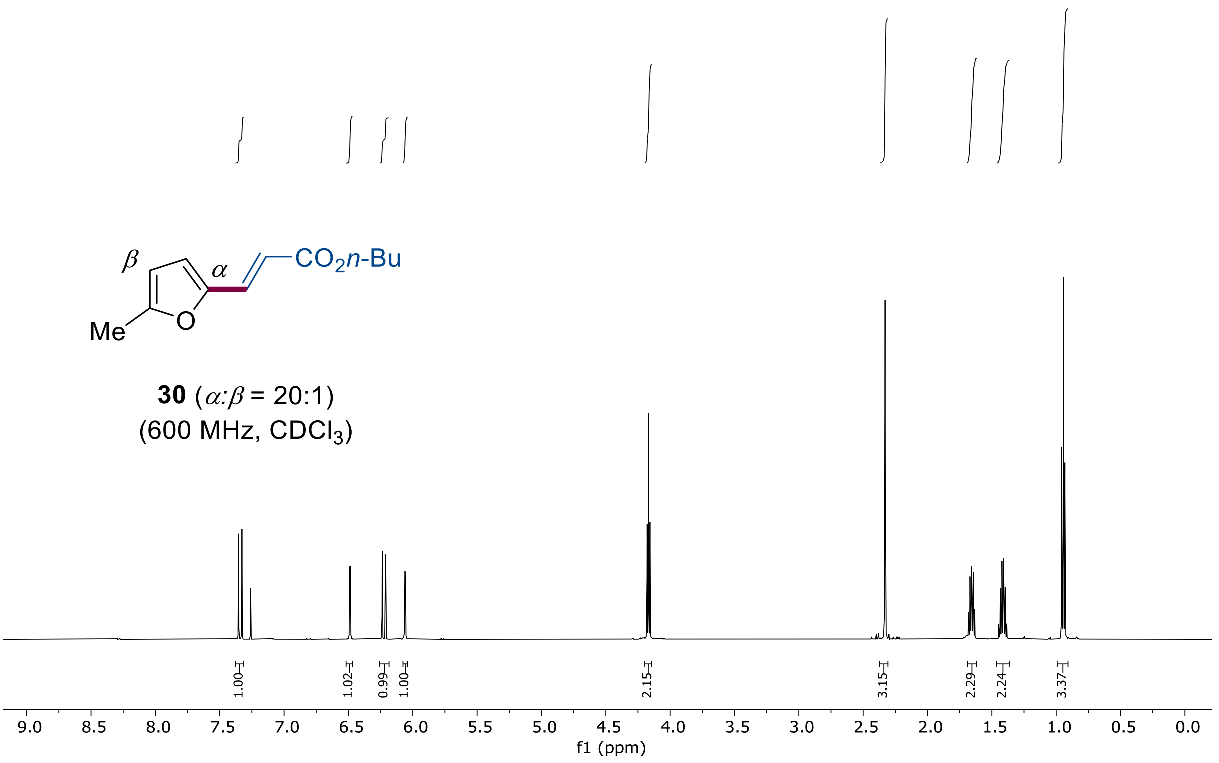

Supplementary Figure 129 **H-NMR of compound 30. 600 MHz, $CDCl_3$, RT**

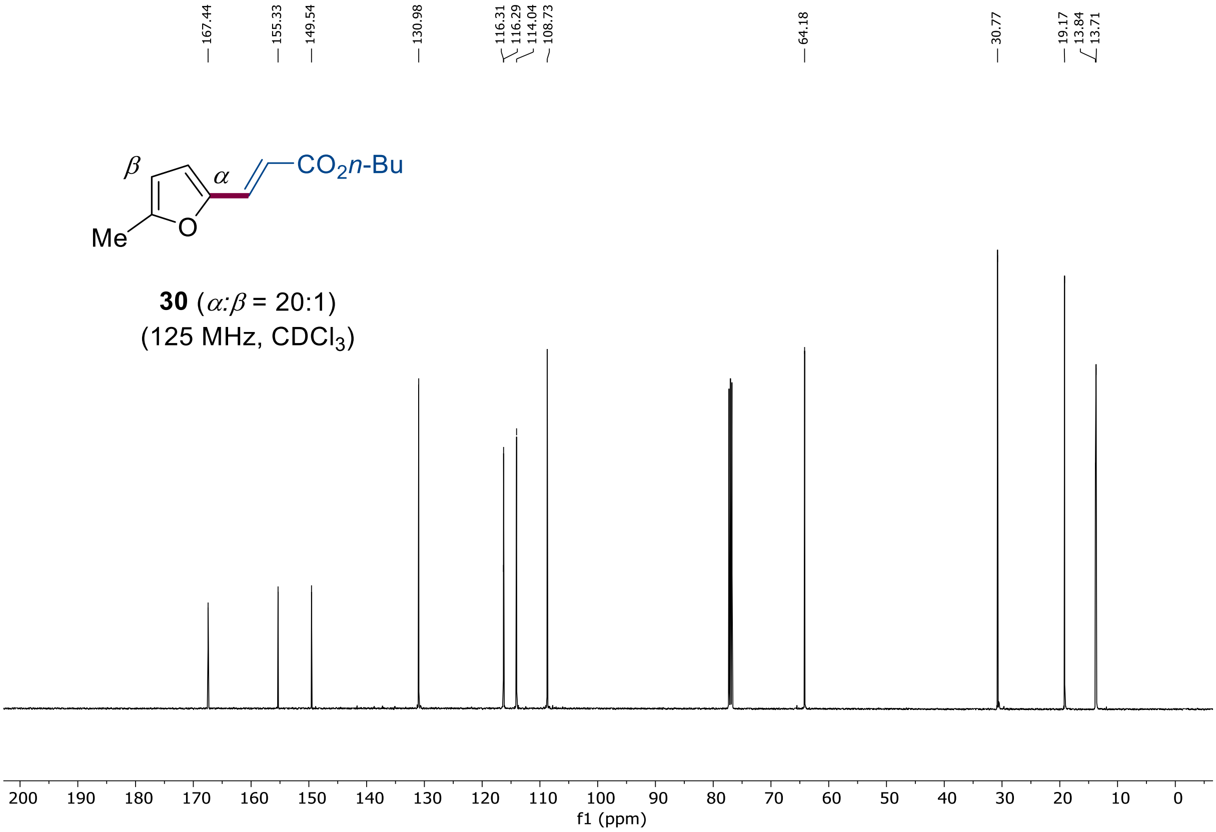

Supplementary Figure 130 C-NMR of compound 30. 125 MHz, $CDCl_3$, RT

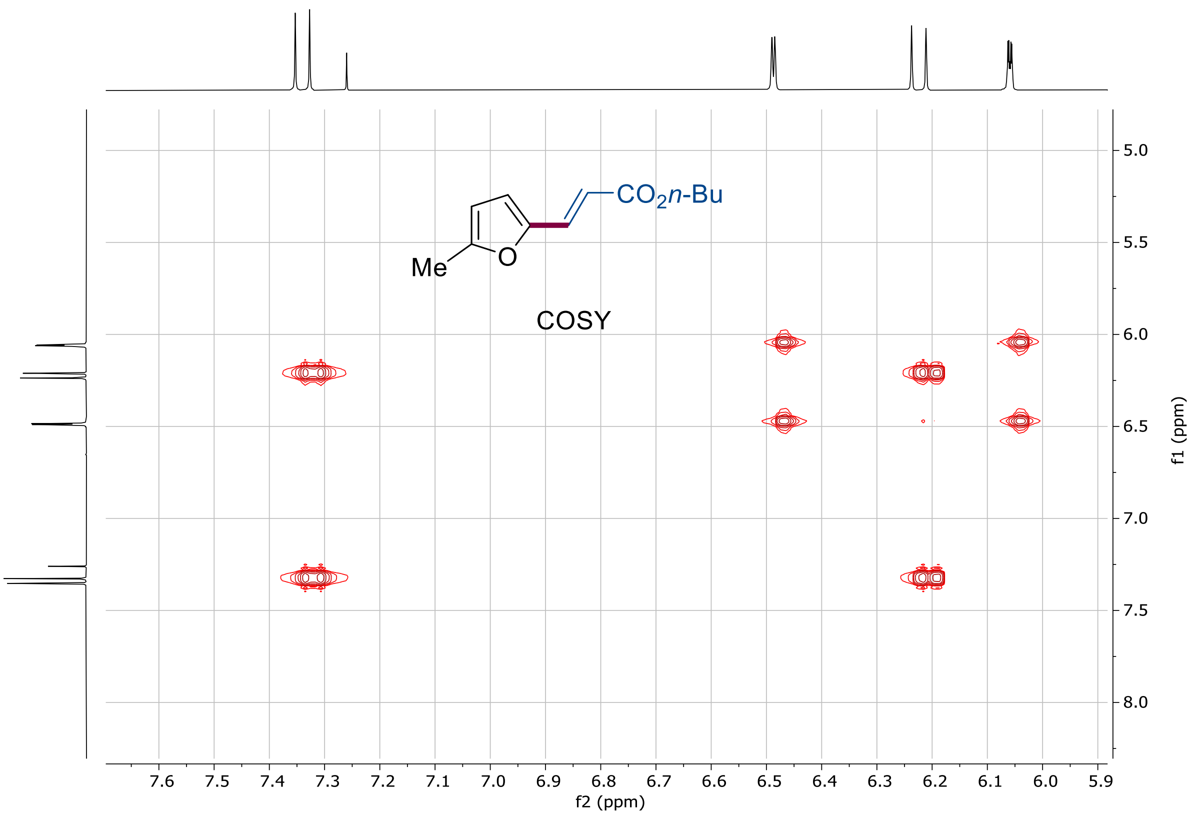

Supplementary Figure 131 COSY-NMR of compound 30. $CDCl_3$, RT

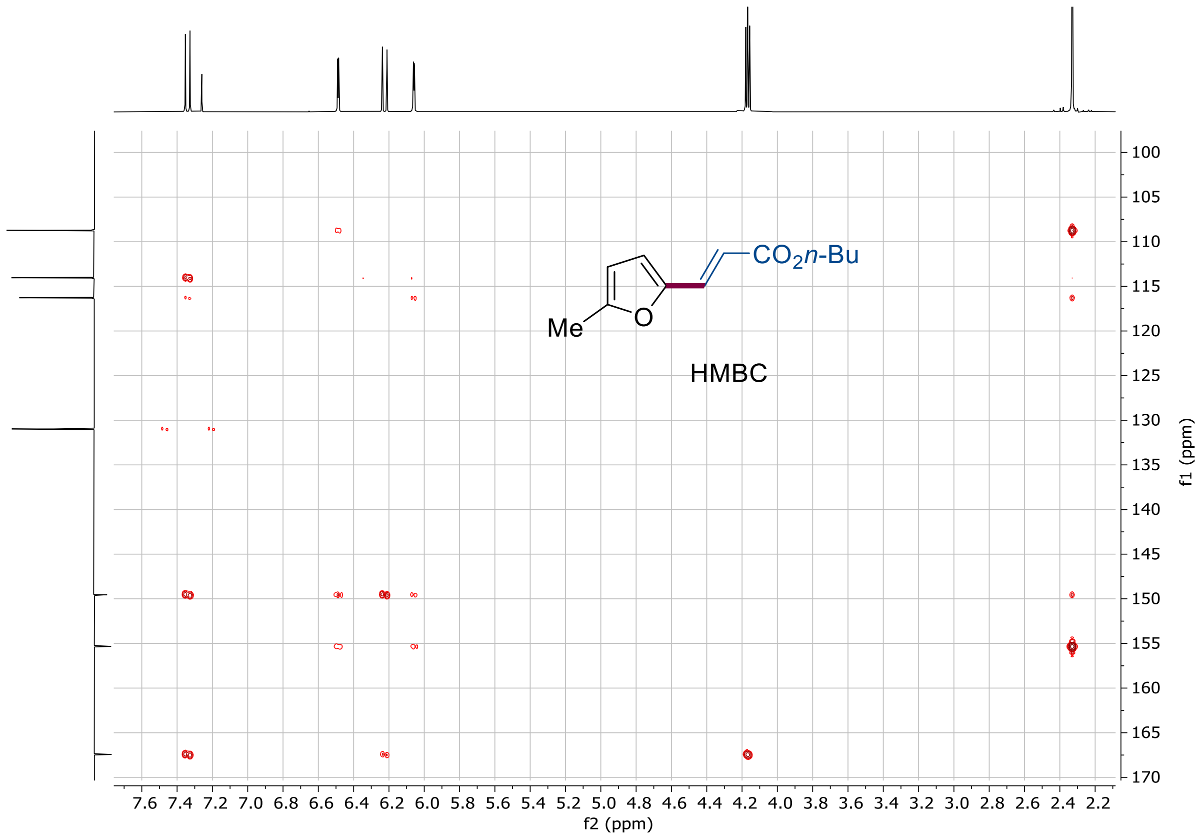

Supplementary Figure 132 HMBC-NMR of compound 30. CDCl3, RT

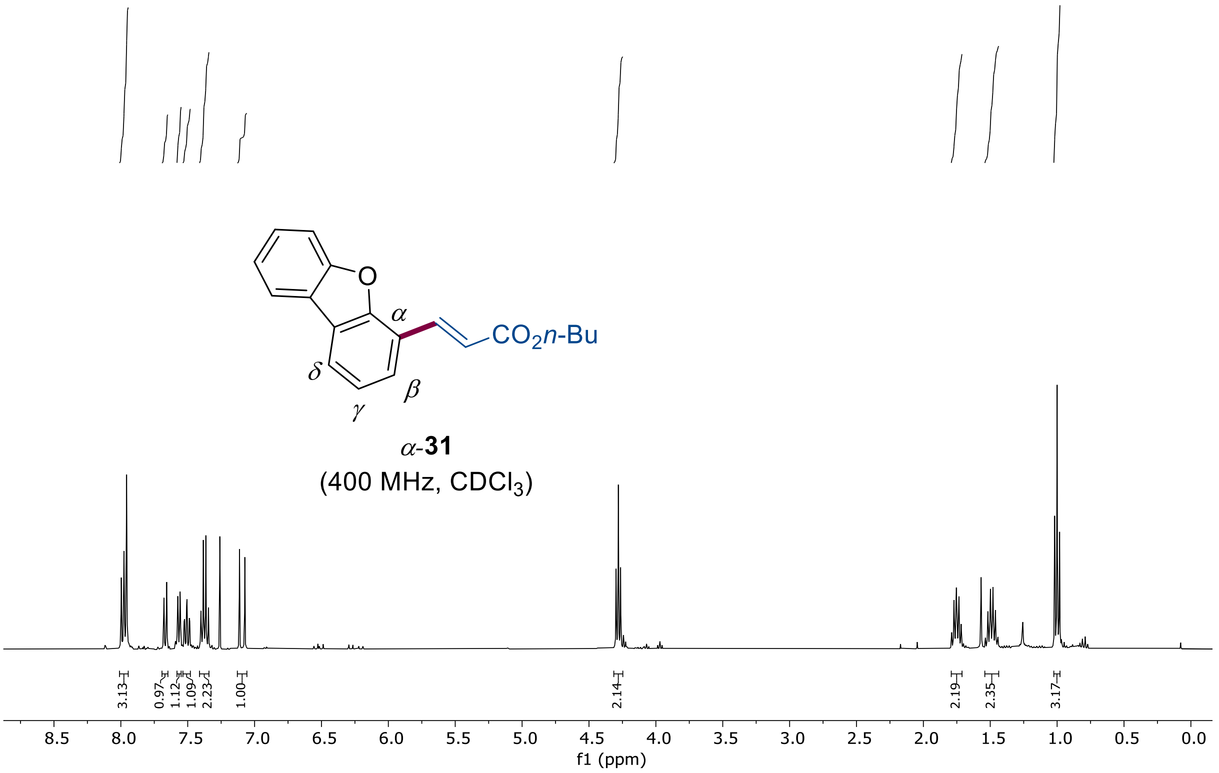

Supplementary Figure 133 H-NMR of compound α-31. 400 MHz, CDCl3, RT

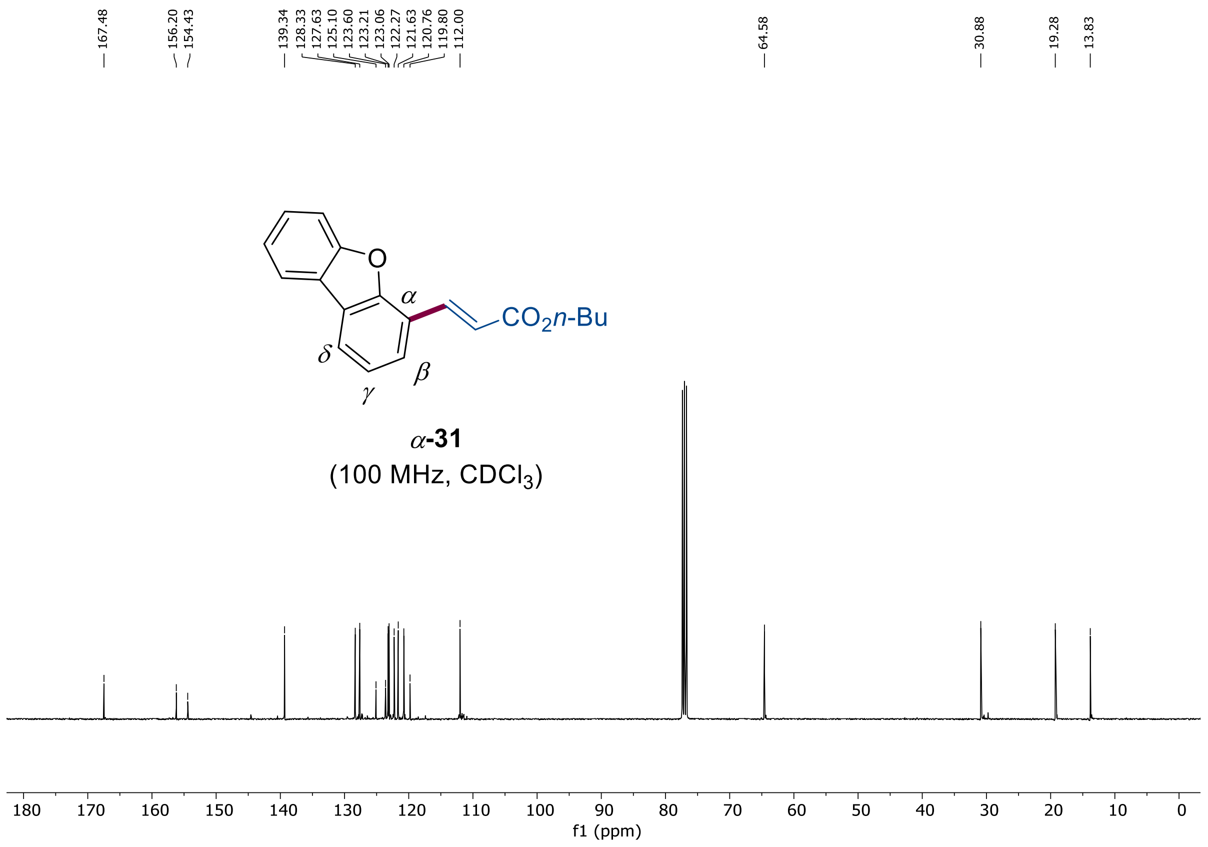

Supplementary Figure 134 C-NMR of compound α-31. 100 MHz, $CDCl_3$, RT

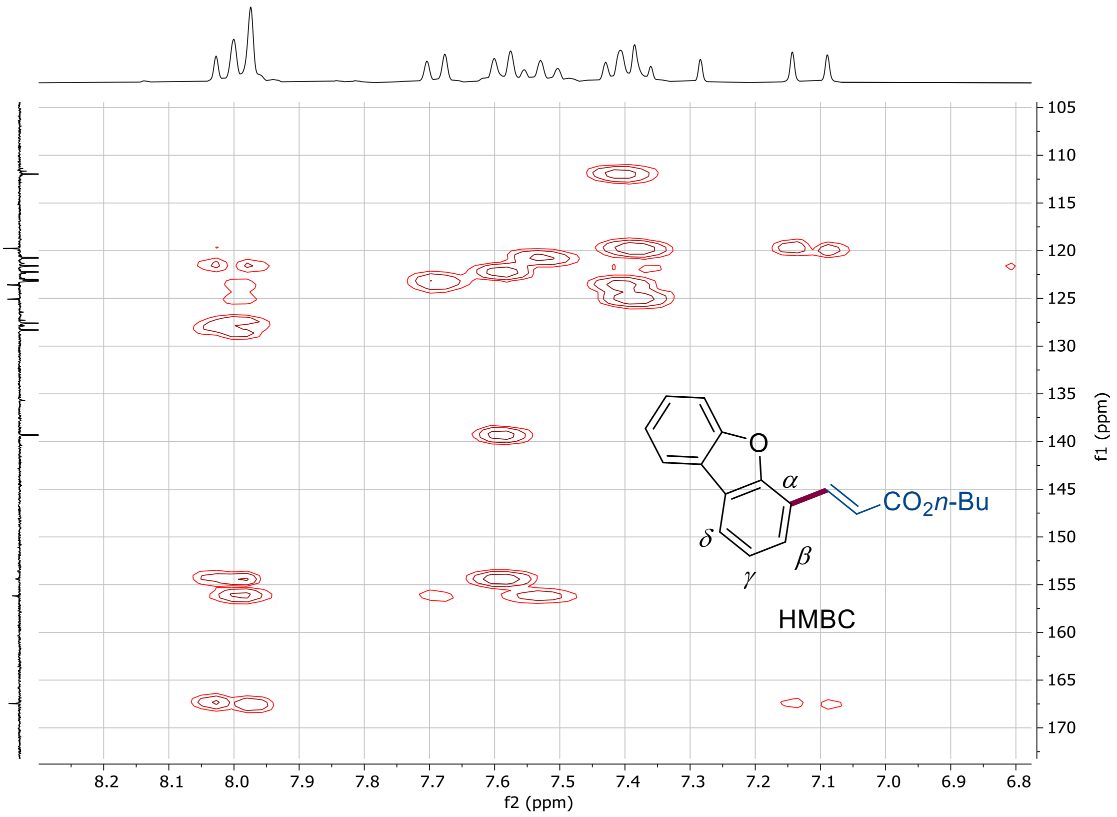

Supplementary Figure 135 HMBC-NMR of compound α-31. $CDCl_3$, RT

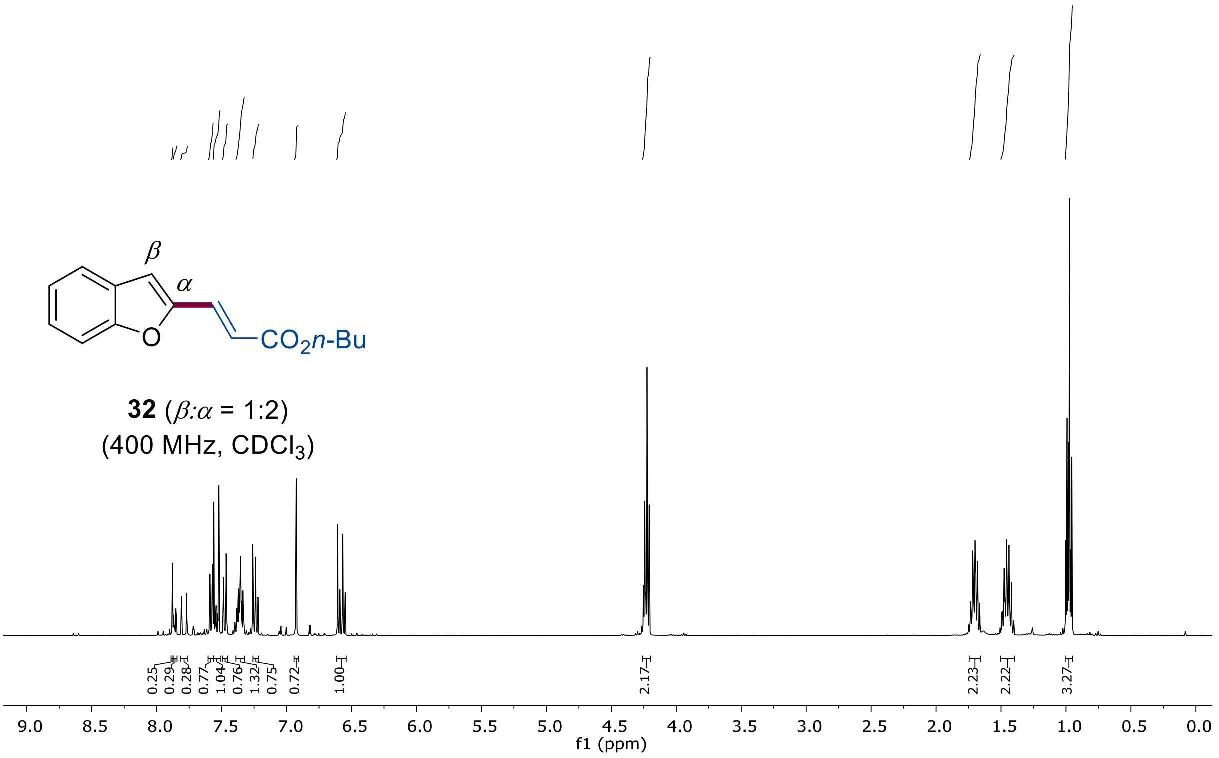

Supplementary Figure 136 H-NMR of compound 32. 400 MHz, $CDCl_3$, RT

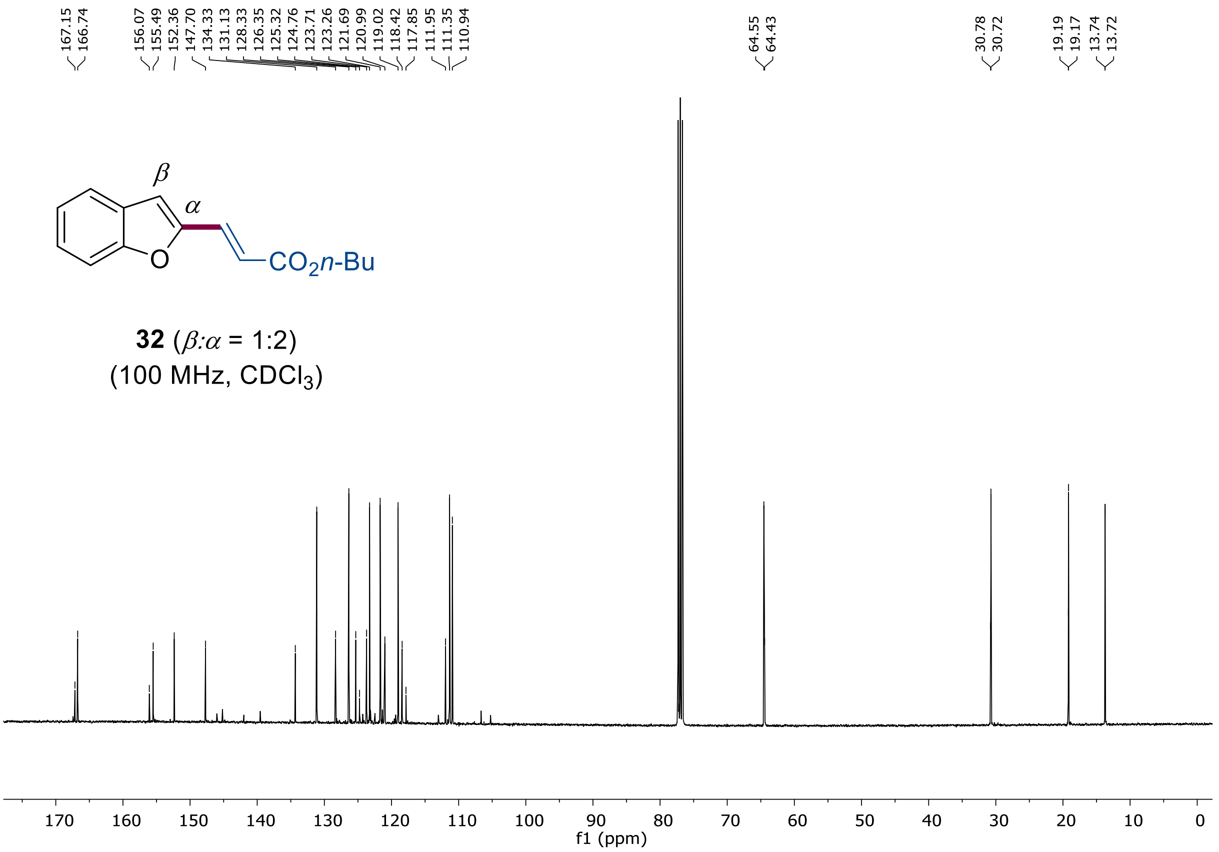

Supplementary Figure 137 C-NMR of compound 32. 100 MHz, $CDCl_3$, RT

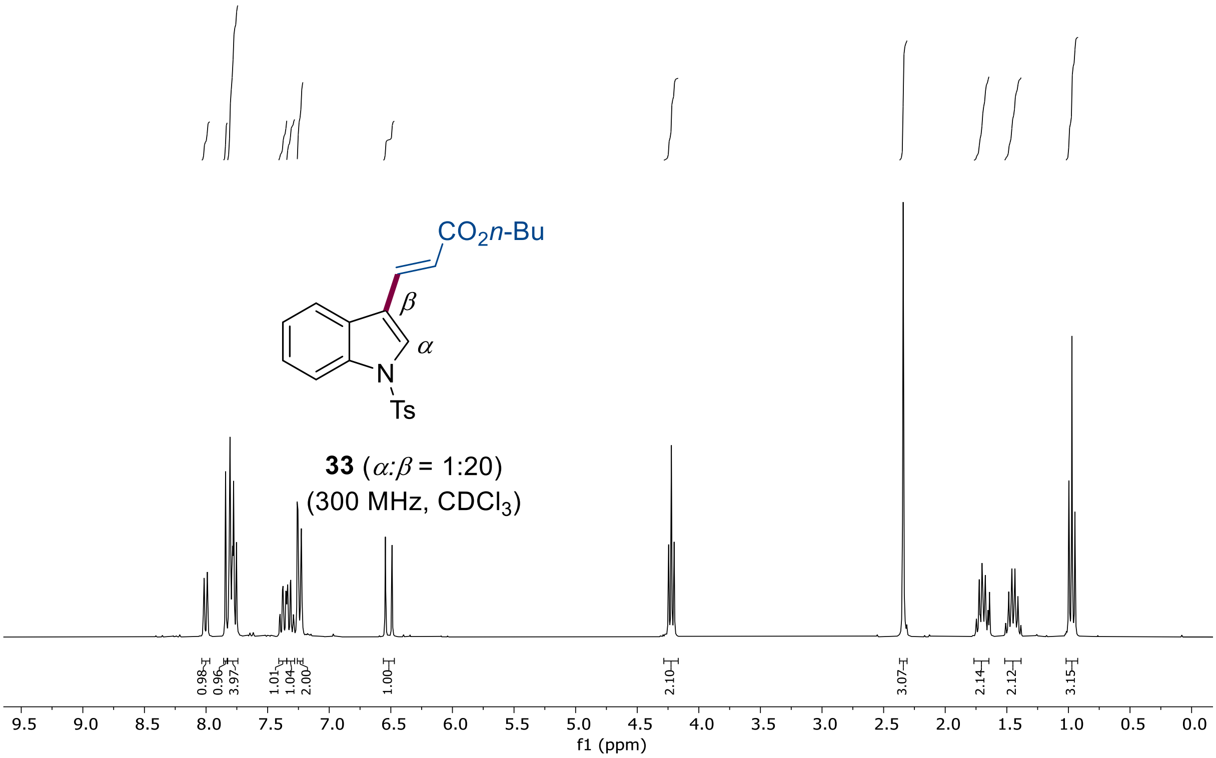

Supplementary Figure 138 H-NMR of compound 33. 300 MHz, $CDCl_3$, RT

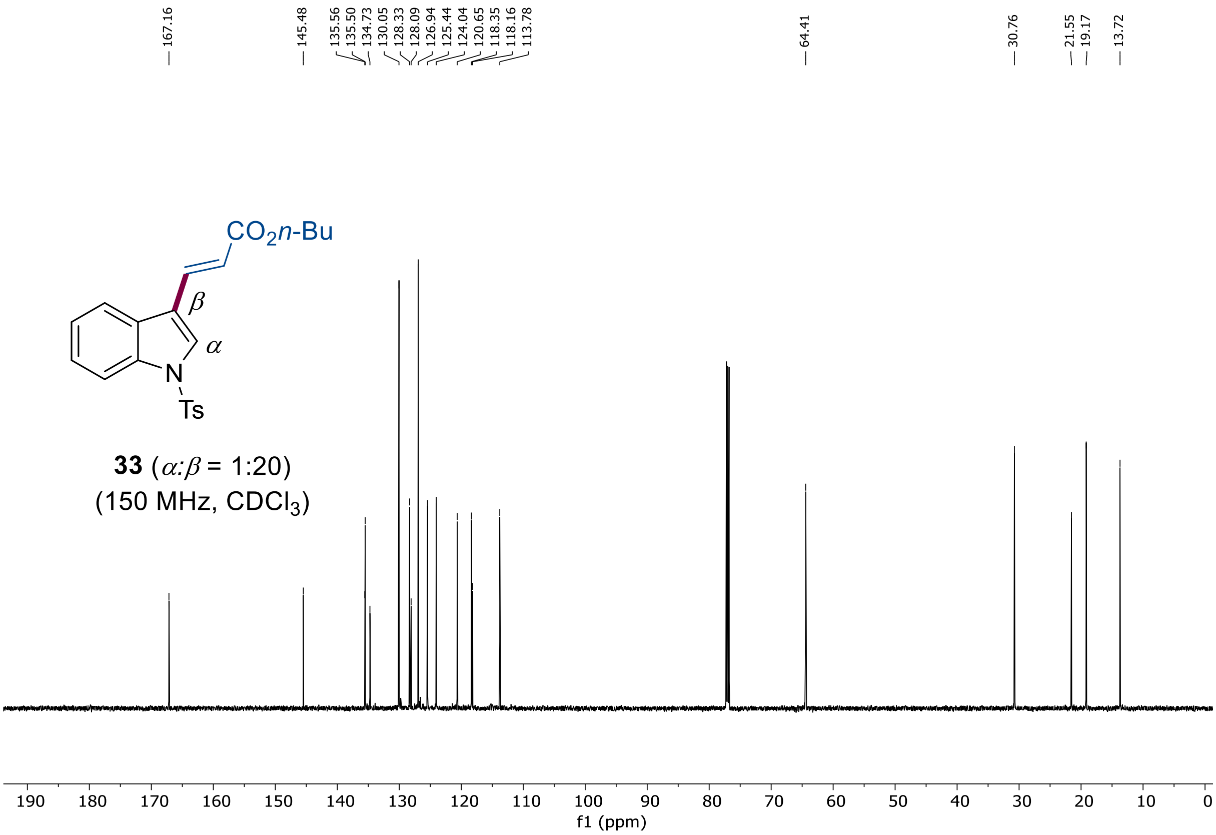

Supplementary Figure 139 C-NMR of compound 33. 150 MHz, $CDCl_3$, RT

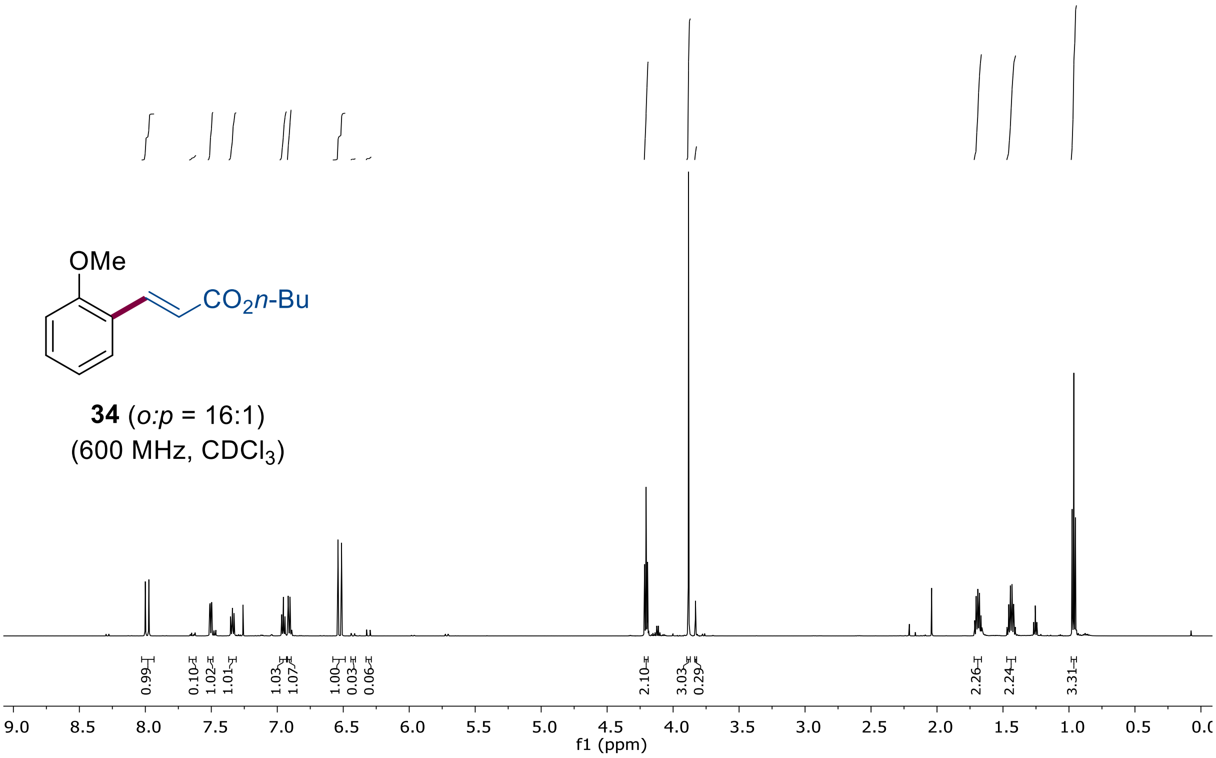

Supplementary Figure 140 H-NMR of compound 34. 600 MHz, $CDCl_3$, RT

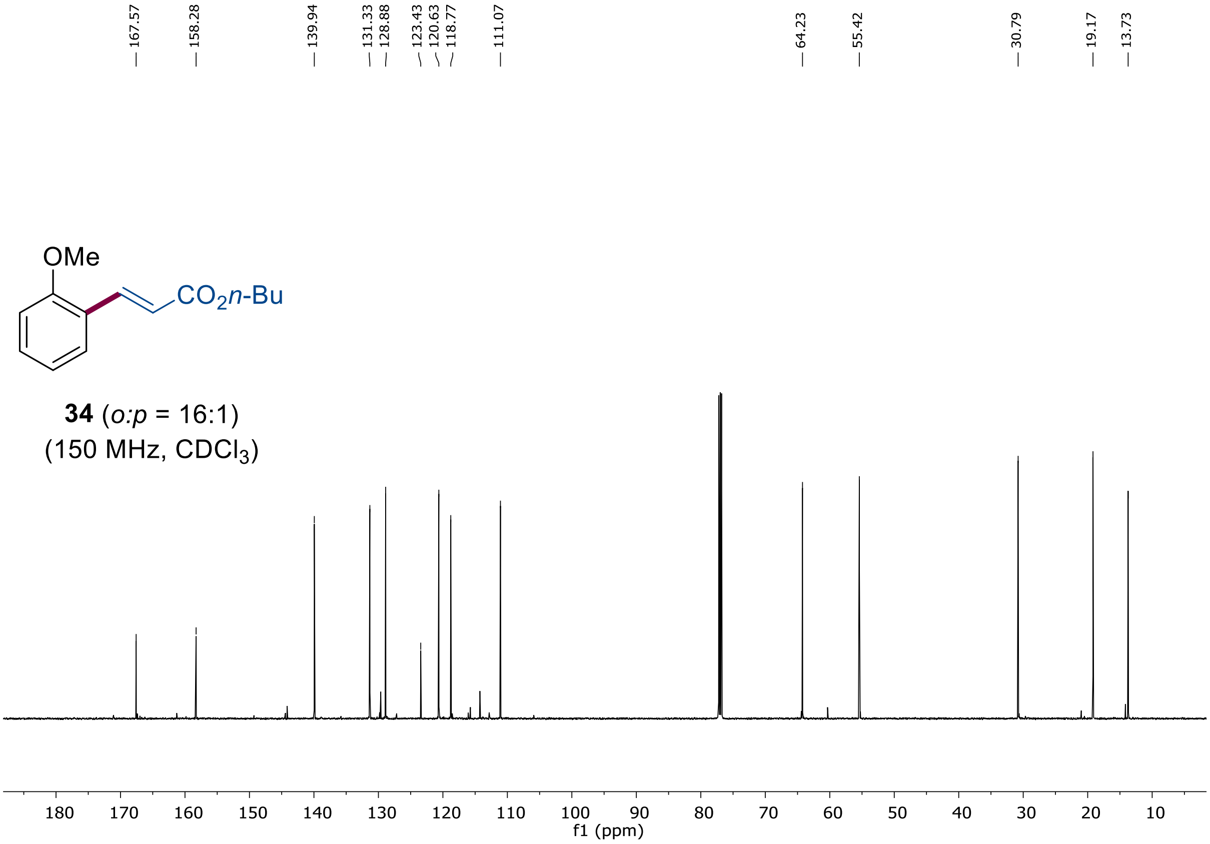

Supplementary Figure 141 C-NMR of compound 34. 150 MHz, $CDCl_3$, RT

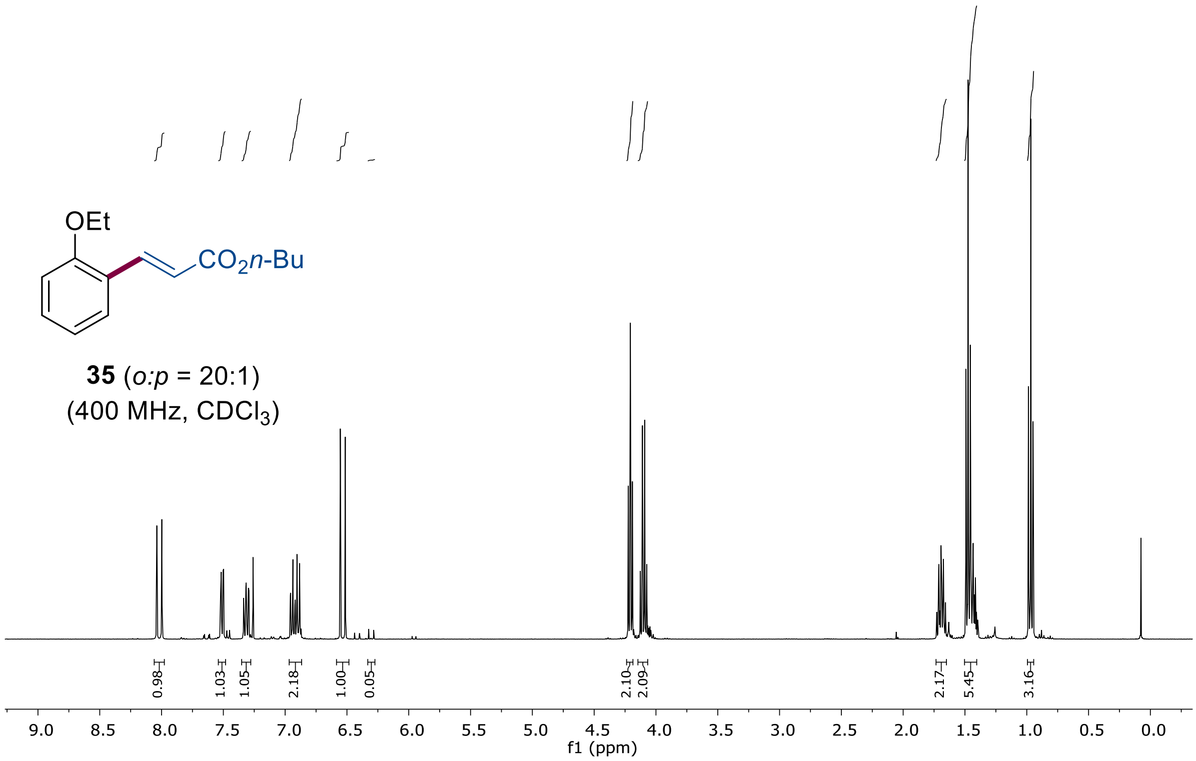

Supplementary Figure 142 H-NMR of compound 35. 400 MHz, $CDCl_3$, RT

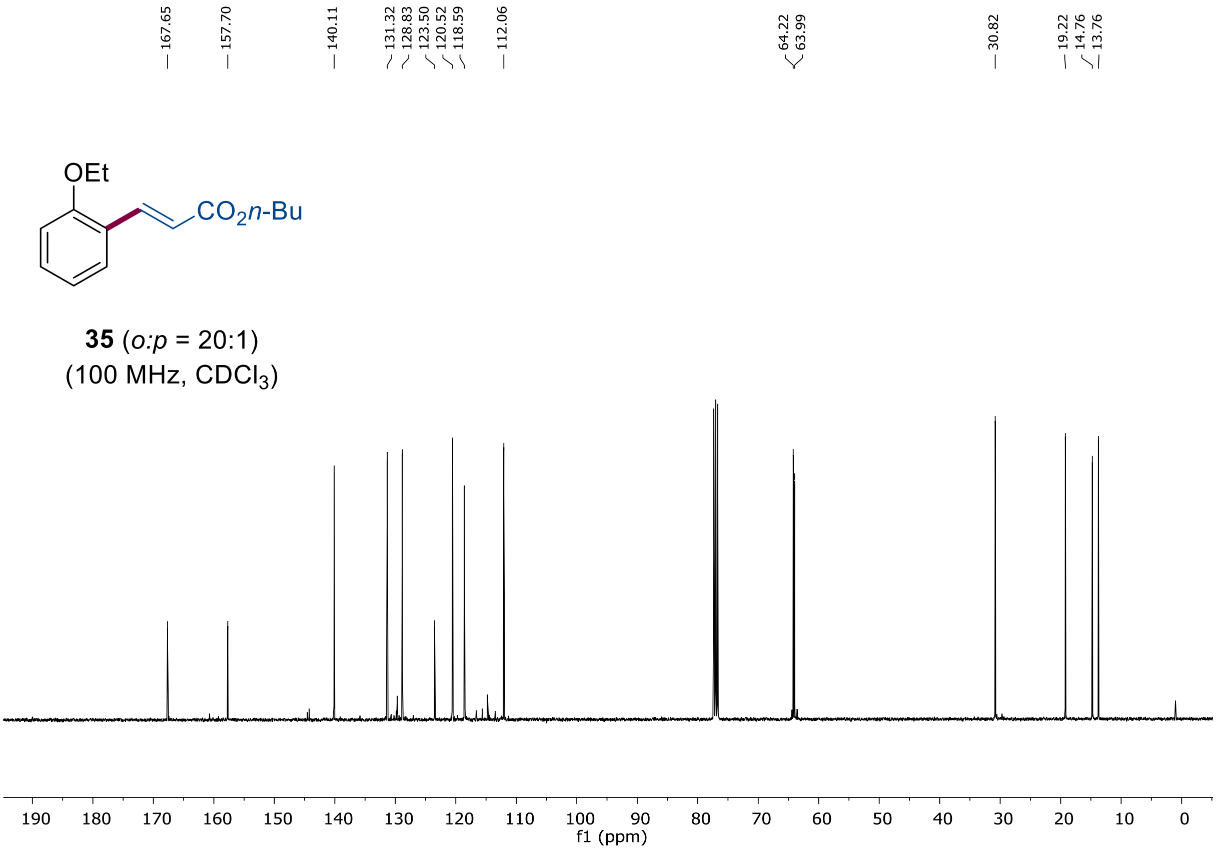

Supplementary Figure 143 C-NMR of compound 35. 100 MHz, $CDCl_3$, RT

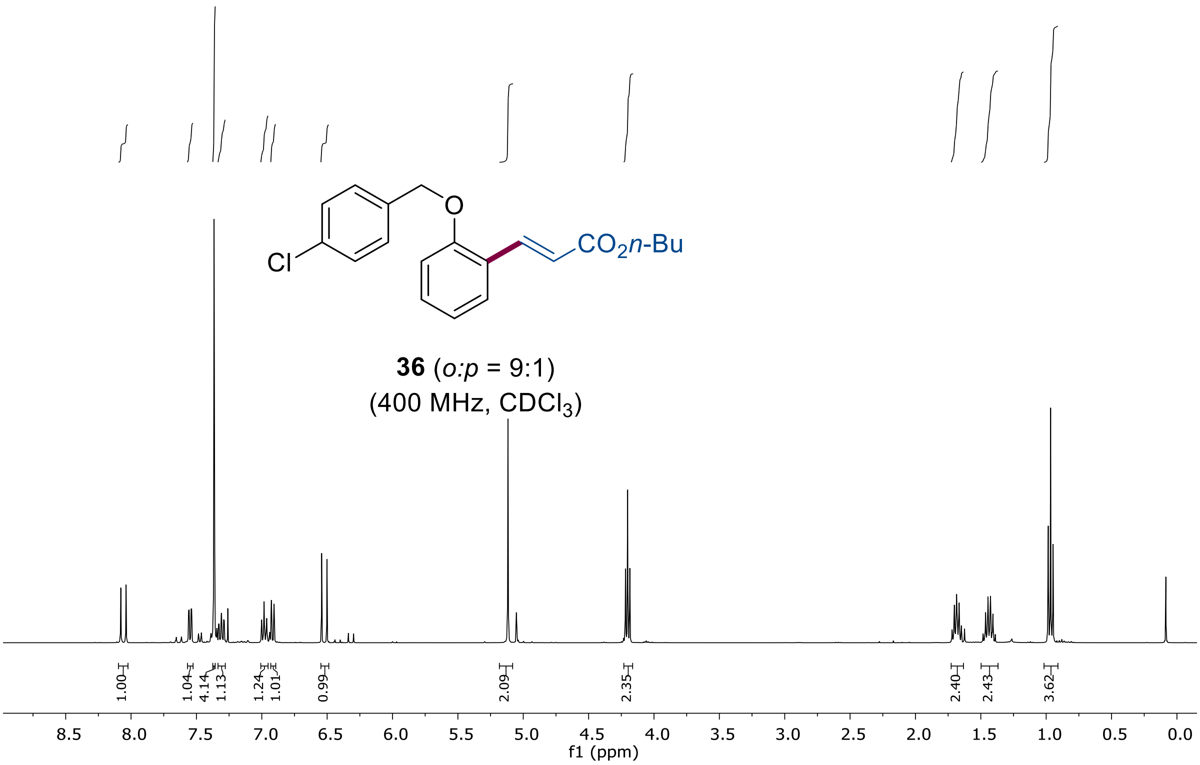

Supplementary Figure 144 H-NMR of compound 36. 400 MHz, CDCl₃, RT

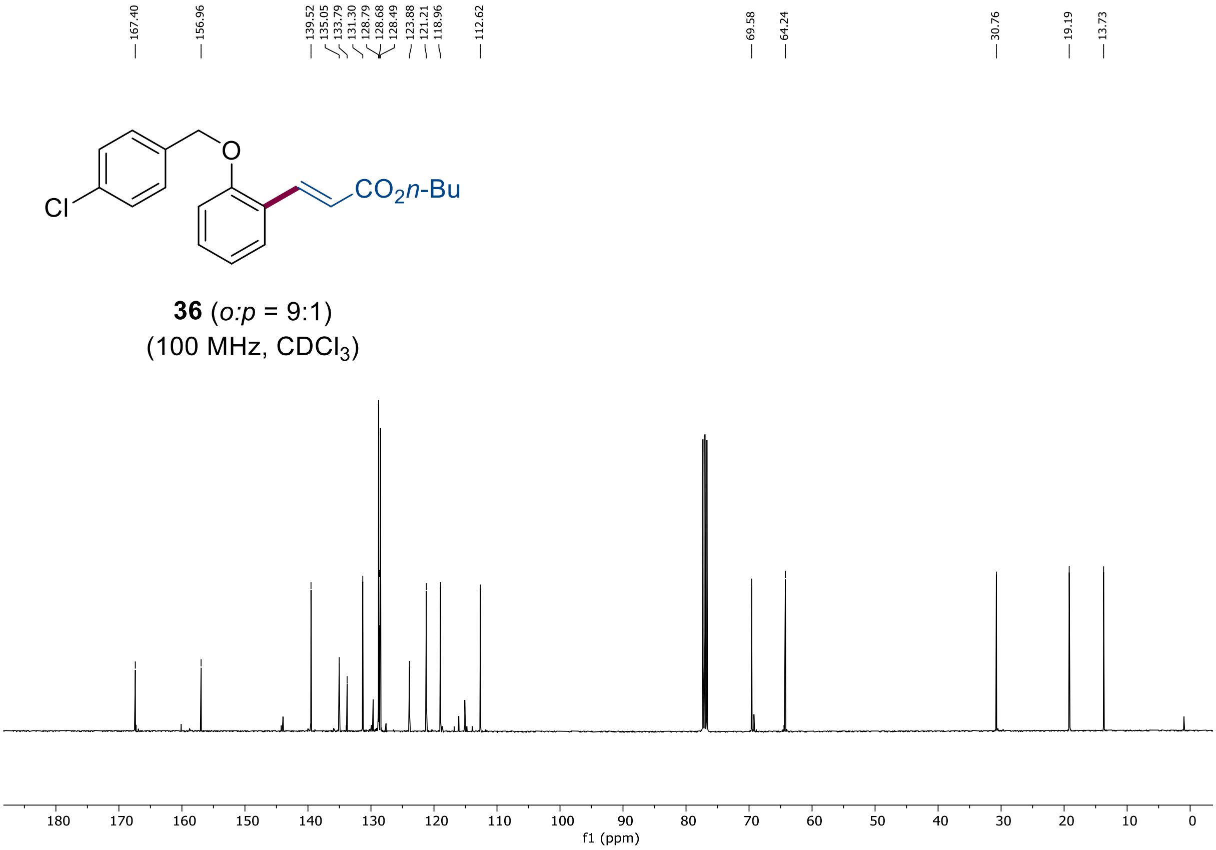

Supplementary Figure 145 C-NMR of compound 36. 100 MHz, CDCl$_3$, RT

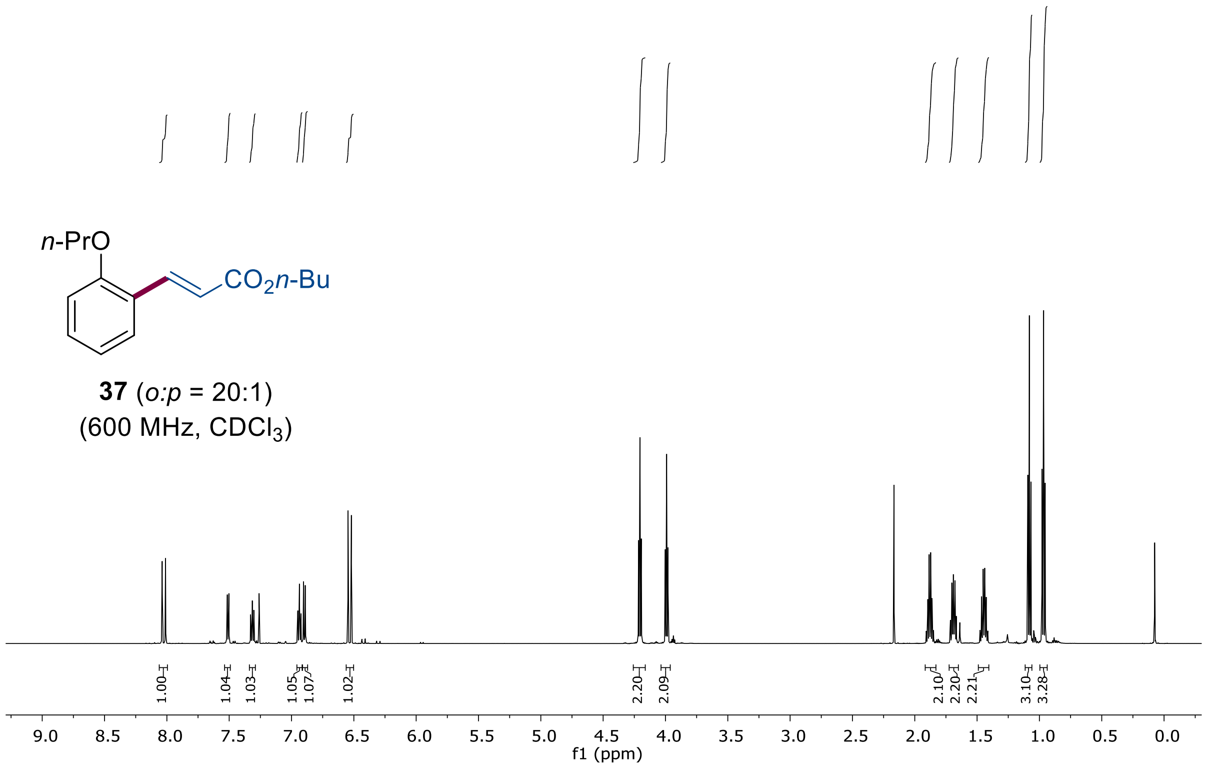

Supplementary Figure 146 H-NMR of compound 37. 600 MHz, CDCl₃, RT

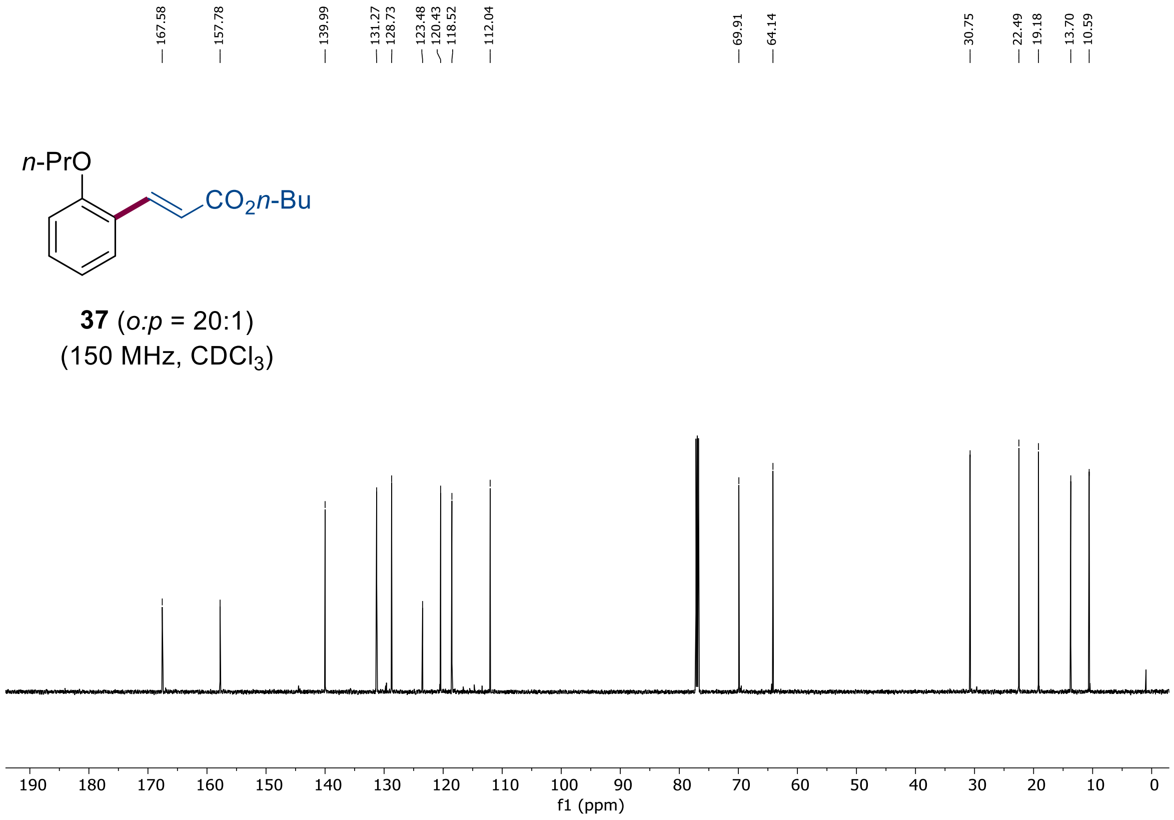

Supplementary Figure 147 C-NMR of compound 37. 150 MHz, CDCl₃, RT

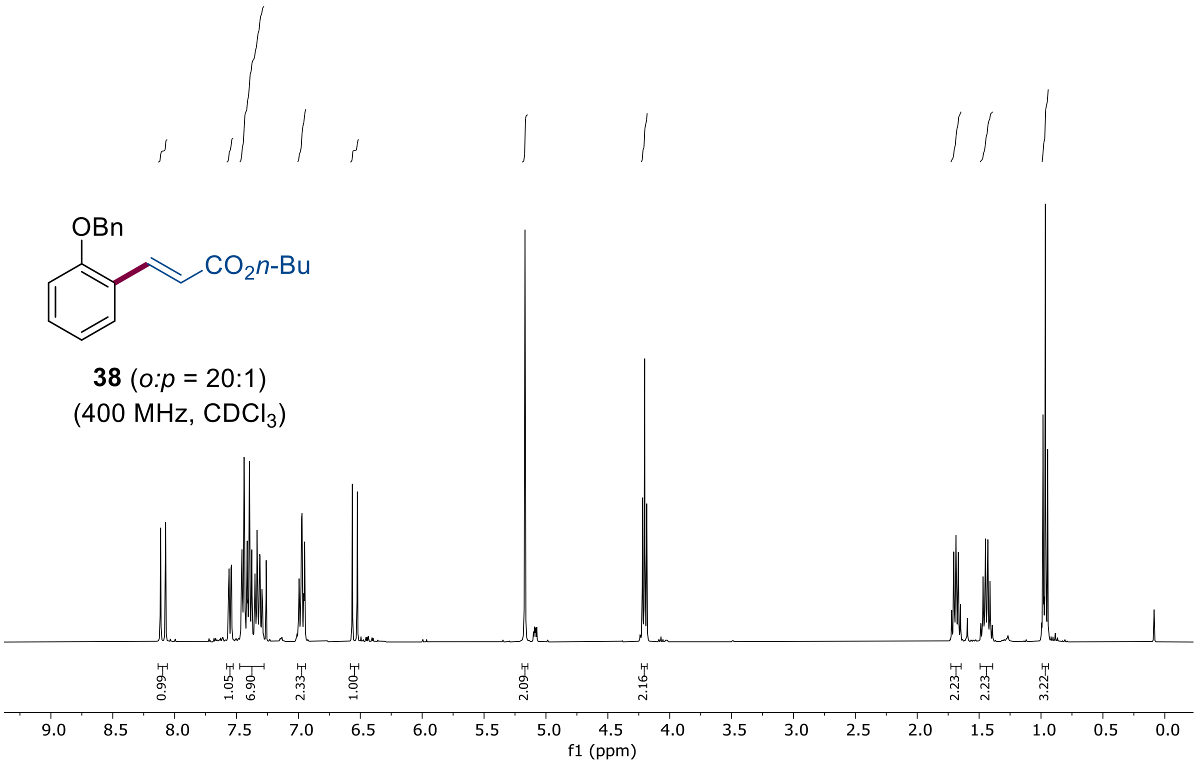

Supplementary Figure 148 H-NMR of compound 38. 400 MHz, $CDCl_3$, RT

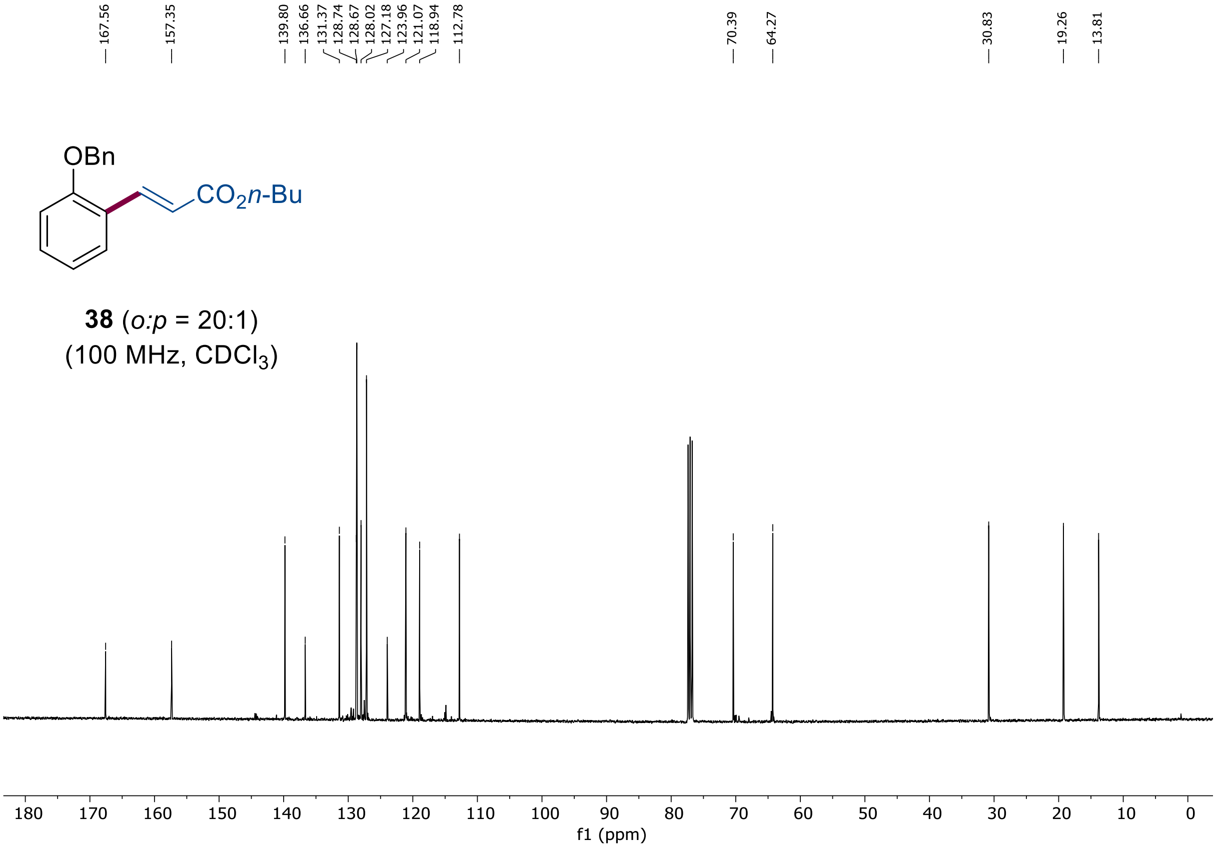

Supplementary Figure 149 C-NMR of compound 38. 100 MHz, $CDCl_3$, RT

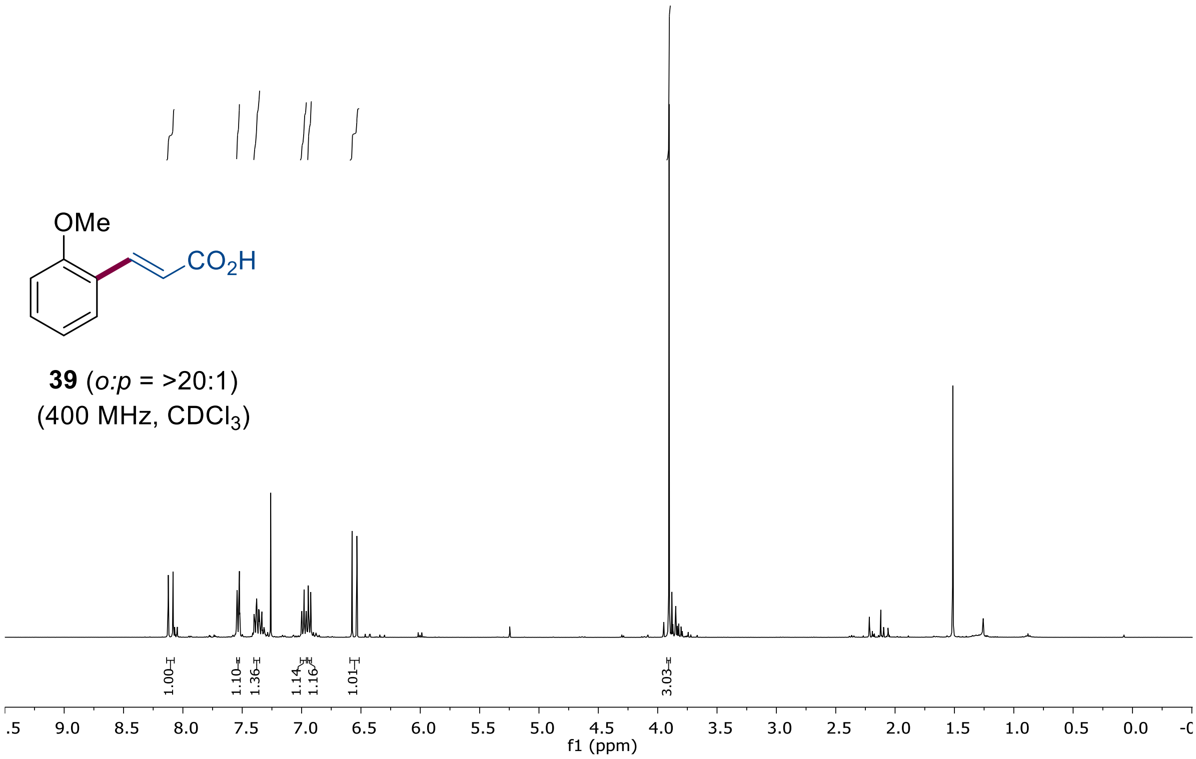

Supplementary Figure 150 H-NMR of compound 39. 400 MHz, $CDCl_3$, RT

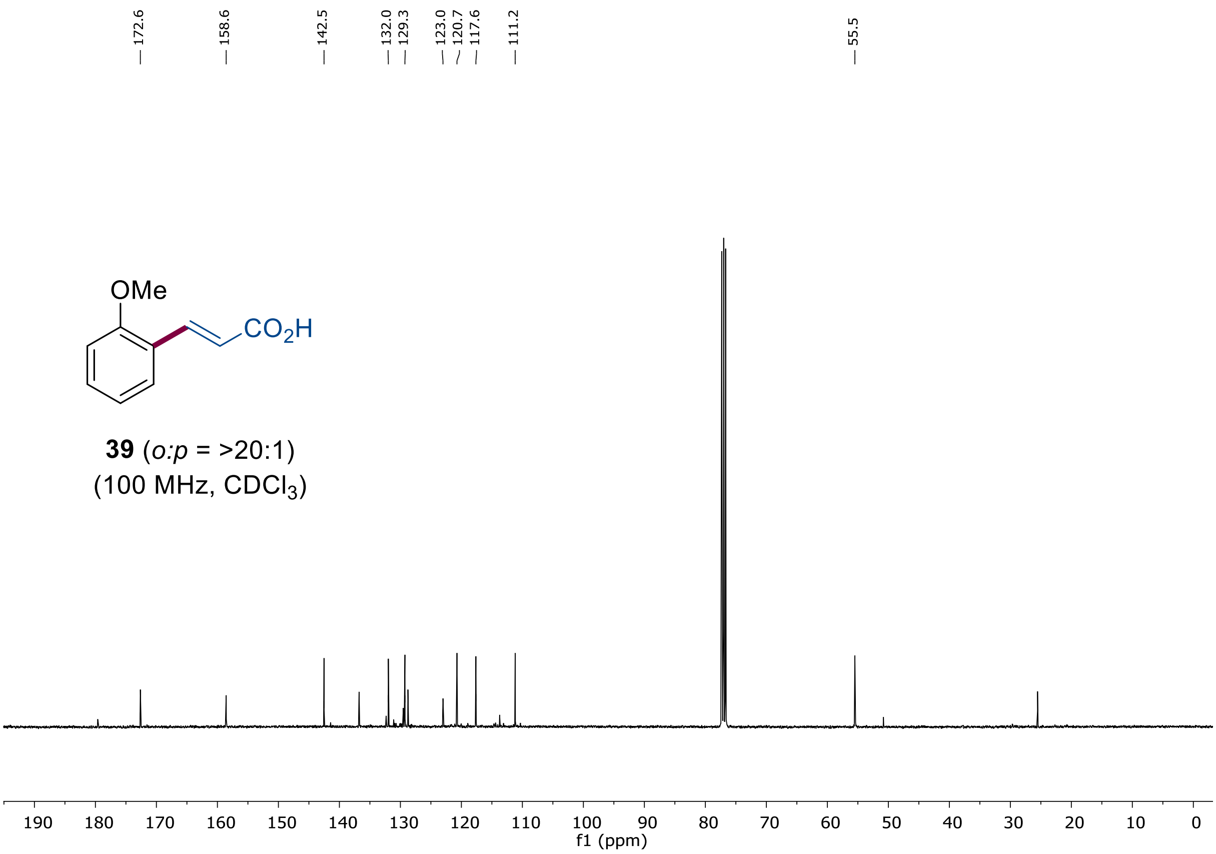

Supplementary Figure 151 C-NMR of compound 39. 100 MHz, $CDCl_3$, RT

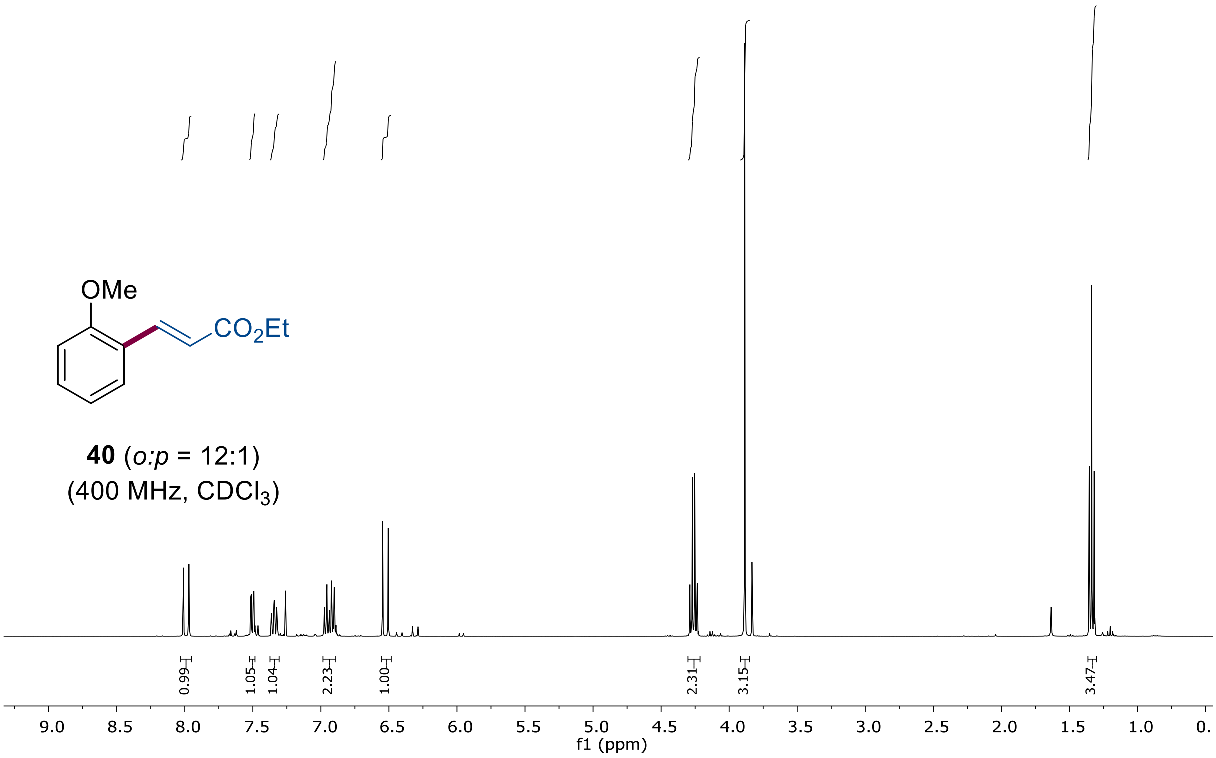

Supplementary Figure 152 H-NMR of compound 40. 400 MHz, $CDCl_3$, RT

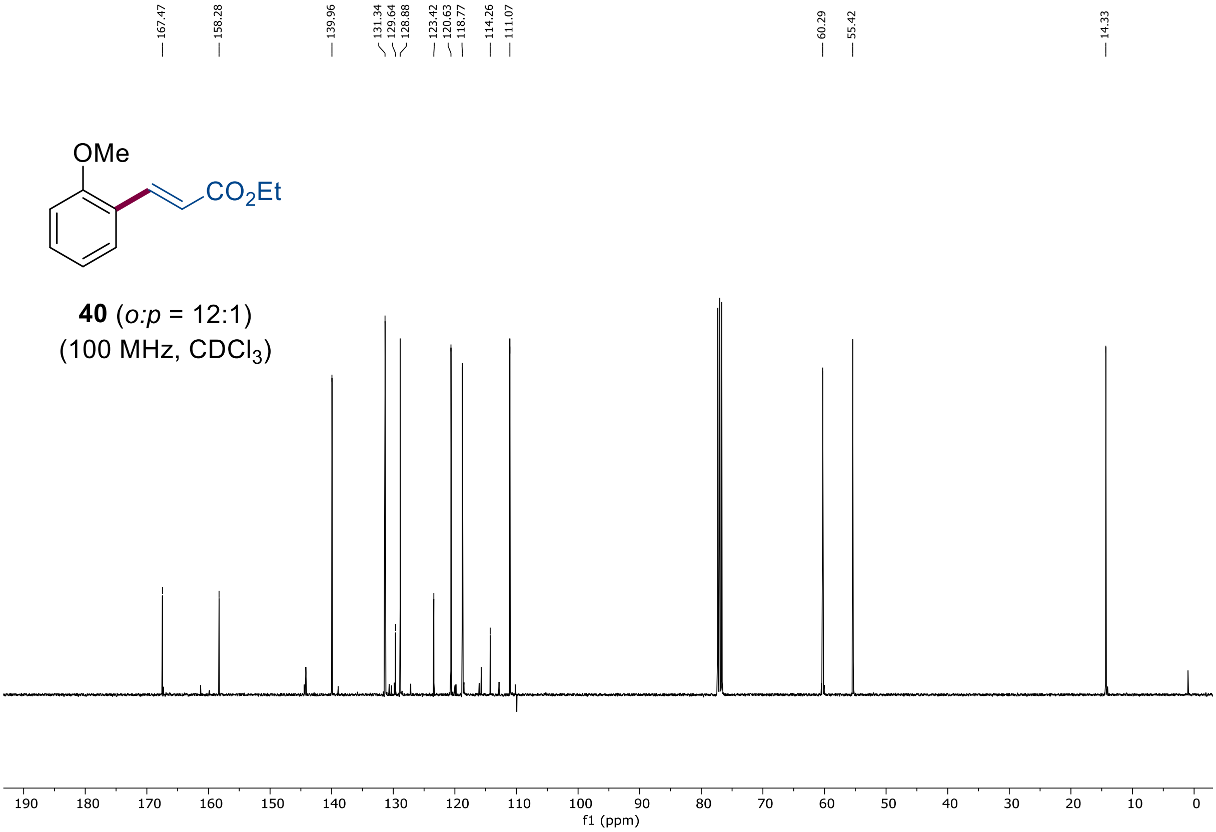

Supplementary Figure 153 C-NMR of compound 40. 100 MHz, $CDCl_3$, RT

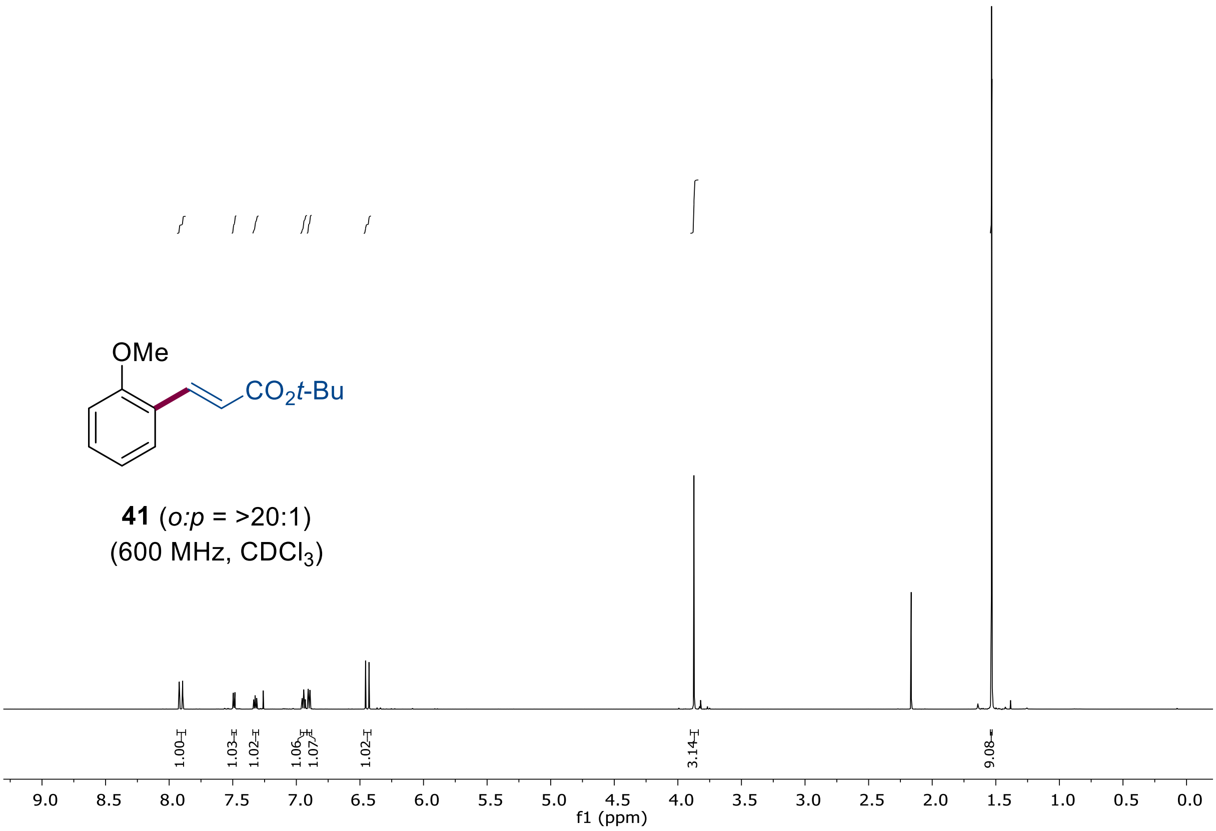

Supplementary Figure 154 H-NMR of compound 41. 600 MHz, $CDCl_3$, RT

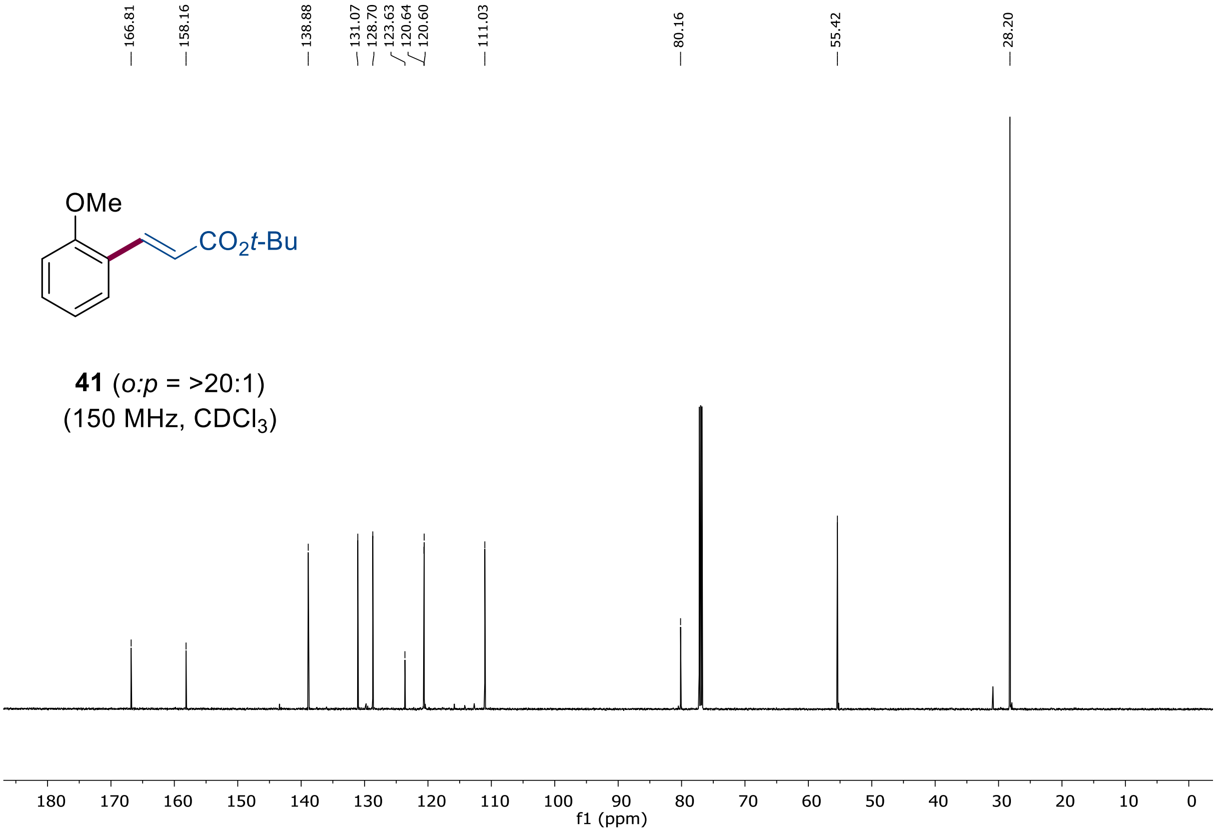

Supplementary Figure 155 C-NMR of compound 41. 150 MHz, $CDCl_3$, RT

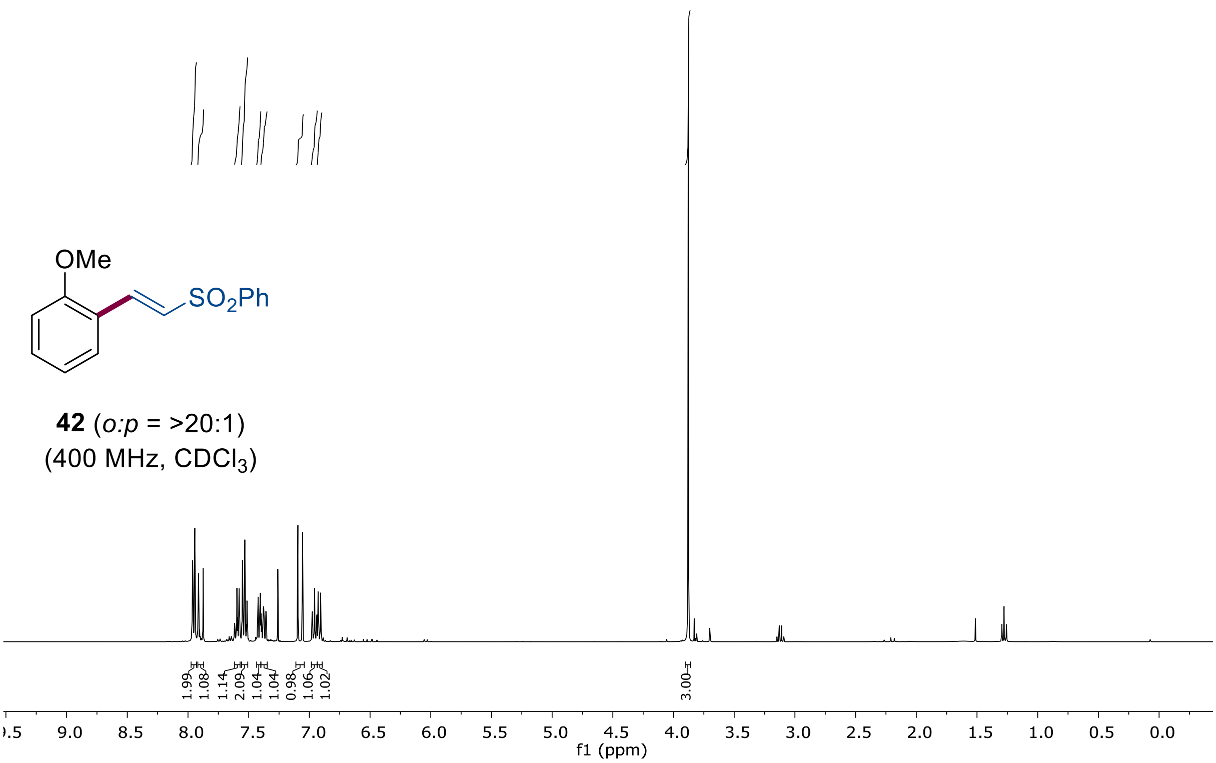

Supplementary Figure 156 H-NMR of compound 42. 400 MHz, CDCl₃, RT

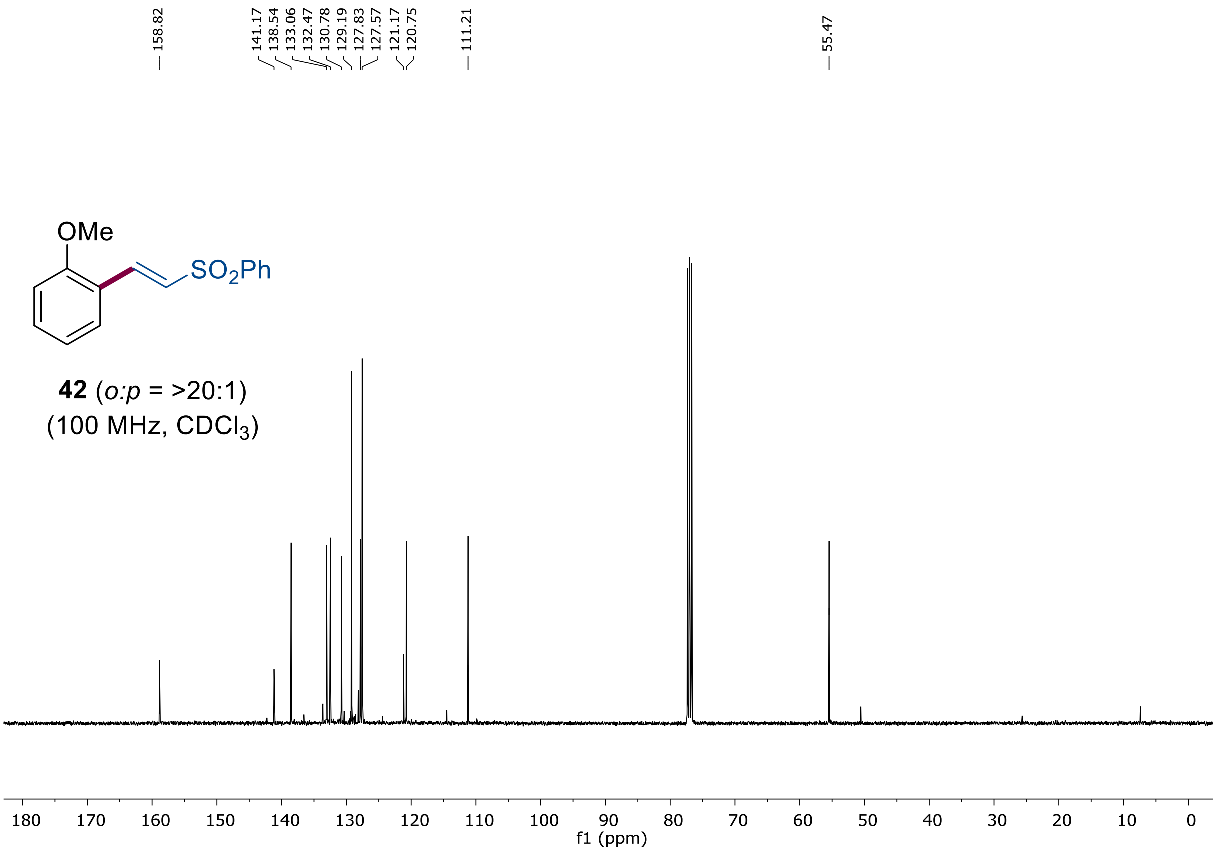

Supplementary Figure 157 C-NMR of compound 42. 100 MHz, CDCl₃, RT

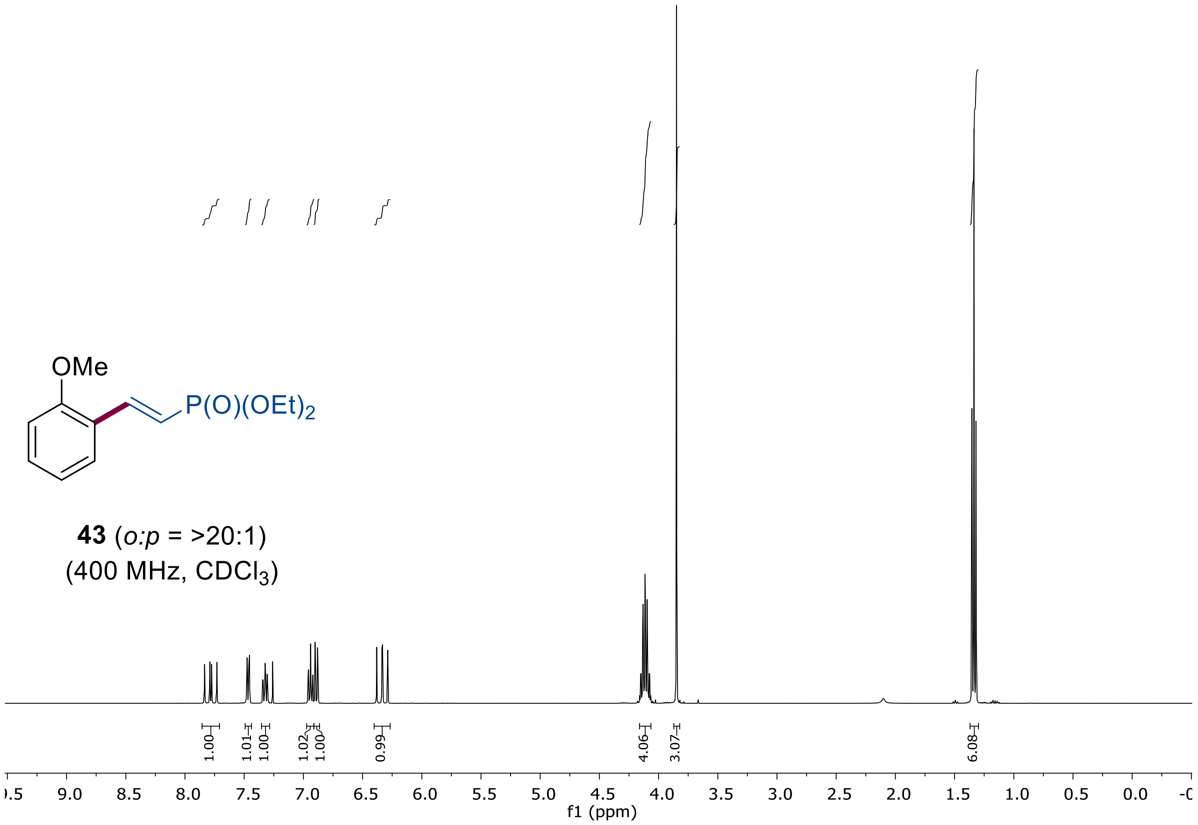

Supplementary Figure 158 H-NMR of compound 43. 400 MHz, $CDCl_3$, RT

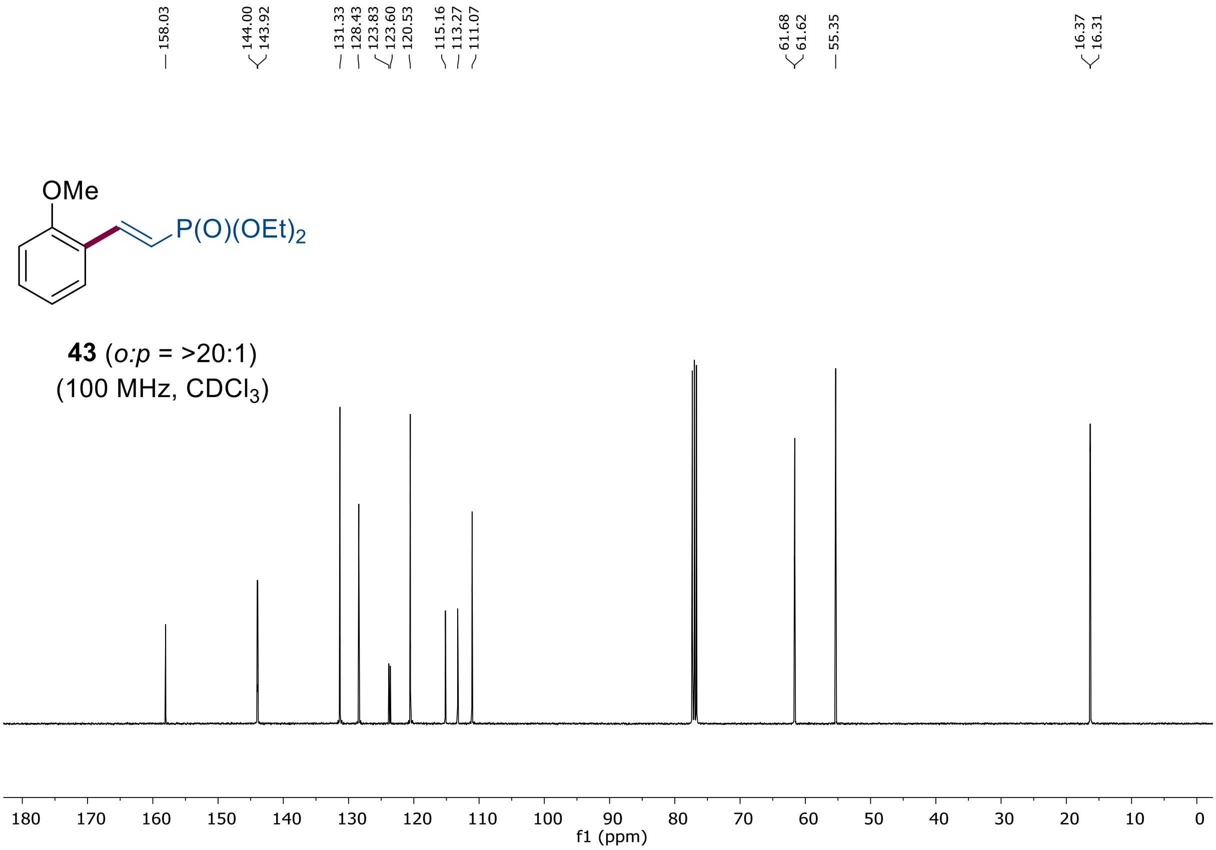

Supplementary Figure 159 C-NMR of compound 43. 100 MHz, $CDCl_3$, RT

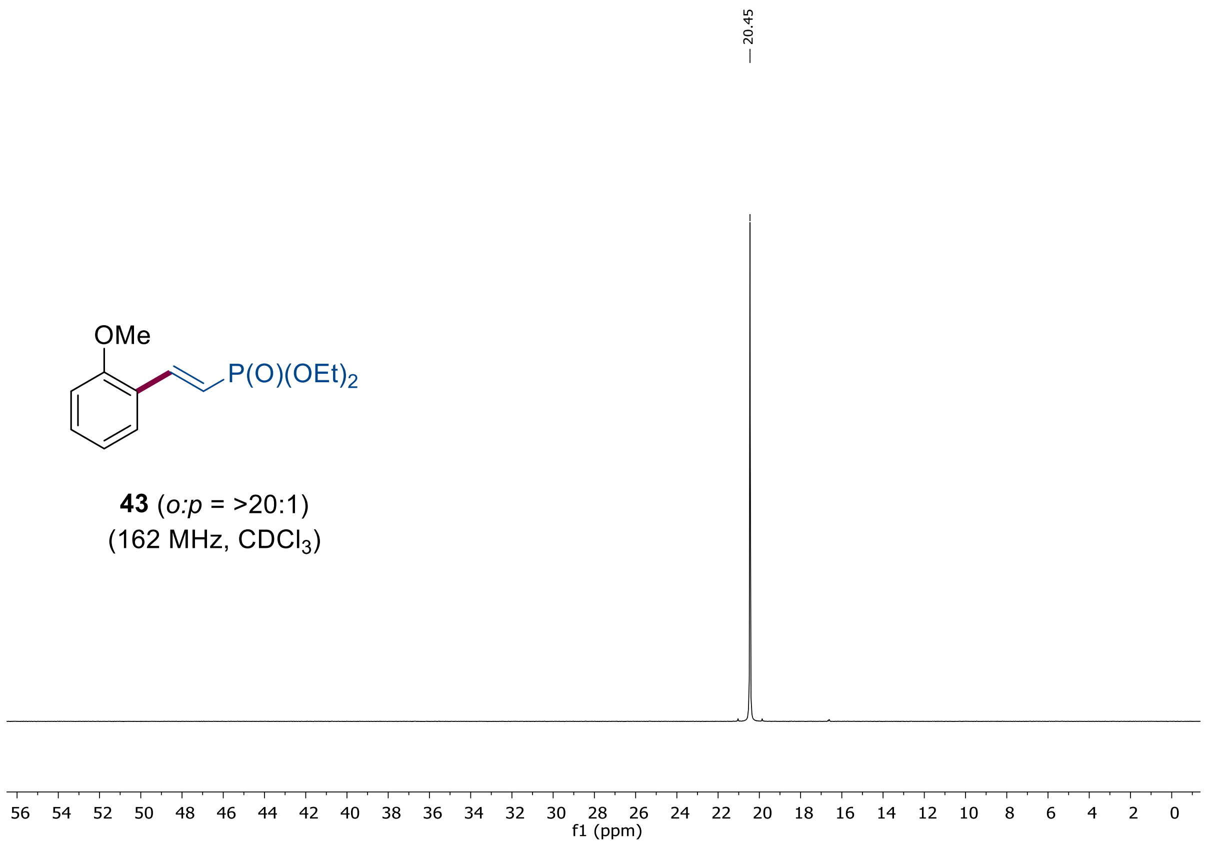

Supplementary Figure 160 P-NMR of compound 43. 162 MHz, $CDCl_3$, RT

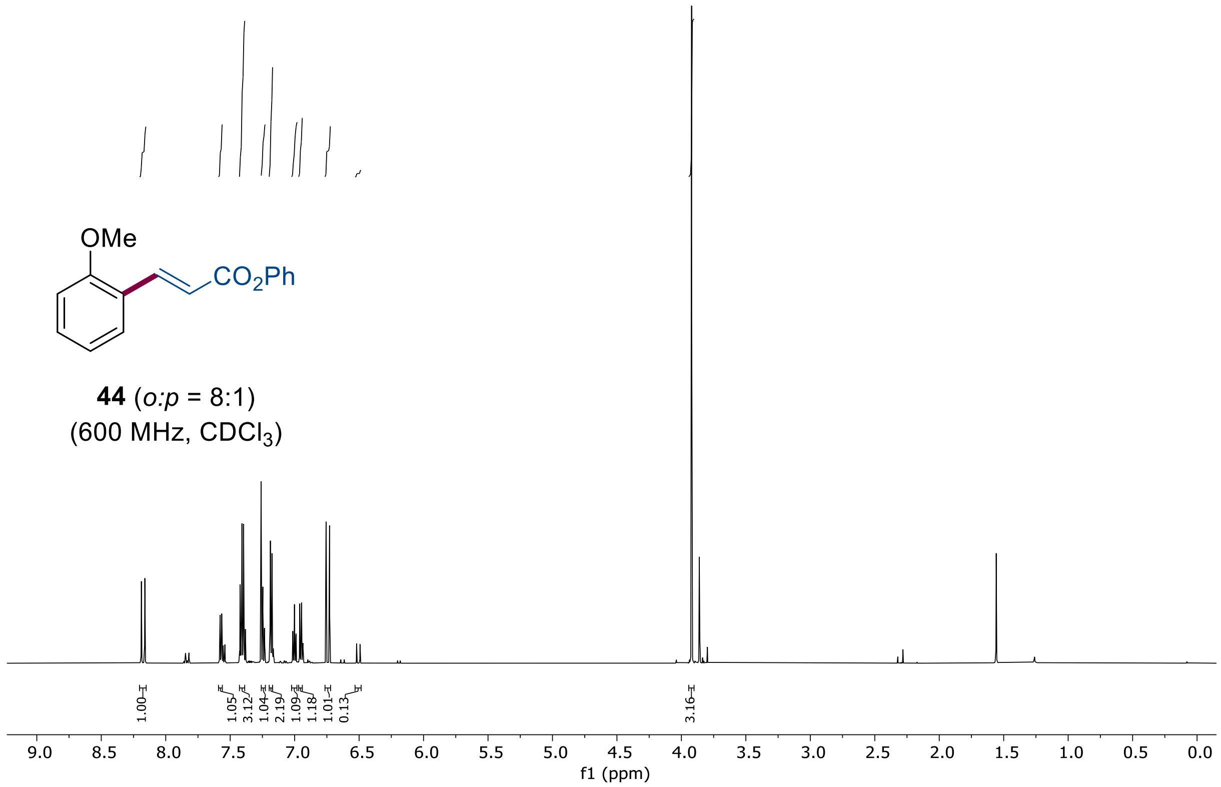

Supplementary Figure 161 H-NMR of compound 44. 600 MHz, $CDCl_3$, RT

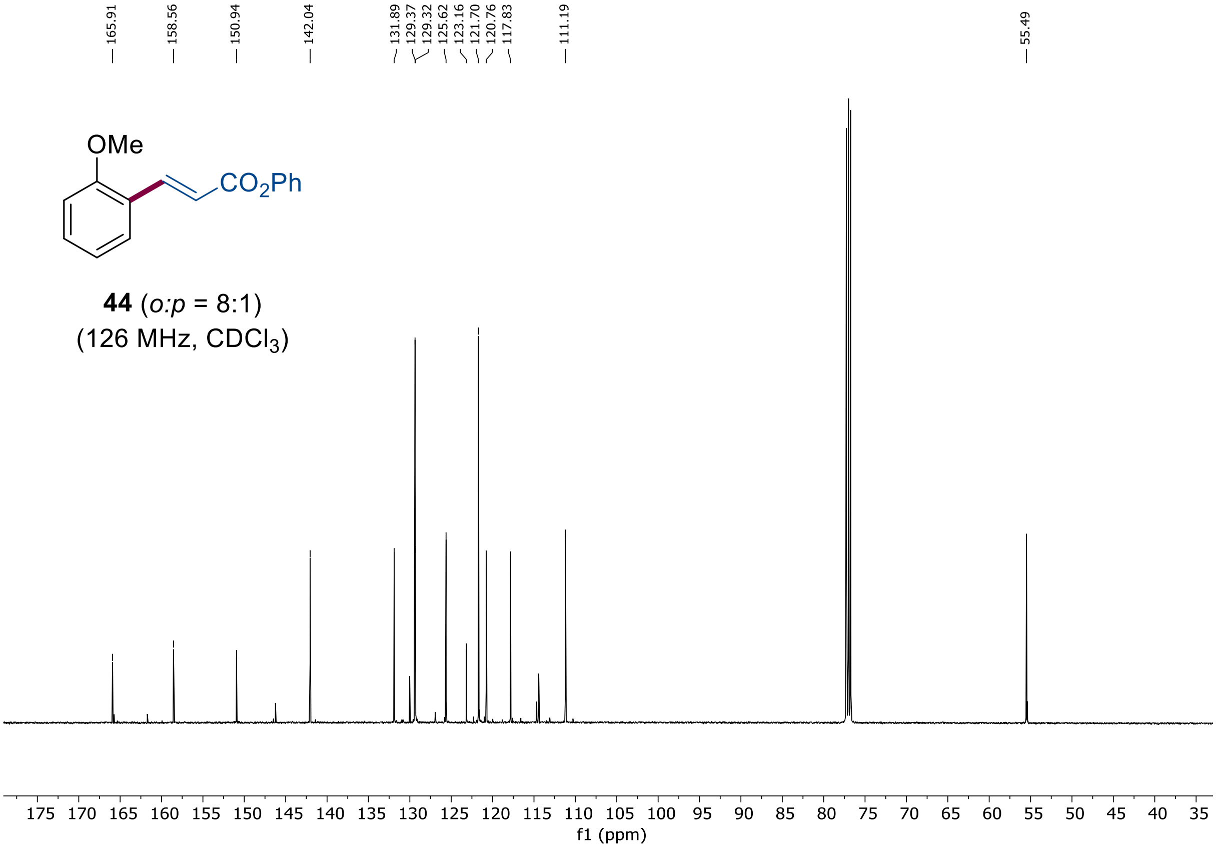

Supplementary Figure 162 C-NMR of compound 44. 126 MHz, $CDCl_3$, RT

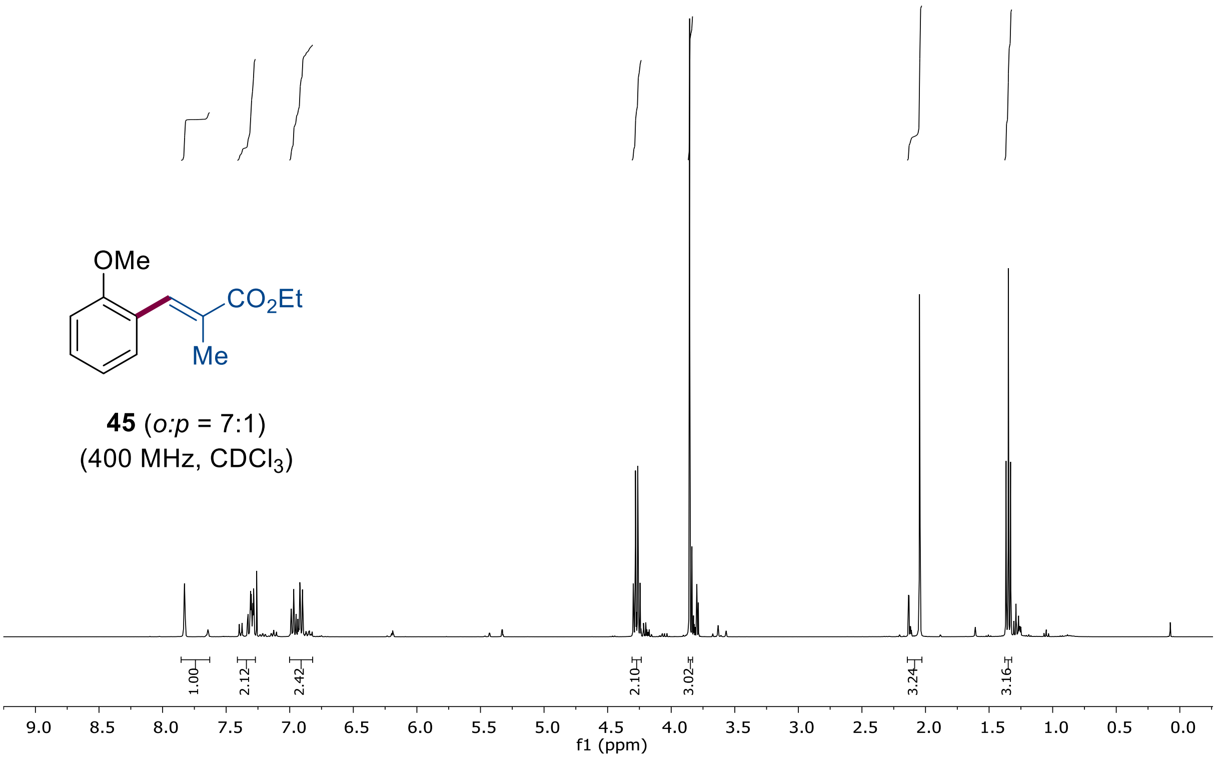

Supplementary Figure 163 H-NMR of compound 45. 400 MHz, $CDCl_3$, RT

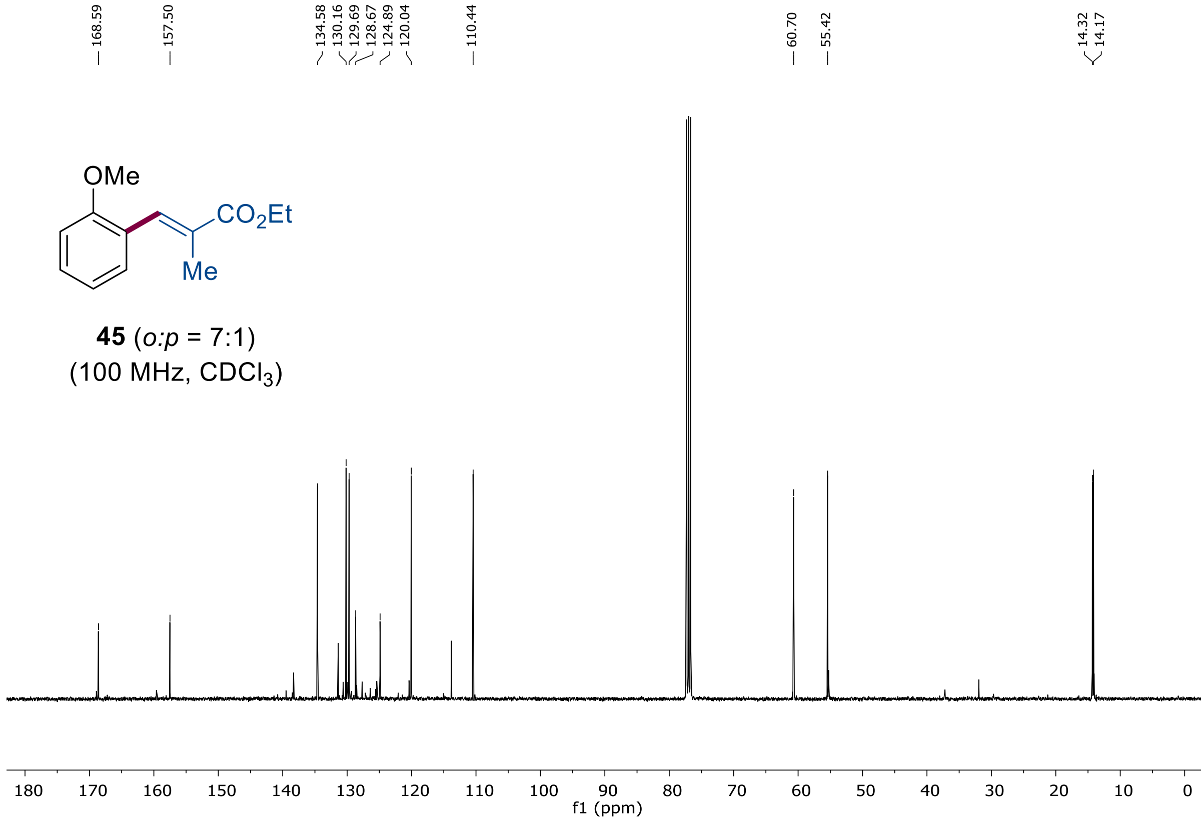

Supplementary Figure 164 C-NMR of compound 45. 100 MHz, $CDCl_3$, RT

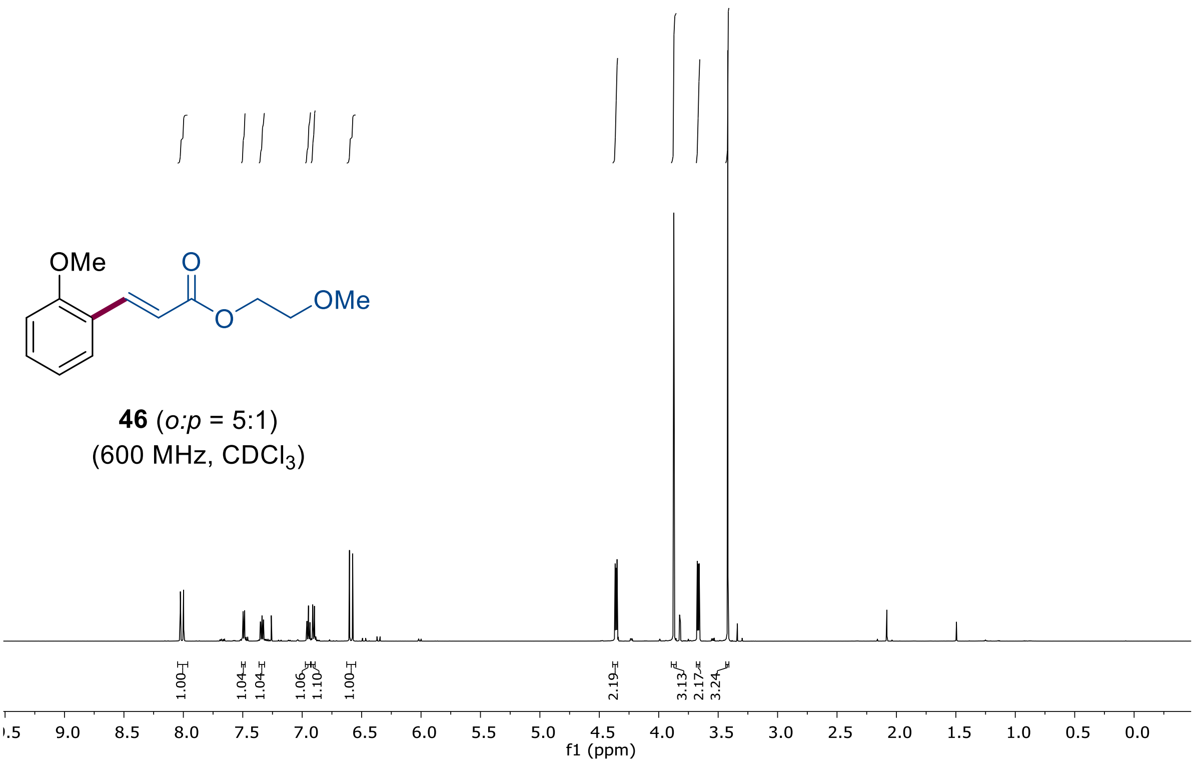

Supplementary Figure 165 H-NMR of compound 46. 600 MHz, $CDCl_3$, RT

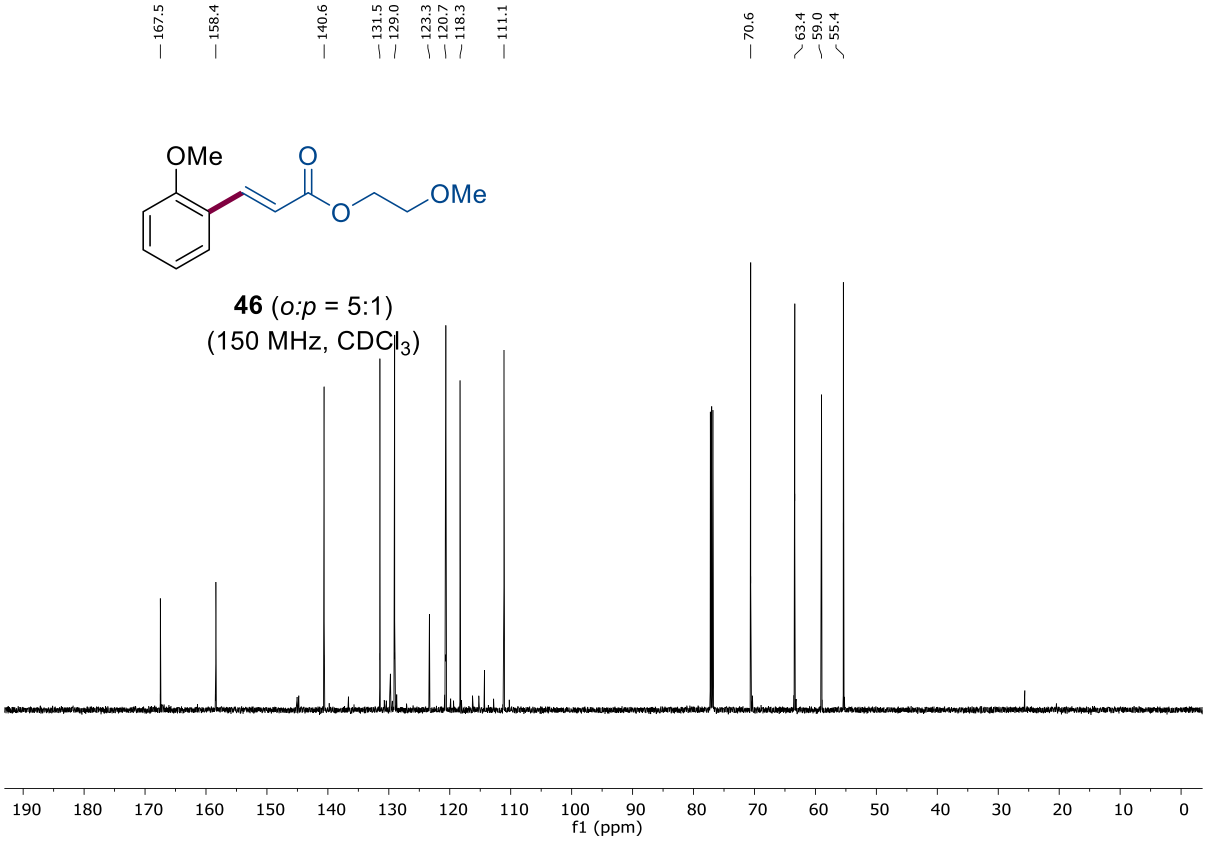

Supplementary Figure 166 C-NMR of compound 46. 150 MHz, $CDCl_3$, RT

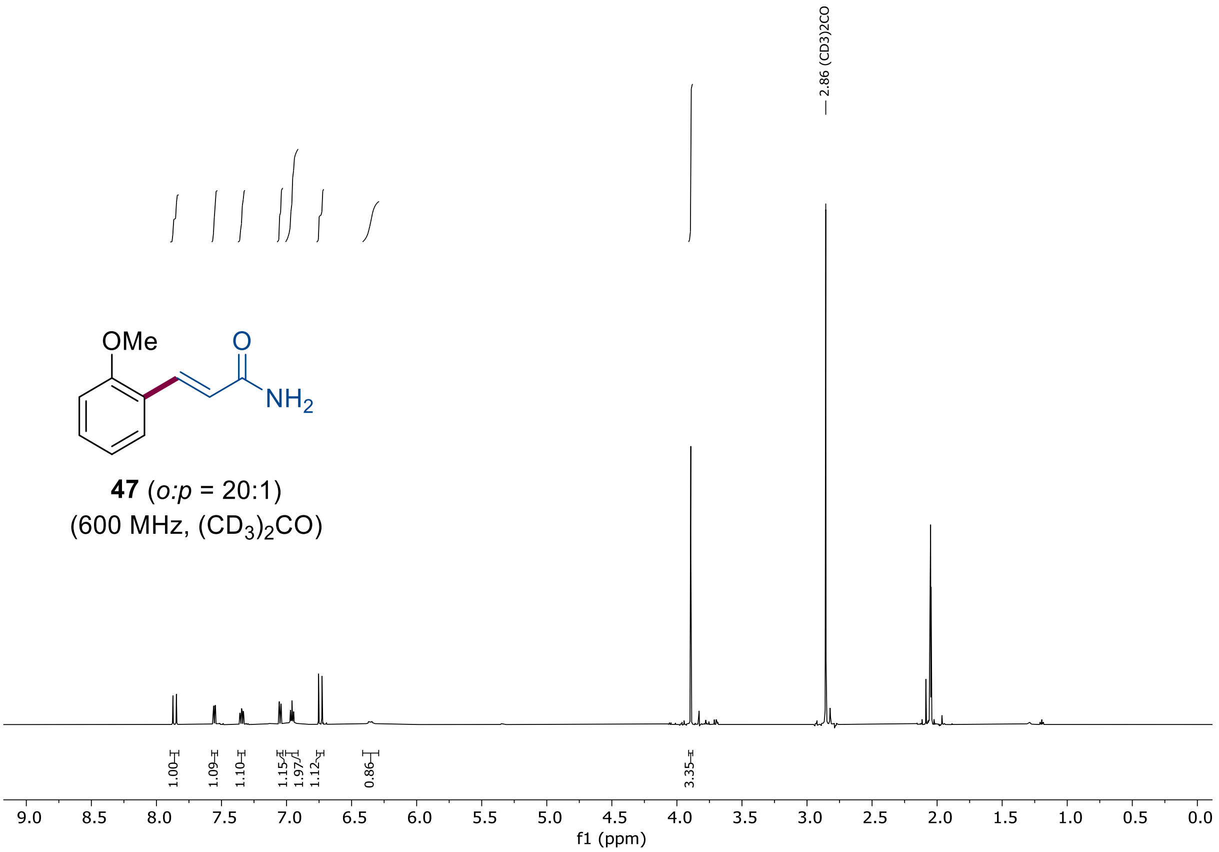

Supplementary Figure 167 H-NMR of compound 47. 600 MHz, $(CD_3)_2CO$, RT

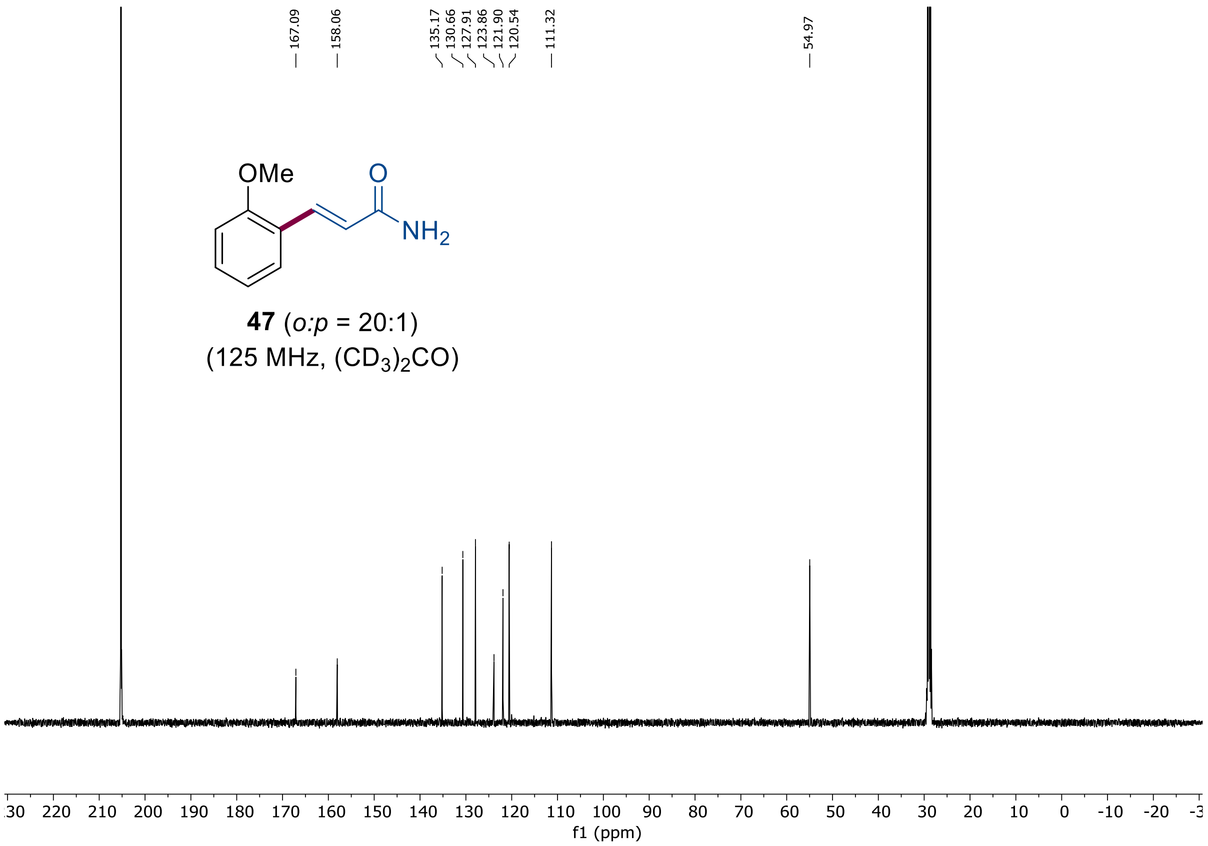

Supplementary Figure 168 C-NMR of compound 47. 125 MHz, $(CD_3)_2CO$, RT

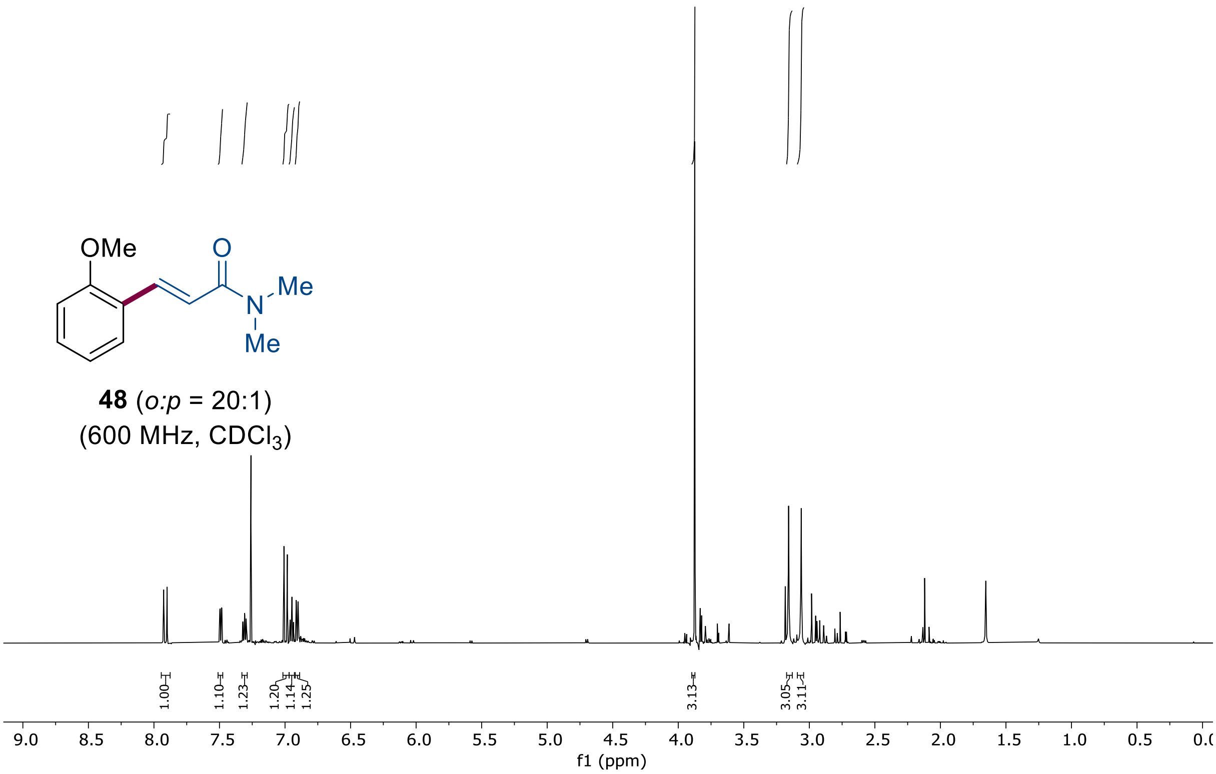

Supplementary Figure 169 H-NMR of compound 48. 600 MHz, $CDCl_3$, RT

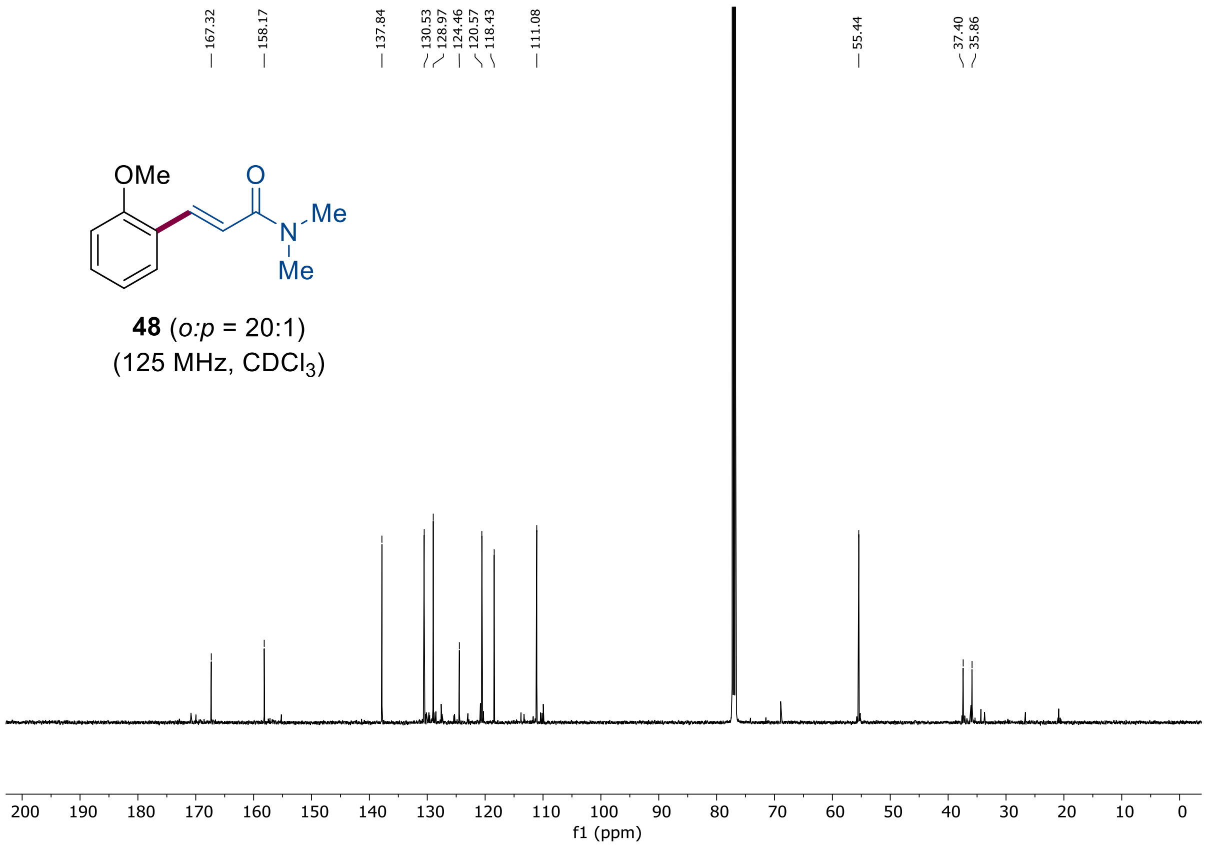

Supplementary Figure 170 C-NMR of compound 48. 600 MHz, $CDCl_3$, RT

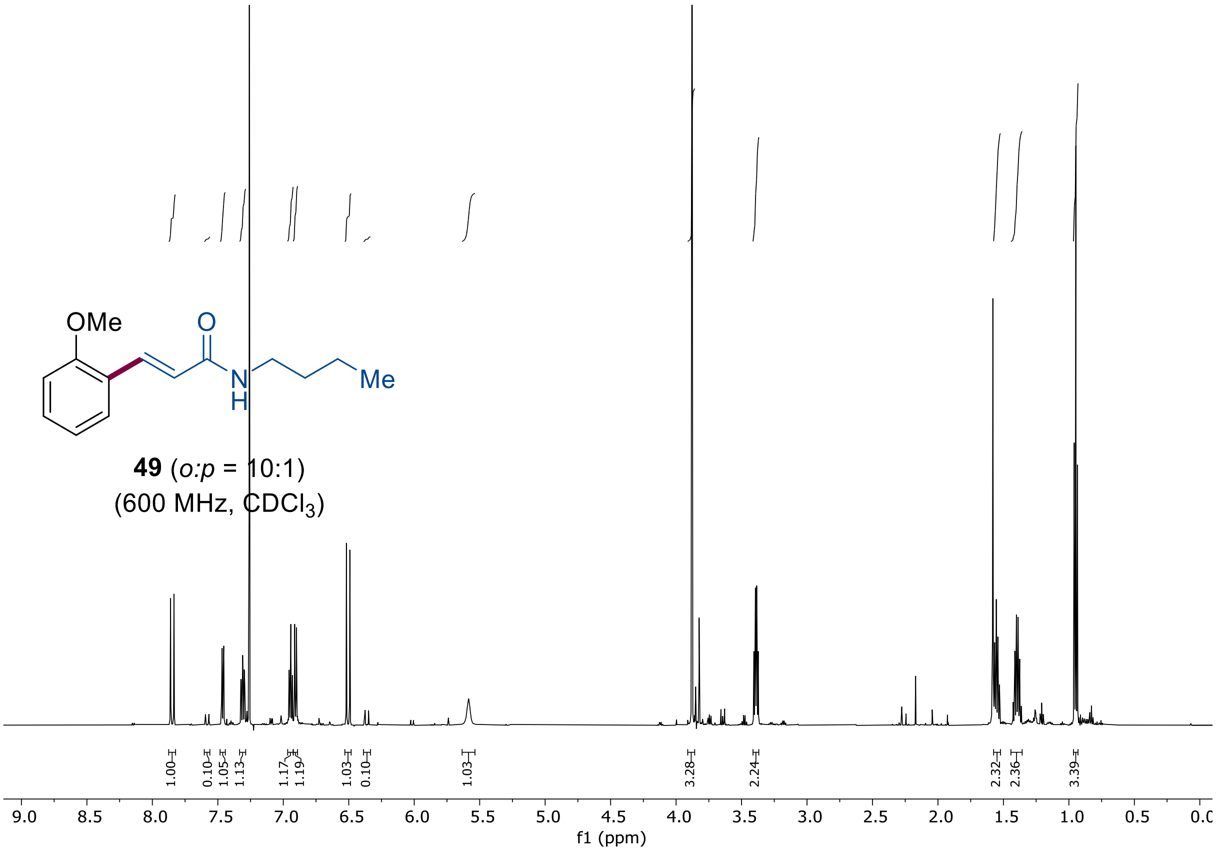

Supplementary Figure 171 H-NMR of compound 49. 600 MHz, $CDCl_3$, RT

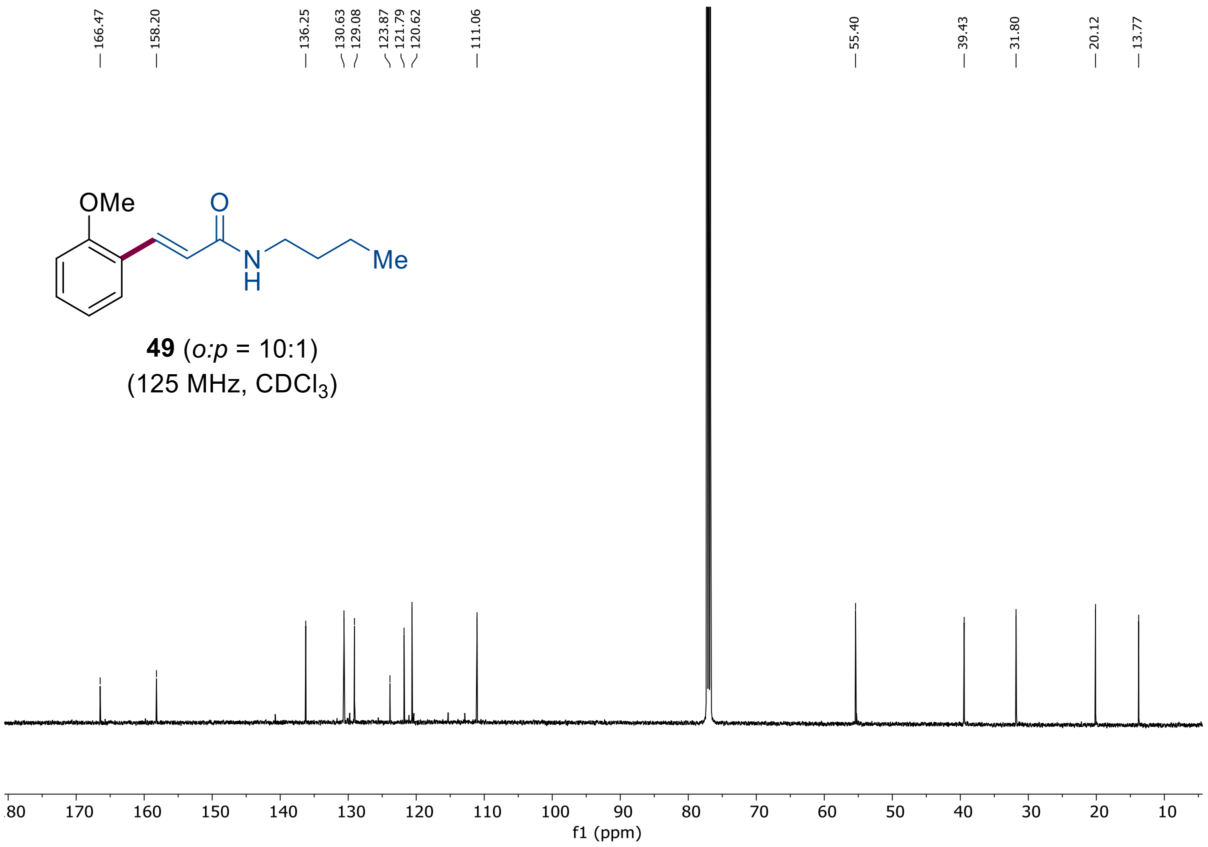

Supplementary Figure 172 C-NMR of compound 49. 125 MHz, $CDCl_3$, RT

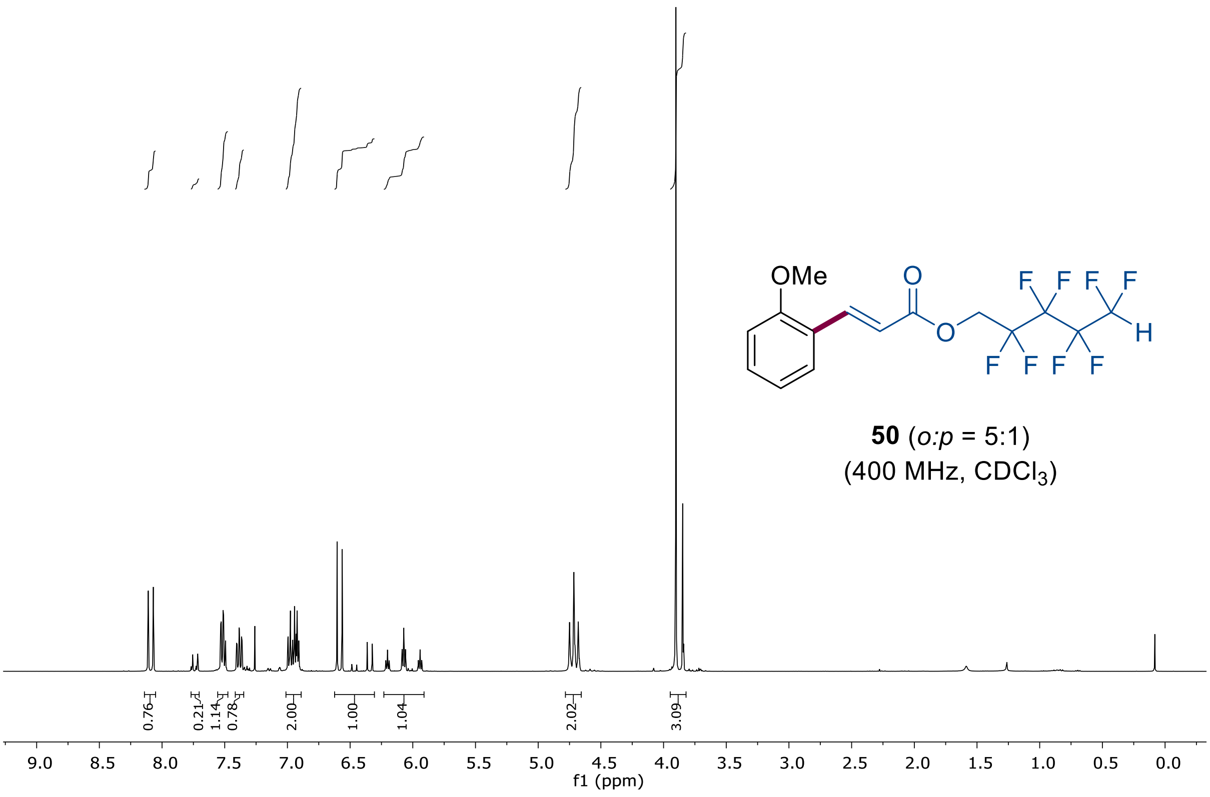

Supplementary Figure 173 H-NMR of compound 50. 400 MHz, $CDCl_3$, RT

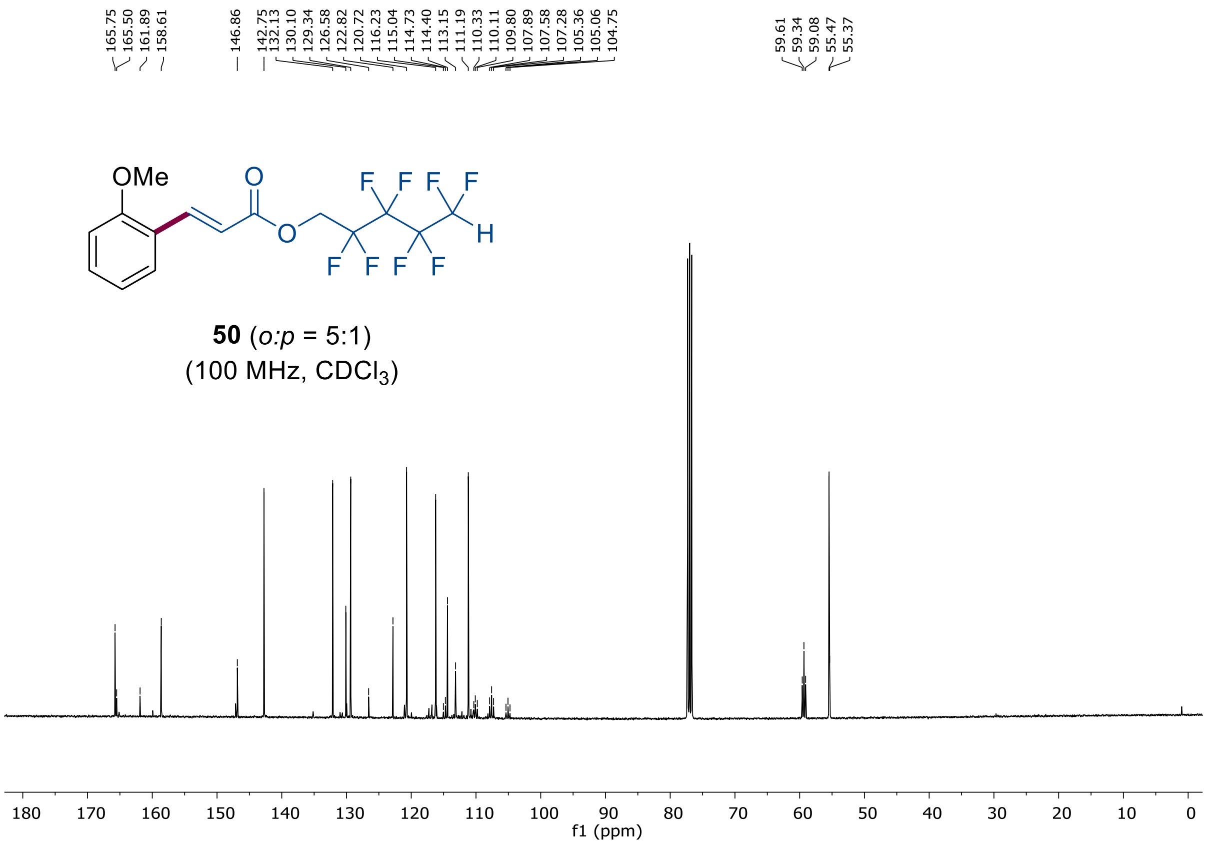

Supplementary Figure 174 C-NMR of compound 50. 100 MHz, $CDCl_3$, RT

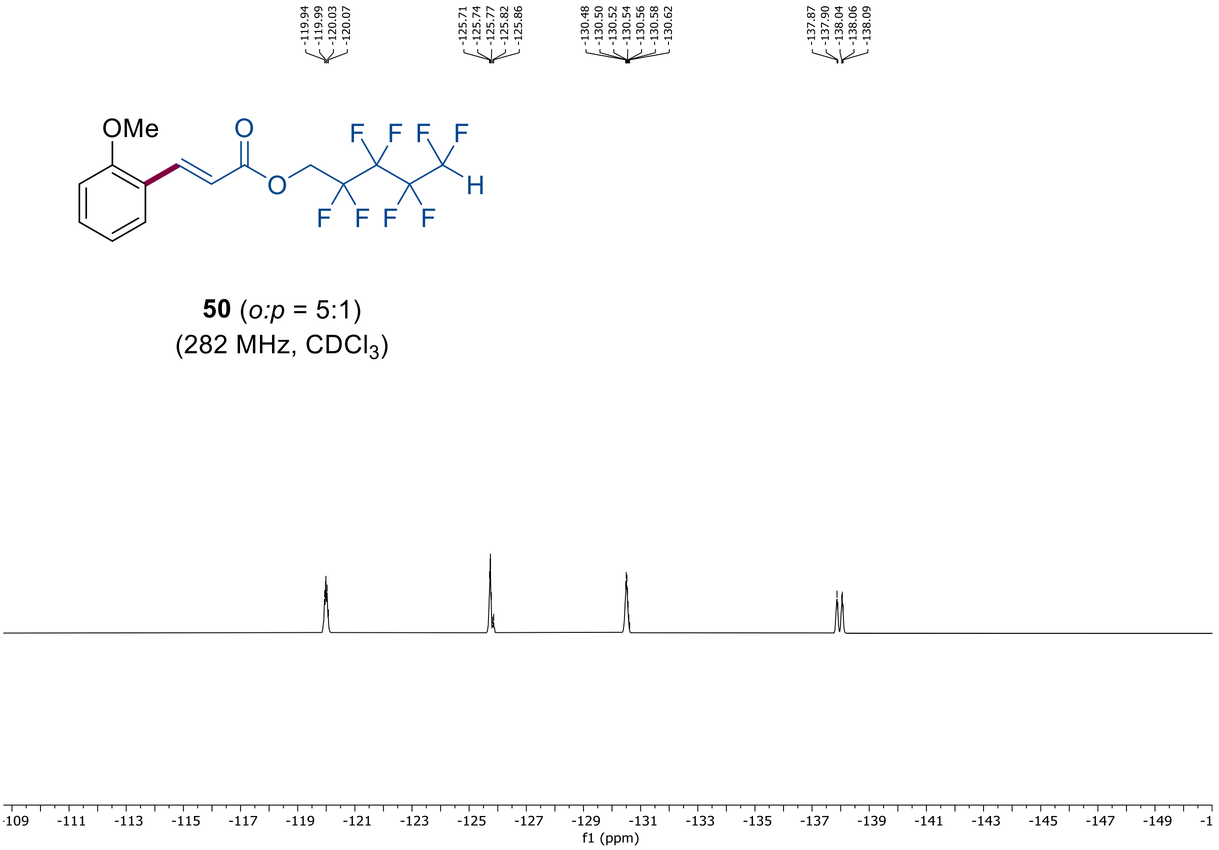

Supplementary Figure 175 F-NMR of compound 50. 282 MHz, $CDCl_3$, RT

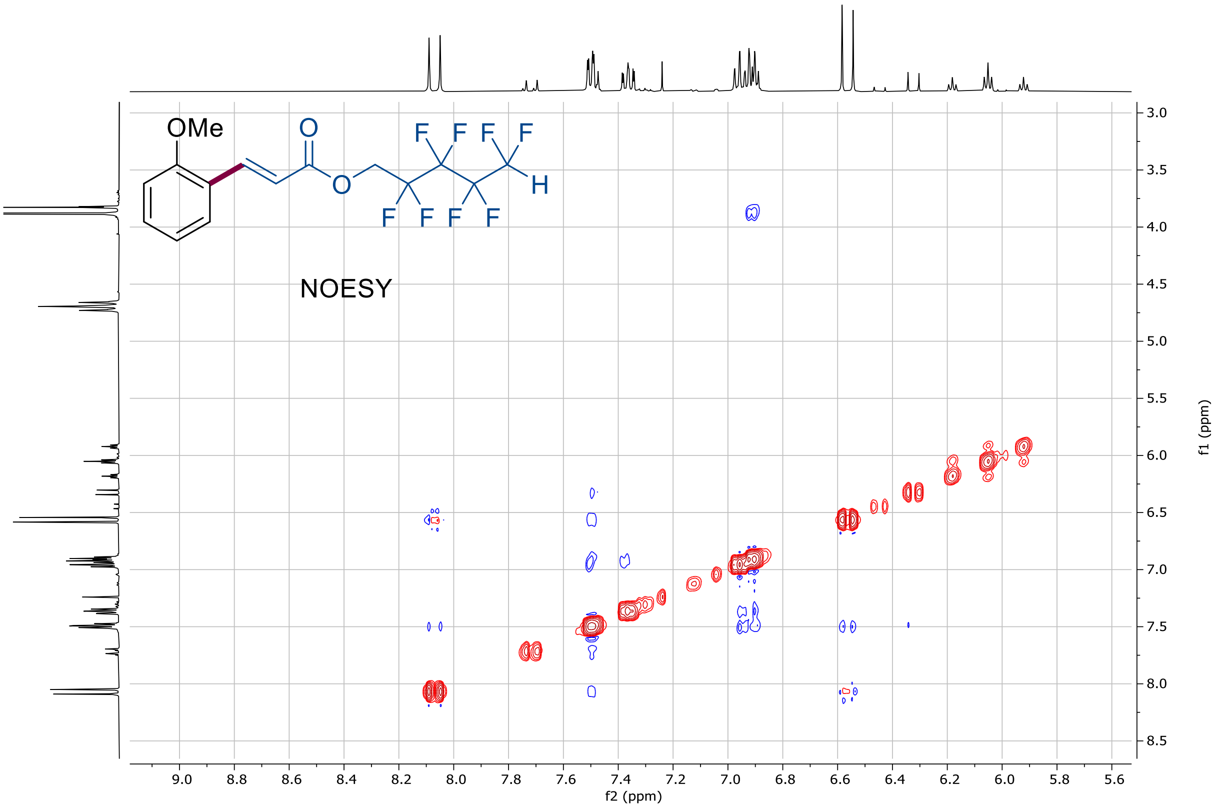

Supplementary Figure 176 NOESY-NMR of compound 50. CDCl₃, RT

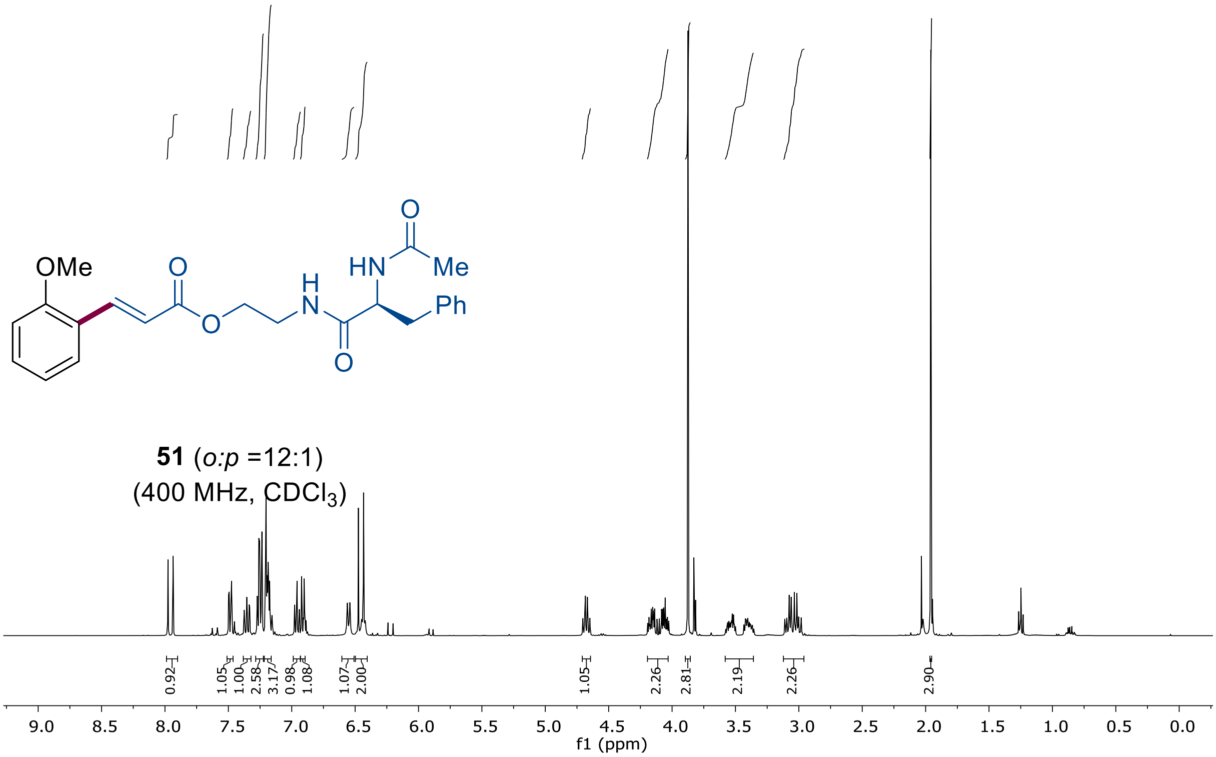

Supplementary Figure 177 H-NMR of compound 51. 400 MHz, $CDCl_3$, RT

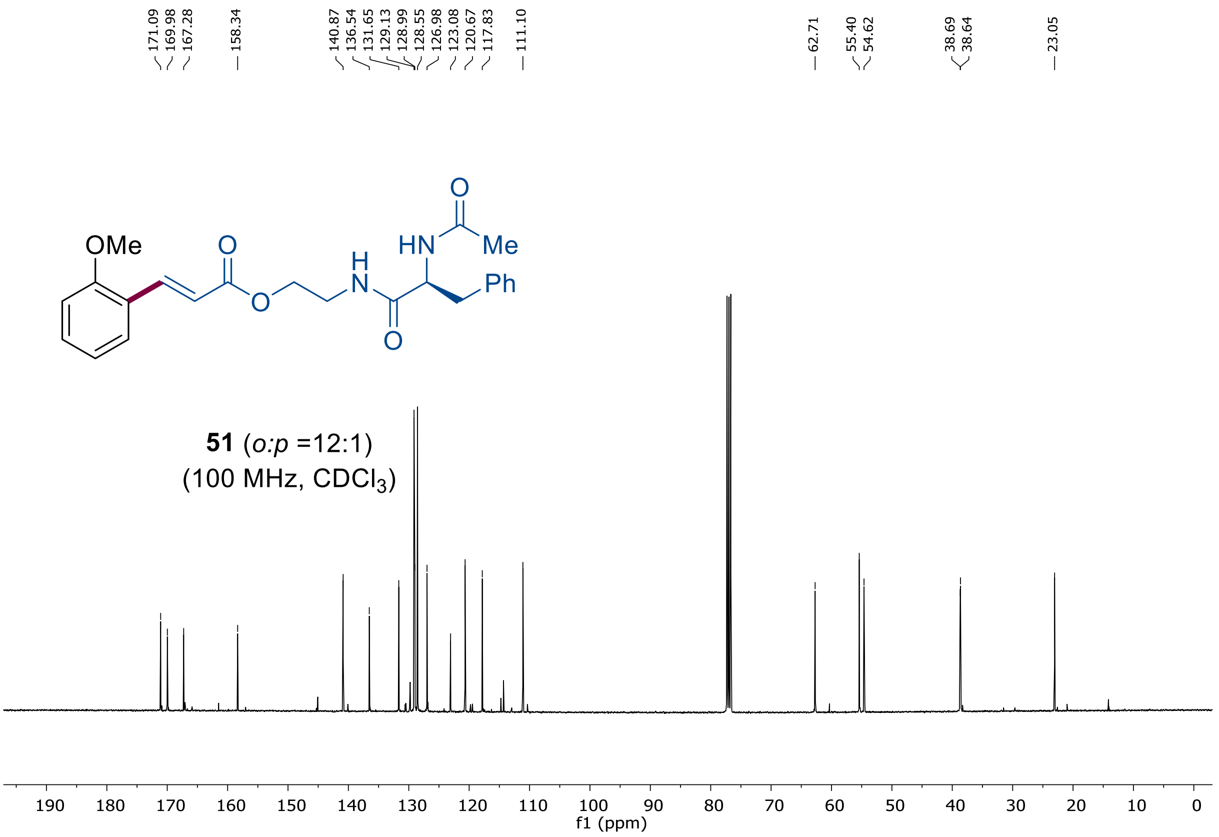

Supplementary Figure 178 C-NMR of compound 51. 100 MHz, CDCl₃, RT

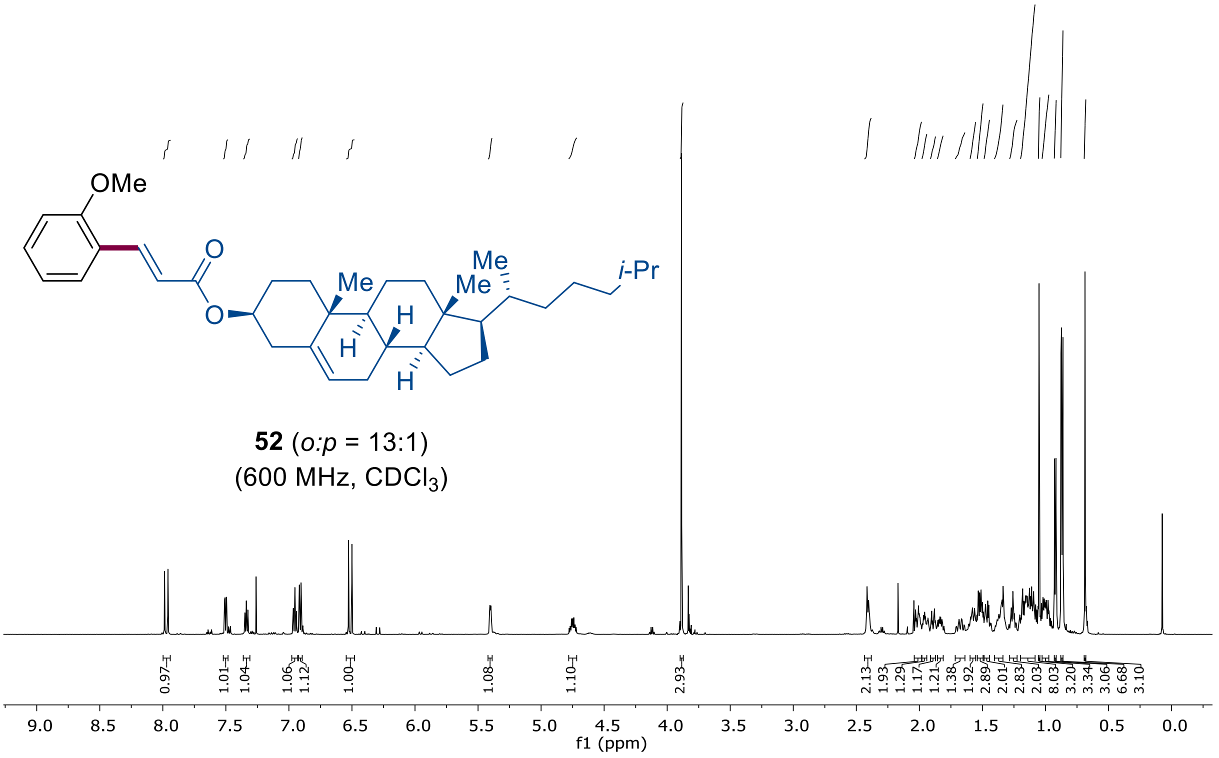

Supplementary Figure 179 H-NMR of compound 52. 600 MHz, CDCl₃, RT

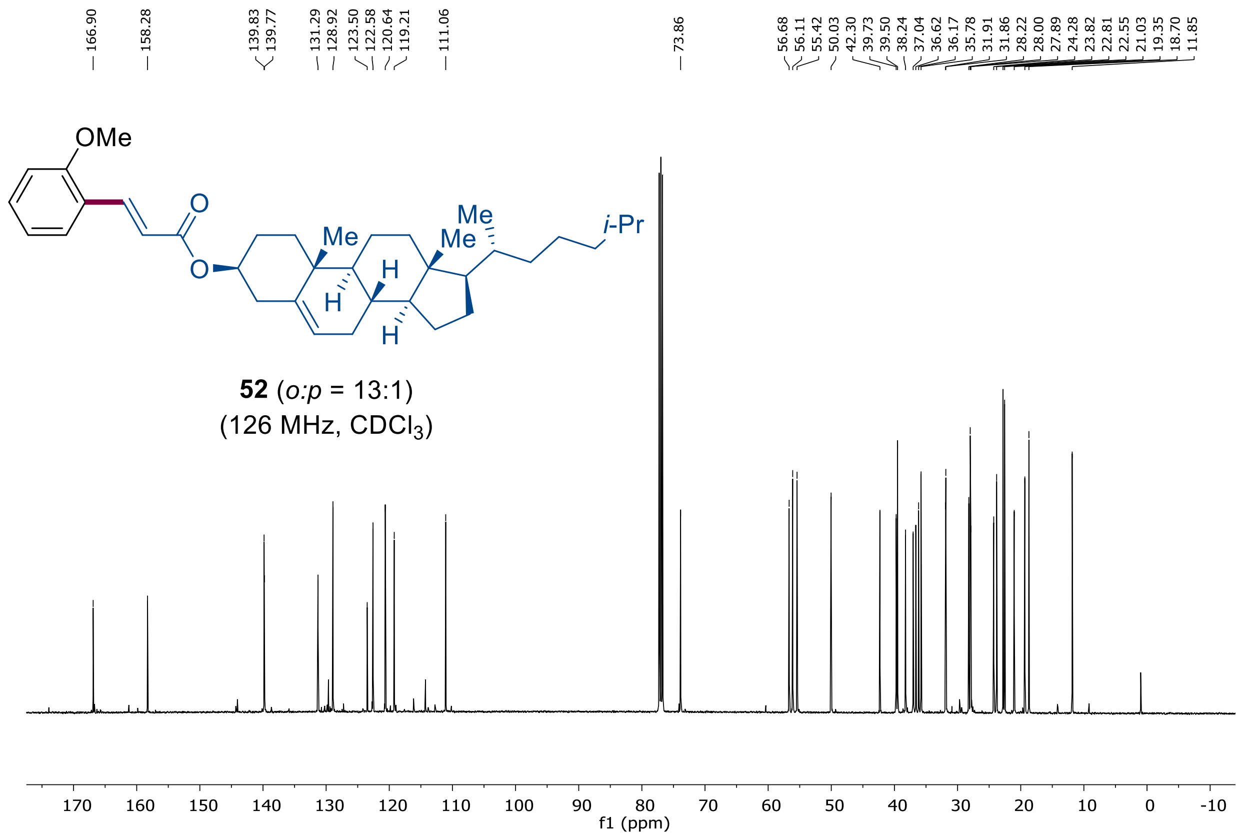

Supplementary Figure 180 C-NMR of compound 52. 126 MHz, $CDCl_3$, RT

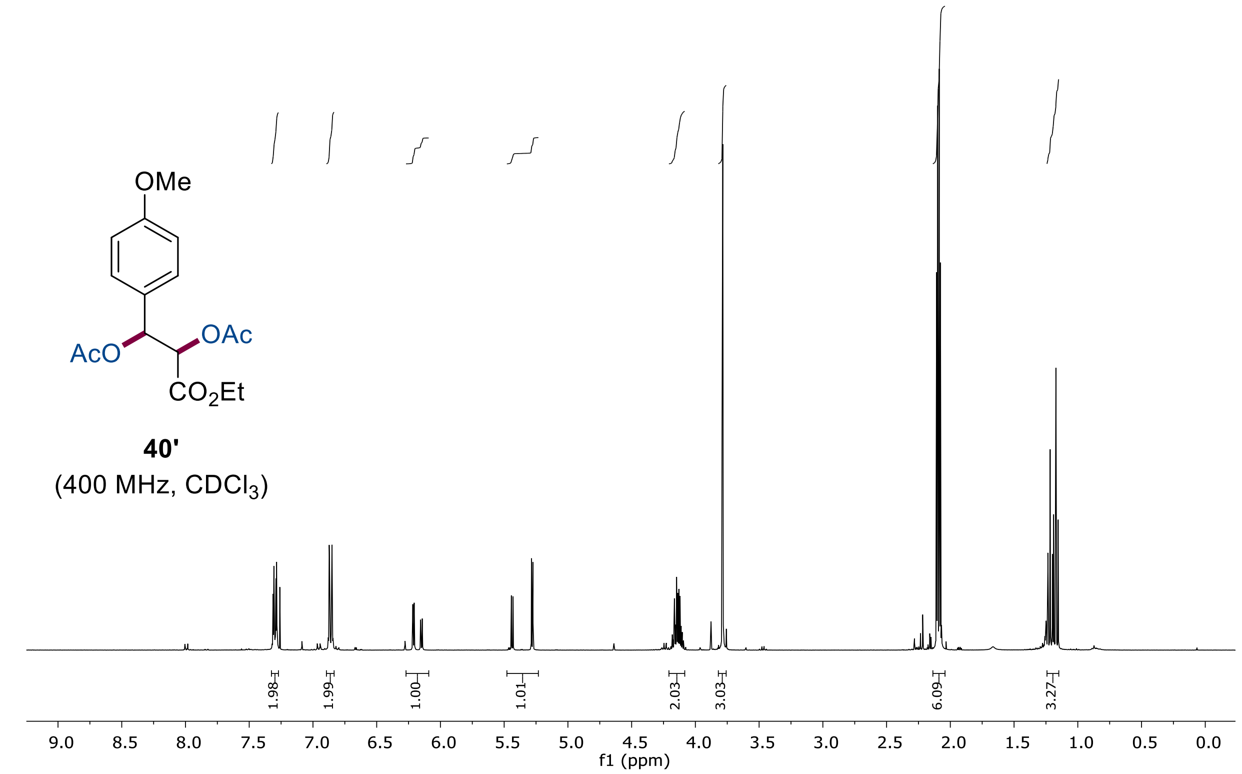

Supplementary Figure 181 H-NMR of compound 40'. 400 MHz, $CDCl_3$, RT

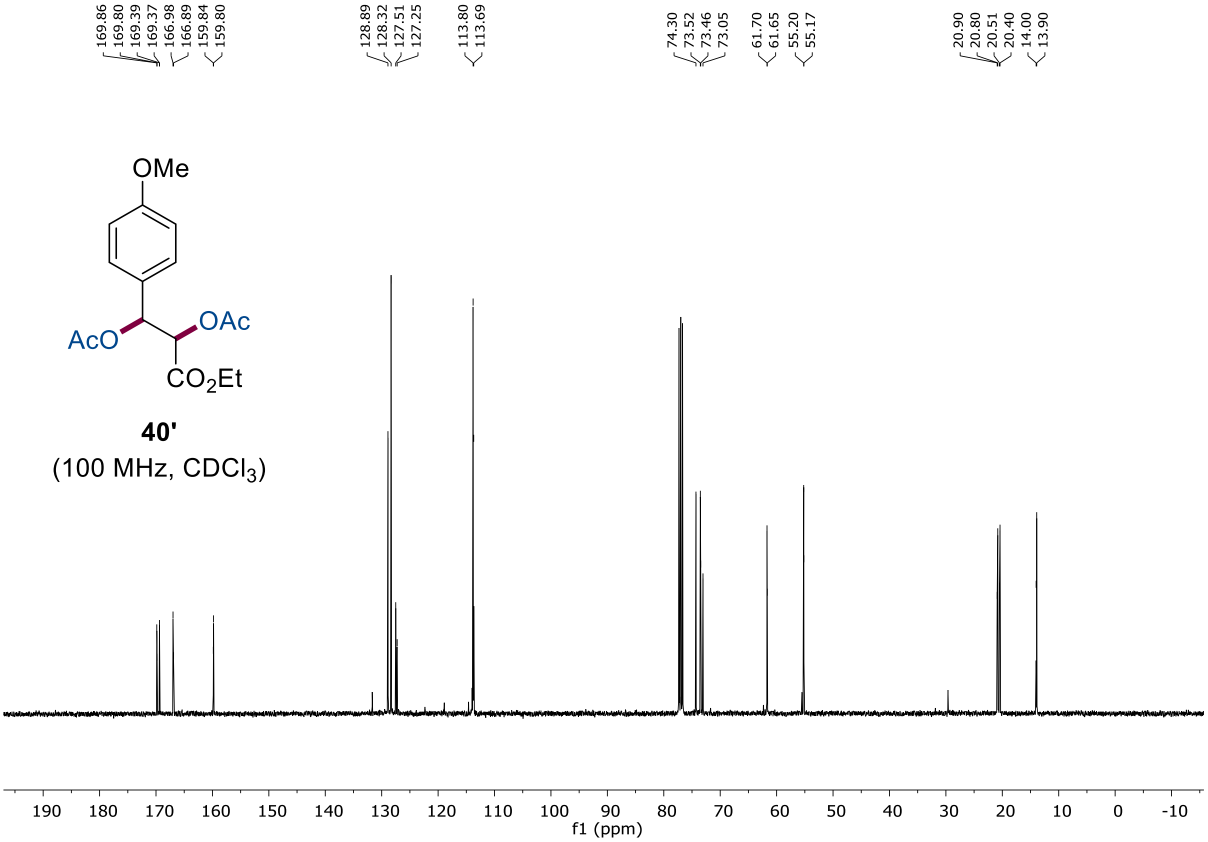

Supplementary Figure 182 C-NMR of compound 40’. 100 MHz, $CDCl_3$, RT

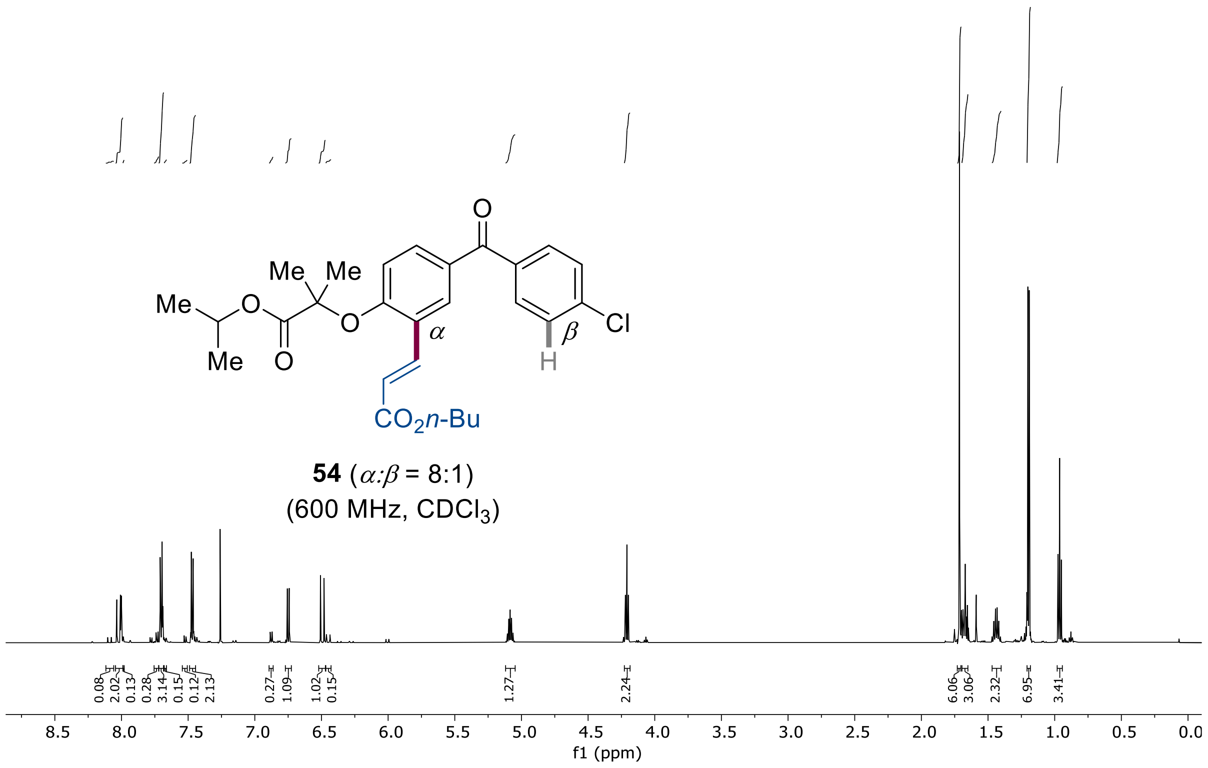

Supplementary Figure 183 H-NMR of compound 54. 600 MHz, $CDCl_3$, RT

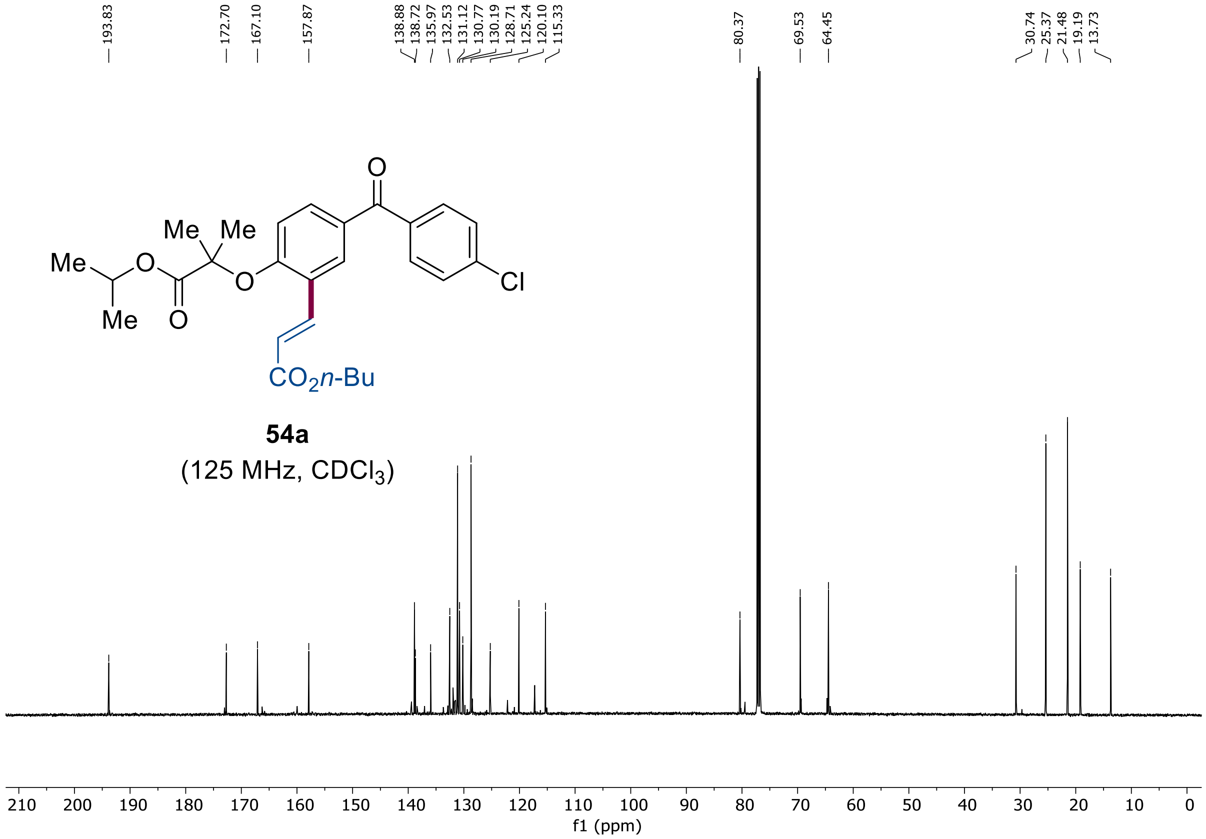

Supplementary Figure 184 C-NMR of compound 54a. 125 MHz, $CDCl_3$, RT

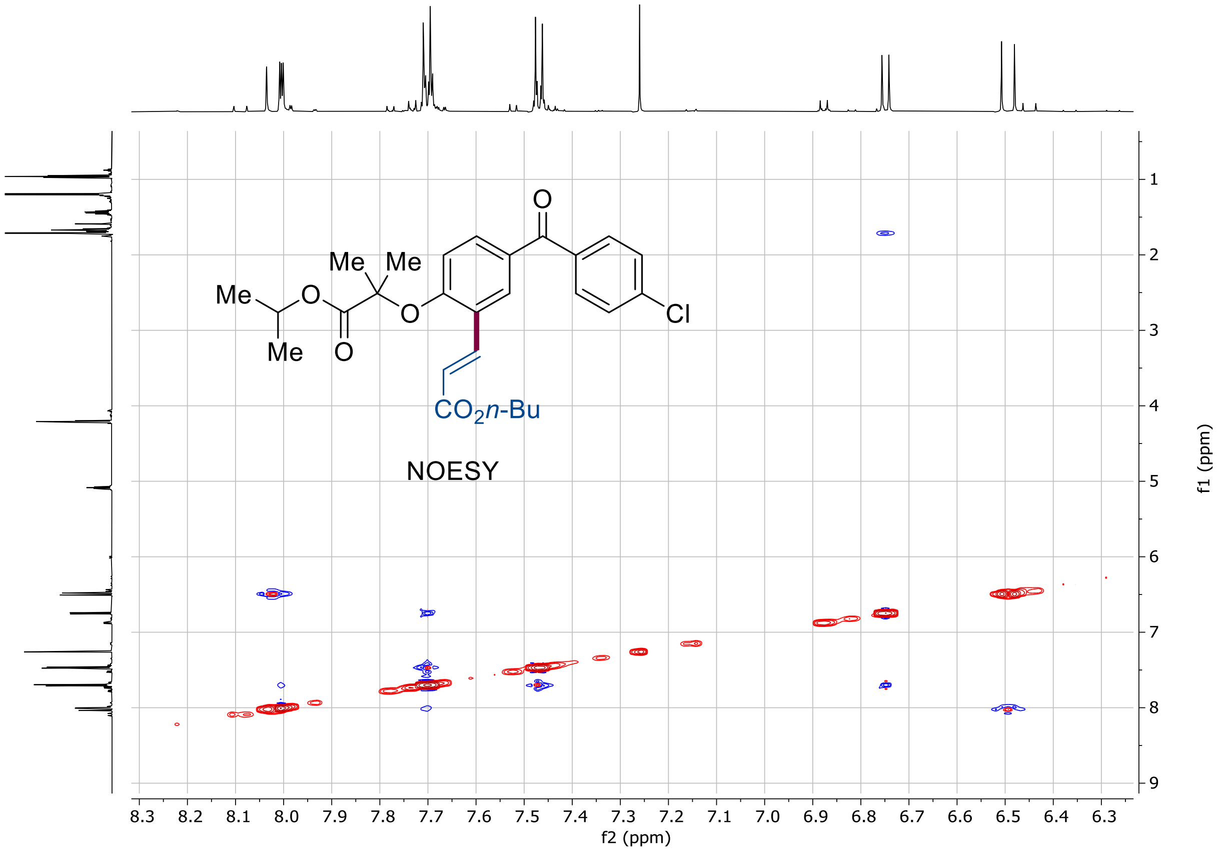

Supplementary Figure 185 NOESY-NMR of compound 54. $CDCl_3$, RT

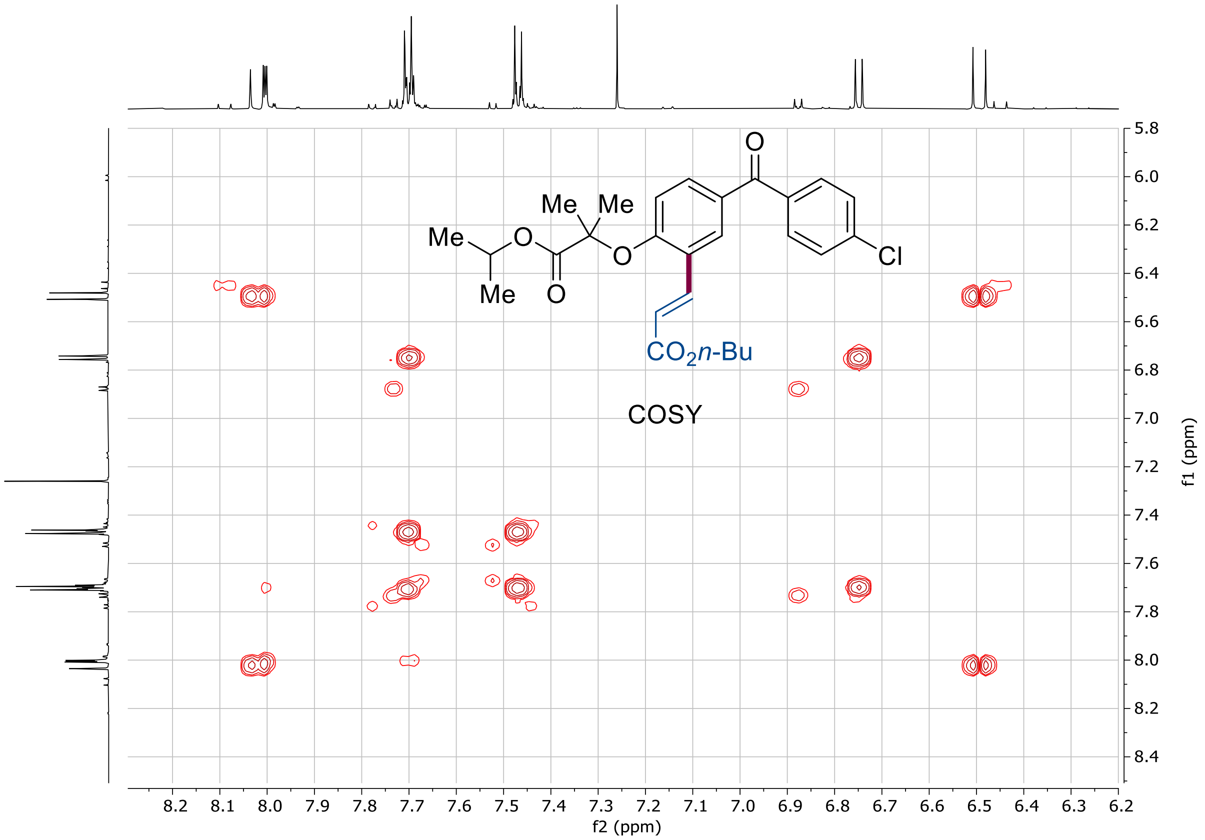

Supplementary Figure 186 COSY-NMR of compound 54. $CDCl_3$, RT

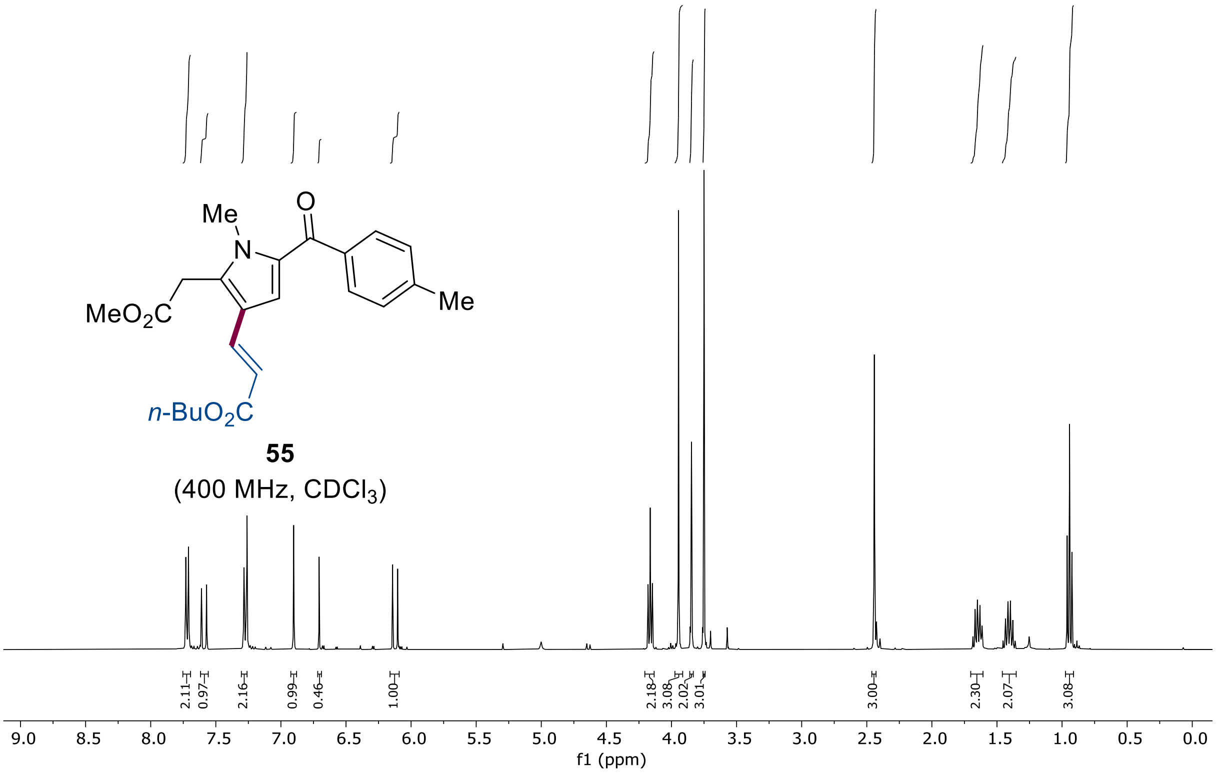

Supplementary Figure 187 H-NMR of compound 55. 400 MHz, $CDCl_3$, RT

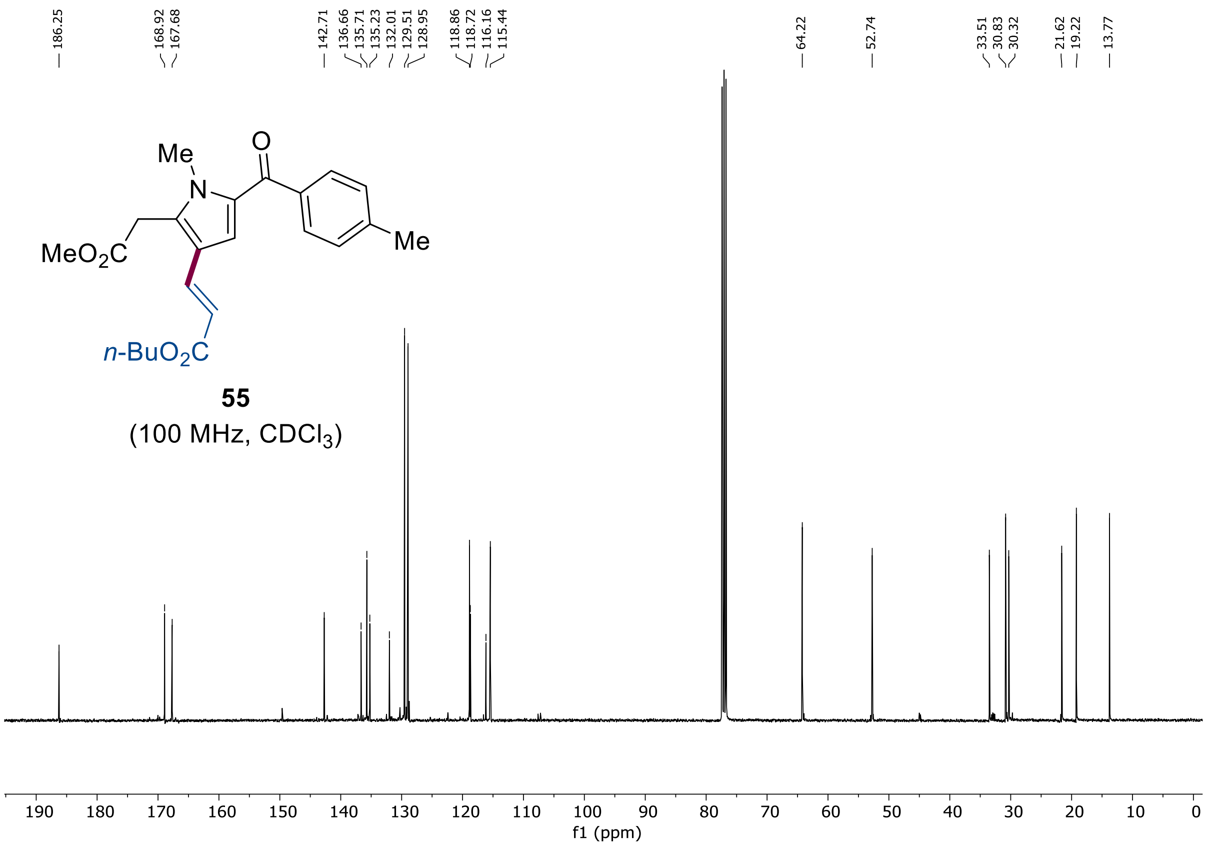

Supplementary Figure 188 C-NMR of compound 55. 100 MHz, $CDCl_3$, RT

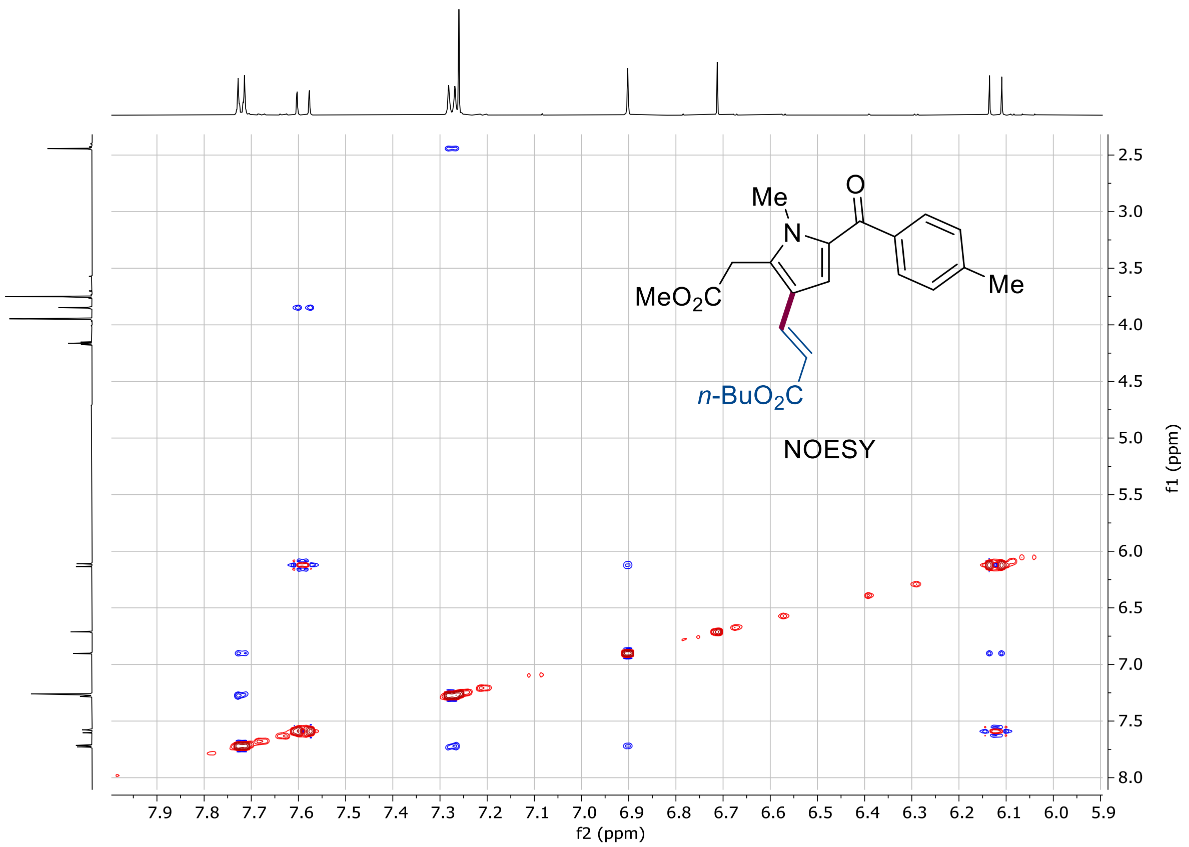

Supplementary Figure 189 NOESY-NMR of compound 55. $CDCl_3$, RT

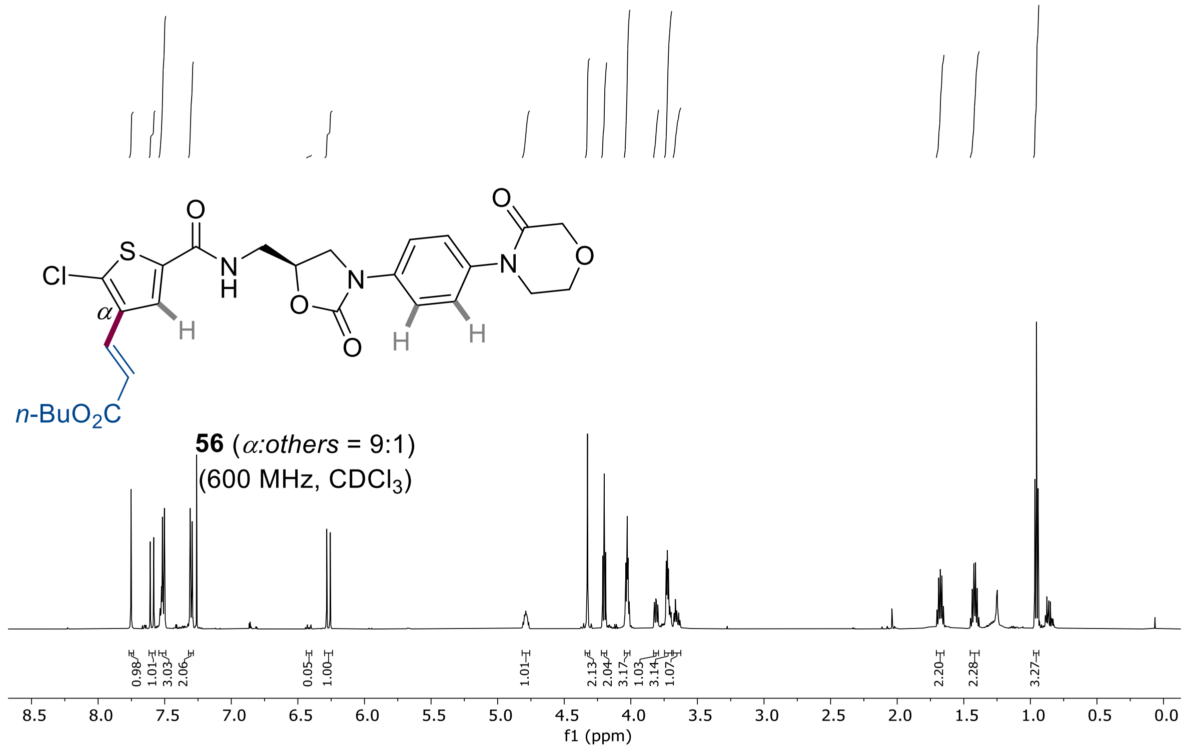

Supplementary Figure 190 H-NMR of compound 56. 600 MHz, $CDCl_3$, RT

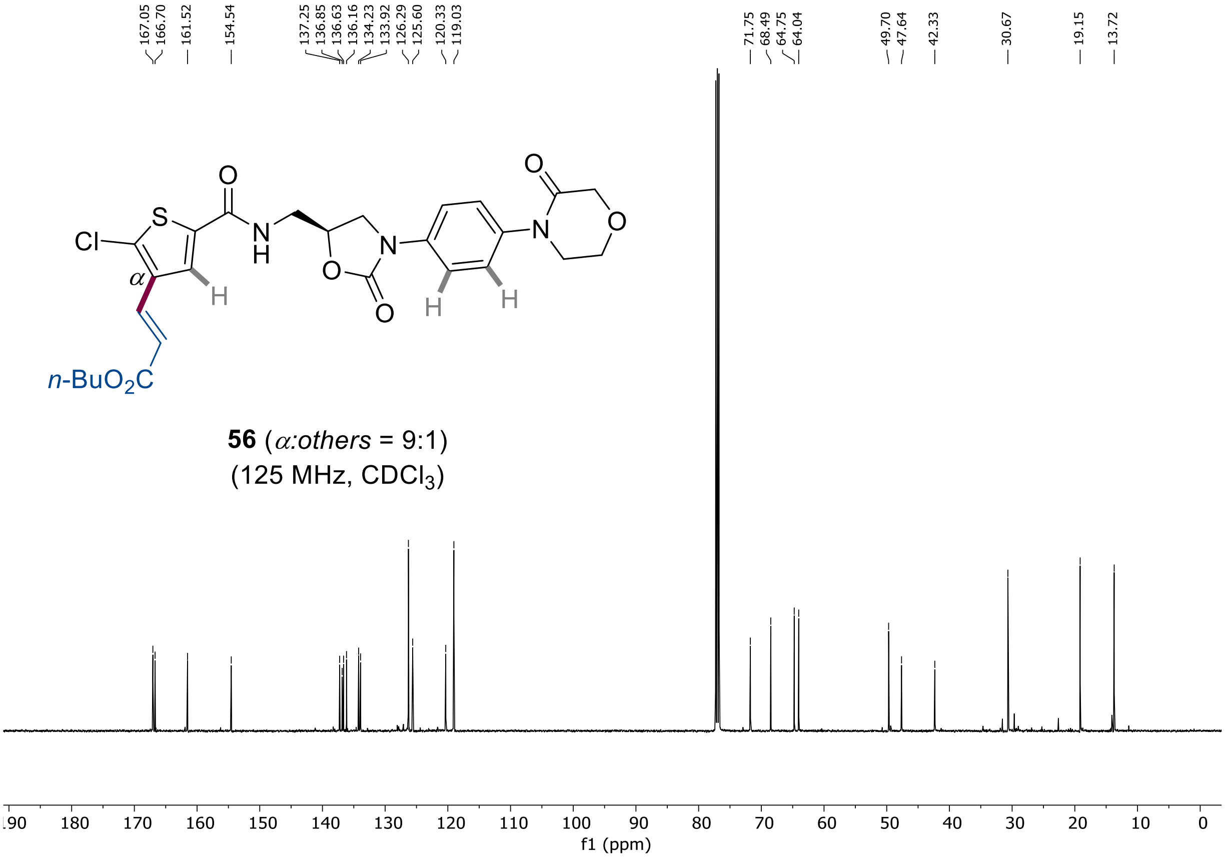

Supplementary Figure 191 C-NMR of compound 56. 125 MHz, CDCl₃, RT

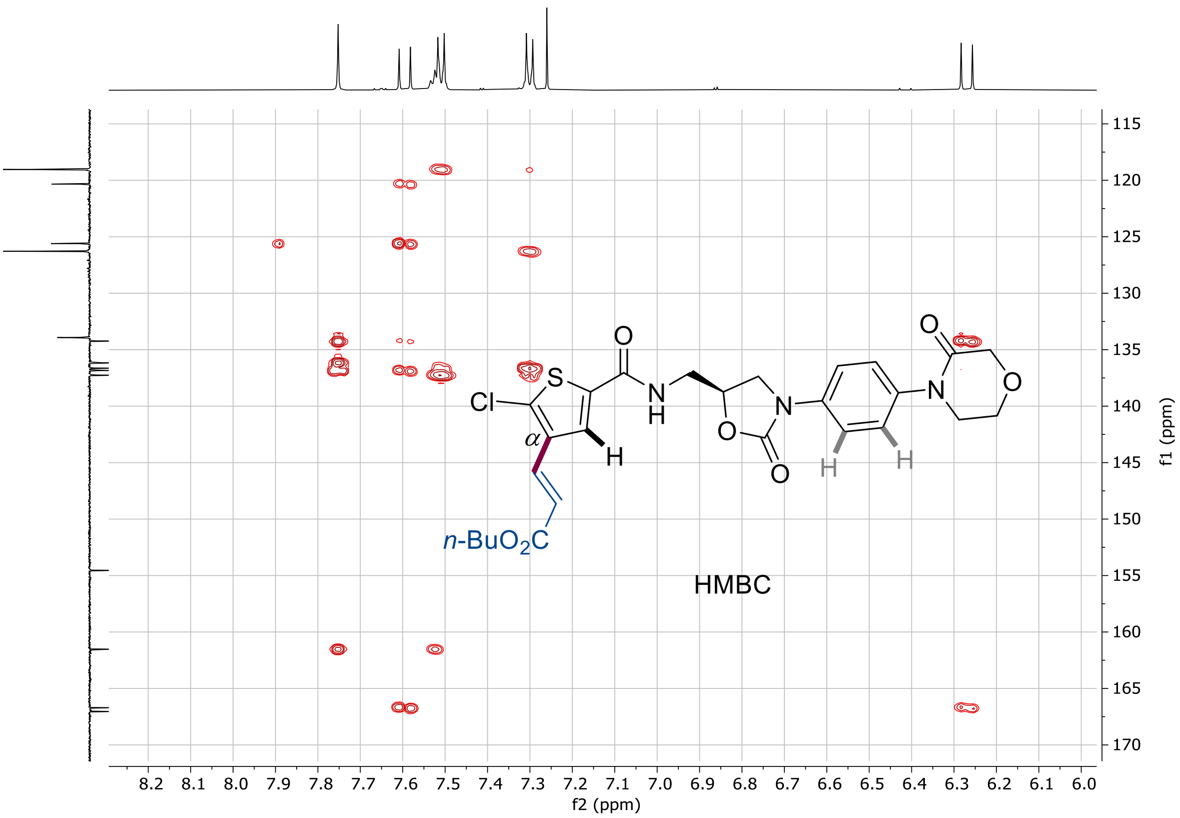

Supplementary Figure 192 HMBC-NMR of compound 56. CDCl₃, RT

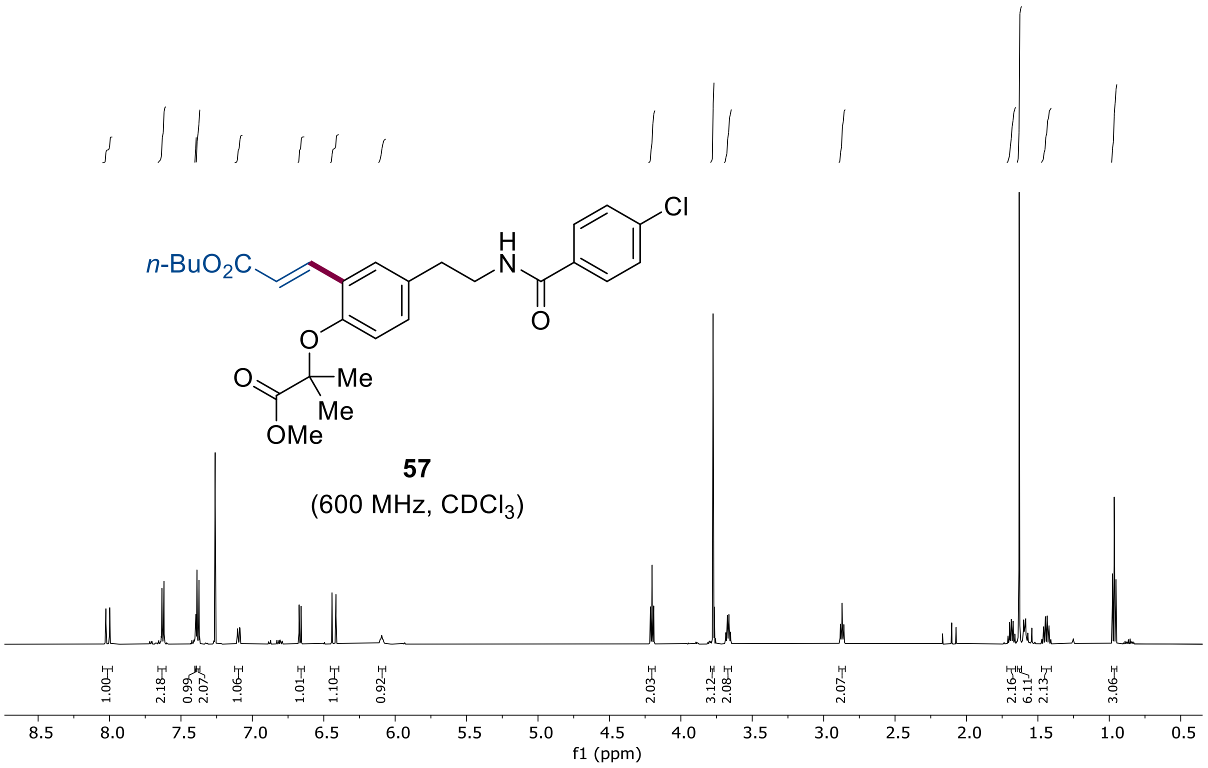

Supplementary Figure 193 H-NMR of compound 57. 600 MHz, $CDCl_3$, RT

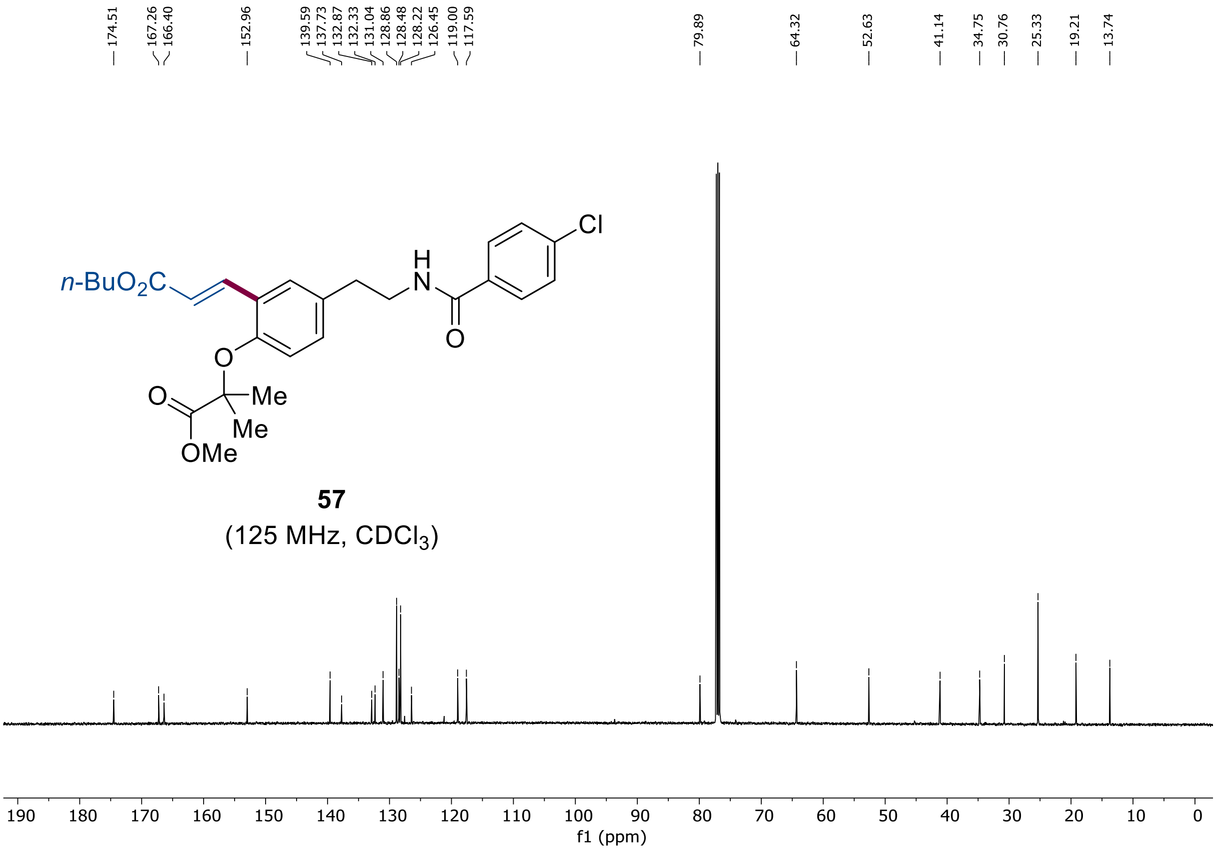

Supplementary Figure 194 C-NMR of compound 57. 125 MHz, $CDCl_3$, RT

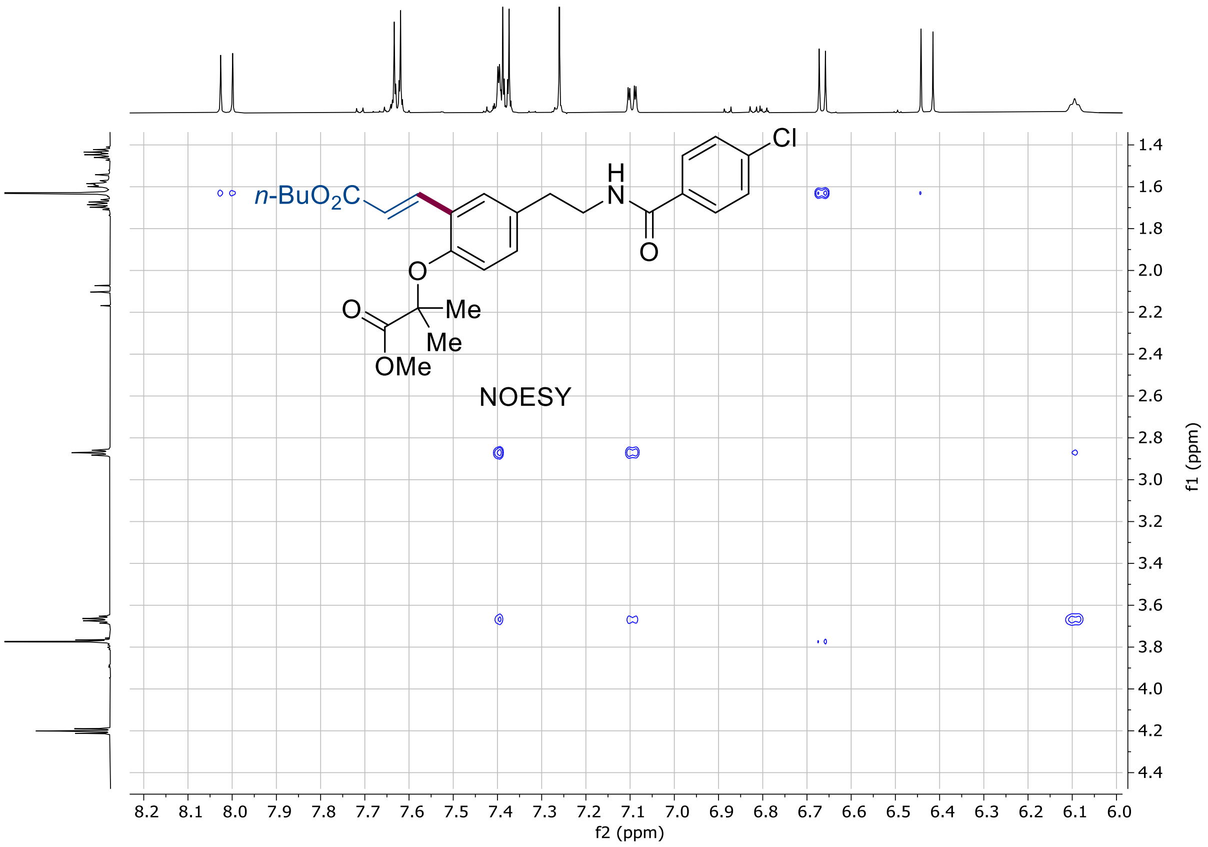

Supplementary Figure 195 NOESY-NMR of compound 57. CDCl₃, RT

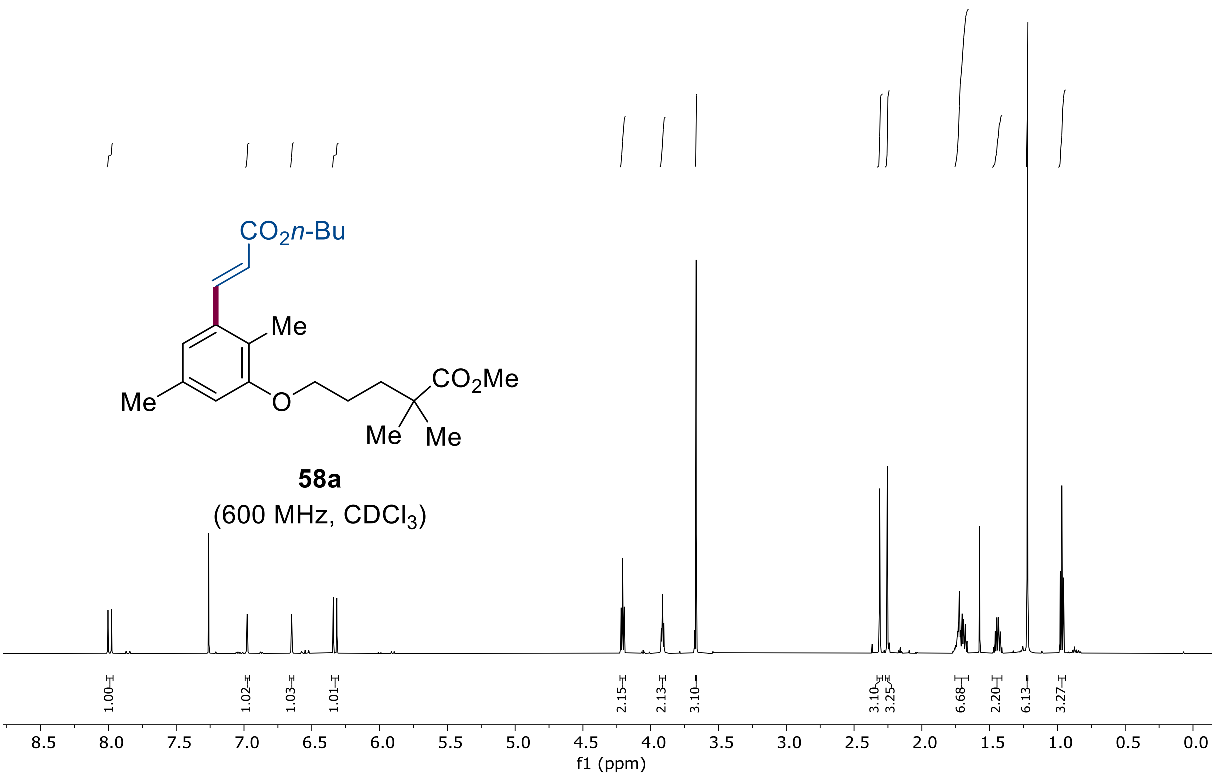

Supplementary Figure 196 H-NMR of compound 58a. 600 MHz, CDCl₃, RT

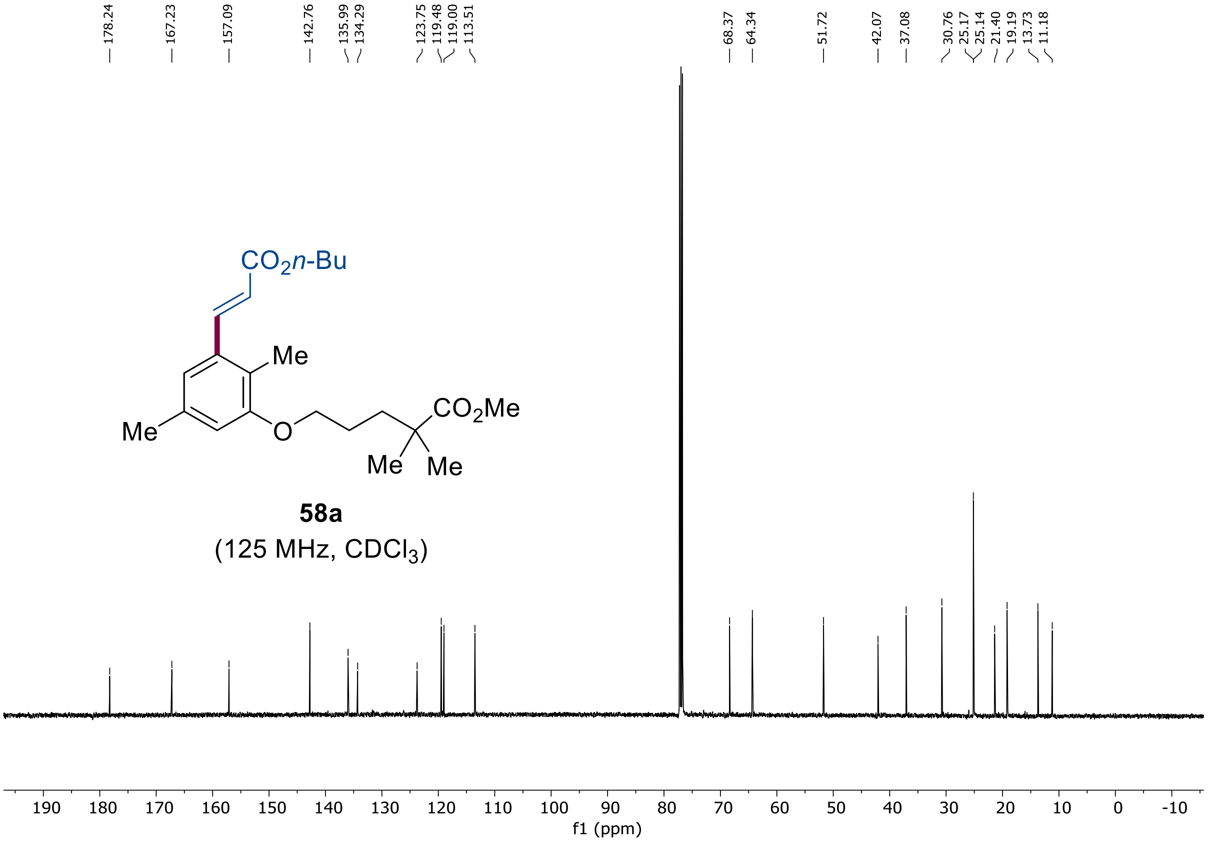

Supplementary Figure 197 C-NMR of compound 58a. 125 MHz, $CDCl_3$, RT

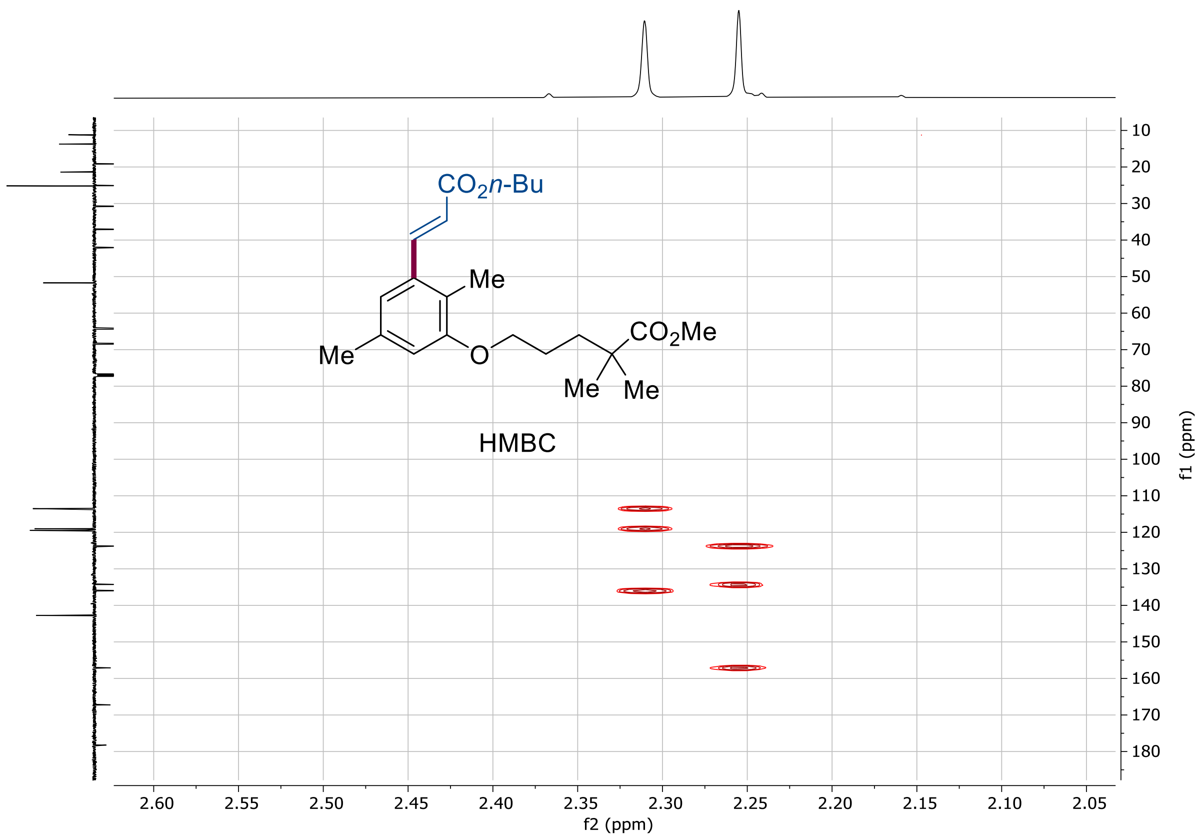

Supplementary Figure 198 HMBC-NMR of compound 58a. $CDCl_3$, RT

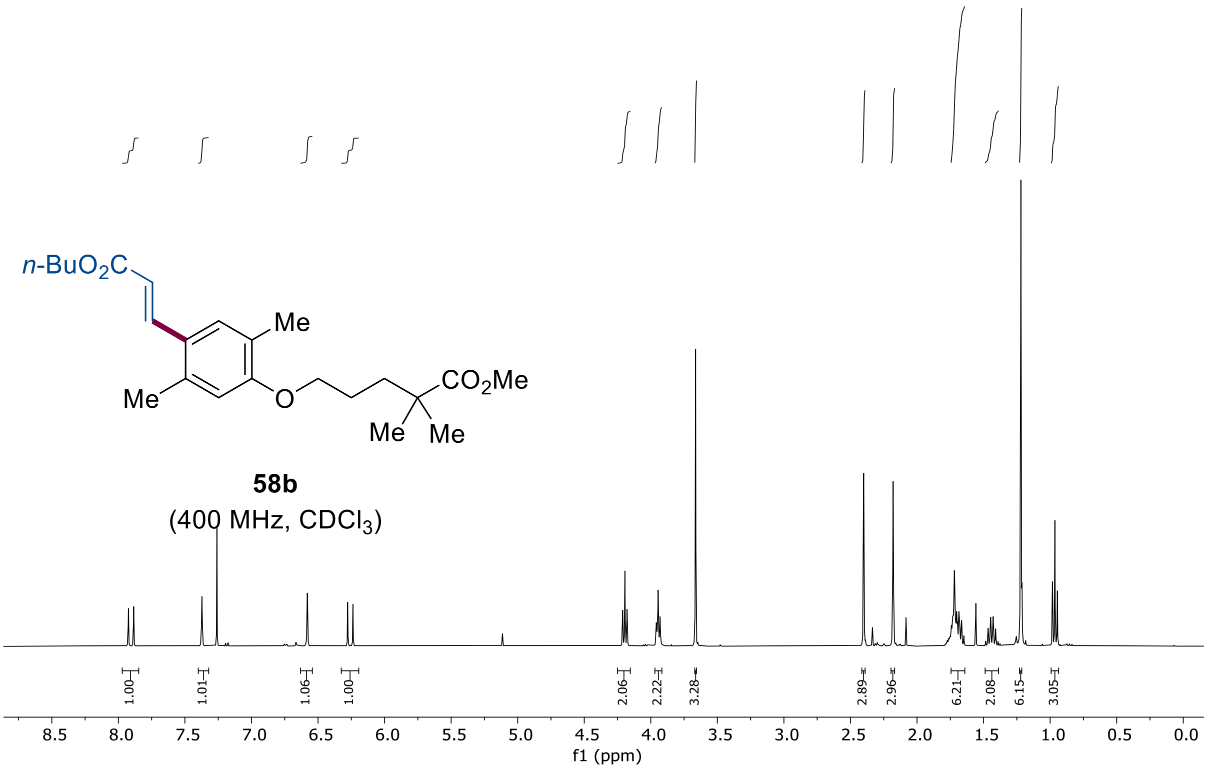

Supplementary Figure 199 H-NMR of compound 58b. 400 MHz, $CDCl_3$, RT

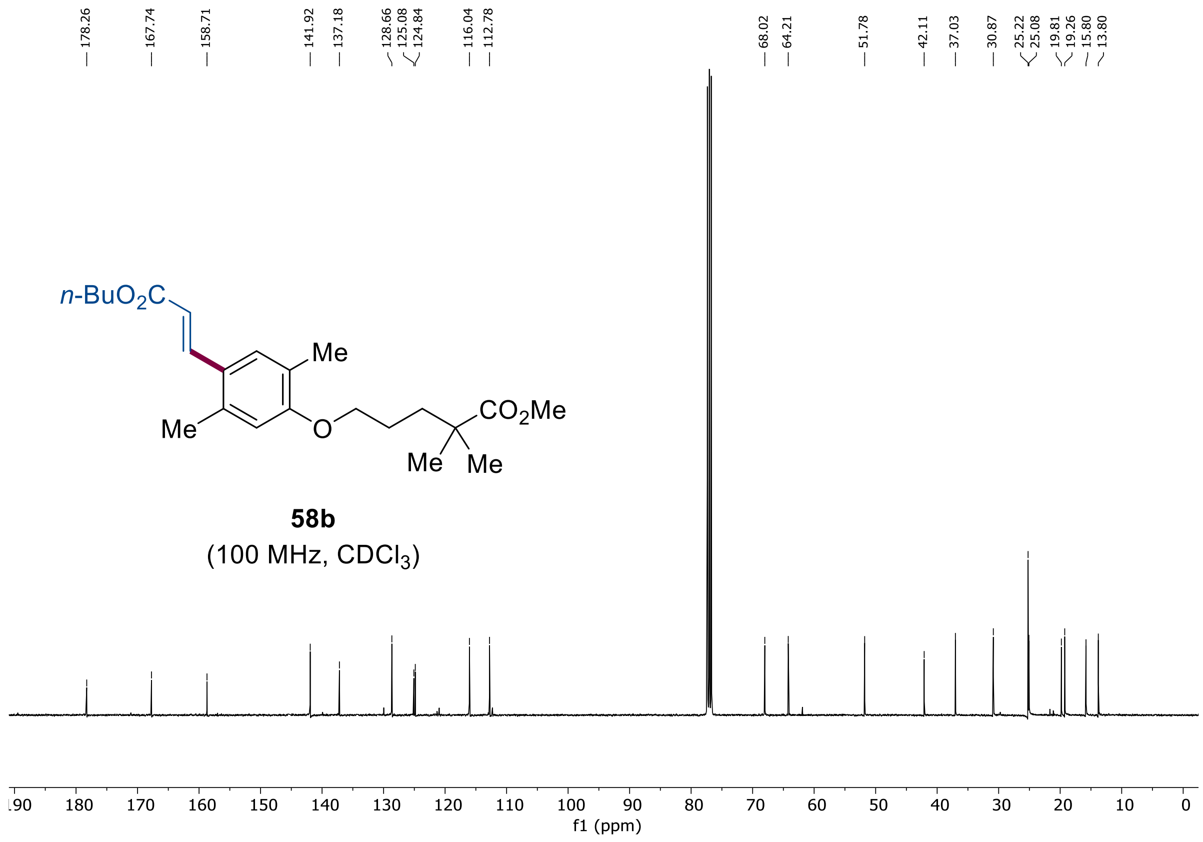

Supplementary Figure 200 C-NMR of compound 58b. 100 MHz, $CDCl_3$, RT

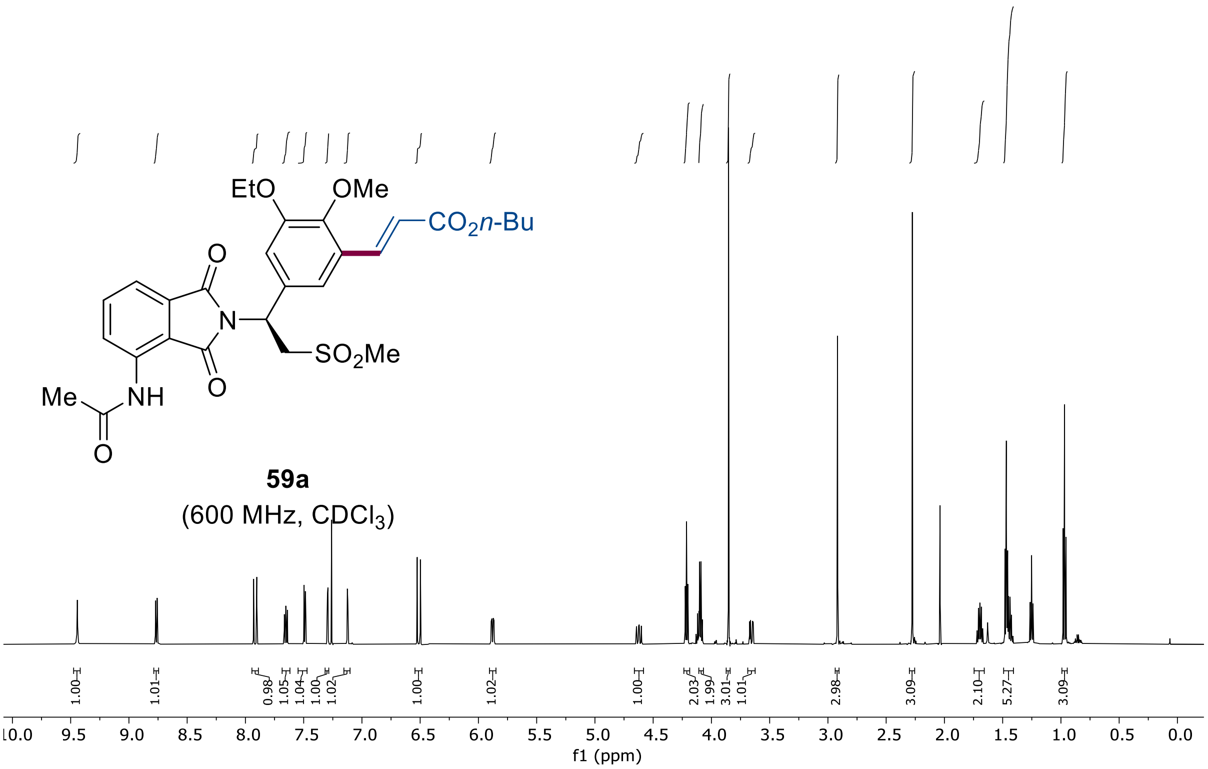

Supplementary Figure 201 H-NMR of compound 59a. 600 MHz, CDCl₃, RT

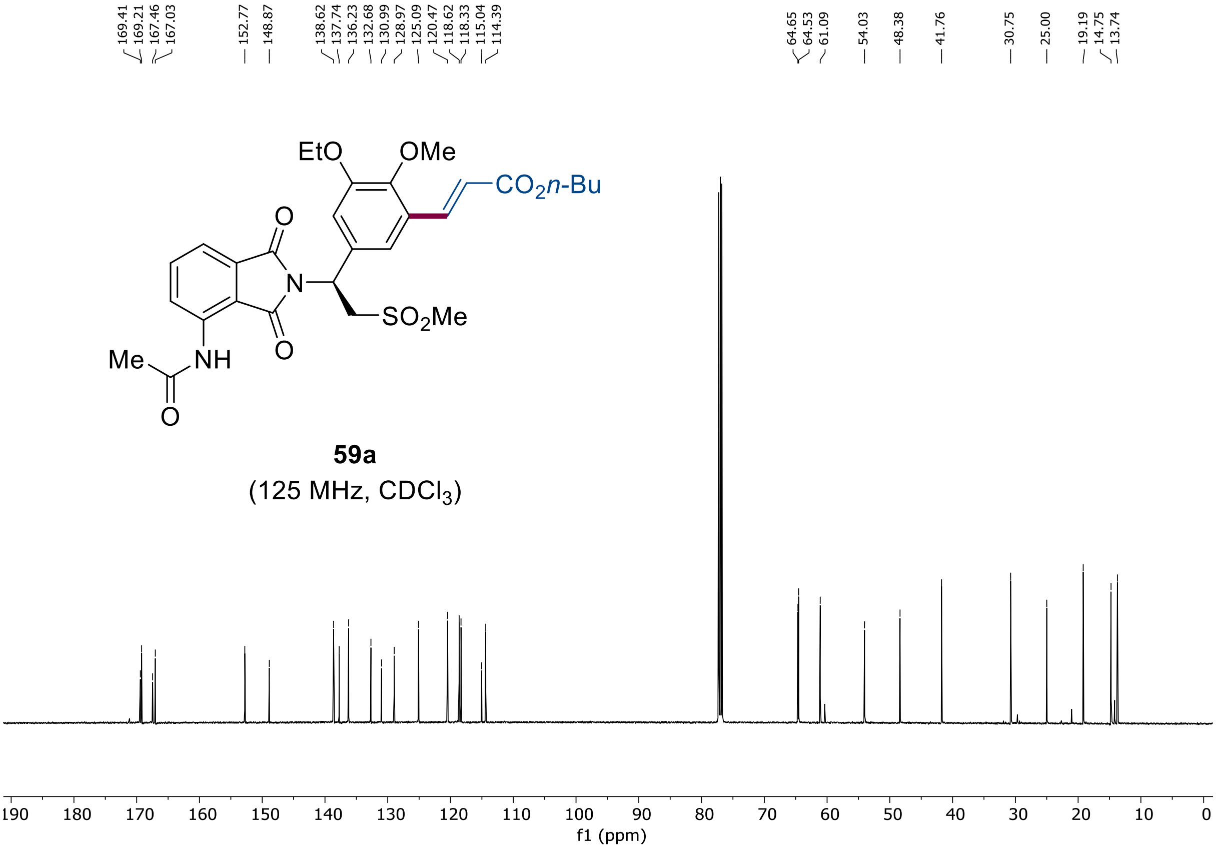

Supplementary Figure 202 C-NMR of compound 59a. 125 MHz, CDCl₃, RT

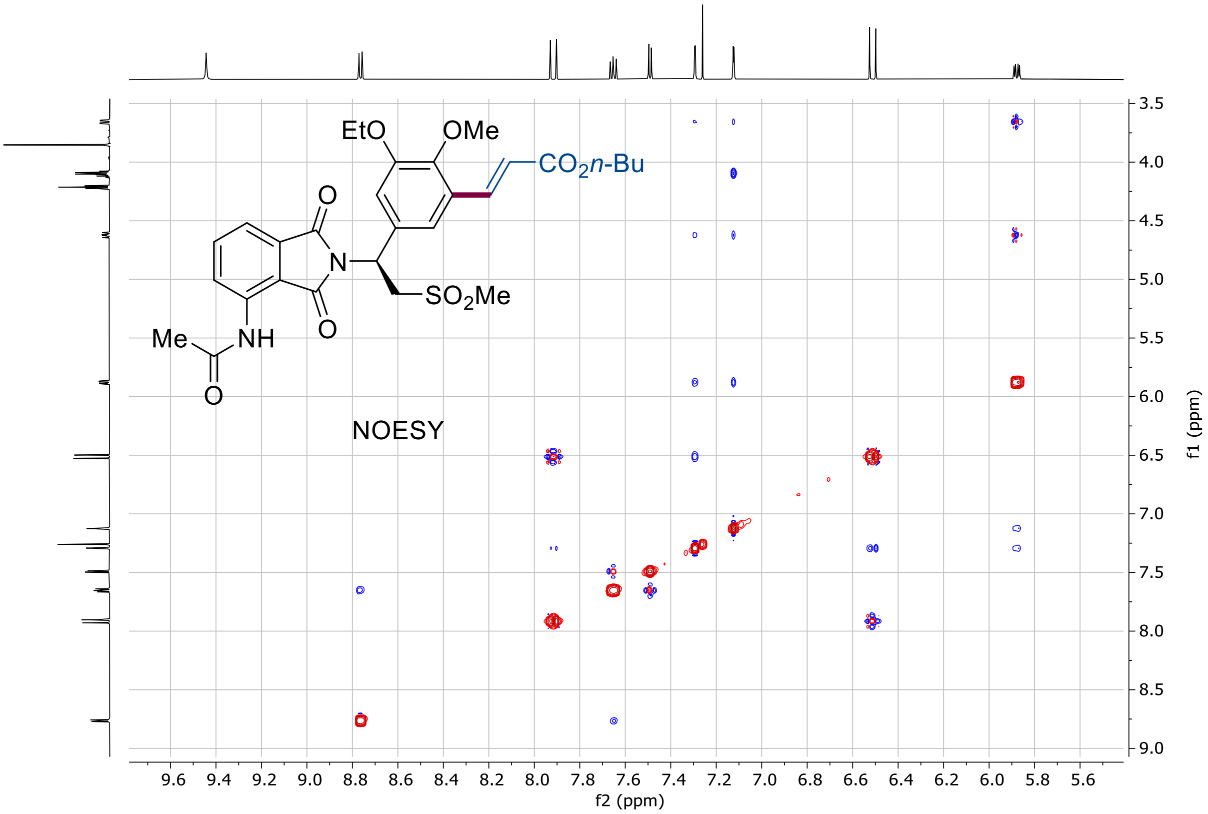

Supplementary Figure 203 NOESY-NMR of compound 59a. CDCl₃, RT

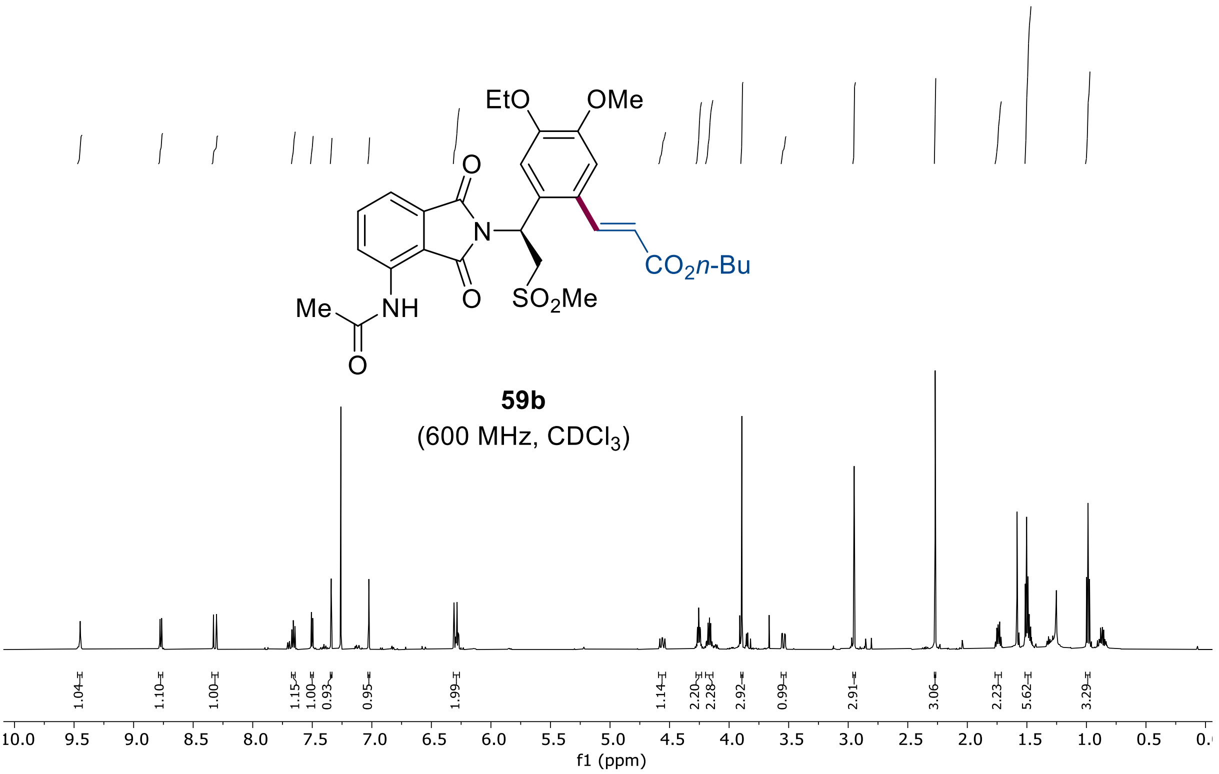

Supplementary Figure 204 H-NMR of compound 59b. 600 MHz, CDCl₃, RT

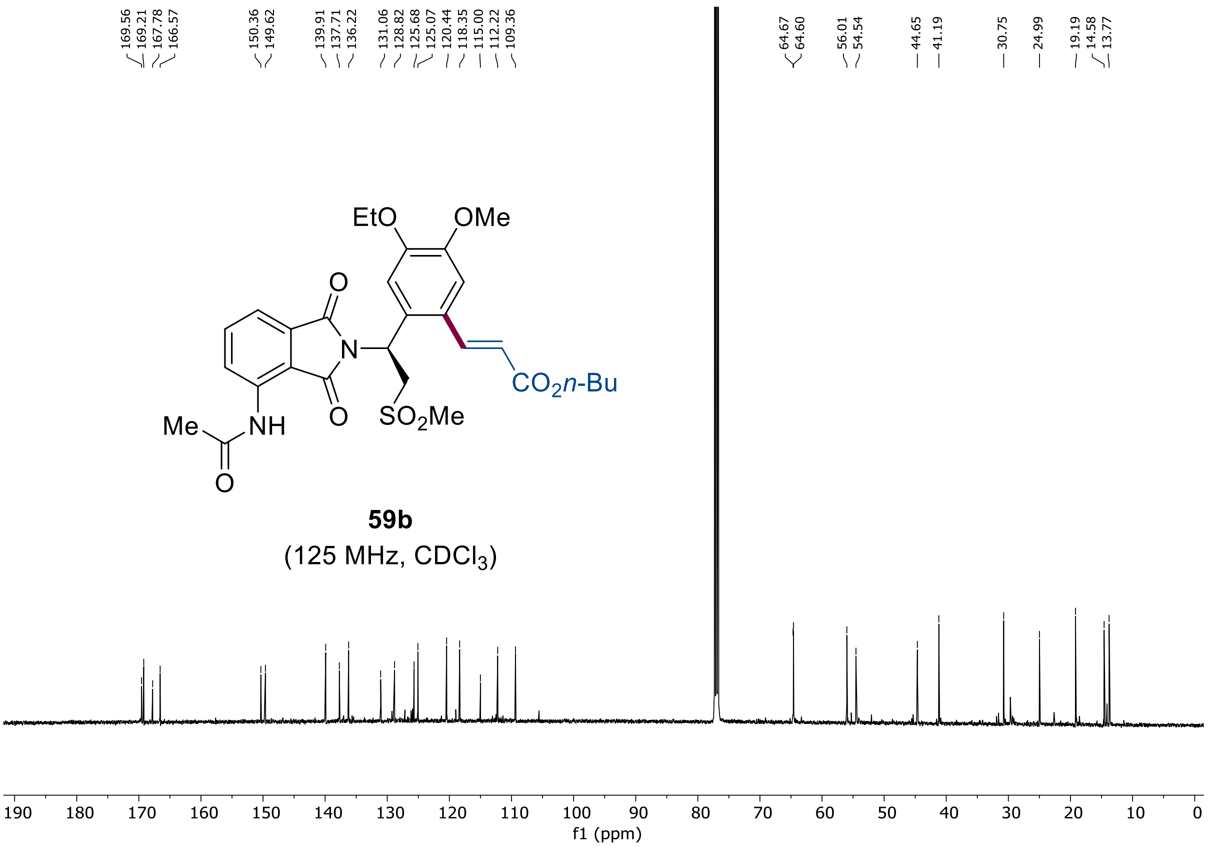

Supplementary Figure 205 C-NMR of compound 59b. 125 MHz, $CDCl_3$, RT

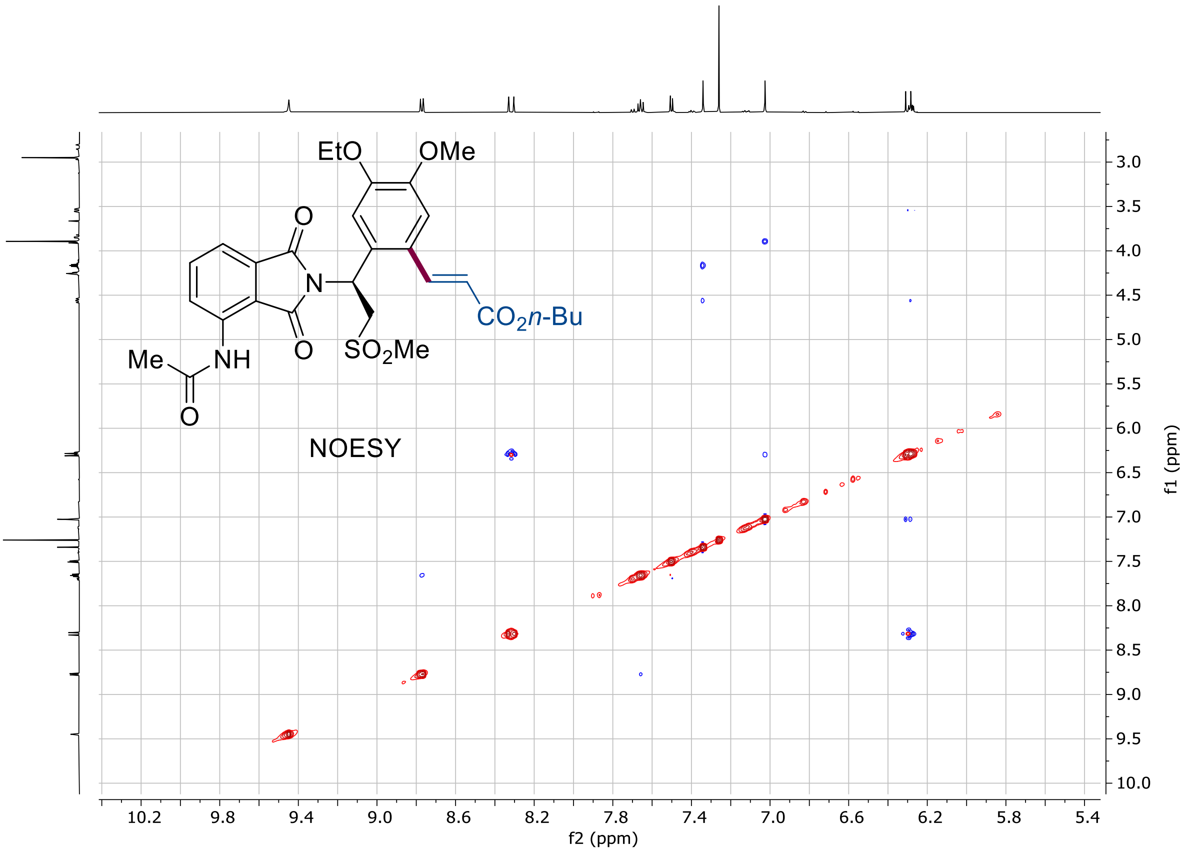

Supplementary Figure 206 NOESY-NMR of compound 59b. $CDCl_3$, RT

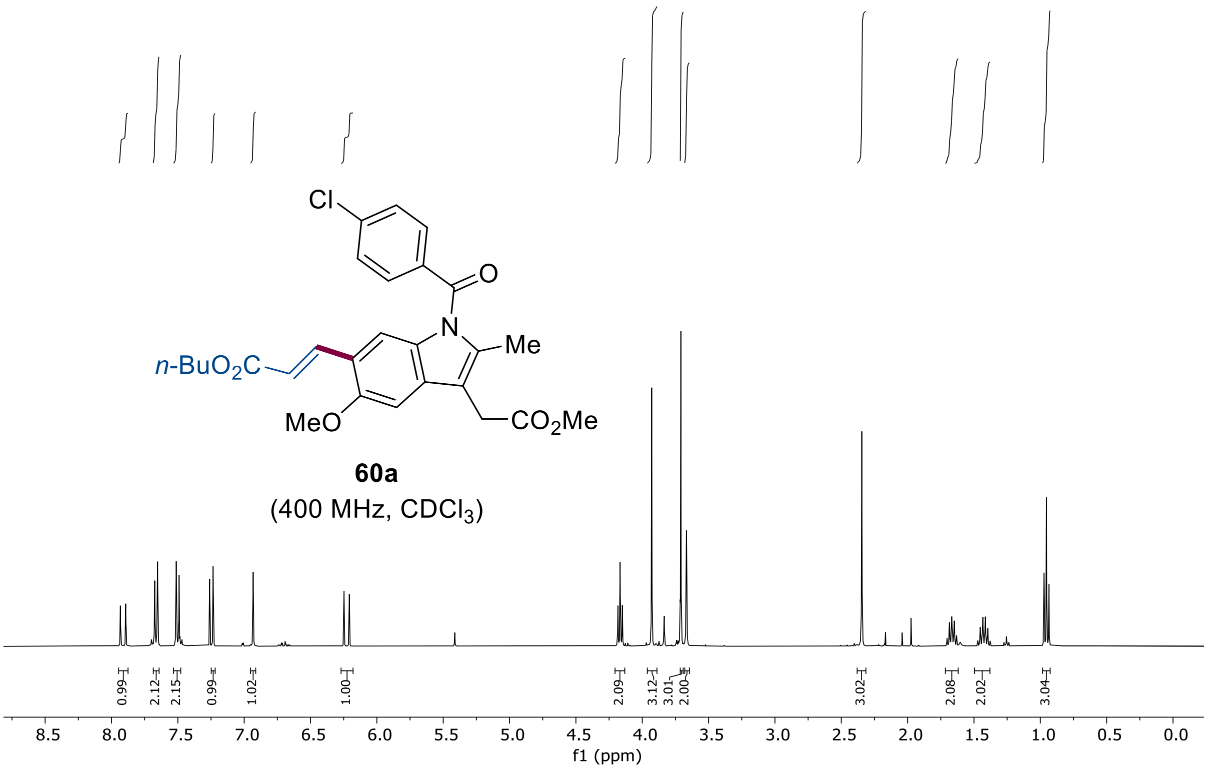

Supplementary Figure 207 H-NMR of compound 60a. 400 MHz, CDCl₃, RT

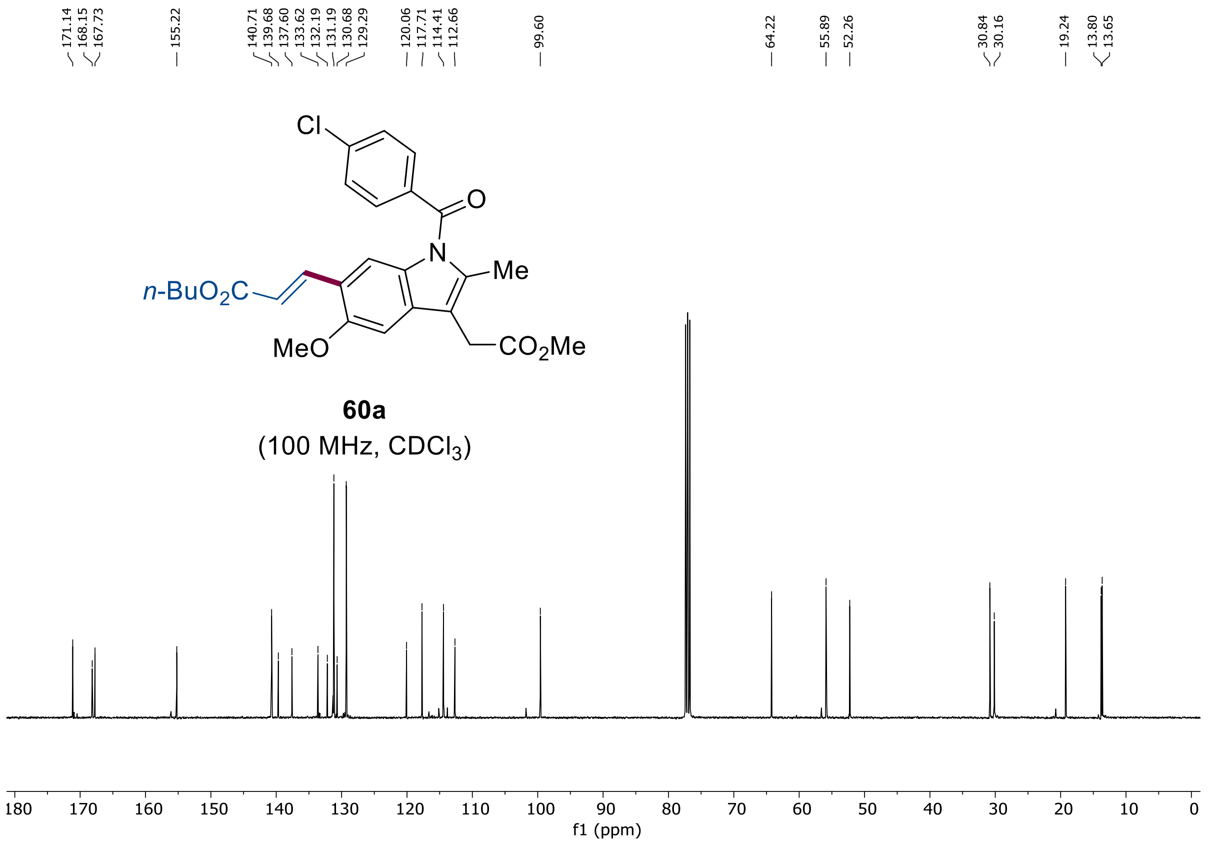

Supplementary Figure 208 C-NMR of compound 60a. 100 MHz, CDCl₃, RT

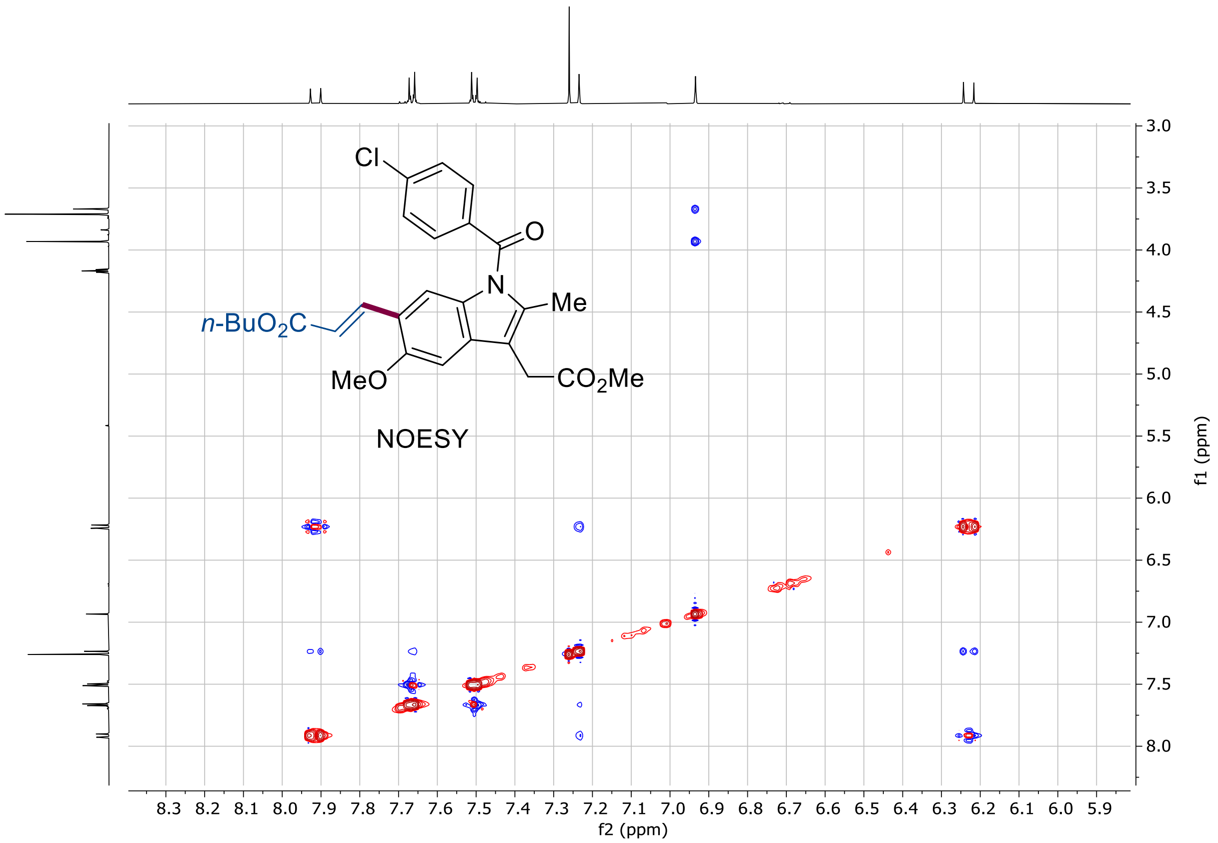

Supplementary Figure 209 NOESY-NMR of compound 60a. CDCl₃, RT

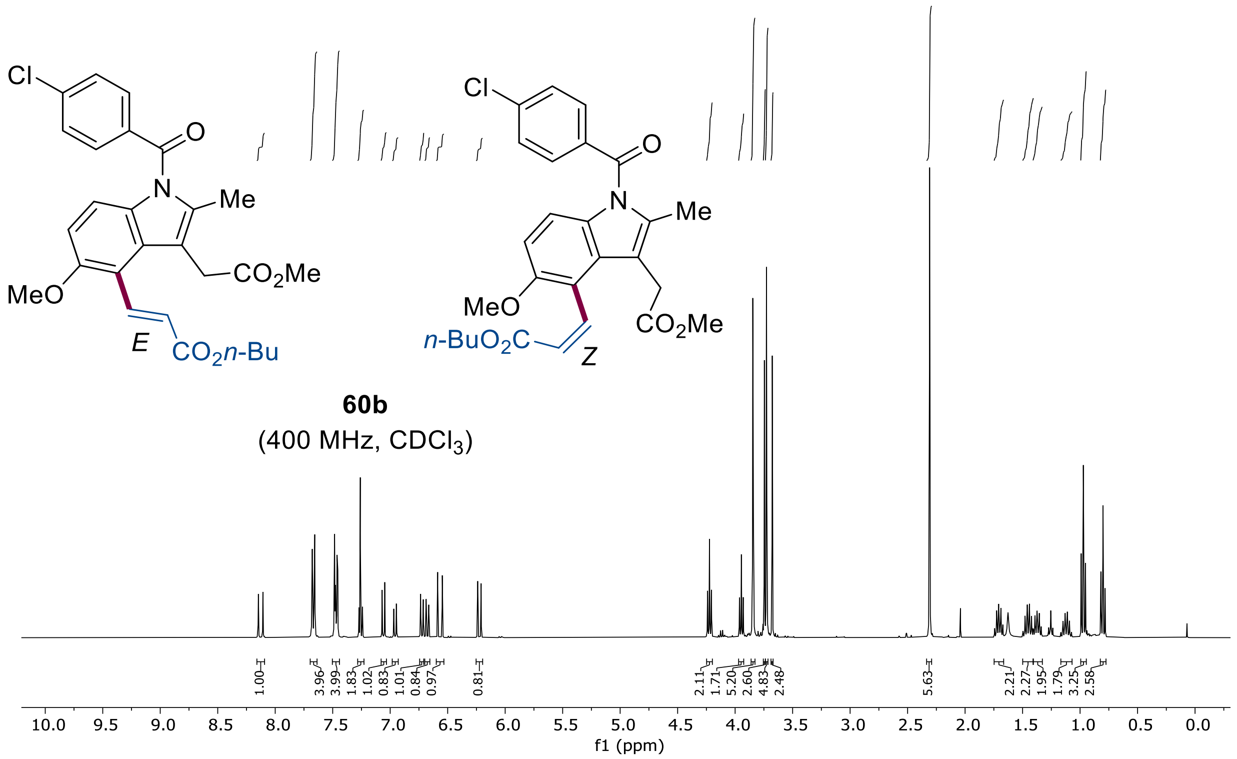

Supplementary Figure 210 H-NMR of compound 60b. 400 MHz, CDCl₃, RT

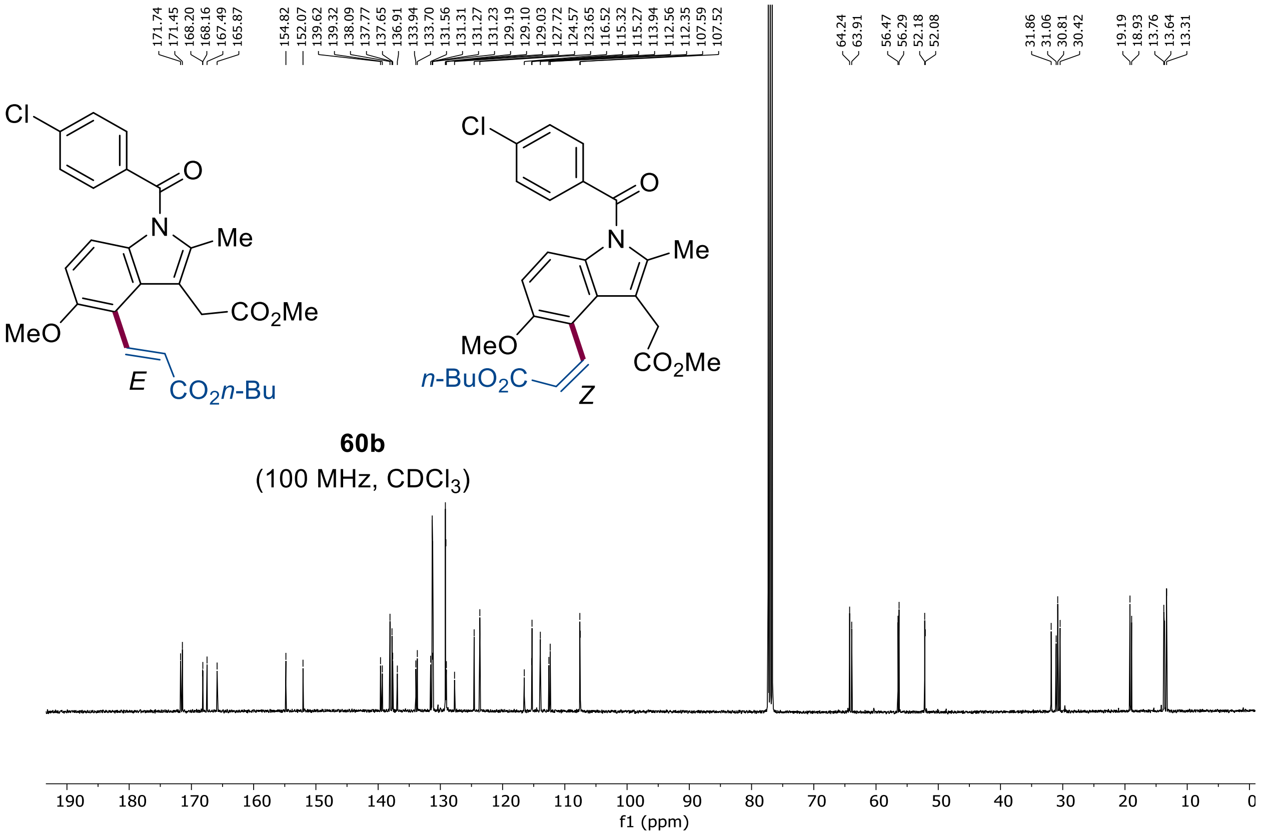

Supplementary Figure 211 C-NMR of compound 60b. 100 MHz, $CDCl_3$, RT

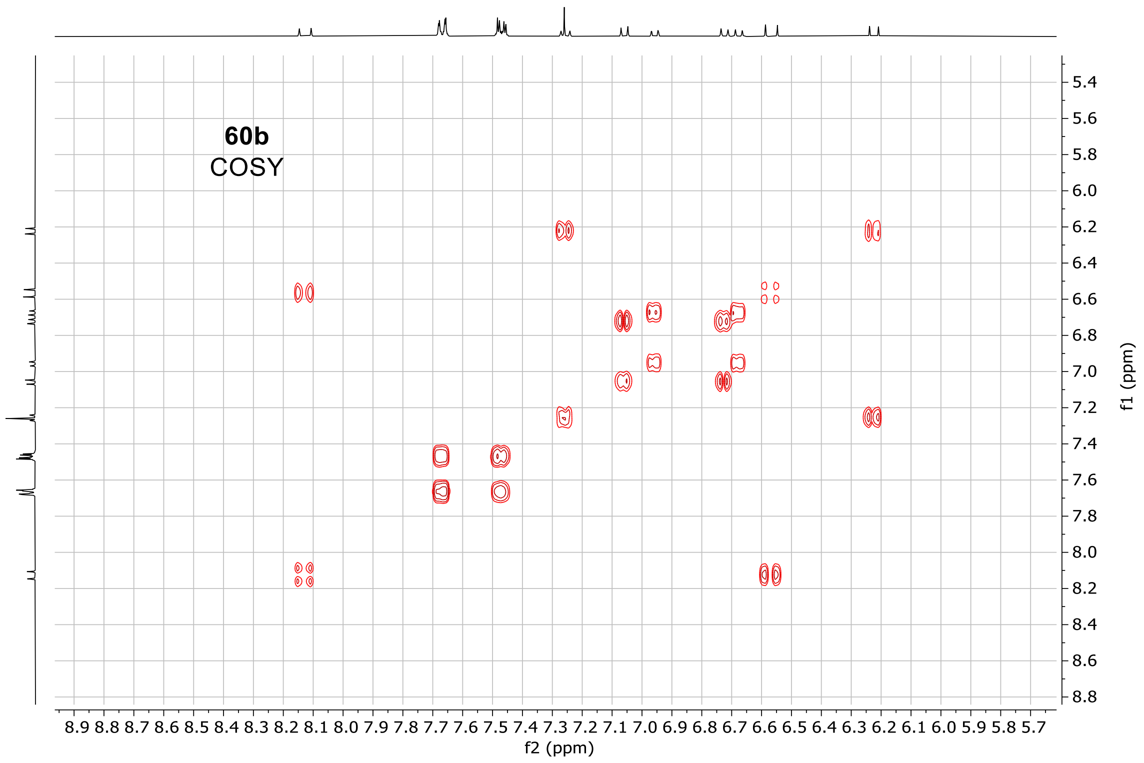

Supplementary Figure 212 COSY-NMR of compound 60b. $CDCl_3$, RT

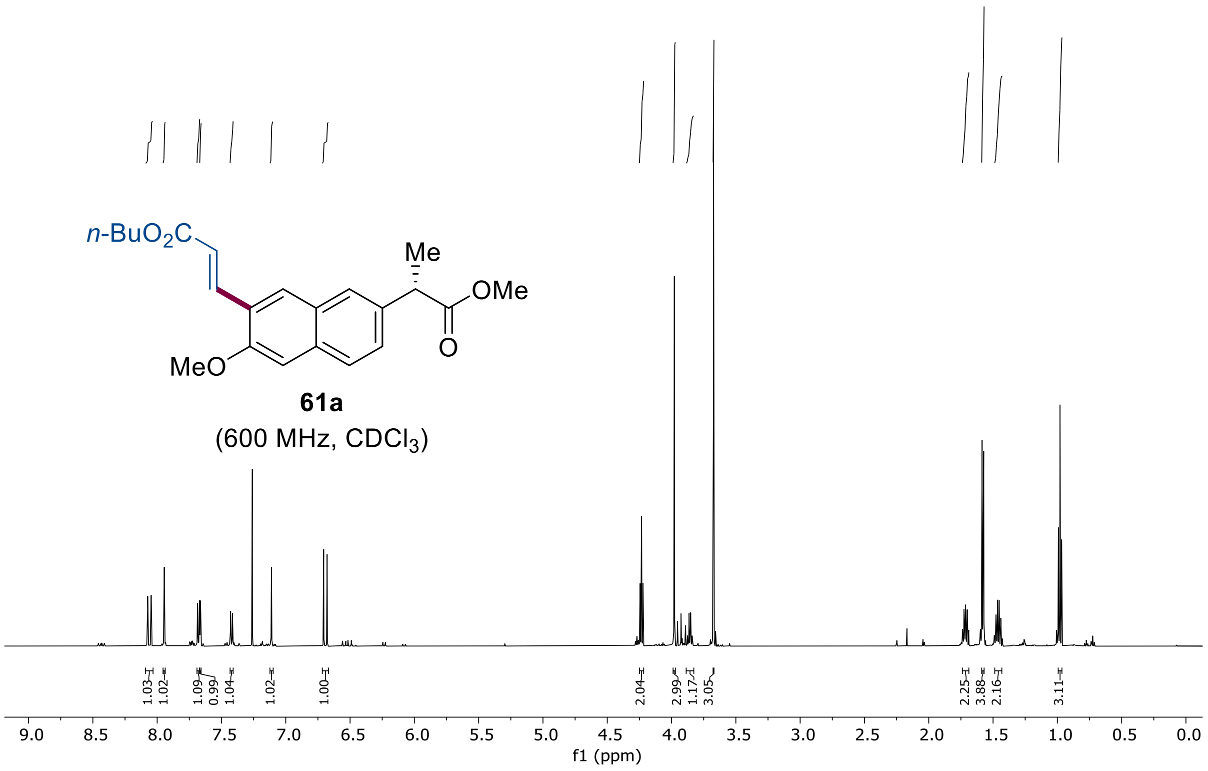

Supplementary Figure 213 H-NMR of compound 61a. 600 MHz, $CDCl_3$, RT

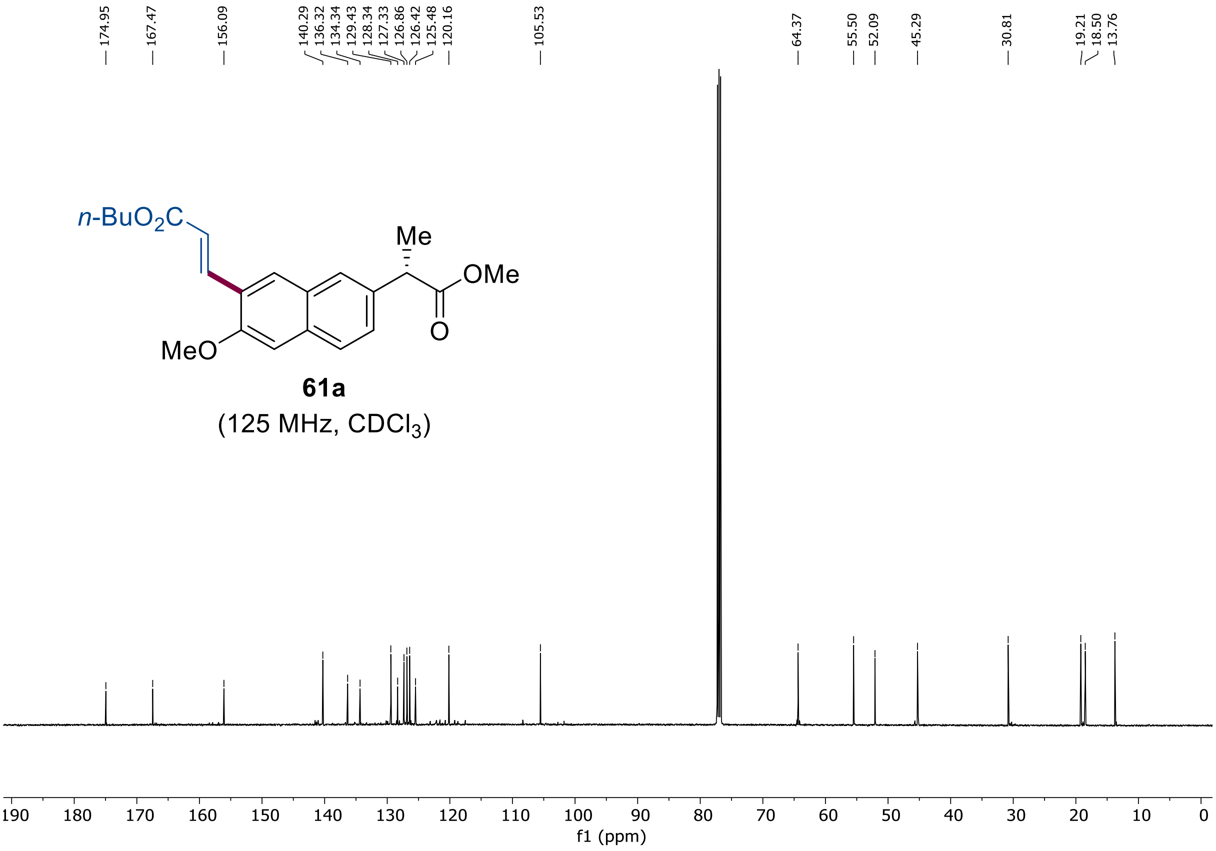

Supplementary Figure 214 C-NMR of compound 61a. 125 MHz, $CDCl_3$, RT

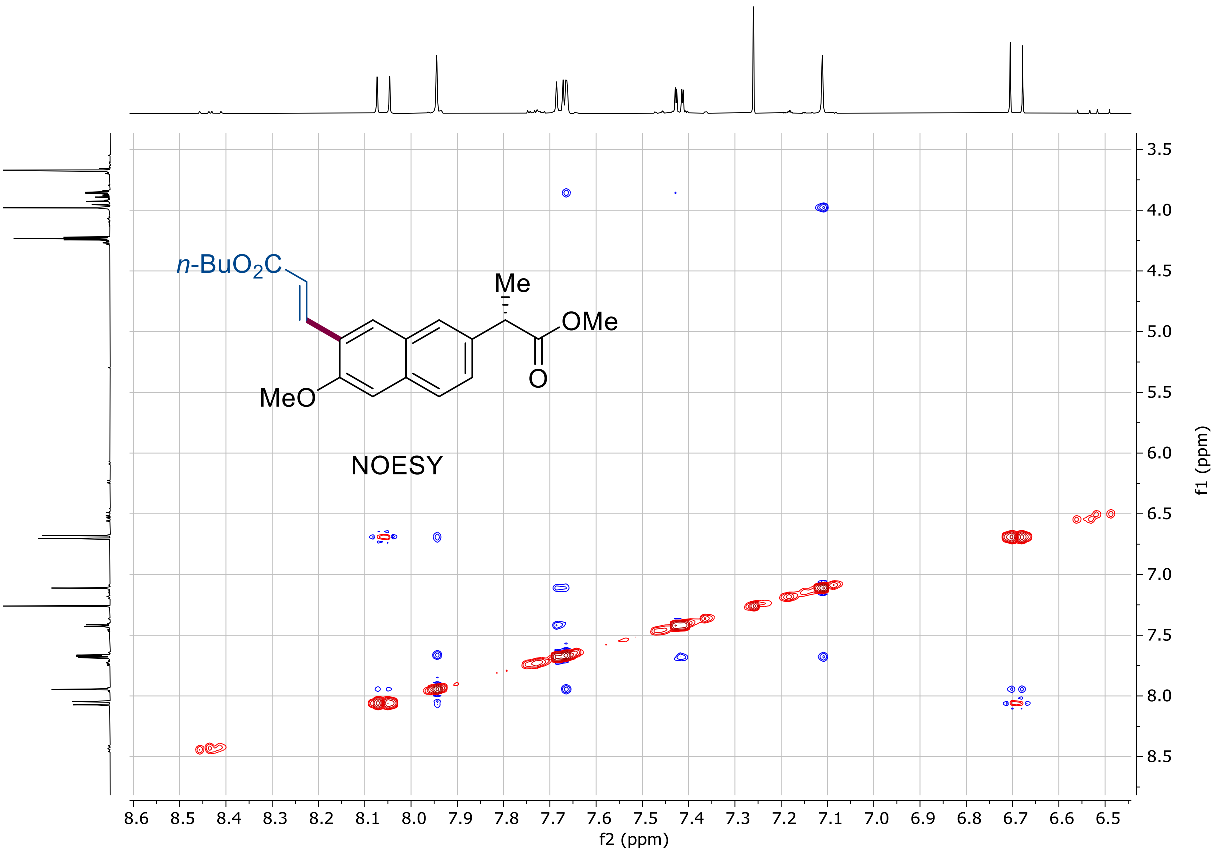

Supplementary Figure 215 NOESY-NMR of compound 61a. $CDCl_3$, RT

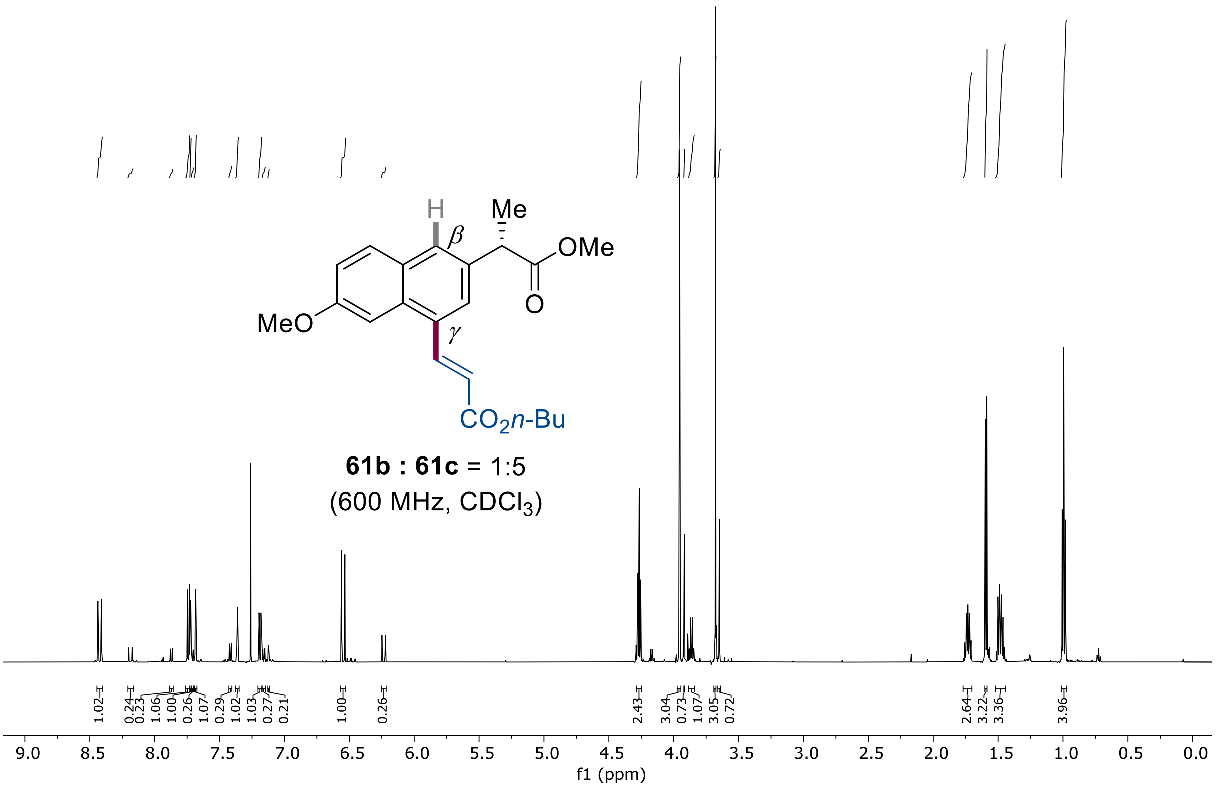

Supplementary Figure 216 H-NMR of compound 61b&c. 600 MHz, $CDCl_3$, RT

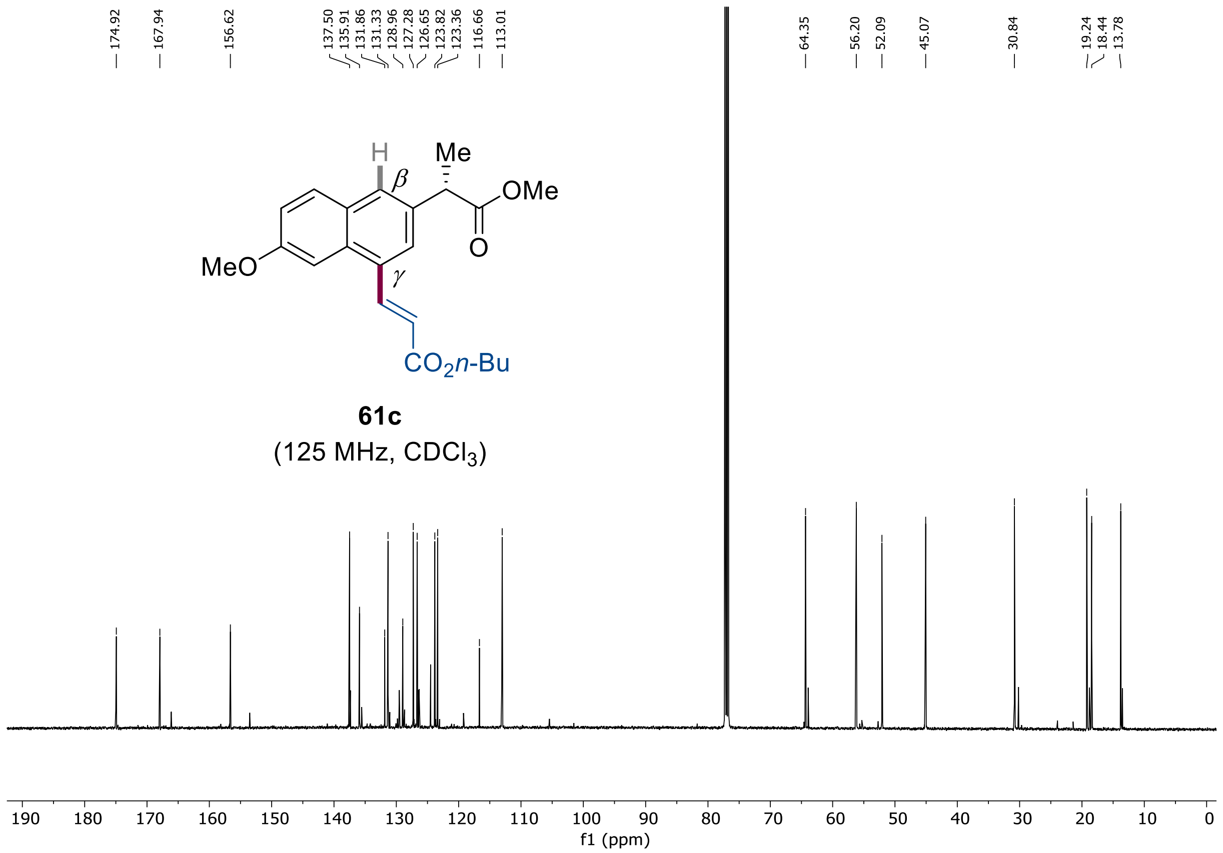

Supplementary Figure 217 C-NMR of compound 61c. 125 MHz, $CDCl_3$, RT

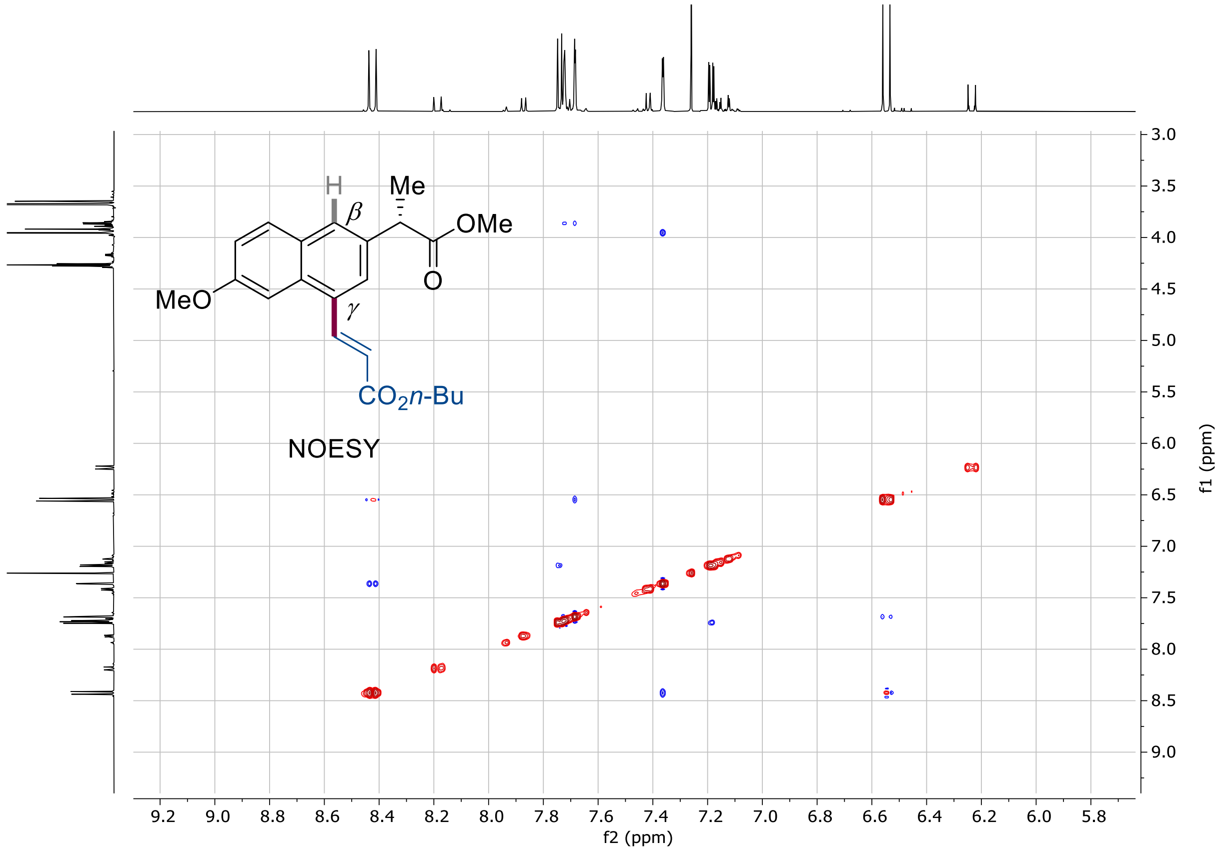

Supplementary Figure 218 NOESY-NMR of compound 61c. $CDCl_3$, RT

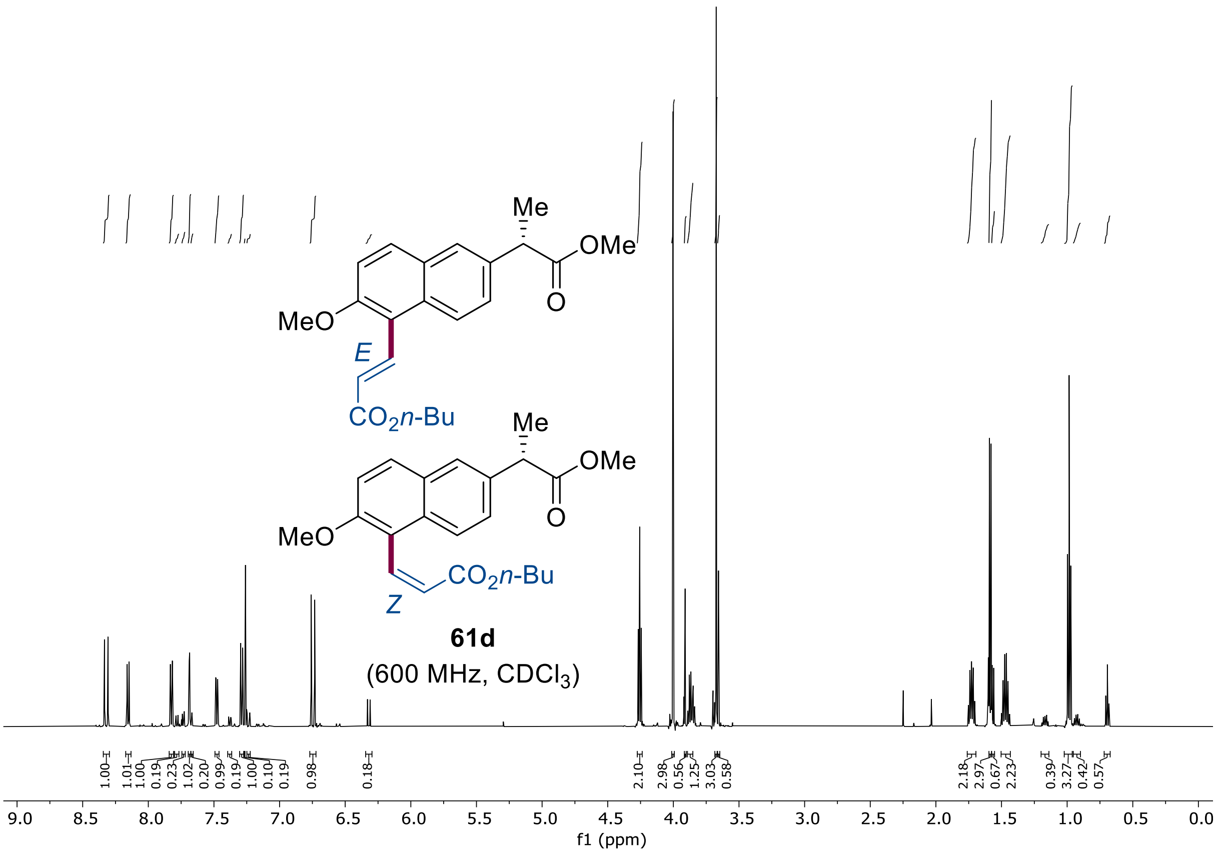

Supplementary Figure 219 H-NMR of compound 61d. 600 MHz, $CDCl_3$, RT

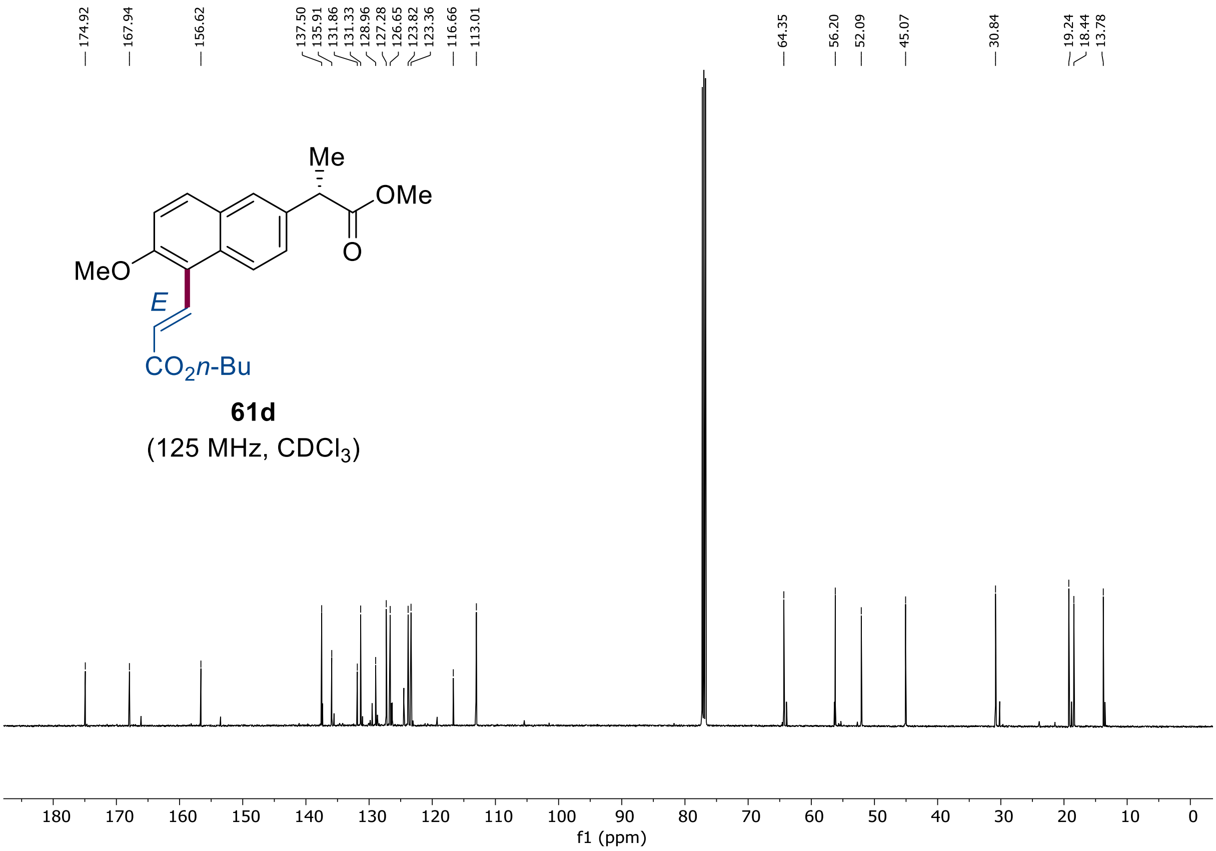

Supplementary Figure 220 C-NMR of compound 61d. 125 MHz, $CDCl_3$, RT

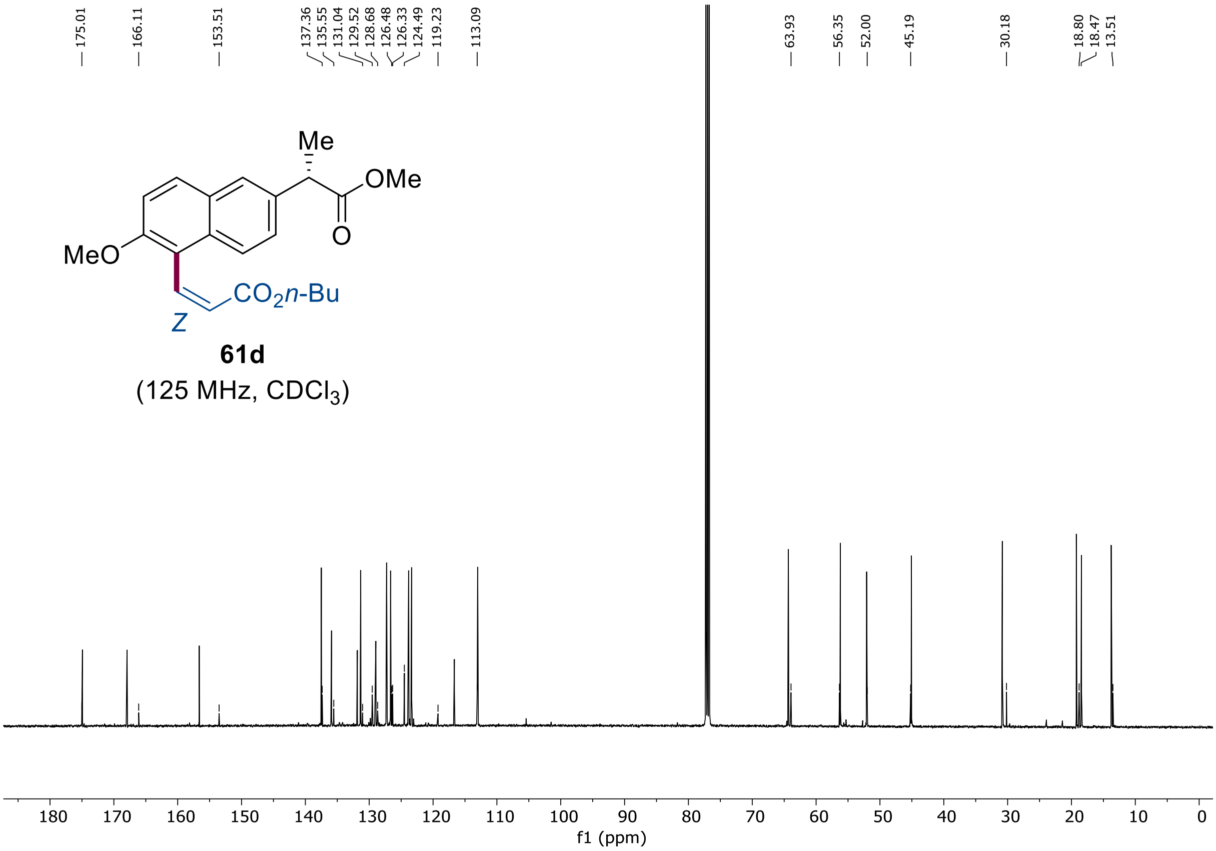

Supplementary Figure 221 C-NMR of compound 61d. 125 MHz, $CDCl_3$, RT

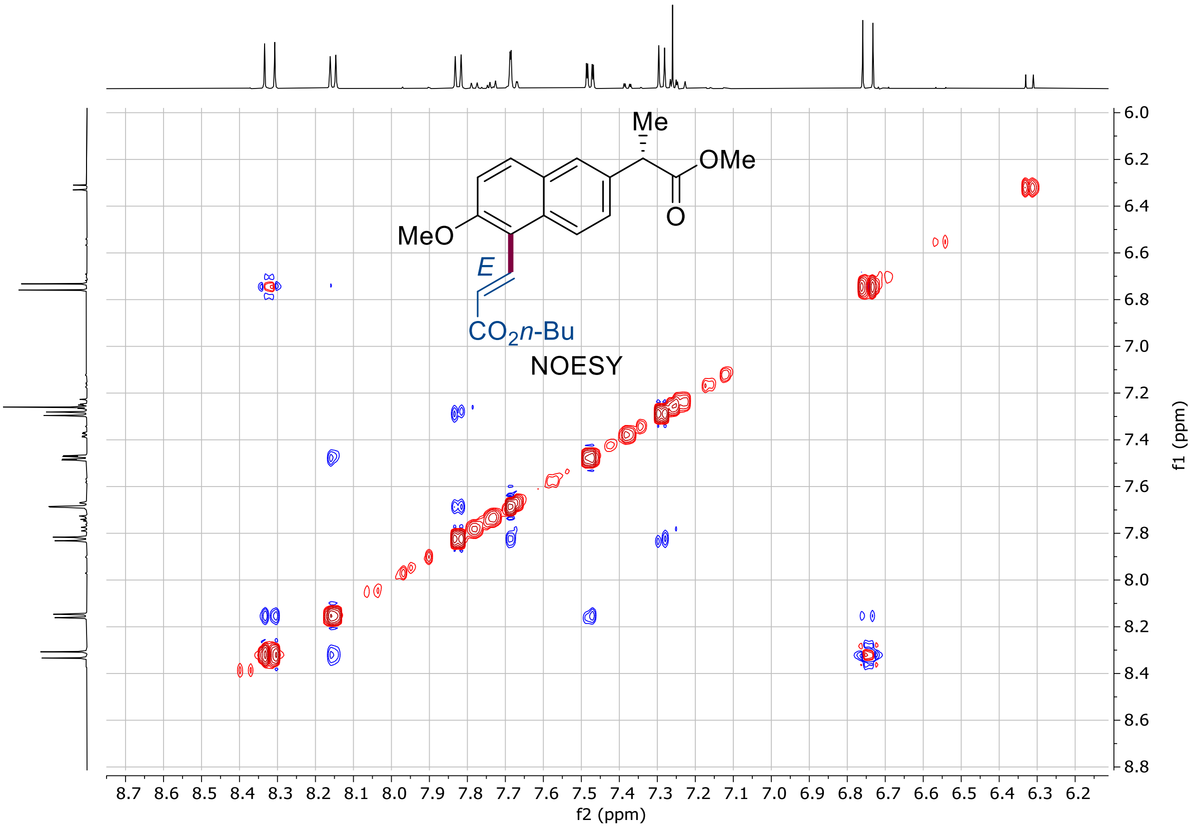

Supplementary Figure 222 NOESY-NMR of compound 61d. $CDCl_3$, RT

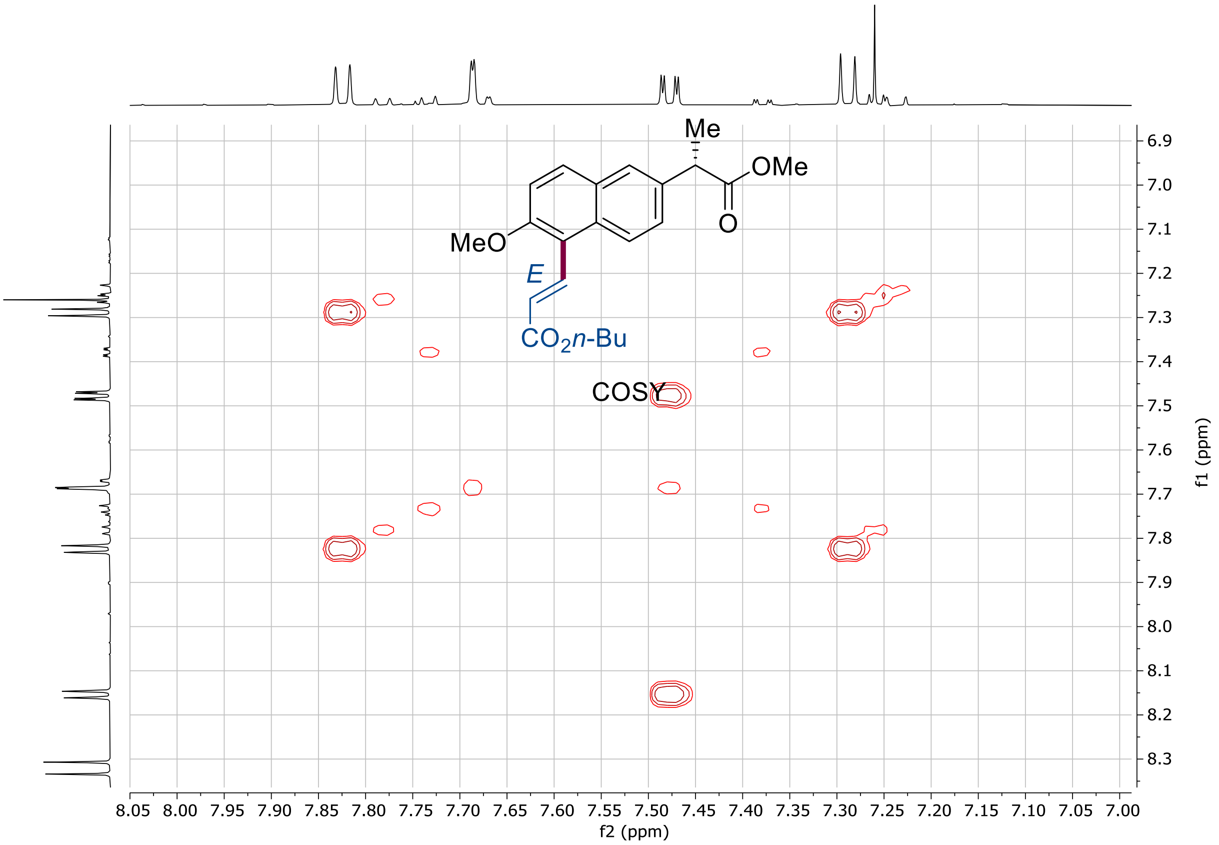

Supplementary Figure 223 COSY-NMR of compound 61d. CDCl₃, RT

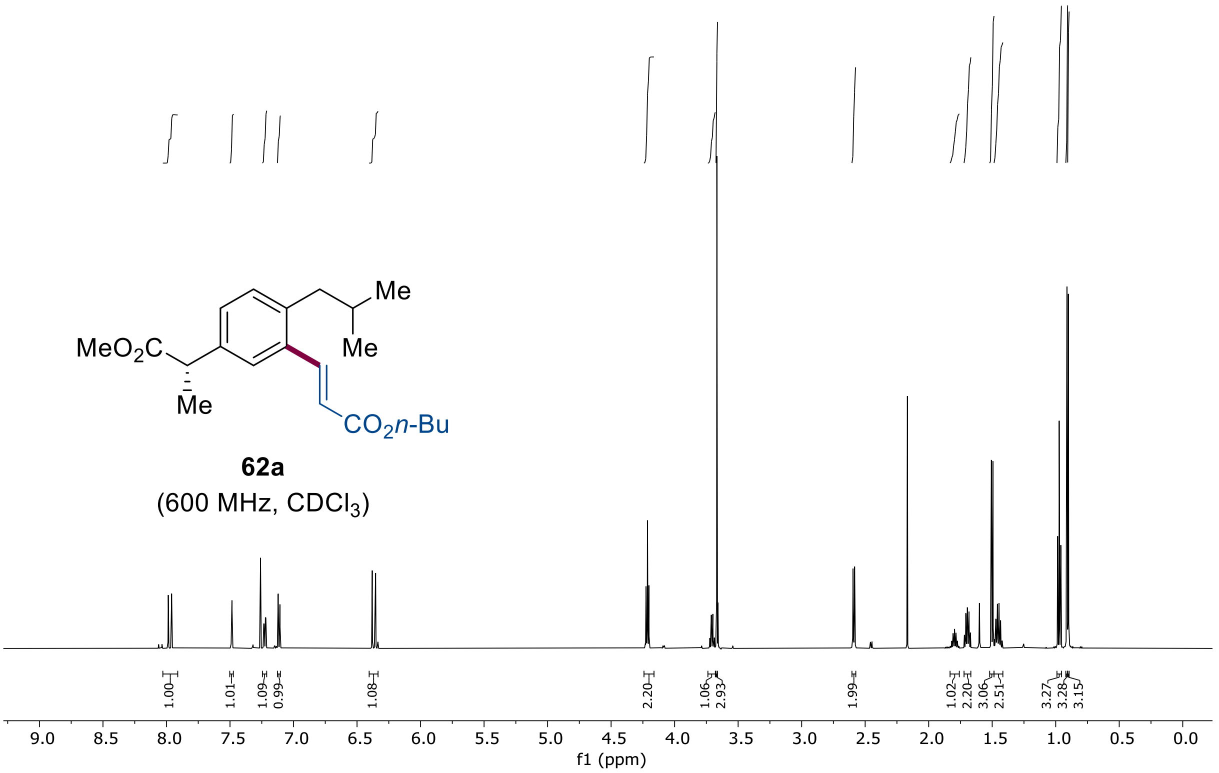

Supplementary Figure 224 H-NMR of compound 62a. 600 MHz, CDCl₃, RT

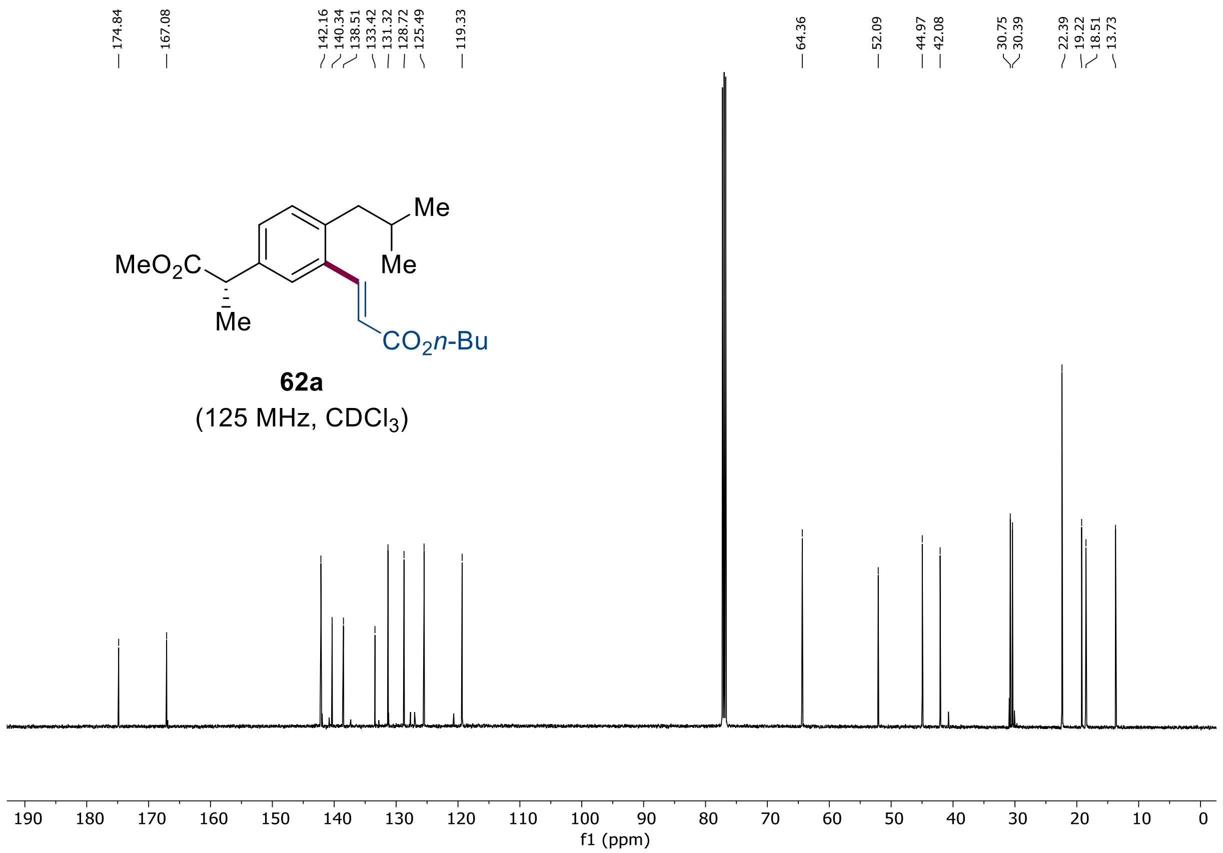

Supplementary Figure 225 C-NMR of compound 62a. 125 MHz, $CDCl_3$, RT

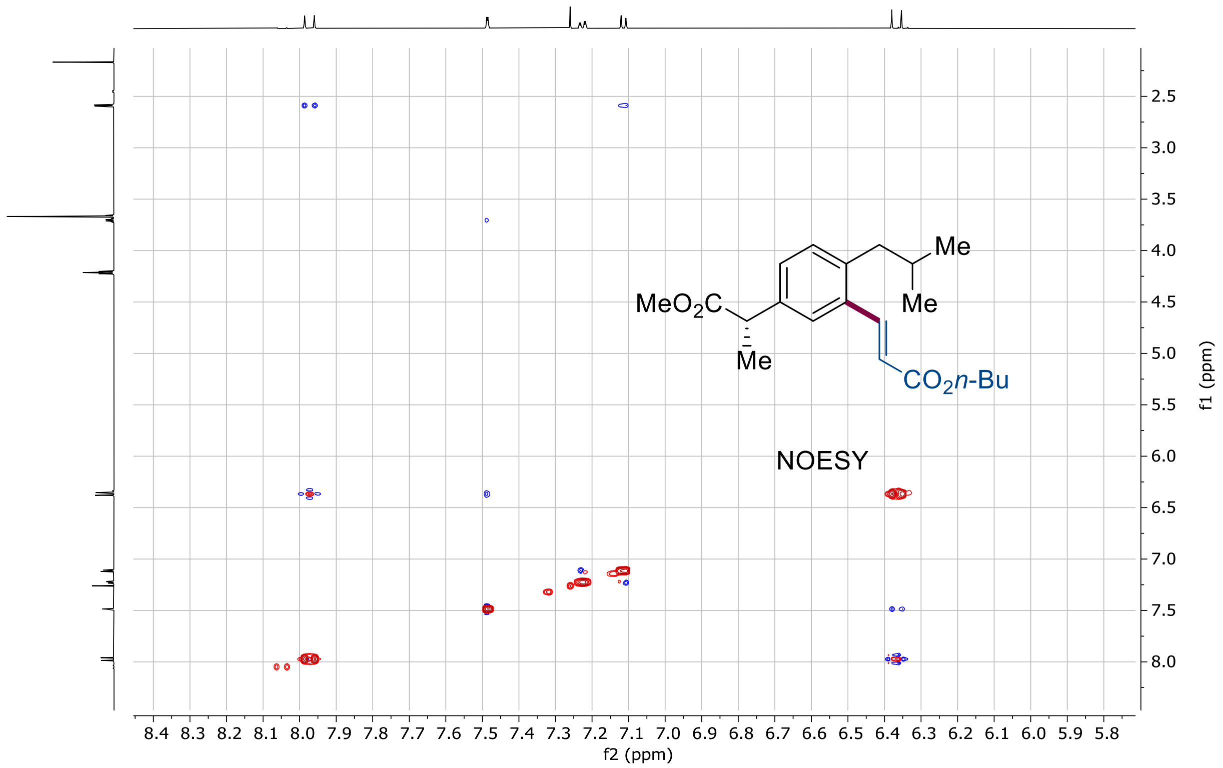

Supplementary Figure 226 NOESY-NMR of compound 62a. $CDCl_3$, RT

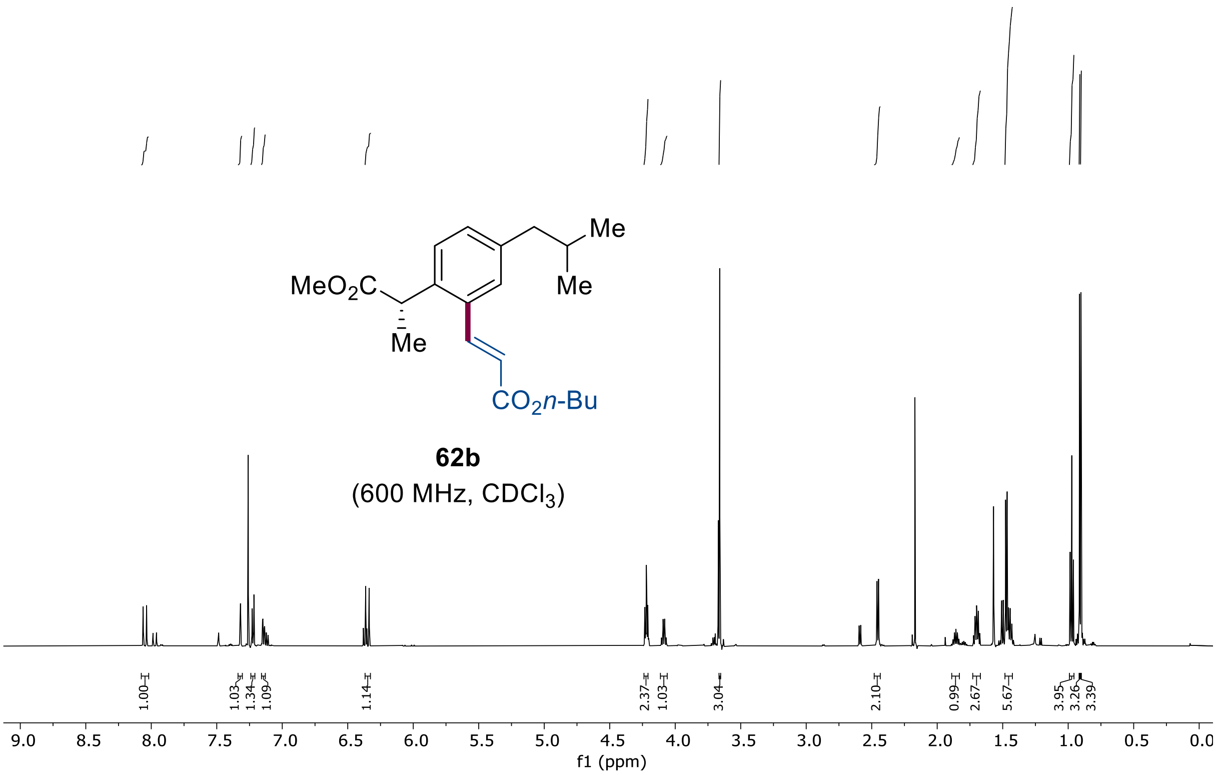

Supplementary Figure 227 H-NMR of compound 62b. 600 MHz, $CDCl_3$, RT

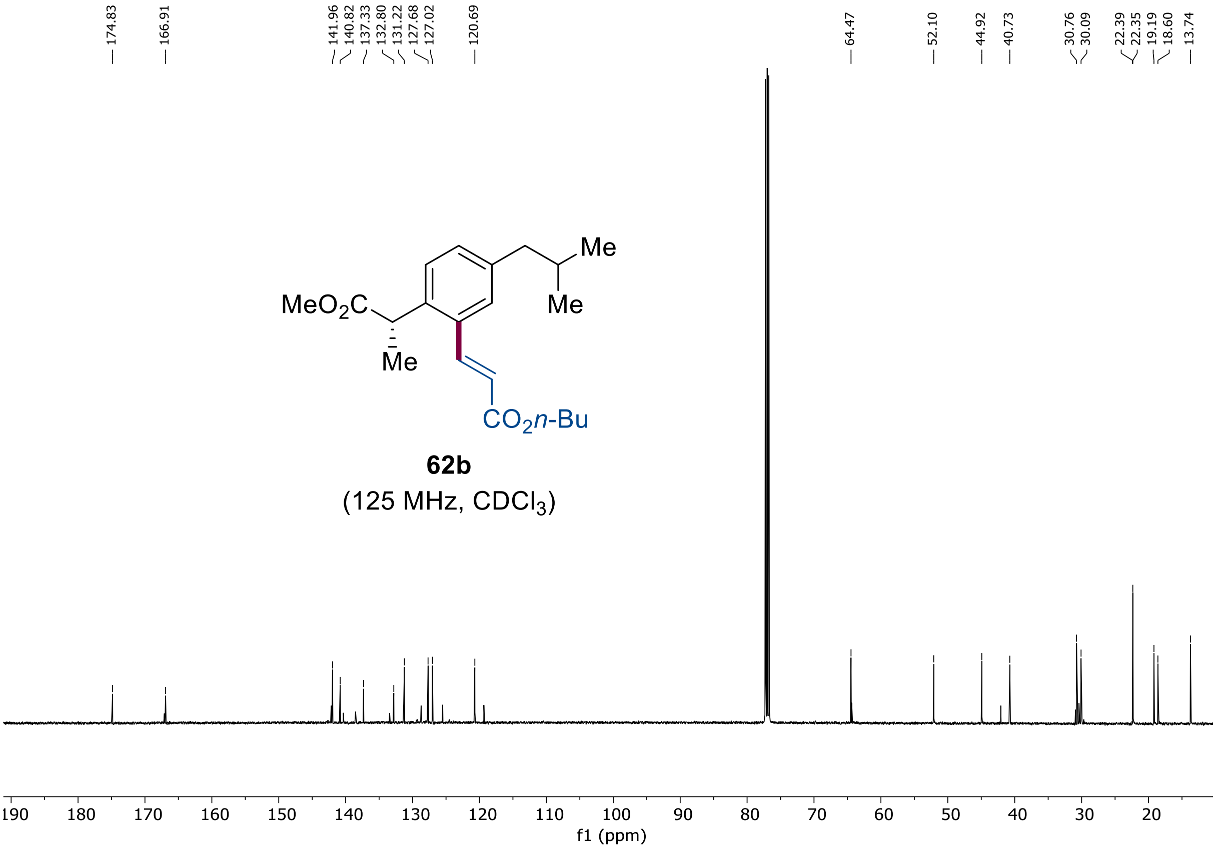

Supplementary Figure 228 C-NMR of compound 62b. 125 MHz, $CDCl_3$, RT

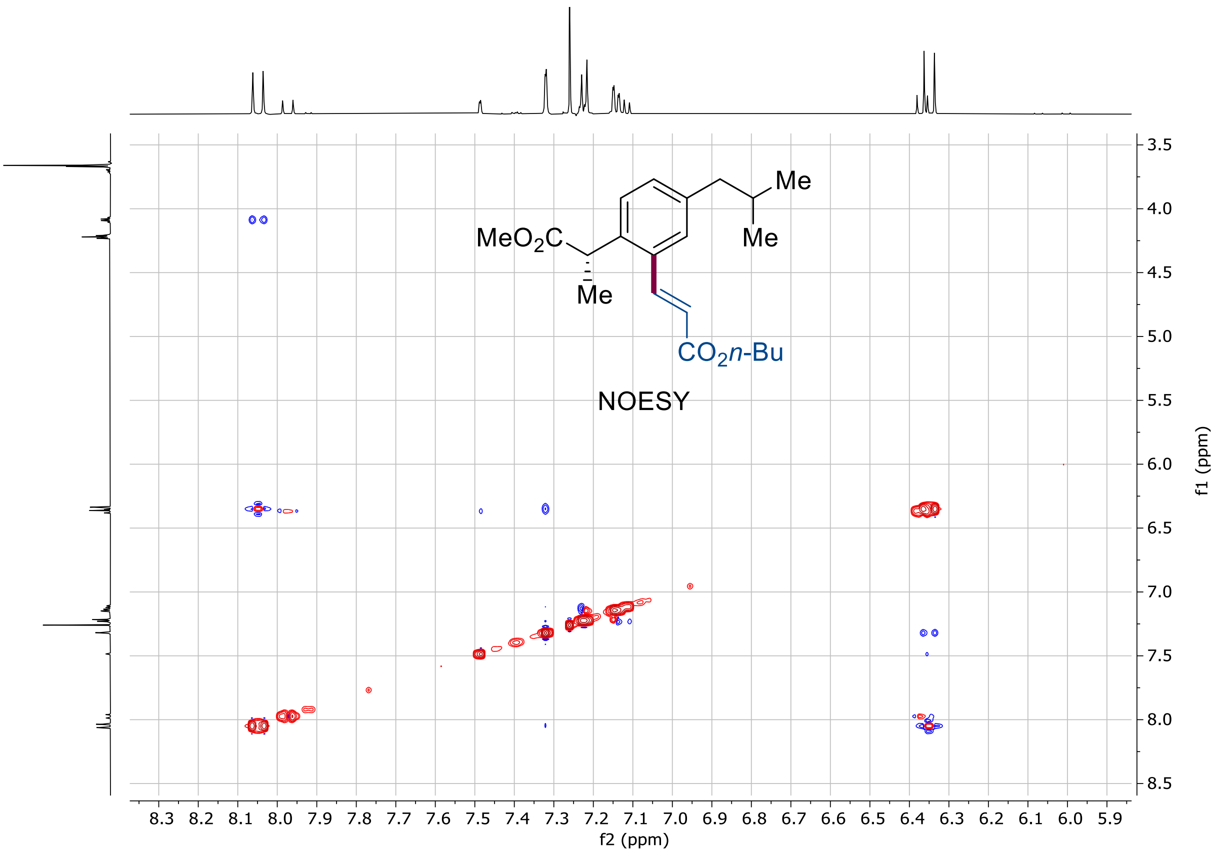

Supplementary Figure 229 NOESY-NMR of compound 62b. CDCl$_3$, RT

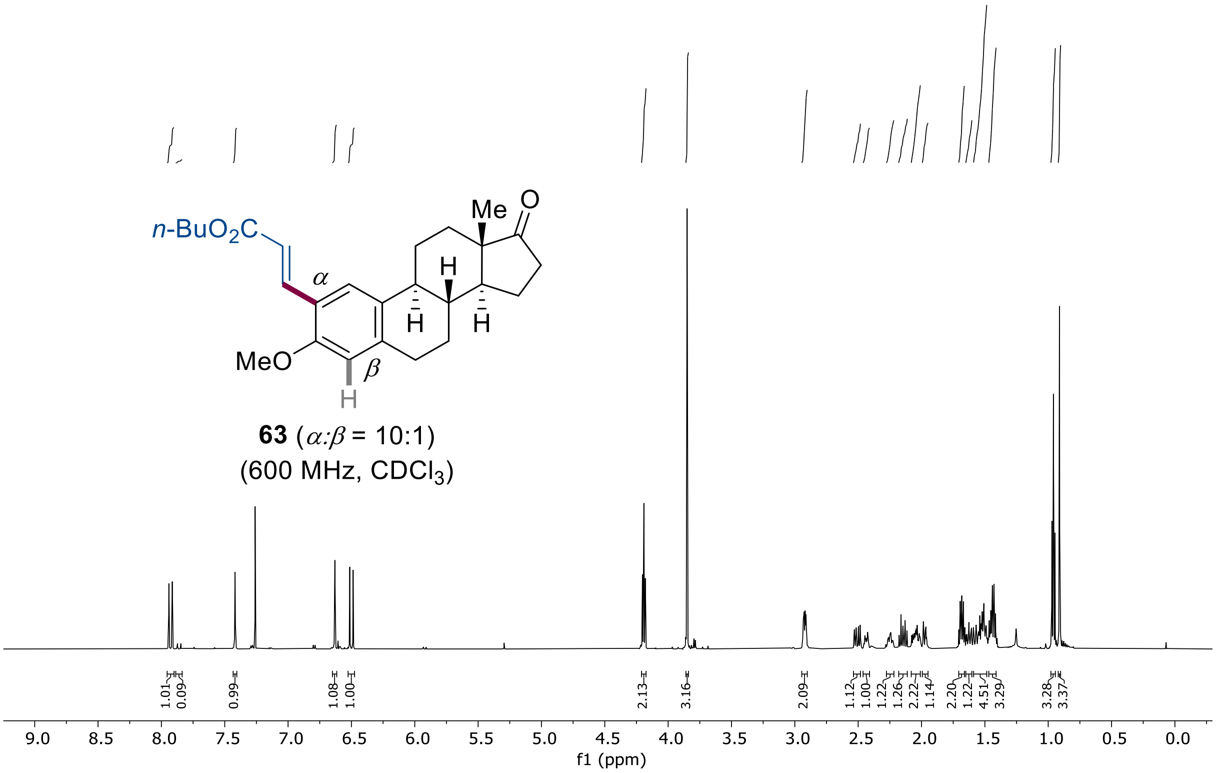

Supplementary Figure 230 H-NMR of compound 63. 600 MHz, CDCl$_3$, RT

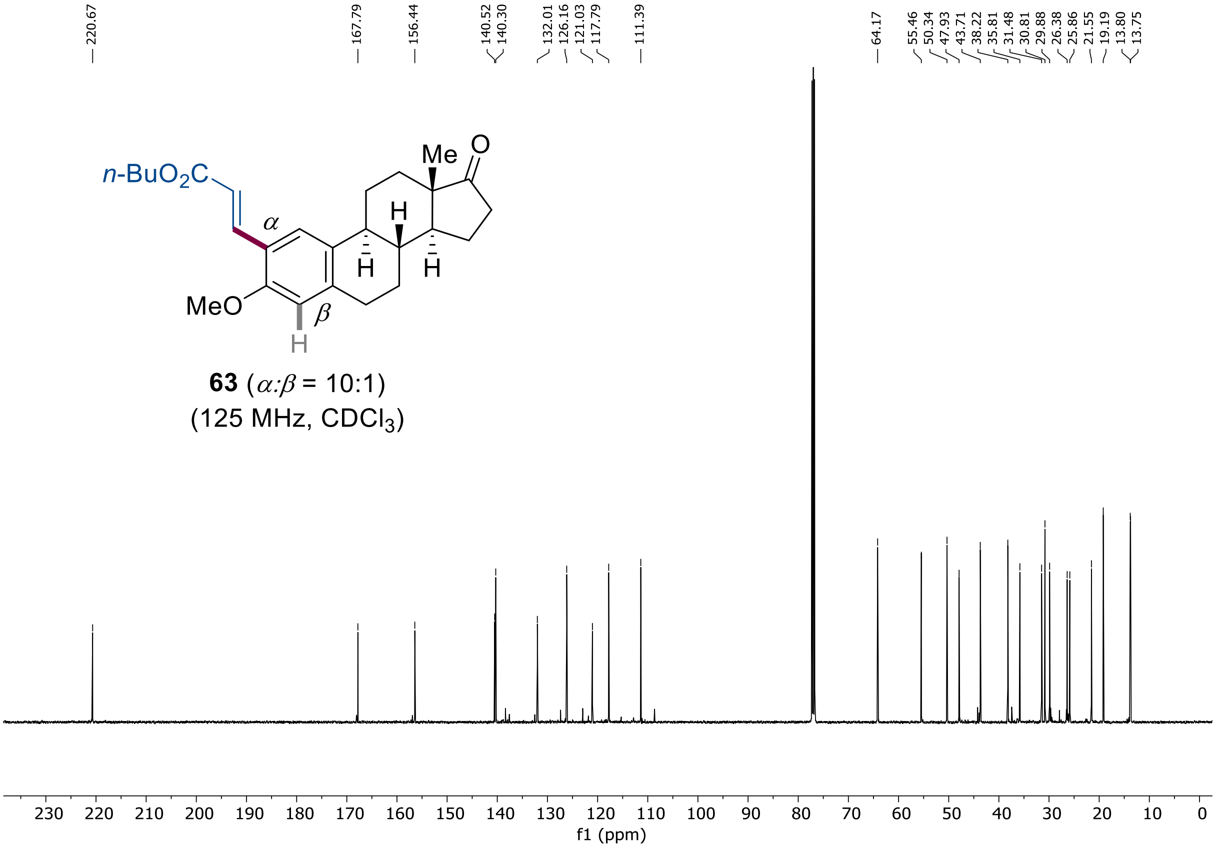

Supplementary Figure 231 C-NMR of compound 63. 125 MHz, $CDCl_3$, RT

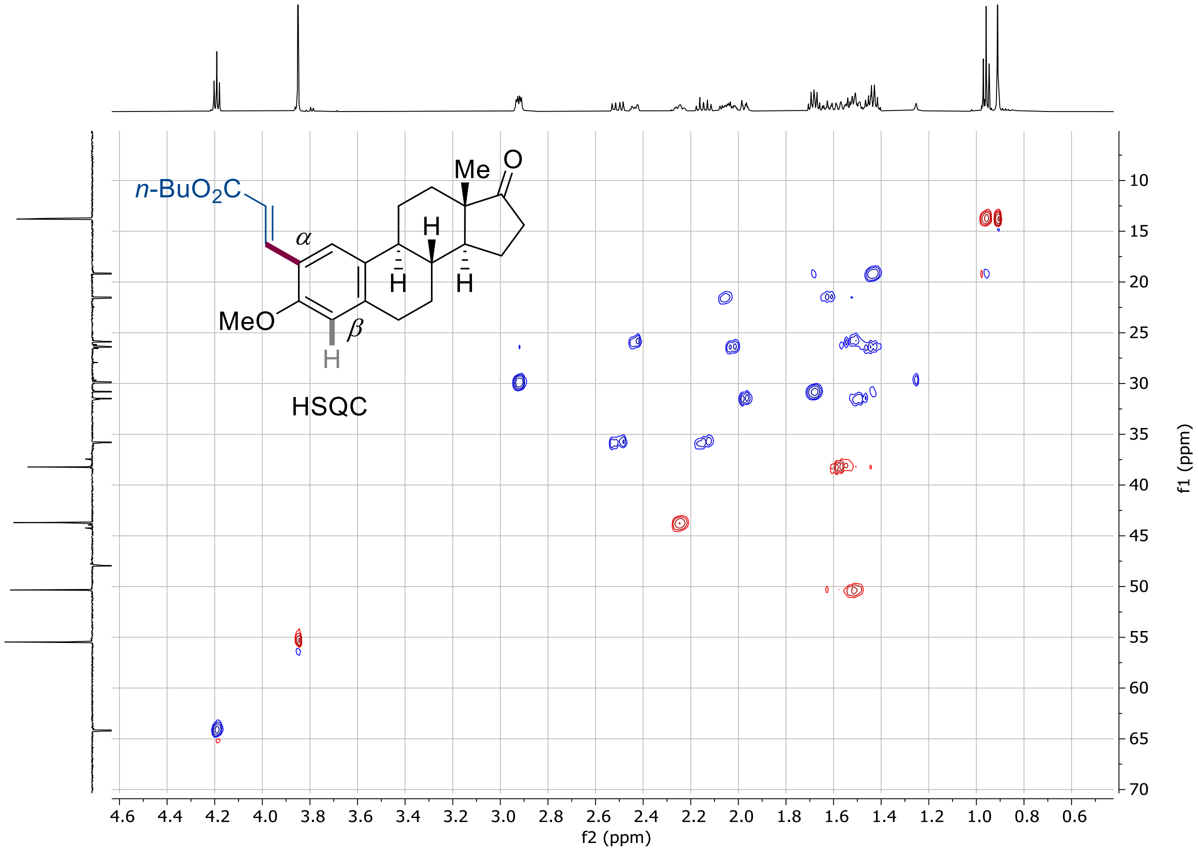

Supplementary Figure 232 HSQC-NMR of compound 63. $CDCl_3$, RT

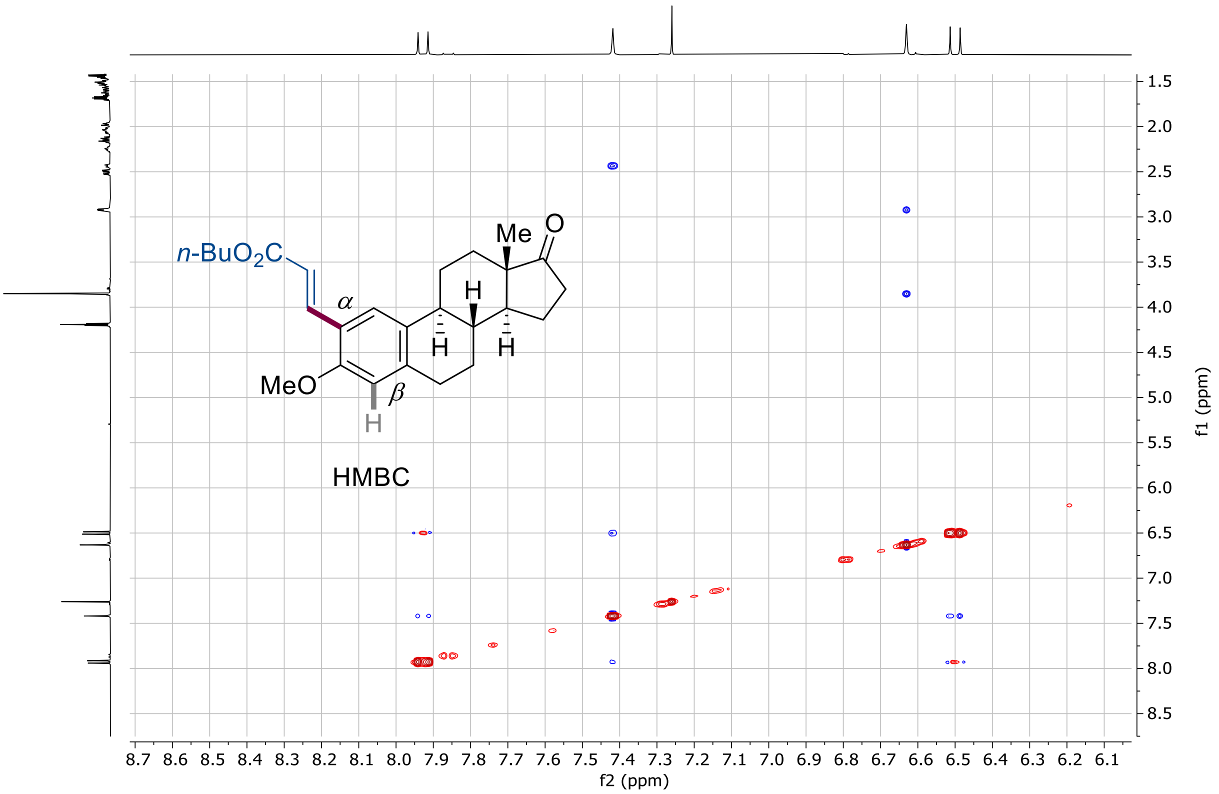

Supplementary Figure 233 HMBC-NMR of compound 63. CDCl₃, RT

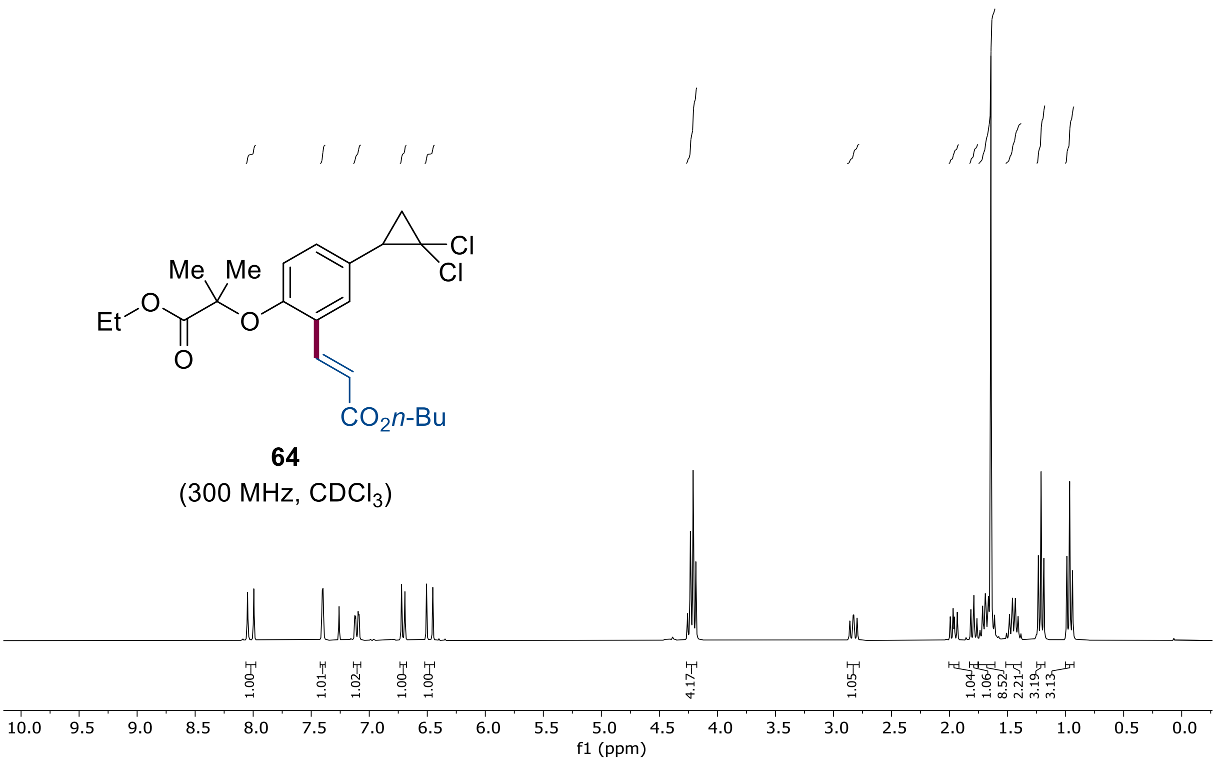

Supplementary Figure 234 H-NMR of compound 64. 300 MHz, CDCl₃, RT

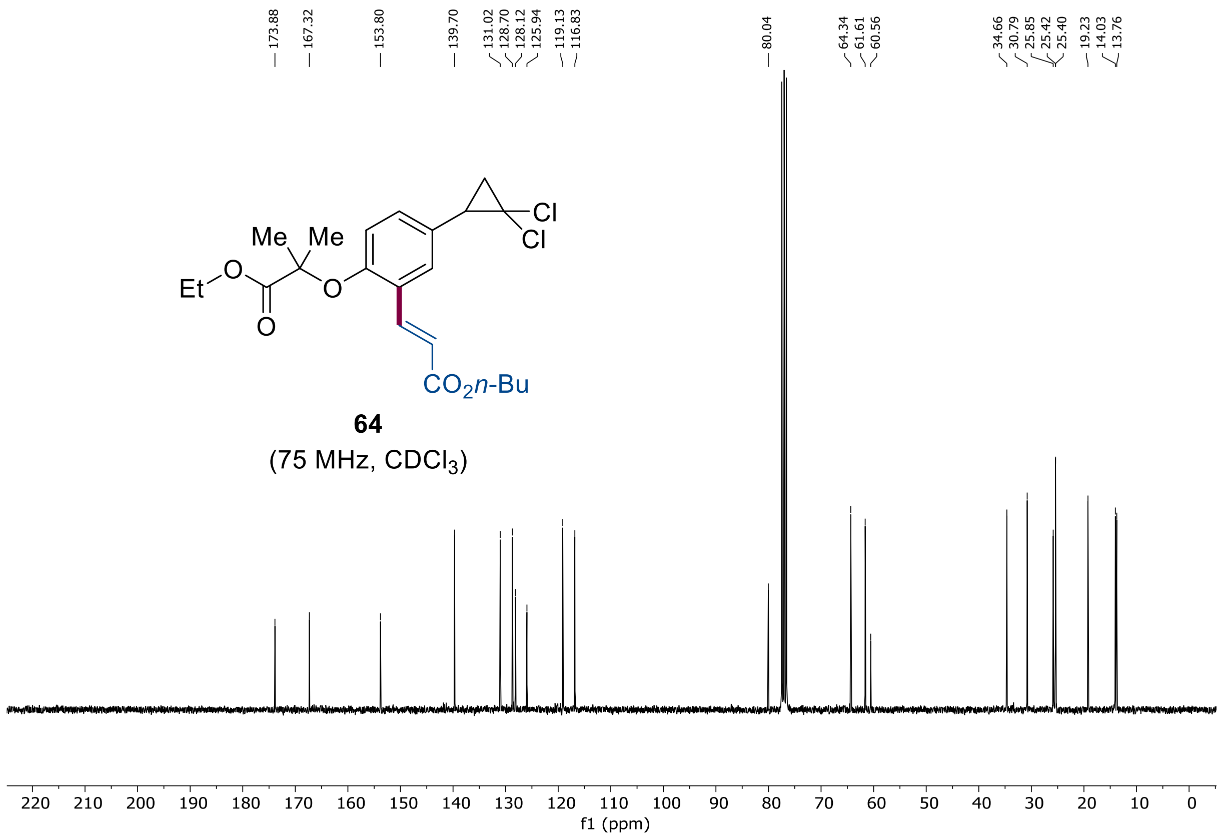

Supplementary Figure 235 C-NMR of compound 64. 75 MHz, $CDCl_3$, RT

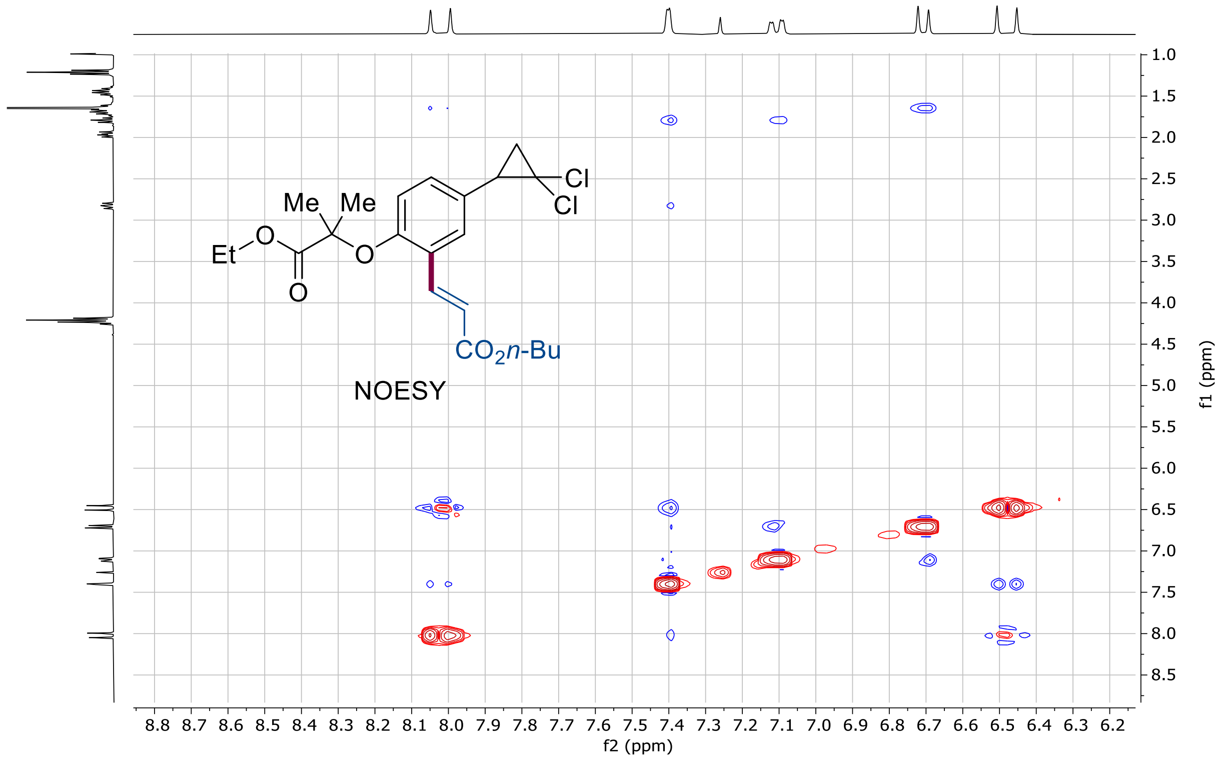

Supplementary Figure 236 NOESY-NMR of compound 64. $CDCl_3$, RT

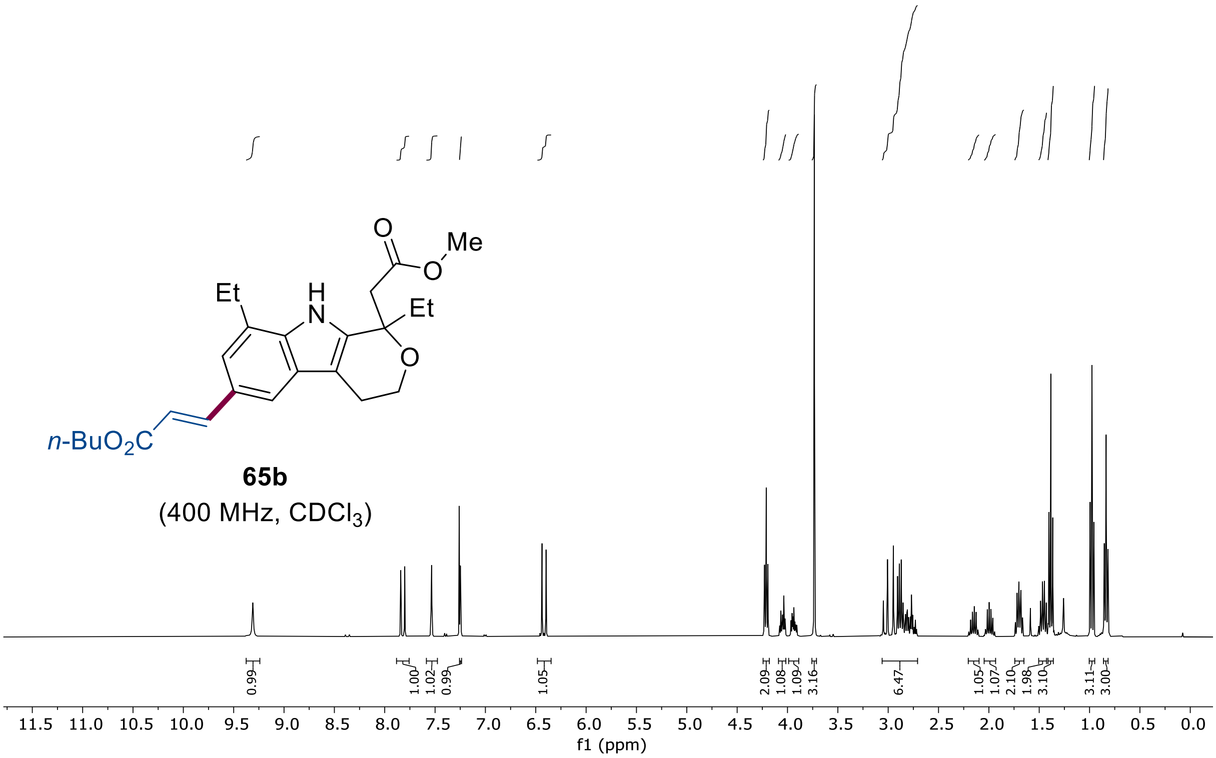

Supplementary Figure 237 H-NMR of compound 65b. 400 MHz, CDCl₃, RT

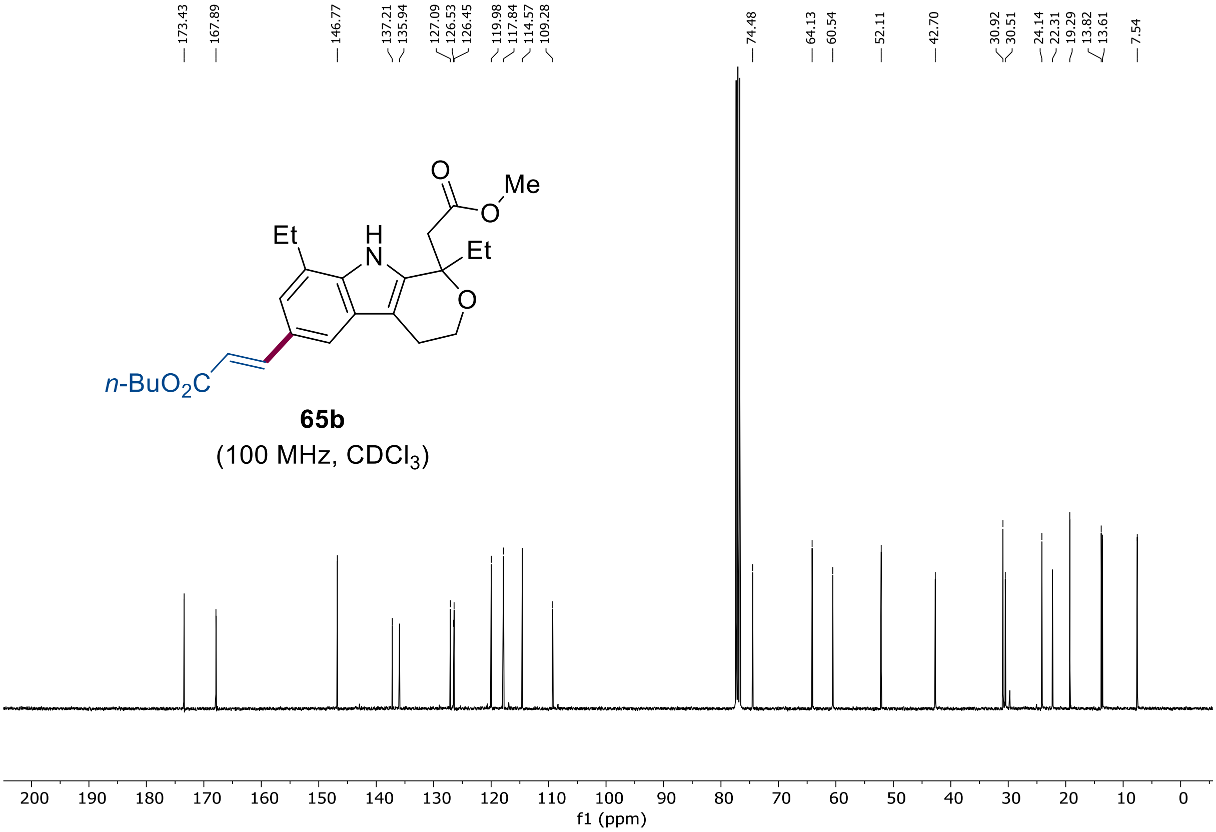

Supplementary Figure 238 C-NMR of compound 65b. 100 MHz, CDCl₃, RT

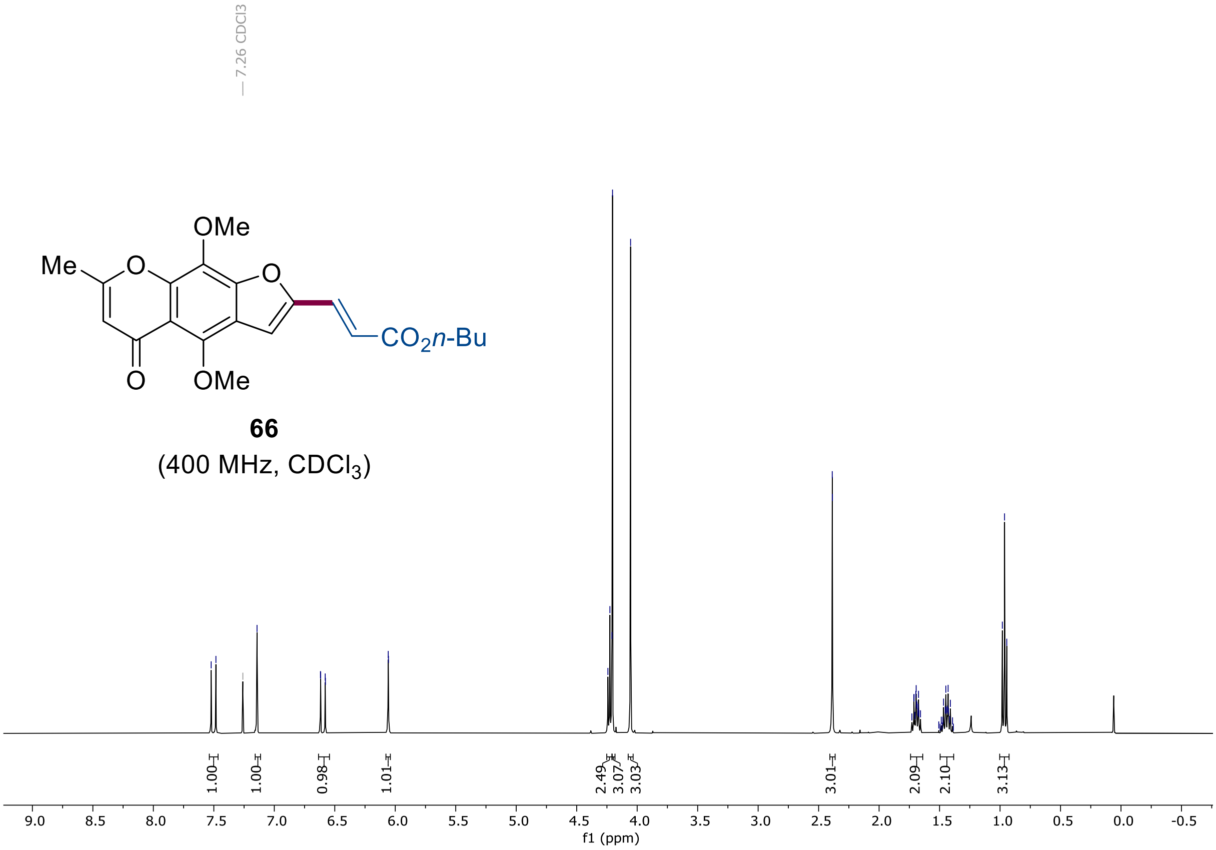

Supplementary Figure 239 H-NMR of compound 66. 400 MHz, $CDCl_3$, RT

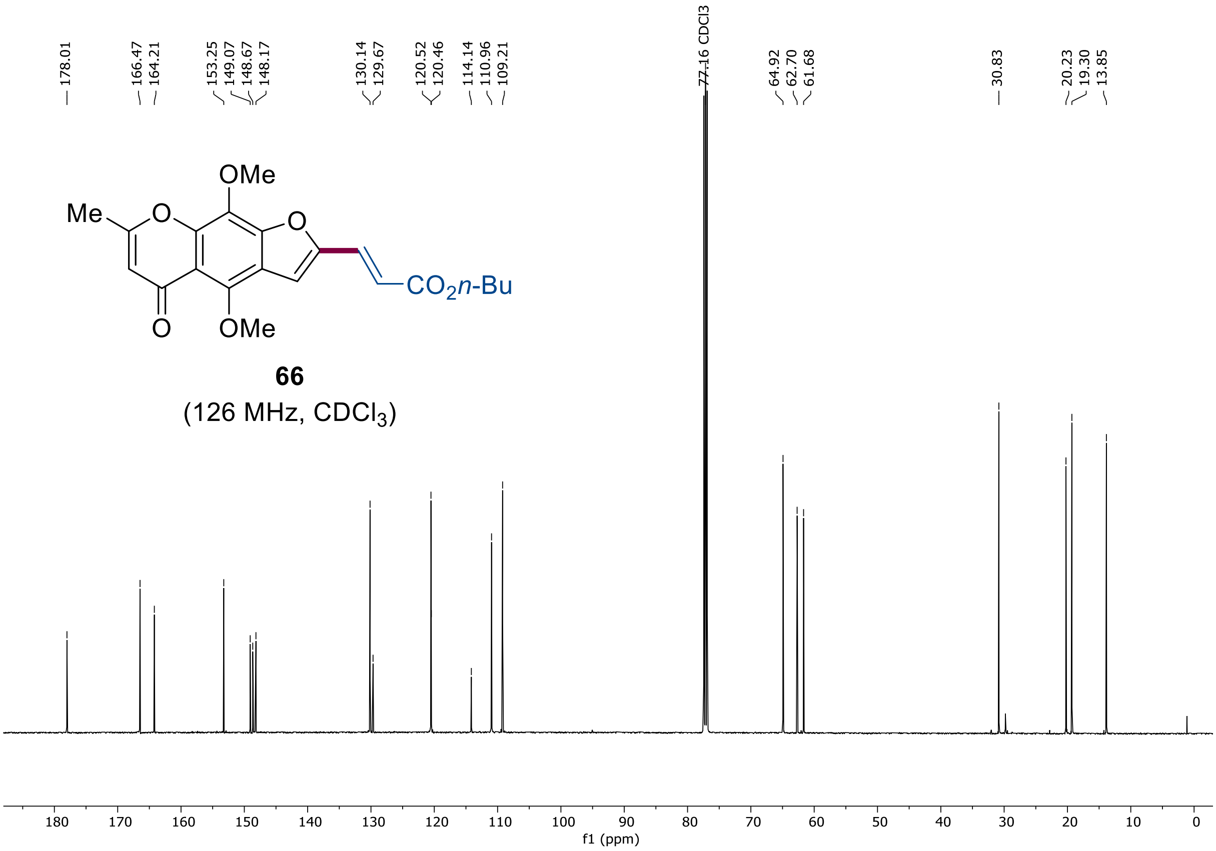

Supplementary Figure 240 C-NMR of compound 66. 126 MHz, $CDCl_3$, RT

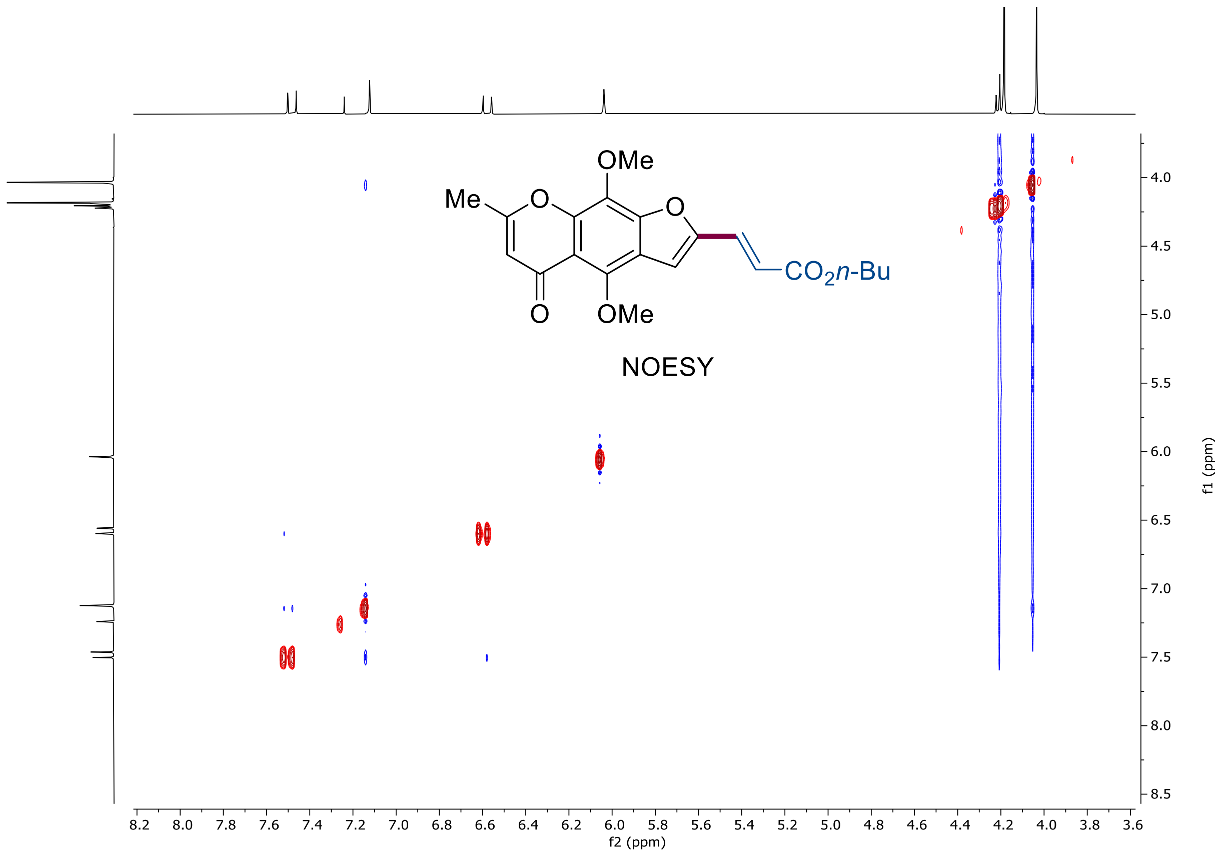

Supplementary Figure 241 NOESY-NMR of compound 66. $CDCl_3$, RT

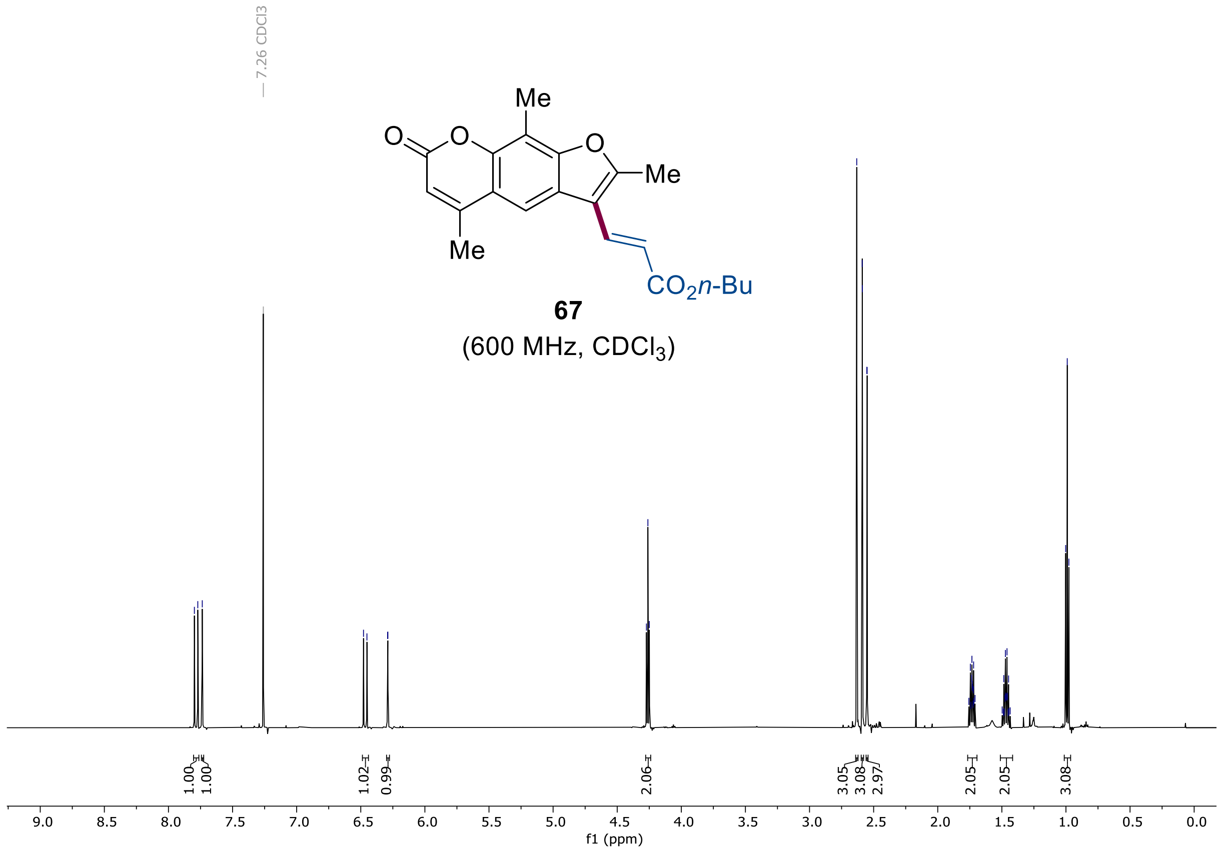

Supplementary Figure 242 H-NMR of compound 67. 600 MHz, $CDCl_3$, RT

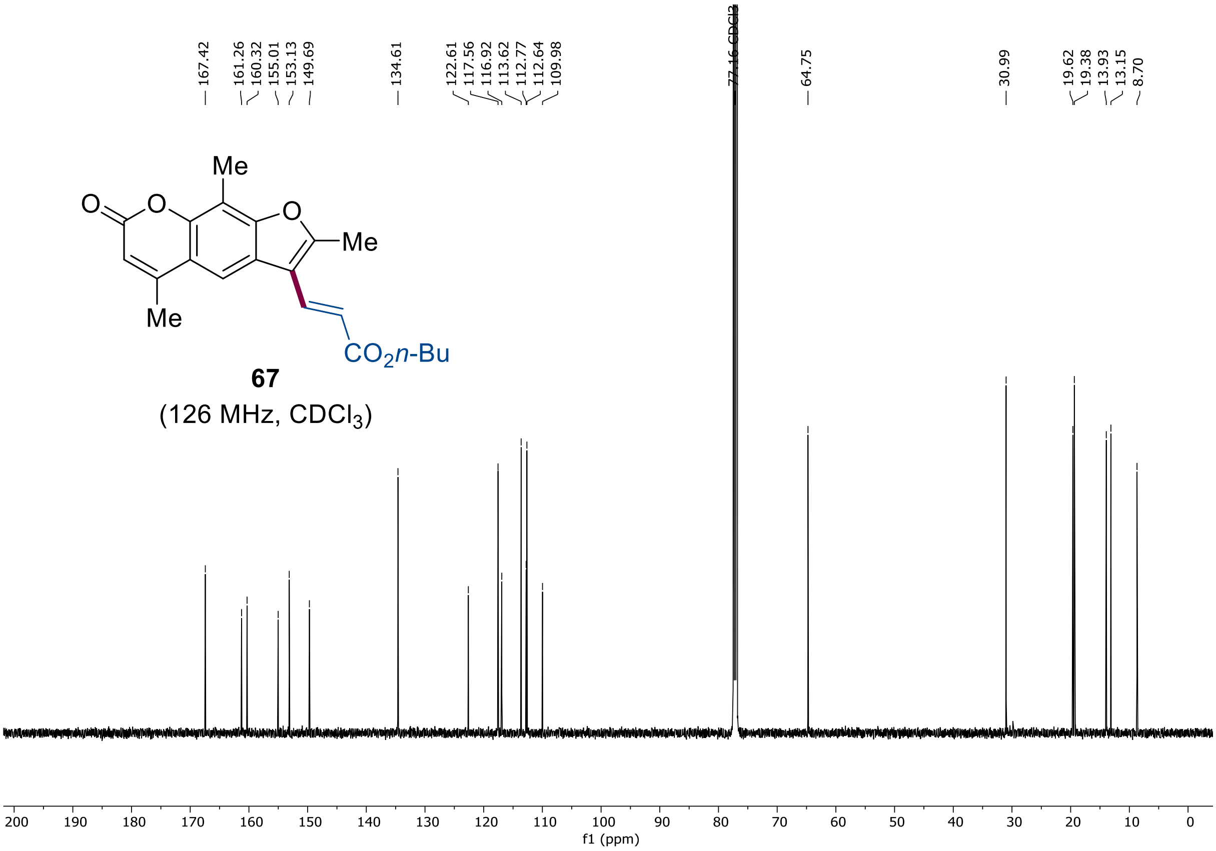

Supplementary Figure 243 C-NMR of compound 67. 126 MHz, $CDCl_3$, RT

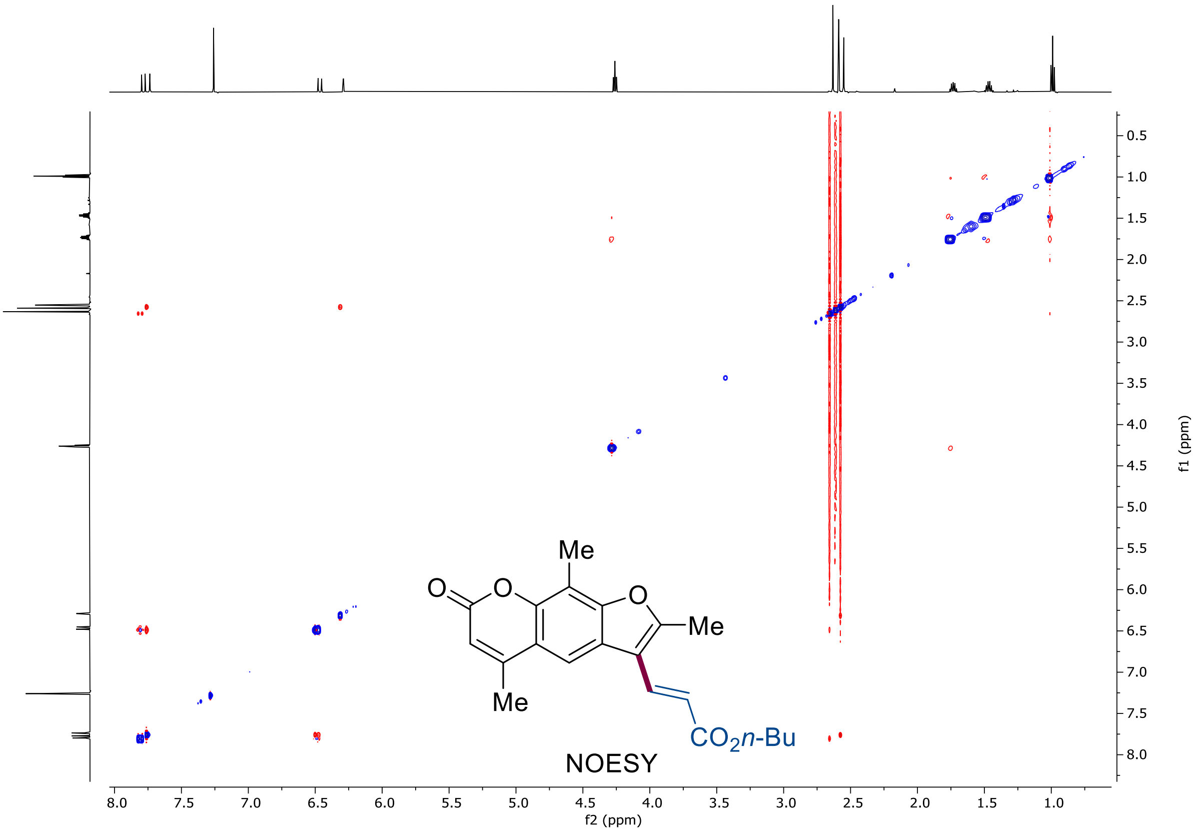

Supplementary Figure 244 NOESY-NMR of compound 67. $CDCl_3$, RT

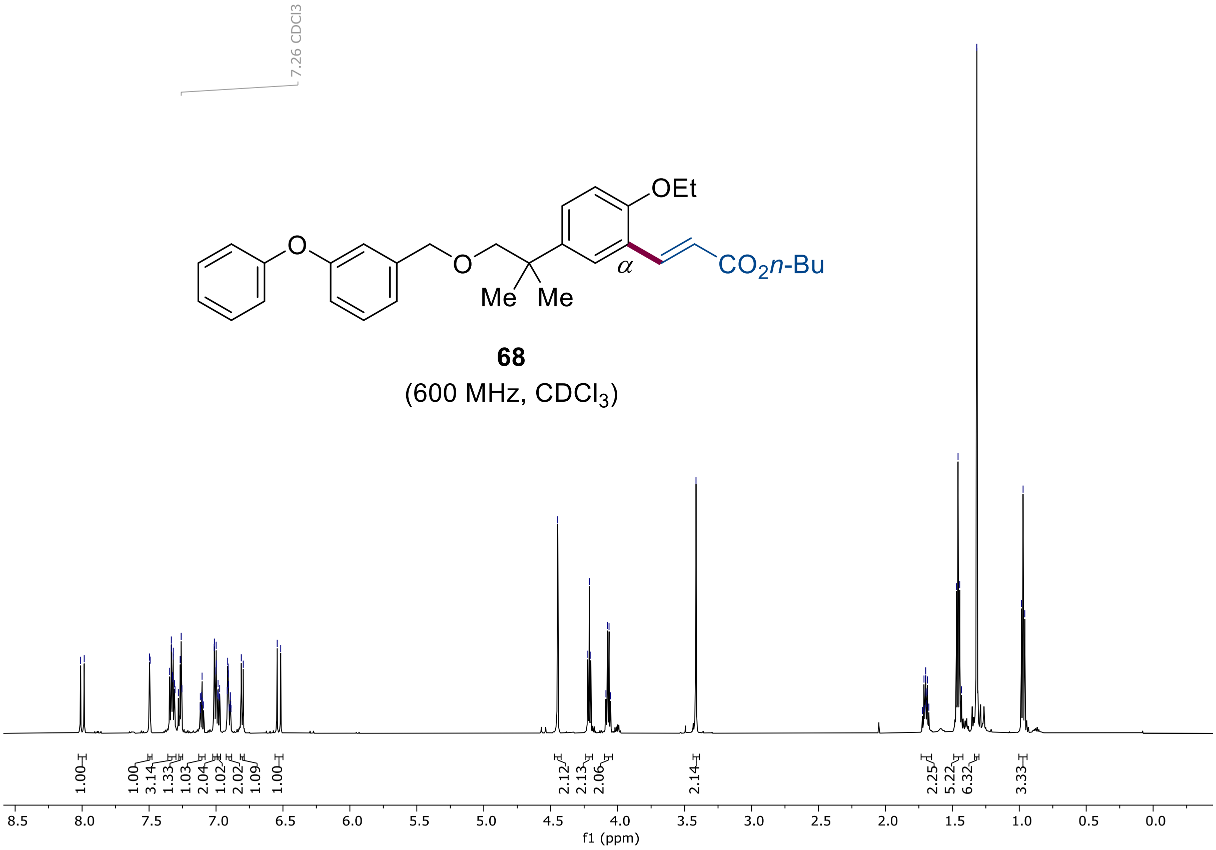

Supplementary Figure 245 H-NMR of compound 68. 600 MHz, $CDCl_3$, RT

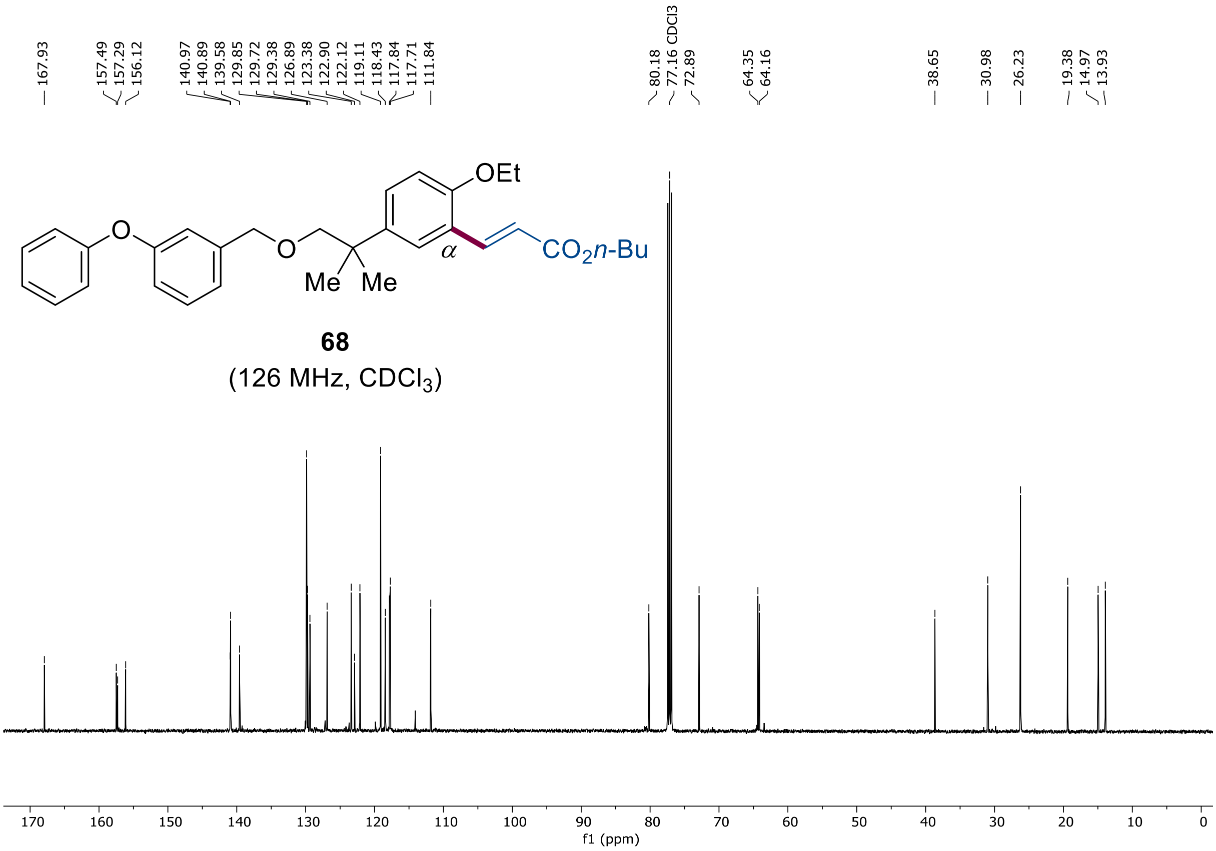

Supplementary Figure 246 C-NMR of compound 68. 126 MHz, $CDCl_3$, RT

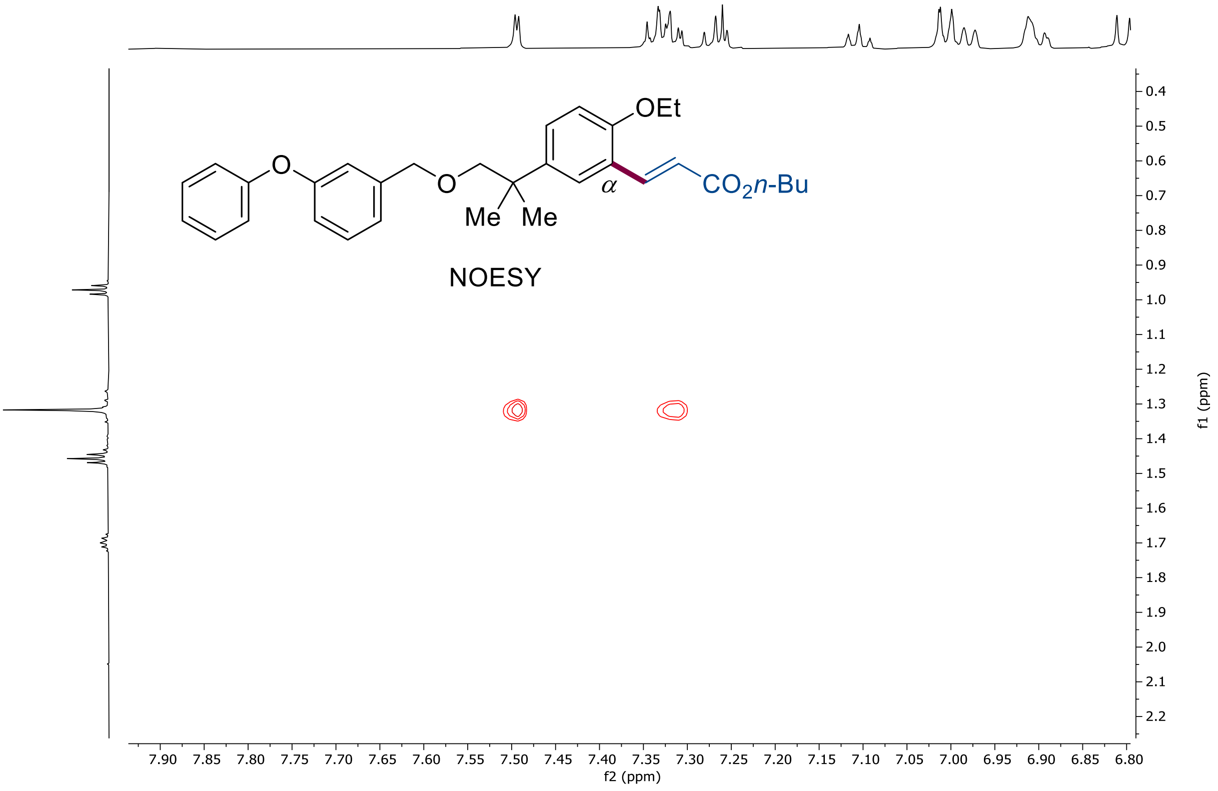

Supplementary Figure 247 NOESY-NMR of compound 68. $CDCl_3$, RT

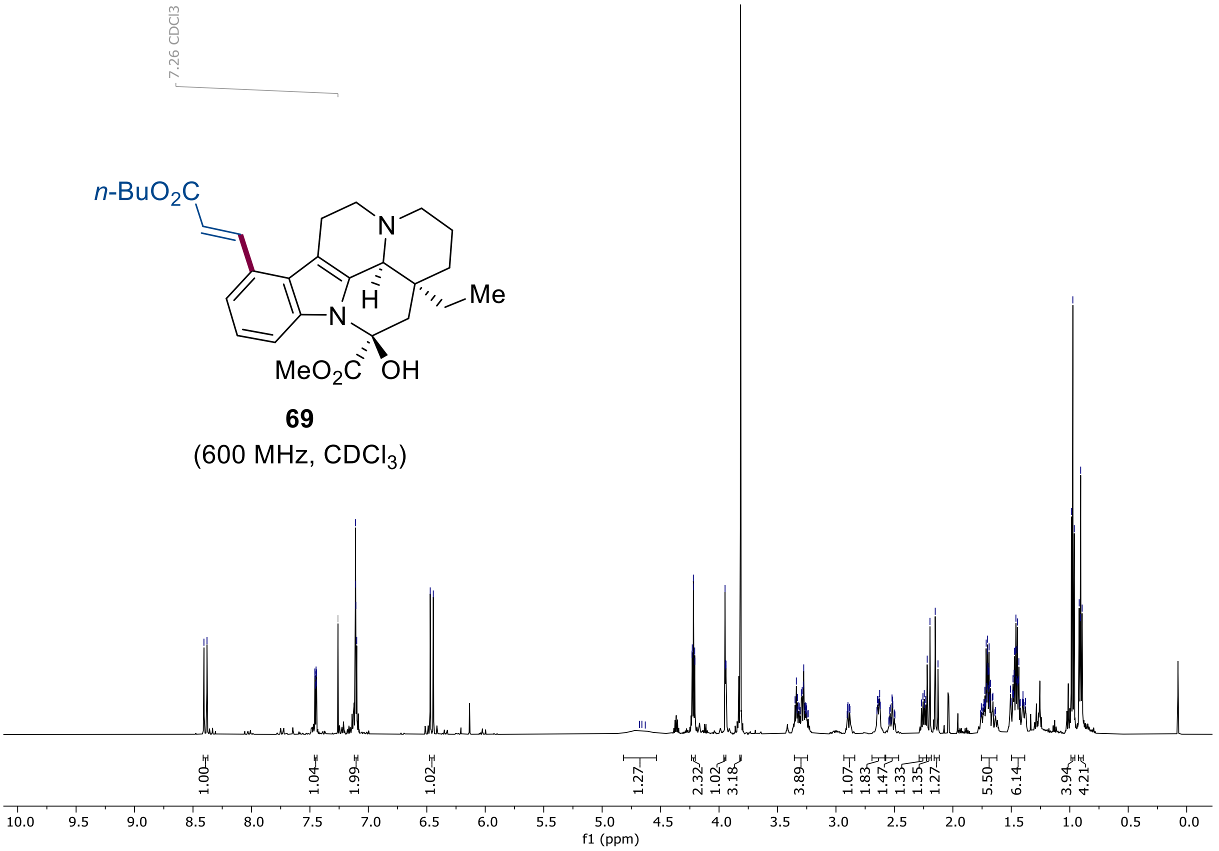

Supplementary Figure 248 H-NMR of compound 69. 600 MHz, $CDCl_3$, RT

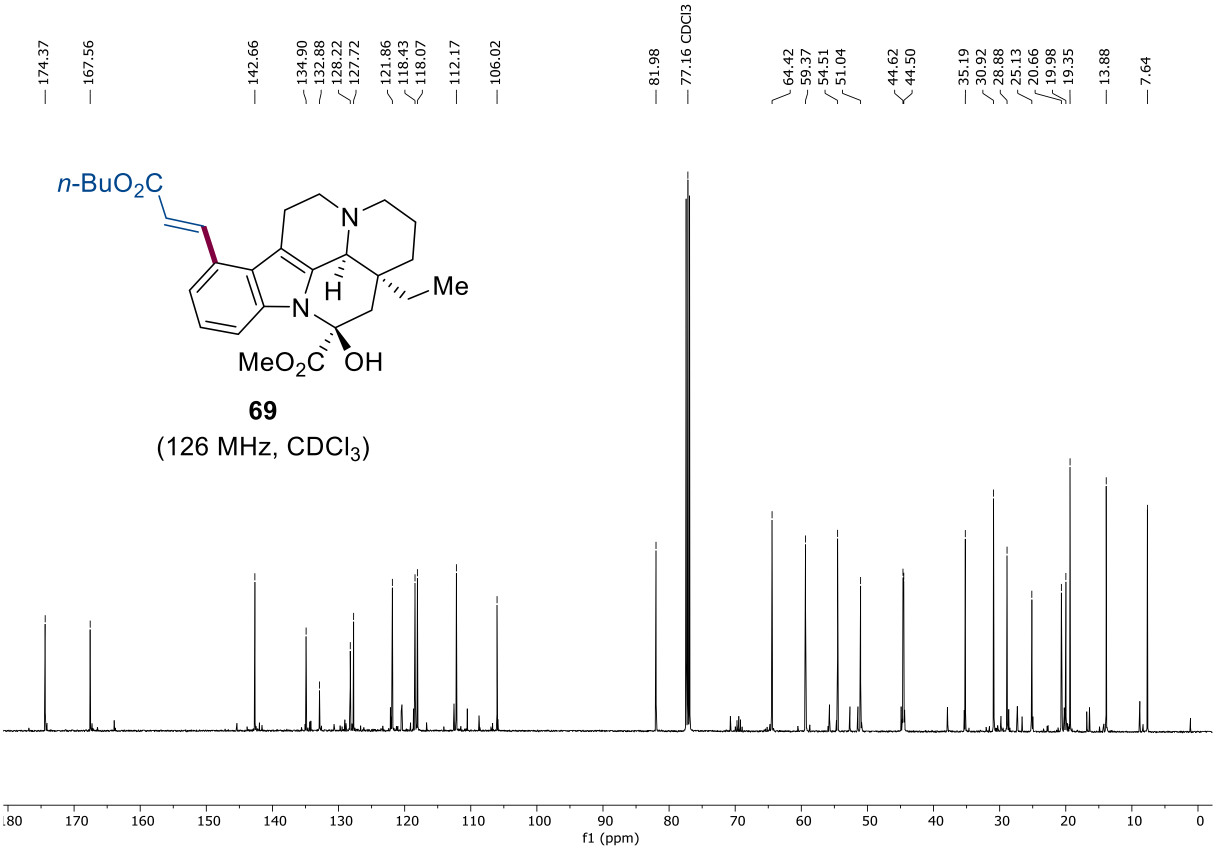

Supplementary Figure 249 C-NMR of compound 69. 126 MHz, CDCl₃, RT

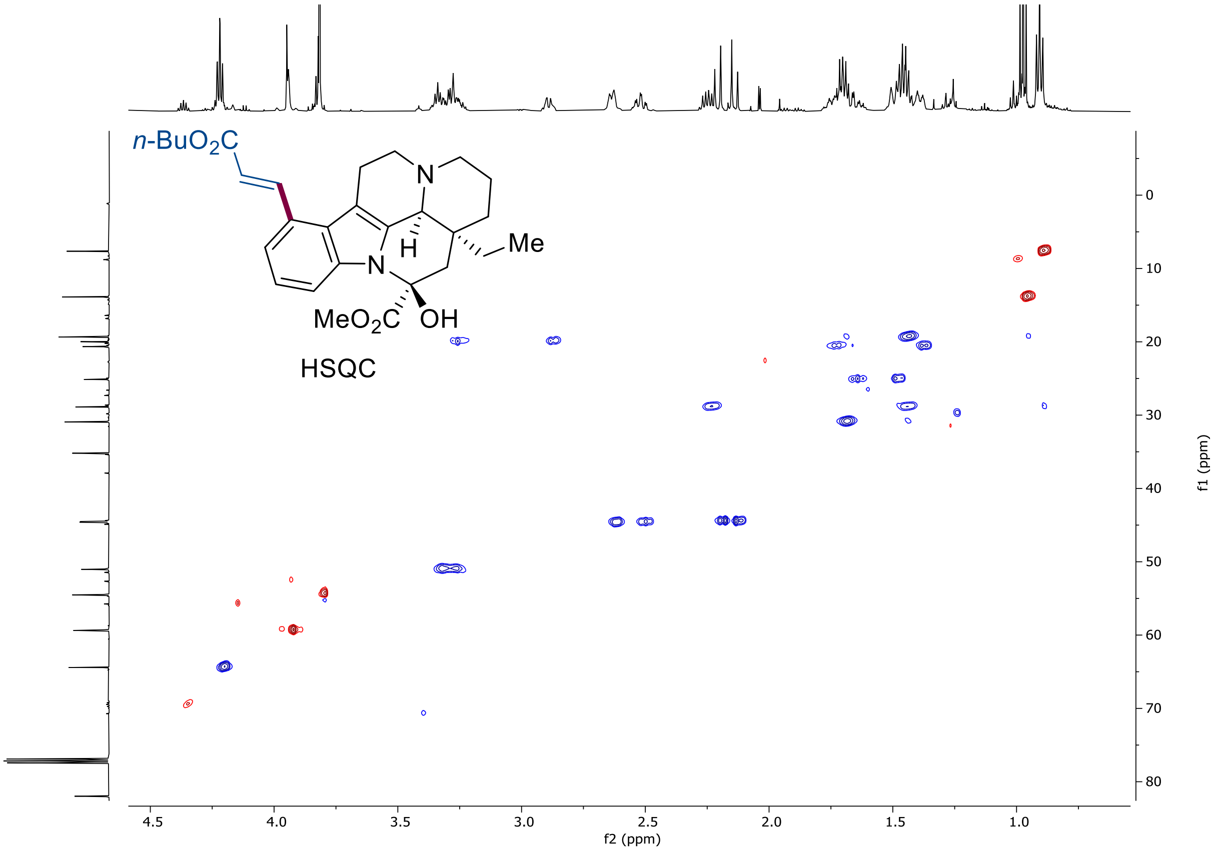

Supplementary Figure 250 HSQC-NMR of compound 69. CDCl₃, RT

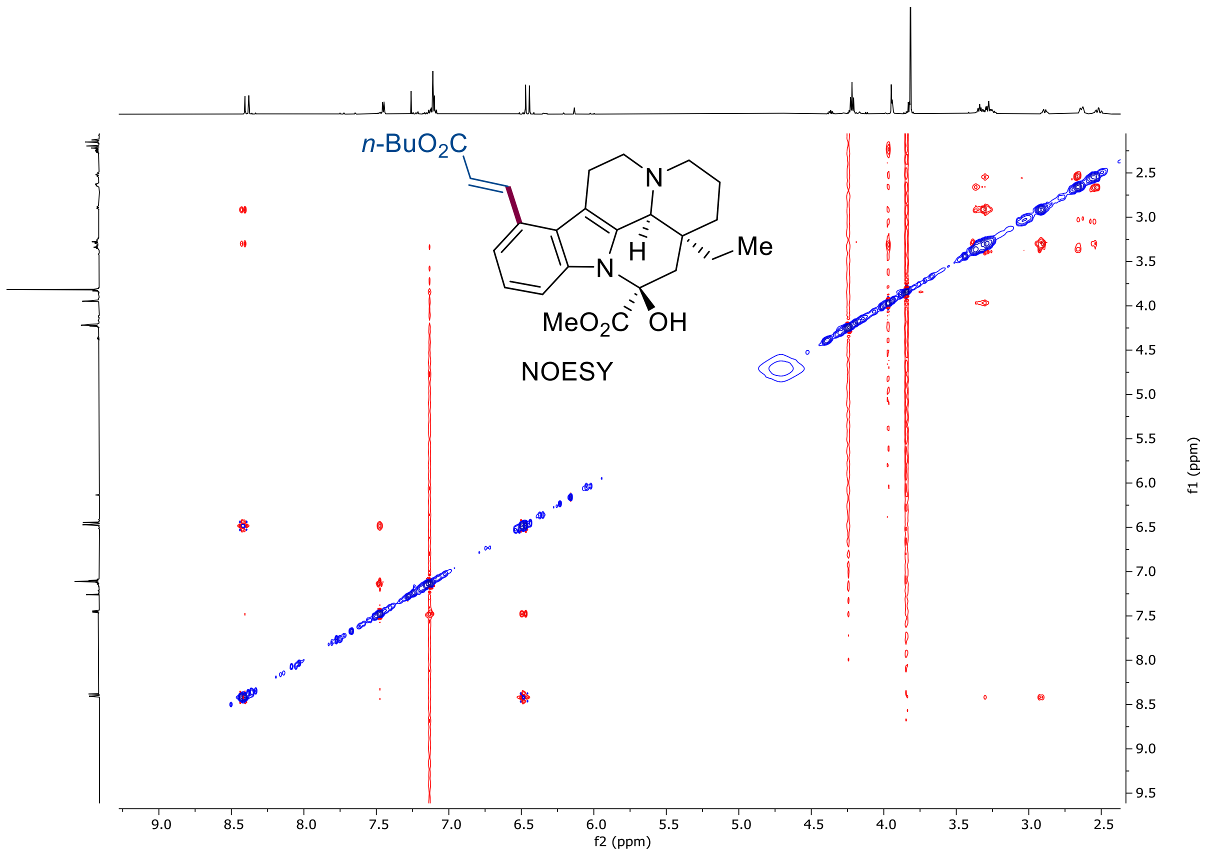

Supplementary Figure 251 NOESY-NMR of compound 69. $CDCl_3$, RT

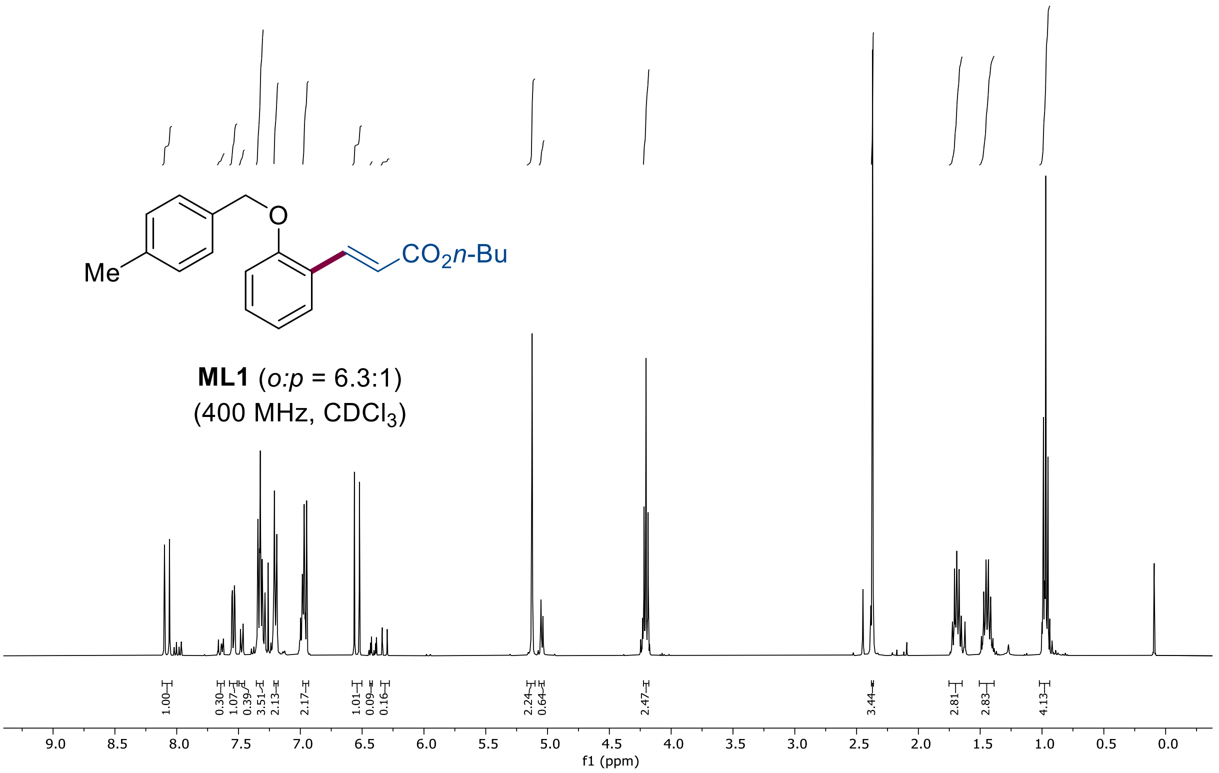

Supplementary Figure 252 H-NMR of compound ML1. 400 MHz, $CDCl_3$, RT

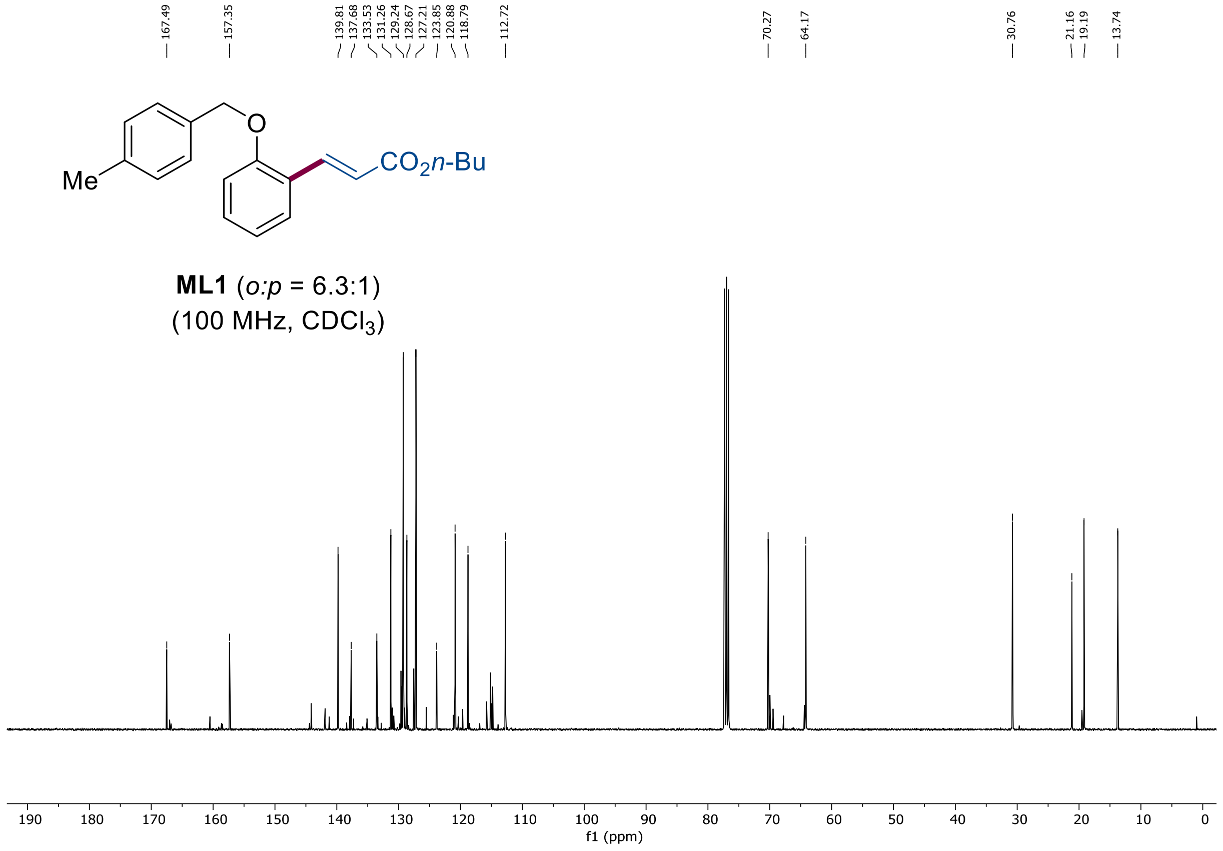

Supplementary Figure 253 C-NMR of compound ML1. 100 MHz, $CDCl_3$, RT

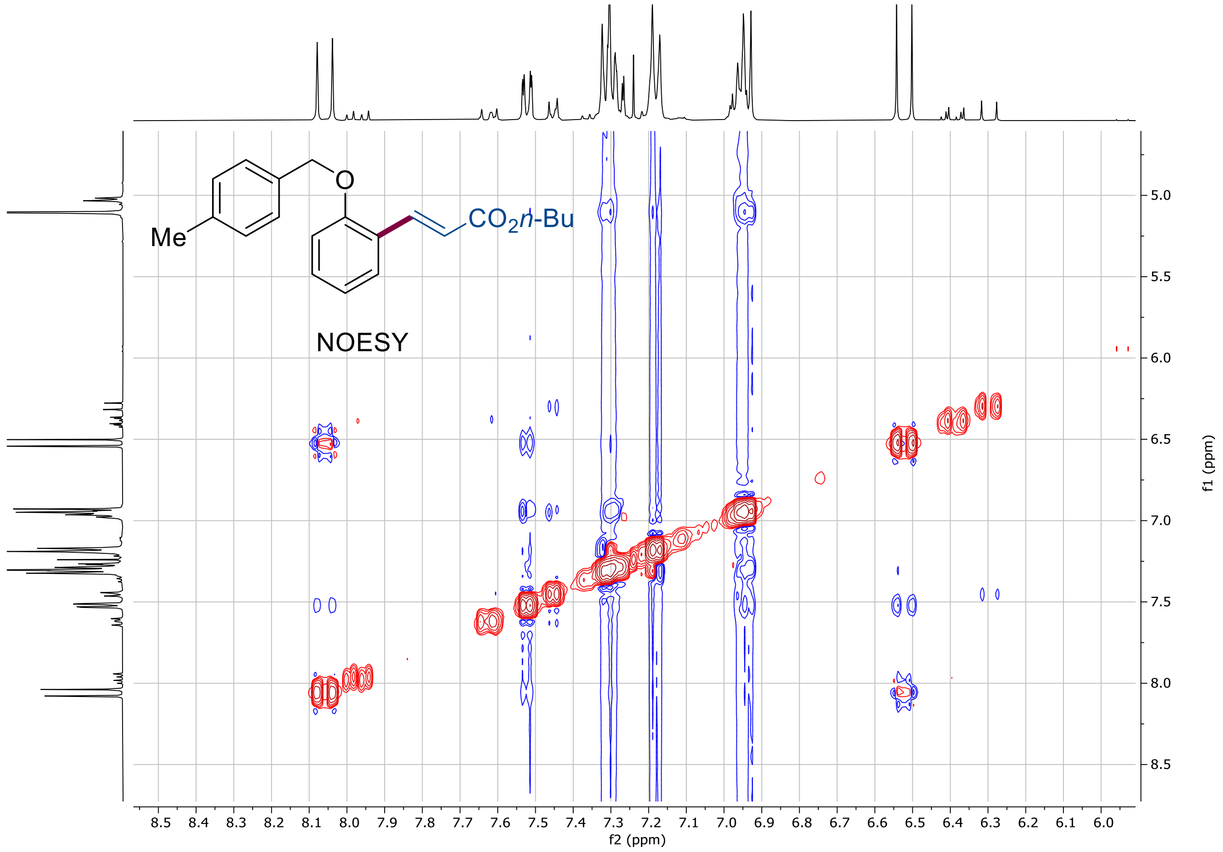

Supplementary Figure 254 NOESY-NMR of compound ML1. $CDCl_3$, RT

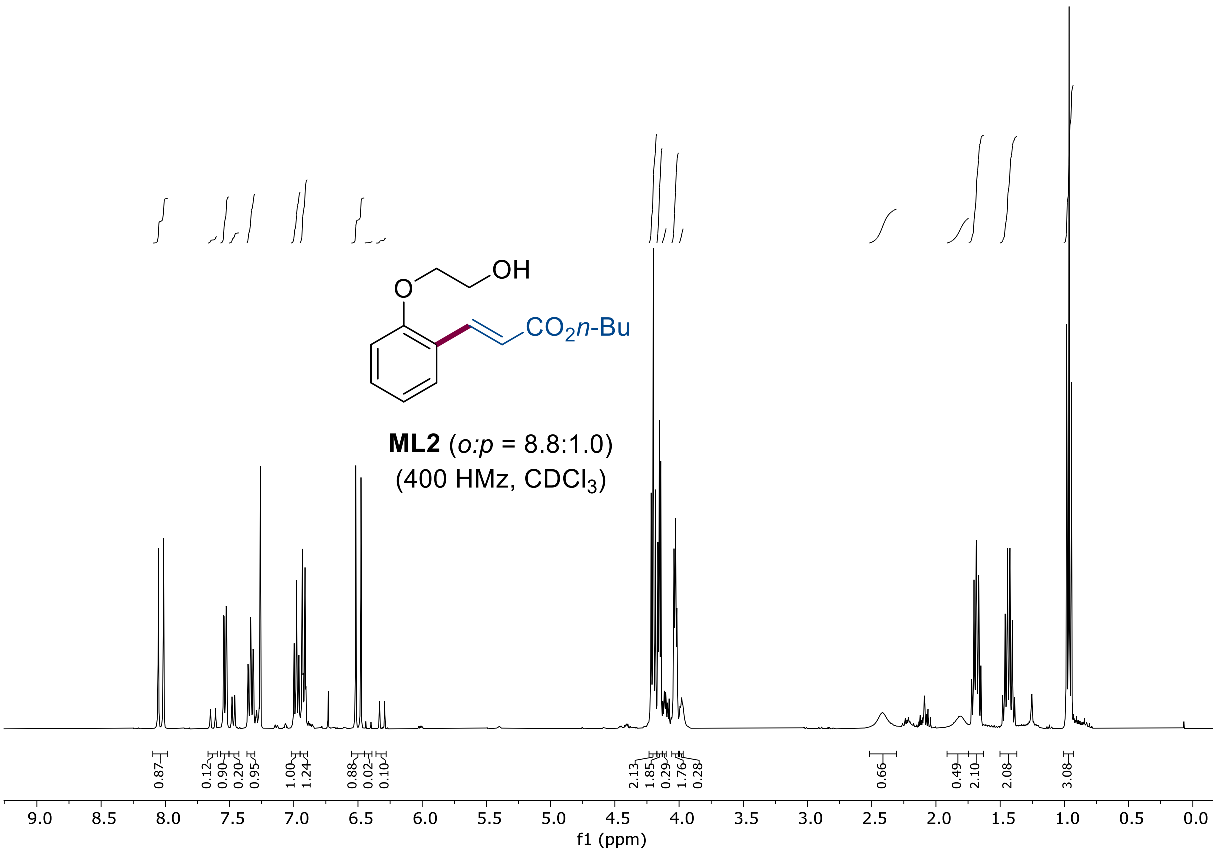

Supplementary Figure 255 H-NMR of compound ML2. 400 MHz, $CDCl_3$, RT

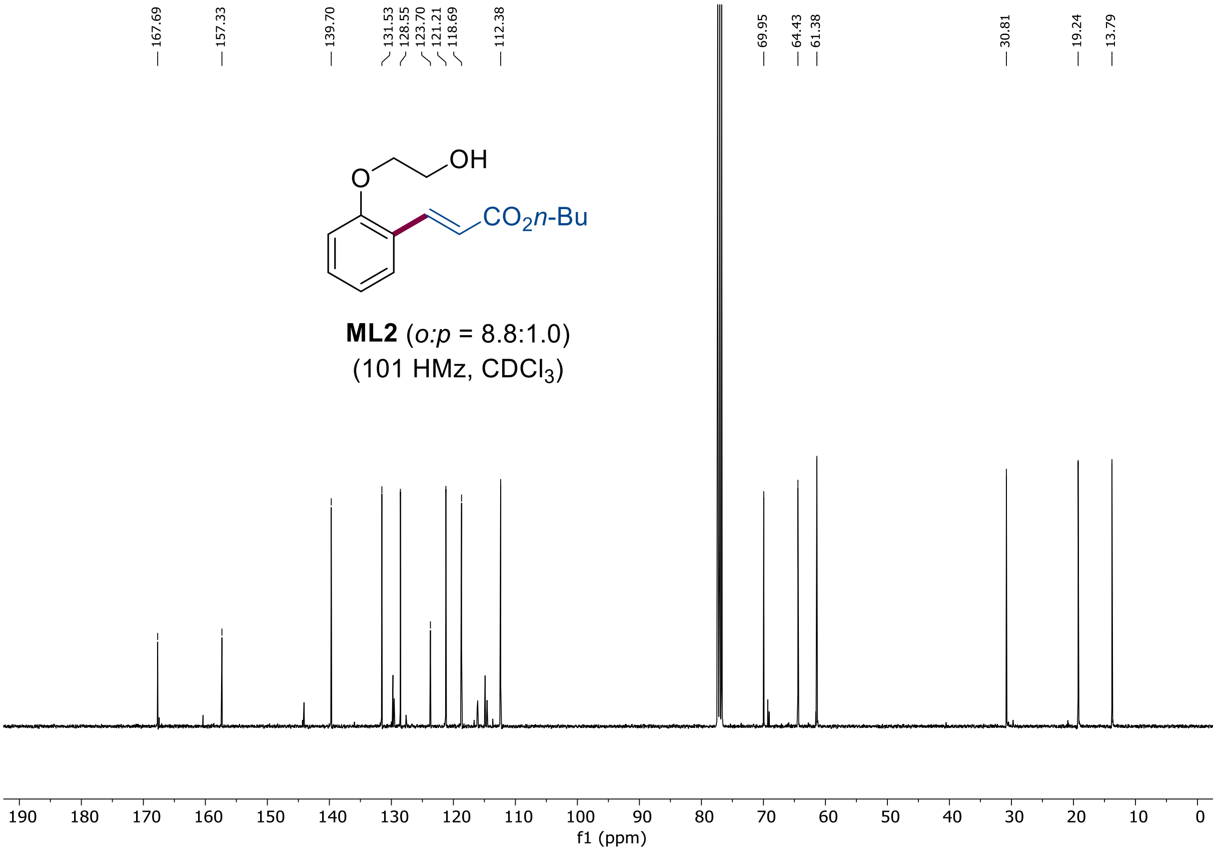

Supplementary Figure 256 C-NMR of compound ML2. 101 MHz, $CDCl_3$, RT

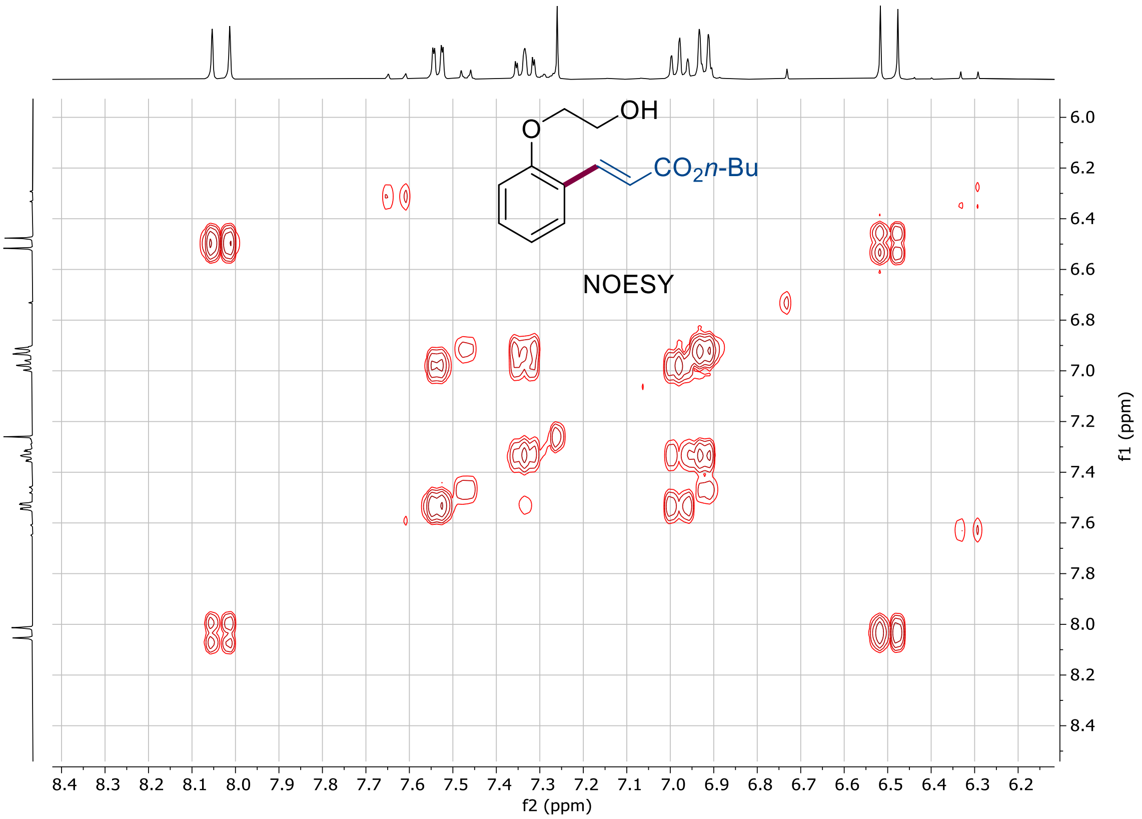

Supplementary Figure 257 NOESY-NMR of compound ML2. $CDCl_3$, RT

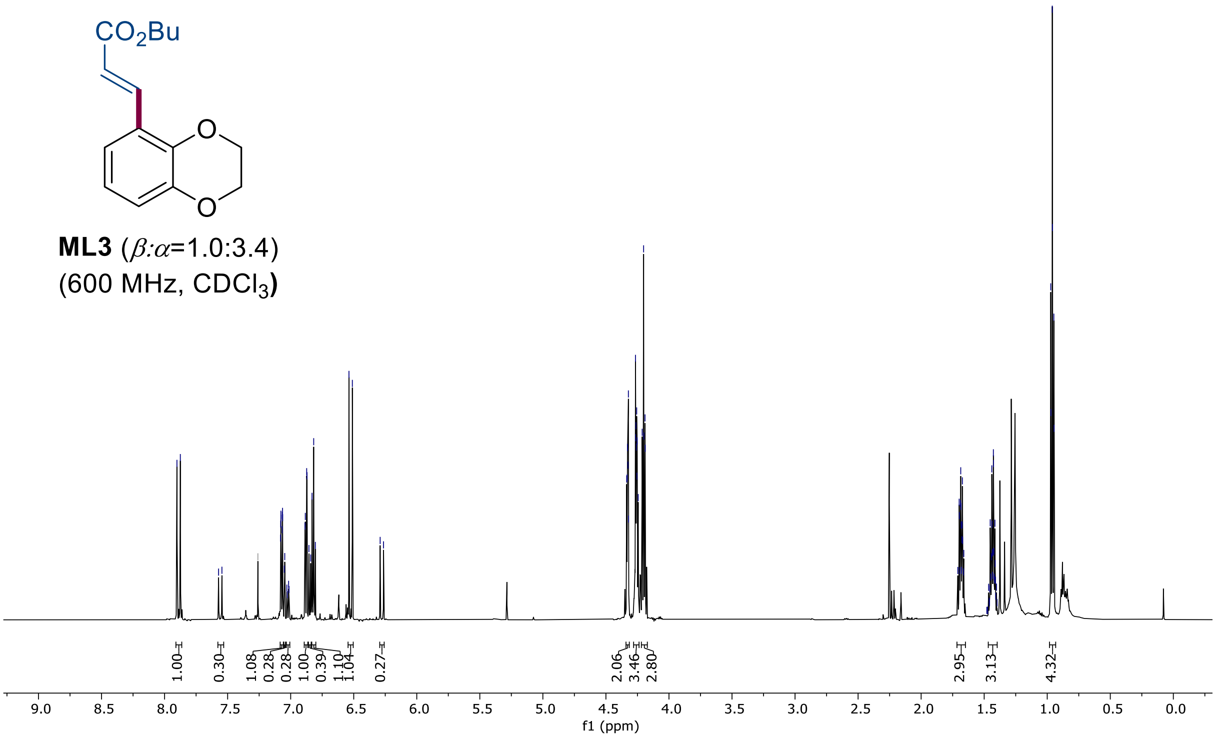

Supplementary Figure 258 H-NMR of compound ML3. 600 MHz, $CDCl_3$, RT

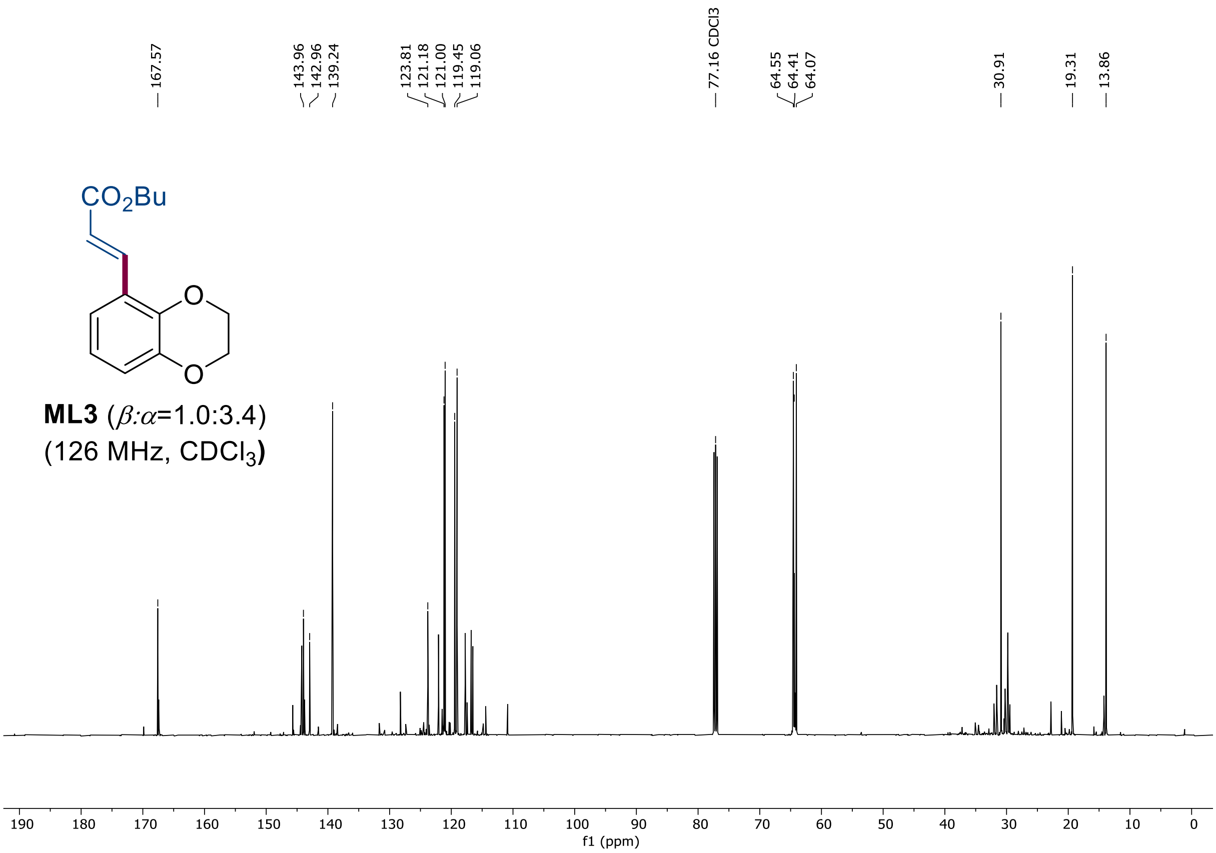

Supplementary Figure 259 C-NMR of compound ML3. 126 MHz, $CDCl_3$, RT

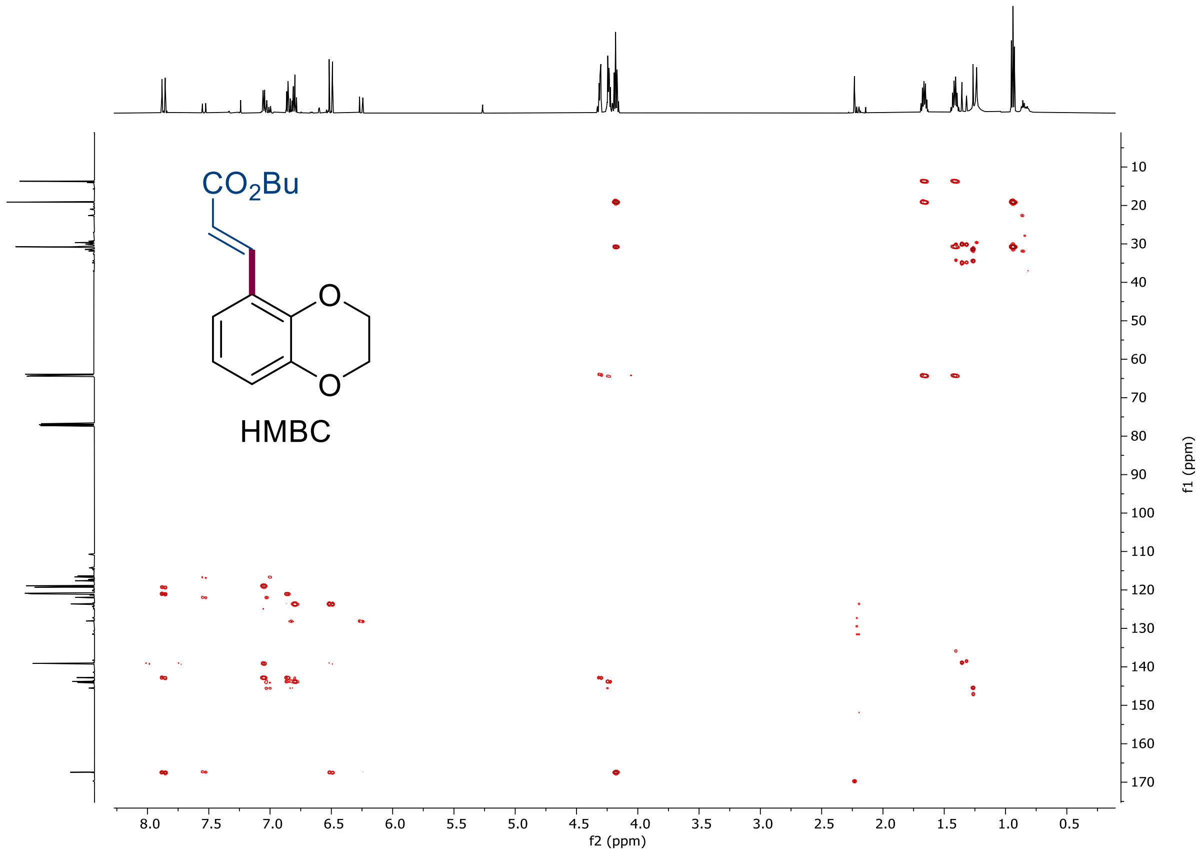

Supplementary Figure 260 HMBC-NMR of compound ML3. $CDCl_3$, RT

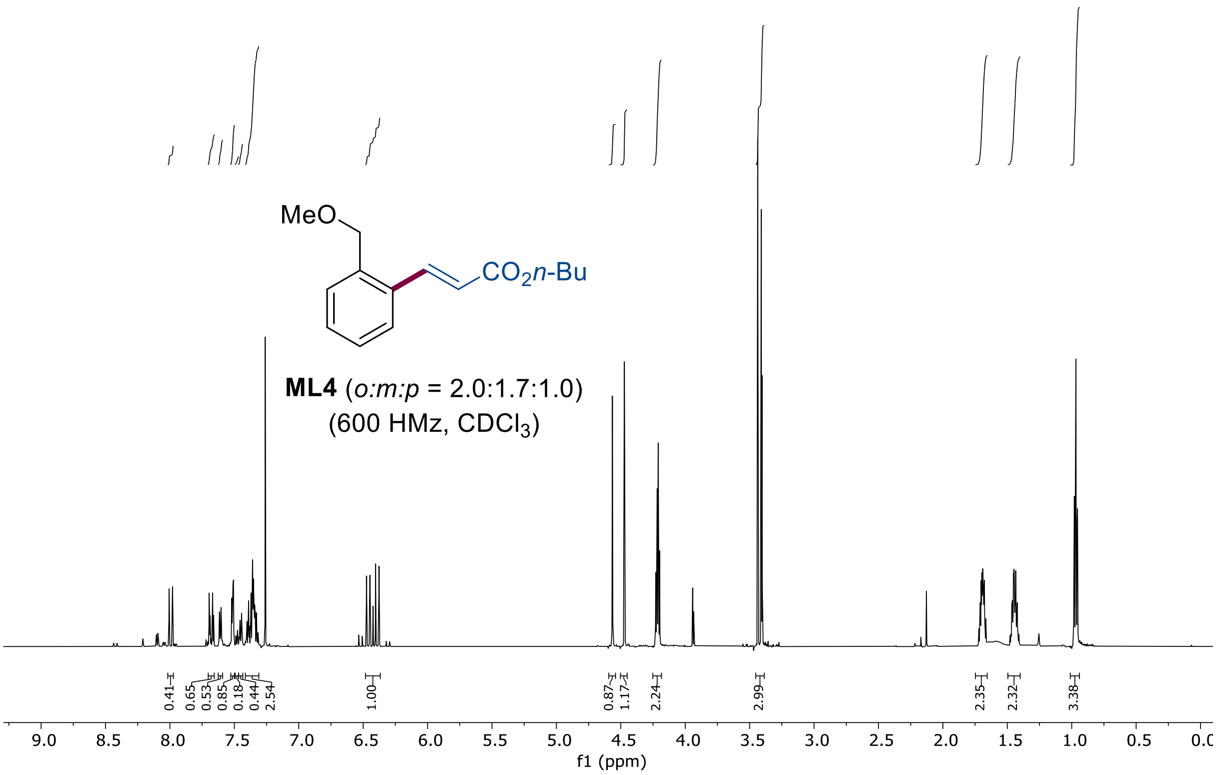

Supplementary Figure 261 H-NMR of compound ML4. 600 MHz, $CDCl_3$, RT

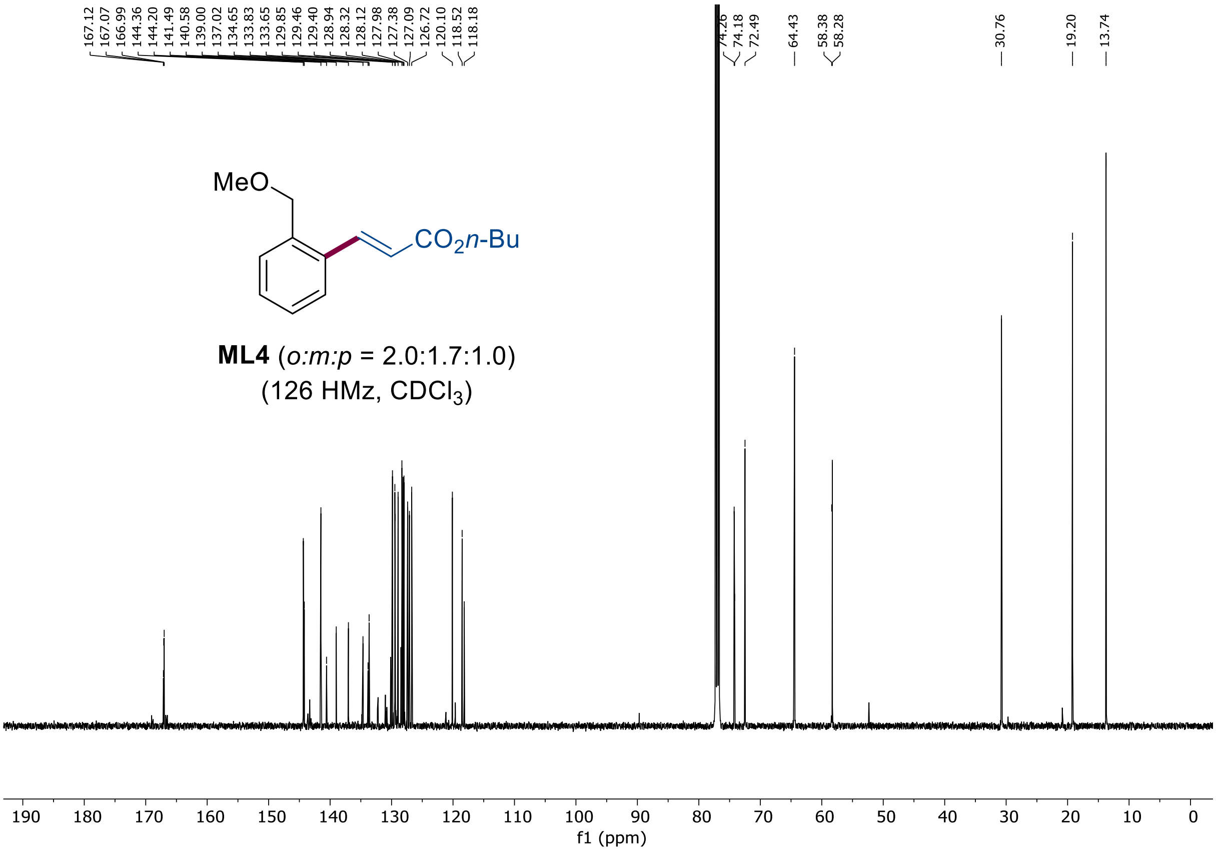

Supplementary Figure 262 C-NMR of compound ML4. 126 MHz, $CDCl_3$, RT

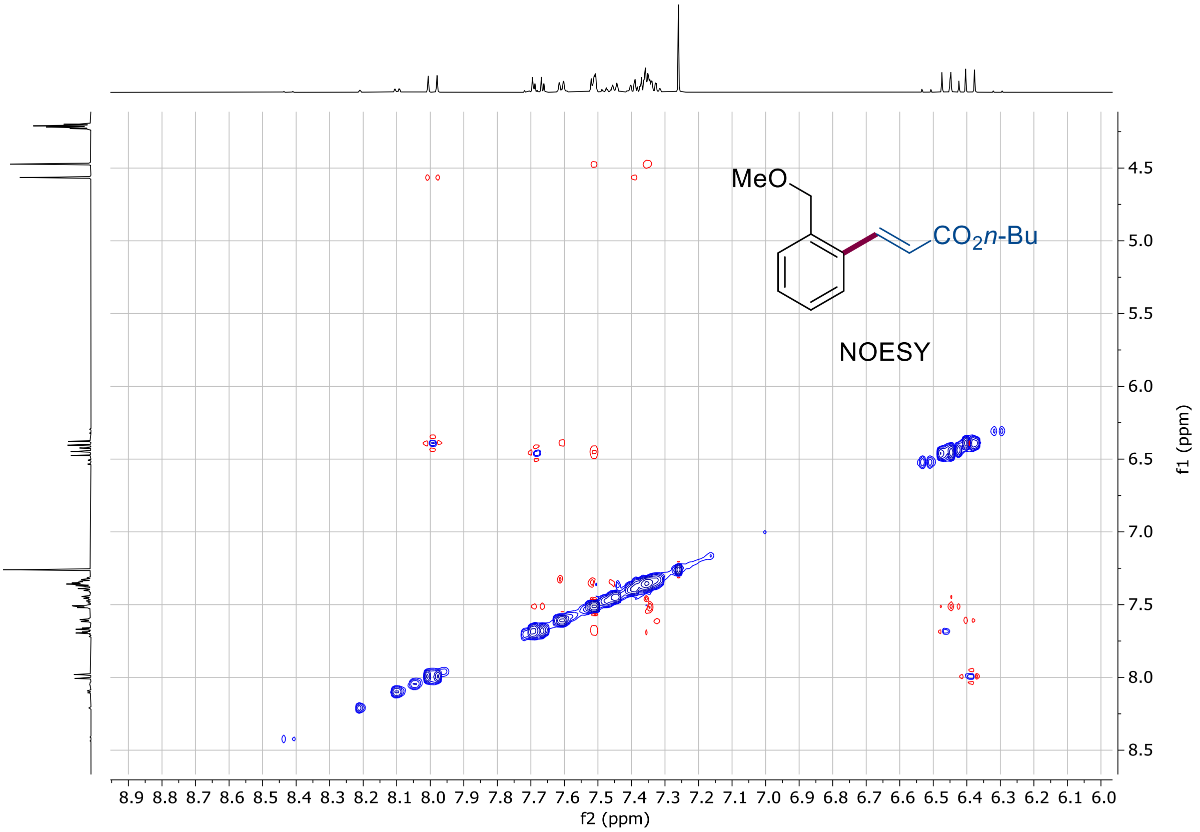

Supplementary Figure 263 NOESY-NMR of compound ML4. $CDCl_3$, RT

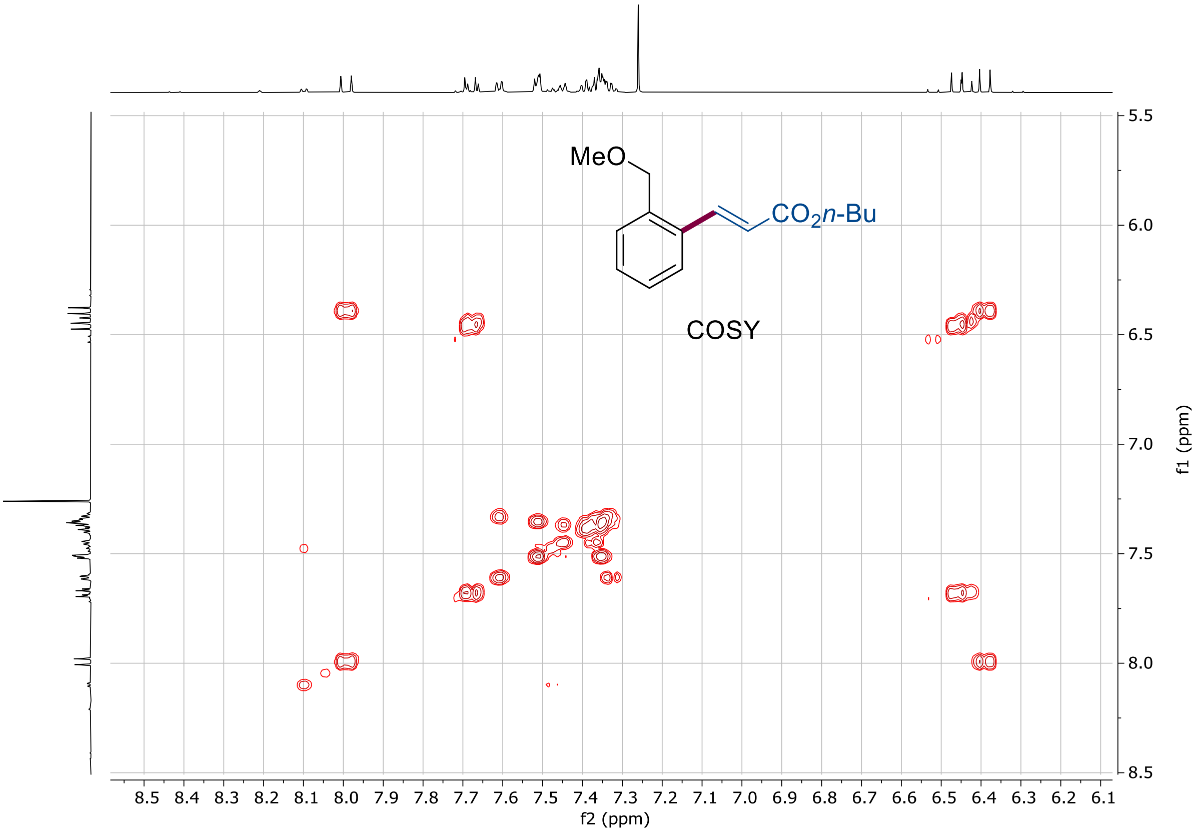

Supplementary Figure 264 COSY-NMR of compound ML4. $CDCl_3$, RT

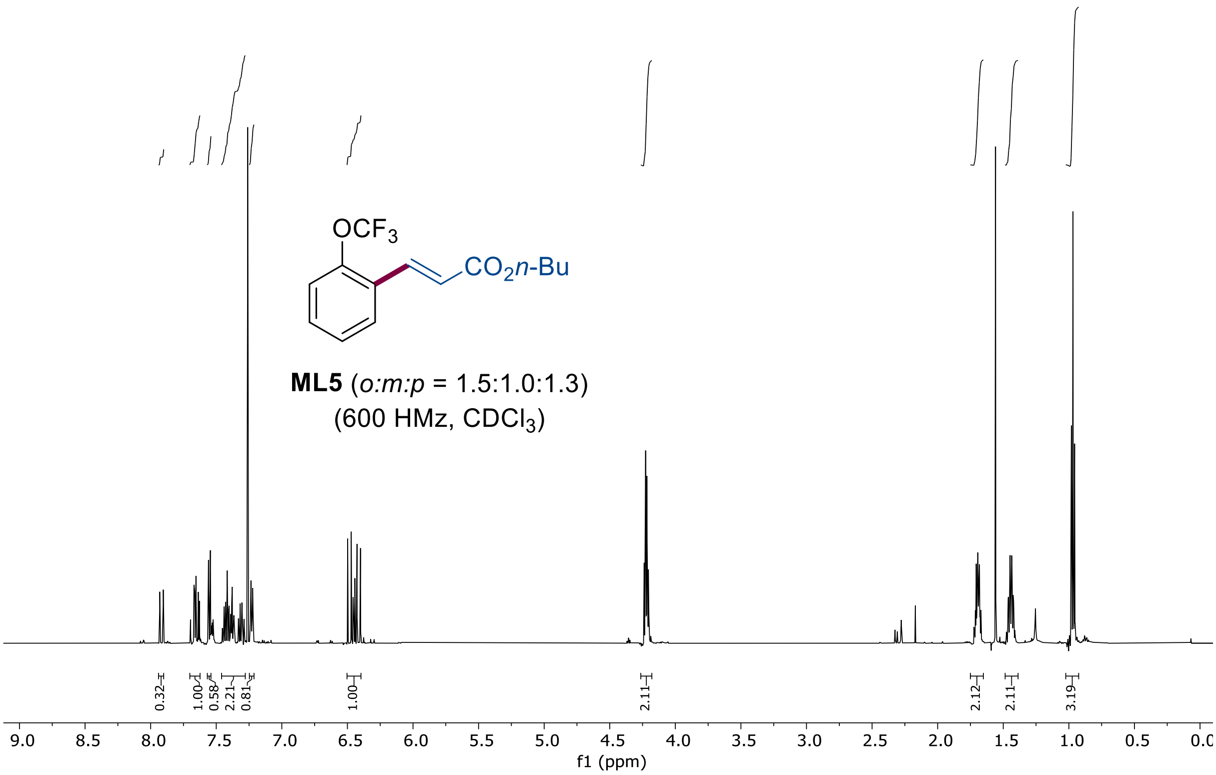

Supplementary Figure 265 H-NMR of compound ML5. 600 MHz, $CDCl_3$, RT

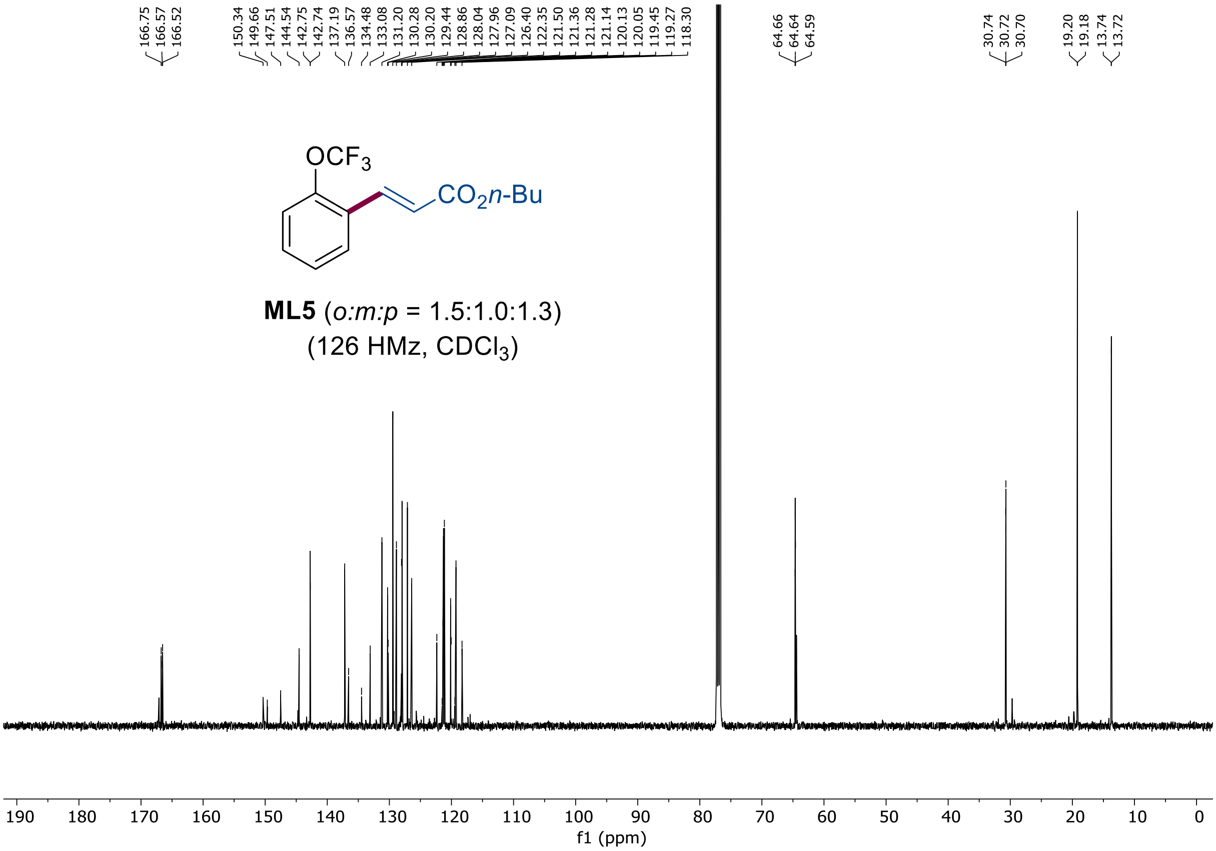

Supplementary Figure 266 C-NMR of compound ML5. 126 MHz, $CDCl_3$, RT

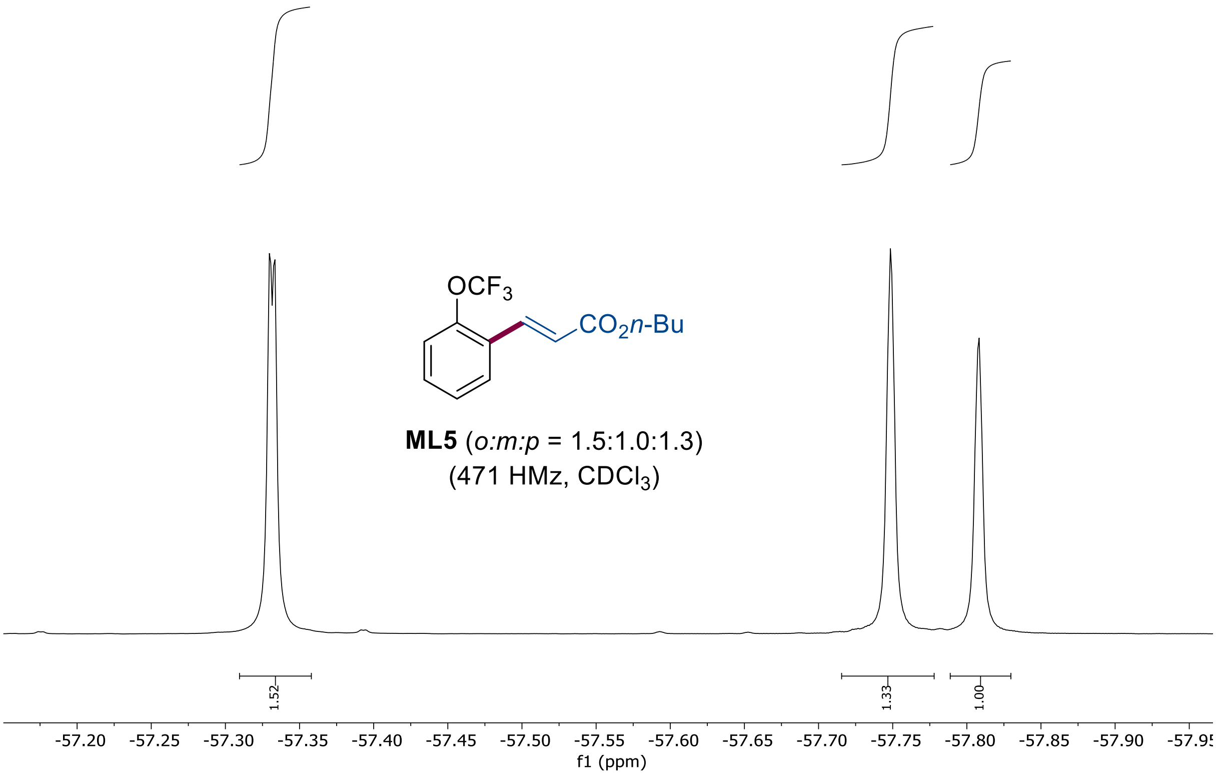

Supplementary Figure 267 F-NMR of compound ML5. 471 MHz, $CDCl_3$, RT

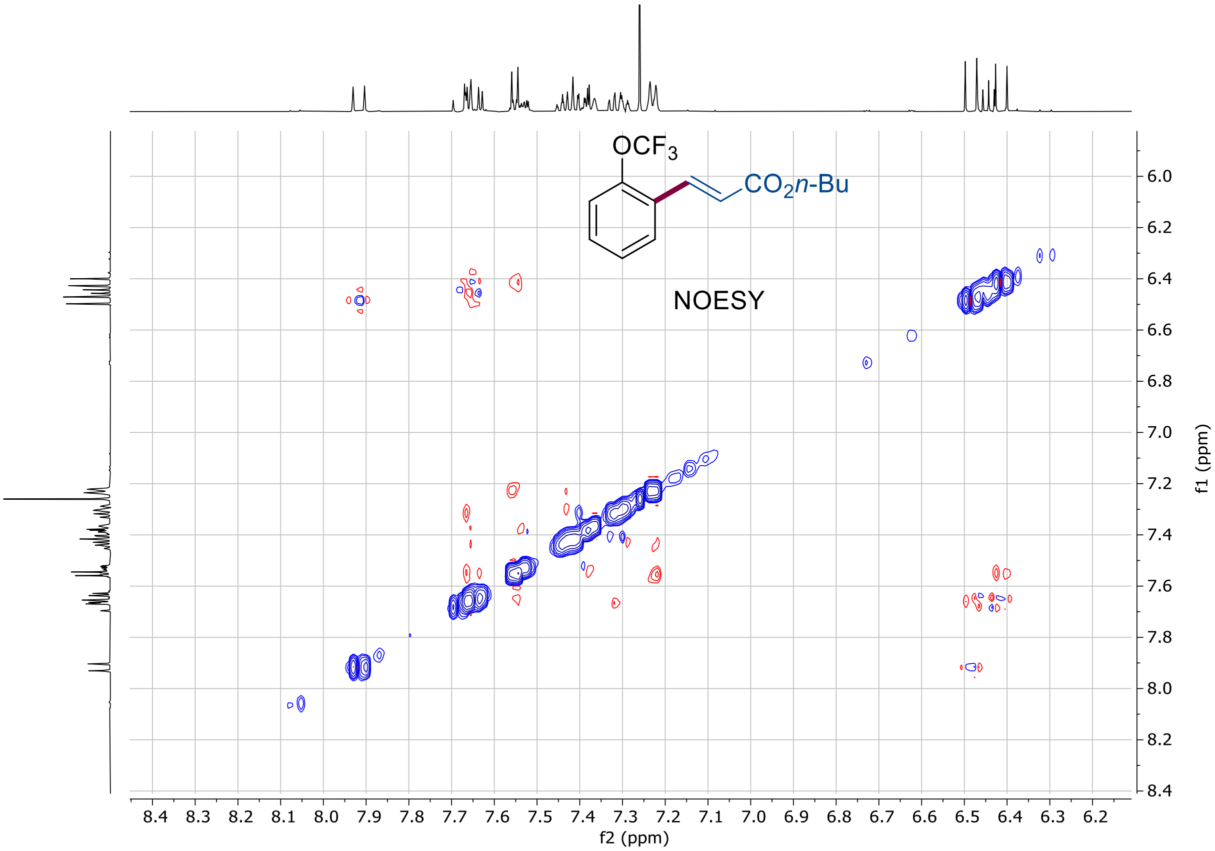

Supplementary Figure 268 NOESY-NMR of compound ML5. $CDCl_3$, RT

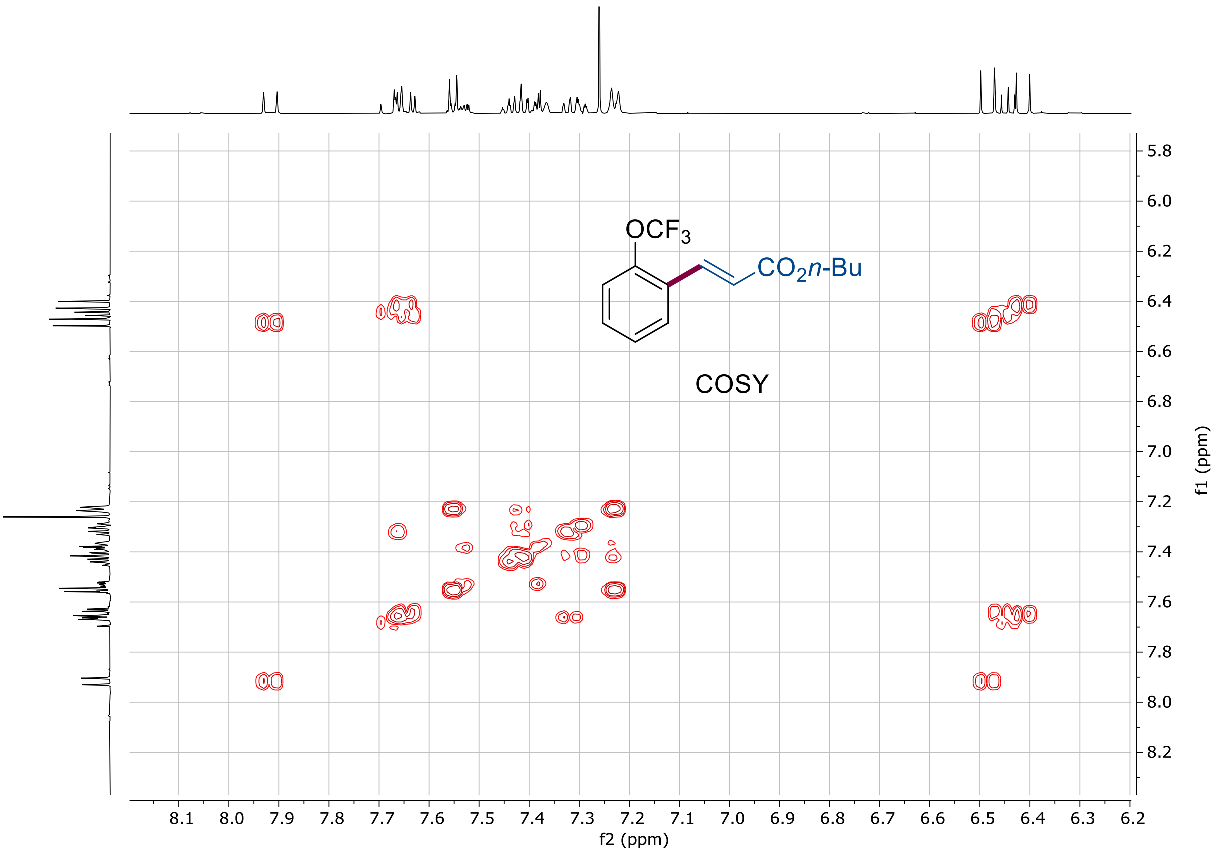

Supplementary Figure 269 **COSY-NMR of compound ML5. $CDCl_3$, RT**

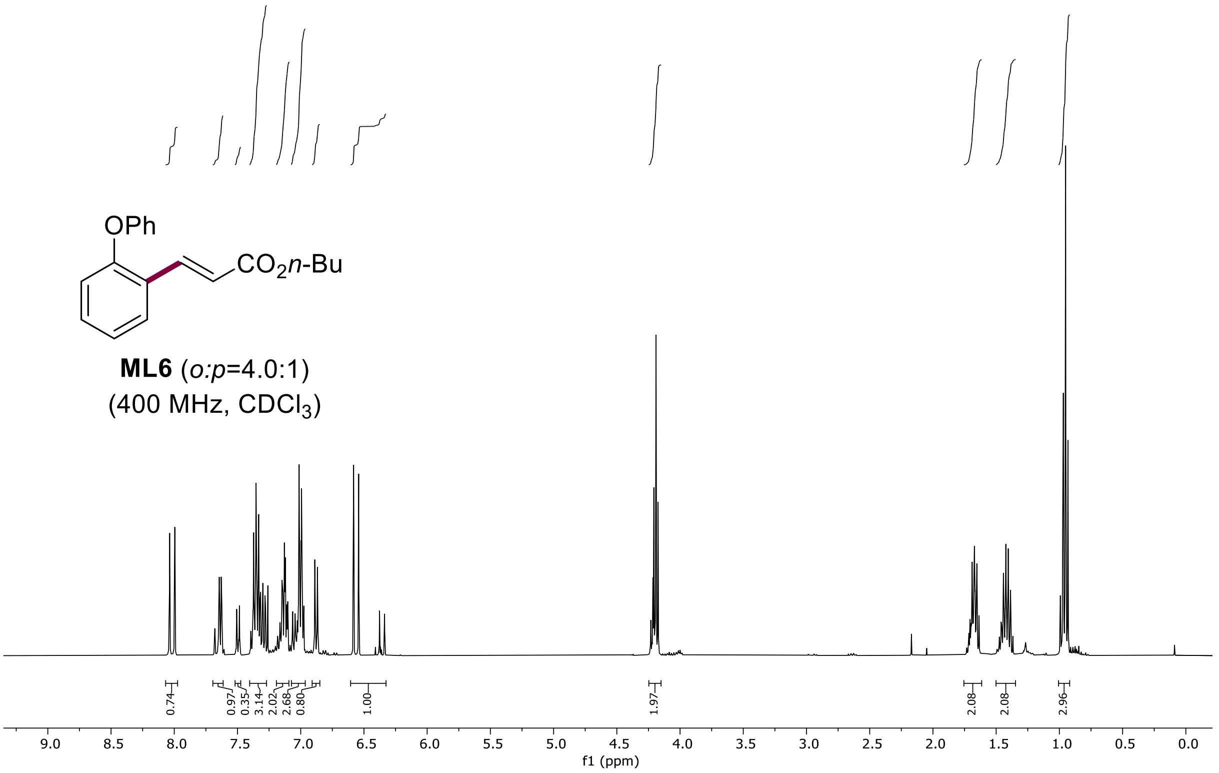

Supplementary Figure 270 **H-NMR of compound ML6. 400 MHz, $CDCl_3$, RT**

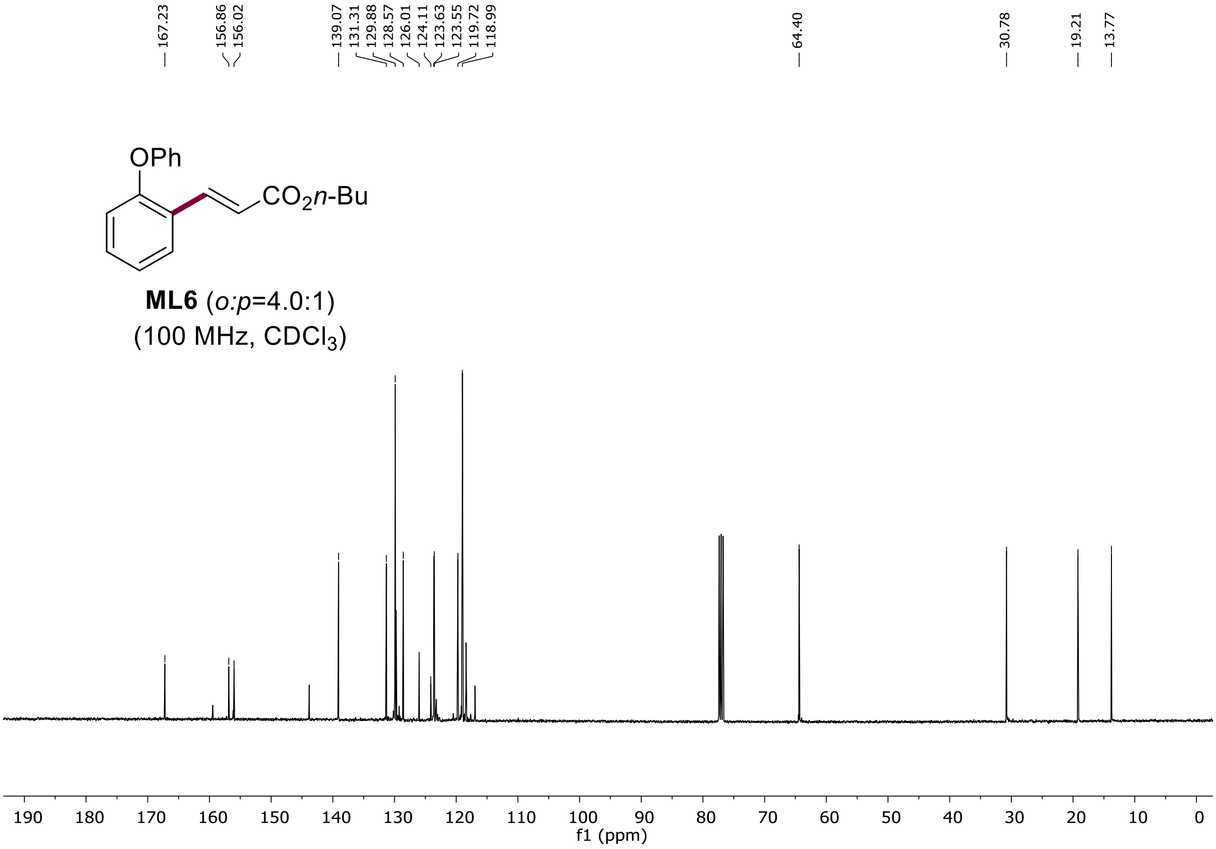

Supplementary Figure 271 C-NMR of compound ML6. 100 MHz, $CDCl_3$, RT

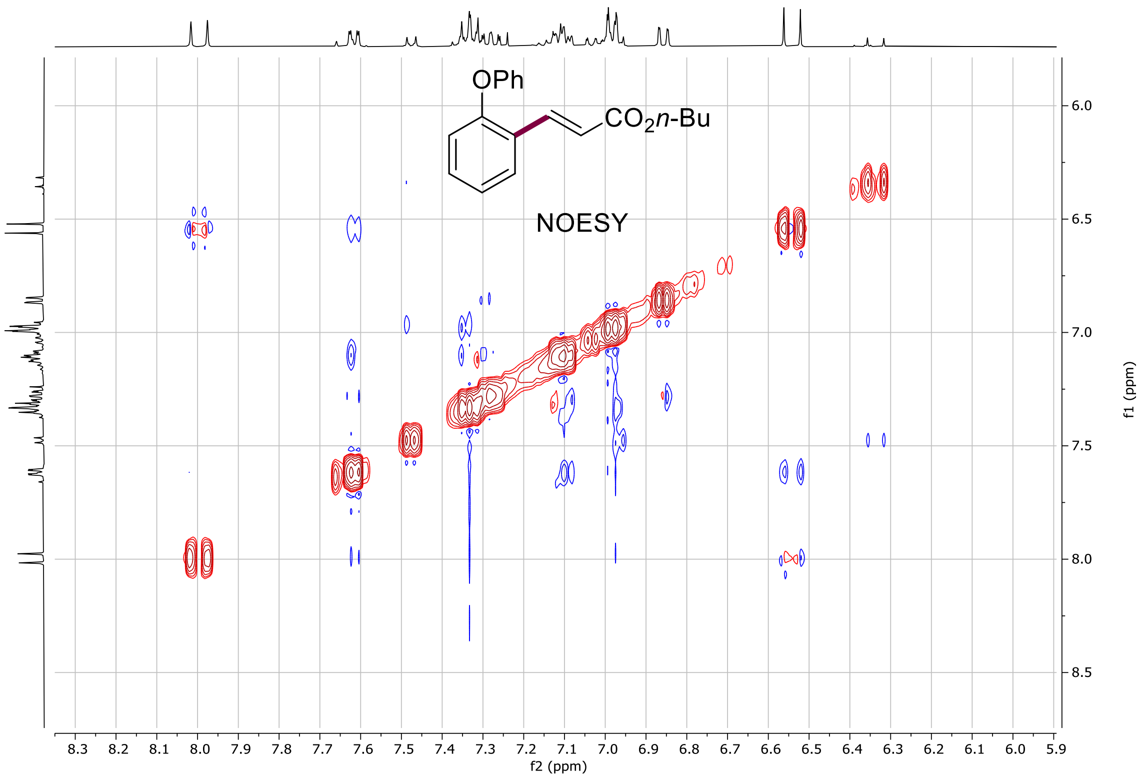

Supplementary Figure 272 NOESY-NMR of compound ML6. $CDCl_3$, RT

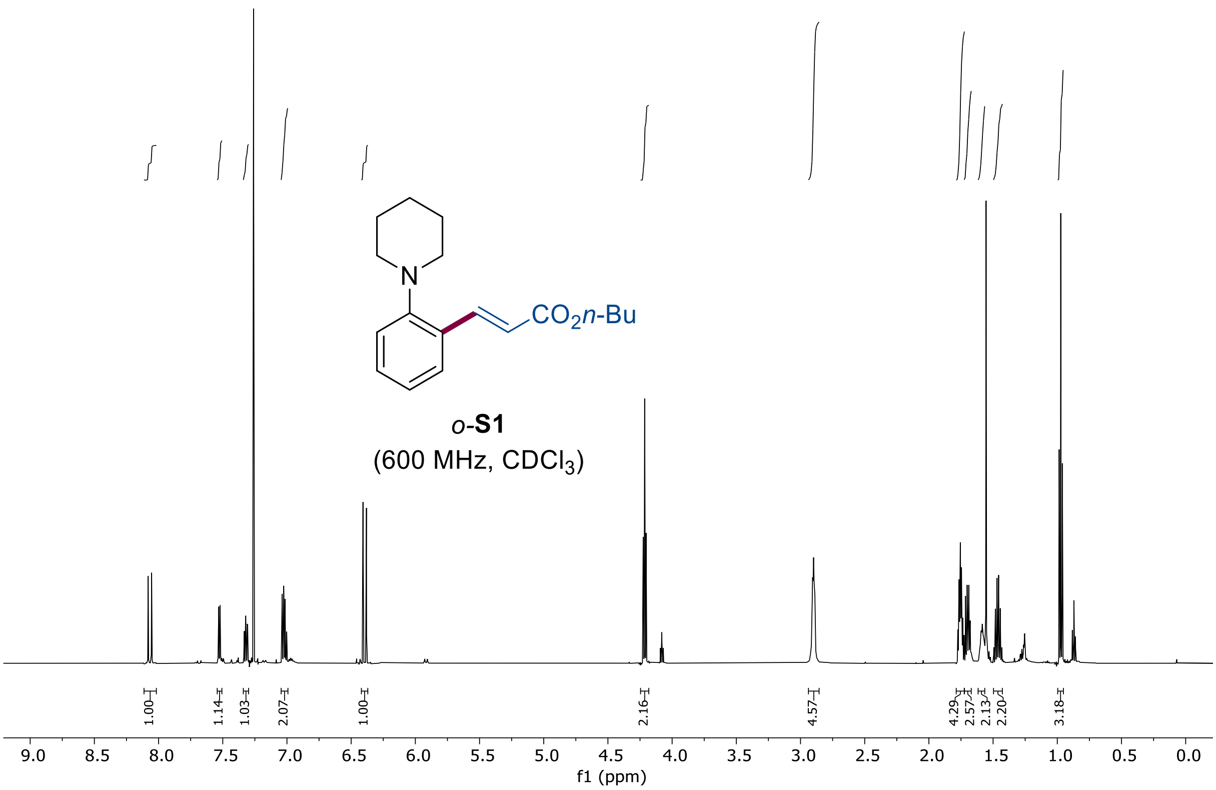

Supplementary Figure 273 H-NMR of compound ***o*-S1**. 600 MHz, $CDCl_3$, RT

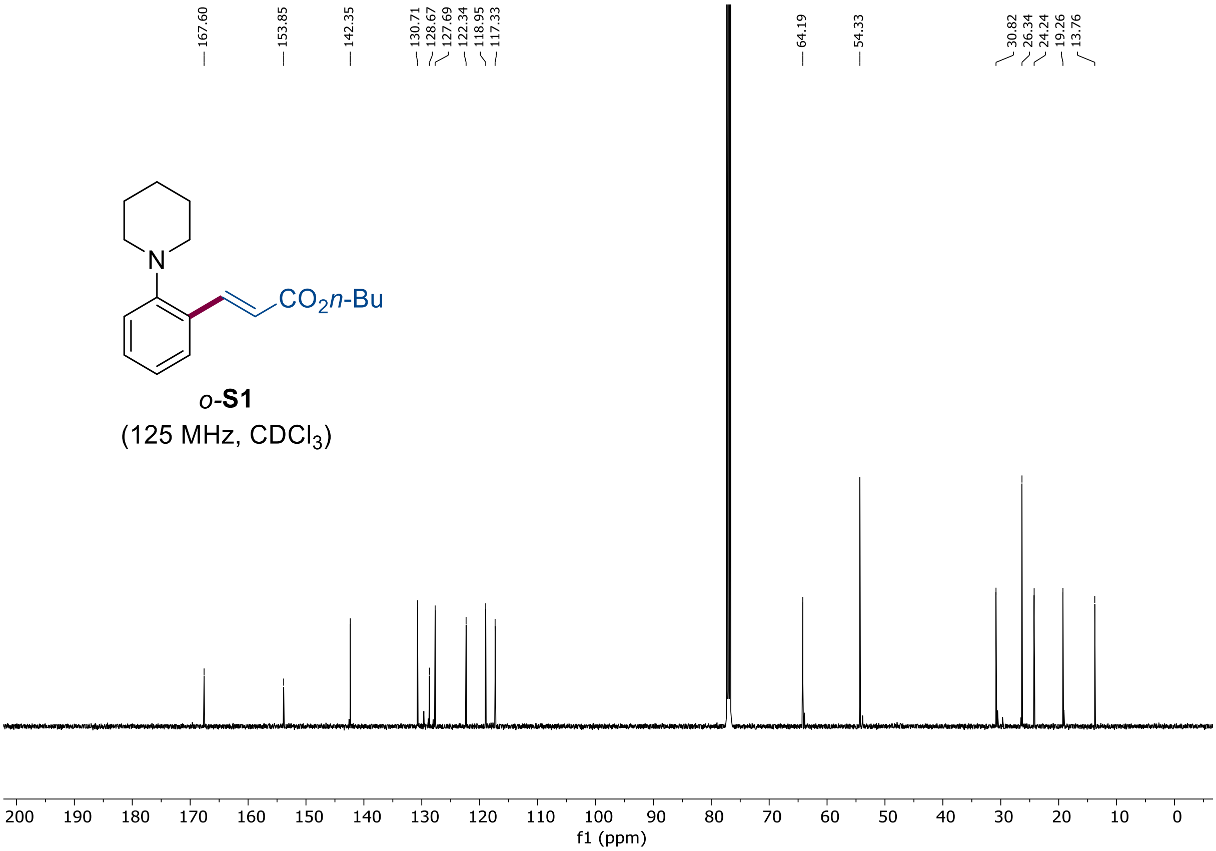

Supplementary Figure 274 C-NMR of compound ***o*-S1**. 125 MHz, $CDCl_3$, RT

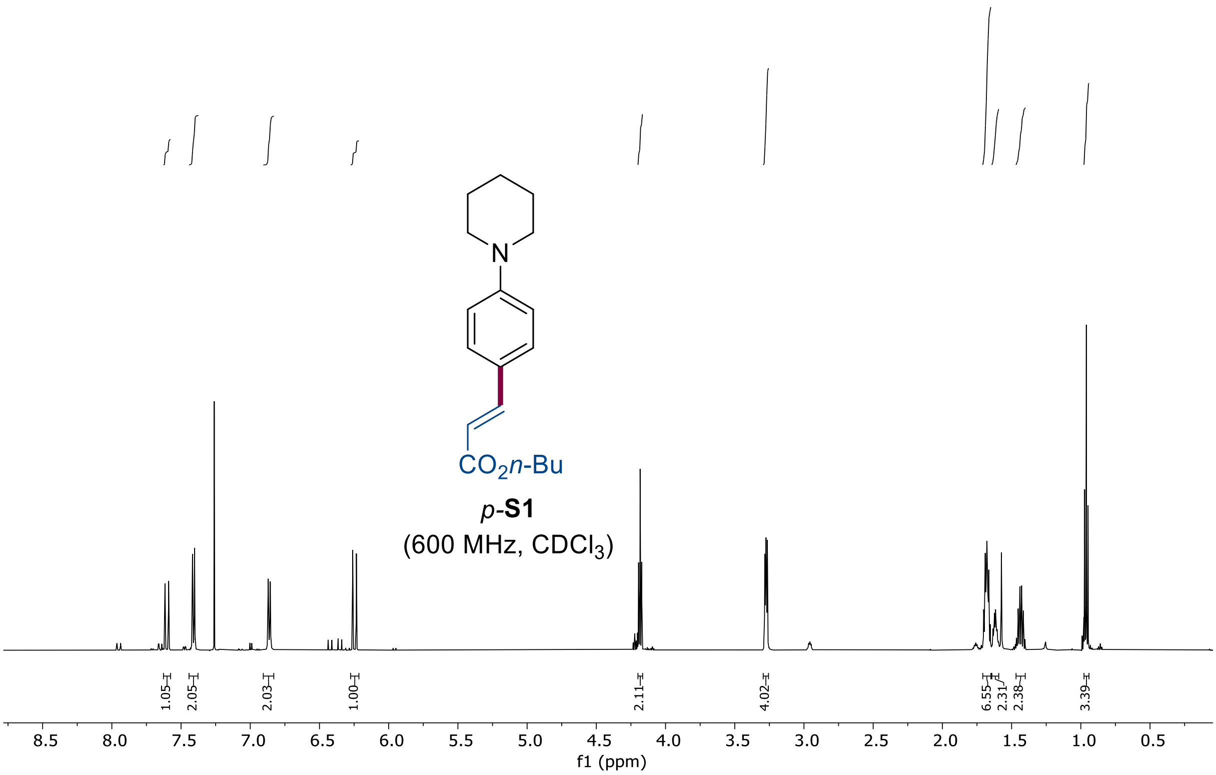

Supplementary Figure 275 H-NMR of compound *p*-S1. 600 MHz, $CDCl_3$, RT

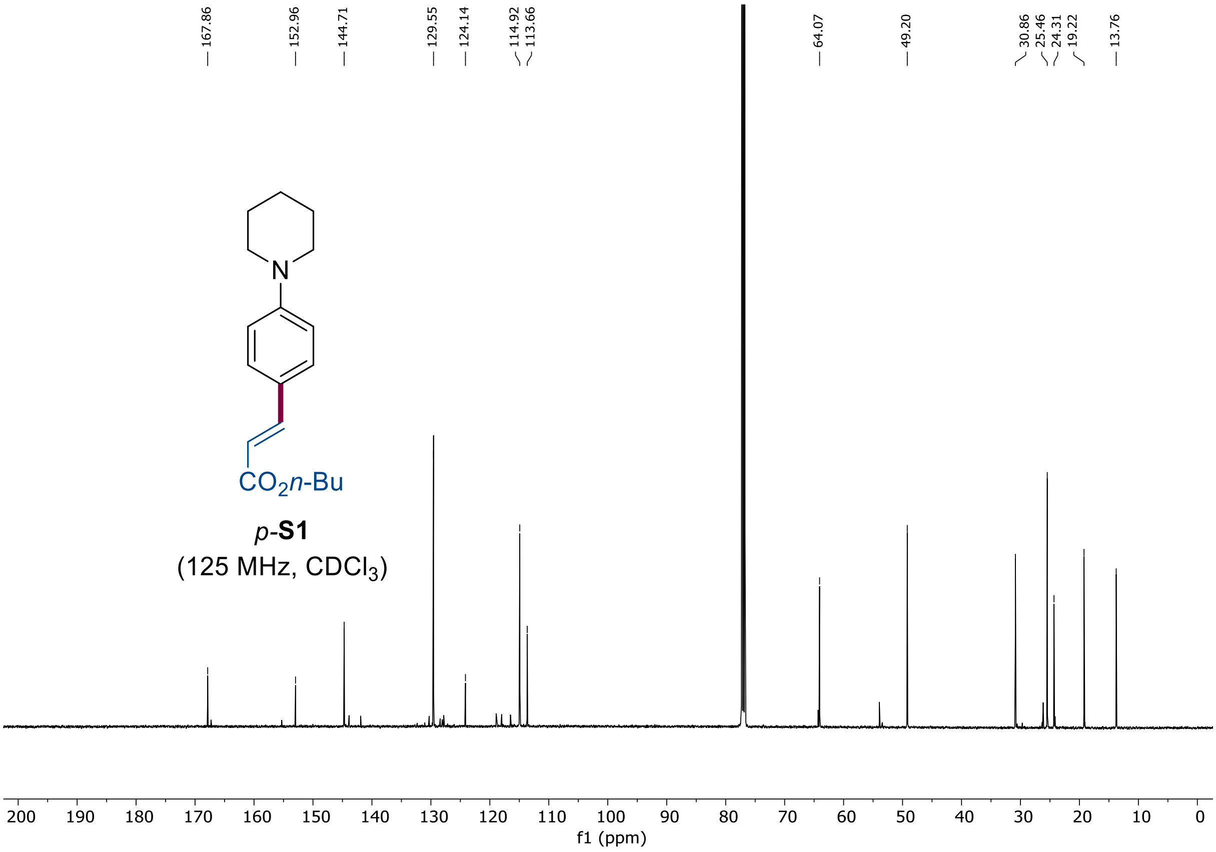

Supplementary Figure 276 C-NMR of compound *p*-S1. 125 MHz, $CDCl_3$, RT

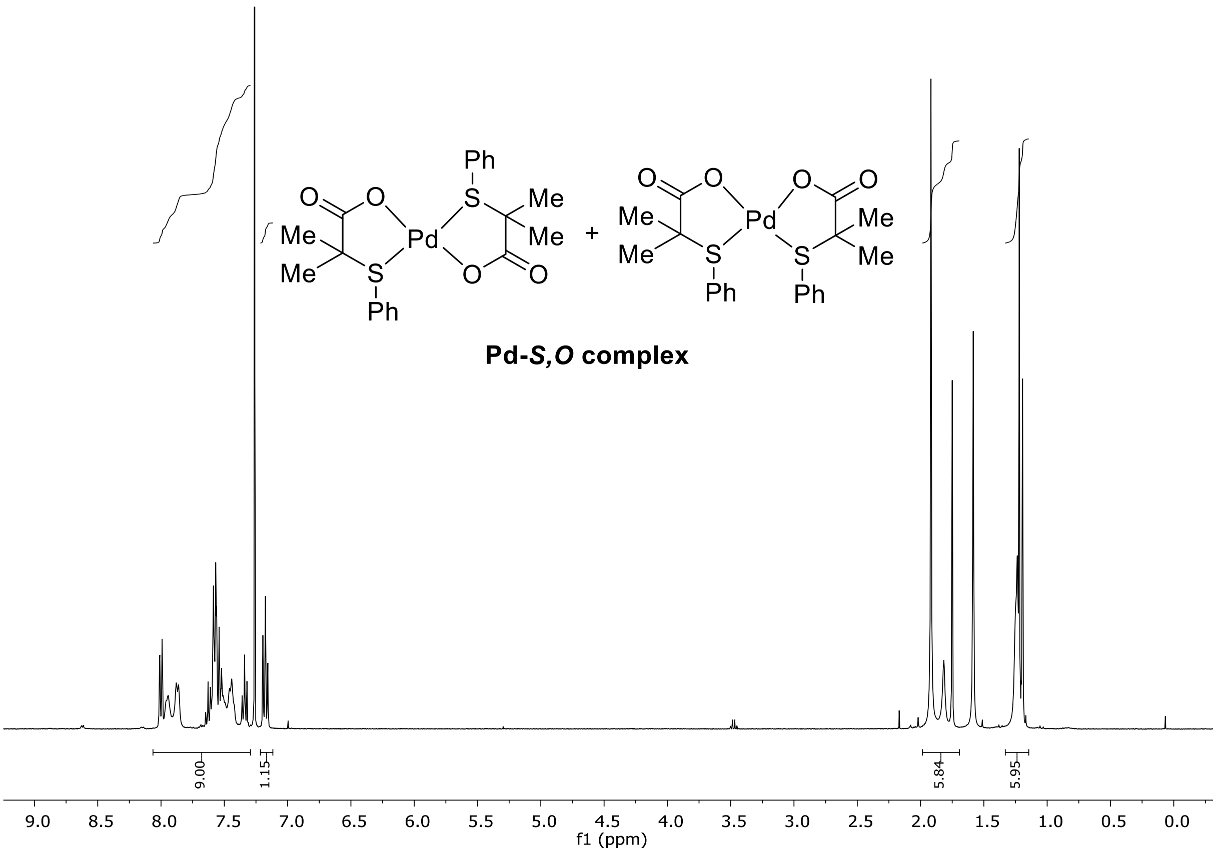

Supplementary Figure 277 H-NMR of compound Pd-*S,O*-complex. 300 MHz, $CDCl_3$, RT

Supplementary References

1 Naksomboon, K., Valderas, C., Gomez-Martinez, M., Alvarez-Casao, Y. & Fernandez-Ibanez, M. A. S,O-Ligand-Promoted Palladium-Catalyzed C–H Functionalization Reactions of Nondirected Arenes. *ACS Catal.* **7**, 6342-6346, doi:10.1021/acscatal.7b02356 (2017).

2 Wang, P. *et al.* Ligand-accelerated non-directed C–H functionalization of arenes. *Nature* **551**, 489-493, doi:10.1038/nature24632 (2017).

3 Devi, A. R. & Rajaram, S. Heck Reaction: Stereo Selective Synthesis of Trisubstituted Olefins: Useful Intermediates for Anthraquinone Carboxylic Acid Derivatives. *Synth. Commun.* **29**, 591-597, doi:10.1080/00397919908085807 (1999).

4 Göthlich, A. P. V. *et al.* Novel Chelating Phosphonite Ligands: Syntheses, Structures, and Nickel-Catalyzed Hydrocyanation of Olefins. *Organometallics* **27**, 2189-2200, doi:10.1021/om701140c (2008).

5 electrochemical: Xiao, Q., He, Q., Li, J. & Wang, J. 1,4-Diazabicyclo[2.2.2]octane-Promoted Aminotrifluoromethylthiolation of *α,β*-Unsaturated Carbonyl Compounds: N-Trifluoromethylthio-4-nitrophthalimide Acts as Both the Nitrogen and SCF_3 Sources. *Org. Lett.* **17**, 6090-6093, doi:10.1021/acs.orglett.5b03116 (2015).

6 Rai, P., Maji, K. & Maji, B. Photoredox/Cobalt Dual Catalysis for Visible-Light-Mediated Alkene–Alkyne Coupling. *Org. Lett.* **21**, 3755-3759, doi:10.1021/acs.orglett.9b01201 (2019).

7 Svec, R. L. & Hergenrother, P. J. Imidazotetrazines as Weighable Diazomethane Surrogates for Esterifications and Cyclopropanations. *Angew. Chem. Int. Ed.* **59**, 1857-1862, doi:https://doi.org/10.1002/anie.201911896 (2020).

8 Margrey, K. A., Levens, A. & Nicewicz, D. A. Direct Aryl C−H Amination with Primary Amines Using Organic Photoredox Catalysis. *Angew. Chem. Int. Ed.* **56**, 15644-15648, doi:https://doi.org/10.1002/anie.201709523 (2017).

9 Chatterjee, A. & König, B. Birch-Type Photoreduction of Arenes and Heteroarenes by Sensitized Electron Transfer. *Angew. Chem. Int. Ed.* **58**, 14289-14294, doi:https://doi.org/10.1002/anie.201905485 (2019).

10 Sheta, A. M. *et al.* Selective Electrosynthetic Hydrocarboxylation of *α,β*-Unsaturated Esters with Carbon Dioxide. *Angew. Chem. Int. Ed.* **60**, 21832-21837, doi:https://doi.org/10.1002/anie.202105490 (2021).

11 Battace, A., Lemhadri, M., Zair, T., Doucet, H. & Santelli, M. Palladium-Catalyzed Direct Arylation of Furans via C−H Functionalization at Low Catalyst Loadings. *Organometallics* **26**, 472-474, doi:10.1021/om0610243 (2007).

12 Riniker, S. & Landrum, G. A. Better Informed Distance Geometry: Using What We Know To Improve Conformation Generation. *J. Chem. Inf. Model.* **55**, 2562-2574, doi:10.1021/acs.jcim.5b00654 (2015).

13 Bannwarth, C. *et al.* Extended tight-binding quantum chemistry methods. *WIREs Comput. Mol. Sci.* **11**, e1493, doi:https://doi.org/10.1002/wcms.1493 (2021).

14 Grimme, S., Bannwarth, C. & Shushkov, P. A Robust and Accurate Tight-Binding Quantum Chemical Method for Structures, Vibrational Frequencies, and Noncovalent Interactions of Large Molecular Systems Parametrized for All spd-Block Elements (Z = 1–86). *J. Chem. Theory Comput.* **13**, 1989-2009, doi:10.1021/acs.jctc.7b00118 (2017).

15 Zhang, J. *et al.* Recent Developments in C–H Activation for Materials Science in the Center for Selective C–H Activation. *Molecules* **23**, 922 (2018).

16 N.P.R.A. Silva, M. E. *et al.* Redox potential and substituent effects in ferrocene derivatives: II. *J. Organomet. Chem.* **480**, 81-90, doi:https://doi.org/10.1016/0022-328X(94)87105-1 (1994).

17 Skurichina, M. & Duin, R. P. W. Bagging for linear classifiers. *Pattern Recogn.* **31**, 909-930, doi:https://doi.org/10.1016/S0031-3203(97)00110-6 (1998).

18 Breiman, L., Friedman, J. H., Olshen, R. A. & Stone, C. J. *Classification and regression trees*. (Routledge, 2017).

19 Geurts, P., Ernst, D. & Wehenkel, L. Extremely randomized trees. *Mach. Learn.* **63**, 3-42 (2006).

20 Friedman, J. H. Greedy function approximation: a gradient boosting machine. *Ann. Stat.*, 1189-1232 (2001).

21 Fix, E. & Hodges, J. L. Discriminatory analysis. Nonparametric discrimination: Consistency properties. *Int. Stat. Rev.* **57**, 238-247 (1989).

22 Cawley, G. C. & Talbot, N. L. Reduced rank kernel ridge regression. *Neural Process* **16**, 293-302 (2002).

23 Cortes, C. & Vapnik, V. Support-vector networks. *Mach. Learn.* **20**, 273-297 (1995).

24 Biau, G. Analysis of a random forests model. *J. Mach. Learn. Res.* **13**, 1063-1095 (2012).

25 García, C., García, J., López Martín, M. & Salmerón, R. Collinearity: revisiting the variance inflation factor in ridge regression. *J. Appl. Stat.* **42**, 648-661 (2015).

26 Zhang, Y. & Chen, L. A study on forecasting the default risk of bond based on XGBoost algorithm and over-sampling method. *Theor. econ. lett.* **11** (2021).

27 Pedregosa, F. *et al.* Scikit-learn: Machine Learning in Python. *J. Mach. Learn. Res.* **12**, 2825-2830 (2011).

28 Yang, G. *et al.* Pd(II)-Catalyzed meta-C–H Olefination, Arylation, and Acetoxylation of Indolines Using a U-Shaped Template. *J. Am. Chem. Soc.* **136**, 10807-10813, doi:10.1021/ja505737x (2014).

29 Sobhani, S., Hosseini Moghadam, H., Skibsted, J. & Sansano, J. M. A hydrophilic heterogeneous cobalt catalyst for fluoride-free Hiyama, Suzuki, Heck and Hirao cross-coupling reactions in water. *Green Chem.* **22**, 1353-1365, doi:10.1039/C9GC03455B (2020).

30 Vora, H. U., Silvestri, A. P., Engelin, C. J. & Yu, J.-Q. Rhodium(II)-Catalyzed Nondirected Oxidative Alkenylation of Arenes: Arene Loading at One Equivalent. *Angew. Chem. Int. Ed.* **53**, 2683-2686, doi:https://doi.org/10.1002/anie.201310539 (2014).

31 Babu, B. P., Meng, X. & Bäckvall, J.-E. Aerobic Oxidative Coupling of Arenes and Olefins through a Biomimetic Approach. *Chem. Eur. J.* **19**, 4140-4145, doi:https://doi.org/10.1002/chem.201300100 (2013).

32 Jadhav, S., Jagdale, A., Kamble, S., Kumbhar, A. & Salunkhe, R. Palladium nanoparticles supported on a titanium dioxide cellulose composite (PdNPs@TiO_2–Cell) for ligand-free carbon–carbon cross coupling reactions. *RSC Adv.* **6**, 3406-3420, doi:10.1039/C5RA20680D (2016).

33 Van Velthoven, N. *et al.* S,O-Functionalized Metal–Organic Frameworks as Heterogeneous Single-Site Catalysts for the Oxidative Alkenylation of Arenes via C–H activation. *ACS Catal.* **10**, 5077-5085, doi:10.1021/acscatal.0c00801 (2020).

34 Mészáros, R. *et al.* Exploiting a silver–bismuth hybrid material as heterogeneous noble metal catalyst for decarboxylations and decarboxylative deuterations of carboxylic acids under batch and continuous flow conditions. *Green Chem.* **23**, 4685-4696, doi:10.1039/D1GC00924A (2021).

35 Enquist, P.-A., Lindh, J., Nilsson, P. & Larhed, M. Open-air oxidative Heck reactions at room temperature. *Green Chem.* **8**, 338-343, doi:10.1039/B517152K (2006).

36 Cervi, A. *et al.* Gold(I)-Catalyzed Intramolecular Hydroarylation of Phenol-Derived Propiolates and Certain Related Ethers as a Route to Selectively Functionalized Coumarins and 2H-Chromenes. *J. Org. Chem.* **86**, 178-198, doi:10.1021/acs.joc.0c02011 (2021).

37 Shen, Y., Liu, G., Zhou, Z. & Lu, X. Rhodium(III)-Catalyzed C–H Olefination for the Synthesis of ortho-Alkenyl Phenols Using an Oxidizing Directing Group. *Org. Lett.* **15**, 3366-3369, doi:10.1021/ol4014188 (2013).

38 Wang, Y.-P. & Lee, H. M. Catalytic application of zwitterionic palladium complexes in Mizoroki–Heck reactions using ionic liquid as solvent. *J. Organomet. Chem.* **791**, 90-98, doi:https://doi.org/10.1016/j.jorganchem.2015.05.020 (2015).

39 Sun, Z.-M., Zhang, J. & Zhao, P. Rh(I)-Catalyzed Decarboxylative Transformations of Arenecarboxylic Acids: Ligand- and Reagent-Controlled Selectivity toward

Hydrodecarboxylation or Heck−Mizoroki Products. *Org. Lett.* **12**, 992-995, doi:10.1021/ol100001b (2010).

40 Yang, W. *et al.* Palladium-catalyzed aerobic oxidative double allylic C–H oxygenation of alkenes: a novel and straightforward route to *α,β*-unsaturated esters. *Chem. Commun.* **51**, 9575-9578, doi:10.1039/C5CC02277K (2015).

41 Li, X. *et al.* Design, synthesis and antibacterial activity of cinnamaldehyde derivatives as inhibitors of the bacterial cell division protein FtsZ. *Eur. J. Med. Chem.* **97**, 32-41, doi:https://doi.org/10.1016/j.ejmech.2015.04.048 (2015).

42 Kubota, A., Emmert, M. H. & Sanford, M. S. Pyridine Ligands as Promoters in PdII/0-Catalyzed C–H Olefination Reactions. *Org. Lett.* **14**, 1760-1763, doi:10.1021/ol300281p (2012).

43 Hayouni, S., Ferlin, N. & Bouquillon, S. High catalytic and recyclable systems for heck reactions in biosourced ionic liquids. *Mol. Catal.* **437**, 121-129, doi:https://doi.org/10.1016/j.mcat.2017.05.007 (2017).

44 Ding, Q.-D. *et al.* Heterogeneous catalytic decarboxylative sulfonylation of cinnamic acids with sodium benzene sulfinates over a manganese(II)-based rod-shaped metal-organic framework catalyst. *Catal. Commun.* **127**, 69-74, doi:https://doi.org/10.1016/j.catcom.2019.05.006 (2019).

45 Xue, J.-F. *et al.* Manganese(iii)-mediated alkenyl Csp^2–P bond formation from the reaction of *β*-nitrostyrenes with dialkyl phosphites. *Org. Biomol. Chem.* **13**, 4896-4902, doi:10.1039/C5OB00404G (2015).

46 Reddy, S. M. K., Kothandapani, J., Sengan, M., Veerappan, A. & Selva Ganesan, S. Exploring the influence of designer surfactant hydrophobicity in key C–C/C–N bond forming reactions. *Mol. Catal.* **465**, 80-86, doi:https://doi.org/10.1016/j.mcat.2019.01.006 (2019).

47 Yan, Y. *et al.* Selective and facile synthesis of α,β-unsaturated nitriles and amides with N-hydroxyphthalimide as the nitrogen source. *Tetrahedron Lett.* **59**, 2793-2796, doi:https://doi.org/10.1016/j.tetlet.2018.06.011 (2018).

48 Zhang, W. & Lu, Y. Automation of Fluorous Solid-Phase Extraction for Parallel Synthesis. *J. Comb. Chem.* **8**, 890-896, doi:10.1021/cc0601130 (2006).

49 Shah, S. S. *et al.* Organophotoredox-Mediated Amide Synthesis by Coupling Alcohol and Amine through Aerobic Oxidation of Alcohol. *Chem. Eur. J.* **26**, 3703-3708, doi:https://doi.org/10.1002/chem.201904924 (2020).

50 Elenkov, I. J., Todorova, D. I., Bankova, V. S. & Milkova, T. S. Synthesis of Steryl Esters of Phenolic Acids by a Heterogeneous Wittig Reaction. *J. Nat. Prod.* **58**, 280-283, doi:10.1021/np50116a021 (1995).

51 Desai, S. B., Argade, N. P. & Ganesh, K. N. Remarkable Chemo-, Regio-, and Enantioselectivity in Lipase-Catalyzed Hydrolysis: Efficient Resolution of (±)-threo-Ethyl 3-(4-Methoxyphenyl)-2,3-diacetoxypropionate Leading to Chiral Intermediates of (+)-Diltiazem. *J. Org. Chem.* **61**, 6730-6732, doi:10.1021/jo960835l (1996).

52 Xiang, L., Yang, K. & Song, Q.-L. Pd-catalyzed ortho-olefination of aromatic acetyl esters. *Chin. Chem. Lett.* **28**, 517-520, doi:https://doi.org/10.1016/j.cclet.2016.11.023 (2017).

53 Frisch, M. *et al.* (Gaussian, Inc. Wallingford, CT, 2016).

54 Adamo, C. & Barone, V. Toward reliable density functional methods without adjustable parameters: The PBE0 model. *J. Chem. Phys.* **110**, 6158-6170 (1999).

55 Ernzerhof, M. & Scuseria, G. E. Assessment of the Perdew–Burke–Ernzerhof exchange-correlation functional. *J. Chem. Phys.* **110**, 5029-5036 (1999).

56 Grimme, S., Ehrlich, S. & Goerigk, L. Effect of the damping function in dispersion corrected density functional theory. *J. Comput. Chem.* **32**, 1456-1465 (2011).

57 Grimme, S., Antony, J., Ehrlich, S. & Krieg, H. A consistent and accurate ab initio parametrization of density functional dispersion correction (DFT-D) for the 94 elements H-Pu. *J. Chem. Phys.* **132**, 154104 (2010).

58 Schäfer, A., Huber, C. & Ahlrichs, R. Fully optimized contracted Gaussian basis sets of triple zeta valence quality for atoms Li to Kr. *J. Chem. Phys.* **100**, 5829-5835 (1994).

59 Weigend, F. Accurate Coulomb-fitting basis sets for H to Rn. *Phys. Chem. Chem. Phys.* **8**, 1057-1065 (2006).

60 Weigend, F. & Ahlrichs, R. Balanced basis sets of split valence, triple zeta valence and quadruple zeta valence quality for H to Rn: Design and assessment of accuracy. *Phys. Chem. Chem. Phys.* **7**, 3297-3305 (2005).

61 Dolg, M., Wedig, U., Stoll, H. & Preuss, H. Energy‐adjusted abinitio pseudopotentials for the first row transition elements. *J. Chem. Phys.* **86**, 866-872 (1987).

62 Martin, J. M. L. & Sundermann, A. Correlation consistent valence basis sets for use with the Stuttgart–Dresden–Bonn relativistic effective core potentials: The atoms Ga–Kr and In–Xe. *J. Chem. Phys.* **114**, 3408-3420, doi:10.1063/1.1337864 (2001).

63 Zhao, Y. & Truhlar, D. G. Design of density functionals that are broadly accurate for thermochemistry, thermochemical kinetics, and nonbonded interactions. *J. Phys. Chem* **109**, 5656-5667 (2005).

64 Chai, J.-D. & Head-Gordon, M. Long-range corrected hybrid density functionals with damped atom–atom dispersion corrections. *Phys. Chem. Chem. Phys.* **10**, 6615-6620 (2008).

65 Caldeweyher, E., Bannwarth, C. & Grimme, S. Extension of the D3 dispersion coefficient model. *J. Chem. Phys.* **147**, 034112 (2017).

66 Caldeweyher, E. *et al.* A generally applicable atomic-charge dependent London dispersion correction. *J. Chem. Phys.* **150**, 154122 (2019).

67 Marenich, A. V., Cramer, C. J. & Truhlar, D. G. Universal solvation model based on solute electron density and on a continuum model of the solvent defined by the bulk dielectric constant and atomic surface tensions. *J. Phys. Chem. B* **113**, 6378-6396 (2009).
