# Supplementary material for: Electrocatalyzed direct arene alkenylations without directing groups for selective late-stage drug diversification
Source: Nat Commun. 2023 Jul 15;14:4224. doi: 10.1038/s41467-023-39747-0 (PMC10349852; doi:10.1038/s41467-023-39747-0)
Supplement: Supplementary file 3 — Description of Additional Supplementary Files [file 41467_2023_39747_MOESM3_ESM.docx]

File Name: Supplementary Data 1
Description: Cartesian Coordinates used for DFT calculations
